# Turtle species and ecology drive carapace microbiome diversity in three seasonally interconnected wetland habitats

**DOI:** 10.1099/acmi.0.000682.v3

**Published:** 2024-01-12

**Authors:** Matthew Parks, Jun Sheng Lee, Kassandra Camua, Ethan Hollender

**Affiliations:** ^1^​ Department of Biology, University of Central Oklahoma, 100 N University Drive, Edmond, Oklahoma 73034, USA; ^2^​ DNA Reference Lab, 5282 Medical Dr. Suite 312, San Antonio, Texas 78229, USA; ^3^​ Department of Biological Sciences, 601 Science Engineering Hall, University of Arkansas, Fayetteville, Arkansas 72701, USA

**Keywords:** freshwater, turtles, 16S rRNA gene amplicon sequencing, 18S rRNA gene amplicon sequencing, metabarcoding

## Abstract

Different species of freshwater turtles exhibit primary behaviours ranging from aerial basking to benthic bottom-walking, cycle between wet and dry conditions at different time intervals, and undertake short-distance overland movements between aquatic habitats. These behaviours in turn may impact the accumulation of microbes on external shell surfaces of turtles and provide novel niches for differentiation of microbial communities. We assessed microbial diversity using 16S and 18S rRNA metabarcoding on carapace surfaces of six species of freshwater turtles residing in three adjacent and seasonally interconnected wetland habitats in southeast Oklahoma (United States). Communities were highly diverse, with nearly 4200 prokaryotic and 500 micro-eukaryotic amplicon sequence variants recovered, and included taxa previously reported as common or differentially abundant on turtle shells. The 16S rRNA alpha diversity tended to be highest for two species of benthic turtles, while 18S rRNA alpha diversity was highest for two basking and one shallow-water benthic species. Beta diversity of communities was more strongly differentiated by turtle species than by collection site, and ordination patterns were largely reflective of turtle species’ primary habits (i.e. benthic, basking, or benthic-basking). Our data support that freshwater turtles could play a role in microbial ecology and evolution in freshwater habitats and warrant additional exploration including in areas with high native turtle diversity and inter-habitat turtle movements.

## Data Summary

All sequence data associated with this study have been deposited in fastq format in NCBI SRA (BioProject ID: PRNJA975189).

Full R coding and outputs for all analyses are available in the supplementary material, available in the online version of this article R markdown (pdf) file.

The authors confirm all supporting data, code and protocols have been provided within the article or through supplementary data files.

**Impact Statement**

Microbial diversity is critical to ecosystem functioning, including in freshwater systems like ponds, lakes, creeks, and rivers. Turtles make outsized contributions to ecosystem biomass in freshwater systems and often accumulate diverse microbial assemblages on their shells, yet relatively little research to date has focused on turtle-associated microbial communities in native habitats. We assessed microbial diversity on the carapace surfaces of six species of freshwater turtles in three adjacent and seasonally connected wetland habitats in southeast Oklahoma (USA) by metataxonomics. As with previous work on turtle shell-associated microbial communities, we recovered highly diverse prokaryotic and eukaryotic communities from our sampled turtle species including taxa previously reported as abundant on both North American and Australian freshwater turtle species. Unlike previous studies, however, our work provides novel insight into the differentiation of microbial communities by turtle species and, by proxy, the ecological habits of freshwater turtles. Our work supports that the primary habit of each turtle species is correlated to the composition of the microbial community carried on its shells. Basking turtles are differentiated from benthic species, including for the prevalence of photosynthetic eukaryotic microbes, and communities on the shells of a single basking-benthic turtle species are intermediate to those of basking and benthic turtles. We also know that our sampled turtle species may traverse short to medium-length overland distances between habitats. Taken together, our work supports that different turtle species may play impactful roles in freshwater systems as microbial accumulators and vectors within and between freshwater habitats, and so could influence longer-term population genetic and evolutionary trends of diverse microbial species.

## Introduction

Microbial communities associated with aquatic and amphibious host environments are important areas of inquiry, due to unique conditions they possess relative to terrestrial counterparts. For example, aquatic-associated microbial communities may be more stable due to dispersal effects and lower disruption of chemical and physical parameters [1], and the presence of keystone microbial taxa [2]. Alternatively, these systems may still feature dynamic patterns of change in both native [3, 4] and controlled [5, 6] settings. Similarly, amphibious microbiomes present unique opportunities to study microbial adaptation, since exposure to diametric habitats (i.e. wet-dry, light-dark) may increase associated microbial diversity [7, 8]. Amphibious microbiomes have been shown to exhibit sensitivity to changes in moisture and temperature [9, 10], and wet-dry cycling may also increase bacterial fitness, including through resistance to antibiotic compounds [11]. Amphibious hosts may also capture a historic record of microbial communities, for example mollusks can serve as microbial ‘archives’ by capturing microbial communities within mineralized layers of the shell [12].

Across amphibious animals, many species of turtles are recognized for microbial accumulation on external shell surfaces. This growth is often extensive, with the result that some freshwater species are colloquially referred to as ‘mossbacks’ [13]. Previous work has demonstrated that turtle shell microbial communities are both diverse and variable [14–19]. Considering also that turtles are relatively ubiquitous to temperate and tropical freshwater aquatic habitats, can reach some of the highest population densities known to vertebrates [20], and have a propensity for overland migration, turtles and their shells present a relatively unique system of both accumulator and potential vector for aquatic and amphibious microbial systems. Three published studies provide insight into shell-associated microbial communities of freshwater turtles through metabarcoding strategies. McKnight *et al*. [17] demonstrated variable prokaryotic microbial communities across shell locations of the Australian Krefft’s river turtle (*Emydura krefftii*), influenced by the presence or absence of macroalgae. Parks *et al*. [18] used sampling across habitats and geographic distance to show consistent differentiation between carapace, plastron and submerged environmental prokaryotic and eukaryotic microbial communities in the widely distributed red-eared slider (*Trachemys scripta*), as well as differences across geographic distance. More recently, White *et al*. [19] documented the influence of habitat quality in shell-associated prokaryotic microbial community assemblage in the western pond turtle (*Emys marmorata*) and overlap in bacterial communities between *E. marmorata* and *T. scripta*. In all three cases [17–19], documented microbial communities included hundreds of microbial genera and amplicon sequence variants (ASVs). Prokaryotic carapace communities of the studied turtle species featured high proportions of Bacteroidia, Gammaproteobacteria, Alphaproteobacteria, and Oxyphotobacteria classes [17–19]. Some bacterial taxa with known extremophilic properties, for example Deinococcus-Thermus bacteria, were also found common to the geographically distant carapace communities of *E. krefftii* and *T. scripta* [18], and were common to *T. scripta* and *E. marmorata* [18, 19].

A fuller understanding of influences on microbial diversity and community assembly associated with shells of freshwater turtles would be beneficial to both the understanding of turtle ecology and microbial ecology. This includes the interplay between microbial community and host organism fitness, which could benefit turtle conservation efforts, including for captivity-based programmes [21, 22]. A fundamental question that can be addressed is, what is the influence of turtle species on shell-associated microbial communities? Answering this question could lend insight into the degree to which turtle shells are passive or selective accumulators of microbial taxa, as well as the influence of differing species’ ecological habits (for example, basking vs. benthic) on microbial accumulation. It is additionally known that microbial community assemblage results from a combination of stochastic and deterministic factors, including important roles for dispersal, drift, selection, and diversification [23–26], and may be further influenced by disturbance [27]. Importantly, turtles have the potential to influence microbial community assemblage through each of these factors. For example, overland movements combined with repeated wet-dry cycling may result in influence on microbial community dispersal, drift, and selection. Repeated ‘re-setting’ of microbial communities through shedding of scutes [28] and physical agitation between shells and hard submerged or exposed substrates results in community disruption that could further provide selective opportunities in short- and long-term microbial community adaptation.

The southeast region of the United States (SE US) holds the second highest level of turtle diversity in the world, behind only southeast Asia [29, 30]. As an example, this region includes the Mobile River basin, which hosts 18 turtle species and is ranked as the second-most turtle-diverse river basin in the world [29]. Across the SE US, there are many additional habitats hosting diverse co-habiting turtle species, providing opportunity to test for differences in species’ shell-associated microbial community while minimizing the influence of geography. In the present study, we focused on a set of small, seasonally interconnected wetland habitats with high turtle diversity in southeast Oklahoma, an area included within the broader SE US. At least ten species of freshwater turtles are present in these wetlands, ranging from basking turtles like red-eared sliders and river cooters (*Pseudemys concinna*), to predominantly benthic species, including common snapping turtle and the Eastern mud turtle (*Chelydra serpentina* and *Kinosternon subrubrum*, respectively). We collected microbial communities from carapace surfaces in these wetlands in early summer across 2 years, from multiple specimens of six different turtle species, and delineated corresponding patterns in prokaryotic and eukaryotic microbial diversity. The main goal of this work was to assess the influence of turtle species identity (and, by proxy, ecology) on patterns of shell-associated microbial community assemblage. Our results provide a unique context for shell-associated microbial communities in wetland ecosystems within a broader and globally important turtle diversity hotspot.

## Methods

### Sample sites and collections

**Fig. 1.**
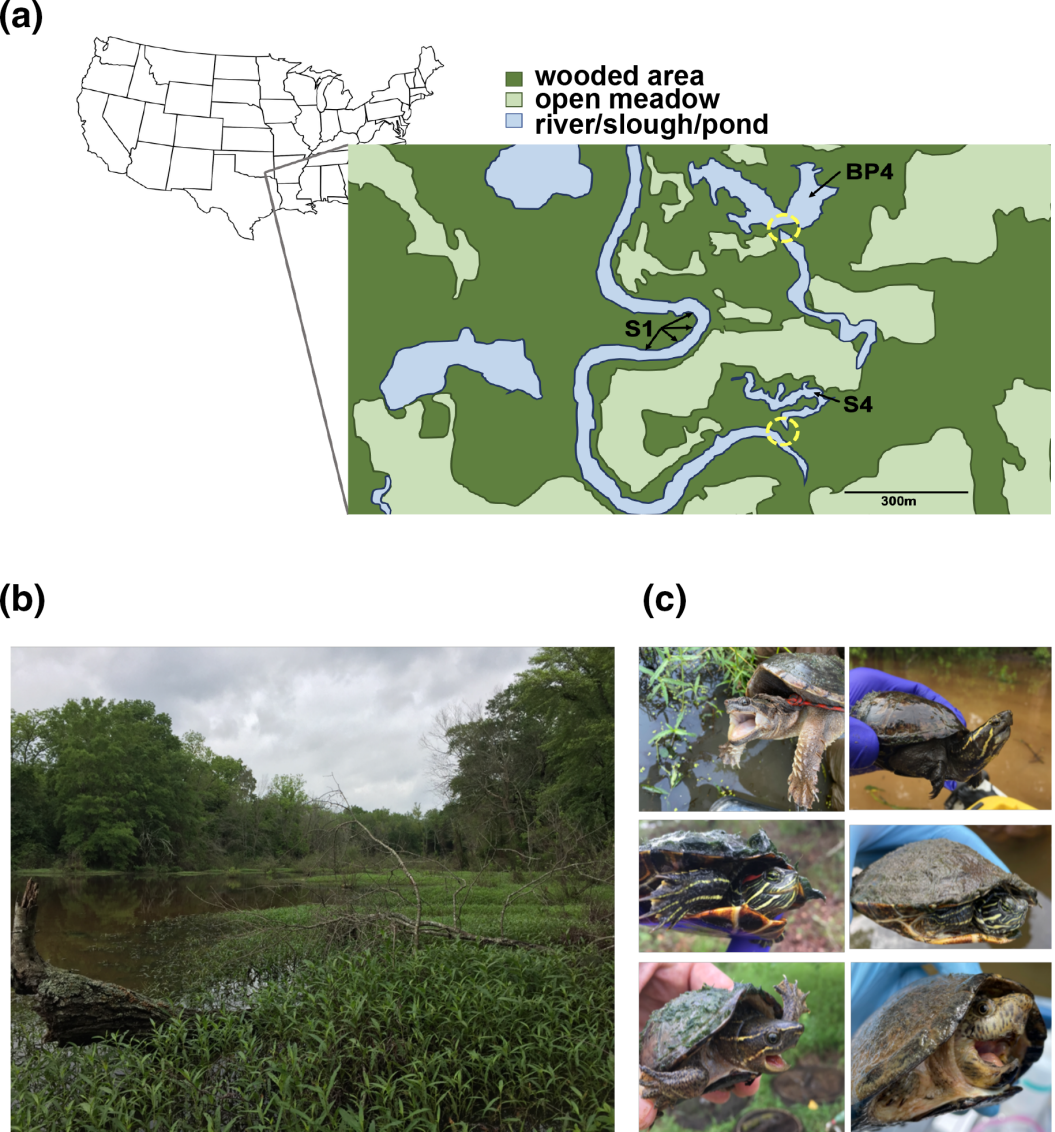
Sample site locations and turtle species in the present study. (**a**) Sites BP4 and S4 were sampled throughout the entire indicated bodies of water; site S1 was sampled along the indicated range. Dashed yellow circles at lower sides of sites BP4 and S4 indicate locations of beaver dam (BP4) and flood-stage connection to adjacent waterways (BP4, **s4**), respectively. (**b**) Example of sampled habitat, partial view of site BP4. (**c**) Turtle species sampled for this study. Clockwise from top left: common snapping turtle (*Chelydra serpentina*), Eastern mud turtle (*Kinosternon subrubrum*), river cooter (*Pseudemys concinna*), razorback musk turtle (*Sternotherus carinatus*), common musk turtle (*Sternotherus odoratus*), red-eared slider (*Trachemys scripta*).

Microbial samples were collected from turtles captured at three sites within a set of closely located and seasonally interconnected wetlands (i.e. physically connected during seasonal high-water events) in SE Oklahoma (Fig. 1a, b). These sites included a backflow slough of a small river (site S1), seasonal overflow from the backflow slough (site S4), and a creek-fed beaver pond (site BP4). These wetlands and the surrounding habitat are in relatively stable condition, and are typical of lowland areas in this region, including bordering mixed-species deciduous forest and adjacent open, grassy meadows. All three sites were located on a privately-owned property used primarily for low-density cattle grazing and hayfield (some fields mowed for hay 2–3 times per year). There are no row-crops, paved roads/paths, or major pieces of construction in the immediate vicinity of these wetlands, and each body of water has at least some degree of wooded buffer surrounding it.

Turtles were trapped in late May 2021 and early June 2022, using mostly submerged hoop net traps baited with tins of sardines. Captured turtle plastron lengths were measured using 300 mm dial callipers (Anytime Tools, Granada Hills, California), and sex was determined primarily by assessing sexually dimorphic tail morphology, supported as needed by a suite of other species-specific sexually dimorphic traits [31]. For microbial scrape samples, the thickest growth on a turtle’s carapace surface was first briefly rinsed with distilled water, then collected using an ethanol-sterilized dental calculus scraper before preservation in 100 % ethanol. Preserved samples were kept as cool as possible over each 2–3 day field collection period, before returning to the lab where they were stored at −20 °C until DNA extractions. In total, single carapace scrape samples were collected from 84 turtles representing the following six species: *Chelydra serpentina* (common snapping turtle), *Kinosternon subrubrum* (Eastern mud turtle), *Pseudemys concinna* (river cooter), *Sternotherus carinatus* (razor-backed musk turtle), *Sternotherus odoratus* (common musk turtle) and *Trachemys scripta* (red-eared slider) (Fig. 1c). Sample sizes ranged from three samples per species (*Chelydra serpentina*) to 25 samples per species (*Sternotherus odoratus*), and not all species were able to be collected from all three sites (Table 1). For efficiency, species names are abbreviated in figures by four letter codes consisting of the first two letters of each species’ genus name and its specific epithet (Table 1).

**Table 1.** Species and collection information for turtle carapace samples in the present study. Four letter codes used in the text for each species are also shown, consisting of the first two letters of genus name and specific epithet. *Dominant habit information retrieved from [101]

Turtle species (abbreviation)	Dominant habit(s)*	no. of individuals sampled	Sites collected	Year(s) collected
*Chelydra serpentina* (CHSE)	benthic	3	BP4	2022
*Kinosternon subrubrum* (KISU)	benthic	14	BP4, S4	2021, 2022
*Pseudemys concinna* (PSCO)	basking	12	BP4	2021, 2022
*Sternotherus carinatus* (STCA)	basking/benthic	8	S1	2022
*Sternotherus odoratus* (STOD)	benthic	25	BP4, S1, S4	2021, 2022
*Trachemys scripta* (TRSC)	basking	22	BP4, S1, S4	2021, 2022

### DNA extraction and PCR amplifications

DNA extractions were performed using a Nucleospin Plant II DNA extraction kit (Takara Bio USA, Mountain View, California), with modifications as described in Parks *et al*. [18]. Subsequent PCR amplifications for the V3–V4 hypervariable regions of the 16S rRNA gene and the V8–V9 hypervariable regions of the 18S rRNA gene were also performed following the same protocols as Parks *et al*. [18]. This PCR amplification method consists of two sequential PCR reactions for each amplified locus (i.e. ‘step 1’ and ‘step 2’ PCR amplifications). Step 1 PCR reactions are performed in triplicate and pooled for each sample. Step 1 PCR pools for each sample are then used as input for step 2 PCR reactions. Tailed primers are used in both rounds of PCR, to allow for addition of unique 5′-index sequence and Illumina-required sequence motifs, resulting in ready-to-sequence amplicon libraries. Only slight modifications were made to the protocol of Parks *et al*. [19], including that a standard thermocycler protocol was used for step 1 PCR amplifications for both 16S and 18S rRNA gene amplifications (95 °C for 2 minutes / 30 cycles of 95 °C for 15 s, 58 °C for 30 s, 68 °C for 30 seconds / final extension at 68 °C for 5 min).

### 16S and 18S rRNA gene metabarcode sequencing

Step 2 PCR reactions were pooled separately for 16S and 18S rRNA gene amplifications and quantified using a NanoDrop spectrophotometer (ThermoFisher Scientific). The 16S rRNA gene and 18S rRNA gene pools were then combined approximately equimolarly and diluted and denatured prior to sequencing following standard Illumina protocols. All samples were sequenced on an Illumina MiSeq (Illumina, Inc., San Diego, California) at the University of Central Oklahoma, using 300 base-pair (bp) paired-end sequencing with Illumina MiSeq 600-cycle V3 kits. Samples from 2021 and 2022 were sequenced collectively by year, on two different sequencing runs.

### Bioinformatic processing and taxonomic assignments

Raw sequence data in fastq format was demultiplexed using custom Unix scripting, and trimmed of adapter and primer sequences using Cutadapt v. 2.10 [32] with minimum length cutoff of 100 bp for forward and reverse reads. Subsequent visualization, denoising and taxonomic assignment were carried out using QIIME2 v. 2021.4 [33], and largely followed the ‘moving pictures’ tutorial guidelines [34]. For QIIME2 processing and subsequent analyses, sequence data from 2021 and 2022 sequencing runs were combined for 16S rRNA gene libraries and for 18S rRNA gene libraries. Samples were first denoised using DADA2 [35]. Based on quality score distributions and levels of chimerism, only the forward reads of all samples were used in data analysis, and these were trimmed to maximum read lengths of 200 bp. Minimum cutoffs for feature frequency (at least 100 reads among samples) and presence (presence in at least two samples) were applied separately for 16S and 18S rRNA gene sample sets. Taxonomy was assigned through the Naïve Bayes classifier (classify-sklearn) and the feature-classifier QIIME2 plugin. Assignments were based on the 16S and 18S rRNA gene Silva v. 138.1 SSU Ref NR99 databases [36], which were trimmed to amplicons using the 16S and 18S forward and reverse step 1 PCR primers following the ‘microbiome_helper’ guidelines [37]. Any features recovered as mitochondria, chloroplast, or Eukaryota were removed from 16S rRNA gene assignments, while any features recovered as mitochondria, chloroplast, bacteria, vertebrate or arthropod were removed from 18S rRNA gene assignments. Both 16S and 18S rRNA gene samples were also filtered for laboratory contamination based on feature counts in associated negative controls using recommended procedures in microDecon [38]. Estimates of taxon counts at the levels of genus, family, and order, was done in a syntactically conservative manner to avoid inflation due to redundancy (for example, ‘g__Aminicenantales’ was considered the same taxon as ‘g__Aminicenantales_soil’). Rarefaction curves were used in QIIME2 to check for potential impact of sequencing depth on recovery of taxonomic diversity across samples.

### Diversity and statistical analyses

Subsequent statistical analyses and visualization were performed in RStudio (v. 2022.07.02), primarily with sample metadata, microbial taxon names and abundance data, and corresponding phylogenetic tree loaded as objects in phyloseq v. 1.42.0 [39]. Complete R markdowns for statistical analyses are available in supplementary material. Rarefaction curves suggested all 16S and 18S rRNA gene carapace samples had sufficient depth to recover virtually all assigned taxonomy, however we included sample read depth as a factor in statistical testing to assess any impact read depth might have on our results. Read counts were transformed to proportions per sample (i.e. total sum normalization) for abundance-based diversity metrics, prior to calculating distances [38]. Initial stacked barplots for taxonomy were prepared using microViz v. 0.10.6 [40]. Overlap in ASV taxonomic assignments between turtle species and between collection sites was assessed using UpSetR v. 1.4.0 in place of typical Venn diagrams due to a high number of intersections when comparing turtle species’ microbial communities [41].

Three different measures of alpha diversity (number of observed features, Shannon diversity, Faith’s phylogenetic diversity) were estimated using the R package phyloseq v. 1.42.0. Multiple regression modelling followed by ANOVA was performed in R to assess the significance and contribution of various factors to alpha diversity, with model assumptions checked visually through residual plots generated in easystats v. 0.6.0 [42]. In initial models, alpha diversity measures were each regressed against turtle species, collection site, sex of individual (male, female, or juvenile), collection year, sample read depth and plastron length. Both the number of observed features and sample read depth were subsequently adjusted to log_10_ scale to improve model fit. Moderate to high levels of collinearity were recovered for species identity and plastron length, so the same regression models were then applied within each turtle species (leaving out species identity as a factor) to specifically assess the impacts of plastron length on alpha diversity measures. Within each turtle species, plastron length was minimally impactful, with a significant effect only for one metric in one species (18S Faith’s phylogenetic diversity in *Sternotherus odoratus*). Based on these results, plastron length was not included as a factor in subsequent testing. The final model tested was: alpha diversity measure ~Species+ Site + Sex+log_10_(read depth) + Year. Outliers were identified using standardized residuals in olsrr v. 0.5.3 [43] and final regression and ANOVA analyses were performed both with and without inclusion of identified outlier samples. Significance of pairwise comparisons was assessed with *p*-values adjusted using the R package emmeans v. 1.8.2 [44] through post-hoc Tukey’s HSD correction, a robust and conservative test recommended for unbalanced sampling design [45].

Trends in beta diversity were visualized using both heatmaps and ordination plots assembled in phyloseq v. 1.42.0 and vegan v. 2.6.4 [46], respectively. Beta diversity was quantified through four measures: Bray-Curtis, Jaccard, UniFrac, and weighted UniFrac metrics. Beta diversity measures were subsequently tested for significant differences in three metrics using vegan v. 2.6.4 : 1) dispersion (an estimate of homogeneity of variance), 2) centroid location (the location of the centre of a cluster of samples), and 3) analysis-of-similarity (ANOSIM) (a metric ranging from 0 to 1 and indicating whether groups are discretely separated; values closer to one indicate more discrete clustering). Beta dispersion effects were measured through ANOVA, with 999 permutations and with post-hoc Tukey’s correction for *p*-values. Differences in species’ centroid locations were tested using PERMANOVA using the adonis2() function with setting by=‘margin’ to negate effects of model order, and with Benjamini-Hochberg correction for *p*-values [47]. In PERMANOVA analyses, species, site, sex, log_10_(read depth) and year of collection were treated as factors. ANOSIM calculations were performed with 999 permutations.

## Results

### Sequencing output and taxonomic assignments

Raw forward sequencing reads ranged from 14 153 to 121 835 reads (average=79 907±20 970 standard deviation) per carapace sample for 16S rRNA gene amplicon sequences, and from 43 496 to 174 528 reads (average=87 904±24 063) for 18S rRNA gene amplicon sequences. Taxonomic assignments supported diverse microbial communities, including 4177 16S rRNA gene ASVs and 489 18S rRNA gene ASVs across carapace shell samples, representing 202 prokaryotic and 50 eukaryotic orders (Table 2).

**Table 2.** Counts of assigned microbial taxonomy for 16S and 18S rRNA gene carapace samples. Counts are shown for unique ASVs, genera, families and orders

Taxonomic level	16S rRNA gene	18S rRNA gene
ASV	4177	489
Genus	443	102
Family	307	57
Order	202	50

**Fig. 2.**
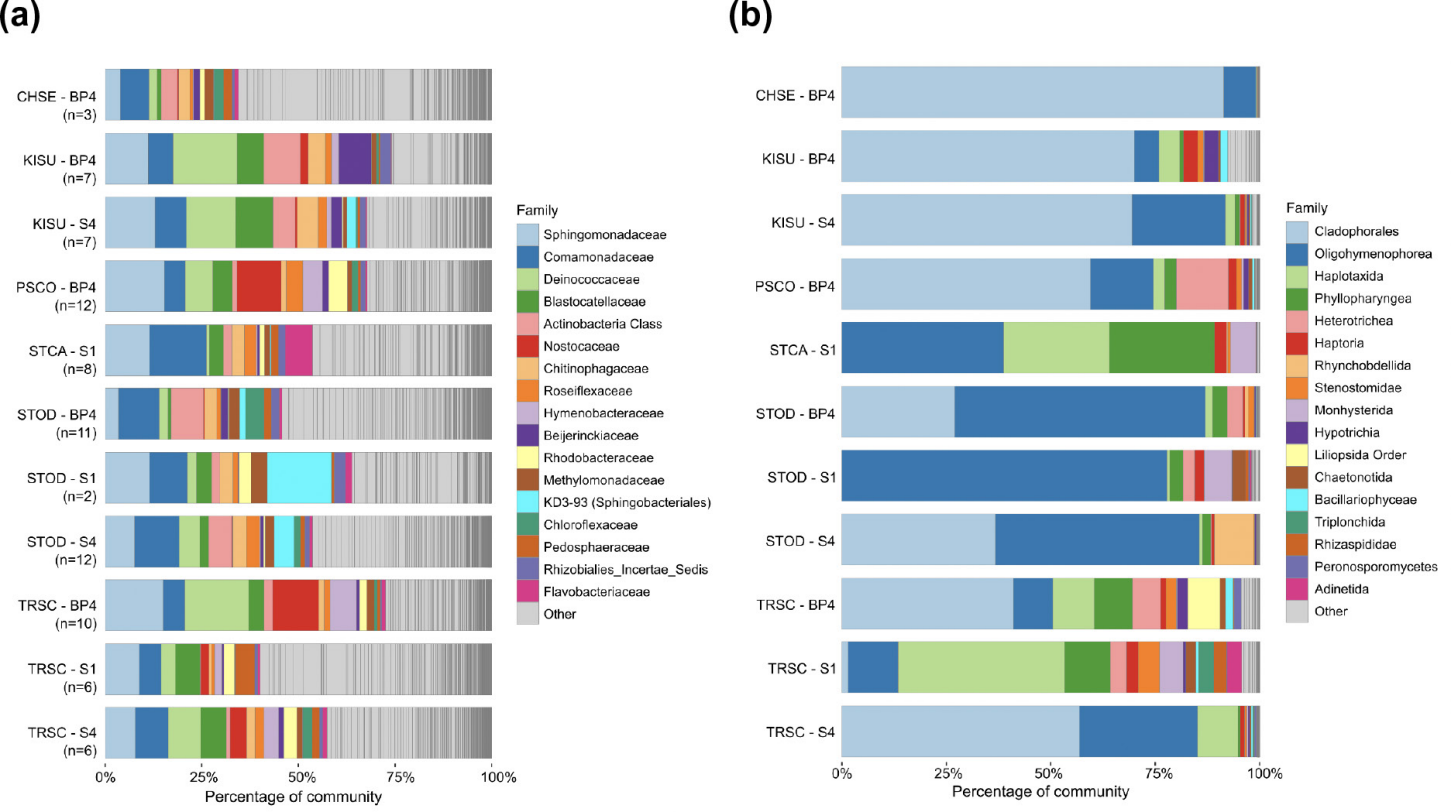
Stacked barplots for prokaryotic and eukaryotic microbial diversity, with samples grouped by turtle species and collection site. Taxonomy is shown at the family level for carapace samples: (a) 16S rRNA gene samples; (**b**) 18S rRNA gene samples. The most prevalent 17 families are indicated by coloured segments for both (a) and (b); the remainder of less prevalent taxa are indicated as ‘other’ and are shown in grey. Number of samples per species-site combination is indicated by ‘(n=)” in (a) and is the same as for (b).

For prokaryotic communities, the most abundant taxa with classification available at the family level were Sphingomonadaceae (class Alphaproteobacteria) (range=3.9–15.3 % abundance per turtle species, 9.1–10.7 % per site), Comamonadaceae (class Betaproteobacteria) (range=5.4–14.8 % abundance per turtle species, 7.1–10.8 % per site), Deinococcaceae (class Deinococci) (range=0.8–14.6 % abundance per turtle species, 2.1–9.3 % per site), and Blastocatellaceae (class Blastocatellia) (range=1.1–8.3 % abundance per turtle species, 3.7–5.4 % per site) (Fig. 2a). For eukaryotic communities, the most abundant taxa with classification available at the family level were Cladophorales (class Ulvophyceae) (range=0.0–91.5 % abundance per turtle species, 0.6–50.8 % per site), Oligohymenophorea (class Oligohymenophorea) (range=7.6–56.0 % abundance per turtle species, 23.3–36.4 % per site), Haplotaxida (class Clitellata) (range=0.3–25.4 % abundance per turtle species, 3.4–27.7 % per site), and Phyllopharyngea (class Phyllopharyngea) (range=0.0–25.1 % abundance per turtle species, 1.3–17.1 % per site) (Fig. 2b).

**Fig. 3.**
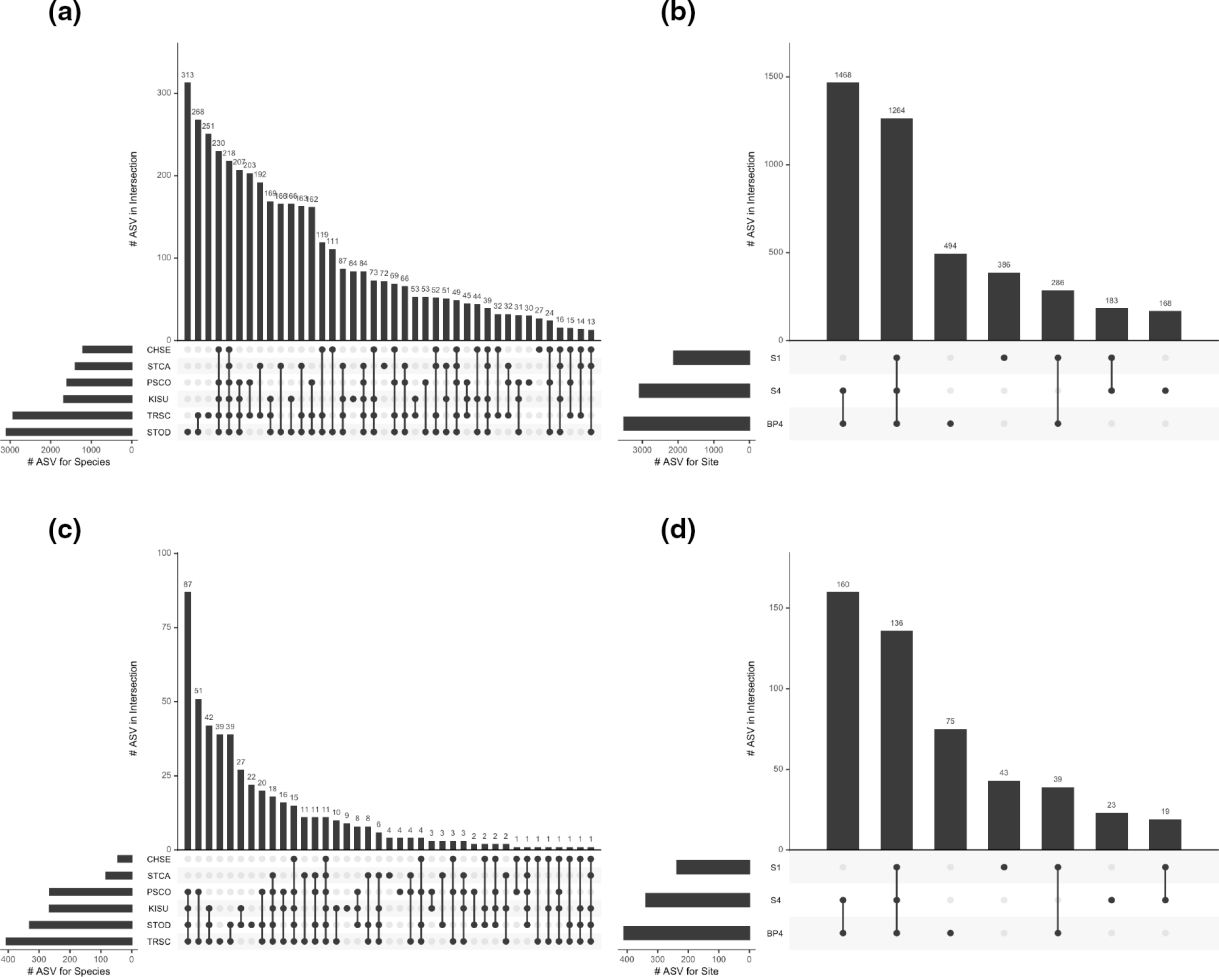
UpSet plots showing microbial diversity by turtle species and collection site. (**a**) 16S rRNA gene ASV distribution by turtle species; (**b**) 16S rRNA gene ASV distribution by collection site; (c) 18S rRNA gene ASV distribution by turtle species; (**d**) 18S rRNA gene ASV distribution by collection site. In all plots, columns with vertical lines connecting turtle species or sites correspond to the number of shared ASVs for those species or sites. Singleton columns correspond to counts of turtle-species- or site-specific ASVs. Zero-count categories, including 18S rRNA gene ASVs unique to *C. serpentina*, are not shown.

The highest numbers of ASVs unique to a turtle species were recovered from *S. odoratus* and *T. scripta* shells for both prokaryotic and eukaryotic communities (16S/18S rRNA gene ASVs: *S. odoratus*=313/22, *T. scripta*=251/39), while the lowest numbers of unique ASVs were recovered from *C. serpentina* shells (16S/18S rRNA gene ASVs: 27/0) (Fig. 3a, c). Relatively small proportions of total ASVs were shared across all species’ shells (16S/18S rRNA gene ASVs: 218/11) (Fig. 3a, c). Across sites, the highest numbers of ASVs were recovered as shared by turtles in sites BP4 and S4, and by turtles in all sites, for both prokaryotic and eukaryotic communities (Fig. 3b, d). Sites BP4 and S1 had substantially higher numbers of unique carapace-associated ASVs for both prokaryotic and eukaryotic communities than did site S4.

### Alpha diversity

#### Species comparisons

**Fig. 4.**
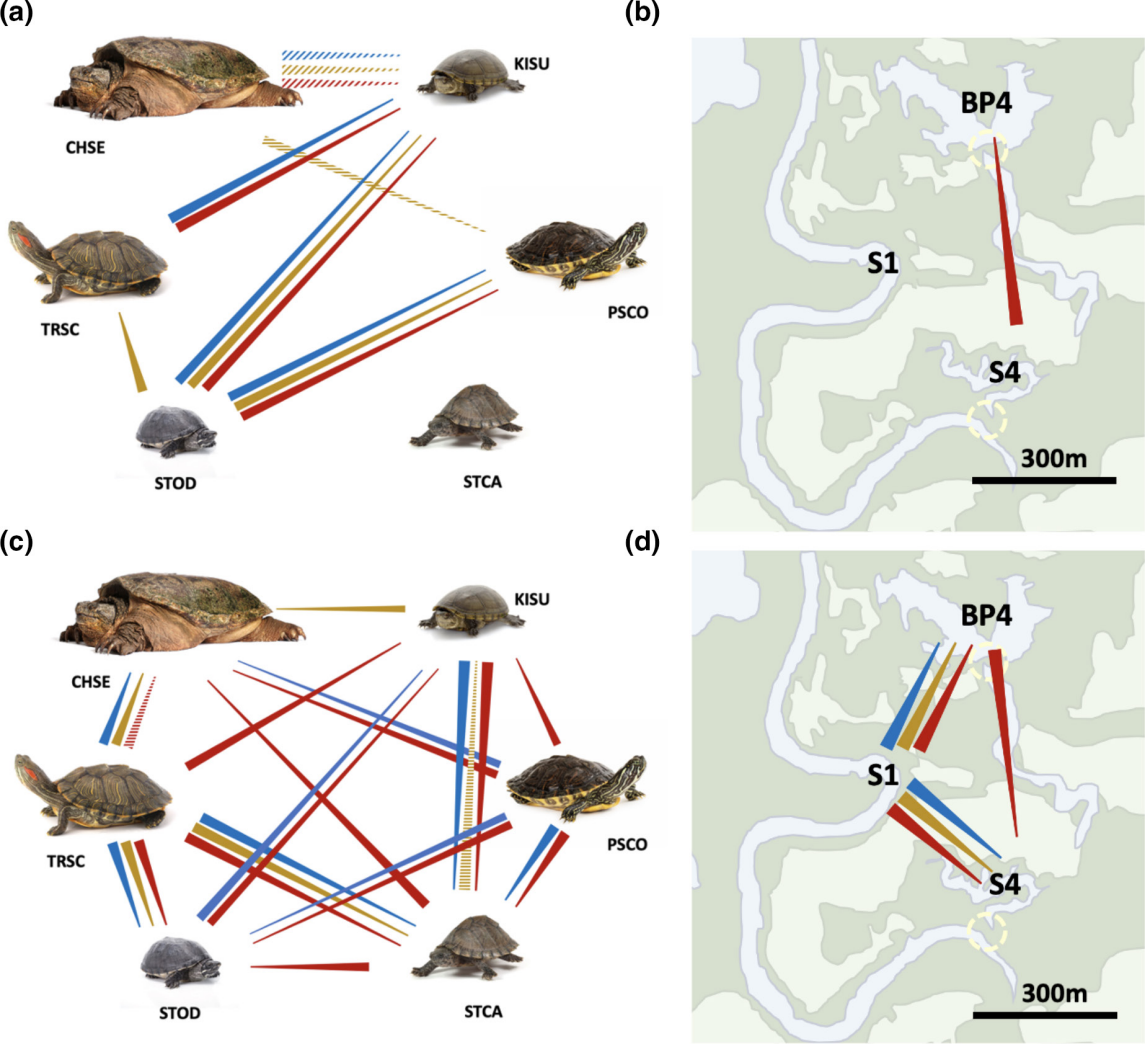
Pairwise comparisons of alpha diversity by turtle species and collection site, at the level of ASV. (**a**) and (b) show pairwise species and site comparisons, respectively, for prokaryotic communities; (**c**) and (d) show pairwise species and site comparisons, respectively, for eukaryotic communities. Significantly different values for alpha diversity are indicated by solid-coloured connectors (blue=observed feature counts; bronze=Shannon diversity index; red=Faith’s phylogenetic diversity); dashed connectors indicate near significance (0.10≥*p*-value-value > 0.05). The direction of comparisons indicates higher and lower levels of diversity, with wide ends of connectors indicating higher median diversity for a given metric. Results shown here are with outlier samples removed; full results and corresponding boxplots are available in supplementary material.

For prokaryotic carapace communities, median diversity was consistently highest from *C. serpentina*, *S. carinatus* and *S. odoratus* shells, and lowest for *K. subrubrum* and *P. concinna* shells, across all alpha diversity metrics. In contrast, for eukaryotic communities, median diversity was generally lowest for *C. serpentina*, *S. carinatus* and *S. odoratus*, and highest for *K. subrubrum*, *P. concinna* and *T. scripta* shells, although eukaryotic Shannon diversity for *S. carinatus* deviated in this regard. In pairwise comparisons of turtle species for prokaryotic alpha diversity measures, *K. subrubrum – S. odoratus*, *K. subrubrum – T. scripta*, and *P. concinna – S. odoratus* comparisons were significant across all alpha diversity measures, while *C. serpentina – K. subrubrum* was consistently nearly significant and *S. odoratus – T. scripta* was significant for Shannon index (Fig. 4a). Significant differences were more common in pairwise comparisons of eukaryotic diversity. For example, only *C. serpentina – S. odoratus* and *P. concinna – T. scripta* pairwise species comparisons did not feature any significant differences between eukaryotic communities for any alpha diversity metric (Fig. 4c).

**Fig. 5.**
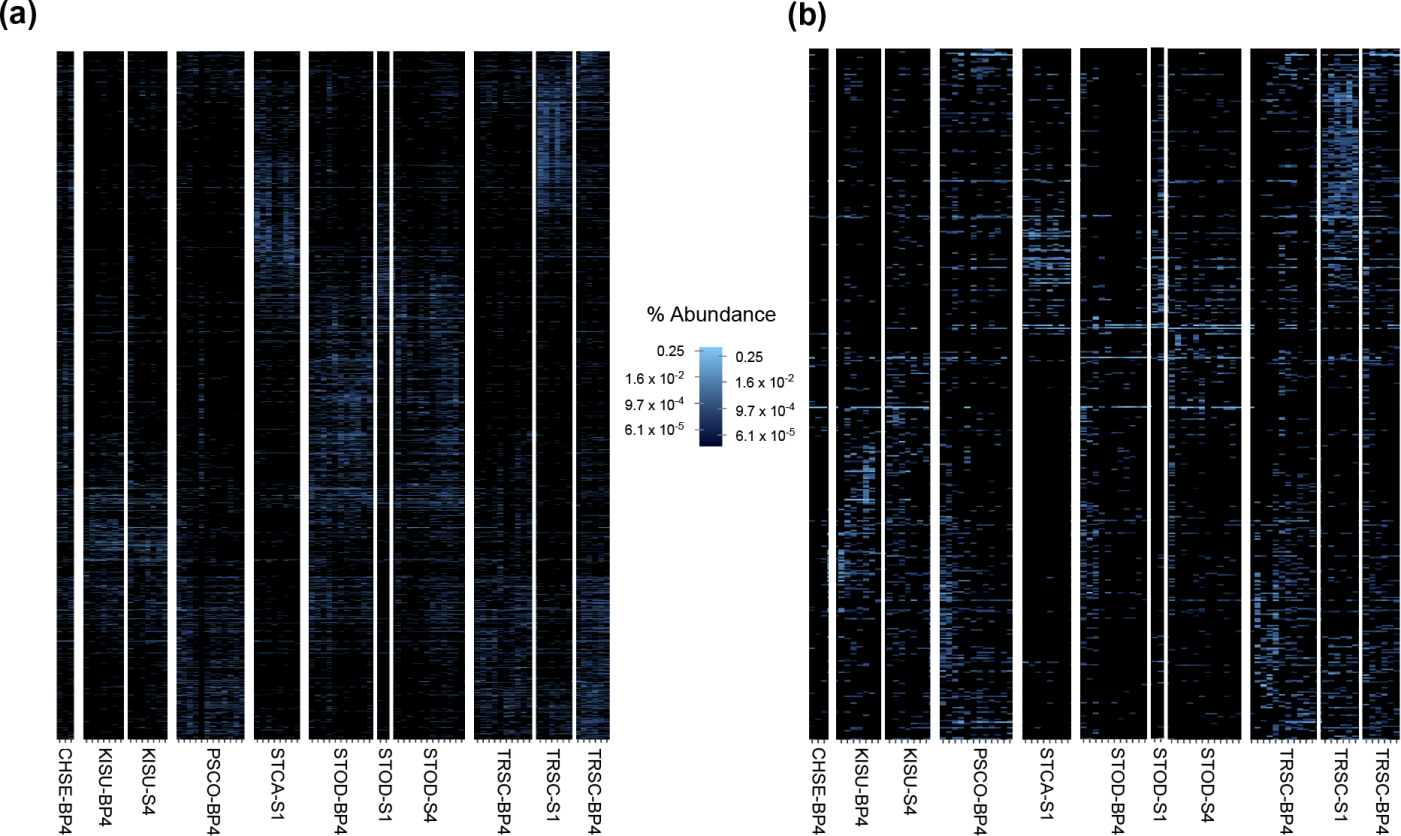
Heatmaps of microbial ASV distribution for turtle species and site combinations for (a) prokaryotic and (b) eukaryotic carapace communities. Each sample is indicated by four-letter abbreviation for species and by name of collection site. The left side of the percent abundance scale correlates to (a) and the right side of the scale correlates to (b). ASV taxonomy is not included on vertical axes due to space limitations; however, heatmaps for ASV through phylum taxonomic levels may be found in supplementary material.

#### Site comparisons

Of the three collection sites, site S1 tended to have the highest median diversity for both prokaryotic and eukaryotic carapace communities. In site pairwise comparisons for prokaryotic diversity, however, only BP4-S4 was significant, and only after removal of outlier samples (Fig. 4b). As with species comparisons, significant differences were more common in pairwise-site comparisons of eukaryotic diversity. Sites BP4 and S4 were significantly different from site S1 across all three alpha diversity metrics, and all three sites were significantly different for Shannon diversity (Fig. 4d).

### Beta diversity

#### General patterns

The distribution of ASVs across samples suggested differentiation of both prokaryotic and eukaryotic microbial communities by turtle species and, to a lesser extent, collection site (Fig. 5). This pattern was progressively less pronounced at the genus level through higher taxonomic levels.

**Fig. 6.**
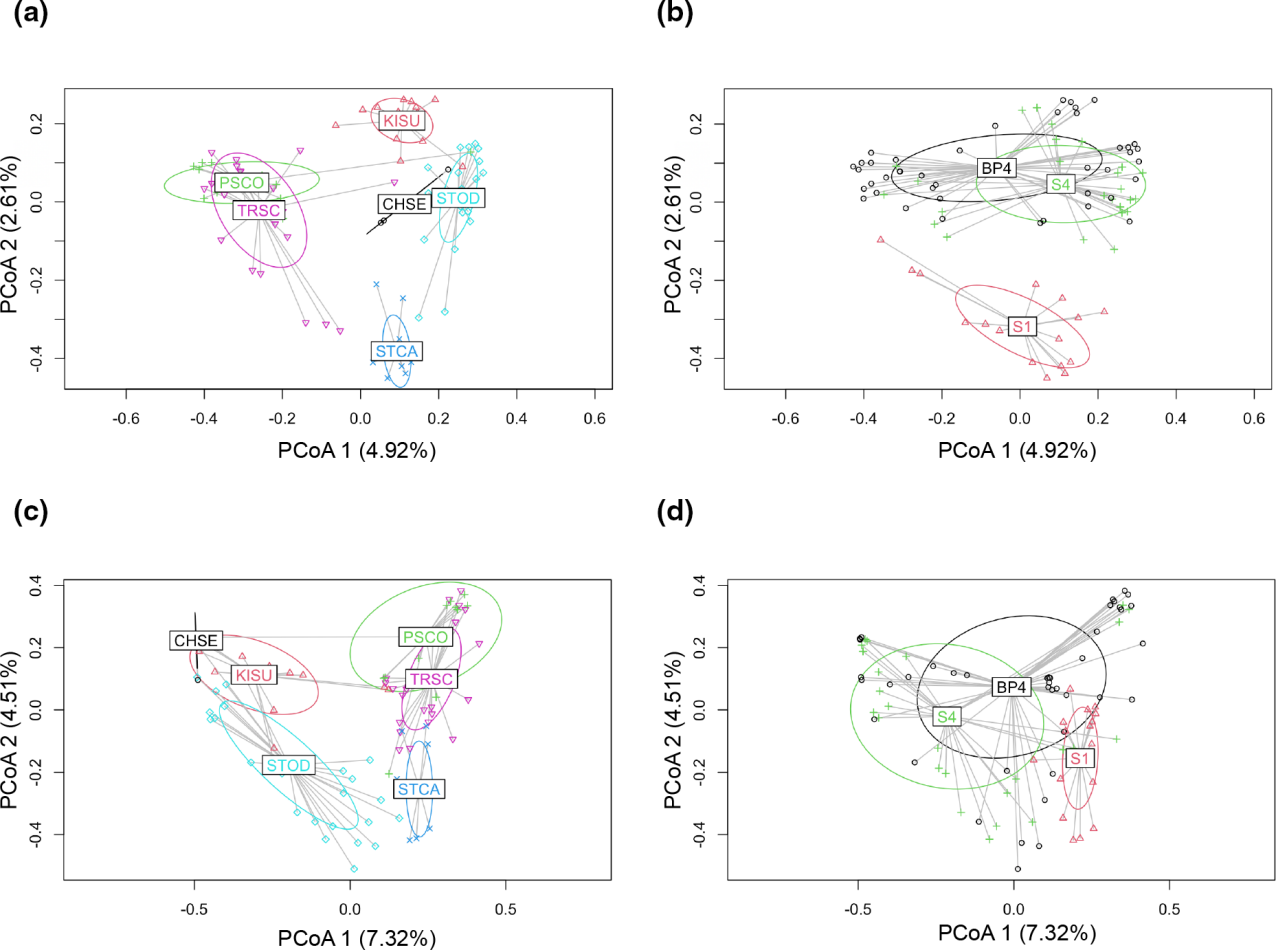
Ordinations of Jaccard indices for prokaryotic and eukaryotic rRNA carapace microbial communities. (**a**) and (b) show ordination of prokaryotic carapace communities by turtle species and collection site, respectively; (**c**) and (d) show ordination of eukaryotic carapace communities by turtle species and collection site, respectively. Data points, centroid locations and single standard deviation ellipses are colour-matched for each species or site. Centroid locations are indicated by species or site labels; grey lines are used to show connections of data points to centroid locations for each species or site. Corresponding ordination plots for Bray–Curtis dissimilarity and unweighted and weighted UniFrac metrics are available in the supplementary material.

Ordination patterns of prokaryotic and eukaryotic carapace microbial communities were largely consistent across all four measures of beta diversity applied. Similar to heatmaps, ordination plots supported influence of microbial communities by turtle species, and also by sites. Generally, the basking species *P. concinna* and *T. scripta* tended to cluster together, as did the benthic species *C. serpentina*, *K. subrubrum*, and *S. odoratus. S. carinatus*, which has both basking and benthic habits, tended to fall intermediate between these two groups (Fig. 6a, c). For sites, S1 tended to segregate from both S4 and BP4, while S4 and BP4 variably overlapped depending on beta diversity metric and whether prokaryotic or eukaryotic communities were considered (Fig. 6b, d).

#### Dispersion comparisons, species

**Fig. 7.**
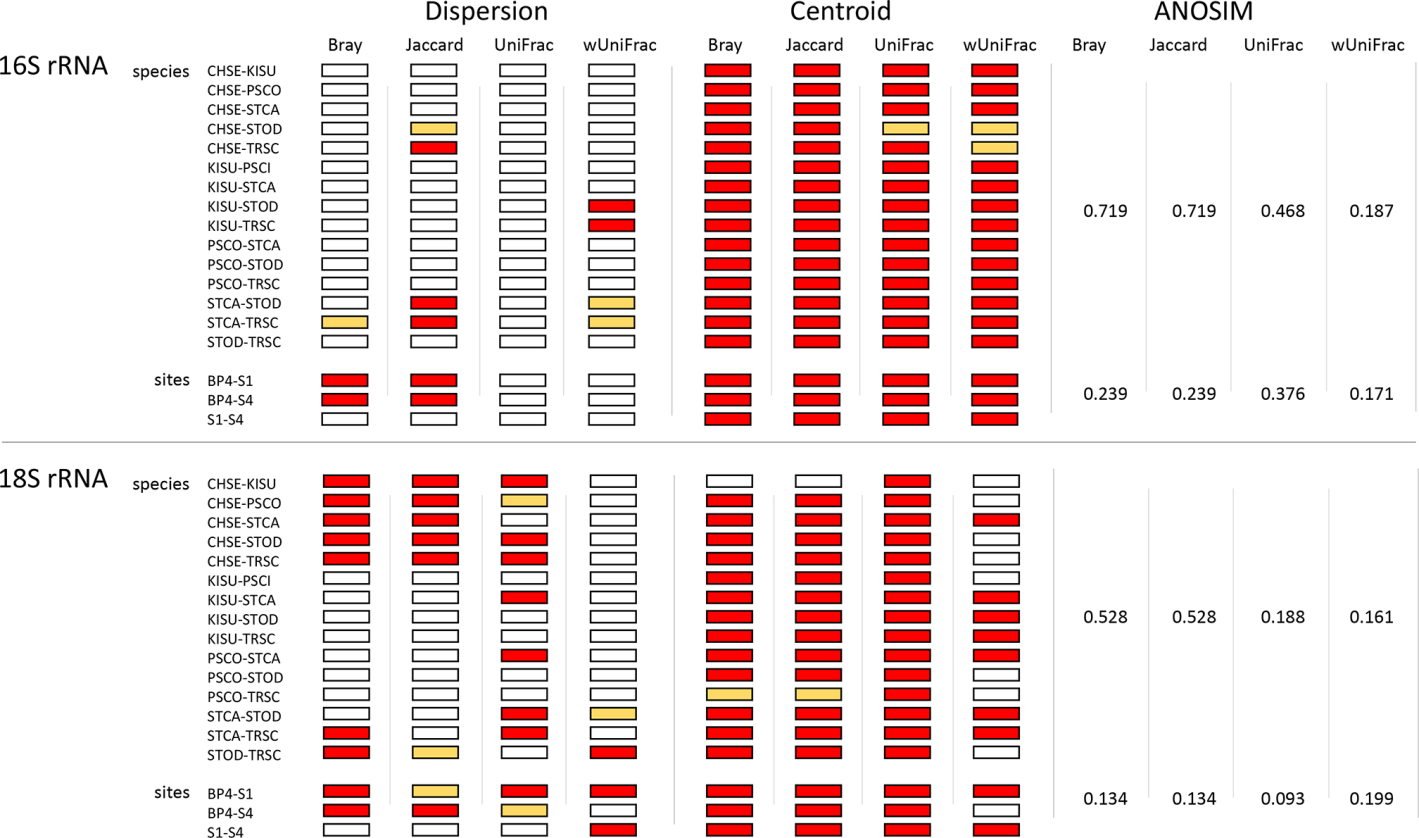
Results of significance testing for differences in species- and site-pairwise comparisons of beta diversity dispersion, centroid location, and ANOSIM scores. Red shaded boxes indicate a significant difference in beta dispersion or centroid location comparison for a given beta diversity metric (*p* ≦ 0.05); orange-shaded boxes represent nearly significant difference (0.05<*p* ≦ 0.10); clear boxes indicate corresponding *p*-values>0.10. ANOSIM scores shown indicate the discreteness of clustering for samples grouped by species or site, under each beta diversity metric. wUniFrac=weighted UniFrac.

For prokaryotic and eukaryotic communities, we recovered significant differences in beta dispersion between turtle species in all ANOVA analyses (prokaryotic: F_5,78_ = 2.5–6.0, *p*-value=0.039–9.6×10^−5^; eukaryotic: F_5,78_ = 5.9×10^−3^ – 14.4, *p*-value=0.0059–5.8×10^−10^). However, relatively few differences in beta dispersion were recovered as significant in subsequent pairwise comparisons of turtle species for prokaryotic communities (Fig. 7). For eukaryotic communities, the majority of pairwise comparisons of beta dispersion for turtle species were similarly not significant, although nearly half of pairwise comparisons of turtle species were recovered as significant for Bray–Curtis and unweighted UniFrac metrics and one-third were recovered as significant for Jaccard diversity (Fig. 7).

#### Dispersion comparisons, sites

For prokaryotic and eukaryotic communities, we recovered significant differences in beta dispersion between sites in all ANOVA analyses, with the exception of prokaryotic communities for UniFrac and weighted UniFrac metrics (prokaryotic: F_2,81_ = 1.5–14.7, *p*-value=0.97–3.5×10^−6^; eukaryotic: F_2,81_ = 5.9–9.4, *p*-value=0.002–2.1×10^−4^). In pairwise comparisons, BP4-S1 and BP4-S4 comparisons were commonly recovered as significant for differences in beta dispersion for both prokaryotic and eukaryotic carapace communities, although no significant differences were recovered between sites for prokaryotic UniFrac and weighted UniFrac metrics, and BP4-S1 and BP4-S4 comparisons were only nearly significant for eukaryotic Jaccard and unweighted UniFrac metrics (Fig. 7). The S1-S4 comparison was additionally recovered as significant only for the weighted UniFrac metric for eukaryotic communities (Fig. 7).

#### Centroid comparisons, species

In species pairwise comparisons, centroid location was recovered as significantly different for all pairwise comparisons of turtle species, across all beta diversity metrics in prokaryotic communities, with the exception of *C. serpentina – S. odoratus* for UniFrac and *C. serpentina – S. odoratus* and *C. serpentina – T. scripta* for weighted UniFrac metrics (Fig. 7). Similarly, a strong majority of pairwise comparisons across turtle species were recovered as significant for centroid location differences for eukaryotic communities, although only six of 15 pairwise comparisons were recovered as significant for the weighted UniFrac metric (Fig. 7). For eukaryotic Bray–Curtis and Jaccard metrics, *C. serpentina – K. subrubrum* and *P. concinna – T. scripta* comparisons did not return significant differences in centroid location. Overall, significant differences in centroid location were much more frequent on average than significant differences in beta dispersion in pairwise species comparisons for any beta diversity metric and for either prokaryotic or eukaryotic comparisons. For example, on average 95 % of species pairwise comparisons were recovered as significant for differences in centroid location for prokaryotic communities, while only 8.25 % of these comparisons were recovered as significant for differences in centroid dispersion (Fig. 7).

#### Centroid comparisons, sites

All pairwise comparisons of collection sites were recovered as significantly different for centroid location across all beta diversity metrics, with the exception of BP4-S4 for the weighted UniFrac metric (Fig. 7). As with species pairwise comparisons, significant differences in centroid location were much more frequent than were significant differences in beta diversity dispersion in pairwise site comparisons. For example, on average 100 % of site pairwise comparisons were recovered as significant for differences in centroid location for eukaryotic communities, while only 33.3 % of these comparisons were recovered as significant for differences in centroid dispersion (Fig. 7).

#### PERMANOVA analyses, impacts of factors

In PERMANOVA analyses for differences in centroid location, all factors tested (species, site, log_10_[read depth], year) were recovered as significant for prokaryotic comparisons (all *p*-values<0.010), with the exception of the factor log_10_(read depth) for the weighted UniFrac metric. Results were similar for eukaryotic communities, except the factor log_10_(read depth) was only recovered as significant for the weighted UniFrac metric, and the factor species was not recovered as significant for the weighted UniFrac metric. Corresponding R^2^ values in PERMANOVA analyses were always highest for the factor species, and second highest for the factor site, except for the weighted UniFrac metric for eukaryotic communities where this order was reversed. R^2^ values for the factor species were on average 3.25-fold higher than for the factor site for prokaryotic communities (range=2.45–3.77-fold), and 2.43-fold higher than for the factor site for eukaryotic communities (range=0.40–3.80-fold), indicating the factor turtle species consistently explained the most variation in beta diversity centroid location across diversity metrics.

#### ANOSIM comparisons

ANOSIM values were higher for species-wise ordinations than for site-wise ordinations, for all beta diversity metrics and for both prokaryotic and eukaryotic communities, except for the weighted UniFrac metric for eukaryotic communities (Fig. 7). ANOSIM values for species-wise ordinations ranged from approximately 0.8–3.9-fold those of site-wise values (Fig. 7), indicating consistently more discrete clustering between species than between sites.

## Discussion

The role of microbial communities is increasingly appreciated in higher level systems process, including influence on host organism, ecosystem and even biosphere functioning [48, 49]. At the organismal level, microbial communities may impact host organism phenotype and fitness. This can include influence on host physiology and metabolism, immune function, development, and complex behaviours like feeding and cognitive activities ranging to predator-prey interactions [50–53]. At the ecosystem level, functional characteristics including nutrient cycling are impacted by microbial communities for native [54–57] and anthropogenic systems [58], ground water and freshwater systems [59, 60], and marine systems [61, 62]. Microbial communities may also serve as reservoirs for pathogens, influenced by both abiotic [63, 64] and biotic host systems [65]. Because of their pervasive impacts, it is important to assess diverse microbial communities to understand the functioning of both native and human-influenced systems more completely. The diverse microbial communities associated with freshwater turtle shells (documented herein and [17–19]) are individually very small parts of larger environments. Nonetheless, movement of turtles within and between diverse aquatic habitats [66, 67], and the prevalence of turtles throughout temperate and tropical biomes, suggests turtles could play important roles as a combination of accumulators and vectors for diverse microbial taxa. For spatial context, previous studies have measured seasonal or multi-year overland movements of *C. serpentina* and *T. scripta*, two of our study species, to at least to 1–2 kilometers [66, 68]. Other of our sampled species may traverse shorter overland distances, for example *S. odoratus* and *P. concinna* have been documented in the range of 100–200 m from water sources [69, 70]. In turn, the role of microbial vector could be further amplified in increasingly urbanized and fragmented habitats, which can result in increased range size for some turtle species [71]. Historically, diverse turtle species have had outsized influence in ecosystem energy flow, mineral cycling and both abiotic and biotic habitat characteristics. This includes that turtles represent some of the highest biomass values per unit area for animals. For example, T. *scripta* populations have been measured at over 855 kilograms/hectare, a number more than four-fold greater than for herds of large African plains herbivores [20]. Based on a combination of shell-associated microbial diversity, potential for overland movement, and population size and density, it should not be unexpected that turtles could play important roles in microbial ecology.

In the current study, we sampled microbial communities from carapace shell surfaces of six species of freshwater turtles, collected from three adjacent and seasonally interconnected wetland habitats. Overall, we recovered diverse microbial communities based on 16S and 18S rRNA gene sequencing, supporting that turtle shells are significant microbial accumulators in freshwater aquatic habitats. The prokaryotic and eukaryotic communities recovered in our study are comparable in their diversity to two previous studies on freshwater carapace-associated microbial communities [17, 18], and therefore support that turtle shells of various species may accumulate levels of microbial diversity similar to and overlapping with continuously submerged environmental substrates (sticks, rocks, etc.). Our results also included specific microbial taxa consistent with the two previous reports on freshwater carapace microbiomes [17, 18], including the genera *Deinococcus* and *Synechococcus* from prokaryotic communities, and *Epistylis*, *Tokophrya*, and *Heliophrya* from eukaryotic communities. The abundances of these taxa varied by turtle species. For example, *Deinococcus* was represented in over 14 % of 16S rRNA sequence reads across all *K. subrubrum* samples, but in less than 1 % of 16S rRNA sequence reads across *S. carinatus* samples. Similarly, *Epistylis* and *Heliophrya* were represented in over 24 and 23 % of 18S rRNA sequence reads, respectively, across *S. carinatus* samples, but were recovered in less than 0.03 % of 18S rRNA sequence reads in *C. serpentina*. We also recovered additional genera previously reported as more abundant on turtle shells compared to submerged environmental substrates [18], on the shells of our sampled turtles. These genera included *Paracoccus* (prokaryote), *Opercularia* (eukaryote) and *Heliophrya* (eukaryote). *Paracoccus* is a diverse taxon described as metabolically flexible, including due to their use of varied electron donors and terminal electron acceptors in aerobic respiration [72] and capabilities of xenobiotic degradation in some strains [73]. *Opercularia* and *Heliophrya* are known aquatic epibionts commonly found in polluted or high nutrient load environments [74, 75], and at least *Opercularia* seems capable of parasitizing host tissues in some cases [76]. While these three genera were not within the top-most abundant genera in our study, each of these genera was still recovered from multiple individuals and turtle species.

The partitioning of microbial taxa in our data also indicates that different turtle species harbour uniquely structured microbial communities, which is supported to be reflective of species ecology and habitat use. We recovered largely contrasting patterns in prokaryotic versus eukaryotic alpha diversity, with *C. serpentina*, *S. carinatus* and *S. odoratus* highest in prokaryotic diversity, but lowest in eukaryotic diversity. During warmer months, these three species tend to remain relatively benthic, which may favour exposure to richer bacterial diversity associated with greater depths and benthic substrates, compared to shallower and pelagic habitats [77, 78]. Additionally, overall eukaryotic microbial diversity can be negatively impacted by oxygen limitations in benthic habitats. This has been demonstrated to occur within the range of several metres’ depth, including for both freshwater [79] and marine [80] systems, and between oxic and hypoxic zones within the same freshwater lake [81]. The effect of oxygen limitation may be reflected in eukaryotic communities associated with the three benthic turtle species in our sampling (*C. serpentina*, *K. subrubrum*, and *S. odoratus*). In each of these species, relatively high proportions of eukaryotic microbial communities consisted of the photosynthetic clade Cladophorales, particularly at sites BP4 and S4 (Fig. 2b, d). These sites tend to maintain relatively clearer (BP4, S4) and shallower (S4) water conditions compared to site S1. Increased light incidence could promote the growth of photosynthetic microbes, while limited oxygen may provide a further advantage to oxygenic micro-organisms; however, without additional physicochemical sampling, our data are limited in addressing this possibility. Sampling site played a lesser role in determining microbial diversity compared to turtle species. This was expected due to the proximity and seasonal connections between our three sites; however, each site still represented a distinct habitat type. Across our three sampling sites, site S1 carried the highest levels of alpha diversity for both prokaryotic and eukaryotic communities (although differences were only significant for eukaryotic communities). As a backwater slough, S1 has a combination of differences to our other sites that likely influence this trend. For example, S1 has a longer wet season compared to S4 (which is ephemeral), and more dynamically fluctuating conditions compared to BP4 (which is less impacted by precipitation events). Site S1 regularly experiences substantial changes in water levels and substantial backflow from the mainstem creek during flooding events, both of which may introduce at least temporary additional microbial diversity between these two habitats and from surrounding terrestrial habitat [82–84].

To understand patterns in beta diversity more clearly, we distinguished between the effects of beta dispersion and centroid location. Our data support that each of these variables was influenced by both turtle species and collection sites; however, centroid location varied much more consistently. This was true when data was partitioned by either turtle species or collection site. For example, the percentage of significant differences in species-pairwise comparisons of centroid locations averaged 95 and 80 % for 16S and 18S rRNA gene ordinations, respectively, versus species-pairwise comparisons of beta dispersion which averaged 8.3 and 33.3 %, respectively (Fig. 7). Similarly, the percentage of significant differences in site-pairwise comparisons of centroid locations averaged 100 and 91.7 % for 16S and 18S rRNA gene ordinations, respectively, compared to differences in beta dispersion which averaged 33.3 and 41.8 %, respectively (Fig. 7). These results support that differences in beta diversity were mainly driven by centroid location, rather than beta dispersion. Following, turtle species was clearly the most influential factor in both 16S and 18S rRNA gene beta diversity centroid locations (with the exception of 18S rRNA gene weighted UniFrac measurements), accounting for up to nearly four times the variation in centroid location as explained by sample site in PERMANOVA analyses. Anosim results further supported that turtle species more strongly influenced microbial community than did sample site, since ANOSIM values were consistently higher in ordinations of communities by turtle species than by site. Overall, while our data do support a role of habitat in microbial community assemblage, even across fine-scale geographic distances, our results strongly support turtle species as the dominant factor impacting microbial communities in our sampled wetlands. These results are generally consistent with previous work regarding interplay between turtle shell-associated and local environmental microbiomes, that evaluated roles for both turtle species and habitat. For example [18], demonstrated that *T. scripta* shell microbial communities consistently clustered distinct from local environmental samples across different habitat types and substantial geographic distances, even though microbial communities were broadly overlapping between turtle shells and environmental substrates. Additionally [19], demonstrated that habitat quality influences prokaryotic diversity for turtle shell microbial communities in *E. marmorata*.

As with patterns in alpha diversity, the general patterns in ordination of beta diversity for both prokaryotic and eukaryotic communities again appear to indicate not just differences between turtle species, but also the influence of turtle species’ ecologies. For example, the benthic species *C. serpentina*, *K. subrubrum* and *S. odoratus* tended to fall more closely together in ordinations, as did the basking species *P. concinna* and *T. scripta. S. carinatus*, which has both basking and benthic habits, tended to fall intermediate to the more fully basking and benthic species. Our results for *P. concinna* and *T. scripta* are congruent to those previously reported by [18], in which a single *P. concinna* sample resided within a cluster of *T. scripta* samples for both 16S and 18S rRNA gene beta diversity ordinations. Similarly, prokaryotic communities of *T. scripta* and the western pond turtle *Emys marmorata*, another basking species, have recently been shown to overlap [19]. For 16S rRNA gene results, the lack of significant differentiation between *C. serpentina* and *S. odoratus* communities (for UniFrac and weighted UniFrac metrics), likely further reflects use of similar benthic habitat for these species. It is possible that the lack of differentiation between *C. serpentina* and *K. subrubrum* eukaryotic communities (for Bray–Curtis and Jaccard metrics) is also due to similar ecologies, although *K. subrubrum* generally utilizes relatively shallow benthic habitats [85]. *P. concinna* and *T. scripta* clustered together in beta diversity across all comparisons of prokaryotic and eukaryotic communities, although these communities were still mostly recovered as significantly different. Similarity in *P. concinna* and *T. scripta* communities is also consistent with previous work [18], and likely reflects shared basking habits and similar habitat usage. Differentiation of microbial communities by turtle species could be further tested in captive environments and could have implications for conservation efforts since captive microbiomes may influence survival in wild-release captive individuals [22, 86]. Future studies may also focus on internal microbiomes (for example, cloacal or intestinal), as these may be additionally reflective of organismal fitness and survival in both wild and wild-release captive individuals [87].

Although our results support a role for species’ ecologies in carapace microbiome composition, our data set does not allow us to strictly determine whether differences in shell surfaces or chemistry may also play a role. The shells of freshwater turtles are complex structures, with a hierarchical and multi-layered microstructure that can include a waxy surface layer over keratin-rich scutes and collagen-rich dermis and sutures [88, 89]. Shell surfaces of turtle species can differ at the nano-scale level, for example in the depth of surface indents [90], which could influence microbial colonization. Beta-keratin peptide composition of shells may also vary, providing unique chemical properties to the shells of different turtle species [91]. To date, detailed structural and chemical descriptions for the shells of our sampled species are not available. Nonetheless, if shell surfaces and chemistry strongly influenced microbial colonization, one might expect similar communities on the shells of more closely related turtle species, since closely related species can have more similar shell chemistry [91]. Our turtle sampling featured both limited turtle species diversity and disparate levels of relatedness within our sampled species. For example, *S. carinatus* and *S. odoratus* are members of the same genus, *P. concinna* and *T. scripta* are members of the same family (Emydidae), and both *P. concinna* and *T. scripta* are separated in a different superfamily from *C. serpentina* (Testudinoidea vs. Cryptodira, respectively) [92, 93]. Because of this, we did not perform formal tests for phylogenetic signal in host-microbial community associations (for example [94]); however, we note here that our two congeneric species, *S. odorata* and *S. carinatus*, did not tend to cluster together in 16S or 18S rRNA gene ordinations. *S. odorata* samples instead tended to cluster most closely with *C. serpentina* in both cases. As noted above, *S. odorata* (colloquially referred to as ‘bottom walker’) shares a predominantly benthic habitat with *C. serpentina* and appears to accumulate a similar microbiota on carapace surfaces. *S. carinatus*, which has both benthic and basking behaviours, was consistently intermediate between the fully benthic and basking species in our data set. A formal analysis of phylogenetic signal in carapace microbial communities would be more robust in a more species-rich area such as the Mobile River basin.

Finally, our data also support that turtles are likely an underappreciated factor in the microbial interconnectivity of freshwater habitats. Since most of our sampled turtle species are known to move between our sampled sites [Hollender, unpublished data], they are likely contributing unique communities of shell-associated bacteria within and between habitats, which in turn could influence freshwater microbial community dynamics over time. Analogous influences have been documented in other vertebrates, including for migratory birds [95, 96] and for cattle [97]. In both cases, vertebrate hosts were shown to act as vectors for bacterial species and their genes, including known bacterial pathogens and antibiotic resistance genes. Turtles may similarly serve as important microbial vectors over potentially large distances if one considers a continuous ‘pond-hopping’ pattern where members of a turtle species are regularly shifting between adjacent habitats, resulting overall in long-distance microbial dispersion. This could impact microbial evolution, either leading to microbial homogenization through gene flow, or to opportunities for microbial adaptation and evolution, depending on the scale and rate of microbial transfer between adjacent habitats. For example, previous work has shown a lack of allopatric signal in a widespread freshwater bacterial species, with data instead supporting high levels of gene flow and recombination [98]. In this sense, turtle shells may be only one of many vectors by which some bacterial species and their genetic material could move across geographic scales, contributing to genetic homogenization through gene flow. Alternatively, turtles as vectors could contribute to the spread of ‘ecoSNPs’ (ecologically differentiated single nucleotide polymorphisms) between bacterial populations variously adapted to different habitat conditions in a relatively small geographic area [99], or could contribute to the maintenance of broad host-range plasmids that enable genetic heterogeneity and increase the robustness of bacterial populations over time [100]. In either case, turtle shell microbiota may provide a unique system for future metabarcoding- and metagenomics-based microbial community analysis, as well as hypothesis testing in microbial evolution, and assuredly warrant additional exploration at the level of the microbiome.

## Supplementary Data

Supplementary material 1
