# Supplementary material for: Turtle species and ecology drive carapace microbiome diversity in three seasonally interconnected wetland habitats
Source: Access Microbiol. 2024 Jan 12;6(1):000682.v3. doi: 10.1099/acmi.0.000682.v3 (PMC10866032; doi:10.1099/acmi.0.000682.v3)
Supplement: Supplementary material 1 [file acmi-6-682.v3-s001.pdf]

R Markdown for analyses of 16S and 18S rRNA gene carapace communities, Parks et al.

2023-12-11

Contents

Overview of supplementary materials 1

16S rRNA gene analyses 1

- Load libraries . . . 1
- Add simplifying code . . . 2
- Set the working directory and phyloseq objects . . . 2
- Estimating alpha diversity . . . 3
- Setting phyloseq objects to different taxonomic levels . . . 9
- Isolate carapace samples for each turtle species . . . 9
- Making stacked barplots for taxonomy . . . 24
- Extracting abundances for several taxa shared with Parks et al. (2020) . . . 24
- Statistical testing for alpha diversity significance . . . 29
- Alpha diversity boxplots for carapace samples . . . 60
- Check for effects of plastron length within each turtle species . . . 66
- Generate heat maps for different taxonomic levels: . . . 105
- Beta diversity comparisons . . . 118
- generate UpSetR plots . . . 147

18S rRNA gene analyses 149

- Load libraries for analysis . . . 149
- Add simplifying code . . . 149
- Set the working directory and phyloseq objects . . . 150
- Estimating alpha diversity . . . 151
- Setting phyloseq objects to different taxonomic levels . . . 157
- Isolate carapace samples for each turtle species . . . 157
- Making stacked barplots for taxonomy . . . 171
- Extracting abundances for several taxa shared with Parks et al. (2020) . . . 172
- Statistical testing for alpha diversity significance . . . 201

Alpha diversity boxplots for carapace samples 232
Check for effects of plastron length within each turtle species 238
Generate heat maps for different taxonomic levels: 277
Beta diversity comparisons 289
generate UpSetR plots 318

Overview of supplementary materials

This pdf includes the R markdowns/code for analyses of 16S and 18S rRNA gene analyses. The code is very similar between the 16S rRNA and 18S rRNA gene analyses, with some nuances relevant to each data set. Both code and text/visual output is included in this R markdown. 16S rRNA gene analyses are presented first, followed by 18S rRNA gene analyses.

16S rRNA gene analyses

Load libraries

Here all necessary libraries are loaded for statistical analyses, with the exception of UpSet Plot-related libraries. The latter may cause problems with the initial set of libraries, so they are loaded and UpSet plots are generated at the end of the R markdown. The library easystats may be updated as necessary along with other libraries.

```
library(vegan)
library(ggplot2)
library(usedist)
library(car)
library(performance)
library(emmeans)
library(plyr)
library(lme4)
library(splitstackshape)
library(kableExtra)
library(ggpubr)
library(gtable)
library(grid)
library(gridExtra)
#easystats::easystats_update()
library(easystats)
library(see)
library(phyloseq)
library(microViz)
library(pairwiseAdonis)
library(btools)
library(olsrr)
```

Add simplifying code

This code snippet simplifies subsequent code.

```
#simplification coding:
#wrapper function with settings used for tables throughout. This simplifies the code elsewhere
# x is the object o be turned into a kable
# caption is the caption
# row.names - determines whether to include row names (NA is default for kbl)
kable.wrap <- function(x,caption,row.names=NA){
  kable_classic(full_width = F,html_font = "Calibri",font_size = 18,kbl(x,align="c",caption=caption,row
}
```

Set the working directory and phyloseq objects

The code below sets working directory and assigns phyloseq objects. Working directory would need to be adjusted for other users/computers.

```
#setting up directory and clarifying metadata
setwd("/Users/mparks10/Desktop/atoka.2021_2022.combined/phyloseq")
meta <- read.csv("Atoka_2021_2022.16S.metadata.w_morphology.csv")

meta$Carapace_length <- as.numeric(as.character(meta$Carapace_length))
meta$Plastron_length <- as.numeric(as.character(meta$Plastron_length))
meta$Mass <- as.numeric(as.character(meta$Mass))
#sapply(meta, class)

rownames(meta) <- meta$sample.ID
meta.phylo <- sample_data(meta)

# function for normalizing to proportions
prop.trans <- function(x){x/sum(x)}

data <- read.csv("16S.asv.table.filter.cleaned.csv")
rownames(data) <- data[,1]
data <- data[,2:(ncol(data)-1)]
data <- as.matrix(data)

data <- apply(data,2,prop.trans) #convert to proportions
asv <- otu_table(data,taxa_are_rows = T)

#reading in numerical data (i.e., integer counts rather than proportions)

data_np <- read.csv("16S.asv.table.filter.cleaned.csv") #note suffice 'np' means 'not proportions', ind
rownames(data_np) <- data_np[,1]
data_np <- data_np[,2:(ncol(data_np)-1)]
data_np <- as.matrix(data_np)
asv_np <- otu_table(data_np,taxa_are_rows = T)


#reading in taxon information
#in this file labels for 'unknown' have been replaced with "unknown_ ..." where "..." = the lowest taxo
taxa <- read.csv("16S.taxa_filter.unknown.cleaned.csv")
rownames(taxa) <- taxa[,1]
taxa <- as.matrix(taxa[,-1])
colnames(taxa)[7] <- "ASV"
taxa <- tax_table(taxa)
```

```r
# load rooted tree file and trim
tree <- read_tree("16S.rooted.tree.nwk")
tree <- prune_taxa(rownames(asv),tree)

# convert to phyloseq, tax_fix can be used to adjust any non-conforming taxon names
phylo <- merge_phyloseq(taxa,asv,tree,meta.phylo)
phylo_np <- merge_phyloseq(taxa,asv_np,tree,meta.phylo)
phylo<-tax_fix(phylo)
phylo_np<-tax_fix(phylo_np)
```

Estimating alpha diversity

The code below calculates different measures of alpha diversity to be used in later analysis.

```r
richness <- estimate_richness(phylo_np,measures=c("Observed","Chao1","ACE","Shannon","Simpson","InvSimps
richness
```

```
##           Observed   Chao1  se.chao1       ACE   se.ACE   Shannon   Simpson
## Sample2         18  18.000 0.0000000       NaN      NaN 2.6539418 0.9170699
## Sample13       146 146.000 0.0000000 146.00000 3.668638 3.7295591 0.9587400
## Sample20       676 676.000 0.0000000 676.00000 5.988648 6.0250509 0.9956702
## Sample22       529 529.000 0.0000000 529.00000 5.866183 5.3434394 0.9879234
## Sample33       300 300.000 0.0000000 300.00000 4.608326 4.4703683 0.9723864
## Sample36       478 478.000 0.0000000 478.00000 4.581206 4.9283860 0.9823568
## Sample37       227 227.000 0.0000000 227.00000 4.716640 4.1703909 0.9714802
## Sample38       416 416.000 0.0000000 416.00000 6.144885 4.5569931 0.9621423
## Sample3         37  37.000 0.0000000  37.00000 2.609753 2.5484615 0.8330370
## Sample18       517 517.000 0.0000000 517.00000 5.635958 5.5252541 0.9888374
## Sample39       488 488.000 0.0000000 488.00000 6.985788 4.7432820 0.9408637
## Sample19       585 585.000 0.0000000 585.00000 7.001099 4.8000594 0.9753980
## Sample24       697 697.000 0.0000000 697.00000 6.282448 5.5953054 0.9865533
## Sample21       734 734.000 0.0000000 734.00000 7.072994 5.6430590 0.9891987
## Sample23       739 739.000 0.0000000 739.00000 6.293885 5.6485079 0.9897543
## Sample14       574 574.000 0.0000000 574.00000 6.568985 5.4738053 0.9898159
## Sample25       431 431.000 0.0000000 431.00000 6.090958 5.1525519 0.9860599
## Sample4         17  17.000 0.0000000  17.00000 2.029199 0.6931472 0.5000000
## Sample5         17  17.000 0.0000000  17.00000 2.029199 0.6931472 0.5000000
## Sample26       392 392.000 0.0000000 392.00000 6.490181 4.7478190 0.9770835
## Sample15       189 189.000 0.0000000 189.00000 3.933306 4.3593686 0.9786393
## Sample27       261 261.000 0.0000000 261.00000 4.838384 4.5964714 0.9824489
## Sample16       328 328.000 0.0000000 328.00000 5.298124 4.6807433 0.9767615
## Sample28       455 455.000 0.0000000 455.00000 8.496929 5.0811422 0.9854272
## Sample29       429 429.000 0.0000000 429.00000 5.742736 5.2745901 0.9904370
## Sample30       374 374.000 0.0000000 374.00000 5.252959 4.7510495 0.9772726
## Sample6         46  47.000 2.3269924  46.40211 3.348487 1.1523786 0.6459958
## Sample7         46  47.000 2.3269924  46.40211 3.348487 1.1523786 0.6459958
## Sample31       294 294.000 0.0000000 294.00000 4.511512 4.4413987 0.9673090
## Sample34       412 412.000 0.0000000 412.00000 6.570565 5.3223507 0.9892672
## Sample35       233 233.000 0.0000000 233.00000 4.552979 3.8795606 0.9459411
## Sample40       442 442.000 0.0000000 442.00000 5.525957 4.2950718 0.9358084
## Sample32       675 675.000 0.0000000 675.00000 6.988456 5.3555324 0.9802597
## Sample17       305 305.000 0.0000000 305.00000 5.200883 4.6101108 0.9779298
```

```
## Sample9         557   557.000 0.0000000   557.00000 5.491956 5.7883798 0.9939780
## Sample8          24    24.000 0.0000000    24.00000 1.354006 2.7577903 0.9178780
## Sample10        588   588.000 0.0000000   588.00000 5.581036 5.8081250 0.9945879
## Sample11        453   453.000 0.0000000   453.00000 4.860073 5.5132798 0.9906974
## Sample12        224   224.000 0.0000000   224.00000 3.234165 2.9153603 0.8583082
## Sample67         61    61.125 0.4435064    61.65619 3.731629 1.5941564 0.7736703
## Sample104        61    61.125 0.4435064    61.65619 3.731629 1.5941564 0.7736703
## Sample41        601   601.000 0.0000000   601.00000 6.831706 4.8130761 0.9771733
## Sample42        915   915.000 0.0000000   915.00000 7.879974 6.1885459 0.9951924
## Sample43        274   274.000 0.0000000   274.00000 4.851067 3.4717520 0.9024037
## Sample44        683   683.000 0.0000000   683.00000 8.358371 5.3962298 0.9877838
## Sample45        286   286.000 0.0000000   286.00000 4.598951 4.2599986 0.9700969
## Sample46        446   446.000 0.0000000   446.00000 6.423053 4.7780558 0.9815483
## Sample47        427   427.000 0.0000000   427.00000 6.406598 4.0646821 0.9187198
## Sample48        270   270.000 0.0000000   270.00000 4.587019 4.2008239 0.9634551
## Sample49        321   321.000 0.2496103   321.13833 6.369213 3.9475908 0.9549357
## Sample50        631   631.000 0.0000000   631.00000 6.120812 4.6501496 0.9571722
## Sample51        381   381.000 0.0000000   381.00000 5.490149 3.8112823 0.9381818
## Sample52        470   470.000 0.0000000   470.00000 6.315129 3.4763936 0.8855343
## Sample53        390   390.000 0.0000000   390.00000 5.570987 4.4800248 0.9766741
## Sample54        857   857.000 0.0000000   857.00000 5.021076 6.3448994 0.9970245
## Sample55        631   631.000 0.0000000   631.00000 6.048929 5.8395266 0.9946624
## Sample56        435   435.000 0.4994250   435.14286 4.227490 4.9603178 0.9801574
## Sample57        832   832.000 0.0000000   832.00000 5.201701 5.3790538 0.9444502
## Sample58        528   528.000 0.0000000   528.00000 6.217644 4.6316397 0.9655145
## Sample59        798   798.000 0.4996866   798.14000 5.758435 6.0610788 0.9942151
## Sample60        597   597.000 0.0000000   597.00000 6.889897 4.4338308 0.9511532
## Sample61        578   578.000 0.0000000   578.00000 4.286658 4.6861238 0.9452630
## Sample62        654   654.000 0.0000000   654.00000 6.055932 5.4444719 0.9886082
## Sample63        670   670.000 0.0000000   670.00000 6.479013 5.7879884 0.9936091
## Sample64        284   284.000 0.0000000   284.00000 4.597535 4.0619816 0.9489557
## Sample65        703   703.000 0.0000000   703.00000 6.814970 5.4965474 0.9882506
## Sample66        763   763.000 0.0000000   763.00000 7.657135 5.7042962 0.9867157
## Sample68        422   422.000 0.0000000   422.00000 5.591124 4.7838369 0.9745292
## Sample69        402   402.000 0.0000000   402.00000 5.796079 4.8803287 0.9834242
## Sample70        596   596.000 0.0000000   596.00000 5.421032 5.4995740 0.9863427
## Sample71        555   555.000 0.0000000   555.00000 5.649524 5.4475030 0.9912641
## Sample72        296   296.000 0.0000000   296.00000 4.784194 4.1626190 0.9240635
## Sample73        799   799.000 0.0000000   799.00000 6.308109 5.8110794 0.9923224
## Sample74        368   368.000 0.0000000   368.00000 5.555060 4.6738697 0.9737371
## Sample75       1019  1019.000 0.0000000  1019.00000 7.572871 6.2316523 0.9953962
## Sample76        930   930.000 0.0000000   930.00000 7.193560 6.0447903 0.9937161
## Sample77        788   788.000 0.0000000   788.00000 7.445231 5.9185322 0.9941129
## Sample78        326   326.000 0.0000000   326.00000 5.059159 4.6964933 0.9780244
## Sample79        426   426.000 0.0000000   426.00000 5.952275 4.5668778 0.9700116
## Sample80        334   334.000 0.0000000   334.00000 5.303089 4.6416482 0.9752843
## Sample81        497   497.000 0.0000000   497.00000 6.585149 4.5583646 0.9654703
## Sample82        880   880.000 0.0000000   880.00000 7.301230 5.9305037 0.9928610
## Sample83        716   716.000 0.0000000   716.00000 6.944312 4.7790259 0.9569248
## Sample84        759   759.000 0.0000000   759.00000 6.573593 5.5358454 0.9858368
## Sample85        540   540.000 0.0000000   540.00000 6.223611 4.7466140 0.9629594
## Sample86        690   690.000 0.0000000   690.00000 6.810052 5.8245574 0.9934699
## Sample87        548   548.000 0.0000000   548.00000 6.860444 5.4735197 0.9912000
## Sample88        528   528.000 0.0000000   528.00000 5.938447 5.3802870 0.9859783
```

```
## Sample89       623  623.000 0.0000000  623.00000 5.669609 5.7039456 0.9930089
## Sample90       240  240.000 0.0000000  240.00000 5.715112 4.9600563 0.9874806
## Sample91       331  331.000 0.0000000  331.00000 5.664326 4.0336212 0.9498691
## Sample92       393  393.000 0.0000000  393.00000 6.124659 4.3513990 0.9641941
## Sample93       268  268.000 0.0000000  268.00000 5.582489 3.8935298 0.9477763
## Sample94       284  284.000 0.0000000  284.00000 5.328636 3.0779299 0.8427348
## Sample95       368  368.000 0.0000000  368.00000 5.768873 4.1454977 0.9050673
## Sample96       280  280.000 0.0000000  280.00000 4.309458 4.5934907 0.9801725
## Sample97       794  794.000 0.0000000  794.00000 6.235613 5.6182702 0.9867562
## Sample98       669  669.000 0.0000000  669.00000 8.254545 5.7270805 0.9928517
## Sample99       526  526.000 0.0000000  526.00000 4.971401 4.7741774 0.9492837
## Sample100      589  589.000 0.0000000  589.00000 4.985207 5.6433767 0.9912493
## Sample101      655  655.000 0.0000000  655.00000 6.605110 5.9022828 0.9929700
## Sample102      444  444.000 0.0000000  444.00000 5.894722 4.7727470 0.9767108
## Sample103      325  325.000 0.0000000  325.00000 6.047504 3.5305378 0.8956419
##           InvSimpson
## Sample2    12.058350
## Sample13   24.236577
## Sample20  230.954918
## Sample22   82.804718
## Sample33   36.214070
## Sample36   56.678980
## Sample37   35.063390
## Sample38   26.414695
## Sample3     5.989351
## Sample18   89.584726
## Sample39   16.910073
## Sample19   40.647034
## Sample24   74.367562
## Sample21   92.581665
## Sample23   97.601674
## Sample14   98.192035
## Sample25   71.735430
## Sample4     2.000000
## Sample5     2.000000
## Sample26   43.636776
## Sample15   46.814935
## Sample27   56.976458
## Sample16   43.032071
## Sample28   68.620849
## Sample29  104.570038
## Sample30   43.999717
## Sample6     2.824825
## Sample7     2.824825
## Sample31   30.589485
## Sample34   93.172320
## Sample35   18.498349
## Sample40   15.578363
## Sample32   50.657670
## Sample17   45.309946
## Sample9   166.057886
## Sample8    12.176998
## Sample10  184.770690
## Sample11  107.496251
```

```
## Sample12    7.057572
## Sample67    4.418333
## Sample104   4.418333
## Sample41   43.808269
## Sample42  208.002744
## Sample43   10.246291
## Sample44   81.858719
## Sample45   33.441381
## Sample46   54.195597
## Sample47   12.303120
## Sample48   27.363619
## Sample49   22.190494
## Sample50   23.349335
## Sample51   16.176454
## Sample52    8.736243
## Sample53   42.870851
## Sample54  336.078611
## Sample55  187.350286
## Sample56   50.396511
## Sample57   18.001872
## Sample58   28.997697
## Sample59  172.864803
## Sample60   20.472158
## Sample61   18.269195
## Sample62   87.782358
## Sample63  156.473164
## Sample64   19.590835
## Sample65   85.111079
## Sample66   75.276906
## Sample68   39.260611
## Sample69   60.328807
## Sample70   73.221035
## Sample71  114.470819
## Sample72   13.168893
## Sample73  130.249452
## Sample74   38.076457
## Sample75  217.213625
## Sample76  159.137052
## Sample77  169.862168
## Sample78   45.504999
## Sample79   33.346242
## Sample80   40.460080
## Sample81   28.960596
## Sample82  140.075028
## Sample83   23.215216
## Sample84   70.605490
## Sample85   26.997425
## Sample86  153.136228
## Sample87  113.636486
## Sample88   71.318109
## Sample89  143.039447
## Sample90   79.875806
## Sample91   19.947793
## Sample92   27.928329
```

```
## Sample93   19.148390
## Sample94    6.358686
## Sample95   10.533782
## Sample96   50.434945
## Sample97   75.506835
## Sample98  139.893796
## Sample99   19.717538
## Sample100 114.276186
## Sample101 142.247518
## Sample102  42.938444
## Sample103   9.582386
```

```
estimate_pd(phylo)
```

```
## Calculating Faiths PD-index...
```

```
##                  PD   SR
## Sample2    4.840115   18
## Sample13  17.195468  146
## Sample20  48.045513  676
## Sample22  39.305437  529
## Sample33  29.239557  300
## Sample36  41.441642  478
## Sample37  25.592896  227
## Sample38  37.976430  416
## Sample3    6.633836   37
## Sample18  39.786667  517
## Sample39  39.915676  488
## Sample19  47.399705  585
## Sample24  48.433193  697
## Sample21  48.984464  734
## Sample23  51.325531  739
## Sample14  40.974125  574
## Sample25  33.612713  431
## Sample4    3.574547   17
## Sample5    3.574547   17
## Sample26  30.479017  392
## Sample15  17.147339  189
## Sample27  25.274839  261
## Sample16  29.474613  328
## Sample28  36.797406  455
## Sample29  34.754421  429
## Sample30  31.016068  374
## Sample6    7.000246   46
## Sample7    7.000246   46
## Sample31  24.457913  294
## Sample34  37.579213  412
## Sample35  20.965606  233
## Sample40  34.212765  442
## Sample32  47.084236  675
## Sample17  26.897772  305
## Sample9   37.554828  557
## Sample8    4.601379   24
```

```
## Sample10  41.529742  588
## Sample11  33.320318  453
## Sample12  21.282598  224
## Sample67   9.288737   61
## Sample104  9.288737   61
## Sample41  48.424937  601
## Sample42  56.990469  915
## Sample43  31.456351  274
## Sample44  49.032939  683
## Sample45  26.241184  286
## Sample46  38.566112  446
## Sample47  39.563490  427
## Sample48  24.201338  270
## Sample49  29.437723  321
## Sample50  51.392932  631
## Sample51  35.443575  381
## Sample52  43.834184  470
## Sample53  34.468508  390
## Sample54  51.315462  857
## Sample55  40.342517  631
## Sample56  31.785639  435
## Sample57  51.961973  832
## Sample58  43.217946  528
## Sample59  47.983102  798
## Sample60  43.836281  597
## Sample61  42.340487  578
## Sample62  48.774181  654
## Sample63  46.349682  670
## Sample64  26.599547  284
## Sample65  51.246919  703
## Sample66  54.437130  763
## Sample68  31.251605  422
## Sample69  32.880012  402
## Sample70  42.504986  596
## Sample71  44.714802  555
## Sample72  25.133393  296
## Sample73  51.254060  799
## Sample74  36.713607  368
## Sample75  64.465244 1019
## Sample76  62.952849  930
## Sample77  55.604261  788
## Sample78  31.396972  326
## Sample79  40.228473  426
## Sample80  29.876123  334
## Sample81  39.143617  497
## Sample82  58.883650  880
## Sample83  52.313153  716
## Sample84  53.904947  759
## Sample85  43.652333  540
## Sample86  47.936171  690
## Sample87  45.013249  548
## Sample88  40.501769  528
## Sample89  48.336361  623
## Sample90  27.132246  240
```

```
## Sample91  33.046970  331
## Sample92  40.255592  393
## Sample93  32.869011  268
## Sample94  30.722509  284
## Sample95  35.495269  368
## Sample96  23.839270  280
## Sample97  53.878659  794
## Sample98  49.996005  669
## Sample99  43.671605  526
## Sample100 42.358659  589
## Sample101 44.126810  655
## Sample102 41.594026  444
## Sample103 33.789740  325
```

Setting phyloseq objects to different taxonomic levels

This code sets phyloseq objects consisting of different taxonomic levels, for both the proportional and numeric versions of counts data.

```
phylo.gen <- tax_glom(phylo, "Genus")
phylo_np.gen <- tax_glom(phylo_np, "Genus")

phylo.fam <- tax_glom(phylo, "Family")
phylo_np.fam <- tax_glom(phylo_np, "Family")

phylo.ord <- tax_glom(phylo, "Order")
phylo_np.ord <- tax_glom(phylo_np, "Order")

phylo.class <- tax_glom(phylo, "Class")
phylo_np.class <- tax_glom(phylo_np, "Class")

phylo.phy <- tax_glom(phylo, "Phylum")
phylo_np.phy <- tax_glom(phylo_np, "Phylum")


phylo.list <- list(phylo,phylo.gen,phylo.fam,phylo.ord,phylo.class,phylo.phy)
phylo_np.list <- list(phylo_np,phylo_np.gen,phylo_np.fam,phylo_np.ord,phylo_np.class,phylo_np.phy)

label.list <- list("ASV","Genus","Family","Order","Class","Phylum")
```

Isolate carapace samples for each turtle species

The code below isolates each species' carapace samples and double-checks that the commands worked. These data subsets are used later to check for any effects of plastron length within each species

```
carapace <- meta[meta$Substrate == "carapace",]
summary(carapace)
```

```
##   sample.ID         LibraryName          Read_depth     ProjectName
##  Length:84          Length:84          Min.   : 14153   Length:84
##  Class :character   Class :character   1st Qu.: 70359   Class :character
```

```
##  Mode  :character   Mode  :character   Median : 81136   Mode  :character
##                                        Mean   : 79907
##                                        3rd Qu.: 92568
##                                        Max.   :121835
##
##     Region           Sample_date            Year          Site
##  Length:84          Min.   :20210525   Min.   :2021   Length:84
##  Class :character   1st Qu.:20210526   1st Qu.:2021   Class :character
##  Mode  :character   Median :20220601   Median :2022   Mode  :character
##                     Mean   :20217963   Mean   :2022
##                     3rd Qu.:20220602   3rd Qu.:2022
##                     Max.   :20220602   Max.   :2022
##
##  Sample_type         Substrate           Species          Species_substrate
##  Length:84          Length:84          Length:84          Length:84
##  Class :character   Class :character   Class :character   Class :character
##  Mode  :character   Mode  :character   Mode  :character   Mode  :character
##
##
##
##
##  Species_site           Sorter          Sex            Carapace_length
##  Length:84          Min.   : 1.00   Length:84          Min.   : 49.4
##  Class :character   1st Qu.:21.00   Class :character   1st Qu.: 87.0
##  Mode  :character   Median :41.00   Mode  :character   Median : 98.0
##                     Mean   :41.22                      Mean   :124.8
##                     3rd Qu.:61.00                      3rd Qu.:165.7
##                     Max.   :82.00                      Max.   :308.0
##                     NA's   :3
##  Plastron_length      Mass        Sample_number    Sex_notes
##  Min.   : 35.8   Min.   :  25.0   Min.   : 1.00   Length:84
##  1st Qu.: 65.0   1st Qu.: 110.0   1st Qu.:12.75   Class :character
##  Median : 83.3   Median : 170.0   Median :31.50   Mode  :character
##  Mean   :104.0   Mean   : 492.7   Mean   :33.75
##  3rd Qu.:152.0   3rd Qu.: 682.5   3rd Qu.:53.25
##  Max.   :239.0   Max.   :6600.0   Max.   :75.00
##
##   PCR1_date         extraction_date       Observed          Chao1
##  Length:84          Min.   :    2021   Min.   : 146.0   Min.   : 146.0
##  Class :character   1st Qu.:    2021   1st Qu.: 368.0   1st Qu.: 368.0
##  Mode  :character   Median :20220620   Median : 492.5   Median : 492.5
##                     Mean   :14925274   Mean   : 518.0   Mean   : 518.0
##                     3rd Qu.:20220623   3rd Qu.: 669.2   3rd Qu.: 669.2
##                     Max.   :20220624   Max.   :1019.0   Max.   :1019.0
##
##     se.chao1            ACE            se.ACE         Shannon
##  Min.   :0.00000   Min.   : 146.0   Min.   :3.669   Min.   :3.078
##  1st Qu.:0.00000   1st Qu.: 368.0   1st Qu.:5.302   1st Qu.:4.538
##  Median :0.00000   Median : 492.5   Median :6.018   Median :4.781
##  Mean   :0.01487   Mean   : 518.0   Mean   :5.980   Mean   :4.942
##  3rd Qu.:0.00000   3rd Qu.: 669.2   3rd Qu.:6.576   3rd Qu.:5.528
##  Max.   :0.49969   Max.   :1019.0   Max.   :8.497   Max.   :6.345
##
##     Simpson         InvSimpson          Fisher       log10_read_depth
```

```
##  Min.   :0.8427   Min.   :  6.359   Min.   : 19.49   Min.   :4.151
##  1st Qu.:0.9613   1st Qu.: 25.870   1st Qu.: 51.16   1st Qu.:4.847
##  Median :0.9783   Median : 46.160   Median : 76.88   Median :4.909
##  Mean   :0.9702   Mean   : 69.895   Mean   : 79.62   Mean   :4.883
##  3rd Qu.:0.9889   3rd Qu.: 90.334   3rd Qu.:105.80   3rd Qu.:4.966
##  Max.   :0.9970   Max.   :336.079   Max.   :163.13   Max.   :5.086
##
##        PD
##  Min.   :17.15
##  1st Qu.:32.60
##  Median :40.42
##  Mean   :40.35
##  3rd Qu.:48.12
##  Max.   :64.47
##
```

```
CHSE<-carapace[carapace$Species == "CHSE",]
KISU<-carapace[carapace$Species == "KISU",]
PSCO<-carapace[carapace$Species == "PSCO",]
STCA<-carapace[carapace$Species == "STCA",]
STOD<-carapace[carapace$Species == "STOD",]
TRSC<-carapace[carapace$Species == "TRSC",]
CHSE
```

```
##           sample.ID
## Sample99   Sample99
## Sample100 Sample100
## Sample101 Sample101
##
## Sample99  ZI.CHSE_M_L2-R9_BP4_02-06-2022_71.CGGAGA_CTTT.16S.R1.adapter_trimmed.fq.gz,ZI.CHSE_M_L2-R9_
## Sample100     ZJ.CHSE_F_L9_BP4_02-06-2022_72.CGGAGA_TTTCA.16S.R1.adapter_trimmed.fq.gz,ZJ.CHSE_F_L9_
## Sample101   ZK.CHSE_M_L9_BP4_02-06-2022_73.CGGAGA_AAAGAA.16S.R1.adapter_trimmed.fq.gz,ZK.CHSE_M_L9_B
##           Read_depth  ProjectName  Region Sample_date Year Site Sample_type
## Sample99       88162 Atoka_turtle 16SV3V4    20220602 2022  BP4      turtle
## Sample100      81949 Atoka_turtle 16SV3V4    20220602 2022  BP4      turtle
## Sample101      71580 Atoka_turtle 16SV3V4    20220602 2022  BP4      turtle
##           Substrate Species Species_substrate Species_site Sorter Sex
## Sample99   carapace    CHSE     CHSE_carapace     CHSE_BP4     78   M
## Sample100  carapace    CHSE     CHSE_carapace     CHSE_BP4     79   F
## Sample101  carapace    CHSE     CHSE_carapace     CHSE_BP4     80   M
##           Carapace_length Plastron_length Mass Sample_number Sex_notes
## Sample99              308             221 6600            71      <NA>
## Sample100             231             179 2525            72      <NA>
## Sample101             209             152 2050            73      <NA>
##           PCR1_date extraction_date Observed Chao1 se.chao1 ACE   se.ACE
## Sample99       2022        20220624      526   526        0 526 4.971401
## Sample100      2022        20220624      589   589        0 589 4.985207
## Sample101      2022        20220624      655   655        0 655 6.605110
##            Shannon   Simpson InvSimpson    Fisher log10_read_depth       PD
## Sample99  4.774177 0.9492837   19.71754  79.71329         4.945281 43.67160
## Sample100 5.643377 0.9912493  114.27619  92.35752         4.913544 42.35866
## Sample101 5.902283 0.9929700  142.24752 107.29049         4.854792 44.12681
```

```
KISU
```

```
##          sample.ID
## Sample13  Sample13
## Sample14  Sample14
## Sample15  Sample15
## Sample16  Sample16
## Sample17  Sample17
## Sample45  Sample45
## Sample46  Sample46
## Sample49  Sample49
## Sample53  Sample53
## Sample74  Sample74
## Sample79  Sample79
## Sample80  Sample80
## Sample81  Sample81
## Sample96  Sample96
##
## Sample13                                          KISU-20210525-01-C-BP4.TGG_GA.16S.R1.adapter_tri
## Sample14                                        KISU-20210525-01-C-S4.GTAA_GTC.16S.R1.adapter_tri
## Sample15                                      KISU-20210525-02-C-S4.GAAAG_TGT.16S.R1.adapter_trimm
## Sample16                                  KISU-20210525-03-C-S4.GAAAG_TTTCA.16S.R1.adapter_trimmed
## Sample17                                      KISU-20210526-01-C-BP4.CCAT_GTC.16S.R1.adapter_trimm
## Sample45         EA.KISU_F_L2-R3-9-10_BP4_01-06-2022_5.AA_AAAGAA.16S.R1.adapter_trimmed.fq.gz,EA.KIS
## Sample46                   FA.KISU_M_L2-R-9-11_BP4_01-06-2022_6.AA_AT.16S.R1.adapter_trimmed.fq.gz,F
## Sample49     IA.KISU_M_L3-10-11-R10-11_BP4_01-06-2022_9.TGG_GA.16S.R1.adapter_trimmed.fq.gz,IA.KISU_
## Sample53           MA.KISU_F_L7-8-11_BP4_01-06-2022_13.TGG_AAAGAA.16S.R1.adapter_trimmed.fq.gz,MA.KI
## Sample74     YJ.KISU_M_L3-10-11-R2_S4_02-06-2022_45.TCCTGA_TGT.16S.R1.adapter_trimmed.fq.gz,YJ.KISU_
## Sample79     YO.KISU_M_L3-10-11-R3_S4_02-06-2022_50.TCCTGA_GTC.16S.R1.adapter_trimmed.fq.gz,YO.KISU_
## Sample80       YP.KISU_F_L3-8-9-R3_S4_02-06-2022_51.TCCTGA_AGAA.16S.R1.adapter_trimmed.fq.gz,YP.KISU
## Sample81               YQ.KISU_M_L3-10-11-R8_S4_02-06-2022_52.GC_GA.16S.R1.adapter_trimmed.fq.gz,YQ.
## Sample96 ZF.KISU_M_L3-10-R1-10_BP4_02-06-2022_68.AAAGAA_AGAA.16S.R1.adapter_trimmed.fq.gz,ZF.KISU_M_
##          Read_depth  ProjectName  Region Sample_date Year Site Sample_type
## Sample13      88081 Atoka_turtle 16SV3V4    20210525 2021  BP4      turtle
## Sample14      68361 Atoka_turtle 16SV3V4    20210525 2021   S4      turtle
## Sample15      26901 Atoka_turtle 16SV3V4    20210525 2021   S4      turtle
## Sample16      44427 Atoka_turtle 16SV3V4    20210525 2021   S4      turtle
## Sample17      65181 Atoka_turtle 16SV3V4    20210526 2021  BP4      turtle
## Sample45     106038 Atoka_turtle 16SV3V4    20220601 2022  BP4      turtle
## Sample46      87751 Atoka_turtle 16SV3V4    20220601 2022  BP4      turtle
## Sample49      93550 Atoka_turtle 16SV3V4    20220601 2022  BP4      turtle
## Sample53      96815 Atoka_turtle 16SV3V4    20220601 2022  BP4      turtle
## Sample74      89226 Atoka_turtle 16SV3V4    20220602 2022   S4      turtle
## Sample79      80323 Atoka_turtle 16SV3V4    20220602 2022   S4      turtle
## Sample80      72485 Atoka_turtle 16SV3V4    20220602 2022   S4      turtle
## Sample81     108222 Atoka_turtle 16SV3V4    20220602 2022   S4      turtle
## Sample96      58558 Atoka_turtle 16SV3V4    20220602 2022  BP4      turtle
##          Substrate Species Species_substrate Species_site Sorter Sex
## Sample13  carapace    KISU     KISU_carapace     KISU_BP4     NA   M
## Sample14  carapace    KISU     KISU_carapace      KISU_S4      7   F
## Sample15  carapace    KISU     KISU_carapace      KISU_S4     11   F
## Sample16  carapace    KISU     KISU_carapace      KISU_S4     13   F
## Sample17  carapace    KISU     KISU_carapace     KISU_BP4     19   F
## Sample45  carapace    KISU     KISU_carapace     KISU_BP4     24   F
```

```
## Sample46  carapace    KISU     KISU_carapace     KISU_BP4     25   M
## Sample49  carapace    KISU     KISU_carapace     KISU_BP4     31   M
## Sample53  carapace    KISU     KISU_carapace     KISU_BP4     32   F
## Sample74  carapace    KISU     KISU_carapace      KISU_S4     52   M
## Sample79  carapace    KISU     KISU_carapace      KISU_S4     57   M
## Sample80  carapace    KISU     KISU_carapace      KISU_S4     59   F
## Sample81  carapace    KISU     KISU_carapace      KISU_S4     58   M
## Sample96  carapace    KISU     KISU_carapace     KISU_BP4     76   M
##          Carapace_length Plastron_length Mass Sample_number Sex_notes
## Sample13            68.5            59.7   60             1      <NA>
## Sample14            90.0            84.9  175            15      <NA>
## Sample15           102.5            97.3  245            18      <NA>
## Sample16            87.7            86.8  145            20      <NA>
## Sample17            86.8            80.6  115            35      <NA>
## Sample45            69.0            60.5   53             5      <NA>
## Sample46            75.0            66.0   76             6      <NA>
## Sample49            77.0            66.0   80             9      <NA>
## Sample53            90.0            72.0  120            13      <NA>
## Sample74            93.0            72.0  130            45      <NA>
## Sample79            73.0            65.0   69            50      <NA>
## Sample80            95.0            91.0  190            51      <NA>
## Sample81            94.0            76.0  130            52      <NA>
## Sample96            65.0            56.0   49            68      <NA>
##           PCR1_date extraction_date Observed Chao1  se.chao1      ACE   se.ACE
## Sample13 15_17_June            2021      146   146 0.0000000 146.0000 3.668638
## Sample14 17_22_June            2021      574   574 0.0000000 574.0000 6.568985
## Sample15 23_25_June            2021      189   189 0.0000000 189.0000 3.933306
## Sample16 23_25_June            2021      328   328 0.0000000 328.0000 5.298124
## Sample17 25_28_June            2021      305   305 0.0000000 305.0000 5.200883
## Sample45       2022        20220617      286   286 0.0000000 286.0000 4.598951
## Sample46       2022        20220617      446   446 0.0000000 446.0000 6.423053
## Sample49       2022        20220617      321   321 0.2496103 321.1383 6.369213
## Sample53       2022        20220617      390   390 0.0000000 390.0000 5.570987
## Sample74       2022        20220623      368   368 0.0000000 368.0000 5.555060
## Sample79       2022        20220623      426   426 0.0000000 426.0000 5.952275
## Sample80       2022        20220623      334   334 0.0000000 334.0000 5.303089
## Sample81       2022        20220623      497   497 0.0000000 497.0000 6.585149
## Sample96       2022        20220624      280   280 0.0000000 280.0000 4.309458
##           Shannon   Simpson InvSimpson   Fisher log10_read_depth       PD
## Sample13 3.729559 0.9587400   24.23658 19.48925         4.944882 17.19547
## Sample14 5.473805 0.9898159   98.19204 91.31702         4.834808 40.97413
## Sample15 4.359369 0.9786393   46.81493 28.17905         4.429768 17.14734
## Sample16 4.680743 0.9767615   43.03207 49.57177         4.647647 29.47461
## Sample17 4.610111 0.9779298   45.30995 44.79549         4.814121 26.89777
## Sample45 4.259999 0.9700969   33.44138 38.14302         5.025462 26.24118
## Sample46 4.778056 0.9815483   54.19560 64.31891         4.943252 38.56611
## Sample49 3.947591 0.9549357   22.19049 42.90746         4.971044 29.43772
## Sample53 4.480025 0.9766741   42.87085 54.12566         4.985943 34.46851
## Sample74 4.673870 0.9737371   38.07646 50.85730         4.950491 36.71361
## Sample79 4.566878 0.9700116   33.34624 62.28957         4.904840 40.22847
## Sample80 4.641648 0.9752843   40.46008 47.66380         4.860248 29.87612
## Sample81 4.558365 0.9654703   28.96060 69.63458         5.034316 39.14362
## Sample96 4.593491 0.9801725   50.43494 40.35768         4.767586 23.83927
```

```
PSCO
```

```
##           sample.ID
## Sample18   Sample18
## Sample19   Sample19
## Sample41   Sample41
## Sample48   Sample48
## Sample90   Sample90
## Sample91   Sample91
## Sample92   Sample92
## Sample93   Sample93
## Sample94   Sample94
## Sample95   Sample95
## Sample102 Sample102
## Sample103 Sample103
##
## Sample18                                        PSCO-20210525-01-C-BP4.GTAA_GA.16S.R1.adapter_trimm
## Sample19                                    PSCO-20210525-02-C-BP4.GTAA_CTTT.16S.R1.adapter_trimmed
## Sample41                    AA.PSCO_J_L2-10-R10_BP4_01-06-2022_1.AA_GA.16S.R1.adapter_trimmed.fq.gz,AA
## Sample48                  HA.PSCO_J_L2-10-R8_BP4_01-06-2022_8.AA_AGAA.16S.R1.adapter_trimmed.fq.gz,HA.
## Sample90        YZ.STOD_M_L2-9-R1-3_BP4_02-06-2022_62.AAAGAA_TGT.16S.R1.adapter_trimmed.fq.gz,YZ.STOD_
## Sample91      ZA.PSCO_M_L2-10-R11_BP4_02-06-2022_63.AAAGAA_CTTT.16S.R1.adapter_trimmed.fq.gz,ZA.PSCO_M_
## Sample92  ZB.PSCO_J_L2-10-R12_BP4_02-06-2022_64.AAAGAA_TTTCA.16S.R1.adapter_trimmed.fq.gz,ZB.PSCO_J_
## Sample93  ZC.PSCO_J_L2-11-R8_BP4_02-06-2022_65.AAAGAA_AAAGAA.16S.R1.adapter_trimmed.fq.gz,ZC.PSCO_J_
## Sample94            ZD.PSCO_M_L2-11-R9_BP4_02-06-2022_66.AAAGAA_AT.16S.R1.adapter_trimmed.fq.gz,ZD.PSC
## Sample95        ZE.PSCO_J_L2-11-R10_BP4_02-06-2022_67.AAAGAA_GTC.16S.R1.adapter_trimmed.fq.gz,ZE.PSCO_
## Sample102         ZL.PSCO_J_L2-10-R10_BP4_02-06-2022_74.CGGAGA_AT.16S.R1.adapter_trimmed.fq.gz,ZL.PSCO
## Sample103           ZM.PSCO_F_L2-R1-8_BP4_02-06-2022_75.CGGAGA_GTC.16S.R1.adapter_trimmed.fq.gz,ZM.PSC
##           Read_depth  ProjectName  Region Sample_date Year Site Sample_type
## Sample18       79793 Atoka_turtle 16SV3V4    20210525 2021  BP4      turtle
## Sample19      116039 Atoka_turtle 16SV3V4    20210525 2021  BP4      turtle
## Sample41      111321 Atoka_turtle 16SV3V4    20220601 2022  BP4      turtle
## Sample48       75729 Atoka_turtle 16SV3V4    20220601 2022  BP4      turtle
## Sample90       14153 Atoka_turtle 16SV3V4    20220602 2022  BP4      turtle
## Sample91       92240 Atoka_turtle 16SV3V4    20220602 2022  BP4      turtle
## Sample92       82070 Atoka_turtle 16SV3V4    20220602 2022  BP4      turtle
## Sample93       69972 Atoka_turtle 16SV3V4    20220602 2022  BP4      turtle
## Sample94       85342 Atoka_turtle 16SV3V4    20220602 2022  BP4      turtle
## Sample95       62424 Atoka_turtle 16SV3V4    20220602 2022  BP4      turtle
## Sample102      85588 Atoka_turtle 16SV3V4    20220602 2022  BP4      turtle
## Sample103      74671 Atoka_turtle 16SV3V4    20220602 2022  BP4      turtle
##           Substrate Species Species_substrate Species_site Sorter Sex
## Sample18   carapace    PSCO     PSCO_carapace     PSCO_BP4      4   M
## Sample19   carapace    PSCO     PSCO_carapace     PSCO_BP4      5   J
## Sample41   carapace    PSCO     PSCO_carapace     PSCO_BP4     20   J
## Sample48   carapace    PSCO     PSCO_carapace     PSCO_BP4     27   J
## Sample90   carapace    PSCO     PSCO_carapace     PSCO_BP4     69   M
## Sample91   carapace    PSCO     PSCO_carapace     PSCO_BP4     70   M
## Sample92   carapace    PSCO     PSCO_carapace     PSCO_BP4     71   J
## Sample93   carapace    PSCO     PSCO_carapace     PSCO_BP4     72   J
## Sample94   carapace    PSCO     PSCO_carapace     PSCO_BP4     73   M
## Sample95   carapace    PSCO     PSCO_carapace     PSCO_BP4     74   J
## Sample102  carapace    PSCO     PSCO_carapace     PSCO_BP4     81   J
## Sample103  carapace    PSCO     PSCO_carapace     PSCO_BP4     82   F
```

```
##           Carapace_length Plastron_length Mass Sample_number Sex_notes
## Sample18            202.3           170.7  900             9      <NA>
## Sample19            133.4           122.0  335            11      <NA>
## Sample41            131.0           120.5  350             1      <NA>
## Sample48             86.0            79.5  110             8      <NA>
## Sample90             84.0            60.0   92            62      <NA>
## Sample91            188.0           166.0  740            63      <NA>
## Sample92            143.0           132.0  425            64      <NA>
## Sample93            137.0           126.0  360            65      <NA>
## Sample94            212.0           186.0 1050            66      <NA>
## Sample95            100.0            93.0  170            67      <NA>
## Sample102           131.0           120.0  320            74      <NA>
## Sample103           260.0           239.0 2400            75      <NA>
##            PCR1_date extraction_date Observed Chao1 se.chao1 ACE   se.ACE
## Sample18  17_22_June            2021      517   517        0 517 5.635958
## Sample19  17_22_June            2021      585   585        0 585 7.001099
## Sample41        2022        20220617      601   601        0 601 6.831706
## Sample48        2022        20220617      270   270        0 270 4.587019
## Sample90        2022        20220624      240   240        0 240 5.715112
## Sample91        2022        20220624      331   331        0 331 5.664326
## Sample92        2022        20220624      393   393        0 393 6.124659
## Sample93        2022        20220624      268   268        0 268 5.582489
## Sample94        2022        20220624      284   284        0 284 5.328636
## Sample95        2022        20220624      368   368        0 368 5.768873
## Sample102       2022        20220624      444   444        0 444 5.894722
## Sample103       2022        20220624      325   325        0 325 6.047504
##            Shannon   Simpson InvSimpson   Fisher log10_read_depth       PD
## Sample18  5.525254 0.9888374  89.584726 87.80640         4.901965 39.78667
## Sample19  4.800059 0.9753980  40.647034 85.96780         5.064604 47.39970
## Sample41  4.813076 0.9771733  43.808269 87.11125         5.046577 48.42494
## Sample48  4.200824 0.9634551  27.363619 37.60393         4.879262 24.20134
## Sample90  4.960056 0.9874806  79.875806 45.20184         4.150849 27.13225
## Sample91  4.033621 0.9498691  19.947793 46.64139         4.964919 33.04697
## Sample92  4.351399 0.9641941  27.928329 56.88421         4.914184 40.25559
## Sample93  3.893530 0.9477763  19.148390 38.27633         4.844924 32.86901
## Sample94  3.077930 0.8427348   6.358686 38.55823         4.931163 30.72251
## Sample95  4.145498 0.9050673  10.533782 55.97138         4.795352 35.49527
## Sample102 4.772747 0.9767108  42.938444 65.78764         4.932413 41.59403
## Sample103 3.530538 0.8956419   9.582386 45.81423         4.873152 33.78974
```

```
STCA
```

```
##          sample.ID
## Sample55  Sample55
## Sample62  Sample62
## Sample63  Sample63
## Sample64  Sample64
## Sample69  Sample69
## Sample70  Sample70
## Sample71  Sample71
## Sample72  Sample72
##
## Sample55            OA.STCA_M_L3-R8-11_S1_01-06-2022_15.TGG_GTC.16S.R1.adapter_trimmed.fq.gz,OA.STC
## Sample62            VA.STCA_M_L3-R9-10_S1_01-06-2022_30.GTAA_AT.16S.R1.adapter_trimmed.fq.gz,VA.STC
```

```
## Sample63           WA.STCA_M_L3-R9-11_S1_01-06-2022_31.GTAA_GTC.16S.R1.adapter_trimmed.fq.gz,WA.STCA_
## Sample64           XA.STCA_M_L1-3-R3_S1_01-06-2022_32.GTAA_AGAA.16S.R1.adapter_trimmed.fq.gz,XA.STCA_
## Sample69 YE.STCA_F_L3-R10-11_S1_02-06-2022_40.GAAAG_AAAGAA.16S.R1.adapter_trimmed.fq.gz,YE.STCA_F_L3-
## Sample70             YF.STCA_M_L2-3-11_S1_02-06-2022_41.GAAAG_AT.16S.R1.adapter_trimmed.fq.gz,YF.STCA
## Sample71           YG.STCA_M_L8-9-10_S1_02-06-2022_42.GAAAG_GTC.16S.R1.adapter_trimmed.fq.gz,YG.STCA_
## Sample72         YH.STCA_M_L8-9-11_S1_02-06-2022_43.GAAAG_AGAA.16S.R1.adapter_trimmed.fq.gz,YH.STCA_
##          Read_depth  ProjectName  Region Sample_date Year Site Sample_type
## Sample55      74145 Atoka_turtle 16SV3V4    20220601 2022   S1      turtle
## Sample62      90812 Atoka_turtle 16SV3V4    20220601 2022   S1      turtle
## Sample63      77522 Atoka_turtle 16SV3V4    20220601 2022   S1      turtle
## Sample64      75757 Atoka_turtle 16SV3V4    20220601 2022   S1      turtle
## Sample69      59419 Atoka_turtle 16SV3V4    20220602 2022   S1      turtle
## Sample70      73955 Atoka_turtle 16SV3V4    20220602 2022   S1      turtle
## Sample71      73859 Atoka_turtle 16SV3V4    20220602 2022   S1      turtle
## Sample72      64928 Atoka_turtle 16SV3V4    20220602 2022   S1      turtle
##          Substrate Species Species_substrate Species_site Sorter Sex
## Sample55  carapace    STCA     STCA_carapace      STCA_S1     35   M
## Sample62  carapace    STCA     STCA_carapace      STCA_S1     41   M
## Sample63  carapace    STCA     STCA_carapace      STCA_S1     42   M
## Sample64  carapace    STCA     STCA_carapace      STCA_S1     43   M
## Sample69  carapace    STCA     STCA_carapace      STCA_S1     47   F
## Sample70  carapace    STCA     STCA_carapace      STCA_S1     48   M
## Sample71  carapace    STCA     STCA_carapace      STCA_S1     49   M
## Sample72  carapace    STCA     STCA_carapace      STCA_S1     50   M
##          Carapace_length Plastron_length Mass Sample_number Sex_notes PCR1_date
## Sample55             136            90.0  360            15      <NA>      2022
## Sample62             122            80.0  240            30      <NA>      2022
## Sample63             131            87.0  340            31      <NA>      2022
## Sample64             124            84.0  220            32      <NA>      2022
## Sample69             108            80.0  170            40      <NA>      2022
## Sample70             129            85.0  305            41      <NA>      2022
## Sample71              88            60.5   92            42      <NA>      2022
## Sample72              94            64.0  105            43      <NA>      2022
##          extraction_date Observed Chao1 se.chao1 ACE   se.ACE  Shannon
## Sample55        20220617      631   631        0 631 6.048929 5.839527
## Sample62        20220623      654   654        0 654 6.055932 5.444472
## Sample63        20220623      670   670        0 670 6.479013 5.787988
## Sample64        20220623      284   284        0 284 4.597535 4.061982
## Sample69        20220623      402   402        0 402 5.796079 4.880329
## Sample70        20220623      596   596        0 596 5.421032 5.499574
## Sample71        20220623      555   555        0 555 5.649524 5.447503
## Sample72        20220623      296   296        0 296 4.784194 4.162619
##            Simpson InvSimpson    Fisher log10_read_depth       PD
## Sample55 0.9946624  187.35029 100.62621         4.870082 40.34252
## Sample62 0.9886082   87.78236 100.01243         4.958143 48.77418
## Sample63 0.9936091  156.47316 105.73627         4.889425 46.34968
## Sample64 0.9489557   19.59083  38.70694         4.879423 26.59955
## Sample69 0.9834242   60.32881  60.38552         4.773925 32.88001
## Sample70 0.9863427   73.22104  92.88732         4.868968 42.50499
## Sample71 0.9912641  114.47082  85.01679         4.868403 44.71480
## Sample72 0.9240635   13.16889  41.60438         4.812432 25.13339
```

```
STOD
```

```
##          sample.ID
## Sample20  Sample20
## Sample21  Sample21
## Sample24  Sample24
## Sample25  Sample25
## Sample26  Sample26
## Sample27  Sample27
## Sample28  Sample28
## Sample30  Sample30
## Sample32  Sample32
## Sample42  Sample42
## Sample44  Sample44
## Sample56  Sample56
## Sample65  Sample65
## Sample66  Sample66
## Sample68  Sample68
## Sample75  Sample75
## Sample76  Sample76
## Sample77  Sample77
## Sample78  Sample78
## Sample86  Sample86
## Sample87  Sample87
## Sample88  Sample88
## Sample89  Sample89
## Sample97  Sample97
## Sample98  Sample98
##
## Sample20                                          STOD-20210525-01-C-BP4.TGG_TGT.16S.R1.adapter_tri
## Sample21                                    STOD-20210525-01-C-S4.GTAA_AAAGAA.16S.R1.adapter_trimme
## Sample24                                    STOD-20210525-02-C-BP4.GTAA_TTTCA.16S.R1.adapter_trimme
## Sample25                                       STOD-20210525-02-C-S4.GTAA_AGAA.16S.R1.adapter_trim
## Sample26                                          STOD-20210525-03-C-S4.GAAAG_GA.16S.R1.adapter_tri
## Sample27                                       STOD-20210525-04-C-S4.GAAAG_CTTT.16S.R1.adapter_trimm
## Sample28                                          STOD-20210525-05-C-S4.GAAAG_AT.16S.R1.adapter_tri
## Sample30                                       STOD-20210525-06-C-S4.GAAAG_AGAA.16S.R1.adapter_trimm
## Sample32                                          STOD-20210526-01-C-BP4.CCAT_AT.16S.R1.adapter_tri
## Sample42               BA.STOD_F_L1-8-9-R11_BP4_01-06-2022_2.AA_TGT.16S.R1.adapter_trimmed.fq.gz,BA.S
## Sample44          DA.STOD_F_L2-9-10-R11_BP4_01-06-2022_4.AA_TTTCA.16S.R1.adapter_trimmed.fq.gz,DA.STO
## Sample56           PA.STOD_F_L2-9-10-R2_S1_01-06-2022_16.TGG_AGAA.16S.R1.adapter_trimmed.fq.gz,PA.ST
## Sample65           YA.STOD_F_L2-9-10-R3_S4_01-06-2022_33.GAAAG_GA.16S.R1.adapter_trimmed.fq.gz,YA.ST
## Sample66           YB.STOD_M_L1-8-9-R2_S4_01-06-2022_34.GAAAG_TGT.16S.R1.adapter_trimmed.fq.gz,YB.ST
## Sample68      YD.STOD_F_L1-8-11-R8_S1_02-06-2022_39.GAAAG_TTTCA.16S.R1.adapter_trimmed.fq.gz,YD.STOD_
## Sample75      YK.STOD_M_L2-9-10-R8_S4_02-06-2022_46.TCCTGA_CTTT.16S.R1.adapter_trimmed.fq.gz,YK.STOD_
## Sample76    YL.STOD_M_L2-9-11-R2_S4_02-06-2022_47.TCCTGA_TTTCA.16S.R1.adapter_trimmed.fq.gz,YL.STOD_M
## Sample77 YM.STOD_M_L2-9-11-R8_S4_02-06-2022_48.TCCTGA_AAAGAA.16S.R1.adapter_trimmed.fq.gz,YM.STOD_M_
## Sample78          YN.STOD_F_L2-9-11-R3_S4_02-06-2022_49.TCCTGA_AT.16S.R1.adapter_trimmed.fq.gz,YN.STO
## Sample86               YV.STOD_M_L2-9-11-R9_BP4_02-06-2022_58.GC_AT.16S.R1.adapter_trimmed.fq.gz,YV.
## Sample87             YW.STOD_M_L2-9-R1-10_BP4_02-06-2022_59.GC_GTC.16S.R1.adapter_trimmed.fq.gz,YW.S
## Sample88           YX.STOD_M_L2-9-R1-11_BP4_02-06-2022_60.GC_AGAA.16S.R1.adapter_trimmed.fq.gz,YX.ST
## Sample89          YY.STOD_M_L2-9-R1-2_BP4_02-06-2022_61.AAAGAA_GA.16S.R1.adapter_trimmed.fq.gz,YY.STO
## Sample97          ZG.STOD_F_L2-9-R1-8_BP4_02-06-2022_69.CGGAGA_GA.16S.R1.adapter_trimmed.fq.gz,ZG.STO
## Sample98        ZH.STOD_F_L1-8-9-10_BP4_02-06-2022_70.CGGAGA_TGT.16S.R1.adapter_trimmed.fq.gz,ZH.STOD
```

```
##          Read_depth  ProjectName  Region Sample_date Year Site Sample_type
## Sample20      59665 Atoka_turtle 16SV3V4    20210525 2021  BP4      turtle
## Sample21      88487 Atoka_turtle 16SV3V4    20210525 2021   S4      turtle
## Sample24      75128 Atoka_turtle 16SV3V4    20210525 2021  BP4      turtle
## Sample25      51275 Atoka_turtle 16SV3V4    20210525 2021   S4      turtle
## Sample26      46318 Atoka_turtle 16SV3V4    20210525 2021   S4      turtle
## Sample27      43416 Atoka_turtle 16SV3V4    20210525 2021   S4      turtle
## Sample28      36984 Atoka_turtle 16SV3V4    20210525 2021   S4      turtle
## Sample30      49867 Atoka_turtle 16SV3V4    20210525 2021   S4      turtle
## Sample32      83818 Atoka_turtle 16SV3V4    20210526 2021  BP4      turtle
## Sample42      77584 Atoka_turtle 16SV3V4    20220601 2022  BP4      turtle
## Sample44      90881 Atoka_turtle 16SV3V4    20220601 2022  BP4      turtle
## Sample56      74818 Atoka_turtle 16SV3V4    20220601 2022   S1      turtle
## Sample65      97182 Atoka_turtle 16SV3V4    20220601 2022   S4      turtle
## Sample66      71091 Atoka_turtle 16SV3V4    20220601 2022   S4      turtle
## Sample68      59218 Atoka_turtle 16SV3V4    20220602 2022   S1      turtle
## Sample75     110689 Atoka_turtle 16SV3V4    20220602 2022   S4      turtle
## Sample76      99483 Atoka_turtle 16SV3V4    20220602 2022   S4      turtle
## Sample77      94447 Atoka_turtle 16SV3V4    20220602 2022   S4      turtle
## Sample78      55843 Atoka_turtle 16SV3V4    20220602 2022   S4      turtle
## Sample86      85314 Atoka_turtle 16SV3V4    20220602 2022  BP4      turtle
## Sample87      72713 Atoka_turtle 16SV3V4    20220602 2022  BP4      turtle
## Sample88      77592 Atoka_turtle 16SV3V4    20220602 2022  BP4      turtle
## Sample89      87706 Atoka_turtle 16SV3V4    20220602 2022  BP4      turtle
## Sample97      98544 Atoka_turtle 16SV3V4    20220602 2022  BP4      turtle
## Sample98      61108 Atoka_turtle 16SV3V4    20220602 2022  BP4      turtle
##          Substrate Species Species_substrate Species_site Sorter Sex
## Sample20  carapace    STOD     STOD_carapace     STOD_BP4     NA   M
## Sample21  carapace    STOD     STOD_carapace      STOD_S4      8   M
## Sample24  carapace    STOD     STOD_carapace     STOD_BP4      6   J
## Sample25  carapace    STOD     STOD_carapace      STOD_S4      9   M
## Sample26  carapace    STOD     STOD_carapace      STOD_S4     10   F
## Sample27  carapace    STOD     STOD_carapace      STOD_S4     12   F
## Sample28  carapace    STOD     STOD_carapace      STOD_S4     14   F
## Sample30  carapace    STOD     STOD_carapace      STOD_S4     15   F
## Sample32  carapace    STOD     STOD_carapace     STOD_BP4     18   M
## Sample42  carapace    STOD     STOD_carapace     STOD_BP4     21   F
## Sample44  carapace    STOD     STOD_carapace     STOD_BP4     23   F
## Sample56  carapace    STOD     STOD_carapace      STOD_S1     33   F
## Sample65  carapace    STOD     STOD_carapace      STOD_S4     45   F
## Sample66  carapace    STOD     STOD_carapace      STOD_S4     44   M
## Sample68  carapace    STOD     STOD_carapace      STOD_S1     46   F
## Sample75  carapace    STOD     STOD_carapace      STOD_S4     53   M
## Sample76  carapace    STOD     STOD_carapace      STOD_S4     54   M
## Sample77  carapace    STOD     STOD_carapace      STOD_S4     56   M
## Sample78  carapace    STOD     STOD_carapace      STOD_S4     55   F
## Sample86  carapace    STOD     STOD_carapace     STOD_BP4     65   M
## Sample87  carapace    STOD     STOD_carapace     STOD_BP4     66   M
## Sample88  carapace    STOD     STOD_carapace     STOD_BP4     67   M
## Sample89  carapace    STOD     STOD_carapace     STOD_BP4     68   M
## Sample97  carapace    STOD     STOD_carapace     STOD_BP4     75   F
## Sample98  carapace    STOD     STOD_carapace     STOD_BP4     77   F
##          Carapace_length Plastron_length  Mass Sample_number
## Sample20            53.3            35.8  25.0             2
```

```
## Sample21           87.8            59.5 110.0             13
## Sample24           49.4            36.6  30.0             12
## Sample25           94.8            62.3 135.0             16
## Sample26           81.1            59.8 105.0             17
## Sample27           89.6            70.6 145.0             19
## Sample28           85.2            63.5 110.0             21
## Sample30           94.6            65.7 140.0             23
## Sample32           75.4            51.8  60.0             34
## Sample42           82.0            65.0 110.0              2
## Sample44           89.0            65.0 130.0              4
## Sample56           93.0            65.0 105.0             16
## Sample65           87.0            71.0 130.0             33
## Sample66           87.0            63.0 120.0             34
## Sample68           91.5            66.0 125.0             39
## Sample75           68.0            49.0  50.5             46
## Sample76           91.0            65.0 120.0             47
## Sample77           96.0            62.0 140.0             48
## Sample78           86.0            66.0 105.0             49
## Sample86           88.0            60.0 110.0             58
## Sample87          105.0            65.0 160.0             59
## Sample88           68.0            46.0  48.0             60
## Sample89           82.0            57.0  92.0             61
## Sample97           84.0            63.0 110.0             69
## Sample98           87.0            68.0 120.0             70
##                                                     Sex_notes  PCR1_date
## Sample20 waiting for confirmation on juvenile status from Ethan 15_17_June
## Sample21                                                 <NA> 17_22_June
## Sample24                                                  J/F 17_22_June
## Sample25                                                 <NA> 17_22_June
## Sample26                                                 <NA> 23-25_June
## Sample27                                                 <NA> 23-25_June
## Sample28                                                 <NA> 23-25_June
## Sample30                                                 <NA> 23-25_June
## Sample32                                                 <NA> 25-28_June
## Sample42                                                 <NA>       2022
## Sample44                                                 <NA>       2022
## Sample56                                                 <NA>       2022
## Sample65                                                 <NA>       2022
## Sample66                                                 <NA>       2022
## Sample68                                                 <NA>       2022
## Sample75                                                 <NA>       2022
## Sample76                                                 <NA>       2022
## Sample77                                                 <NA>       2022
## Sample78                                                 <NA>       2022
## Sample86                                                 <NA>       2022
## Sample87                                                 <NA>       2022
## Sample88                                                 <NA>       2022
## Sample89                                                 <NA>       2022
## Sample97                                                 <NA>       2022
## Sample98                                                 <NA>       2022
##          extraction_date Observed Chao1 se.chao1       ACE   se.ACE  Shannon
## Sample20            2021      676   676 0.000000  676.0000 5.988648 6.025051
## Sample21            2021      734   734 0.000000  734.0000 7.072994 5.643059
## Sample24            2021      697   697 0.000000  697.0000 6.282448 5.595305
```

```
## Sample25           2021      431   431 0.000000  431.0000 6.090958 5.152552
## Sample26           2021      392   392 0.000000  392.0000 6.490181 4.747819
## Sample27           2021      261   261 0.000000  261.0000 4.838384 4.596471
## Sample28           2021      455   455 0.000000  455.0000 8.496929 5.081142
## Sample30           2021      374   374 0.000000  374.0000 5.252959 4.751049
## Sample32           2021      675   675 0.000000  675.0000 6.988456 5.355532
## Sample42       20220617      915   915 0.000000  915.0000 7.879974 6.188546
## Sample44       20220617      683   683 0.000000  683.0000 8.358371 5.396230
## Sample56       20220617      435   435 0.499425  435.1429 4.227490 4.960318
## Sample65       20220623      703   703 0.000000  703.0000 6.814970 5.496547
## Sample66       20220623      763   763 0.000000  763.0000 7.657135 5.704296
## Sample68       20220623      422   422 0.000000  422.0000 5.591124 4.783837
## Sample75       20220623     1019  1019 0.000000 1019.0000 7.572871 6.231652
## Sample76       20220623      930   930 0.000000  930.0000 7.193560 6.044790
## Sample77       20220623      788   788 0.000000  788.0000 7.445231 5.918532
## Sample78       20220623      326   326 0.000000  326.0000 5.059159 4.696493
## Sample86       20220624      690   690 0.000000  690.0000 6.810052 5.824557
## Sample87       20220624      548   548 0.000000  548.0000 6.860444 5.473520
## Sample88       20220624      528   528 0.000000  528.0000 5.938447 5.380287
## Sample89       20220624      623   623 0.000000  623.0000 5.669609 5.703946
## Sample97       20220624      794   794 0.000000  794.0000 6.235613 5.618270
## Sample98       20220624      669   669 0.000000  669.0000 8.254545 5.727080
##            Simpson InvSimpson    Fisher log10_read_depth       PD
## Sample20 0.9956702  230.95492 115.02328         4.775720 48.04551
## Sample21 0.9891987   92.58167 114.04568         4.946879 48.98446
## Sample24 0.9865533   74.36756 112.36838         4.875802 48.43319
## Sample25 0.9860599   71.73543  66.71265         4.709906 33.61271
## Sample26 0.9770835   43.63678  60.35810         4.665750 30.47902
## Sample27 0.9824489   56.97646  37.81026         4.637650 25.27484
## Sample28 0.9854272   68.62085  75.28950         4.568014 36.79741
## Sample30 0.9772726   43.99972  56.42700         4.697813 31.01607
## Sample32 0.9802597   50.65767 106.00153         4.923337 47.08424
## Sample42 0.9951924  208.00274 153.60045         4.889772 56.99047
## Sample44 0.9877838   81.85872 105.38450         4.958473 49.03294
## Sample56 0.9801574   50.39651  63.48925         4.874006 31.78564
## Sample65 0.9882506   85.11108 106.85453         4.987586 51.24692
## Sample66 0.9867157   75.27691 123.97393         4.851815 54.43713
## Sample68 0.9745292   39.26061  63.30293         4.772454 31.25161
## Sample75 0.9953962  217.21363 163.13200         5.044104 64.46524
## Sample76 0.9937161  159.13705 150.36745         4.997749 62.95285
## Sample77 0.9941129  169.86217 125.44868         4.975188 55.60426
## Sample78 0.9780244   45.50500  48.02370         4.746969 31.39697
## Sample86 0.9934699  153.13623 109.95045         4.931020 47.93617
## Sample87 0.9912000  113.63649  85.61834         4.861612 45.01325
## Sample88 0.9859783   71.31811  83.20963         4.889817 40.50177
## Sample89 0.9930089  143.03945  97.49460         4.943029 48.33636
## Sample97 0.9867562   75.50683 124.04016         4.993630 53.87866
## Sample98 0.9928517  139.89380 111.03861         4.786098 49.99600
```

```
TRSC
```

```
##          sample.ID
## Sample33  Sample33
## Sample34  Sample34
```

```
## Sample36  Sample36
## Sample38  Sample38
## Sample39  Sample39
## Sample40  Sample40
## Sample43  Sample43
## Sample47  Sample47
## Sample50  Sample50
## Sample51  Sample51
## Sample52  Sample52
## Sample54  Sample54
## Sample57  Sample57
## Sample58  Sample58
## Sample59  Sample59
## Sample60  Sample60
## Sample61  Sample61
## Sample73  Sample73
## Sample82  Sample82
## Sample83  Sample83
## Sample84  Sample84
## Sample85  Sample85
##
## Sample33                                  TRSC-20210525-01-C-BP4.TGG_TTTCA.16S.R1.adapter_trimmed.f
## Sample34                                    TRSC-20210525-01-C-S4.CCAT_CTTT.16S.R1.adapter_trimmed.
## Sample36                                TRSC-20210525-02-C-BP4.TGG_AAAGAA.16S.R1.adapter_trimmed.fq
## Sample38                                    TRSC-20210525-03-C-BP4.TGG_AGAA.16S.R1.adapter_trimmed.
## Sample39                                    TRSC-20210525-04-C-BP4.GTAA_TGT.16S.R1.adapter_trimmed.
## Sample40                                  TRSC-20210526-01-C-BP4.CCAT_AAAGAA.16S.R1.adapter_trimmed.fq.
## Sample43            CA.TRSC_M_L3-4-7-8_BP4_01-06-2022_3.AA_CTTT.16S.R1.adapter_trimmed.fq.gz,CA.TRS
## Sample47        GA.TRSC_M_L4-7-8-9-10_BP4_01-06-2022_7.AA_GTC.16S.R1.adapter_trimmed.fq.gz,GA.TRSC_
## Sample50      JA.TRSC_F_L1-3-9-R12_BP4_01-06-2022_10.TGG_TGT.16S.R1.adapter_trimmed.fq.gz,JA.TRSC_F
## Sample51     KA.TRSC_F_L1-7-8-R10_BP4_01-06-2022_11.TGG_CTTT.16S.R1.adapter_trimmed.fq.gz,KA.TRSC_F_
## Sample52     LA.TRSC_M_L1-9-R3-9_BP4_01-06-2022_12.TGG_TTTCA.16S.R1.adapter_trimmed.fq.gz,LA.TRSC_M_
## Sample54           NA.TRSC_M_L1-9-R8-10_S1_01-06-2022_14.TGG_AT.16S.R1.adapter_trimmed.fq.gz,NA.TRSC
## Sample57        QA.TRSC_M_L1-9-R8-11_S1_01-06-2022_25.GTAA_GA.16S.R1.adapter_trimmed.fq.gz,QA.TRSC_
## Sample58      RA.TRSC_M_L1-9-R8-12_S1_01-06-2022_26.GTAA_TGT.16S.R1.adapter_trimmed.fq.gz,RA.TRSC_M
## Sample59        SA.TRSC_M_L9-10-R7_S1_01-06-2022_27.GTAA_CTTT.16S.R1.adapter_trimmed.fq.gz,SA.TRSC_
## Sample60   TA.TRSC_M_L1-9-R9-10_S1_01-06-2022_28.GTAA_TTTCA.16S.R1.adapter_trimmed.fq.gz,TA.TRSC_M_L
## Sample61 UA.TRSC_M_L1-9-R9-11_S1_01-06-2022_29.GTAA_AAAGAA.16S.R1.adapter_trimmed.fq.gz,UA.TRSC_M_L1
## Sample73      YI.TRSC_M_L11-R7-10_S4_02-06-2022_44.TCCTGA_GA.16S.R1.adapter_trimmed.fq.gz,YI.TRSC_M
## Sample82             YR.TRSC_M_L11-R2-8_S4_02-06-2022_53.GC_TGT.16S.R1.adapter_trimmed.fq.gz,YR.TR
## Sample83     YS.TRSC_F_L10-11-12-R3_S4_02-06-2022_54.GC_CTTT.16S.R1.adapter_trimmed.fq.gz,YS.TRSC_F_
## Sample84   YT.TRSC_F_L10-11-12-R8_S4_02-06-2022_55.GC_TTTCA.16S.R1.adapter_trimmed.fq.gz,YT.TRSC_F_L
## Sample85        YU.TRSC_M_L11-R1-7_S4_02-06-2022_56.GC_AAAGAA.16S.R1.adapter_trimmed.fq.gz,YU.TRSC_
##          Read_depth  ProjectName  Region Sample_date Year Site Sample_type
## Sample33      47711 Atoka_turtle 16SV3V4    20210525 2021  BP4      turtle
## Sample34      70488 Atoka_turtle 16SV3V4    20210525 2021   S4      turtle
## Sample36      75166 Atoka_turtle 16SV3V4    20210525 2021  BP4      turtle
## Sample38      59670 Atoka_turtle 16SV3V4    20210525 2021  BP4      turtle
## Sample39      70960 Atoka_turtle 16SV3V4    20210525 2021  BP4      turtle
## Sample40      86554 Atoka_turtle 16SV3V4    20210526 2021  BP4      turtle
## Sample43     121835 Atoka_turtle 16SV3V4    20220601 2022  BP4      turtle
## Sample47      84002 Atoka_turtle 16SV3V4    20220601 2022  BP4      turtle
## Sample50     112117 Atoka_turtle 16SV3V4    20220601 2022  BP4      turtle
## Sample51     118728 Atoka_turtle 16SV3V4    20220601 2022  BP4      turtle
```

```
## Sample52     120102 Atoka_turtle 16SV3V4    20220601 2022  BP4      turtle
## Sample54      78663 Atoka_turtle 16SV3V4    20220601 2022   S1      turtle
## Sample57      99783 Atoka_turtle 16SV3V4    20220601 2022   S1      turtle
## Sample58      85807 Atoka_turtle 16SV3V4    20220601 2022   S1      turtle
## Sample59      82423 Atoka_turtle 16SV3V4    20220601 2022   S1      turtle
## Sample60      93886 Atoka_turtle 16SV3V4    20220601 2022   S1      turtle
## Sample61      99906 Atoka_turtle 16SV3V4    20220601 2022   S1      turtle
## Sample73     111000 Atoka_turtle 16SV3V4    20220602 2022   S4      turtle
## Sample82      85925 Atoka_turtle 16SV3V4    20220602 2022   S4      turtle
## Sample83     103984 Atoka_turtle 16SV3V4    20220602 2022   S4      turtle
## Sample84      95570 Atoka_turtle 16SV3V4    20220602 2022   S4      turtle
## Sample85      91384 Atoka_turtle 16SV3V4    20220602 2022   S4      turtle
##          Substrate Species Species_substrate Species_site Sorter Sex
## Sample33  carapace    TRSC     TRSC_carapace     TRSC_BP4     NA   F
## Sample34  carapace    TRSC     TRSC_carapace      TRSC_S4     16   M
## Sample36  carapace    TRSC     TRSC_carapace     TRSC_BP4      1   F
## Sample38  carapace    TRSC     TRSC_carapace     TRSC_BP4      2   M
## Sample39  carapace    TRSC     TRSC_carapace     TRSC_BP4      3   F
## Sample40  carapace    TRSC     TRSC_carapace     TRSC_BP4     17   J
## Sample43  carapace    TRSC     TRSC_carapace     TRSC_BP4     22   M
## Sample47  carapace    TRSC     TRSC_carapace     TRSC_BP4     26   M
## Sample50  carapace    TRSC     TRSC_carapace     TRSC_BP4     28   F
## Sample51  carapace    TRSC     TRSC_carapace     TRSC_BP4     29   F
## Sample52  carapace    TRSC     TRSC_carapace     TRSC_BP4     30   M
## Sample54  carapace    TRSC     TRSC_carapace      TRSC_S1     34   M
## Sample57  carapace    TRSC     TRSC_carapace      TRSC_S1     36   M
## Sample58  carapace    TRSC     TRSC_carapace      TRSC_S1     37   M
## Sample59  carapace    TRSC     TRSC_carapace      TRSC_S1     38   M
## Sample60  carapace    TRSC     TRSC_carapace      TRSC_S1     39   M
## Sample61  carapace    TRSC     TRSC_carapace      TRSC_S1     40   M
## Sample73  carapace    TRSC     TRSC_carapace      TRSC_S4     51   M
## Sample82  carapace    TRSC     TRSC_carapace      TRSC_S4     60   M
## Sample83  carapace    TRSC     TRSC_carapace      TRSC_S4     61   F
## Sample84  carapace    TRSC     TRSC_carapace      TRSC_S4     62   F
## Sample85  carapace    TRSC     TRSC_carapace      TRSC_S4     63   M
##          Carapace_length Plastron_length Mass Sample_number Sex_notes
## Sample33           117.1           109.6  250             4      <NA>
## Sample34           170.7           156.9  775            25      <NA>
## Sample36           177.8           171.4  850             6      <NA>
## Sample38           203.7           181.5 1225             8      <NA>
## Sample39           153.9           142.8  560            10      <NA>
## Sample40            91.2            82.6  125            33       J/M
## Sample43           171.0           152.0  710             3      <NA>
## Sample47           187.5           170.0 1075             7      <NA>
## Sample50           164.0           149.0  680            10      <NA>
## Sample51           204.0           183.0 1250            11      <NA>
## Sample52           163.0           151.0  715            12      <NA>
## Sample54           199.0           180.0 1200            14      <NA>
## Sample57           160.0           152.0  610            25      <NA>
## Sample58           155.0           142.0  510            26      <NA>
## Sample59           188.0           168.0  915            27      <NA>
## Sample60           179.0           161.0  750            28      <NA>
## Sample61           178.0           159.0  690            29      <NA>
## Sample73           174.0           155.0  610            44      <NA>
```

```
## Sample82          176.0          152.0  695           53      <NA>
## Sample83          204.0          196.0 1250           54      <NA>
## Sample84          201.0          186.0 1200           55      <NA>
## Sample85          113.0          101.0  210           56      <NA>
##           PCR1_date extraction_date Observed Chao1  se.chao1    ACE   se.ACE
## Sample33 15_17_June            2021      300   300 0.0000000 300.00 4.608326
## Sample34 25-28_June            2021      412   412 0.0000000 412.00 6.570565
## Sample36 15_17_June            2021      478   478 0.0000000 478.00 4.581206
## Sample38 15_17_June            2021      416   416 0.0000000 416.00 6.144885
## Sample39 17_22_June            2021      488   488 0.0000000 488.00 6.985788
## Sample40 25-28_June            2021      442   442 0.0000000 442.00 5.525957
## Sample43       2022        20220617      274   274 0.0000000 274.00 4.851067
## Sample47       2022        20220617      427   427 0.0000000 427.00 6.406598
## Sample50       2022        20220617      631   631 0.0000000 631.00 6.120812
## Sample51       2022        20220617      381   381 0.0000000 381.00 5.490149
## Sample52       2022        20220617      470   470 0.0000000 470.00 6.315129
## Sample54       2022        20220617      857   857 0.0000000 857.00 5.021076
## Sample57       2022        20220617      832   832 0.0000000 832.00 5.201701
## Sample58       2022        20220617      528   528 0.0000000 528.00 6.217644
## Sample59       2022        20220617      798   798 0.4996866 798.14 5.758435
## Sample60       2022        20220617      597   597 0.0000000 597.00 6.889897
## Sample61       2022        20220623      578   578 0.0000000 578.00 4.286658
## Sample73       2022        20220623      799   799 0.0000000 799.00 6.308109
## Sample82       2022        20220623      880   880 0.0000000 880.00 7.301230
## Sample83       2022        20220623      716   716 0.0000000 716.00 6.944312
## Sample84       2022        20220624      759   759 0.0000000 759.00 6.573593
## Sample85       2022        20220624      540   540 0.0000000 540.00 6.223611
##            Shannon   Simpson InvSimpson    Fisher log10_read_depth       PD
## Sample33 4.470368 0.9723864  36.214070  44.32634         4.678619 29.23956
## Sample34 5.322351 0.9892672  93.172320  71.67752         4.848115 37.57921
## Sample36 4.928386 0.9823568  56.678980  71.59689         4.876021 41.44164
## Sample38 4.556993 0.9621423  26.414695  63.97328         4.775756 37.97643
## Sample39 4.743282 0.9408637  16.910073  78.47029         4.851014 39.91568
## Sample40 4.295072 0.9358084  15.578363  63.76856         4.937287 34.21276
## Sample43 3.471752 0.9024037  10.246291  35.35081         5.085772 31.45635
## Sample47 4.064682 0.9187198  12.303120  61.27496         4.924290 39.56349
## Sample50 4.650150 0.9571722  23.349335  93.26989         5.049671 51.39293
## Sample51 3.811282 0.9381818  16.176454  51.26438         5.074553 35.44358
## Sample52 3.476394 0.8855343   8.736243  64.85302         5.079550 43.83418
## Sample54 6.344899 0.9970245 336.078611 142.22115         4.895771 51.31546
## Sample57 5.379054 0.9444502  18.001872 131.33118         4.999057 51.96197
## Sample58 4.631640 0.9655145  28.997697  79.01687         4.933523 43.21795
## Sample59 6.061079 0.9942151 172.864803 130.69838         4.916048 47.98310
## Sample60 4.433831 0.9511532  20.472158  88.07033         4.972601 43.83628
## Sample61 4.686124 0.9452630  18.269195  85.00461         4.999592 42.34049
## Sample73 5.811079 0.9923224 130.249452 123.42333         5.045323 51.25406
## Sample82 5.930504 0.9928610 140.075028 142.22304         4.934120 58.88365
## Sample83 4.779026 0.9569248  23.215216 107.88713         5.016967 52.31315
## Sample84 5.535845 0.9858368  70.605490 118.04832         4.980322 53.90495
## Sample85 4.746614 0.9629594  26.997425  81.62241         4.960870 43.65233
```

Making stacked barplots for taxonomy

This code generates stacked taxonomy barplots for turtle-species/site combinations levels at the taxonomic rank of family. Only the top 17 most common taxa are indicated for each panel of plots. Warnings are disabled as use of microDecon to adjust data for contamination introduced fractional counts less than one.

```
phylo_carapace<-subset_samples(phylo,Substrate=="carapace")
p1 <- phyloseq::merge_samples(phylo_carapace, group = "Species_site") %>%
  comp_barplot(
    tax_level = "Family", n_taxa = 17, merge_other=FALSE, bar_outline_colour = "grey50",
    #sample_order = c( "TRSC", "STOD", "STCA", "PSCO", "KISU", "CHSE"),
    sample_order = c("TRSC_S4", "TRSC_S1", "TRSC_BP4", "STOD_S4", "STOD_S1", "STOD_BP4", "STCA_S1", "PS
    bar_width = 0.8
  ) +
  coord_flip() + labs(x = NULL, y = NULL)
p1
```

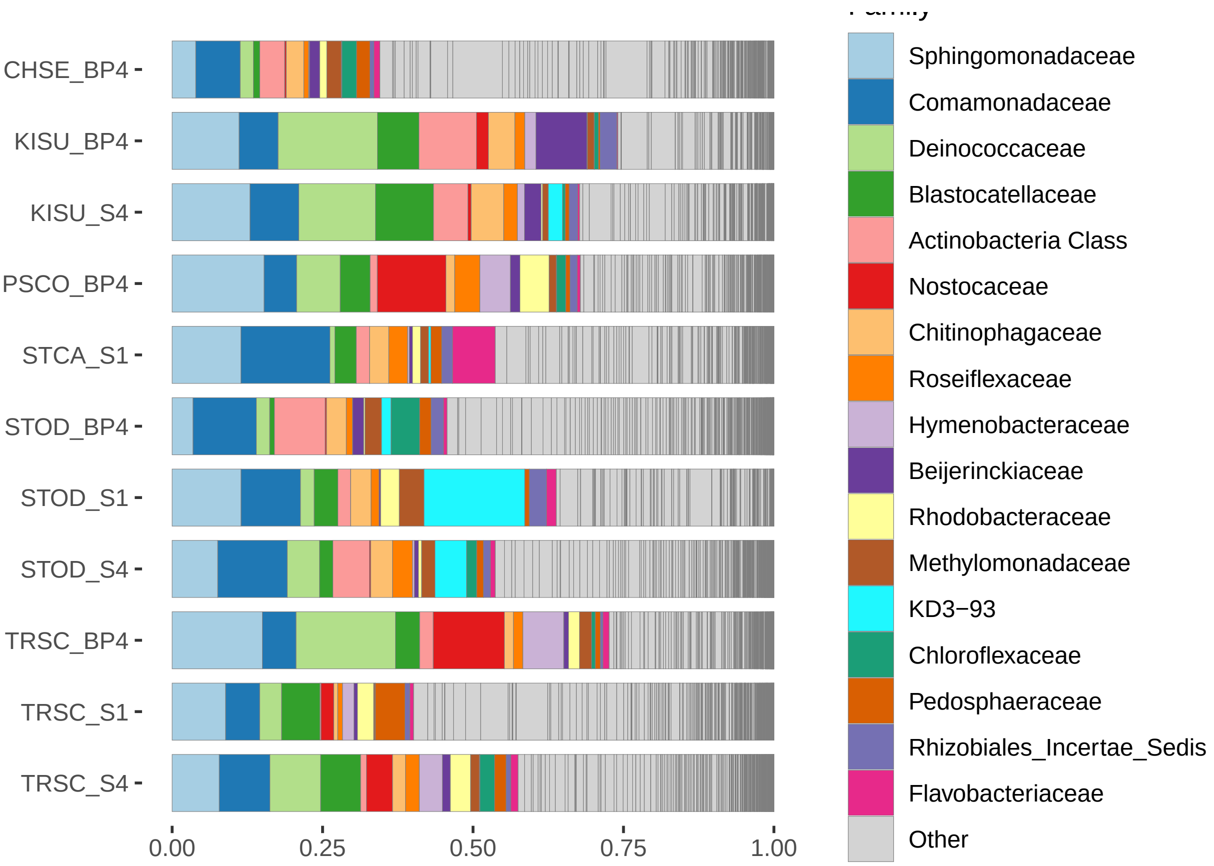

Extracting abundances for several taxa shared with Parks et al. (2020)

This codes includes merging ASVs into shared genera, and merging all samples from a turtle species into a single representative sample for the turtle species. The resulting abundances are for several taxa recovered in carapace microbiomes shared in common to those recovered by Parks et al. (2020) in sampling of Trachemys scripta shell microbial communities.

```r
phylo_carapace
```

```
## phyloseq-class experiment-level object
## otu_table()   OTU Table:         [ 4270 taxa and 84 samples ]
## sample_data() Sample Data:       [ 84 samples by 33 sample variables ]
## tax_table()   Taxonomy Table:    [ 4270 taxa by 7 taxonomic ranks ]
## phy_tree()    Phylogenetic Tree: [ 4270 tips and 4236 internal nodes ]
```

```r
genus = tax_glom(phylo_carapace, taxrank = "Genus")

genus <- merge_samples(genus, "Species")

# Use psmelt to obtain a long-format data.frame
genus_merged <- genus %>% tax_glom(taxrank = "Genus") %>% transform_sample_counts(function(x) {x/sum(x)
chse <- subset(genus_merged, Sample == "CHSE")
chse_deinococcus <- subset(chse, Genus == "Deinococcus")
chse_deinococcus
```

```
##                                   OTU Sample  Abundance sample.ID LibraryName
## 1775 56f57a074056606c1476f34453753d64   CHSE 0.01676918        NA          NA
##      Read_depth ProjectName Region Sample_date Year Site Sample_type Substrate
## 1775   80563.67          NA     NA    20220602 2022   NA          NA        NA
##      Species Species_substrate Species_site Sorter Sex Carapace_length
## 1775      NA                NA           NA     79  NA        249.3333
##      Plastron_length Mass Sample_number Sex_notes PCR1_date extraction_date
## 1775             184 3725            72        NA      2022        20220624
##      Observed Chao1 se.chao1 ACE   se.ACE  Shannon   Simpson InvSimpson
## 1775      590   590        0 590 5.520573 5.439946 0.9778343   92.08041
##        Fisher log10_read_depth       PD   Domain       Phylum      Class
## 1775 93.12043         4.904539 43.38569 Bacteria Deinococcota Deinococci
##             Order          Family       Genus
## 1775 Deinococcales Deinococcaceae Deinococcus
```

```r
chse_synechococcus <- subset(chse, Genus == "Synechococcus_PCC-7902")
chse_synechococcus
```

```
##                                   OTU Sample   Abundance sample.ID LibraryName
## 4643 ed6ea3b0d65425a8ab401ddc288363ff   CHSE 0.002344365        NA          NA
##      Read_depth ProjectName Region Sample_date Year Site Sample_type Substrate
## 4643   80563.67          NA     NA    20220602 2022   NA          NA        NA
##      Species Species_substrate Species_site Sorter Sex Carapace_length
## 4643      NA                NA           NA     79  NA        249.3333
##      Plastron_length Mass Sample_number Sex_notes PCR1_date extraction_date
## 4643             184 3725            72        NA      2022        20220624
##      Observed Chao1 se.chao1 ACE   se.ACE  Shannon   Simpson InvSimpson
## 4643      590   590        0 590 5.520573 5.439946 0.9778343   92.08041
##        Fisher log10_read_depth       PD   Domain        Phylum          Class
## 4643 93.12043         4.904539 43.38569 Bacteria Cyanobacteria Cyanobacteriia
##                 Order      Family                  Genus
## 4643 Cyanobacteriales Nostocaceae Synechococcus_PCC-7902
```

```r
kisu <- subset(genus_merged, Sample == "KISU")
kisu_deinococcus <- subset(kisu, Genus == "Deinococcus")
kisu_deinococcus
```

```
##                                   OTU Sample Abundance sample.ID LibraryName
## 1771 56f57a074056606c1476f34453753d64   KISU 0.1456303        NA          NA
##      Read_depth ProjectName Region Sample_date     Year Site Sample_type
## 1771   77565.64          NA     NA    20217003 2021.643   NA          NA
##      Substrate Species Species_substrate Species_site Sorter Sex
## 1771        NA      NA                NA           NA     NA  NA
##      Carapace_length Plastron_length     Mass Sample_number Sex_notes PCR1_date
## 1771        83.32143        73.84286 116.9286      27.71429        NA        NA
##      extraction_date Observed    Chao1    se.chao1      ACE   se.ACE  Shannon
## 1771        12999692 349.2857 349.2857 0.01782931 349.2956 5.381227 4.525251
##        Simpson InvSimpson   Fisher log10_read_depth       PD   Domain
## 1771 0.9735584   42.96873 50.26075         4.865315 30.72885 Bacteria
##            Phylum      Class         Order          Family       Genus
## 1771 Deinococcota Deinococci Deinococcales Deinococcaceae Deinococcus
```

```r
kisu_synechococcus <- subset(kisu, Genus == "Synechococcus_PCC-7902")
kisu_synechococcus
```

```
##                                   OTU Sample  Abundance sample.ID LibraryName
## 4644 ed6ea3b0d65425a8ab401ddc288363ff   KISU 0.01257471        NA          NA
##      Read_depth ProjectName Region Sample_date     Year Site Sample_type
## 4644   77565.64          NA     NA    20217003 2021.643   NA          NA
##      Substrate Species Species_substrate Species_site Sorter Sex
## 4644        NA      NA                NA           NA     NA  NA
##      Carapace_length Plastron_length     Mass Sample_number Sex_notes PCR1_date
## 4644        83.32143        73.84286 116.9286      27.71429        NA        NA
##      extraction_date Observed    Chao1    se.chao1      ACE   se.ACE  Shannon
## 4644        12999692 349.2857 349.2857 0.01782931 349.2956 5.381227 4.525251
##        Simpson InvSimpson   Fisher log10_read_depth       PD   Domain
## 4644 0.9735584   42.96873 50.26075         4.865315 30.72885 Bacteria
##            Phylum          Class            Order      Family
## 4644 Cyanobacteria Cyanobacteriia Cyanobacteriales Nostocaceae
##                       Genus
## 4644 Synechococcus_PCC-7902
```

```r
psco <- subset(genus_merged, Sample == "PSCO")
psco_deinococcus <- subset(psco, Genus == "Deinococcus")
psco_deinococcus
```

```
##                                   OTU Sample Abundance sample.ID LibraryName
## 1776 56f57a074056606c1476f34453753d64   PSCO 0.0720635        NA          NA
##      Read_depth ProjectName Region Sample_date     Year Site Sample_type
## 1776   79111.83          NA     NA    20218922 2021.833   NA          NA
##      Substrate Species Species_substrate Species_site Sorter Sex
## 1776        NA      NA                NA           NA     54  NA
##      Carapace_length Plastron_length     Mass Sample_number Sex_notes PCR1_date
## 1776        150.6417        134.5583 604.3333      47.08333        NA        NA
##      extraction_date Observed Chao1 se.chao1   ACE   se.ACE  Shannon   Simpson
```

```
## 1776        16850856    385.5 385.5        0 385.5 5.848509 4.342044 0.9478616
##      InvSimpson   Fisher log10_read_depth      PD   Domain       Phylum
## 1776   34.80977 57.63539          4.85828 36.2265 Bacteria Deinococcota
##           Class         Order         Family       Genus
## 1776 Deinococci Deinococcales Deinococcaceae Deinococcus
```

```
psco_synechococcus <- subset(psco, Genus == "Synechococcus_PCC-7902")
psco_synechococcus
```

```
##                                   OTU Sample Abundance sample.ID LibraryName
## 4639 ed6ea3b0d65425a8ab401ddc288363ff   PSCO 0.1098069        NA          NA
##      Read_depth ProjectName Region Sample_date     Year Site Sample_type
## 4639   79111.83          NA     NA    20218922 2021.833   NA          NA
##      Substrate Species Species_substrate Species_site Sorter Sex
## 4639        NA      NA                NA           NA     54  NA
##      Carapace_length Plastron_length     Mass Sample_number Sex_notes PCR1_date
## 4639        150.6417        134.5583 604.3333      47.08333        NA        NA
##      extraction_date Observed Chao1 se.chao1   ACE   se.ACE  Shannon   Simpson
## 4639        16850856    385.5 385.5        0 385.5 5.848509 4.342044 0.9478616
##      InvSimpson   Fisher log10_read_depth      PD   Domain        Phylum
## 4639   34.80977 57.63539          4.85828 36.2265 Bacteria Cyanobacteria
##               Class             Order      Family                  Genus
## 4639 Cyanobacteriia Cyanobacteriales Nostocaceae Synechococcus_PCC-7902
```

```
stca <- subset(genus_merged, Sample == "STCA")
stca_deinococcus <- subset(stca, Genus == "Deinococcus")
stca_deinococcus
```

```
##                                   OTU Sample   Abundance sample.ID LibraryName
## 1772 56f57a074056606c1476f34453753d64   STCA 0.006970875        NA          NA
##      Read_depth ProjectName Region Sample_date Year Site Sample_type Substrate
## 1772   73799.62          NA     NA    20220602 2022   NA          NA        NA
##      Species Species_substrate Species_site Sorter Sex Carapace_length
## 1772      NA                NA           NA 44.375  NA           116.5
##      Plastron_length Mass Sample_number Sex_notes PCR1_date extraction_date
## 1772         78.8125  229         34.25        NA      2022        20220622
##      Observed Chao1 se.chao1 ACE  se.ACE  Shannon   Simpson InvSimpson   Fisher
## 1772      511   511        0 511 5.60403 5.140499 0.9763662   89.04827 78.12198
##      log10_read_depth       PD   Domain       Phylum      Class         Order
## 1772           4.8651 38.41239 Bacteria Deinococcota Deinococci Deinococcales
##              Family       Genus
## 1772 Deinococcaceae Deinococcus
```

```
stca_synechococcus <- subset(stca, Genus == "Synechococcus_PCC-7902")
stca_synechococcus
```

```
##                                   OTU Sample    Abundance sample.ID LibraryName
## 4640 ed6ea3b0d65425a8ab401ddc288363ff   STCA 0.0001619552        NA          NA
##      Read_depth ProjectName Region Sample_date Year Site Sample_type Substrate
## 4640   73799.62          NA     NA    20220602 2022   NA          NA        NA
##      Species Species_substrate Species_site Sorter Sex Carapace_length
## 4640      NA                NA           NA 44.375  NA           116.5
```

```
##      Plastron_length Mass Sample_number Sex_notes PCR1_date extraction_date
## 4640         78.8125  229         34.25        NA      2022        20220622
##      Observed Chao1 se.chao1 ACE  se.ACE  Shannon   Simpson InvSimpson   Fisher
## 4640      511   511        0 511 5.60403 5.140499 0.9763662   89.04827 78.12198
##      log10_read_depth       PD   Domain        Phylum          Class
## 4640           4.8651 38.41239 Bacteria Cyanobacteria Cyanobacteriia
##                 Order      Family                  Genus
## 4640 Cyanobacteriales Nostocaceae Synechococcus_PCC-7902
```

```
stod <- subset(genus_merged, Sample == "STOD")
stod_deinococcus <- subset(stod, Genus == "Deinococcus")
stod_deinococcus
```

```
##                                   OTU Sample  Abundance sample.ID LibraryName
## 1773 56f57a074056606c1476f34453753d64   STOD 0.03192816        NA          NA
##      Read_depth ProjectName Region Sample_date    Year Site Sample_type
## 1773   73966.84          NA     NA    20216974 2021.64   NA          NA
##      Substrate Species Species_substrate Species_site Sorter Sex
## 1773        NA      NA                NA           NA     NA  NA
##      Carapace_length Plastron_length   Mass Sample_number Sex_notes PCR1_date
## 1773          83.828          60.064 105.42         34.08        NA        NA
##      extraction_date Observed  Chao1 se.chao1      ACE   se.ACE  Shannon
## 1773        12941926   621.24 621.24 0.019977 621.2457 6.602822 5.443875
##        Simpson InvSimpson   Fisher log10_read_depth       PD   Domain
## 1773 0.9870847   102.4675 98.35862         4.852168 44.98215 Bacteria
##            Phylum      Class         Order          Family       Genus
## 1773 Deinococcota Deinococci Deinococcales Deinococcaceae Deinococcus
```

```
stod_synechococcus <- subset(stod, Genus == "Synechococcus_PCC-7902")
stod_synechococcus
```

```
##                                   OTU Sample  Abundance sample.ID LibraryName
## 4642 ed6ea3b0d65425a8ab401ddc288363ff   STOD 0.00155643        NA          NA
##      Read_depth ProjectName Region Sample_date    Year Site Sample_type
## 4642   73966.84          NA     NA    20216974 2021.64   NA          NA
##      Substrate Species Species_substrate Species_site Sorter Sex
## 4642        NA      NA                NA           NA     NA  NA
##      Carapace_length Plastron_length   Mass Sample_number Sex_notes PCR1_date
## 4642          83.828          60.064 105.42         34.08        NA        NA
##      extraction_date Observed  Chao1 se.chao1      ACE   se.ACE  Shannon
## 4642        12941926   621.24 621.24 0.019977 621.2457 6.602822 5.443875
##        Simpson InvSimpson   Fisher log10_read_depth       PD   Domain
## 4642 0.9870847   102.4675 98.35862         4.852168 44.98215 Bacteria
##             Phylum          Class            Order      Family
## 4642 Cyanobacteria Cyanobacteriia Cyanobacteriales Nostocaceae
##                       Genus
## 4642 Synechococcus_PCC-7902
```

```
trsc <- subset(genus_merged, Sample == "TRSC")
trsc_deinococcus <- subset(trsc, Genus == "Deinococcus")
trsc_deinococcus
```

```
##                                   OTU Sample Abundance sample.ID LibraryName
## 1774 56f57a074056606c1476f34453753d64   TRSC 0.1077818        NA          NA
##      Read_depth ProjectName Region Sample_date     Year Site Sample_type
## 1774      90712          NA     NA    20217853 2021.727   NA          NA
##      Substrate Species Species_substrate Species_site Sorter Sex
## 1774        NA      NA                NA           NA     NA  NA
##      Carapace_length Plastron_length     Mass Sample_number Sex_notes PCR1_date
## 1774        169.5864        154.6273 766.1364      24.54545        NA        NA
##      extraction_date Observed    Chao1    se.chao1    ACE   se.ACE  Shannon
## 1774        14706456 572.8636 572.8636 0.02271303 572.87 5.923943 4.824109
##        Simpson InvSimpson   Fisher log10_read_depth       PD   Domain
## 1774 0.9578801   59.16395 87.69876         4.947038 43.75996 Bacteria
##            Phylum      Class         Order         Family       Genus
## 1774 Deinococcota Deinococci Deinococcales Deinococcaceae Deinococcus
```

```
trsc_synechococcus <- subset(trsc, Genus == "Synechococcus_PCC-7902")
trsc_synechococcus
```

```
##                                   OTU Sample  Abundance sample.ID LibraryName
## 4641 ed6ea3b0d65425a8ab401ddc288363ff   TRSC 0.06990532        NA          NA
##      Read_depth ProjectName Region Sample_date     Year Site Sample_type
## 4641      90712          NA     NA    20217853 2021.727   NA          NA
##      Substrate Species Species_substrate Species_site Sorter Sex
## 4641        NA      NA                NA           NA     NA  NA
##      Carapace_length Plastron_length     Mass Sample_number Sex_notes PCR1_date
## 4641        169.5864        154.6273 766.1364      24.54545        NA        NA
##      extraction_date Observed    Chao1    se.chao1    ACE   se.ACE  Shannon
## 4641        14706456 572.8636 572.8636 0.02271303 572.87 5.923943 4.824109
##        Simpson InvSimpson   Fisher log10_read_depth       PD   Domain
## 4641 0.9578801   59.16395 87.69876         4.947038 43.75996 Bacteria
##             Phylum          Class            Order      Family
## 4641 Cyanobacteria Cyanobacteriia Cyanobacteriales Nostocaceae
##                       Genus
## 4641 Synechococcus_PCC-7902
```

Statistical testing for alpha diversity significance

ANOVA Testing is done first for Shannon diversity with all carapace samples and then again after removing standardized residual outliers. Factors tested are turtle species, collection site, turtle sex and (log10) read depth. This is repeated for (log10) observed features and Faith's Phylogenetic Diversity.

Turtle species and plastron length showed consistently high to moderate levels of collinearity. Because of this, plastron length was not used as a factor, but was subsequently checked for any effects within individual species (see next section).

Q-Q and residual plots, among other diagnostics, are used to assess assumptions of normality. P-values are Tukey-adjusted for pairwise comparisons in ANOVA analyses.

```
#Testing for Shannon diversity, all samples included
#NOTE: Reordering the factors in the below model does not change the resulting p-values
pl.shannon.lm <- lm(Shannon~Species+Site+Plastron_length+Sex+log10_read_depth+Year,data=carapace)
#NOTE: running the above model, both Species and Plastron_length have high or moderate levels of collin
#Because of this, Plastron_length is taken out of the model and is checked later for any effects within
```

```r
pl.shannon.lm <- lm(Shannon~Species+Site+Sex+log10_read_depth+Year,data=carapace)

summary(pl.shannon.lm)
```

```
##
## Call:
## lm(formula = Shannon ~ Species + Site + Sex + log10_read_depth +
##     Year, data = carapace)
##
## Residuals:
##      Min       1Q   Median       3Q      Max
## -1.27252 -0.43228  0.02523  0.36420  1.29749
##
## Coefficients:
##                   Estimate Std. Error t value Pr(>|t|)
## (Intercept)       36.43468  376.10846   0.097   0.9231
## SpeciesKISU       -1.03081    0.40740  -2.530   0.0136 *
## SpeciesPSCO       -1.17073    0.43606  -2.685   0.0090 **
## SpeciesSTCA       -0.68116    0.49635  -1.372   0.1742
## SpeciesSTOD       -0.13726    0.39410  -0.348   0.7286
## SpeciesTRSC       -0.80752    0.40169  -2.010   0.0481 *
## SiteS1             0.33855    0.26958   1.256   0.2132
## SiteS4             0.30926    0.17418   1.776   0.0800 .
## SexJ               0.26417    0.31800   0.831   0.4089
## SexM               0.23286    0.15679   1.485   0.1419
## log10_read_depth   0.13565    0.57350   0.237   0.8137
## Year              -0.01573    0.18661  -0.084   0.9330
## ---
## Signif. codes:  0 '***' 0.001 '**' 0.01 '*' 0.05 '.' 0.1 ' ' 1
##
## Residual standard error: 0.6184 on 72 degrees of freedom
## Multiple R-squared:  0.3803, Adjusted R-squared:  0.2856
## F-statistic: 4.016 on 11 and 72 DF,  p-value: 0.0001404
```

```r
confint(pl.shannon.lm)
```

```
##                          2.5 %       97.5 %
## (Intercept)      -713.32382684 786.19318847
## SpeciesKISU        -1.84295411  -0.21867514
## SpeciesPSCO        -2.04000689  -0.30145171
## SpeciesSTCA        -1.67061447   0.30828887
## SpeciesSTOD        -0.92288308   0.64836228
## SpeciesTRSC        -1.60826670  -0.00677576
## SiteS1             -0.19884751   0.87595575
## SiteS4             -0.03795518   0.65647876
## SexJ               -0.36974700   0.89809100
## SexM               -0.07969749   0.54541537
## log10_read_depth   -1.00760591   1.27891308
## Year               -0.38774124   0.35627211
```

```r
check_model(pl.shannon.lm)
```

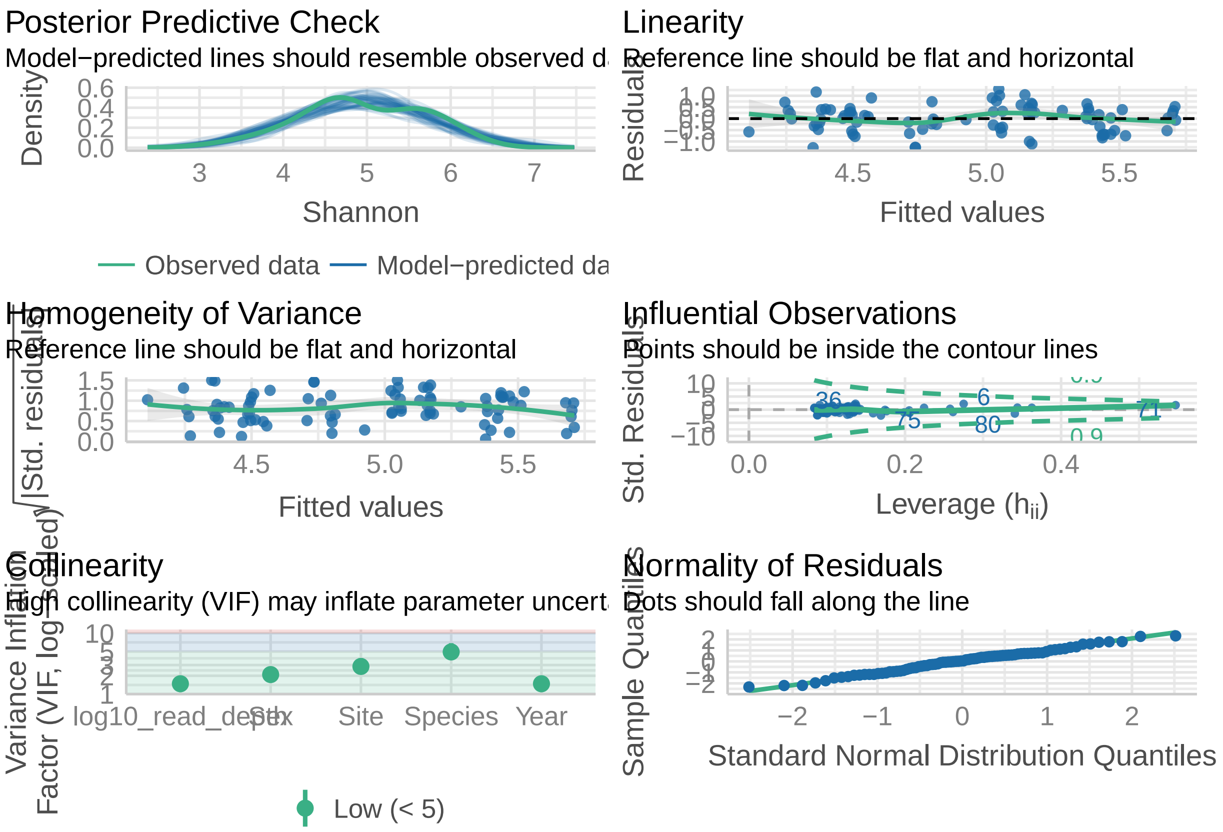


```r
plot(pl.shannon.lm, which = 1)
```

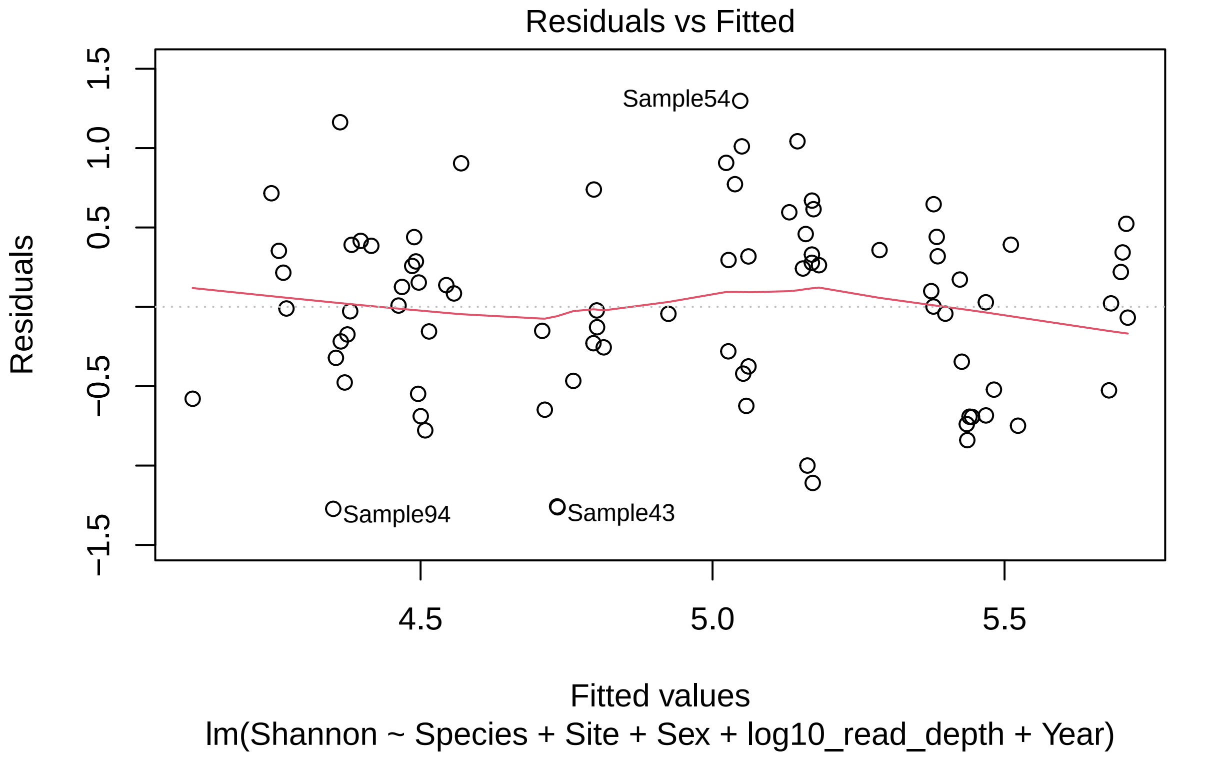

```r
plot(pl.shannon.lm, which = 2)
```

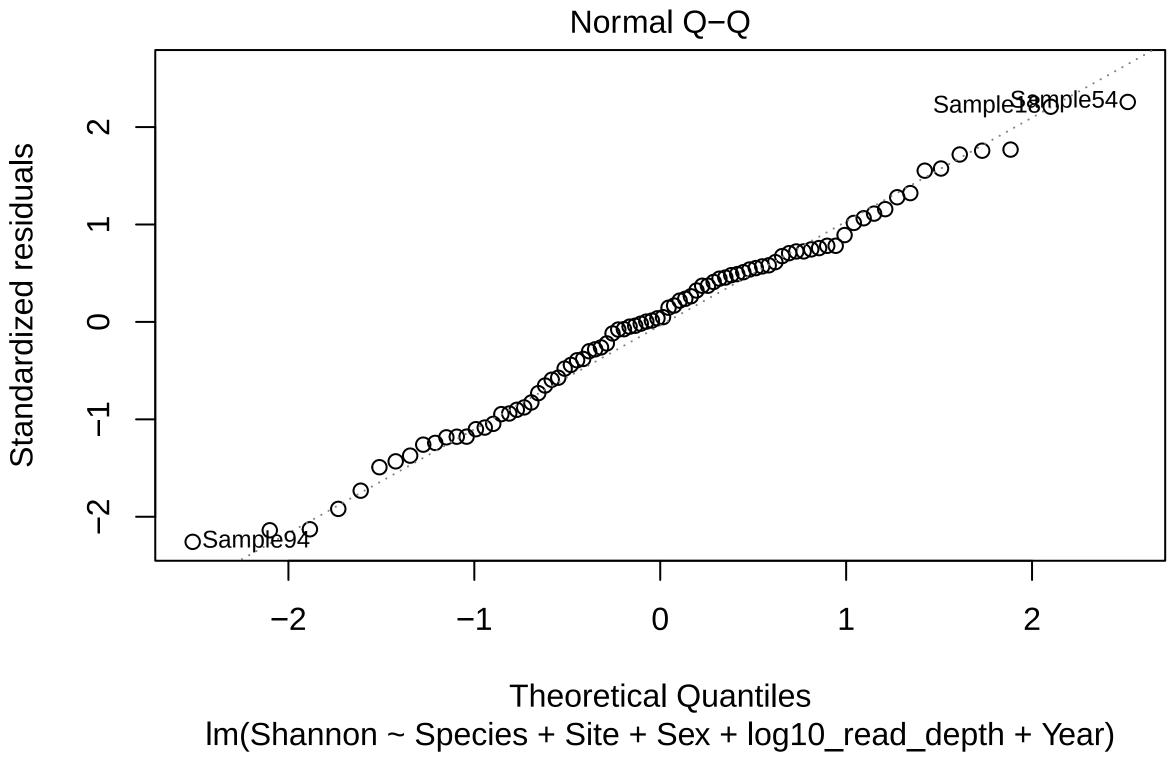


```r
ols_plot_resid_stand(pl.shannon.lm)
```

Table 1: Result: ANOVA comparing Shannon diversity of turtle species by different factors

	Sum Sq	Df	F value	Pr(>F)
Species	12.1142635	5	6.3359524	0.0000629
Site	1.4446211	2	1.8888995	0.1586567
Sex	0.8775711	2	1.1474591	0.3231781
log10_read_depth	0.0213946	1	0.0559486	0.8136895
Year	0.0027186	1	0.0071093	0.9330388
Residuals	27.5326238	72	NA	NA

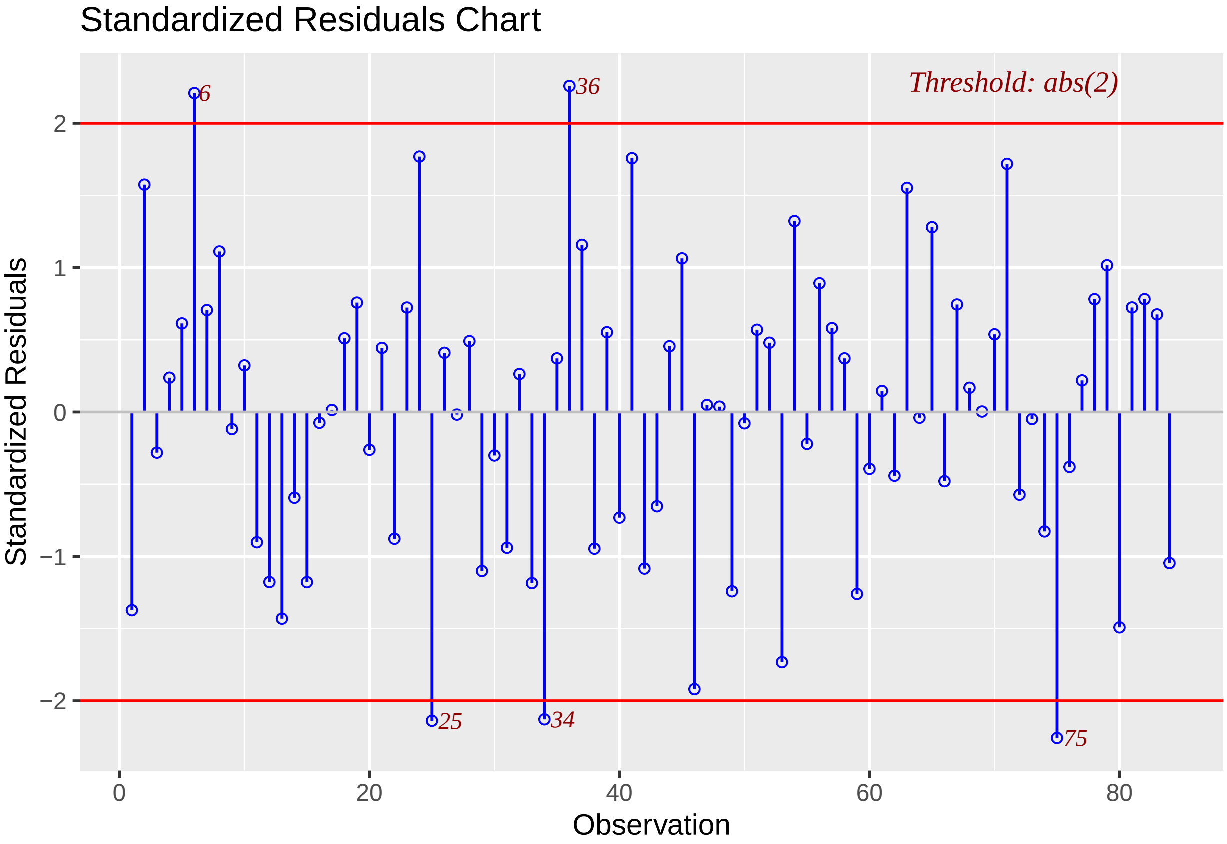


```
#ANOVA with pairwise comparisons (Tukey adjustment for pairwise comparisons):
kable.wrap(Anova(pl.shannon.lm),"Result: ANOVA comparing Shannon diversity of turtle species by differe
```

```
emmeans(pl.shannon.lm, list(pairwise ~ Species), adjust = "tukey")
```

```
## $`emmeans of Species`
##  Species emmean    SE df lower.CL upper.CL
##  CHSE      5.67 0.401 72     4.87     6.47
##  KISU      4.64 0.212 72     4.22     5.06
```

```
##  PSCO      4.50 0.251 72     4.00     5.00
##  STCA      4.99 0.290 72     4.41     5.57
##  STOD      5.53 0.167 72     5.20     5.87
##  TRSC      4.86 0.164 72     4.54     5.19
##
## Results are averaged over the levels of: Site, Sex, Year
## Confidence level used: 0.95
##
## $`pairwise differences of Species`
##  1            estimate    SE df t.ratio p.value
##  CHSE - KISU    1.031 0.407 72   2.530  0.1290
##  CHSE - PSCO    1.171 0.436 72   2.685  0.0909
##  CHSE - STCA    0.681 0.496 72   1.372  0.7432
##  CHSE - STOD    0.137 0.394 72   0.348  0.9993
##  CHSE - TRSC    0.808 0.402 72   2.010  0.3468
##  KISU - PSCO    0.140 0.317 72   0.442  0.9978
##  KISU - STCA   -0.350 0.372 72  -0.940  0.9347
##  KISU - STOD   -0.894 0.208 72  -4.297  0.0007
##  KISU - TRSC   -0.223 0.227 72  -0.982  0.9224
##  PSCO - STCA   -0.490 0.417 72  -1.173  0.8480
##  PSCO - STOD   -1.033 0.291 72  -3.554  0.0085
##  PSCO - TRSC   -0.363 0.308 72  -1.181  0.8445
##  STCA - STOD   -0.544 0.342 72  -1.592  0.6063
##  STCA - TRSC    0.126 0.317 72   0.399  0.9986
##  STOD - TRSC    0.670 0.195 72   3.441  0.0120
##
## Results are averaged over the levels of: Site, Sex, Year
## P value adjustment: tukey method for comparing a family of 6 estimates
```

```
emmeans(pl.shannon.lm, list(pairwise ~ Site), adjust = "tukey")
```

```
## $`emmeans of Site`
##  Site emmean    SE df lower.CL upper.CL
##  BP4    4.82 0.131 72     4.56     5.08
##  S1     5.16 0.247 72     4.66     5.65
##  S4     5.13 0.176 72     4.78     5.48
##
## Results are averaged over the levels of: Species, Sex, Year
## Confidence level used: 0.95
##
## $`pairwise differences of Site`
##  1         estimate    SE df t.ratio p.value
##  BP4 - S1  -0.3386 0.270 72  -1.256  0.4247
##  BP4 - S4  -0.3093 0.174 72  -1.776  0.1851
##  S1 - S4    0.0293 0.277 72   0.106  0.9939
##
## Results are averaged over the levels of: Species, Sex, Year
## P value adjustment: tukey method for comparing a family of 3 estimates
```

```
#Rerun above analysis, but with standardized residual outliers removed
carapace_shannon_no_outliers<-carapace[-c(6,25,34,36,75),]
summary(carapace_shannon_no_outliers)
```

```
##    sample.ID         LibraryName          Read_depth     ProjectName
```

```
##  Length:79          Length:79          Min.   : 14153   Length:79
##  Class :character   Class :character   1st Qu.: 69166   Class :character
##  Mode  :character   Mode  :character   Median : 80323   Mode  :character
##                                        Mean   : 78816
##                                        3rd Qu.: 91812
##                                        Max.   :118728
##
##     Region           Sample_date           Year          Site
##  Length:79          Min.   :20210525   Min.   :2021   Length:79
##  Class :character   1st Qu.:20210526   1st Qu.:2021   Class :character
##  Mode  :character   Median :20220601   Median :2022   Mode  :character
##                     Mean   :20217923   Mean   :2022
##                     3rd Qu.:20220602   3rd Qu.:2022
##                     Max.   :20220602   Max.   :2022
##
##  Sample_type         Substrate          Species          Species_substrate
##  Length:79          Length:79          Length:79          Length:79
##  Class :character   Class :character   Class :character   Class :character
##  Mode  :character   Mode  :character   Mode  :character   Mode  :character
##
##
##
##
##  Species_site          Sorter          Sex            Carapace_length
##  Length:79          Min.   : 1.00   Length:79          Min.   : 49.4
##  Class :character   1st Qu.:20.75   Class :character   1st Qu.: 86.9
##  Mode  :character   Median :42.50   Mode  :character   Median : 94.8
##                     Mean   :41.79                      Mean   :120.7
##                     3rd Qu.:61.25                      3rd Qu.:154.4
##                     Max.   :82.00                      Max.   :308.0
##                     NA's   :3
##  Plastron_length       Mass      Sample_number    Sex_notes
##  Min.   : 35.80   Min.   :  25   Min.   : 1.00   Length:79
##  1st Qu.: 64.50   1st Qu.: 110   1st Qu.:14.00   Class :character
##  Median : 80.00   Median : 145   Median :33.00   Mode  :character
##  Mean   : 99.93   Mean   : 466   Mean   :34.57
##  3rd Qu.:142.40   3rd Qu.: 535   3rd Qu.:53.50
##  Max.   :239.00   Max.   :6600   Max.   :75.00
##
##   PCR1_date         extraction_date       Observed          Chao1
##  Length:79          Min.   :    2021   Min.   : 146.0   Min.   : 146.0
##  Class :character   1st Qu.:    2021   1st Qu.: 371.0   1st Qu.: 371.0
##  Mode  :character   Median :20220623   Median : 497.0   Median : 497.0
##                     Mean   :14846057   Mean   : 520.3   Mean   : 520.3
##                     3rd Qu.:20220623   3rd Qu.: 669.5   3rd Qu.: 669.5
##                     Max.   :20220624   Max.   :1019.0   Max.   :1019.0
##
##     se.chao1           ACE             se.ACE         Shannon
##  Min.   :0.00000   Min.   : 146.0   Min.   :3.669   Min.   :3.531
##  1st Qu.:0.00000   1st Qu.: 371.0   1st Qu.:5.362   1st Qu.:4.563
##  Median :0.00000   Median : 497.0   Median :6.049   Median :4.784
##  Mean   :0.01581   Mean   : 520.3   Mean   :6.015   Mean   :4.978
##  3rd Qu.:0.00000   3rd Qu.: 669.5   3rd Qu.:6.595   3rd Qu.:5.518
##  Max.   :0.49969   Max.   :1019.0   Max.   :8.497   Max.   :6.232
```

```
##
##     Simpson          InvSimpson          Fisher        log10_read_depth
##  Min.   :0.8956   Min.   :  9.582   Min.   : 19.49   Min.   :4.151
##  1st Qu.:0.9632   1st Qu.: 27.181   1st Qu.: 52.70   1st Qu.:4.840
##  Median :0.9786   Median : 46.815   Median : 78.47   Median :4.905
##  Mean   :0.9732   Mean   : 68.610   Mean   : 79.99   Mean   :4.877
##  3rd Qu.:0.9889   3rd Qu.: 90.182   3rd Qu.:105.87   3rd Qu.:4.963
##  Max.   :0.9957   Max.   :230.955   Max.   :163.13   Max.   :5.075
##
##        PD
##  Min.   :17.15
##  1st Qu.:32.87
##  Median :40.50
##  Mean   :40.41
##  3rd Qu.:48.19
##  Max.   :64.47
##
```

```
pl.shannon_no_outliers.lm <- lm(Shannon~Species+Site+Sex+log10_read_depth+Year,data=carapace_shannon_no_

summary(pl.shannon_no_outliers.lm)
```

```
##
## Call:
## lm(formula = Shannon ~ Species + Site + Sex + log10_read_depth +
##     Year, data = carapace_shannon_no_outliers)
##
## Residuals:
##      Min       1Q   Median       3Q      Max
## -1.11427 -0.28472 -0.01211  0.34544  1.15944
##
## Coefficients:
##                    Estimate Std. Error t value Pr(>|t|)
## (Intercept)      -2.581e+02  3.484e+02  -0.741  0.46136
## SpeciesKISU      -9.278e-01  3.484e-01  -2.663  0.00968 **
## SpeciesPSCO      -1.108e+00  4.058e-01  -2.731  0.00806 **
## SpeciesSTCA      -2.808e-01  4.318e-01  -0.650  0.51774
## SpeciesSTOD      -4.392e-04  3.373e-01  -0.001  0.99896
## SpeciesTRSC      -5.643e-01  3.487e-01  -1.618  0.11033
## SiteS1           -6.291e-02  2.448e-01  -0.257  0.79796
## SiteS4            2.239e-01  1.504e-01   1.489  0.14119
## SexJ              2.369e-01  3.040e-01   0.779  0.43849
## SexM              2.583e-01  1.355e-01   1.906  0.06088 .
## log10_read_depth  2.420e-01  5.128e-01   0.472  0.63858
## Year              1.297e-01  1.729e-01   0.750  0.45590
## ---
## Signif. codes:  0 '***' 0.001 '**' 0.01 '*' 0.05 '.' 0.1 ' ' 1
##
## Residual standard error: 0.5275 on 67 degrees of freedom
## Multiple R-squared:  0.4554, Adjusted R-squared:  0.366
## F-statistic: 5.094 on 11 and 67 DF,  p-value: 9.726e-06
```

```r
confint(pl.shannon_no_outliers.lm)
```

```
##                          2.5 %      97.5 %
## (Intercept)      -953.53620092 437.2890847
## SpeciesKISU        -1.62317613  -0.2324561
## SpeciesPSCO        -1.91803727  -0.2982335
## SpeciesSTCA        -1.14273339   0.5811219
## SpeciesSTOD        -0.67363479   0.6727565
## SpeciesTRSC        -1.26032247   0.1317665
## SiteS1             -0.55146808   0.4256529
## SiteS4             -0.07626588   0.5241573
## SexJ               -0.36981390   0.8436423
## SexM               -0.01213618   0.5287109
## log10_read_depth   -0.78163034   1.2655692
## Year               -0.21544799   0.4747997
```

```r
check_model(pl.shannon_no_outliers.lm)
```

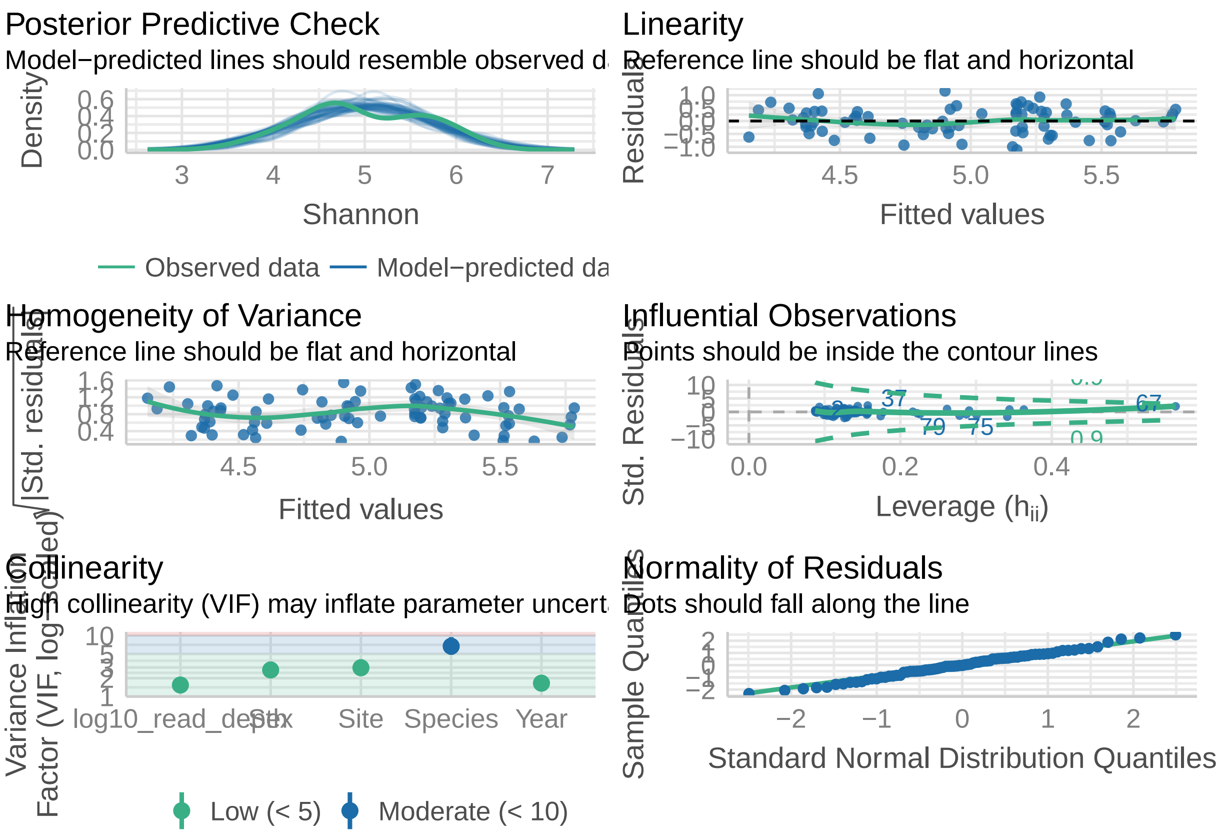


```r
plot(pl.shannon_no_outliers.lm, which = 1)
```

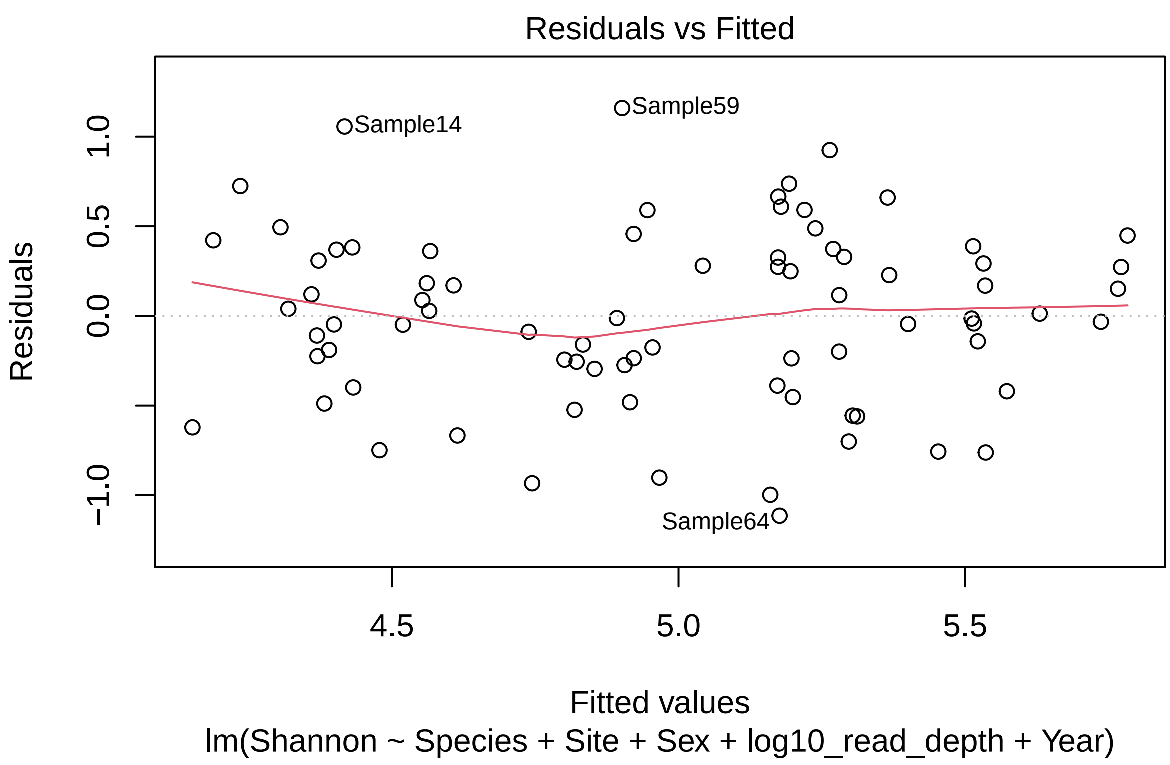


```
plot(pl.shannon_no_outliers.lm, which = 2)
```

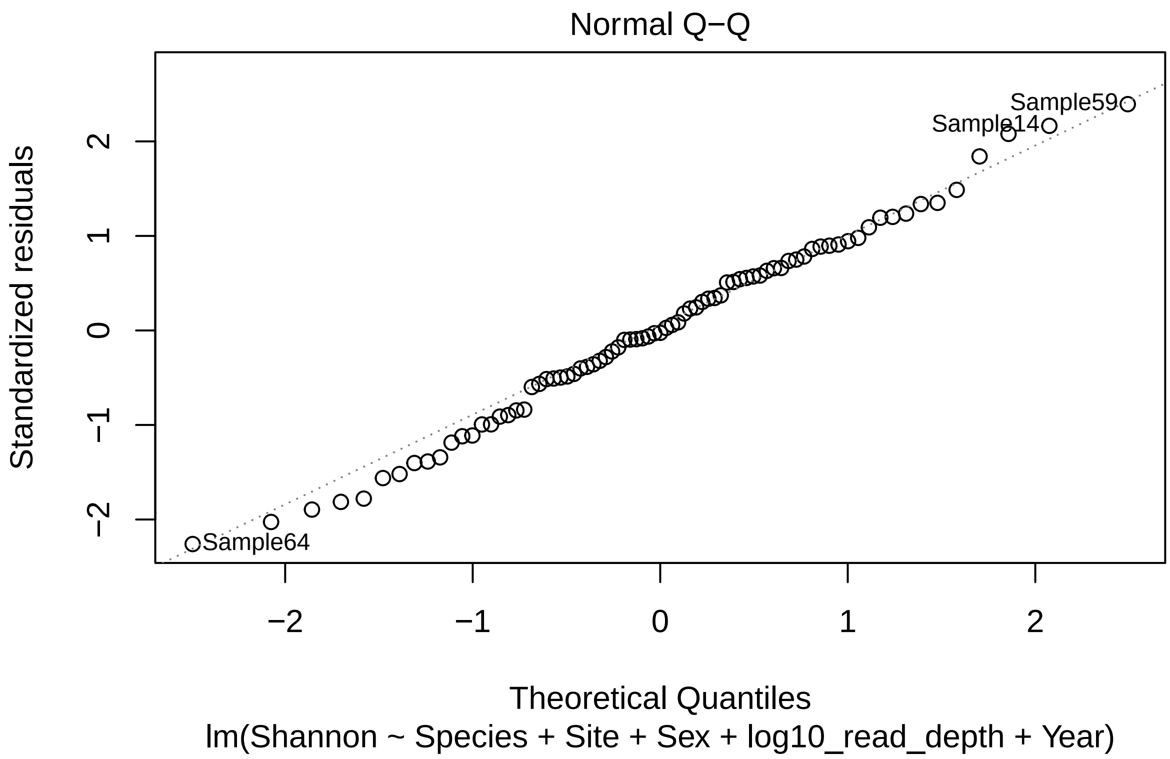


```
#ANOVA with pairwise comparisons (Tukey adjustment for pairwise comparisons):
kable.wrap(Anova(pl.shannon_no_outliers.lm),"Result: ANOVA comparing Shannon diversity of turtle species
```

Table 2: Result: ANOVA comparing Shannon diversity of turtle species by different factors, standardized residual outliers removed

	Sum Sq	Df	F value	Pr(>F)
Species	11.2057569	5	8.0544048	0.0000054
Site	0.7617154	2	1.3687527	0.2614455
Sex	1.0283229	2	1.8478290	0.1655195
log10_read_depth	0.0619474	1	0.2226309	0.6385756
Year	0.1565059	1	0.5624617	0.4558956
Residuals	18.6428603	67	NA	NA

```
emmeans(pl.shannon_no_outliers.lm, list(pairwise ~ Species), adjust = "tukey")
```

```
## $`emmeans of Species`
##  Species emmean    SE df lower.CL upper.CL
##  CHSE      5.42 0.346 67     4.72     6.11
##  KISU      4.49 0.187 67     4.11     4.86
##  PSCO      4.31 0.256 67     3.80     4.82
##  STCA      5.13 0.254 67     4.63     5.64
##  STOD      5.41 0.146 67     5.12     5.71
##  TRSC      4.85 0.151 67     4.55     5.15
##
## Results are averaged over the levels of: Site, Sex, Year
## Confidence level used: 0.95
##
## $`pairwise differences of Species`
##  1            estimate    SE df t.ratio p.value
##  CHSE - KISU  0.927816 0.348 67   2.663  0.0965
##  CHSE - PSCO  1.108135 0.406 67   2.731  0.0824
##  CHSE - STCA  0.280806 0.432 67   0.650  0.9866
##  CHSE - STOD  0.000439 0.337 67   0.001  1.0000
##  CHSE - TRSC  0.564278 0.349 67   1.618  0.5897
##  KISU - PSCO  0.180319 0.320 67   0.563  0.9931
##  KISU - STCA -0.647010 0.326 67  -1.984  0.3622
##  KISU - STOD -0.927377 0.178 67  -5.220  <.0001
##  KISU - TRSC -0.363538 0.199 67  -1.830  0.4538
##  PSCO - STCA -0.827330 0.400 67  -2.067  0.3169
##  PSCO - STOD -1.107696 0.298 67  -3.713  0.0054
##  PSCO - TRSC -0.543857 0.320 67  -1.697  0.5383
##  STCA - STOD -0.280367 0.299 67  -0.937  0.9354
##  STCA - TRSC  0.283472 0.281 67   1.008  0.9137
##  STOD - TRSC  0.563839 0.171 67   3.296  0.0188
##
## Results are averaged over the levels of: Site, Sex, Year
## P value adjustment: tukey method for comparing a family of 6 estimates
```

```r
emmeans(pl.shannon_no_outliers.lm, list(pairwise ~ Site), adjust = "tukey")
```

```
## $`emmeans of Site`
##  Site emmean    SE df lower.CL upper.CL
##  BP4    4.88 0.113 67     4.65     5.11
##  S1     4.82 0.220 67     4.38     5.26
##  S4     5.11 0.151 67     4.80     5.41
##
## Results are averaged over the levels of: Species, Sex, Year
## Confidence level used: 0.95
##
## $`pairwise differences of Site`
##  1        estimate    SE df t.ratio p.value
##  BP4 - S1   0.0629 0.245 67   0.257  0.9643
##  BP4 - S4  -0.2239 0.150 67  -1.489  0.3027
##  S1 - S4   -0.2869 0.247 67  -1.160  0.4808
##
## Results are averaged over the levels of: Species, Sex, Year
## P value adjustment: tukey method for comparing a family of 3 estimates
```

```r
#Testing for observed features, all samples included
#NOTE: Reordering the factors in the below model does not change the resulting p-values
#also, log10 of observed features counts does seem to improve the model fit
pl.observed.lm <- lm(log10(Observed)~Species+Site+Sex+log10_read_depth+Year,data=carapace)

summary(pl.observed.lm)
```

```
##
## Call:
## lm(formula = log10(Observed) ~ Species + Site + Sex + log10_read_depth +
##     Year, data = carapace)
##
## Residuals:
##      Min       1Q   Median       3Q      Max
## -0.35539 -0.06169  0.01695  0.08110  0.24636
##
## Coefficients:
##                   Estimate Std. Error t value Pr(>|t|)
## (Intercept)      -46.083243  77.621471  -0.594 0.554579
## SpeciesKISU       -0.255946   0.084080  -3.044 0.003258 **
## SpeciesPSCO       -0.192683   0.089995  -2.141 0.035657 *
## SpeciesSTCA       -0.144487   0.102437  -1.411 0.162696
## SpeciesSTOD       -0.004525   0.081334  -0.056 0.955789
## SpeciesTRSC       -0.090480   0.082900  -1.091 0.278722
## SiteS1             0.074621   0.055636   1.341 0.184066
## SiteS4             0.076112   0.035947   2.117 0.037686 *
## SexJ               0.044246   0.065629   0.674 0.502349
## SexM               0.029751   0.032359   0.919 0.360954
## log10_read_depth   0.486528   0.118360   4.111 0.000103 ***
## Year               0.022970   0.038513   0.596 0.552759
## ---
## Signif. codes:  0 '***' 0.001 '**' 0.01 '*' 0.05 '.' 0.1 ' ' 1
```

```
##
## Residual standard error: 0.1276 on 72 degrees of freedom
## Multiple R-squared:  0.5327, Adjusted R-squared:  0.4613
## F-statistic: 7.462 on 11 and 72 DF,  p-value: 2.232e-08
```

```r
confint(pl.observed.lm)
```

```
##                          2.5 %       97.5 %
## (Intercept)      -2.008188e+02 108.65233100
## SpeciesKISU      -4.235562e-01  -0.08833659
## SpeciesPSCO      -3.720847e-01  -0.01328077
## SpeciesSTCA      -3.486911e-01   0.05971609
## SpeciesSTOD      -1.666620e-01   0.15761245
## SpeciesTRSC      -2.557381e-01   0.07477852
## SiteS1           -3.628869e-02   0.18552980
## SiteS4            4.452815e-03   0.14777048
## SexJ             -8.658228e-02   0.17507483
## SexM             -3.475498e-02   0.09425616
## log10_read_depth  2.505811e-01   0.72247419
## Year             -5.380445e-02   0.09974543
```

```r
check_model(pl.observed.lm)
```

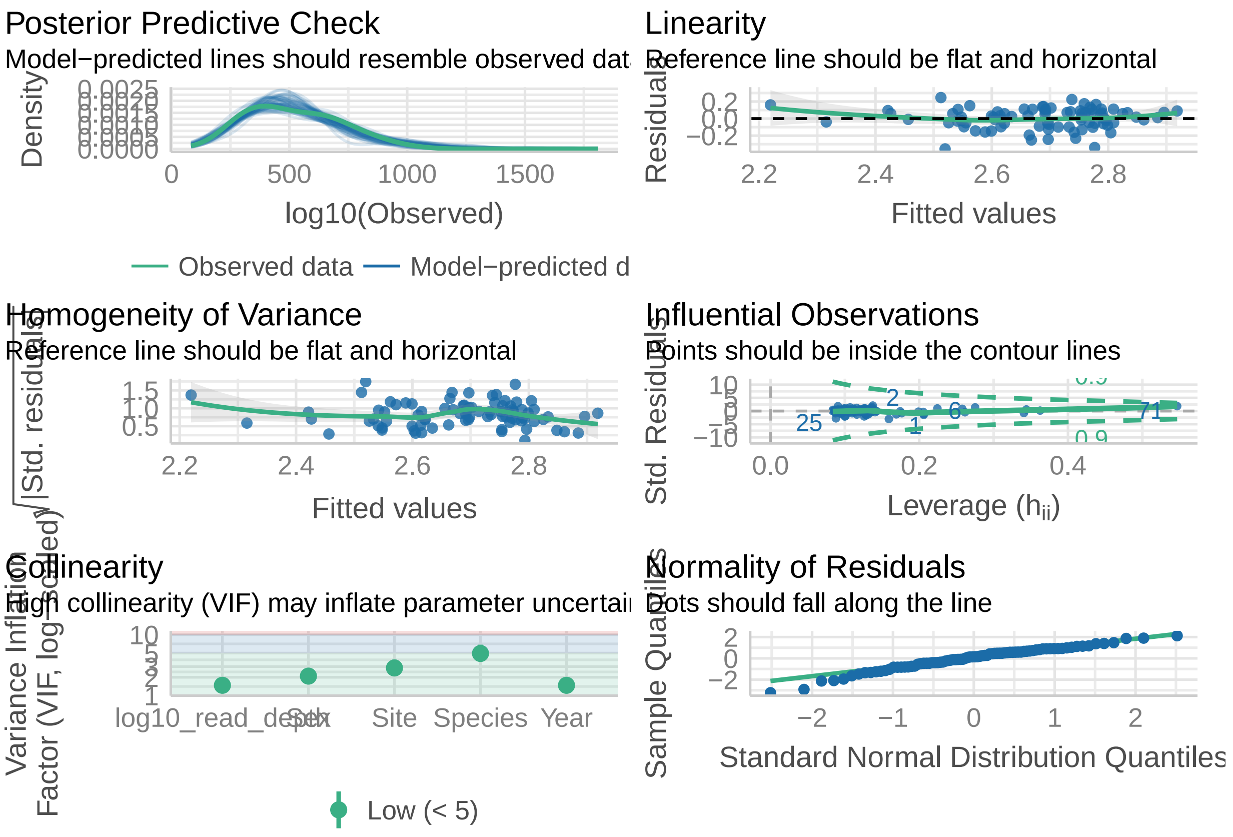


```r
plot(pl.observed.lm, which = 1)
```

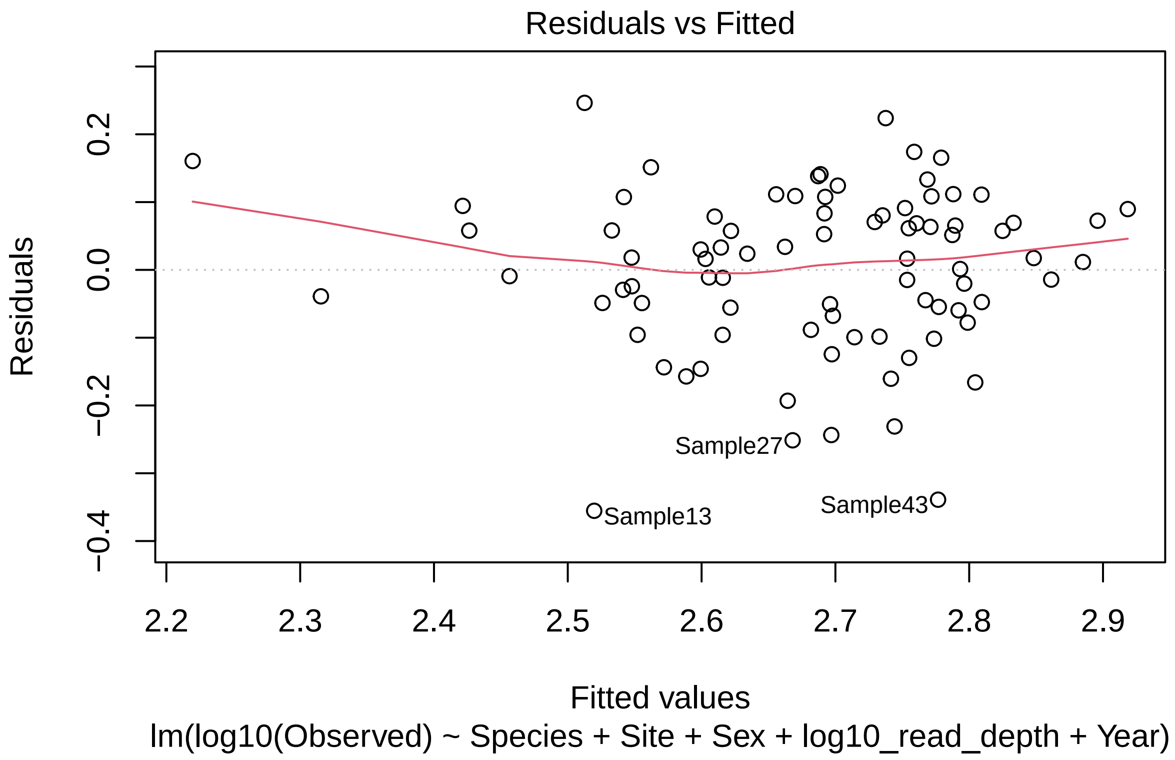


```
plot(pl.observed.lm, which = 2)
```

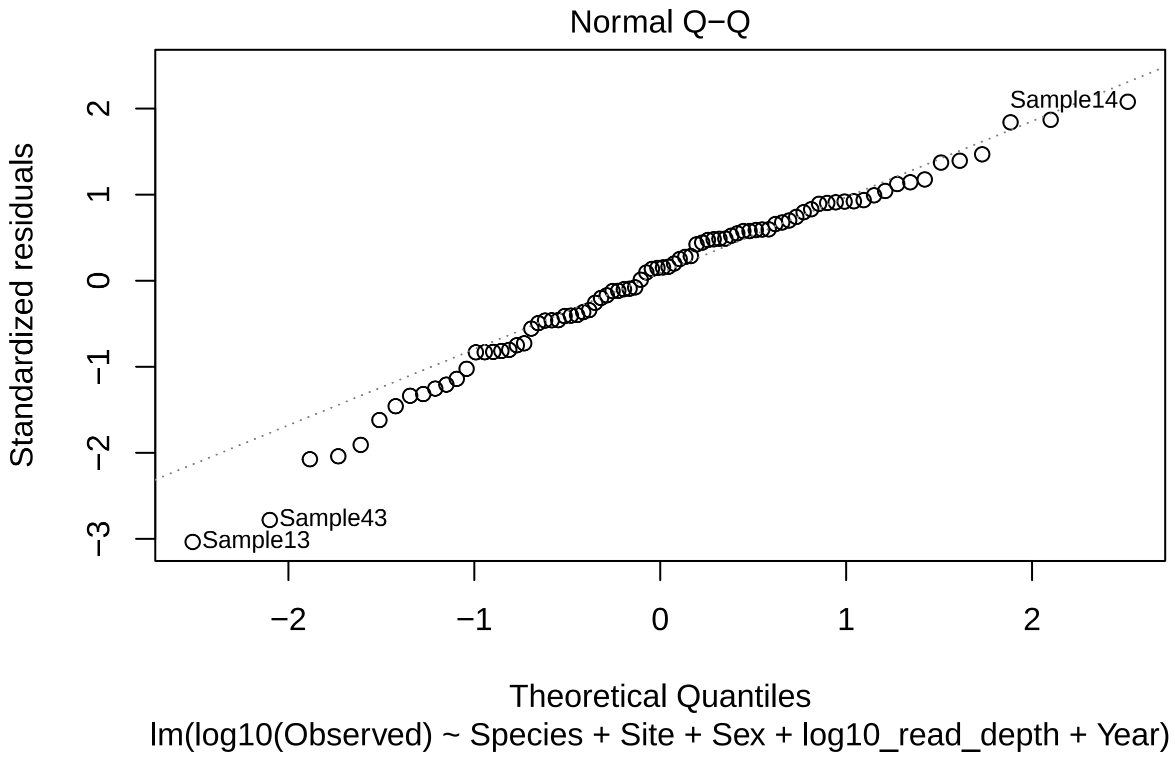


```
ols_plot_resid_stand(pl.observed.lm)
```

Table 3: Result: ANOVA comparing observed features diversity of turtle species by different factors

	Sum Sq	Df	F value	Pr(>F)
Species	0.6780501	5	8.3260524	0.0000030
Site	0.0829284	2	2.5457781	0.0854525
Sex	0.0158466	2	0.4864657	0.6168014
log10_read_depth	0.2752055	1	16.8968018	0.0001033
Year	0.0057939	1	0.3557280	0.5527591
Residuals	1.1726952	72	NA	NA

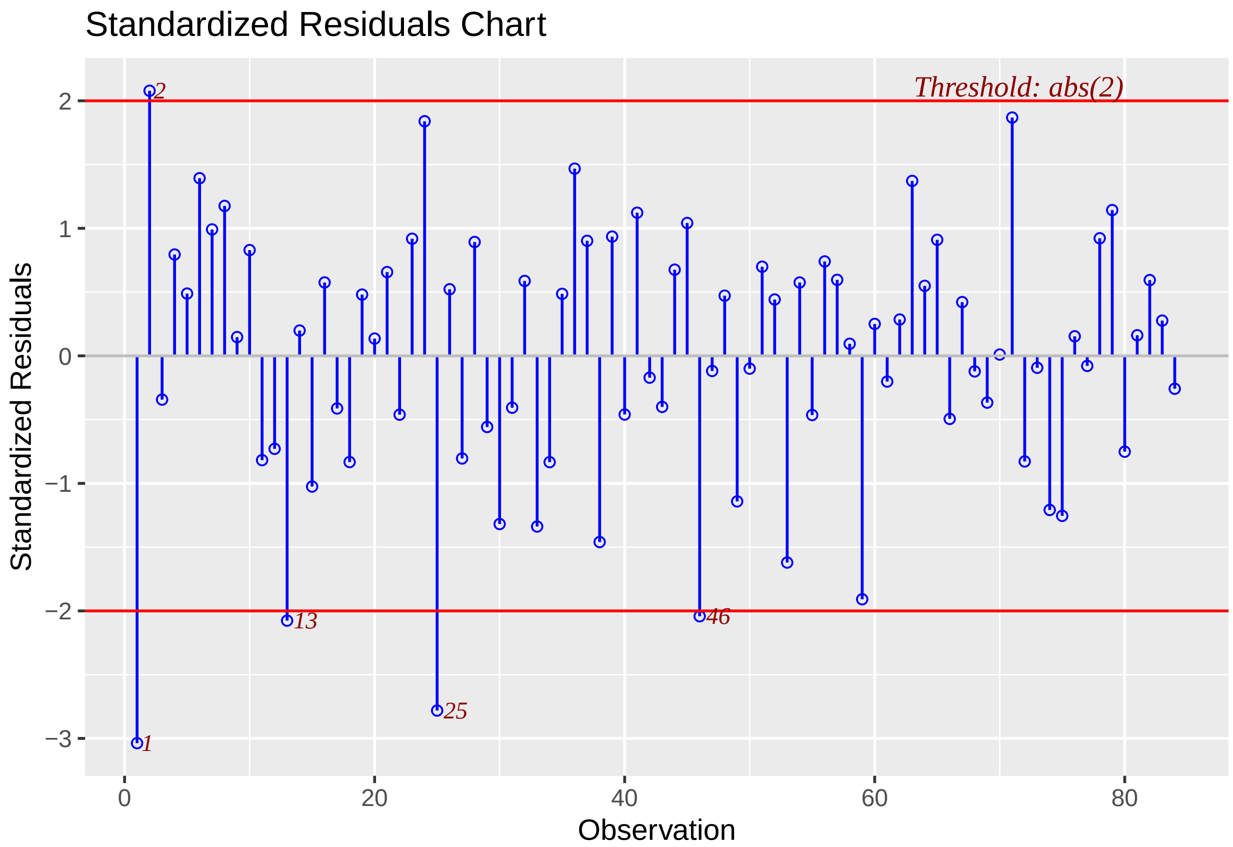


```
#ANOVA with pairwise comparisons (Tukey adjustment for pairwise comparisons):
kable.wrap(Anova(pl.observed.lm),"Result: ANOVA comparing observed features diversity of turtle species
```

```
emmeans(pl.observed.lm, list(pairwise ~ Species), adjust = "tukey")
```

```
## $`emmeans of Species`
##  Species emmean     SE df lower.CL upper.CL
##  CHSE      2.80 0.0828 72     2.64     2.97
##  KISU      2.55 0.0439 72     2.46     2.63
```

```
##  PSCO      2.61 0.0518 72     2.51     2.71
##  STCA      2.66 0.0599 72     2.54     2.78
##  STOD      2.80 0.0344 72     2.73     2.87
##  TRSC      2.71 0.0339 72     2.64     2.78
##
## Results are averaged over the levels of: Site, Sex, Year
## Results are given on the log10 (not the response) scale.
## Confidence level used: 0.95
##
## $`pairwise differences of Species`
##  1            estimate     SE df t.ratio p.value
##  CHSE - KISU  0.25595 0.0841 72   3.044  0.0369
##  CHSE - PSCO  0.19268 0.0900 72   2.141  0.2785
##  CHSE - STCA  0.14449 0.1024 72   1.411  0.7206
##  CHSE - STOD  0.00452 0.0813 72   0.056  1.0000
##  CHSE - TRSC  0.09048 0.0829 72   1.091  0.8833
##  KISU - PSCO -0.06326 0.0654 72  -0.968  0.9266
##  KISU - STCA -0.11146 0.0768 72  -1.452  0.6954
##  KISU - STOD -0.25142 0.0429 72  -5.858  <.0001
##  KISU - TRSC -0.16547 0.0469 72  -3.524  0.0093
##  PSCO - STCA -0.04820 0.0861 72  -0.560  0.9933
##  PSCO - STOD -0.18816 0.0600 72  -3.135  0.0288
##  PSCO - TRSC -0.10220 0.0635 72  -1.610  0.5948
##  STCA - STOD -0.13996 0.0705 72  -1.985  0.3608
##  STCA - TRSC -0.05401 0.0653 72  -0.827  0.9616
##  STOD - TRSC  0.08595 0.0402 72   2.138  0.2798
##
## Results are averaged over the levels of: Site, Sex, Year
## Results are given on the log10 (not the response) scale.
## P value adjustment: tukey method for comparing a family of 6 estimates
```

```
emmeans(pl.observed.lm, list(pairwise ~ Site), adjust = "tukey")
```

```
## $`emmeans of Site`
##  Site emmean     SE df lower.CL upper.CL
##  BP4    2.64 0.0270 72     2.58     2.69
##  S1     2.71 0.0509 72     2.61     2.81
##  S4     2.71 0.0364 72     2.64     2.79
##
## Results are averaged over the levels of: Species, Sex, Year
## Results are given on the log10 (not the response) scale.
## Confidence level used: 0.95
##
## $`pairwise differences of Site`
##  1         estimate     SE df t.ratio p.value
##  BP4 - S1 -0.07462 0.0556 72  -1.341  0.3773
##  BP4 - S4 -0.07611 0.0359 72  -2.117  0.0934
##  S1 - S4  -0.00149 0.0572 72  -0.026  0.9996
##
## Results are averaged over the levels of: Species, Sex, Year
## Results are given on the log10 (not the response) scale.
## P value adjustment: tukey method for comparing a family of 3 estimates
```

```r
#Rerun above analysis, but with standardized residual outliers removed
carapace_observed_no_outliers<-carapace[-c(1,2,13,25,46),]
summary(carapace_observed_no_outliers)
```

```
##   sample.ID          LibraryName          Read_depth     ProjectName
##  Length:79          Length:79          Min.   : 14153   Length:79
##  Class :character   Class :character   1st Qu.: 70724   Class :character
##  Mode  :character   Mode  :character   Median : 81949   Mode  :character
##                                        Mean   : 79933
##                                        3rd Qu.: 92895
##                                        Max.   :120102
##
##     Region           Sample_date            Year          Site
##  Length:79          Min.   :20210525   Min.   :2021   Length:79
##  Class :character   1st Qu.:20220601   1st Qu.:2022   Class :character
##  Mode  :character   Median :20220601   Median :2022   Mode  :character
##                     Mean   :20218178   Mean   :2022
##                     3rd Qu.:20220602   3rd Qu.:2022
##                     Max.   :20220602   Max.   :2022
##
##  Sample_type         Substrate           Species          Species_substrate
##  Length:79          Length:79          Length:79          Length:79
##  Class :character   Class :character   Class :character   Class :character
##  Mode  :character   Mode  :character   Mode  :character   Mode  :character
##
##
##
##
##  Species_site           Sorter          Sex            Carapace_length
##  Length:79          Min.   : 1.00   Length:79          Min.   : 49.4
##  Class :character   1st Qu.:23.00   Class :character   1st Qu.: 87.0
##  Mode  :character   Median :42.00   Mode  :character   Median :100.0
##                     Mean   :42.27                      Mean   :125.8
##                     3rd Qu.:62.00                      3rd Qu.:167.3
##                     Max.   :82.00                      Max.   :308.0
##                     NA's   :2
##  Plastron_length      Mass         Sample_number   Sex_notes
##  Min.   : 35.8   Min.   :  25.0   Min.   : 1.0   Length:79
##  1st Qu.: 65.0   1st Qu.: 110.0   1st Qu.:13.0   Class :character
##  Median : 82.6   Median : 170.0   Median :33.0   Mode  :character
##  Mean   :104.9   Mean   : 507.3   Mean   :35.0
##  3rd Qu.:152.0   3rd Qu.: 685.0   3rd Qu.:54.5
##  Max.   :239.0   Max.   :6600.0   Max.   :75.0
##
##   PCR1_date         extraction_date       Observed          Chao1
##  Length:79          Min.   :    2021   Min.   : 189.0   Min.   : 189.0
##  Class :character   1st Qu.:20220617   1st Qu.: 385.5   1st Qu.: 385.5
##  Mode  :character   Median :20220623   Median : 517.0   Median : 517.0
##                     Mean   :15357920   Mean   : 531.3   Mean   : 531.3
##                     3rd Qu.:20220624   3rd Qu.: 672.5   3rd Qu.: 672.5
##                     Max.   :20220624   Max.   :1019.0   Max.   :1019.0
##
##     se.chao1            ACE              se.ACE          Shannon
```

```
##  Min.   :0.00000   Min.   : 189.0   Min.   :3.933   Min.   :3.078  
##  1st Qu.:0.00000   1st Qu.: 385.5   1st Qu.:5.456   1st Qu.:4.563  
##  Median :0.00000   Median : 517.0   Median :6.049   Median :4.800  
##  Mean   :0.01581   Mean   : 531.3   Mean   :6.048   Mean   :4.985  
##  3rd Qu.:0.00000   3rd Qu.: 672.5   3rd Qu.:6.595   3rd Qu.:5.566  
##  Max.   :0.49969   Max.   :1019.0   Max.   :8.497   Max.   :6.345  
##                                                                    
##     Simpson         InvSimpson          Fisher       log10_read_depth
##  Min.   :0.8427   Min.   :  6.359   Min.   : 28.18   Min.   :4.151   
##  1st Qu.:0.9632   1st Qu.: 27.180   1st Qu.: 56.20   1st Qu.:4.850   
##  Median :0.9786   Median : 46.815   Median : 79.02   Median :4.914   
##  Mean   :0.9711   Mean   : 71.670   Mean   : 81.84   Mean   :4.884   
##  3rd Qu.:0.9890   3rd Qu.: 91.083   3rd Qu.:106.43   3rd Qu.:4.968   
##  Max.   :0.9970   Max.   :336.079   Max.   :163.13   Max.   :5.080   
##                                                                      
##        PD       
##  Min.   :17.15  
##  1st Qu.:33.33  
##  Median :41.44  
##  Mean   :41.12  
##  3rd Qu.:48.38  
##  Max.   :64.47  
## 
```

```
#pl.observed_no_outliers.lm <- lm(Observed~Species+Site+Plastron_length+Sex+log10_read_depth+Year,data=
pl.observed_no_outliers.lm <- lm(Observed~Species+Site+Sex+log10_read_depth+Year,data=carapace_observed_

summary(pl.observed_no_outliers.lm)
```

```
## 
## Call:
## lm(formula = Observed ~ Species + Site + Sex + log10_read_depth + 
##     Year, data = carapace_observed_no_outliers)
## 
## Residuals:
##     Min      1Q  Median      3Q     Max 
## -263.02  -96.56    9.77   83.76  324.53 
## 
## Coefficients:
##                   Estimate Std. Error t value Pr(>|t|)    
## (Intercept)      -64931.20   85233.38  -0.762 0.448850    
## SpeciesKISU        -237.81      88.05  -2.701 0.008750 ** 
## SpeciesPSCO        -181.94      93.20  -1.952 0.055115 .  
## SpeciesSTCA         -86.99     107.98  -0.806 0.423326    
## SpeciesSTOD          50.48      84.51   0.597 0.552278    
## SpeciesTRSC         -46.39      86.71  -0.535 0.594424    
## SiteS1               49.60      58.68   0.845 0.400948    
## SiteS4               71.92      38.50   1.868 0.066125 .  
## SexJ                 47.32      68.21   0.694 0.490191    
## SexM                 63.64      34.29   1.856 0.067869 .  
## log10_read_depth    513.73     124.89   4.114 0.000109 ***
## Year                 31.14      42.29   0.736 0.464109    
## ---
## Signif. codes:  0 '***' 0.001 '**' 0.01 '*' 0.05 '.' 0.1 ' ' 1
```

```
##
## Residual standard error: 132.1 on 67 degrees of freedom
## Multiple R-squared:  0.5847, Adjusted R-squared:  0.5165
## F-statistic: 8.574 on 11 and 67 DF,  p-value: 3.202e-09
```

```r
confint(pl.observed_no_outliers.lm)
```

```
##                            2.5 %        97.5 %
## (Intercept)       -2.350577e+05 105195.332904
## SpeciesKISU       -4.135678e+02    -62.062128
## SpeciesPSCO       -3.679709e+02      4.098347
## SpeciesSTCA       -3.025125e+02    128.538744
## SpeciesSTOD       -1.181981e+02    219.163320
## SpeciesTRSC       -2.194687e+02    126.687452
## SiteS1            -6.752612e+01    166.735913
## SiteS4            -4.924972e+00    148.755308
## SexJ              -8.881654e+01    183.462234
## SexM              -4.805436e+00    132.093365
## log10_read_depth   2.644596e+02    763.005376
## Year              -5.326954e+01    115.543675
```

```r
check_model(pl.observed_no_outliers.lm)
```

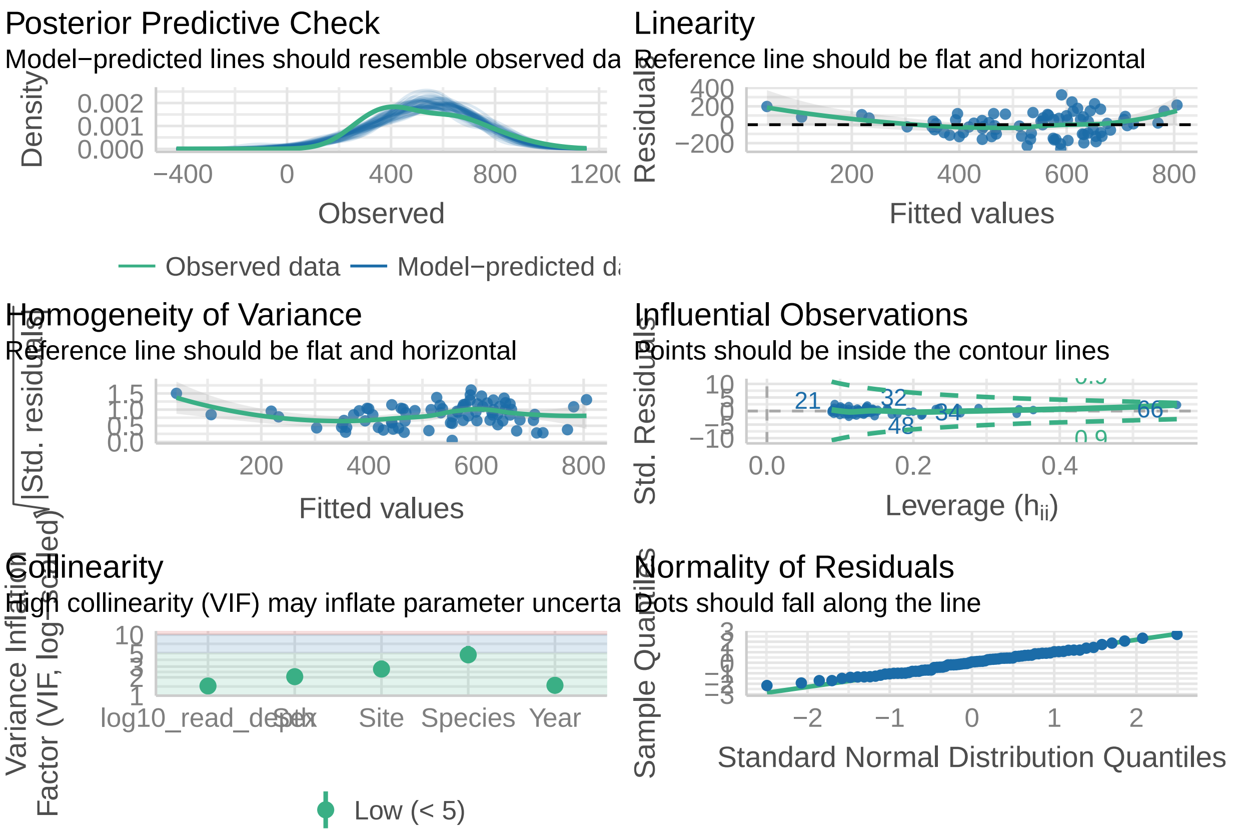


```r
plot(pl.observed_no_outliers.lm, which = 1)
```

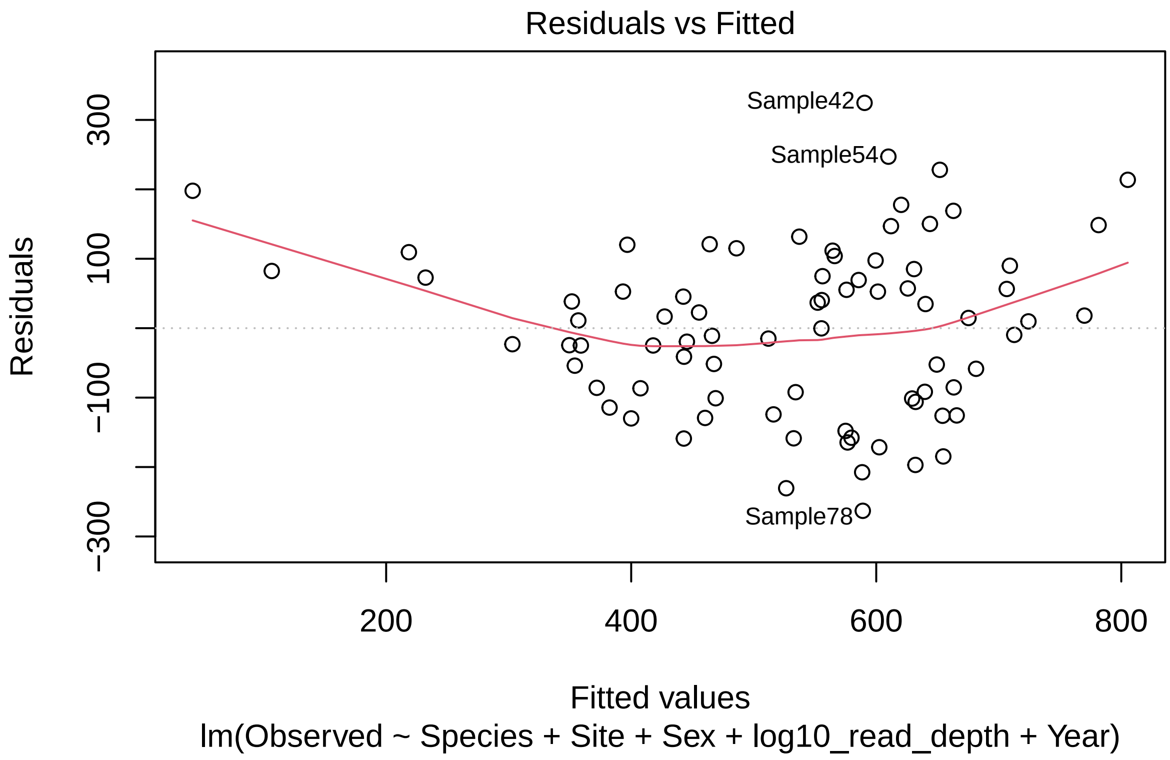


```
plot(pl.observed_no_outliers.lm, which = 2)
```

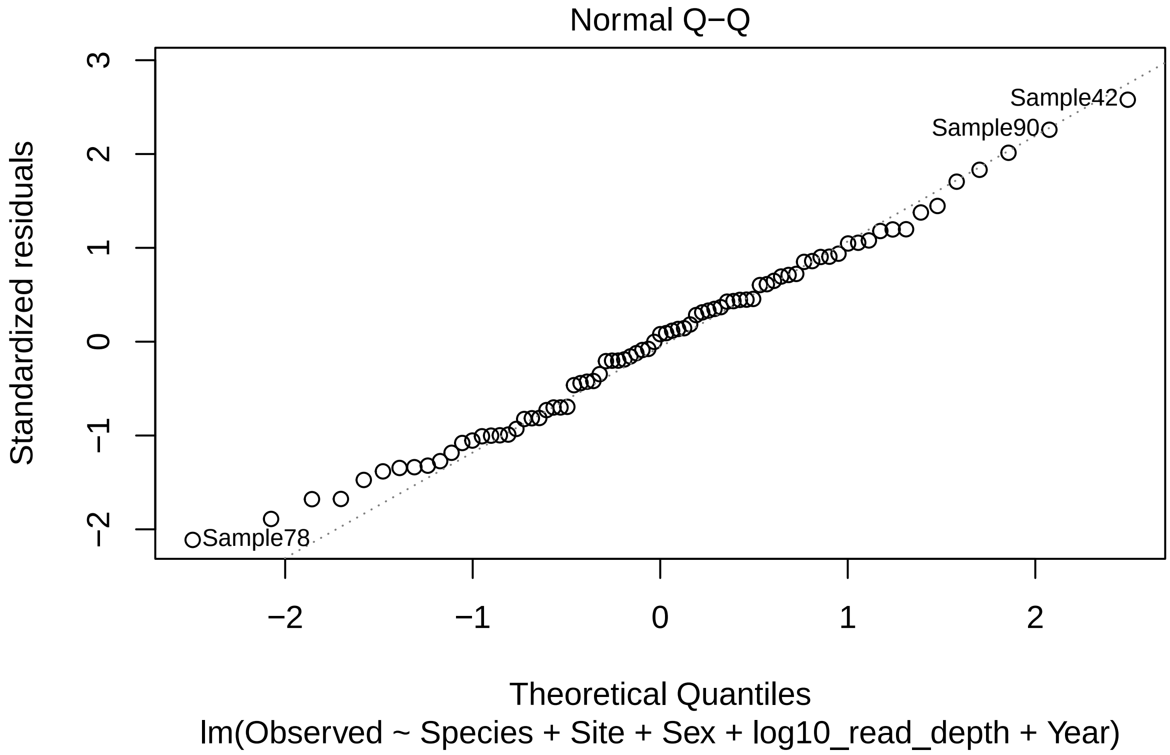


```
#ANOVA with pairwise comparisons (Tukey adjustment for pairwise comparisons):
kable.wrap(Anova(pl.observed_no_outliers.lm),"Result: ANOVA comparing observed features diversity of tu
```

Table 4: Result: ANOVA comparing observed features diversity of turtle species by different factors, standardized residual outliers removed

	Sum Sq	Df	F value	Pr(>F)
Species	777104.446	5	8.9047909	0.0000016
Site	62463.930	2	1.7894256	0.1749473
Sex	60116.012	2	1.7221640	0.1864936
log10_read_depth	295348.215	1	16.9218830	0.0001088
Year	9462.654	1	0.5421598	0.4641093
Residuals	1169392.930	67	NA	NA

```
emmeans(pl.observed_no_outliers.lm, list(pairwise ~ Species), adjust = "tukey")
```

```
## $`emmeans of Species`
##  Species emmean   SE df lower.CL upper.CL
##  CHSE       599 86.5 67      426      771
##  KISU       361 48.7 67      264      458
##  PSCO       417 54.7 67      308      526
##  STCA       512 64.6 67      383      641
##  STOD       649 36.2 67      577      722
##  TRSC       552 35.4 67      482      623
##
## Results are averaged over the levels of: Site, Sex, Year
## Confidence level used: 0.95
##
## $`pairwise differences of Species`
##  1           estimate    SE df t.ratio p.value
##  CHSE - KISU    237.8  88.1 67   2.701  0.0885
##  CHSE - PSCO    181.9  93.2 67   1.952  0.3806
##  CHSE - STCA     87.0 108.0 67   0.806  0.9655
##  CHSE - STOD    -50.5  84.5 67  -0.597  0.9909
##  CHSE - TRSC     46.4  86.7 67   0.535  0.9945
##  KISU - PSCO    -55.9  68.6 67  -0.815  0.9638
##  KISU - STCA   -150.8  83.0 67  -1.817  0.4622
##  KISU - STOD   -288.3  47.2 67  -6.108  <.0001
##  KISU - TRSC   -191.4  52.0 67  -3.683  0.0059
##  PSCO - STCA    -94.9  91.3 67  -1.040  0.9027
##  PSCO - STOD   -232.4  62.5 67  -3.716  0.0053
##  PSCO - TRSC   -135.5  66.7 67  -2.032  0.3357
##  STCA - STOD   -137.5  75.2 67  -1.827  0.4559
##  STCA - TRSC    -40.6  69.9 67  -0.581  0.9920
##  STOD - TRSC     96.9  42.3 67   2.292  0.2118
##
## Results are averaged over the levels of: Site, Sex, Year
## P value adjustment: tukey method for comparing a family of 6 estimates
```

```r
emmeans(pl.observed_no_outliers.lm, list(pairwise ~ Site), adjust = "tukey")
```

```
## $`emmeans of Site`
##  Site emmean   SE df lower.CL upper.CL
##  BP4     474 28.6 67      417      532
##  S1      524 54.1 67      416      632
##  S4      546 38.9 67      469      624
##
## Results are averaged over the levels of: Species, Sex, Year
## Confidence level used: 0.95
##
## $`pairwise differences of Site`
##  1        estimate   SE df t.ratio p.value
##  BP4 - S1    -49.6 58.7 67  -0.845  0.6763
##  BP4 - S4    -71.9 38.5 67  -1.868  0.1559
##  S1 - S4     -22.3 59.7 67  -0.373  0.9261
##
## Results are averaged over the levels of: Species, Sex, Year
## P value adjustment: tukey method for comparing a family of 3 estimates
```

```r
#Testing for Faith's Phylogenetic Diversity, all samples included
#NOTE: Reordering the factors in the below model does not change the resulting p-values
pl.PD.lm <- lm(PD~Species+Site+Sex+log10_read_depth+Year,data=carapace)

summary(pl.PD.lm)
```

```
##
## Call:
## lm(formula = PD ~ Species + Site + Sex + log10_read_depth + Year,
##     data = carapace)
##
## Residuals:
##      Min       1Q   Median       3Q      Max
## -17.0974  -4.5401   0.3689   5.3953  13.1209
##
## Coefficients:
##                   Estimate Std. Error t value Pr(>|t|)
## (Intercept)      -6954.216   4446.453  -1.564   0.1222
## SpeciesKISU        -12.178      4.816  -2.528   0.0137 *
## SpeciesPSCO         -5.896      5.155  -1.144   0.2565
## SpeciesSTCA         -3.317      5.868  -0.565   0.5736
## SpeciesSTOD          2.591      4.659   0.556   0.5799
## SpeciesTRSC         -0.948      4.749  -0.200   0.8423
## SiteS1              -1.032      3.187  -0.324   0.7471
## SiteS4               4.586      2.059   2.227   0.0291 *
## SexJ                 2.550      3.760   0.678   0.4998
## SexM                 2.515      1.854   1.357   0.1791
## log10_read_depth    29.121      6.780   4.295 5.37e-05 ***
## Year                 3.389      2.206   1.536   0.1289
## ---
## Signif. codes:  0 '***' 0.001 '**' 0.01 '*' 0.05 '.' 0.1 ' ' 1
##
```

```
## Residual standard error: 7.311 on 72 degrees of freedom
## Multiple R-squared:  0.5473, Adjusted R-squared:  0.4781
## F-statistic: 7.913 on 11 and 72 DF,  p-value: 8.01e-09
```

```
confint(pl.PD.lm)
```

```
##                          2.5 %      97.5 %
## (Intercept)      -1.581806e+04 1909.627399
## SpeciesKISU      -2.177916e+01   -2.576507
## SpeciesPSCO      -1.617315e+01    4.380505
## SpeciesSTCA      -1.501461e+01    8.380512
## SpeciesSTOD      -6.697281e+00   11.878397
## SpeciesTRSC      -1.041458e+01    8.518671
## SiteS1           -7.385038e+00    5.321570
## SiteS4            4.808257e-01    8.690607
## SexJ             -4.944446e+00   10.044269
## SexM             -1.180077e+00    6.210172
## log10_read_depth  1.560489e+01   42.636717
## Year             -1.008693e+00    7.787229
```

```
check_model(pl.PD.lm)
```

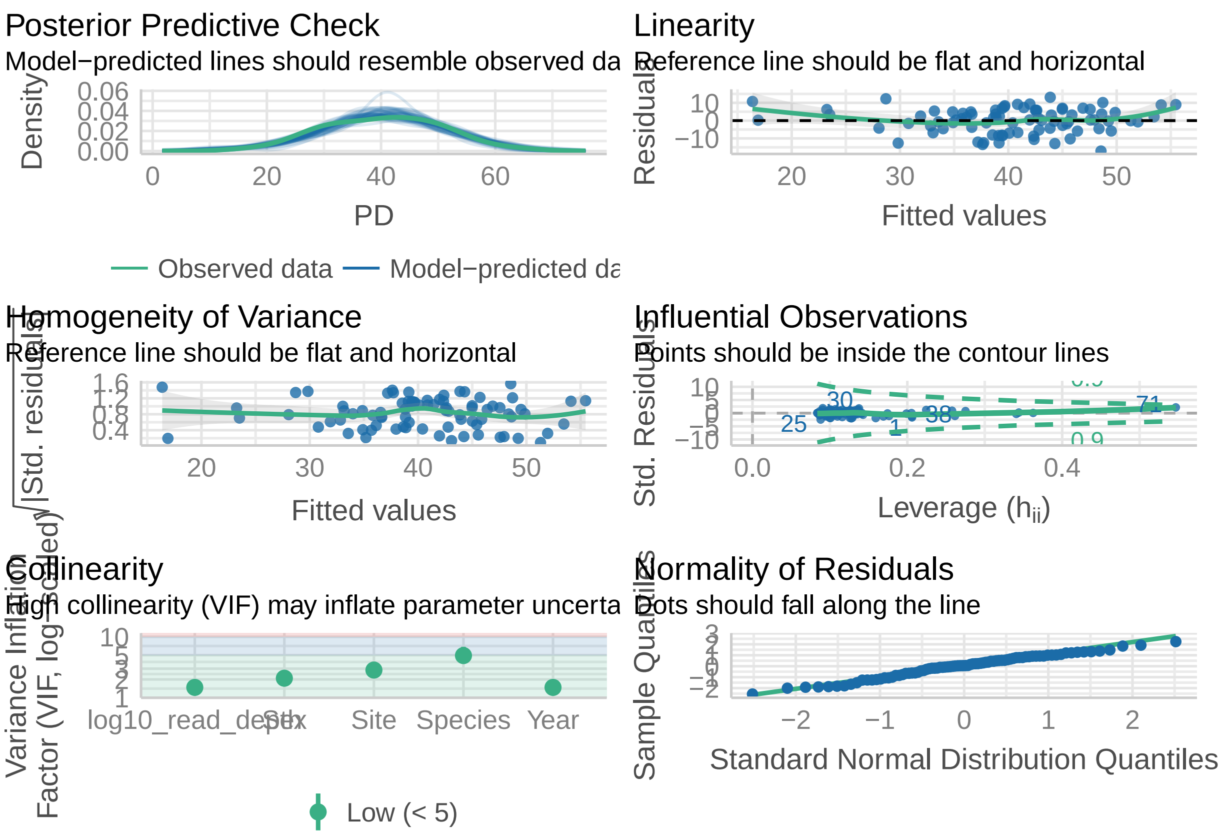


```
plot(pl.PD.lm, which = 1)
```

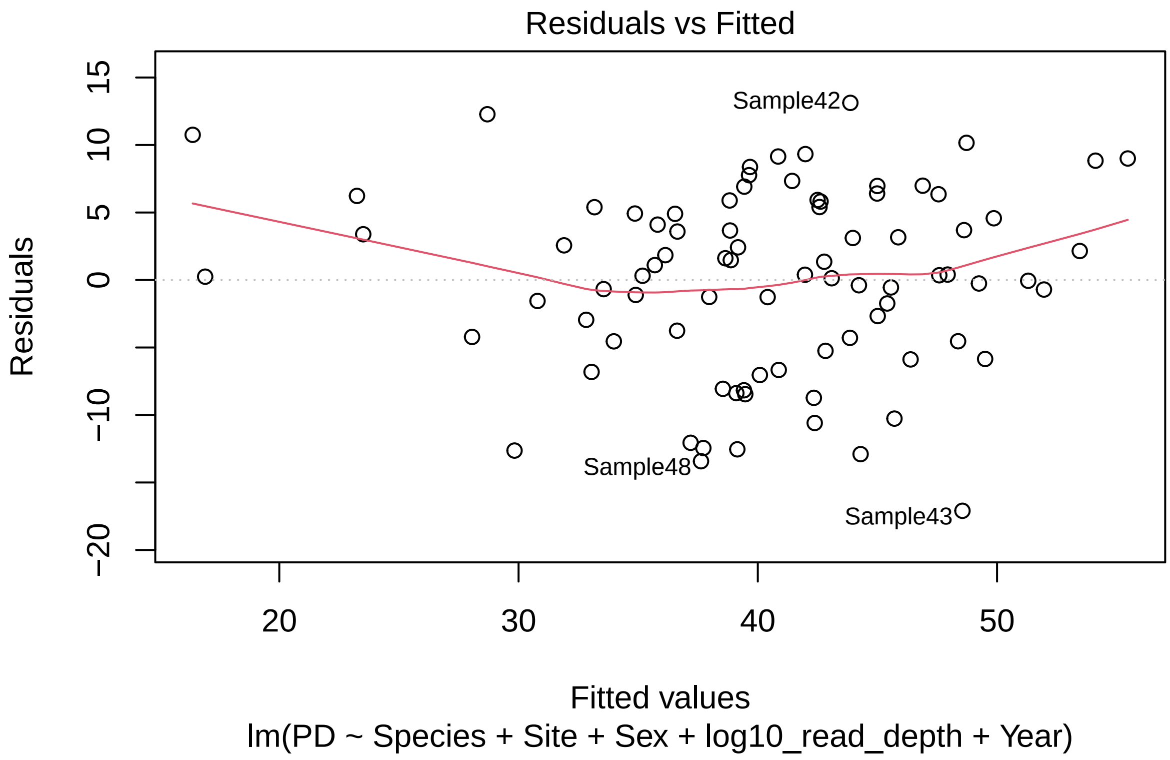


```r
plot(pl.PD.lm, which = 2)
```

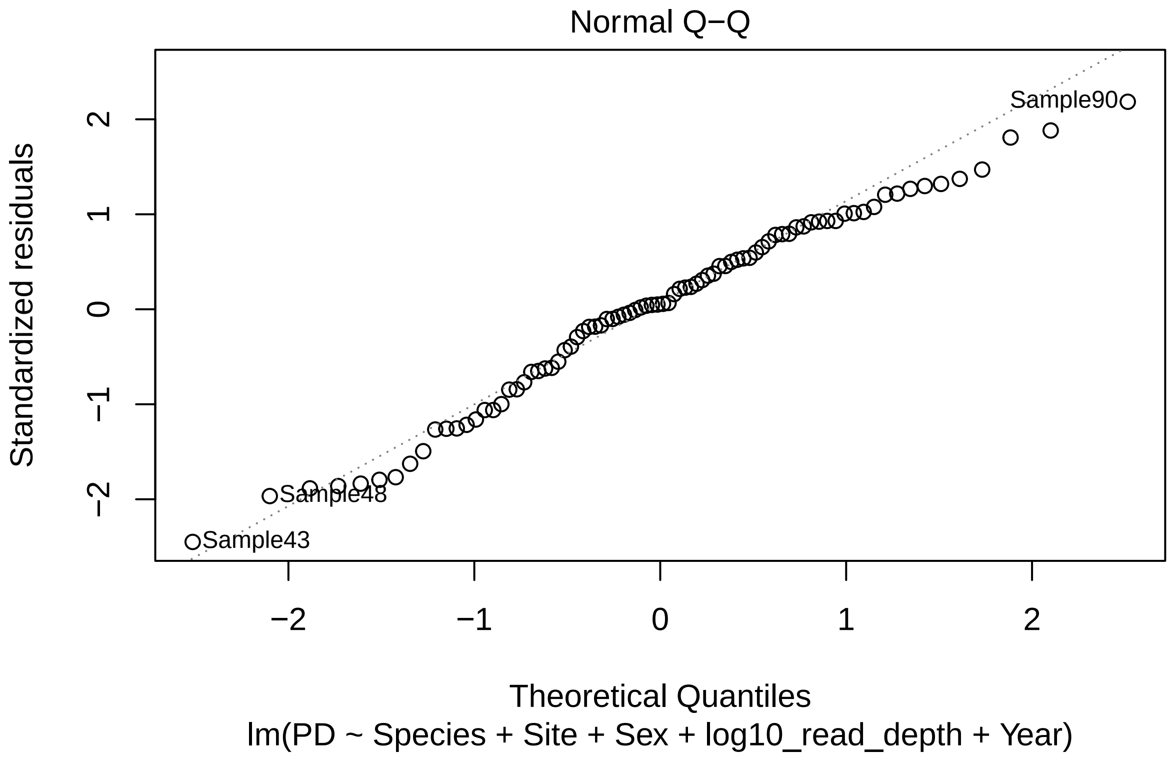


```r
ols_plot_resid_stand(pl.PD.lm)#gives same outliers as resid_level, and more than resid_student, so will
```

Table 5: Result: Multiple regression comparing PD of turtle species by different factors

	Sum Sq	Df	F value	Pr(>F)
Species	2111.6525	5	7.9019823	0.0000056
Site	317.0308	2	2.9658899	0.0578422
Sex	100.2415	2	0.9377806	0.3962245
log10_read_depth	985.9360	1	18.4472800	0.0000537
Year	126.1369	1	2.3600746	0.1288593
Residuals	3848.1225	72	NA	NA

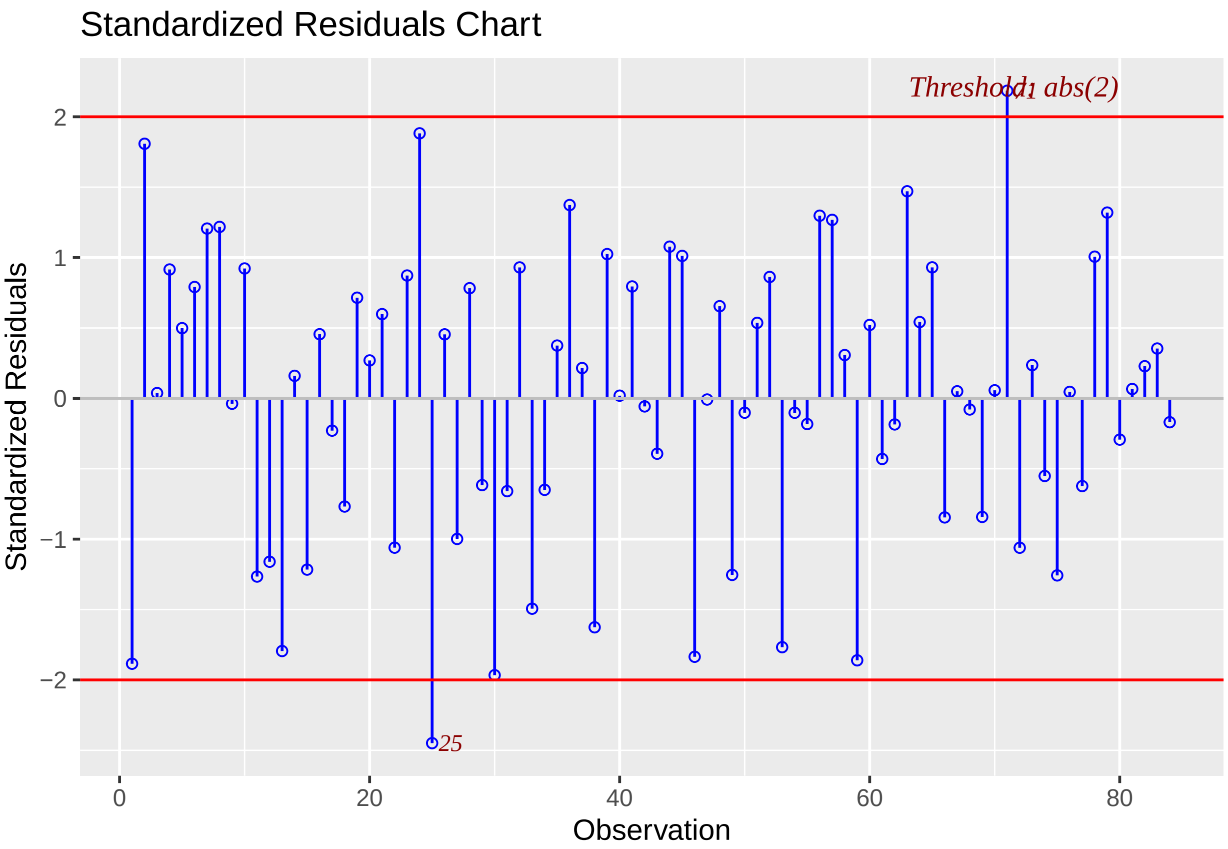


```
#ANOVA with pairwise comparisons (Tukey adjustment for pairwise comparisons):
kable.wrap(Anova(pl.PD.lm),"Result: Multiple regression comparing PD of turtle species by different fac
```

```
emmeans(pl.PD.lm, list(pairwise ~ Species), adjust = "tukey")
```

```
## $`emmeans of Species`
##  Species emmean   SE df lower.CL upper.CL
##  CHSE      42.3 4.75 72     32.8     51.7
##  KISU      30.1 2.51 72     25.1     35.1
```

```
##  PSCO      36.4 2.96 72     30.5     42.3
##  STCA      38.9 3.43 72     32.1     45.8
##  STOD      44.9 1.97 72     40.9     48.8
##  TRSC      41.3 1.94 72     37.4     45.2
##
## Results are averaged over the levels of: Site, Sex, Year
## Confidence level used: 0.95
##
## $`pairwise differences of Species`
##  1           estimate   SE df t.ratio p.value
##  CHSE - KISU   12.178 4.82 72   2.528  0.1296
##  CHSE - PSCO    5.896 5.16 72   1.144  0.8613
##  CHSE - STCA    3.317 5.87 72   0.565  0.9929
##  CHSE - STOD   -2.591 4.66 72  -0.556  0.9935
##  CHSE - TRSC    0.948 4.75 72   0.200  1.0000
##  KISU - PSCO   -6.282 3.75 72  -1.677  0.5511
##  KISU - STCA   -8.861 4.40 72  -2.015  0.3443
##  KISU - STOD  -14.768 2.46 72  -6.007  <.0001
##  KISU - TRSC  -11.230 2.69 72  -4.176  0.0011
##  PSCO - STCA   -2.579 4.93 72  -0.523  0.9951
##  PSCO - STOD   -8.487 3.44 72  -2.469  0.1473
##  PSCO - TRSC   -4.948 3.64 72  -1.361  0.7499
##  STCA - STOD   -5.908 4.04 72  -1.463  0.6886
##  STCA - TRSC   -2.369 3.74 72  -0.633  0.9881
##  STOD - TRSC    3.539 2.30 72   1.537  0.6421
##
## Results are averaged over the levels of: Site, Sex, Year
## P value adjustment: tukey method for comparing a family of 6 estimates
```

```
emmeans(pl.PD.lm, list(pairwise ~ Site), adjust = "tukey")
```

```
## $`emmeans of Site`
##  Site emmean   SE df lower.CL upper.CL
##  BP4    37.8 1.55 72     34.7     40.9
##  S1     36.8 2.91 72     30.9     42.6
##  S4     42.4 2.08 72     38.2     46.5
##
## Results are averaged over the levels of: Species, Sex, Year
## Confidence level used: 0.95
##
## $`pairwise differences of Site`
##  1        estimate   SE df t.ratio p.value
##  BP4 - S1     1.03 3.19 72   0.324  0.9439
##  BP4 - S4    -4.59 2.06 72  -2.227  0.0734
##  S1 - S4     -5.62 3.28 72  -1.715  0.2065
##
## Results are averaged over the levels of: Species, Sex, Year
## P value adjustment: tukey method for comparing a family of 3 estimates
```

```
#Rerun above analysis, but with standardized residual outliers removed
carapace_PD_no_outliers<-carapace[-c(25,71),]
summary(carapace_PD_no_outliers)
```

```
##    sample.ID          LibraryName          Read_depth     ProjectName
```

```
##  Length:82          Length:82          Min.   : 26901   Length:82
##  Class :character   Class :character   1st Qu.: 70606   Class :character
##  Mode  :character   Mode  :character   Median : 81136   Mode  :character
##                                        Mean   : 80198
##                                        3rd Qu.: 92026
##                                        Max.   :120102
##
##     Region           Sample_date            Year          Site
##  Length:82          Min.   :20210525   Min.   :2021   Length:82
##  Class :character   1st Qu.:20210526   1st Qu.:2021   Class :character
##  Mode  :character   Median :20220601   Median :2022   Mode  :character
##                     Mean   :20217898   Mean   :2022
##                     3rd Qu.:20220602   3rd Qu.:2022
##                     Max.   :20220602   Max.   :2022
##
##  Sample_type         Substrate           Species          Species_substrate
##  Length:82          Length:82          Length:82          Length:82
##  Class :character   Class :character   Class :character   Class :character
##  Mode  :character   Mode  :character   Mode  :character   Mode  :character
##
##
##
##
##  Species_site           Sorter          Sex            Carapace_length
##  Length:82          Min.   : 1.00   Length:82          Min.   : 49.4
##  Class :character   1st Qu.:20.50   Class :character   1st Qu.: 87.0
##  Mode  :character   Median :41.00   Mode  :character   Median : 98.0
##                     Mean   :41.11                      Mean   :124.7
##                     3rd Qu.:60.50                      3rd Qu.:163.8
##                     Max.   :82.00                      Max.   :308.0
##                     NA's   :3
##  Plastron_length      Mass        Sample_number    Sex_notes
##  Min.   : 35.8   Min.   :  25.0   Min.   : 1.00   Length:82
##  1st Qu.: 65.0   1st Qu.: 110.0   1st Qu.:13.00   Class :character
##  Median : 83.3   Median : 170.0   Median :31.50   Mode  :character
##  Mean   :103.9   Mean   : 494.9   Mean   :33.78
##  3rd Qu.:151.8   3rd Qu.: 662.5   3rd Qu.:52.75
##  Max.   :239.0   Max.   :6600.0   Max.   :75.00
##
##   PCR1_date         extraction_date       Observed          Chao1
##  Length:82          Min.   :    2021   Min.   : 146.0   Min.   : 146.0
##  Class :character   1st Qu.:    2021   1st Qu.: 375.8   1st Qu.: 375.8
##  Mode  :character   Median :20220620   Median : 507.0   Median : 507.0
##                     Mean   :14796119   Mean   : 524.3   Mean   : 524.3
##                     3rd Qu.:20220623   3rd Qu.: 669.8   3rd Qu.: 669.8
##                     Max.   :20220624   Max.   :1019.0   Max.   :1019.0
##
##     se.chao1           ACE            se.ACE         Shannon
##  Min.   :0.00000   Min.   : 146.0   Min.   :3.669   Min.   :3.078
##  1st Qu.:0.00000   1st Qu.: 375.8   1st Qu.:5.309   1st Qu.:4.557
##  Median :0.00000   Median : 507.0   Median :6.048   Median :4.781
##  Mean   :0.01523   Mean   : 524.3   Mean   :5.997   Mean   :4.960
##  3rd Qu.:0.00000   3rd Qu.: 669.8   3rd Qu.:6.582   3rd Qu.:5.533
##  Max.   :0.49969   Max.   :1019.0   Max.   :8.497   Max.   :6.345
```

```
##
##     Simpson          InvSimpson          Fisher       log10_read_depth
##  Min.   :0.8427   Min.   :  6.359   Min.   : 19.49   Min.   :4.430
##  1st Qu.:0.9623   1st Qu.: 26.560   1st Qu.: 54.59   1st Qu.:4.849
##  Median :0.9783   Median : 46.160   Median : 78.74   Median :4.909
##  Mean   :0.9708   Mean   : 70.501   Mean   : 80.58   Mean   :4.890
##  3rd Qu.:0.9891   3rd Qu.: 91.832   3rd Qu.:105.94   3rd Qu.:4.964
##  Max.   :0.9970   Max.   :336.079   Max.   :163.13   Max.   :5.080
##
##        PD
##  Min.   :17.15
##  1st Qu.:32.92
##  Median :40.74
##  Mean   :40.62
##  3rd Qu.:48.26
##  Max.   :64.47
##
```

```
#pl.PD_no_outliers.lm <- lm(PD~Species+Site+Plastron_length+Sex+log10_read_depth+Year,data=carapace_PD_
pl.PD_no_outliers.lm <- lm(PD~Species+Site+Sex+log10_read_depth+Year,data=carapace_PD_no_outliers)

summary(pl.PD_no_outliers.lm)
```

```
##
## Call:
## lm(formula = PD ~ Species + Site + Sex + log10_read_depth + Year,
##     data = carapace_PD_no_outliers)
##
## Residuals:
##     Min       1Q  Median      3Q     Max
## -14.456  -4.766   1.008   4.859  12.933
##
## Coefficients:
##                   Estimate Std. Error t value Pr(>|t|)
## (Intercept)      -3539.9236  4467.8340  -0.792  0.43086
## SpeciesKISU        -12.4361     4.5065  -2.760  0.00738 **
## SpeciesPSCO         -7.9712     4.9091  -1.624  0.10892
## SpeciesSTCA         -2.6892     5.4982  -0.489  0.62630
## SpeciesSTOD          2.4913     4.3608   0.571  0.56963
## SpeciesTRSC         -1.3168     4.5197  -0.291  0.77164
## SiteS1              -0.9054     3.0598  -0.296  0.76819
## SiteS4               4.7980     1.9547   2.455  0.01659 *
## SexJ                 2.8798     3.5135   0.820  0.41521
## SexM                 1.7495     1.7915   0.977  0.33214
## log10_read_depth    44.2011     8.6327   5.120 2.57e-06 ***
## Year                 1.6644     2.2210   0.749  0.45613
## ---
## Signif. codes:  0 '***' 0.001 '**' 0.01 '*' 0.05 '.' 0.1 ' ' 1
##
## Residual standard error: 6.821 on 70 degrees of freedom
## Multiple R-squared:  0.6048, Adjusted R-squared:  0.5427
## F-statistic: 9.737 on 11 and 70 DF,  p-value: 2.087e-10
```

```r
confint(pl.PD_no_outliers.lm)
```

```
##                            2.5 %       97.5 %
## (Intercept)        -1.245074e+04 5370.890374
## SpeciesKISU        -2.142394e+01   -3.448233
## SpeciesPSCO        -1.776205e+01    1.819618
## SpeciesSTCA        -1.365493e+01    8.276607
## SpeciesSTOD        -6.205995e+00   11.188535
## SpeciesTRSC        -1.033099e+01    7.697362
## SiteS1             -7.008046e+00    5.197285
## SiteS4              8.994152e-01    8.696578
## SexJ               -4.127691e+00    9.887313
## SexM               -1.823442e+00    5.322453
## log10_read_depth    2.698381e+01   61.418475
## Year               -2.765221e+00    6.093962
```

```r
check_model(pl.PD_no_outliers.lm)
```

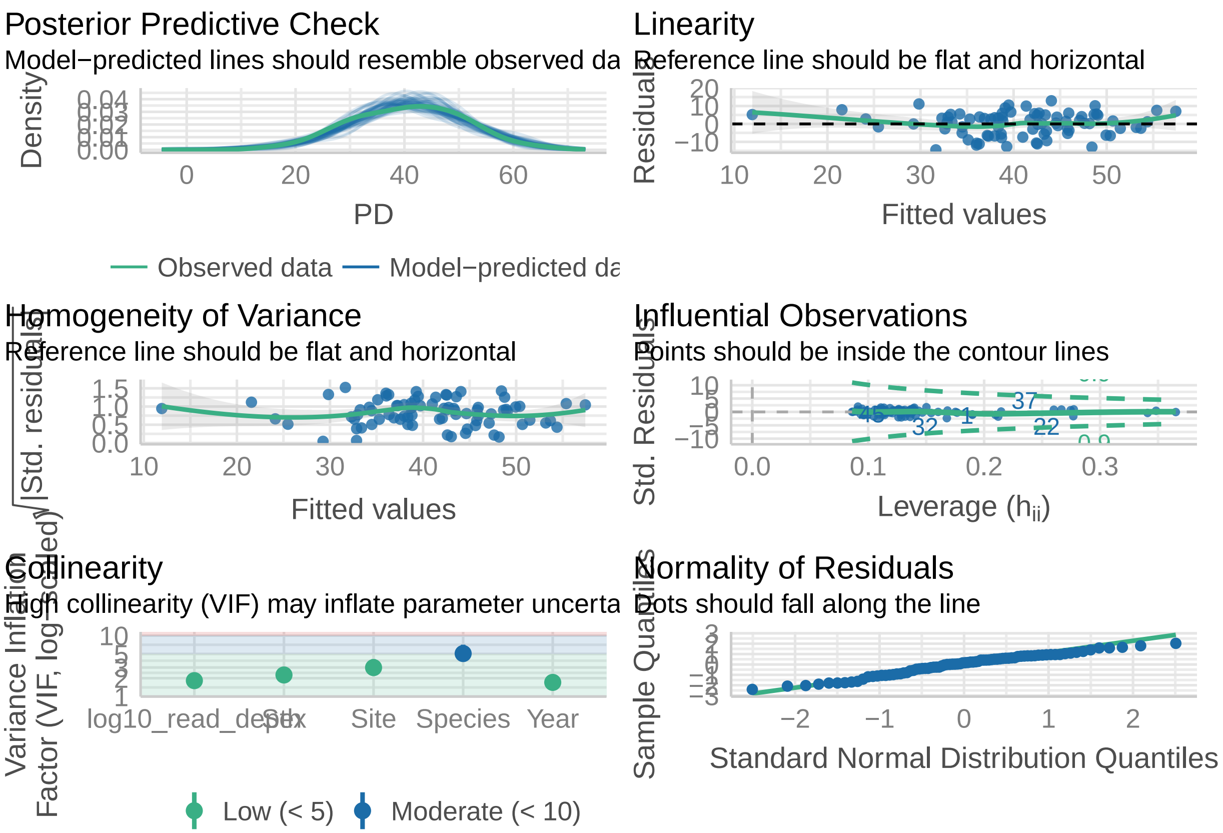


```r
plot(pl.PD_no_outliers.lm, which = 1)
```

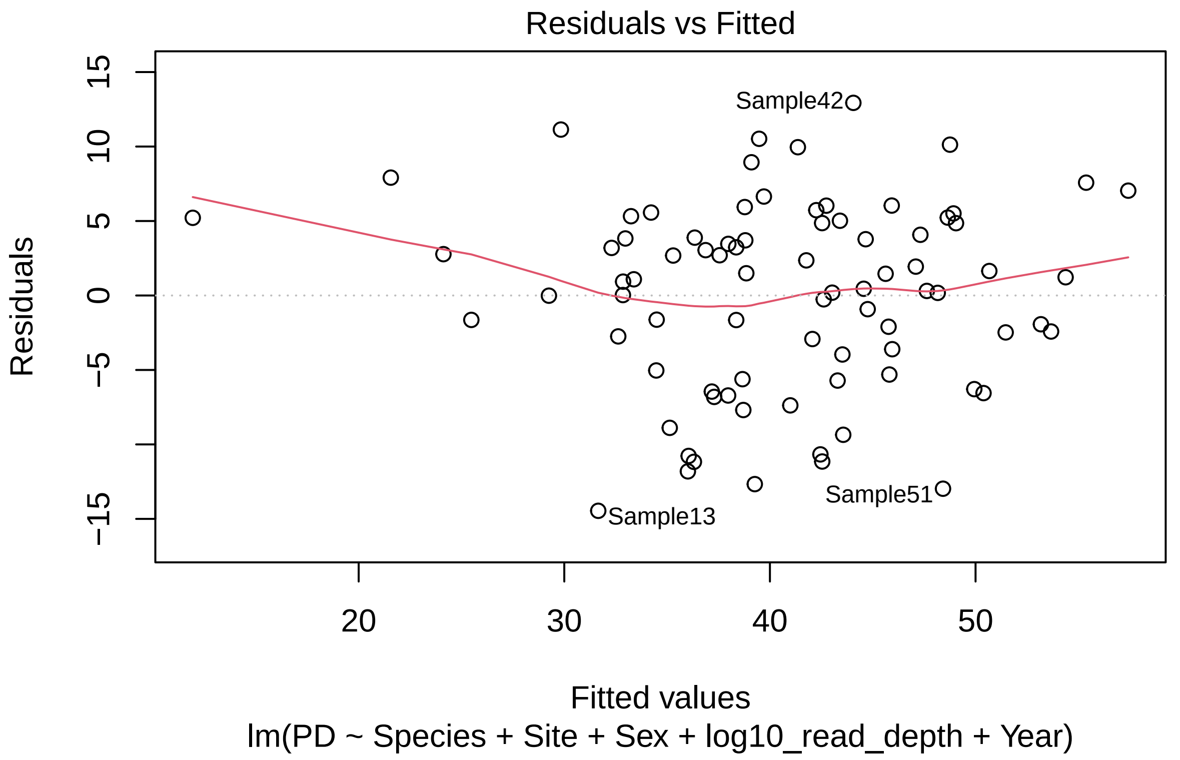


```
plot(pl.PD_no_outliers.lm, which = 2)
```

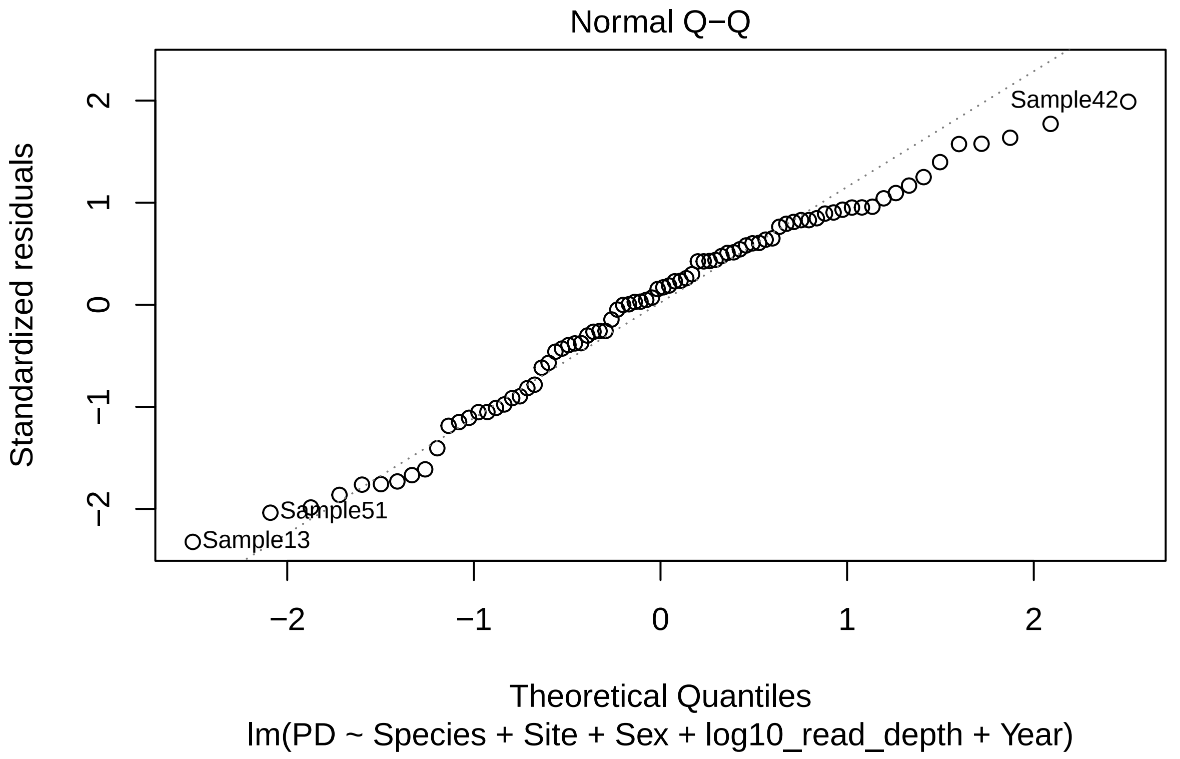


```
#ANOVA with pairwise comparisons (Tukey adjustment for pairwise comparisons):
kable.wrap(Anova(pl.PD_no_outliers.lm),"Result: Multiple regression comparing PD of turtle species by di
```

Table 6: Result: Multiple regression comparing PD of turtle species by different factors, standardized residual outliers removed

	Sum Sq	Df	F value	Pr(>F)
Species	2224.60619	5	9.5627422	0.0000006
Site	337.91212	2	3.6313916	0.0315842
Sex	56.71217	2	0.6094606	0.5465032
log10_read_depth	1219.76664	1	26.2165815	0.0000026
Year	26.12847	1	0.5615821	0.4561346
Residuals	3256.85730	70	NA	NA

```
emmeans(pl.PD_no_outliers.lm, list(pairwise ~ Species), adjust = "tukey")
```

```
## $`emmeans of Species`
##  Species emmean   SE df lower.CL upper.CL
##  CHSE      43.6 4.52 70     34.6     52.6
##  KISU      31.1 2.41 70     26.3     35.9
##  PSCO      35.6 2.79 70     30.0     41.2
##  STCA      40.9 3.26 70     34.4     47.4
##  STOD      46.1 1.93 70     42.2     49.9
##  TRSC      42.3 1.82 70     38.6     45.9
##
## Results are averaged over the levels of: Site, Sex, Year
## Confidence level used: 0.95
##
## $`pairwise differences of Species`
##  1            estimate   SE df t.ratio p.value
##  CHSE - KISU     12.44 4.51 70   2.760  0.0764
##  CHSE - PSCO      7.97 4.91 70   1.624  0.5859
##  CHSE - STCA      2.69 5.50 70   0.489  0.9964
##  CHSE - STOD     -2.49 4.36 70  -0.571  0.9926
##  CHSE - TRSC      1.32 4.52 70   0.291  0.9997
##  KISU - PSCO     -4.46 3.56 70  -1.254  0.8086
##  KISU - STCA     -9.75 4.11 70  -2.370  0.1813
##  KISU - STOD    -14.93 2.29 70  -6.506  <.0001
##  KISU - TRSC    -11.12 2.57 70  -4.326  0.0007
##  PSCO - STCA     -5.28 4.68 70  -1.130  0.8673
##  PSCO - STOD    -10.46 3.29 70  -3.185  0.0253
##  PSCO - TRSC     -6.65 3.43 70  -1.941  0.3864
##  STCA - STOD     -5.18 3.78 70  -1.372  0.7434
##  STCA - TRSC     -1.37 3.53 70  -0.389  0.9988
##  STOD - TRSC      3.81 2.22 70   1.718  0.5251
##
## Results are averaged over the levels of: Site, Sex, Year
## P value adjustment: tukey method for comparing a family of 6 estimates
```

```r
emmeans(pl.PD_no_outliers.lm, list(pairwise ~ Site), adjust = "tukey")
```

```
## $`emmeans of Site`
##  Site emmean   SE df lower.CL upper.CL
##  BP4    38.6 1.46 70     35.7     41.5
##  S1     37.7 2.83 70     32.1     43.4
##  S4     43.4 2.00 70     39.4     47.4
##
## Results are averaged over the levels of: Species, Sex, Year
## Confidence level used: 0.95
##
## $`pairwise differences of Site`
##  1        estimate   SE df t.ratio p.value
##  BP4 - S1    0.905 3.06 70   0.296  0.9529
##  BP4 - S4   -4.798 1.95 70  -2.455  0.0433
##  S1 - S4    -5.703 3.08 70  -1.853  0.1601
##
## Results are averaged over the levels of: Species, Sex, Year
## P value adjustment: tukey method for comparing a family of 3 estimates
```

Alpha diversity boxplots for carapace samples

The below plots show diversity distributions for carapace samples with standardized residual outliers removed, for Shannon diversity, observed species, and Faith's Phylogenetic Diversity. Plots are first by turtle species, then by site.

```r
ggplot(carapace_observed_no_outliers,aes(x=Species,y=Shannon))+
  geom_boxplot()+
  theme_classic()+
  theme(axis.text.x = element_text(angle = 90,hjust = 0.5),axis.title.x=element_blank())+
  ggtitle("16S Shannon diversity carapace samples (patterns by species) (standardized residual outliers
```

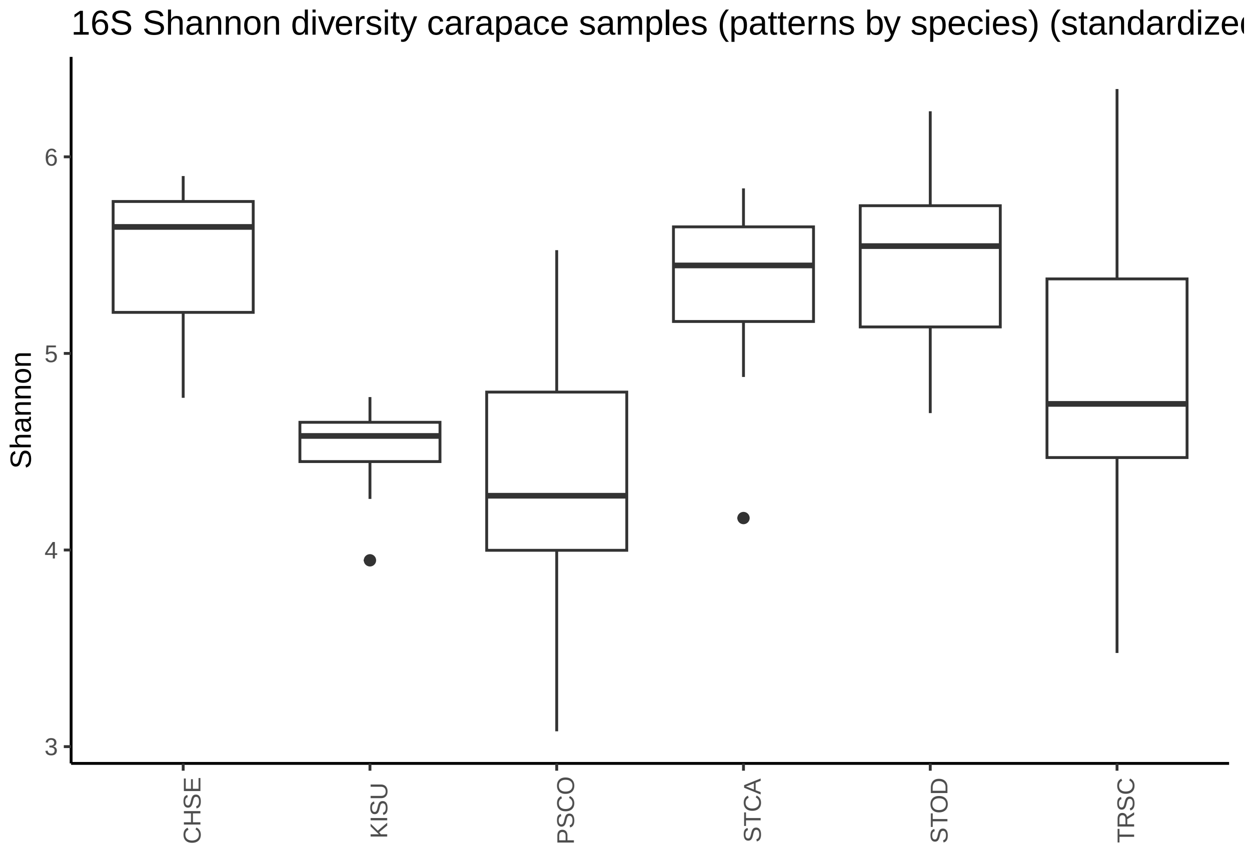


```
ggplot(carapace_shannon_no_outliers,aes(x=Species,y=Observed))+
  geom_boxplot()+
  theme_classic()+
  theme(axis.text.x = element_text(angle = 90,hjust = 0.5),axis.title.x=element_blank())+
  ggtitle("16S Observed features carapace samples (patterns by species) (standardized residual outliers
```

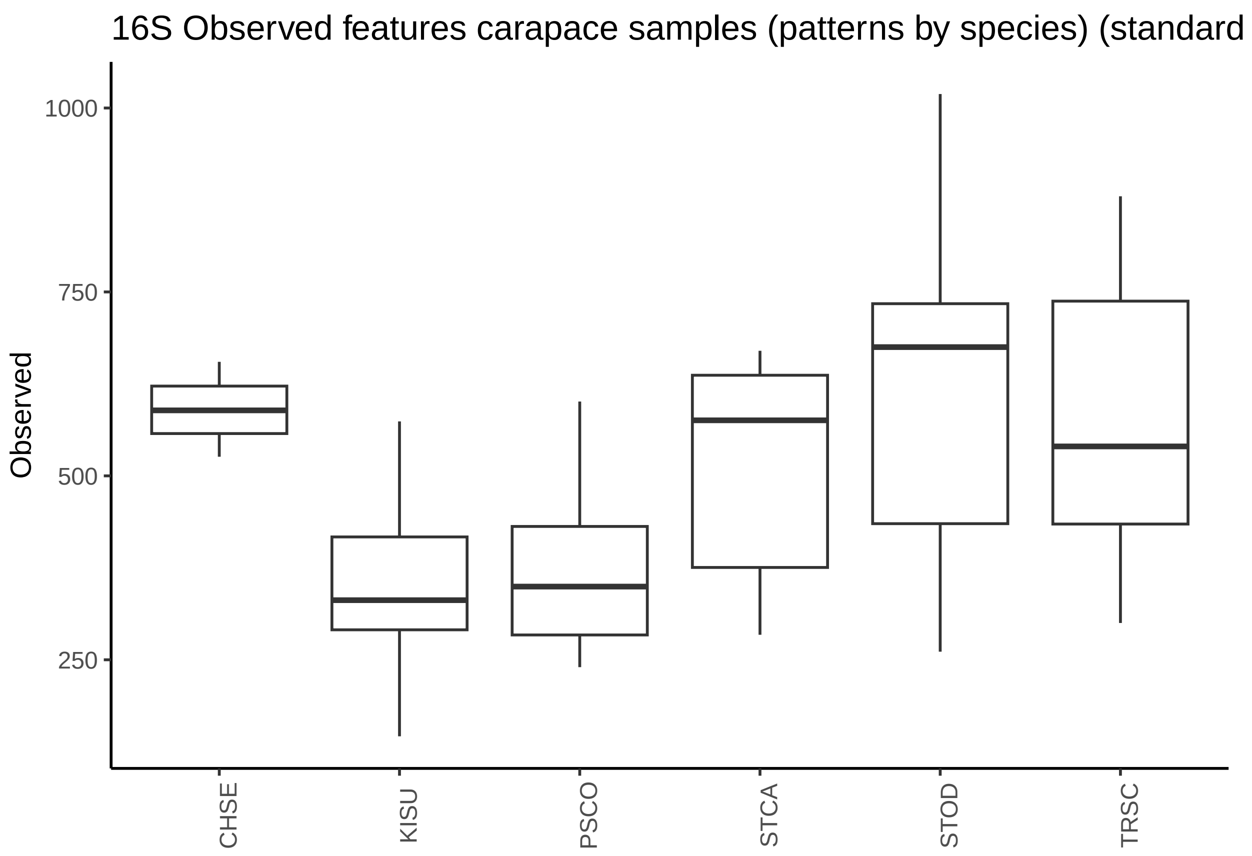


```
ggplot(carapace_PD_no_outliers,aes(x=Species,y=PD))+
  geom_boxplot()+
  theme_classic()+
  theme(axis.text.x = element_text(angle = 90,hjust = 0.5),axis.title.x=element_blank())+
  ggtitle("16S Faith's Phylogenetic Diversity carapace samples (patterns by species) (standardized resi
```

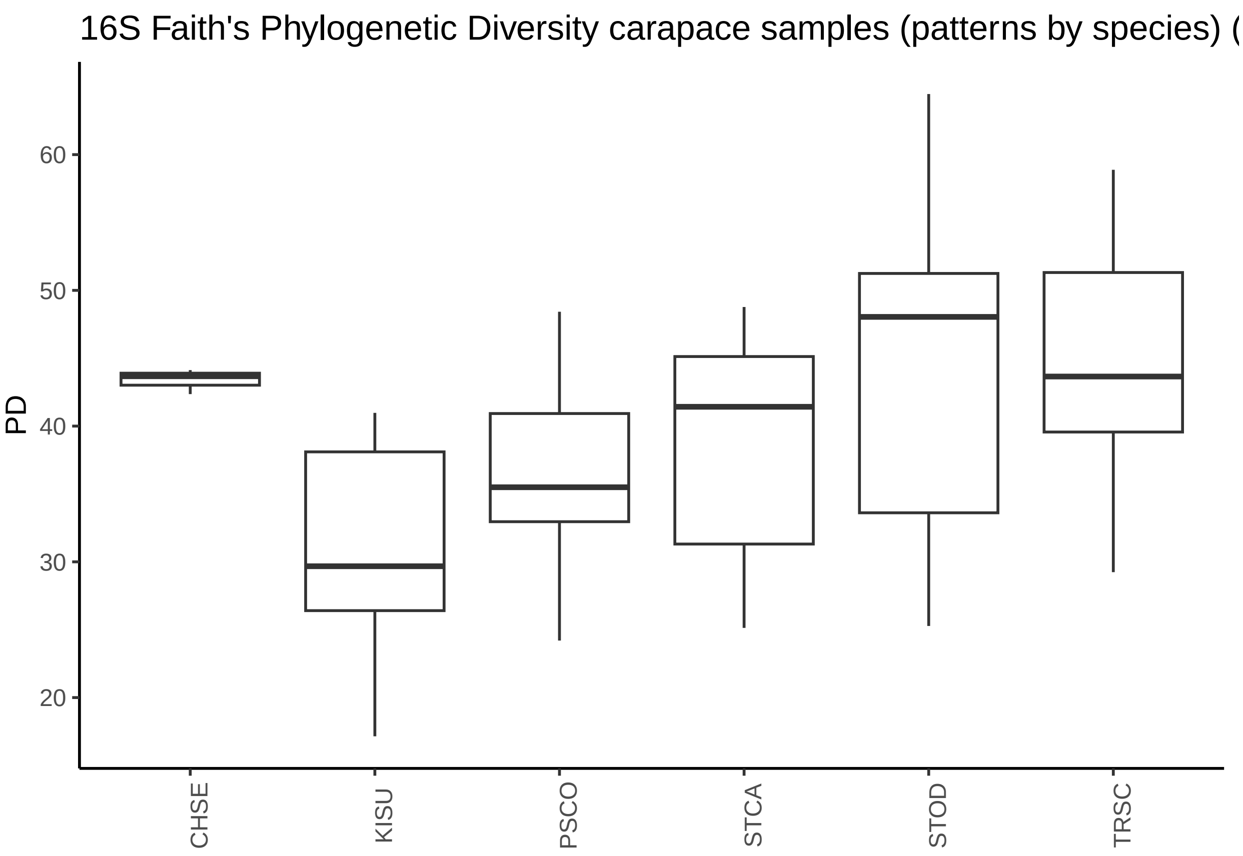


```
ggplot(carapace_observed_no_outliers,aes(x=Site,y=Shannon))+
  geom_boxplot()+
  theme_classic()+
  theme(axis.text.x = element_text(angle = 90,hjust = 0.5),axis.title.x=element_blank())+
  ggtitle("16S Shannon diversity carapace samples (patterns by site) (standardized residual outliers rem
```

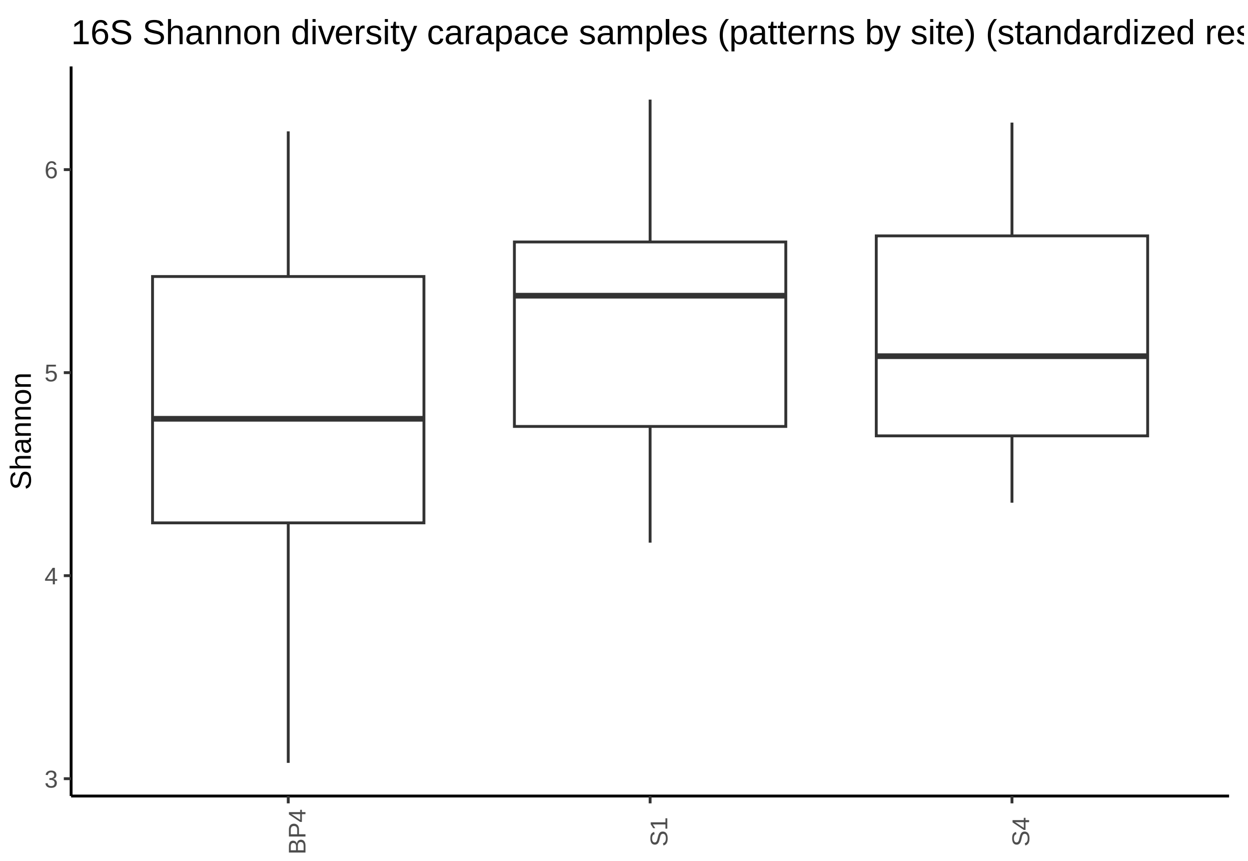


```
ggplot(carapace_shannon_no_outliers,aes(x=Site,y=Observed))+
  geom_boxplot()+
  theme_classic()+
  theme(axis.text.x = element_text(angle = 90,hjust = 0.5),axis.title.x=element_blank())+
  ggtitle("16S Observed features carapace samples (patterns by site) (standardized residual outliers re
```

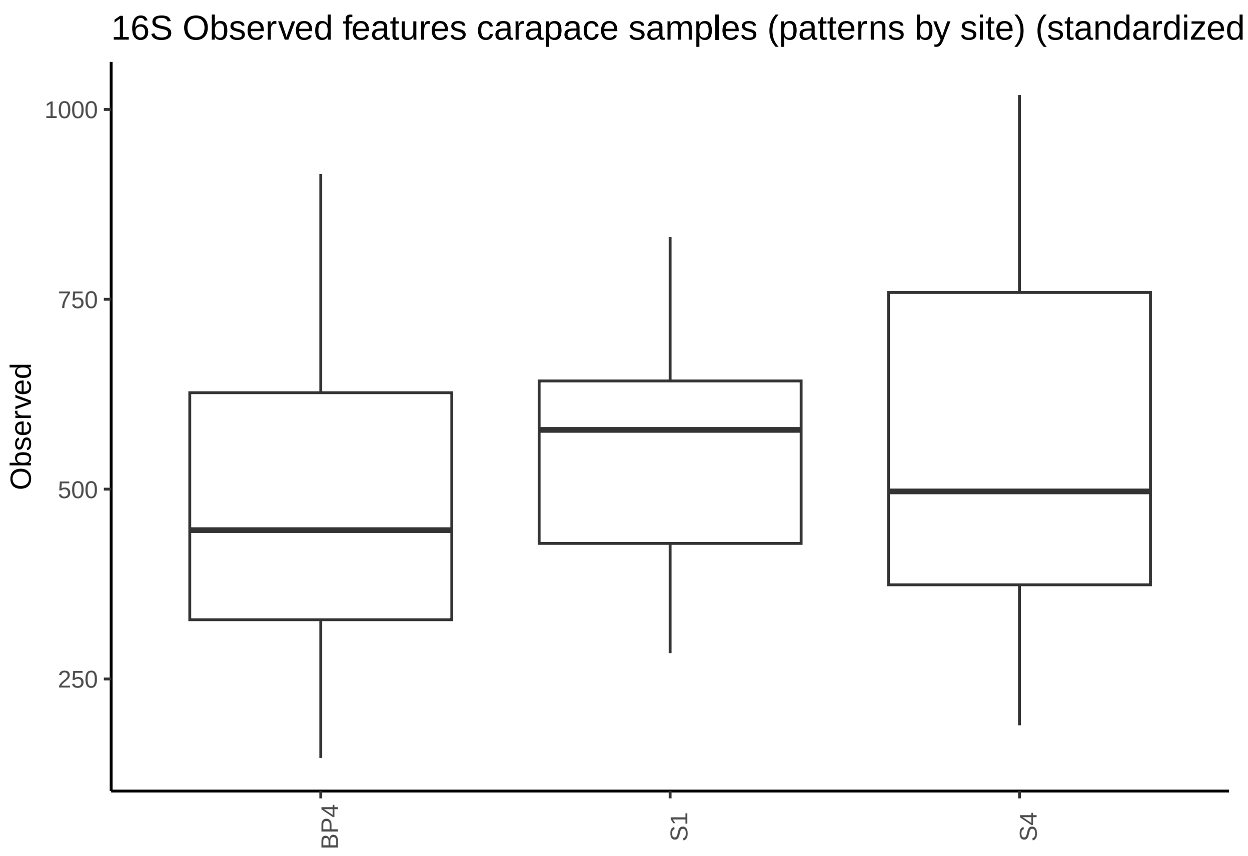


```
ggplot(carapace_PD_no_outliers,aes(x=Site,y=PD))+
  geom_boxplot()+
  theme_classic()+
  theme(axis.text.x = element_text(angle = 90,hjust = 0.5),axis.title.x=element_blank())+
  ggtitle("16S Faith's Phylogenetic Diversity carapace samples (patterns by site) (standardized residual
```

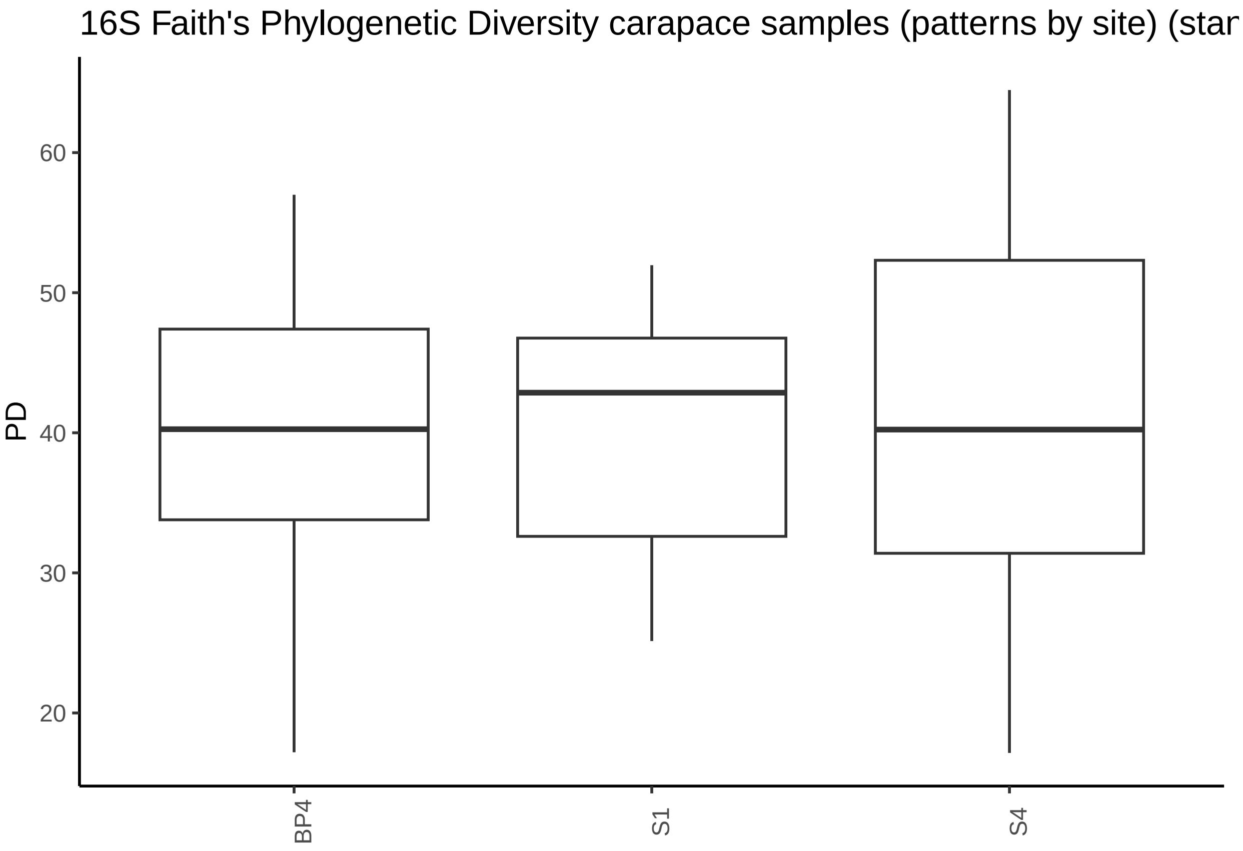

Check for effects of plastron length within each turtle species

In the below section, effect of plastron length is checked within each species, since plastron lengths are expected to differ between species and have moderate to high collinearity with turtle species in multiple regression analysis. CHSE is not included in this analysis, as the regression will not work with only three samples.

```
#Check Shannon diversity first
#check for effects in KISU
pl.shannon.KISU <- lm(Shannon~Plastron_length+Site+Sex+log10_read_depth+Year,data=KISU)

summary(pl.shannon.KISU)
```

```
##
## Call:
## lm(formula = Shannon ~ Plastron_length + Site + Sex + log10_read_depth +
##     Year, data = KISU)
##
## Residuals:
##      Min       1Q   Median       3Q      Max
## -0.49008 -0.25577 -0.06856  0.16371  0.67812
##
## Coefficients:
##                    Estimate Std. Error t value Pr(>|t|)
## (Intercept)      -79.277679 646.121762  -0.123    0.905
```

```
## Plastron_length   -0.002233   0.021396  -0.104    0.919
## SiteS4             0.393722   0.360658   1.092    0.307
## SexM              -0.262427   0.356977  -0.735    0.483
## log10_read_depth   0.216595   1.058648   0.205    0.843
## Year               0.040981   0.320670   0.128    0.901
##
## Residual standard error: 0.4305 on 8 degrees of freedom
## Multiple R-squared:  0.2956, Adjusted R-squared:  -0.1446
## F-statistic: 0.6715 on 5 and 8 DF,  p-value: 0.6567
```

```
confint(pl.shannon.KISU)
```

```
##                          2.5 %       97.5 %
## (Intercept)      -1.569237e+03 1.410682e+03
## Plastron_length  -5.157158e-02 4.710615e-02
## SiteS4           -4.379557e-01 1.225401e+00
## SexM             -1.085618e+00 5.607632e-01
## log10_read_depth -2.224651e+00 2.657841e+00
## Year             -6.984857e-01 7.804471e-01
```

```
check_model(pl.shannon.KISU)
```

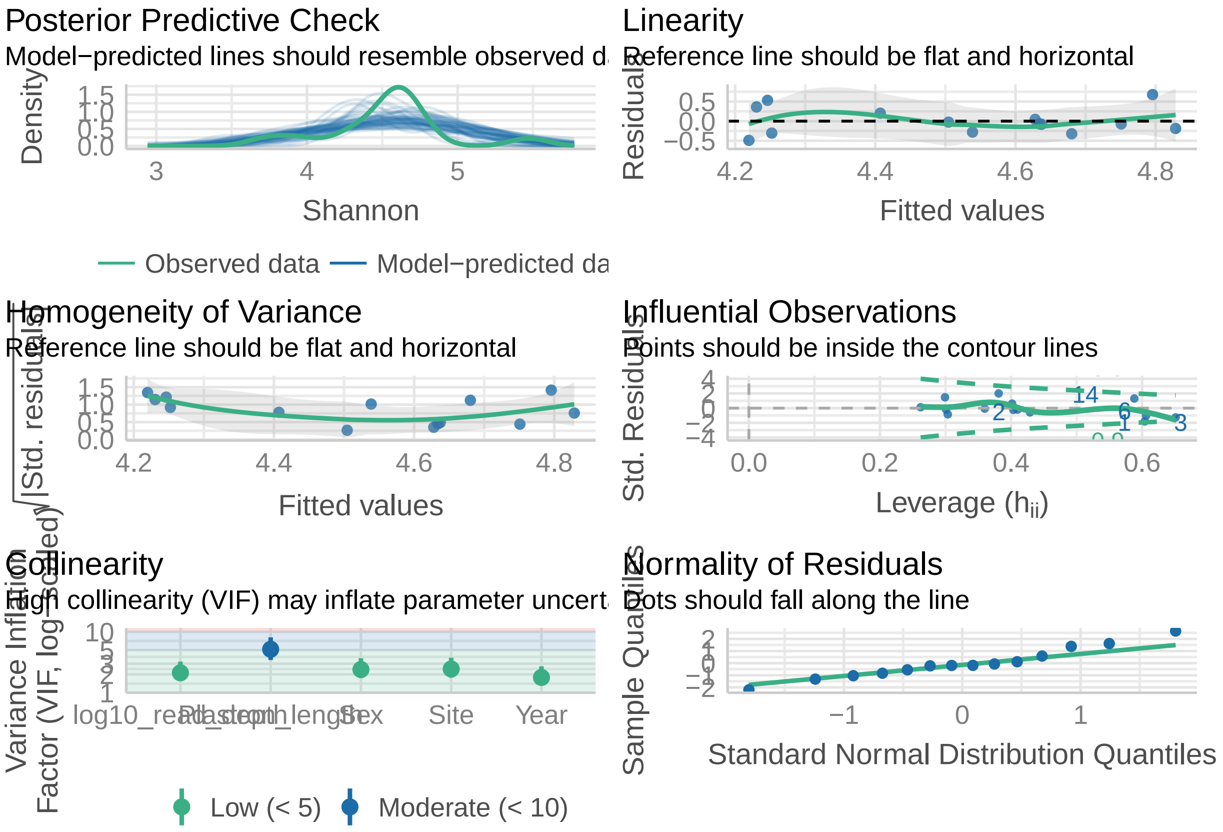


```
plot(pl.shannon.KISU, which = 1)
```

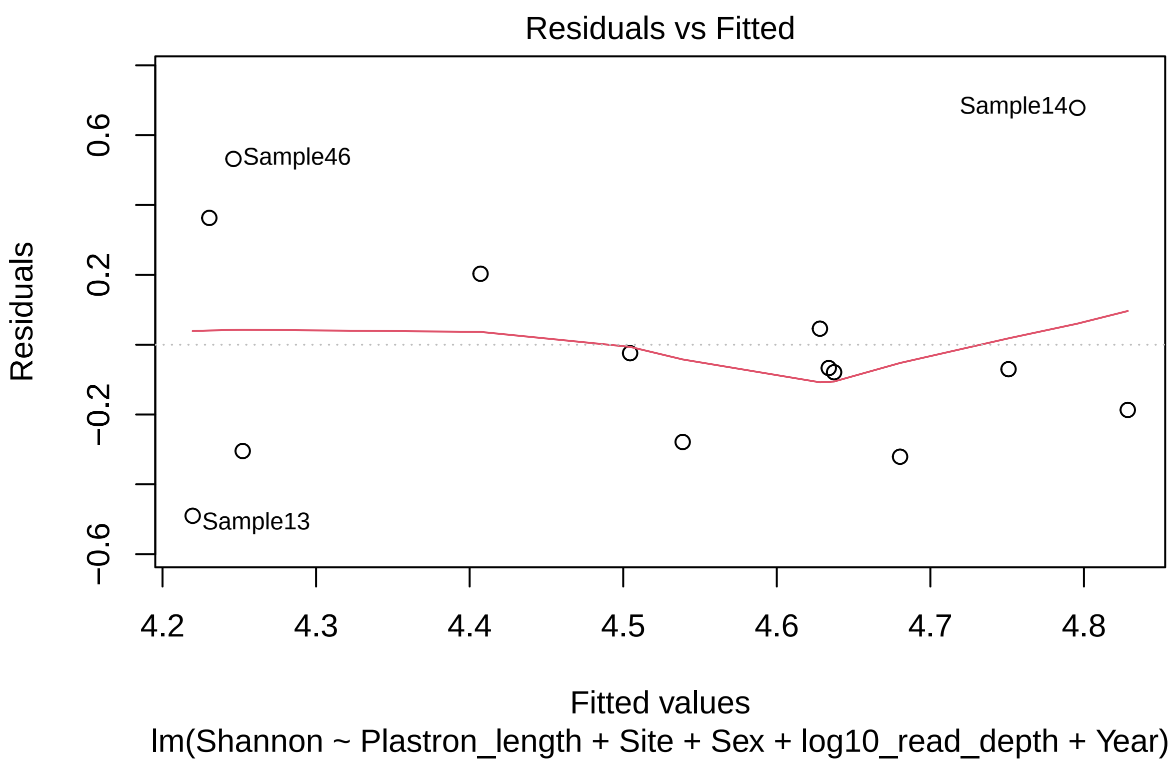


```r
plot(pl.shannon.KISU, which = 2)
```

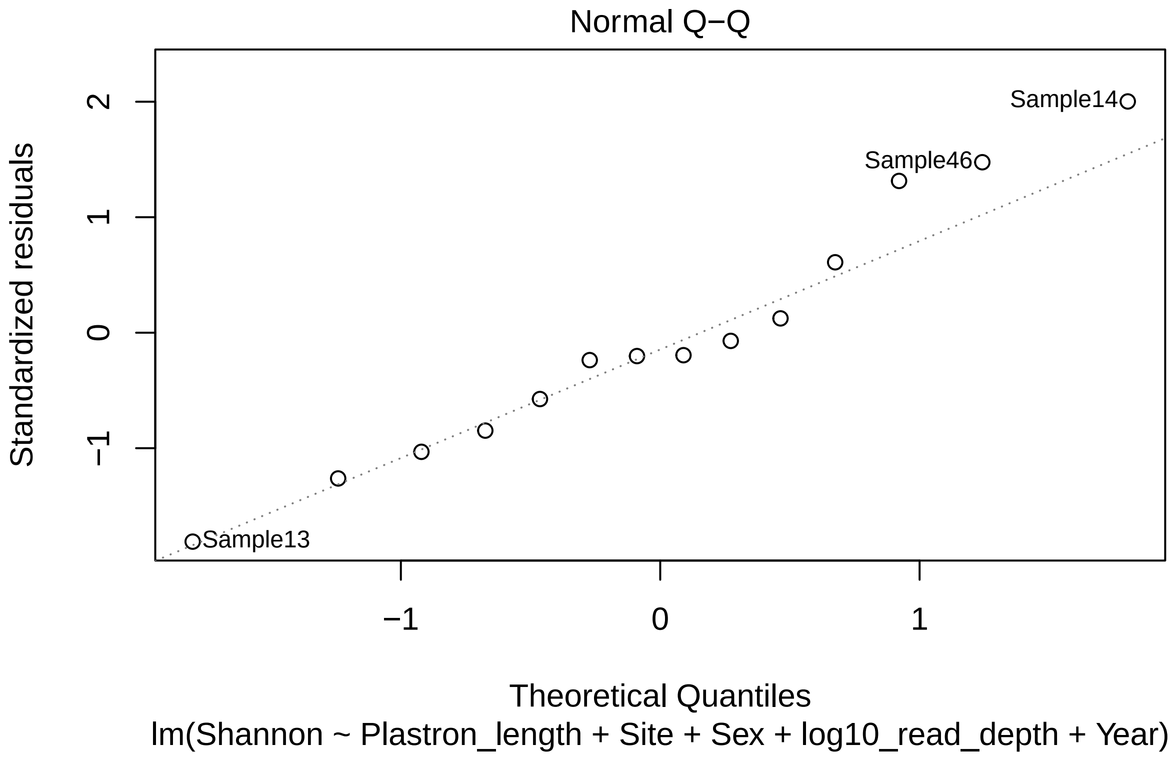


```r
#check for effects in PSCO
#took out variable 'sex' from PSCO model for Shannon diversity, as sex and plastron_length were highly
pl.shannon.PSCO <- lm(Shannon~Plastron_length+log10_read_depth+Year,data=PSCO)
```

```r
summary(pl.shannon.PSCO)
```

```
##
## Call:
## lm(formula = Shannon ~ Plastron_length + log10_read_depth + Year,
##     data = PSCO)
##
## Residuals:
##      Min       1Q   Median       3Q      Max
## -0.72873 -0.29920  0.06667  0.27179  0.63428
##
## Coefficients:
##                   Estimate Std. Error t value Pr(>|t|)
## (Intercept)       2.281e+03  8.191e+02   2.784   0.0238 *
## Plastron_length  -6.281e-03  3.470e-03  -1.810   0.1078
## log10_read_depth -3.404e-01  7.459e-01  -0.456   0.6602
## Year             -1.125e+00  4.047e-01  -2.779   0.0240 *
## ---
## Signif. codes:  0 '***' 0.001 '**' 0.01 '*' 0.05 '.' 0.1 ' ' 1
##
## Residual standard error: 0.5062 on 8 degrees of freedom
## Multiple R-squared:  0.5909, Adjusted R-squared:  0.4375
## F-statistic: 3.852 on 3 and 8 DF,  p-value: 0.05647
```

```r
confint(pl.shannon.PSCO)
```

```
##                         2.5 %        97.5 %
## (Intercept)      391.92575878  4.169604e+03
## Plastron_length   -0.01428221  1.719337e-03
## log10_read_depth  -2.06038346  1.379509e+00
## Year              -2.05797755 -1.913907e-01
```

```r
check_model(pl.shannon.PSCO)
```

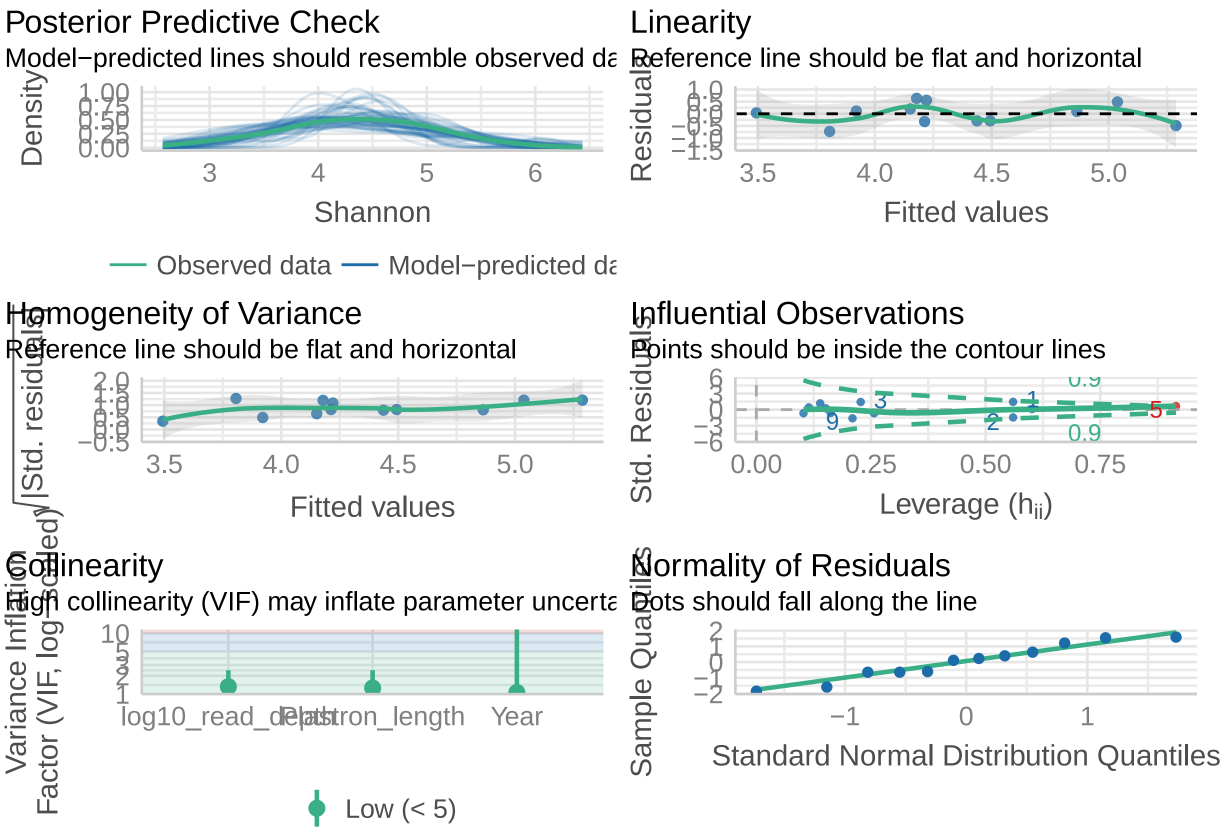


```
plot(pl.shannon.PSCO, which = 1)
```

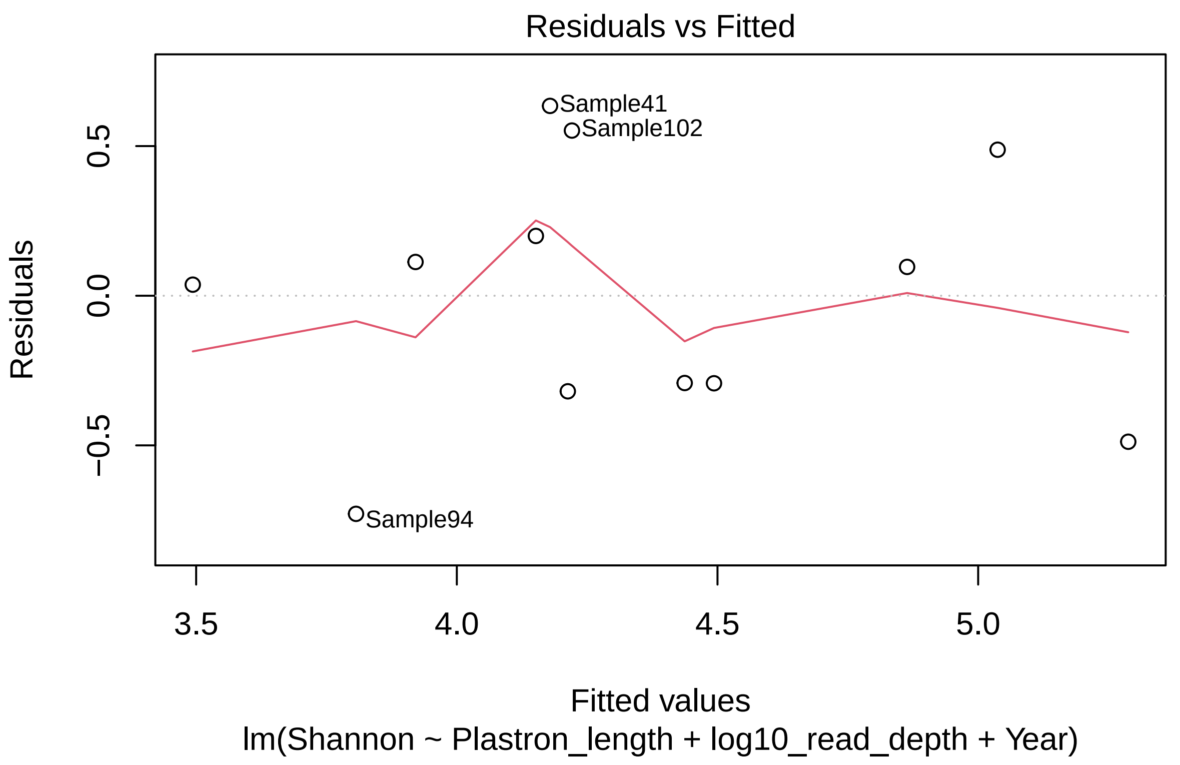

```r
plot(pl.shannon.PSCO, which = 2)
```

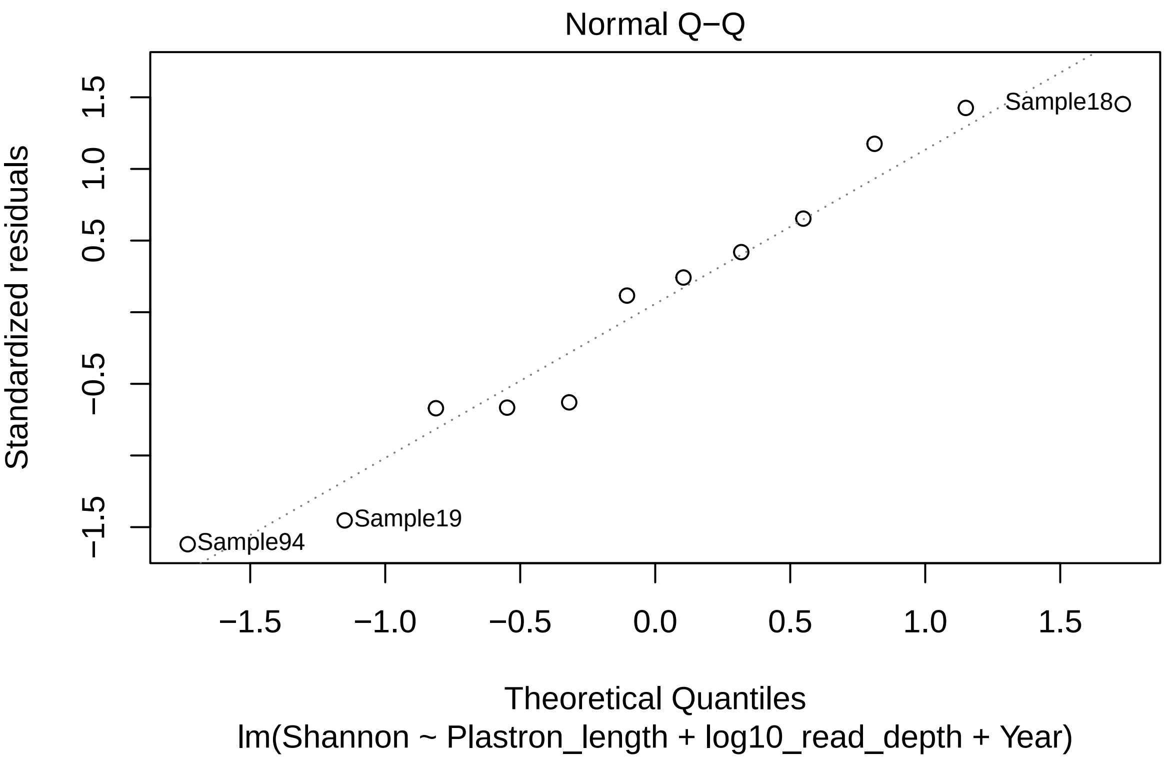


```r
#check for effects in STCA
pl.shannon.STCA <- lm(Shannon~Plastron_length+Sex+log10_read_depth+Year,data=STCA)

summary(pl.shannon.STCA)
```

```
##
## Call:
## lm(formula = Shannon ~ Plastron_length + Sex + log10_read_depth +
##     Year, data = STCA)
##
## Residuals:
##   Sample55   Sample62   Sample63   Sample64   Sample69   Sample70   Sample71
##  5.413e-01 -2.214e-01  4.209e-01 -1.202e+00  1.943e-16  2.817e-01  5.945e-01
##   Sample72
## -4.146e-01
##
## Coefficients: (1 not defined because of singularities)
##                   Estimate Std. Error t value Pr(>|t|)
## (Intercept)      -24.23356   38.71873  -0.626    0.565
## Plastron_length    0.01476    0.03065   0.481    0.655
## SexM              -0.29233    1.22986  -0.238    0.824
## log10_read_depth   5.85123    8.30003   0.705    0.520
## Year                    NA         NA      NA       NA
##
## Residual standard error: 0.8015 on 4 degrees of freedom
## Multiple R-squared:  0.2466, Adjusted R-squared:  -0.3184
## F-statistic: 0.4365 on 3 and 4 DF,  p-value: 0.7391
```

```r
confint(pl.shannon.STCA)
```

```
##                          2.5 %      97.5 %
## (Intercept)       -131.7339939 83.26686621
## Plastron_length     -0.0703397  0.09985329
## SexM                -3.7069586  3.12229327
## log10_read_depth   -17.1933344 28.89580037
## Year                        NA          NA
```

```r
check_model(pl.shannon.STCA)
```

```
## Model matrix is rank deficient. VIFs may not be sensible.
```

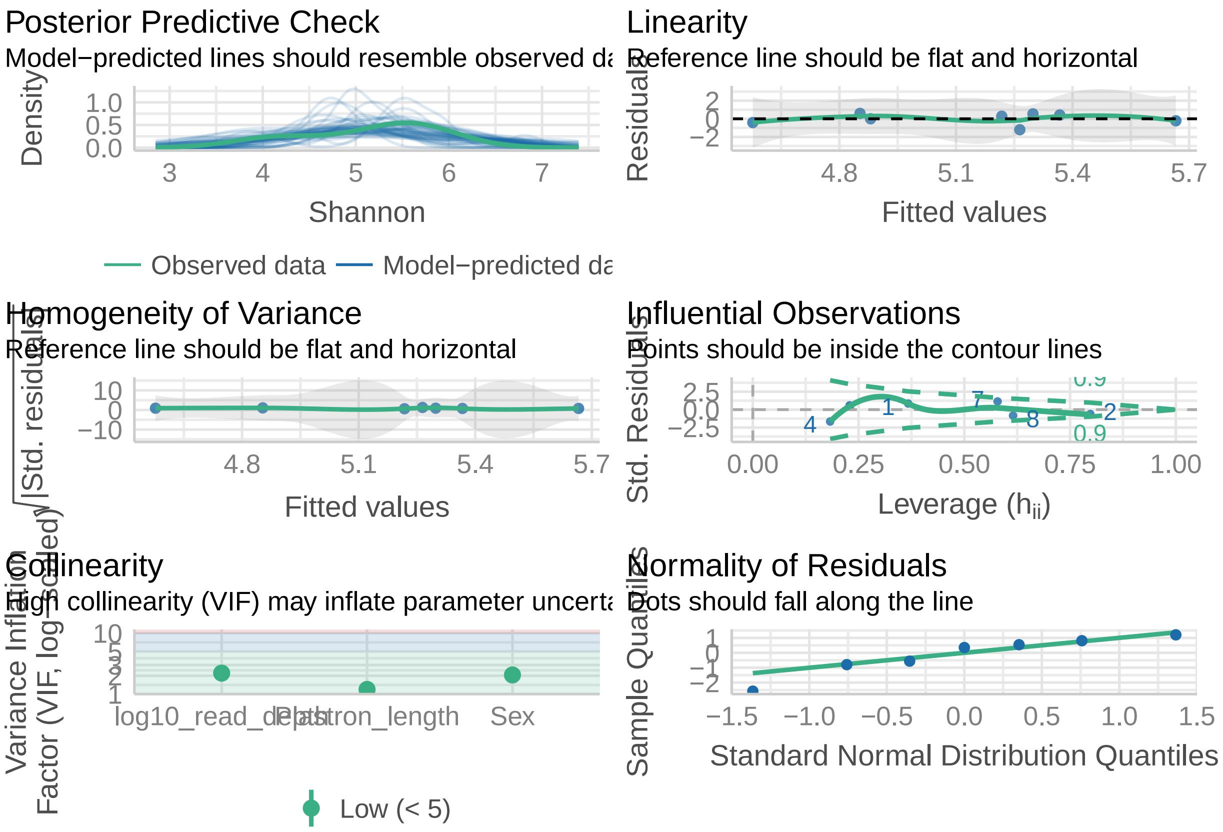


```r
plot(pl.shannon.STCA, which = 1)
```

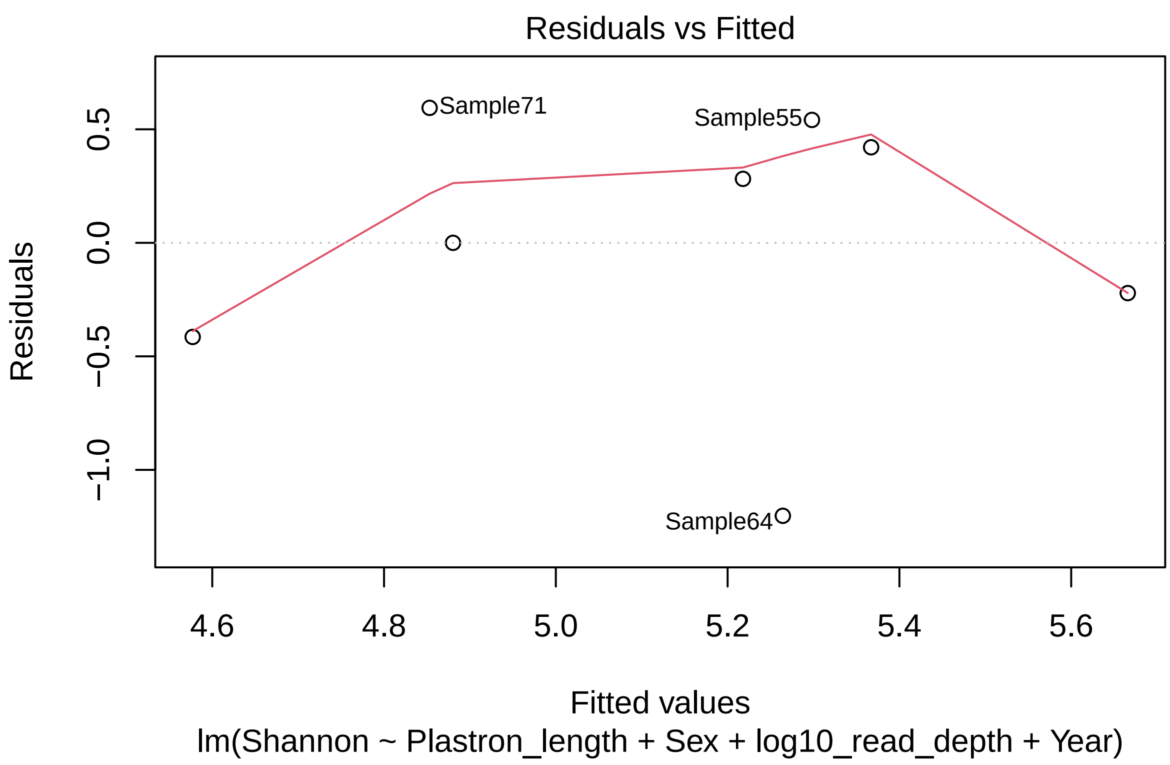


```
plot(pl.shannon.STCA, which = 2)
```

```
## Warning: not plotting observations with leverage one:
##   5
```

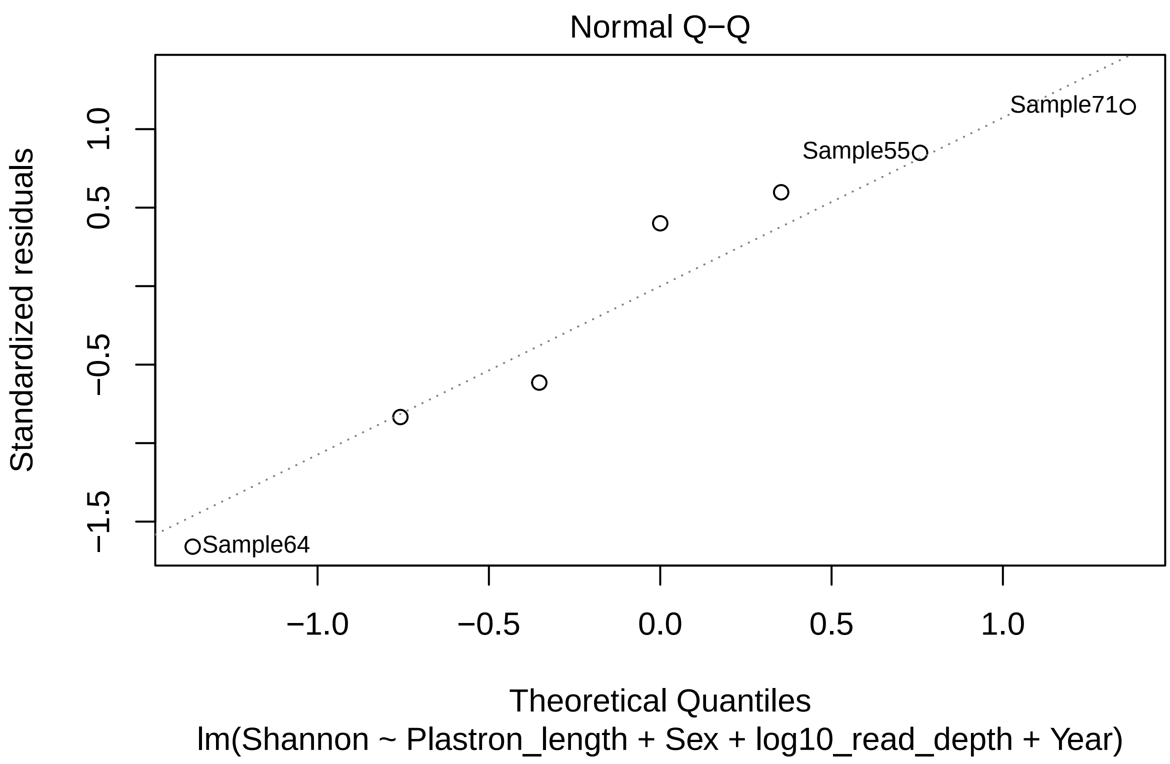

```r
#check for effects in STOD
pl.shannon.STOD <- lm(Shannon~Plastron_length+Sex+log10_read_depth+Year,data=STOD)

summary(pl.shannon.STOD)
```

```
##
## Call:
## lm(formula = Shannon ~ Plastron_length + Sex + log10_read_depth +
##     Year, data = STOD)
##
## Residuals:
##      Min       1Q   Median       3Q      Max
## -0.46839 -0.14082 -0.01283  0.13066  0.79281
##
## Coefficients:
##                   Estimate Std. Error t value Pr(>|t|)
## (Intercept)     -278.72514  425.06392  -0.656   0.5199
## Plastron_length   -0.01208    0.01148  -1.052   0.3059
## SexJ               0.01890    0.50037   0.038   0.9703
## SexM               0.22327    0.19413   1.150   0.2644
## log10_read_depth   1.87033    0.83767   2.233   0.0378 *
## Year               0.13638    0.21169   0.644   0.5271
## ---
## Signif. codes:  0 '***' 0.001 '**' 0.01 '*' 0.05 '.' 0.1 ' ' 1
##
## Residual standard error: 0.3476 on 19 degrees of freedom
## Multiple R-squared:  0.5971, Adjusted R-squared:  0.4911
## F-statistic: 5.632 on 5 and 19 DF,  p-value: 0.002385
```

```r
confint(pl.shannon.STOD)
```

```
##                          2.5 %       97.5 %
## (Intercept)      -1.168394e+03 610.94386114
## Plastron_length  -3.612057e-02   0.01195325
## SexJ             -1.028377e+00   1.06618233
## SexM             -1.830448e-01   0.62957848
## log10_read_depth  1.170616e-01   3.62358927
## Year             -3.066904e-01   0.57945087
```

```r
check_model(pl.shannon.STOD)
```

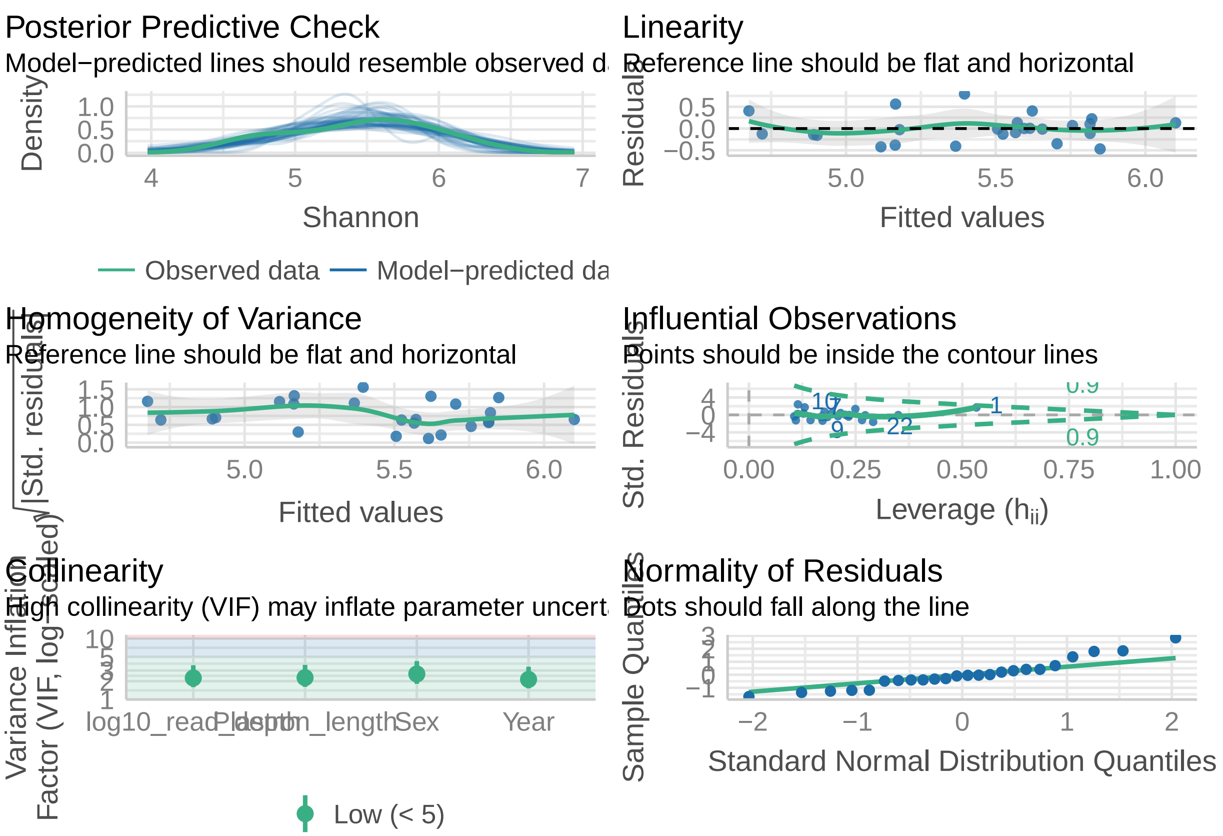


```
plot(pl.shannon.STOD, which = 1)
```

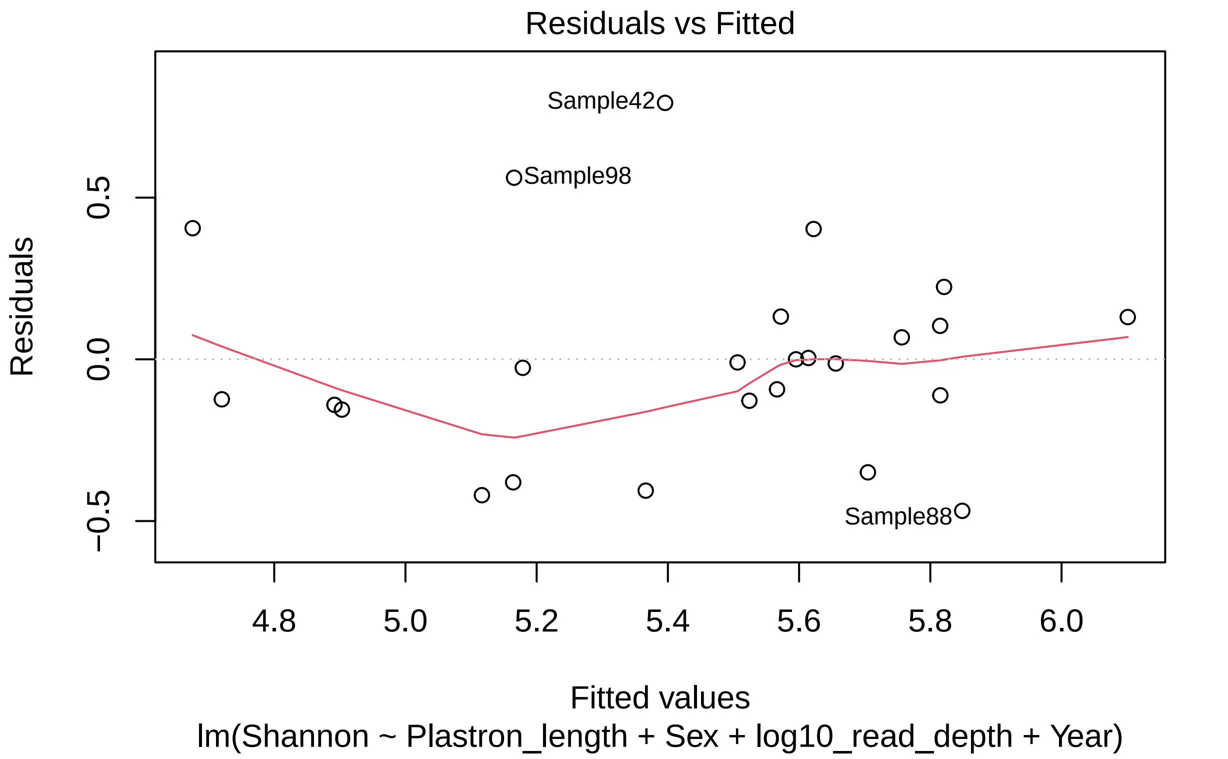

```r
plot(pl.shannon.STOD, which = 2)
```

```
## Warning: not plotting observations with leverage one:
##   3
```

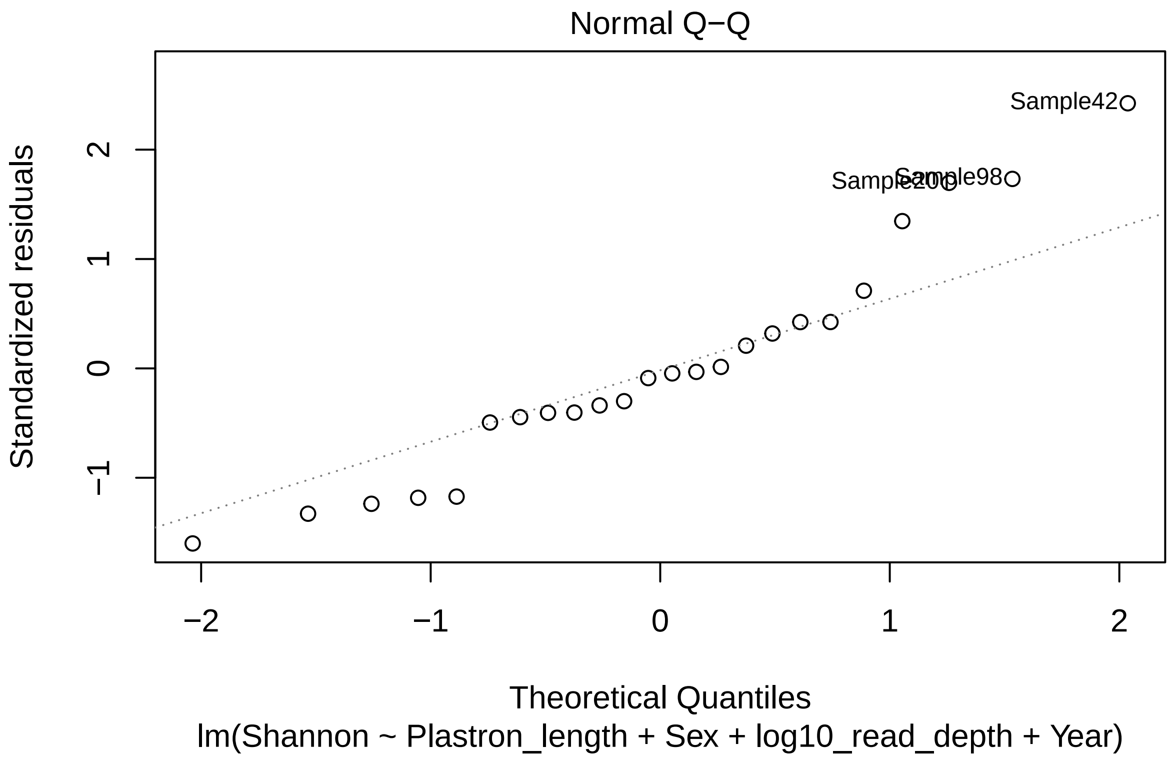


```r
#check for effects in TRSC
pl.shannon.TRSC <- lm(Shannon~Plastron_length+Sex+log10_read_depth+Year,data=TRSC)

summary(pl.shannon.TRSC)
```

```
##
## Call:
## lm(formula = Shannon ~ Plastron_length + Sex + log10_read_depth +
##     Year, data = TRSC)
##
## Residuals:
##     Min      1Q  Median      3Q     Max
## -1.3027 -0.6257  0.0951  0.5639  1.2668
##
## Coefficients:
##                   Estimate Std. Error t value Pr(>|t|)
## (Intercept)      -2.024e+03  1.382e+03  -1.465   0.1624
## Plastron_length   8.721e-03  7.850e-03   1.111   0.2830
## SexJ              8.983e-01  1.118e+00   0.803   0.4336
## SexM              1.173e-01  3.955e-01   0.297   0.7705
## log10_read_depth -5.720e+00  2.797e+00  -2.045   0.0577 .
## Year              1.017e+00  6.889e-01   1.476   0.1594
## ---
```

```
## Signif. codes:  0 '***' 0.001 '**' 0.01 '*' 0.05 '.' 0.1 ' ' 1
##
## Residual standard error: 0.777 on 16 degrees of freedom
## Multiple R-squared:  0.2623, Adjusted R-squared:  0.03172
## F-statistic: 1.138 on 5 and 16 DF,  p-value: 0.3808
```

```
confint(pl.shannon.TRSC)
```

```
##                          2.5 %       97.5 %
## (Intercept)       -4.953319e+03 905.62861301
## Plastron_length   -7.920411e-03   0.02536291
## SexJ              -1.472438e+00   3.26900078
## SexM              -7.210218e-01   0.95570041
## log10_read_depth  -1.164976e+01   0.21049708
## Year              -4.437132e-01   2.47712364
```

```
check_model(pl.shannon.TRSC)
```

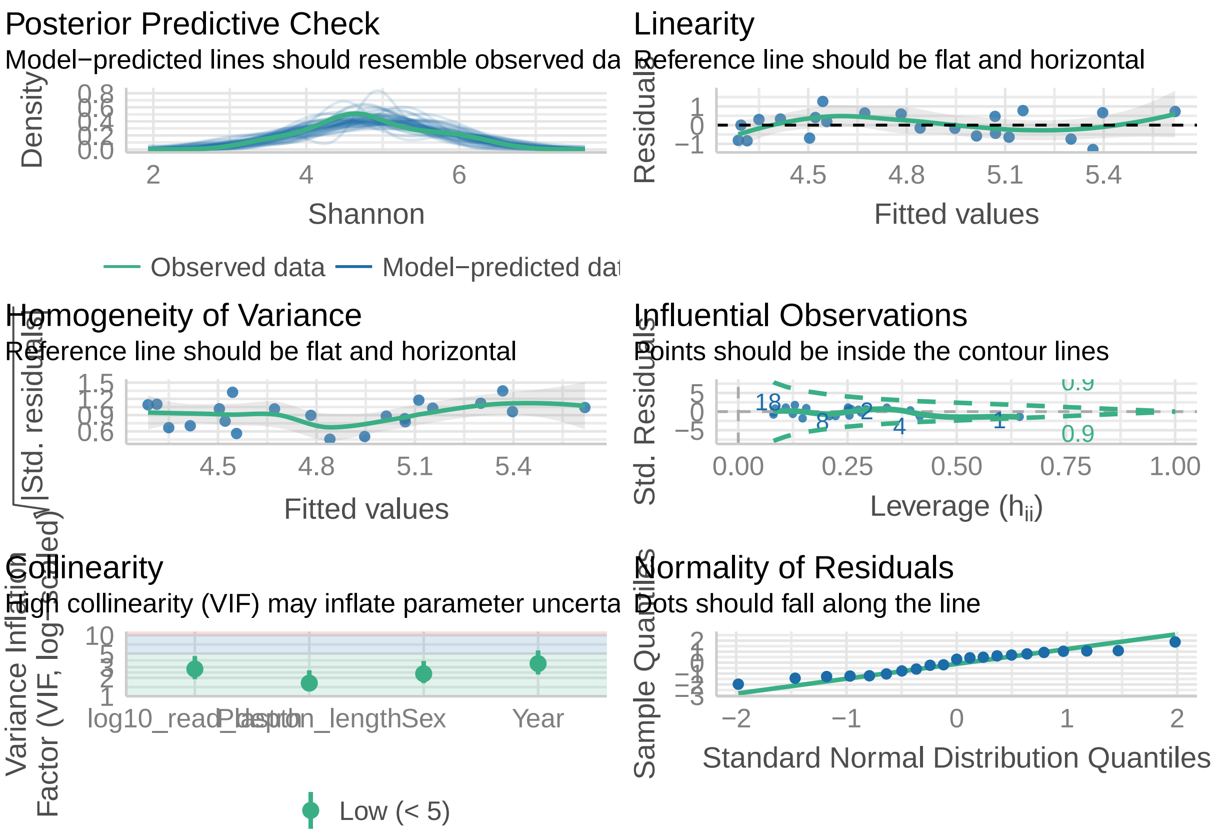

```r
plot(pl.shannon.TRSC, which = 1)
```

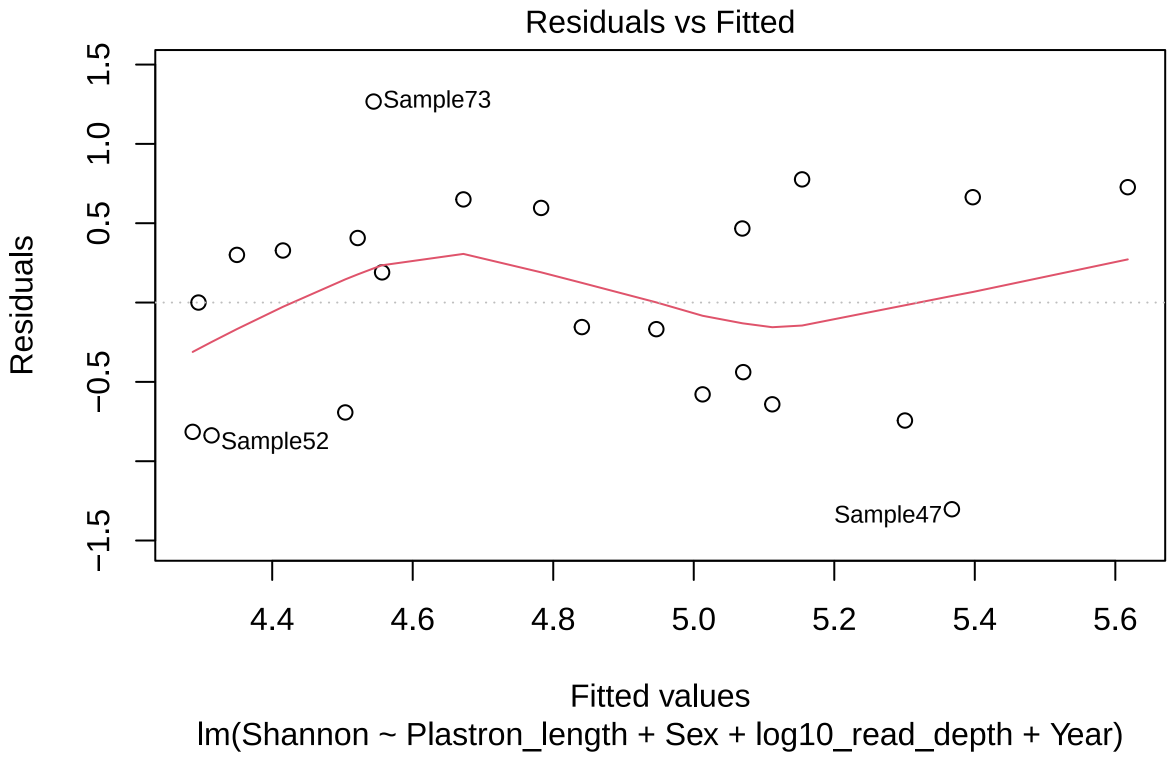


```r
plot(pl.shannon.TRSC, which = 2)
```

```
## Warning: not plotting observations with leverage one:
##   6
```

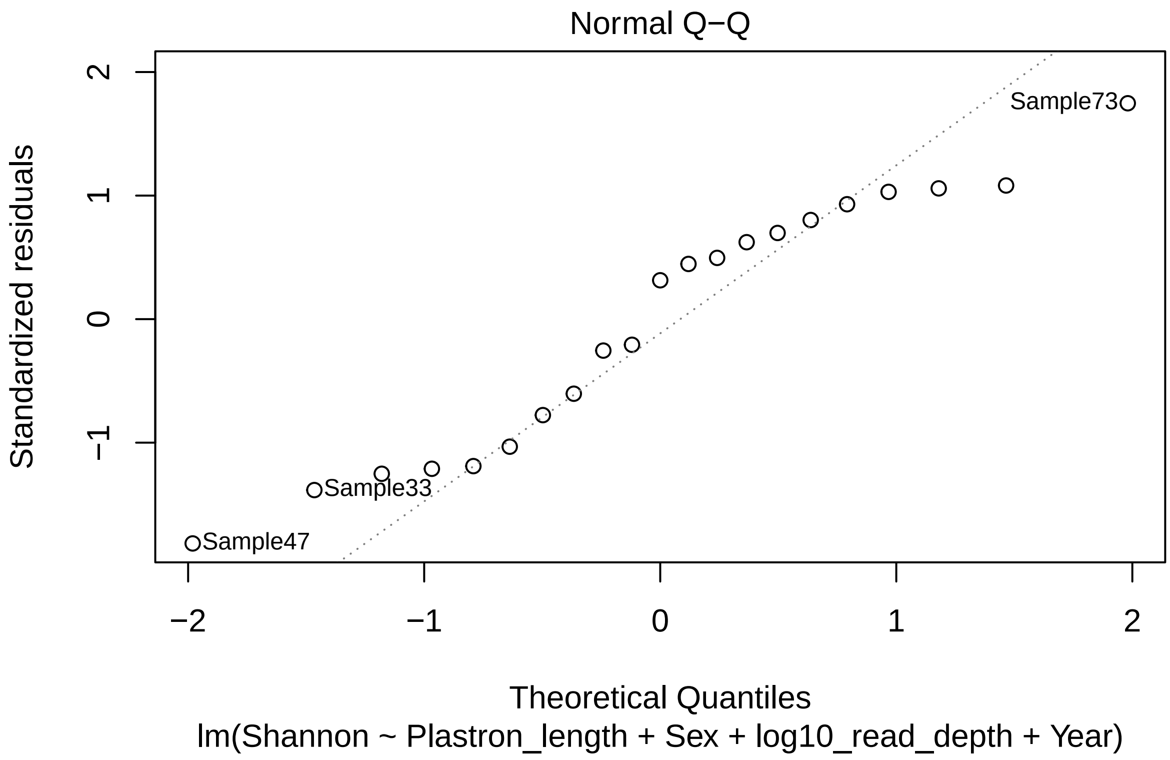


```r
#Check observed counts
#check for effects in KISU
pl.Observed.KISU <- lm(Observed~Plastron_length+Site+Sex+log10_read_depth+Year,data=KISU)

summary(pl.Observed.KISU)
```

```
##
## Call:
## lm(formula = Observed ~ Plastron_length + Site + Sex + log10_read_depth +
##     Year, data = KISU)
##
## Residuals:
##     Min      1Q  Median      3Q     Max
## -145.78  -63.36   13.23   28.61  175.66
##
## Coefficients:
##                    Estimate Std. Error t value Pr(>|t|)
## (Intercept)      -39064.006 164916.470  -0.237    0.819
## Plastron_length       2.054      5.461   0.376    0.717
## SiteS4               95.449     92.054   1.037    0.330
## SexM                 -7.517     91.115  -0.083    0.936
## log10_read_depth    437.648    270.210   1.620    0.144
## Year                 18.346     81.848   0.224    0.828
##
## Residual standard error: 109.9 on 8 degrees of freedom
## Multiple R-squared:  0.4279, Adjusted R-squared:  0.07034
## F-statistic: 1.197 on 5 and 8 DF,  p-value: 0.3902
```

```r
confint(pl.Observed.KISU)
```

```
##                            2.5 %       97.5 %
## (Intercept)       -419362.06793 341234.05617
## Plastron_length       -10.53892     14.64764
## SiteS4               -116.82862    307.72737
## SexM                 -217.62859    202.59456
## log10_read_depth     -185.45736   1060.75244
## Year                 -170.39618    207.08746
```

```r
check_model(pl.Observed.KISU)
```

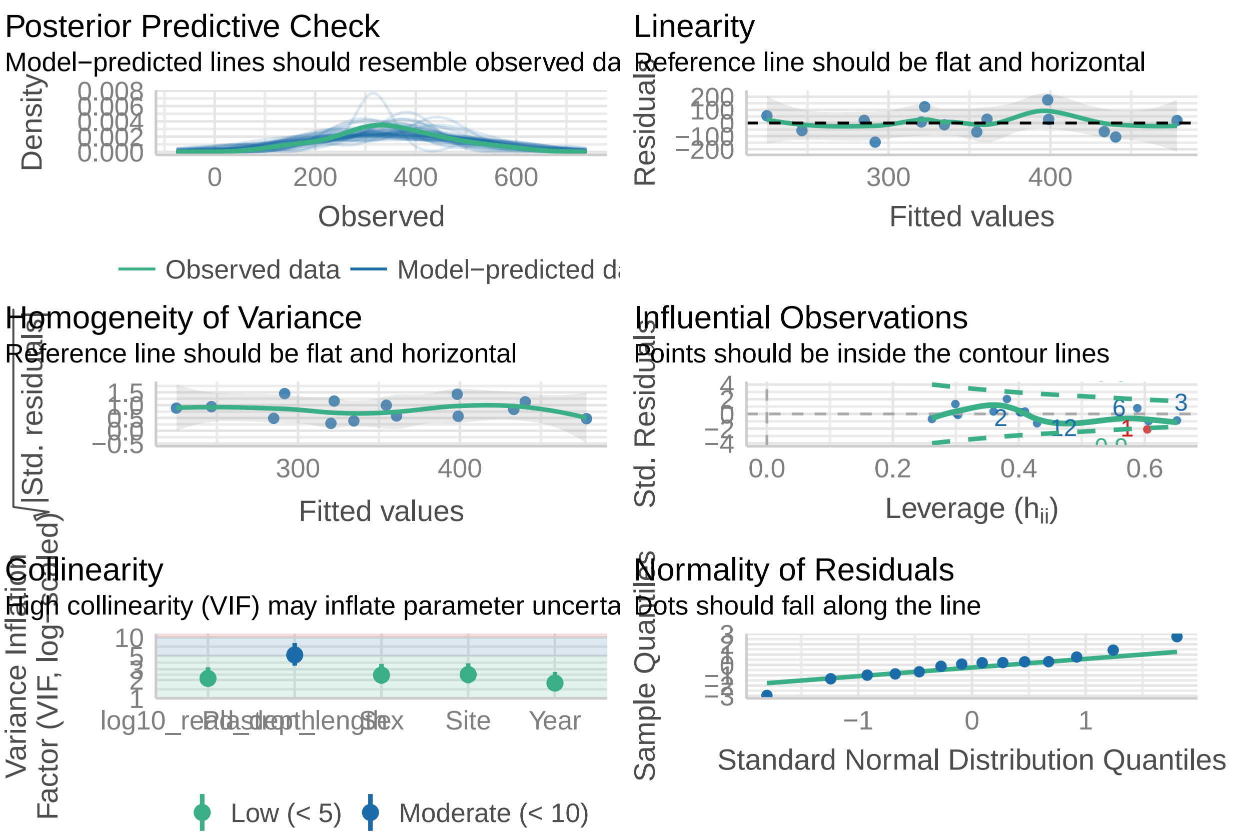


```r
plot(pl.Observed.KISU, which = 1)
```

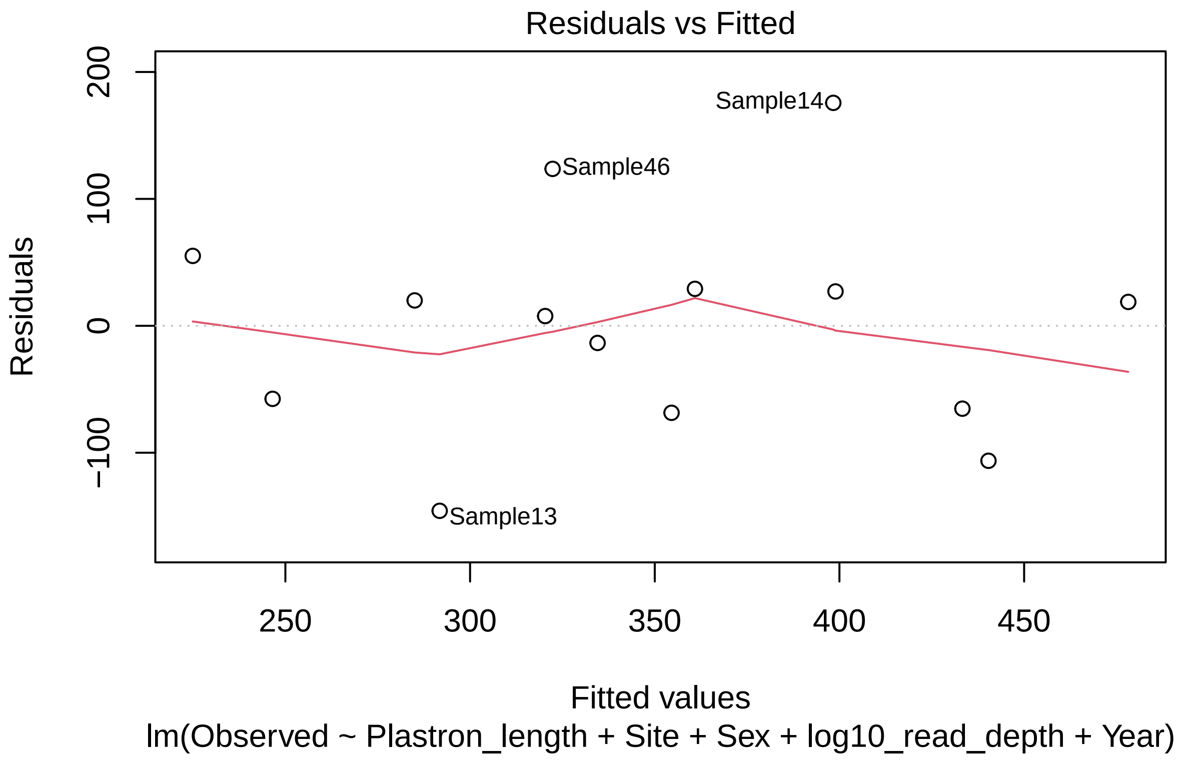


```r
plot(pl.Observed.KISU, which = 2)
```

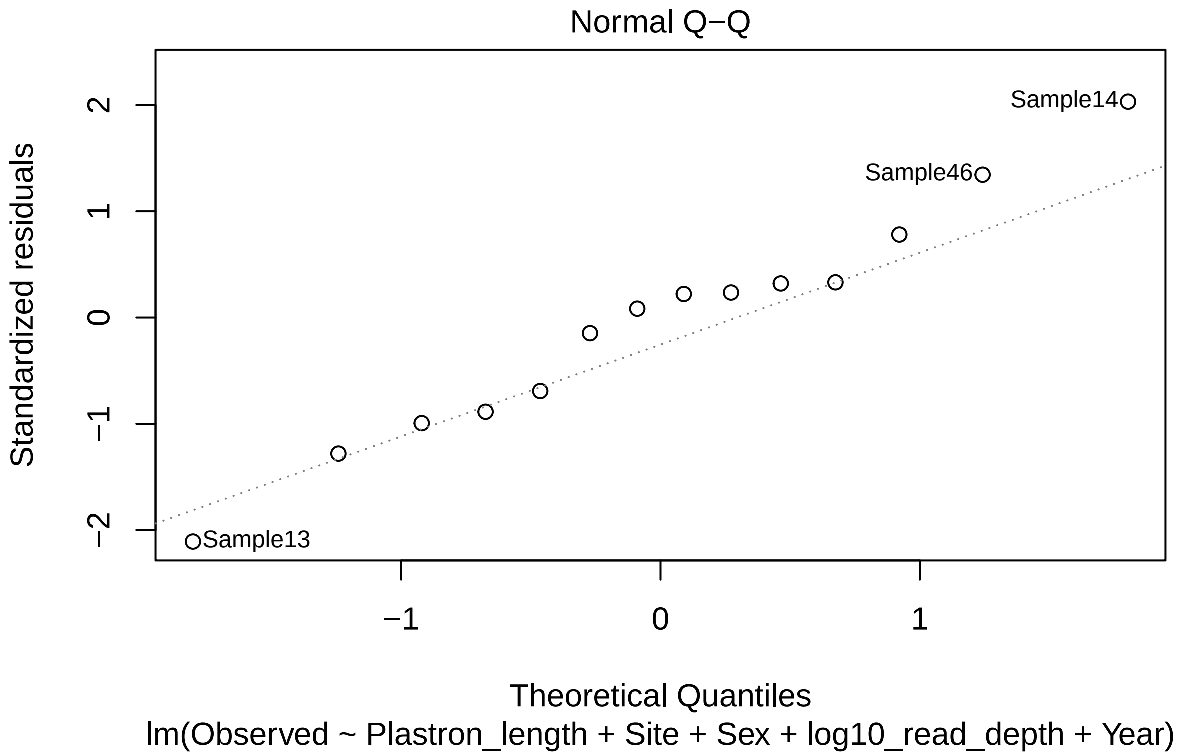


```r
#check for effects in PSCO
#took out variable 'sex' from PSCO model for observed features, as sex and plastron_length were highly
pl.Observed.PSCO <- lm(Observed~Plastron_length+log10_read_depth+Year,data=PSCO)
```

```r
summary(pl.Observed.PSCO)
```

```
##
## Call:
## lm(formula = Observed ~ Plastron_length + log10_read_depth +
##     Year, data = PSCO)
##
## Residuals:
##      Min       1Q   Median       3Q      Max
## -128.322  -44.690    3.417   30.415  179.763
##
## Coefficients:
##                    Estimate Std. Error t value Pr(>|t|)
## (Intercept)       3.308e+05  1.496e+05   2.210   0.0580 .
## Plastron_length  -6.194e-01  6.338e-01  -0.977   0.3570
## log10_read_depth  2.887e+02  1.363e+02   2.119   0.0669 .
## Year             -1.641e+02  7.394e+01  -2.219   0.0573 .
## ---
## Signif. codes:  0 '***' 0.001 '**' 0.01 '*' 0.05 '.' 0.1 ' ' 1
##
## Residual standard error: 92.47 on 8 degrees of freedom
## Multiple R-squared:  0.6036, Adjusted R-squared:  0.4549
## F-statistic:  4.06 on 3 and 8 DF,  p-value: 0.05017
```

```r
confint(pl.Observed.PSCO)
```

```
##                          2.5 %       97.5 %
## (Intercept)      -14313.090414 6.758143e+05
## Plastron_length      -2.081071 8.421797e-01
## log10_read_depth    -25.460248 6.029584e+02
## Year               -334.550616 6.447849e+00
```

```r
check_model(pl.Observed.PSCO)
```

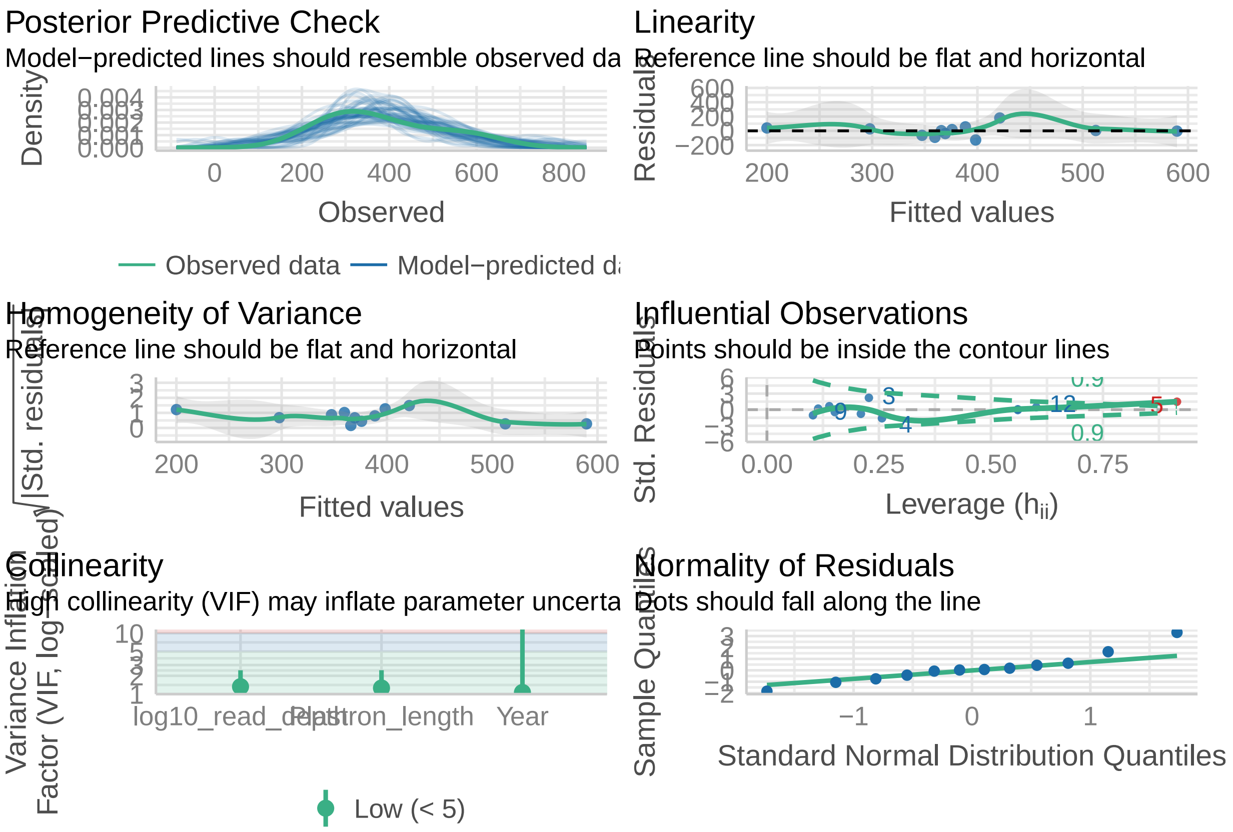


```r
plot(pl.Observed.PSCO, which = 1)
```

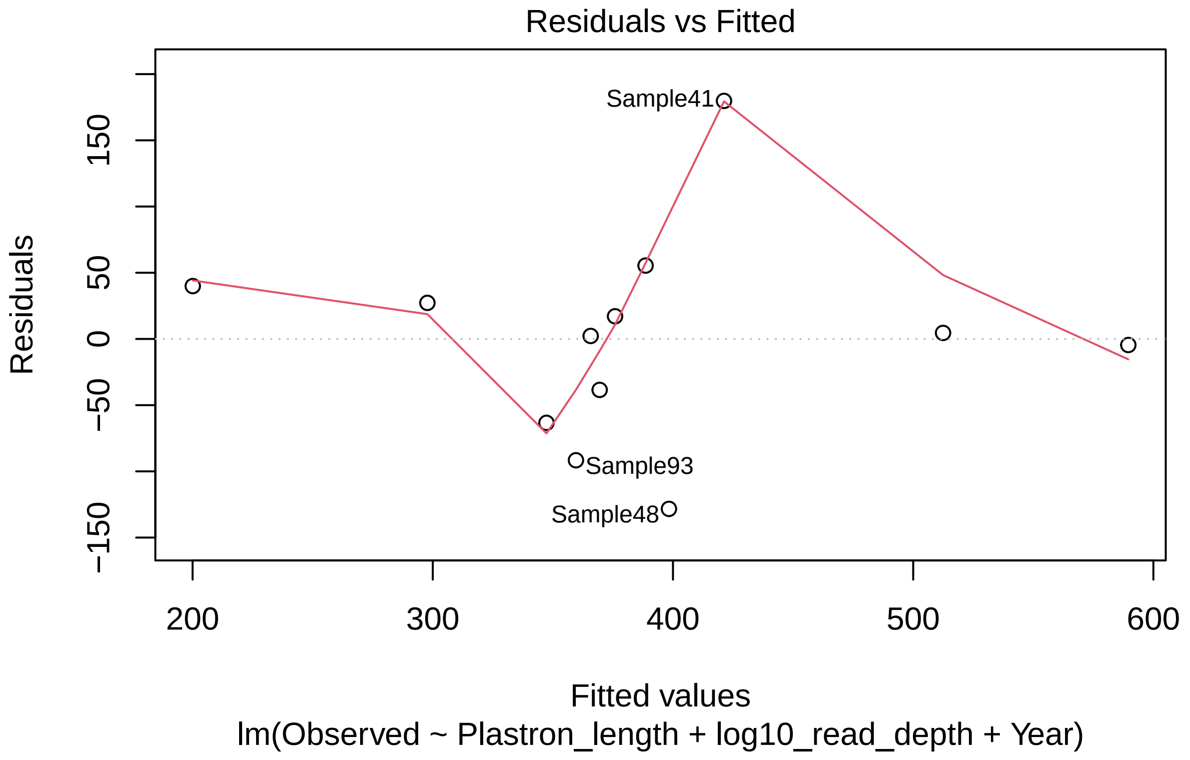

```r
plot(pl.Observed.PSCO, which = 2)
```

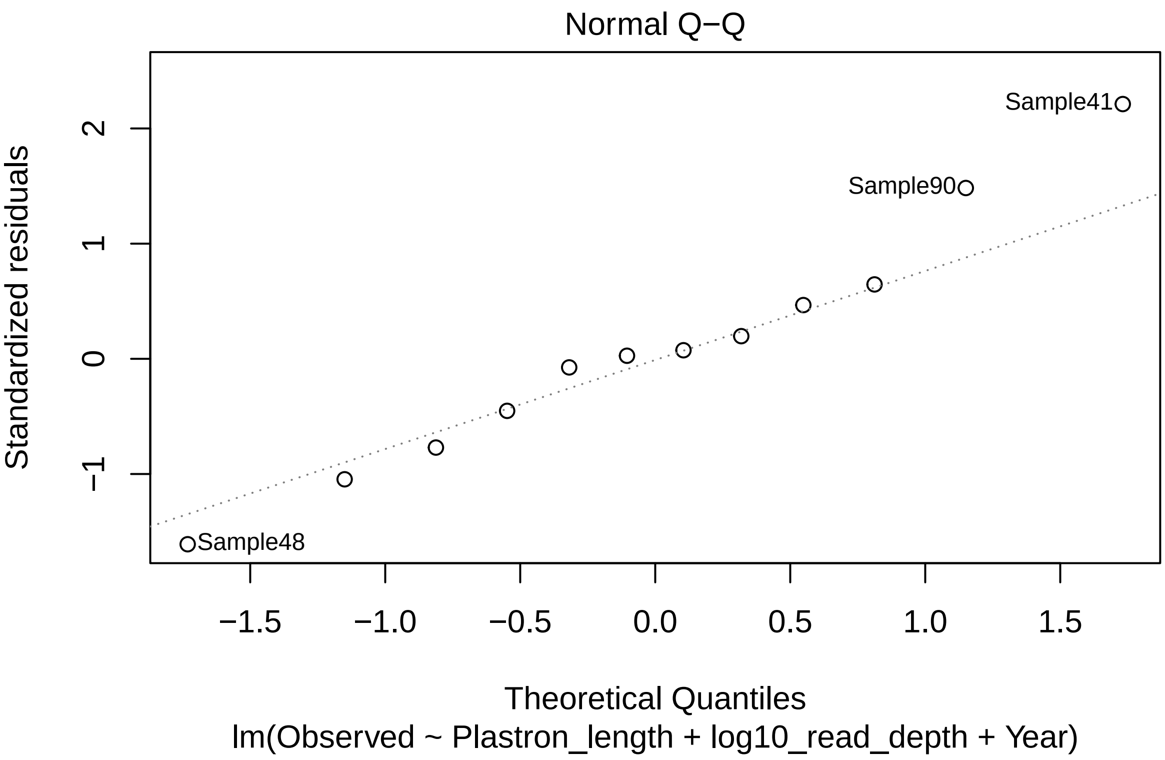


```r
#check for effects in STCA
pl.Observed.STCA <- lm(Observed~Plastron_length+Sex+log10_read_depth+Year,data=STCA)

summary(pl.Observed.STCA)
```

```
##
## Call:
## lm(formula = Observed ~ Plastron_length + Sex + log10_read_depth +
##     Year, data = STCA)
##
## Residuals:
##   Sample55   Sample62   Sample63   Sample64   Sample69   Sample70   Sample71
##  8.907e+01 -3.227e+01  9.856e+01 -2.597e+02  4.619e-14  6.991e+01  9.697e+01
##   Sample72
## -6.249e+01
##
## Coefficients: (1 not defined because of singularities)
##                  Estimate Std. Error t value Pr(>|t|)
## (Intercept)      -9123.095   7802.772  -1.169    0.307
## Plastron_length      2.733      6.177   0.443    0.681
## SexM               -74.849    247.846  -0.302    0.778
## log10_read_depth  1949.431   1672.659   1.165    0.309
## Year                    NA         NA      NA       NA
##
## Residual standard error: 161.5 on 4 degrees of freedom
## Multiple R-squared:  0.4168, Adjusted R-squared:  -0.02059
## F-statistic: 0.9529 on 3 and 4 DF,  p-value: 0.4955
```

```r
confint(pl.Observed.STCA)
```

```
##                          2.5 %      97.5 %
## (Intercept)       -30787.06440 12540.87450
## Plastron_length      -14.41581    19.88225
## SexM                -762.98023   613.28129
## log10_read_depth   -2694.61386  6593.47557
## Year                        NA          NA
```

```r
check_model(pl.Observed.STCA)
```

```
## Model matrix is rank deficient. VIFs may not be sensible.
```

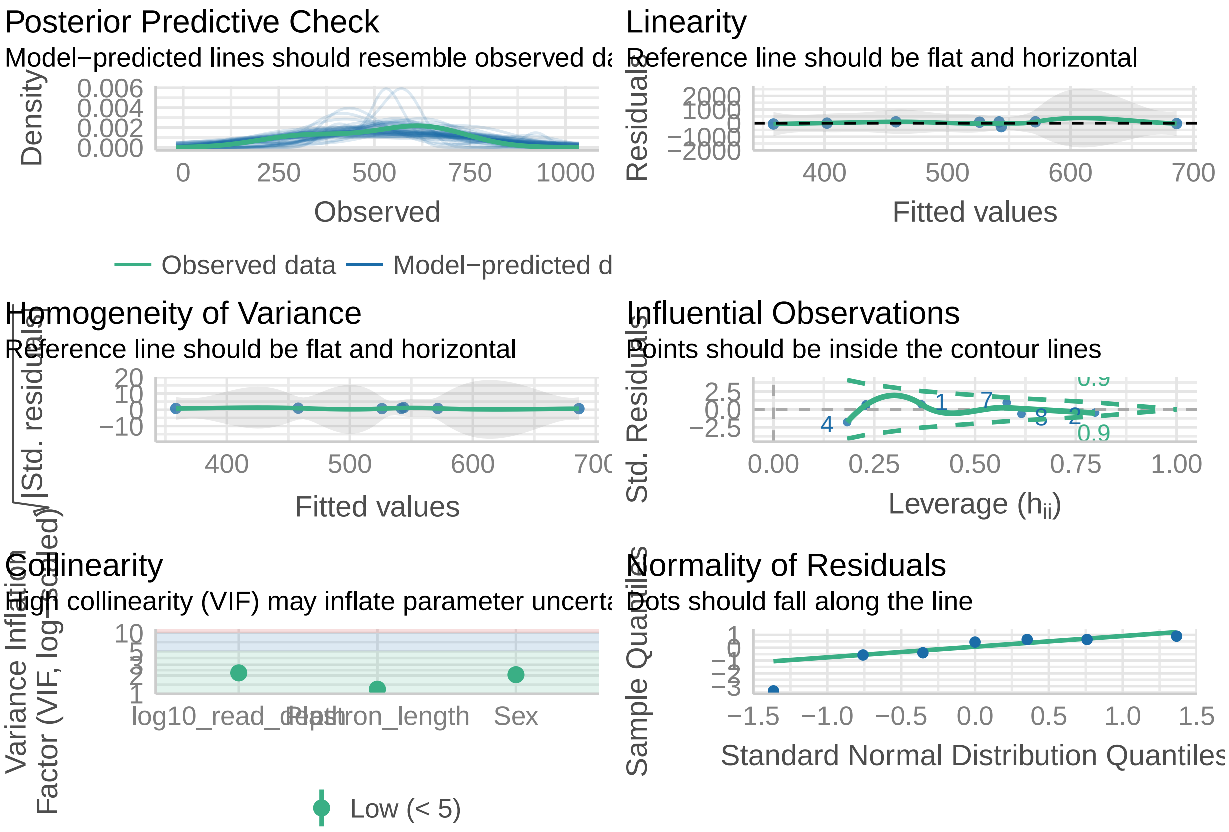


```r
plot(pl.Observed.STCA, which = 1)
```

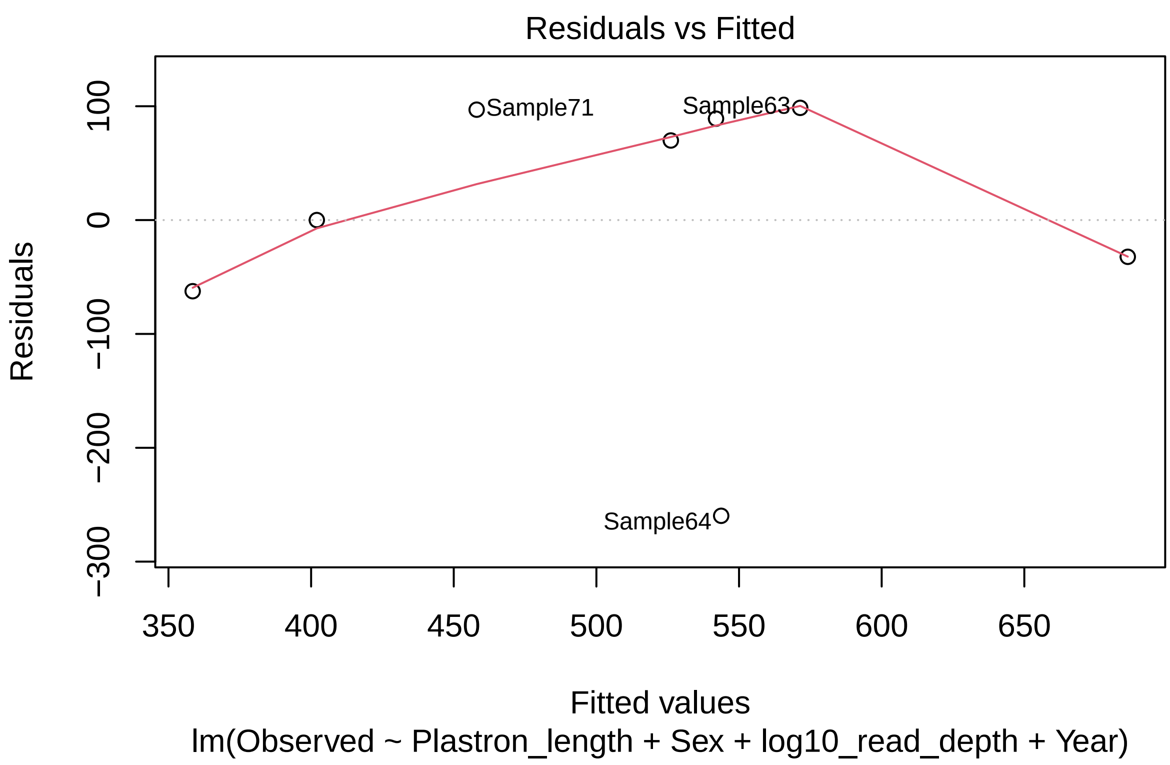


```
plot(pl.Observed.STCA, which = 2)
```

```
## Warning: not plotting observations with leverage one:
##   5
```

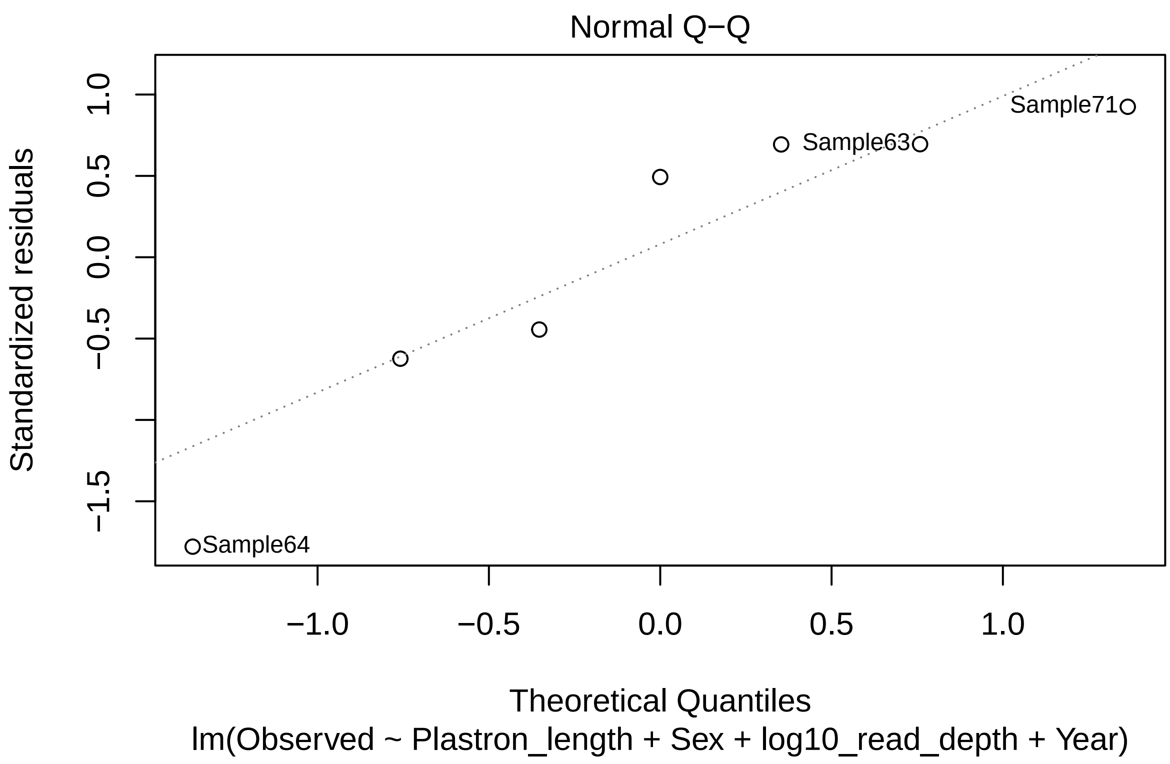

```r
#check for effects in STOD
pl.Observed.STOD <- lm(Observed~Plastron_length+Sex+log10_read_depth+Year,data=STOD)

summary(pl.Observed.STOD)
```

```
##
## Call:
## lm(formula = Observed ~ Plastron_length + Sex + log10_read_depth +
##     Year, data = STOD)
##
## Residuals:
##     Min      1Q  Median      3Q     Max
## -189.54  -74.39  -29.11   75.36  271.03
##
## Coefficients:
##                    Estimate Std. Error t value Pr(>|t|)
## (Intercept)       32627.587 161207.486   0.202  0.84176
## Plastron_length      -2.238      4.355  -0.514  0.61325
## SexJ                -12.050    189.766  -0.063  0.95003
## SexM                 13.122     73.623   0.178  0.86042
## log10_read_depth   1232.267    317.690   3.879  0.00101 **
## Year                -18.726     80.284  -0.233  0.81807
## ---
## Signif. codes:  0 '***' 0.001 '**' 0.01 '*' 0.05 '.' 0.1 ' ' 1
##
## Residual standard error: 131.8 on 19 degrees of freedom
## Multiple R-squared:  0.6496, Adjusted R-squared:  0.5574
## F-statistic: 7.045 on 5 and 19 DF,  p-value: 0.0007051
```

```r
confint(pl.Observed.STOD)
```

```
##                          2.5 %       97.5 %
## (Intercept)      -304783.55870 3.700387e+05
## Plastron_length      -11.35437 6.877848e+00
## SexJ                -409.23554 3.851358e+02
## SexM                -140.97312 1.672181e+02
## log10_read_depth     567.33354 1.897201e+03
## Year                -186.76245 1.493108e+02
```

```r
check_model(pl.Observed.STOD)
```

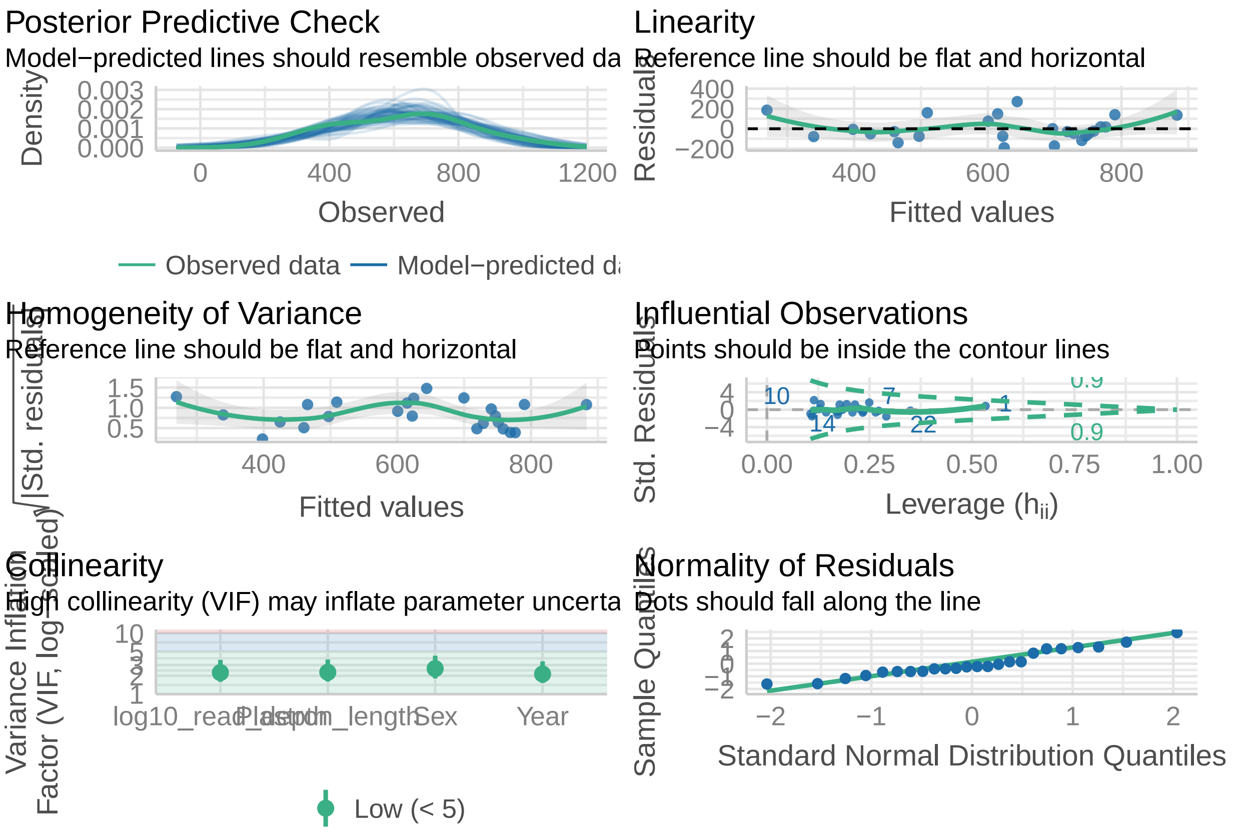


```r
plot(pl.Observed.STOD, which = 1)
```

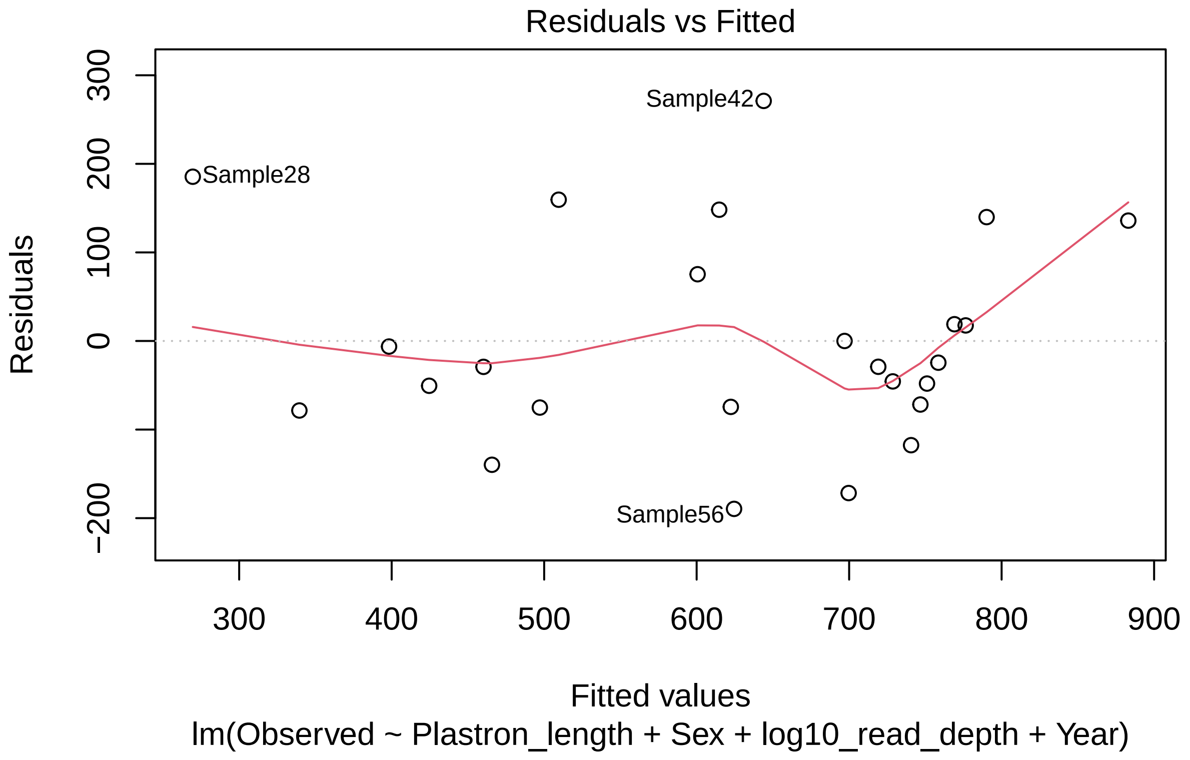

```r
plot(pl.Observed.STOD, which = 2)
```

```
## Warning: not plotting observations with leverage one:
##   3
```

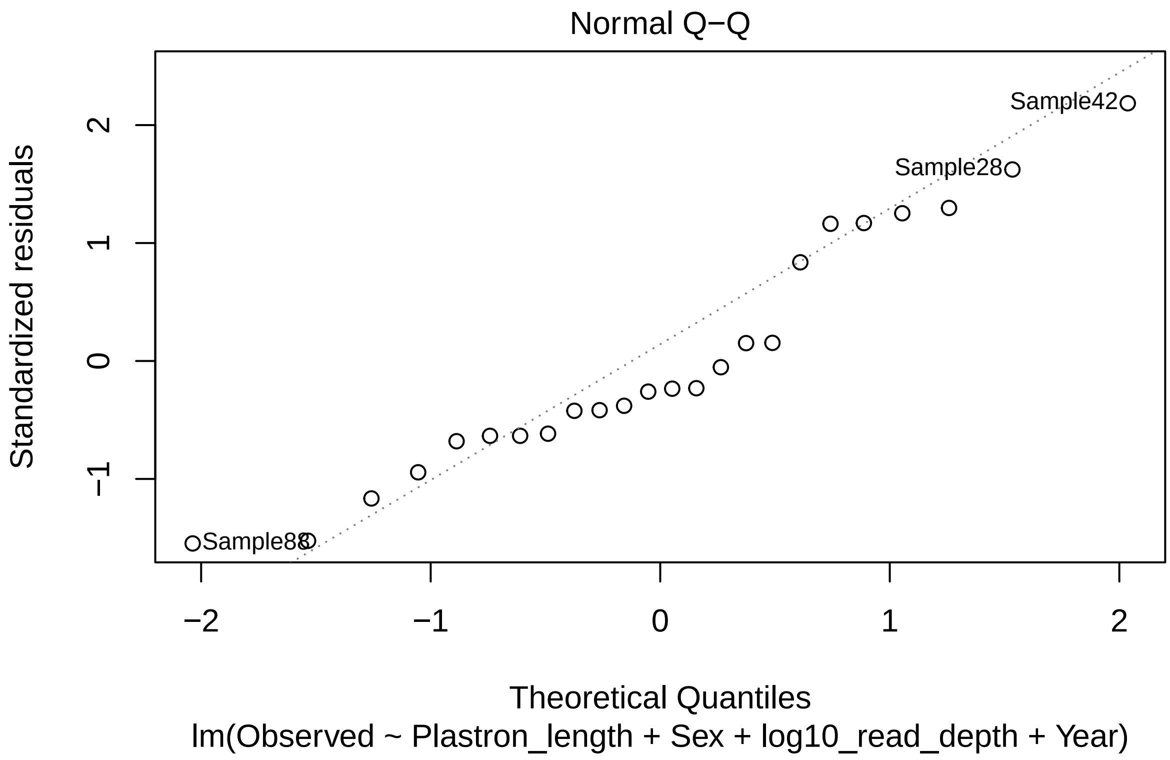


```r
#check for effects in TRSC
pl.Observed.TRSC <- lm(Observed~Plastron_length+Sex+log10_read_depth+Year,data=TRSC)

summary(pl.Observed.TRSC)
```

```
##
## Call:
## lm(formula = Observed ~ Plastron_length + Sex + log10_read_depth +
##     Year, data = TRSC)
##
## Residuals:
##     Min      1Q  Median      3Q     Max
## -281.67  -81.26   15.79   84.53  228.05
##
## Coefficients:
##                    Estimate Std. Error t value Pr(>|t|)
## (Intercept)      -7.226e+05  2.973e+05  -2.431   0.0272 *
## Plastron_length   2.095e+00  1.689e+00   1.241   0.2326
## SexJ              2.831e+02  2.406e+02   1.177   0.2564
## SexM             -4.422e+00  8.507e+01  -0.052   0.9592
## log10_read_depth -8.782e+02  6.017e+02  -1.459   0.1638
## Year              3.597e+02  1.482e+02   2.427   0.0274 *
## ---
```

```
## Signif. codes:  0 '***' 0.001 '**' 0.01 '*' 0.05 '.' 0.1 ' ' 1
##
## Residual standard error: 167.1 on 16 degrees of freedom
## Multiple R-squared:  0.3814, Adjusted R-squared:  0.1881
## F-statistic: 1.973 on 5 and 16 DF,  p-value: 0.1378
```

```
confint(pl.Observed.TRSC)
```

```
##                          2.5 %        97.5 %
## (Intercept)       -1.352798e+06 -92471.385061
## Plastron_length   -1.484877e+00      5.674747
## SexJ              -2.268471e+02    793.090733
## SexM              -1.847628e+02    175.919413
## log10_read_depth  -2.153842e+03    397.435442
## Year               4.554869e+01    673.854193
```

```
check_model(pl.Observed.TRSC)
```

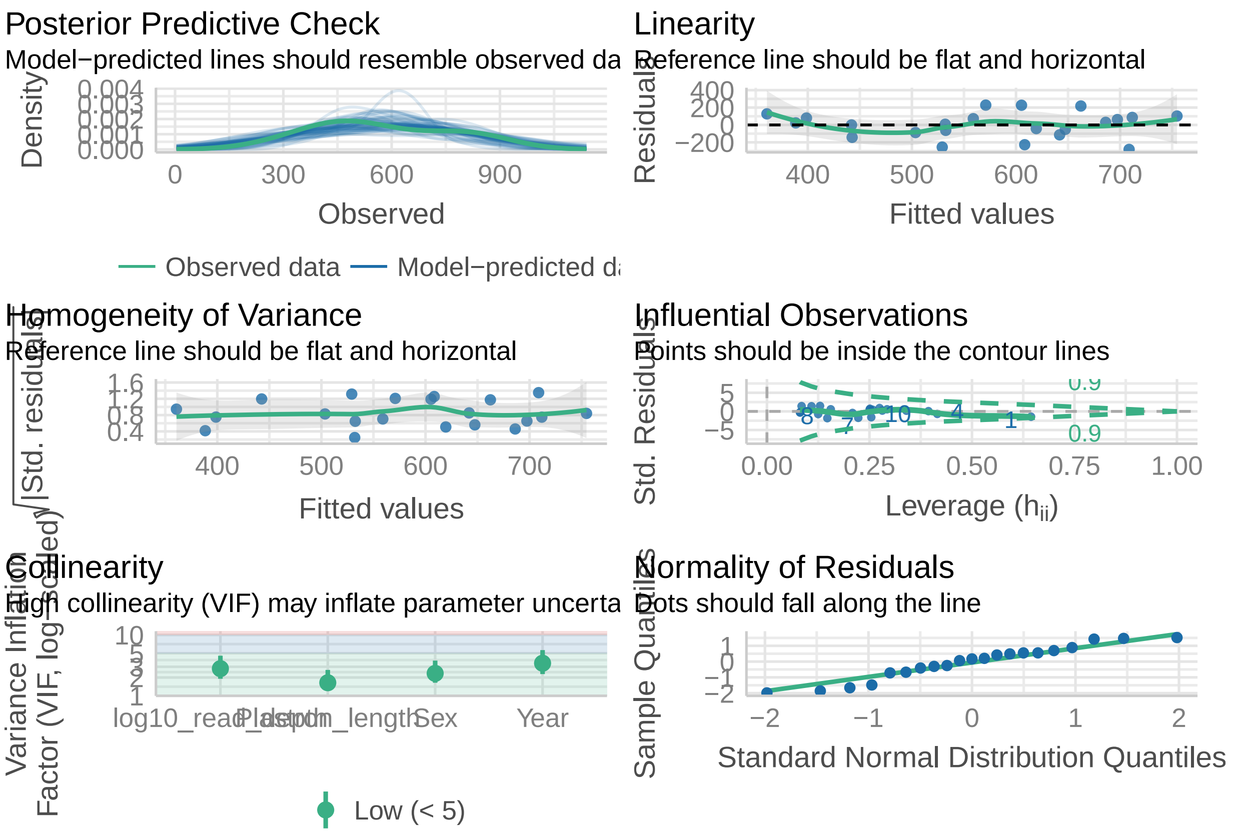

```r
plot(pl.Observed.TRSC, which = 1)
```

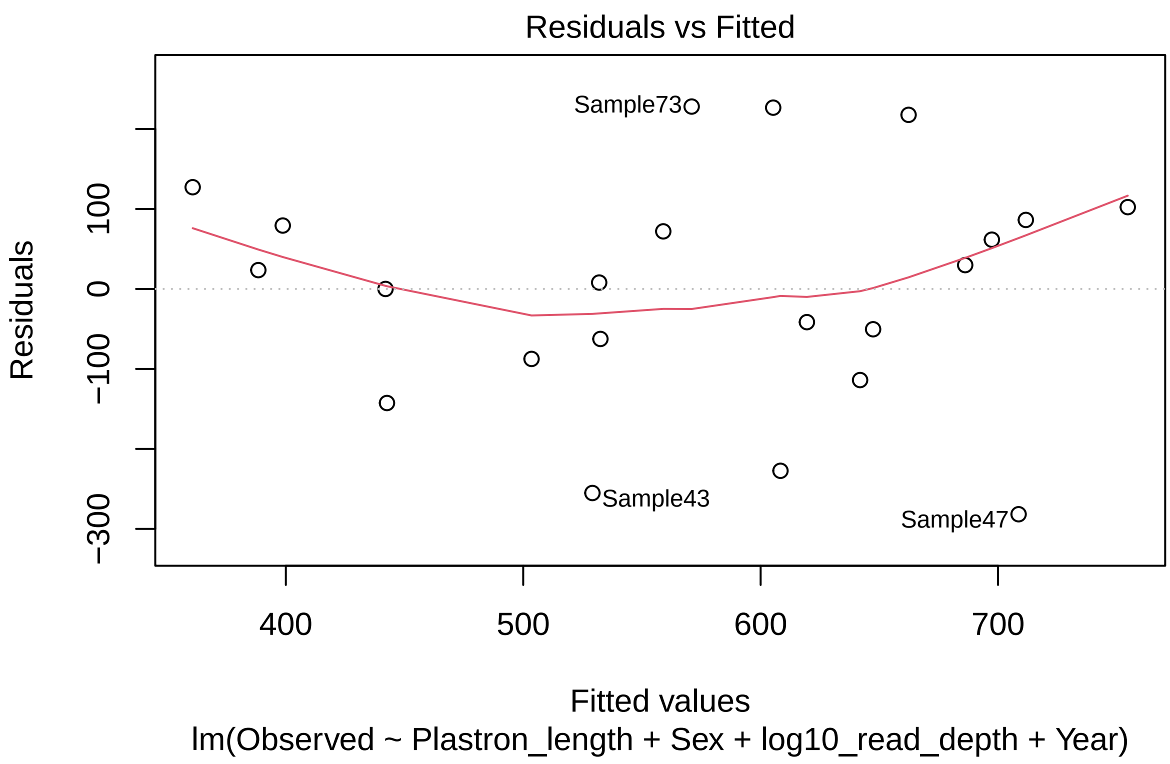


```r
plot(pl.Observed.TRSC, which = 2)
```

```
## Warning: not plotting observations with leverage one:
##   6
```

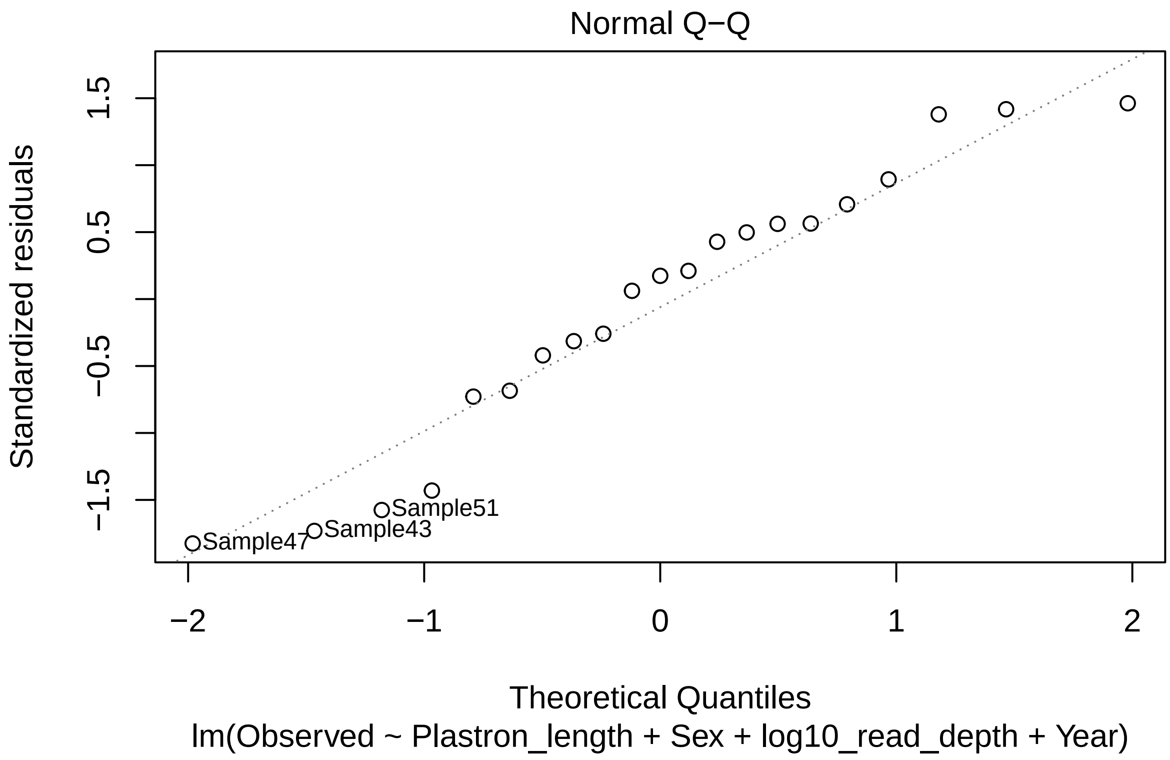


```r
#Check Faith's Phylogenetic Diversity
#check for effects in KISU
pl.PD.KISU <- lm(PD~Plastron_length+Site+Sex+log10_read_depth+Year,data=KISU)

summary(pl.PD.KISU)
```

```
##
## Call:
## lm(formula = PD ~ Plastron_length + Site + Sex + log10_read_depth +
##     Year, data = KISU)
##
## Residuals:
##     Min      1Q  Median      3Q     Max
## -9.1388 -3.6851 -0.2408  3.0385  8.8552
##
## Coefficients:
##                   Estimate Std. Error t value Pr(>|t|)
## (Intercept)      -5743.2623 10250.9421  -0.560   0.5906
## Plastron_length      0.1036     0.3395   0.305   0.7680
## SiteS4               7.5866     5.7220   1.326   0.2215
## SexM                 0.3157     5.6636   0.056   0.9569
## log10_read_depth    31.2921    16.7958   1.863   0.0995 .
## Year                 2.7750     5.0875   0.545   0.6003
## ---
## Signif. codes:  0 '***' 0.001 '**' 0.01 '*' 0.05 '.' 0.1 ' ' 1
##
## Residual standard error: 6.83 on 8 degrees of freedom
## Multiple R-squared:  0.551,  Adjusted R-squared:  0.2703
## F-statistic: 1.963 on 5 and 8 DF,  p-value: 0.1891
```

```r
confint(pl.PD.KISU)
```

```
##                           2.5 %       97.5 %
## (Intercept)       -2.938198e+04 1.789545e+04
## Plastron_length   -6.791921e-01 8.863638e-01
## SiteS4            -5.608227e+00 2.078149e+01
## SexM              -1.274448e+01 1.337591e+01
## log10_read_depth  -7.439062e+00 7.002333e+01
## Year              -8.956845e+00 1.450693e+01
```

```r
check_model(pl.PD.KISU)
```

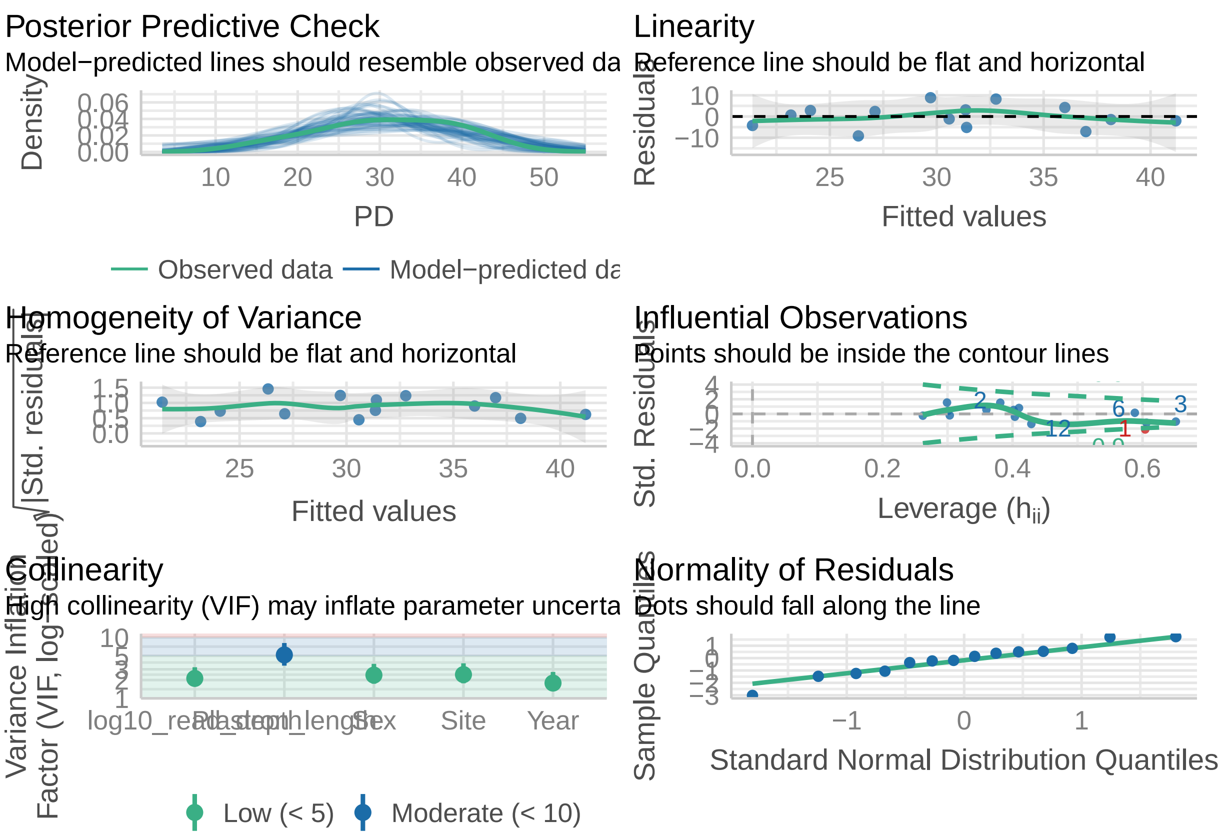


```r
plot(pl.PD.KISU, which = 1)
```

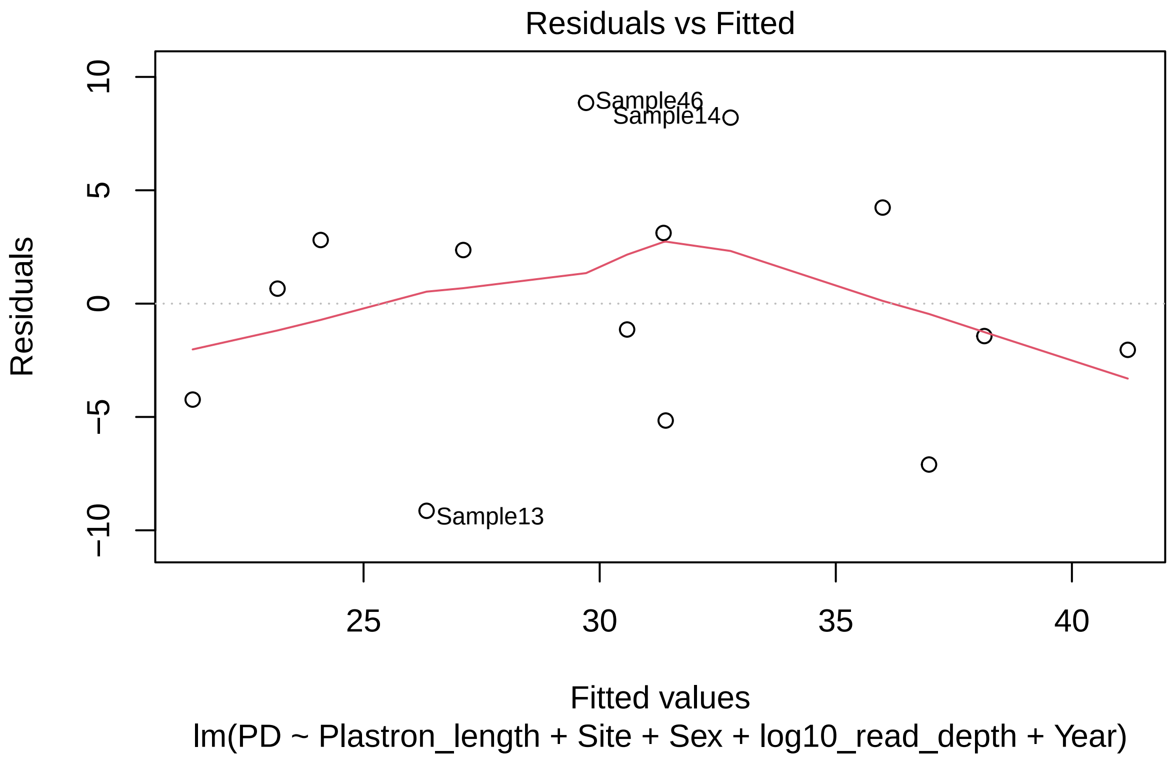


```r
plot(pl.PD.KISU, which = 2)
```

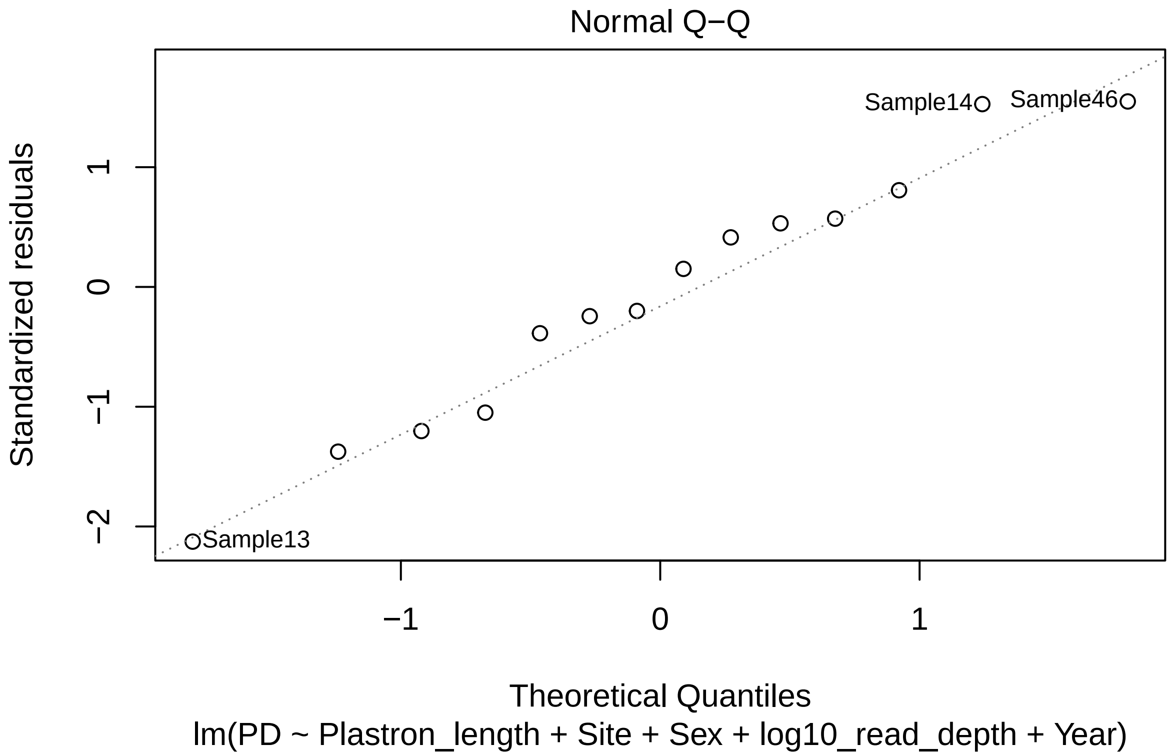


```r
#check for effects in PSCO
#took out variable 'sex' from PSCO model for PD, as sex and plastron_length were highly collinear
pl.PD.PSCO <- lm(PD~Plastron_length+log10_read_depth+Year,data=PSCO)
```

```r
summary(pl.PD.PSCO)
```

```
##
## Call:
## lm(formula = PD ~ Plastron_length + log10_read_depth + Year,
##     data = PSCO)
##
## Residuals:
##      Min       1Q   Median       3Q      Max
## -12.7048  -2.4737   0.7354   2.9064   9.6283
##
## Coefficients:
##                    Estimate Std. Error t value Pr(>|t|)
## (Intercept)       1.323e+04  1.057e+04   1.251    0.246
## Plastron_length  -2.554e-02  4.479e-02  -0.570    0.584
## log10_read_depth  1.756e+01  9.629e+00   1.823    0.106
## Year             -6.568e+00  5.225e+00  -1.257    0.244
##
## Residual standard error: 6.535 on 8 degrees of freedom
## Multiple R-squared:  0.4462, Adjusted R-squared:  0.2385
## F-statistic: 2.148 on 3 and 8 DF,  p-value: 0.1723
```

```r
confint(pl.PD.PSCO)
```

```
##                          2.5 %       97.5 %
## (Intercept)      -1.115048e+04 3.761739e+04
## Plastron_length  -1.288216e-01 7.774998e-02
## log10_read_depth -4.647445e+00 3.975979e+01
## Year             -1.861618e+01 5.480492e+00
```

```r
check_model(pl.PD.PSCO)
```

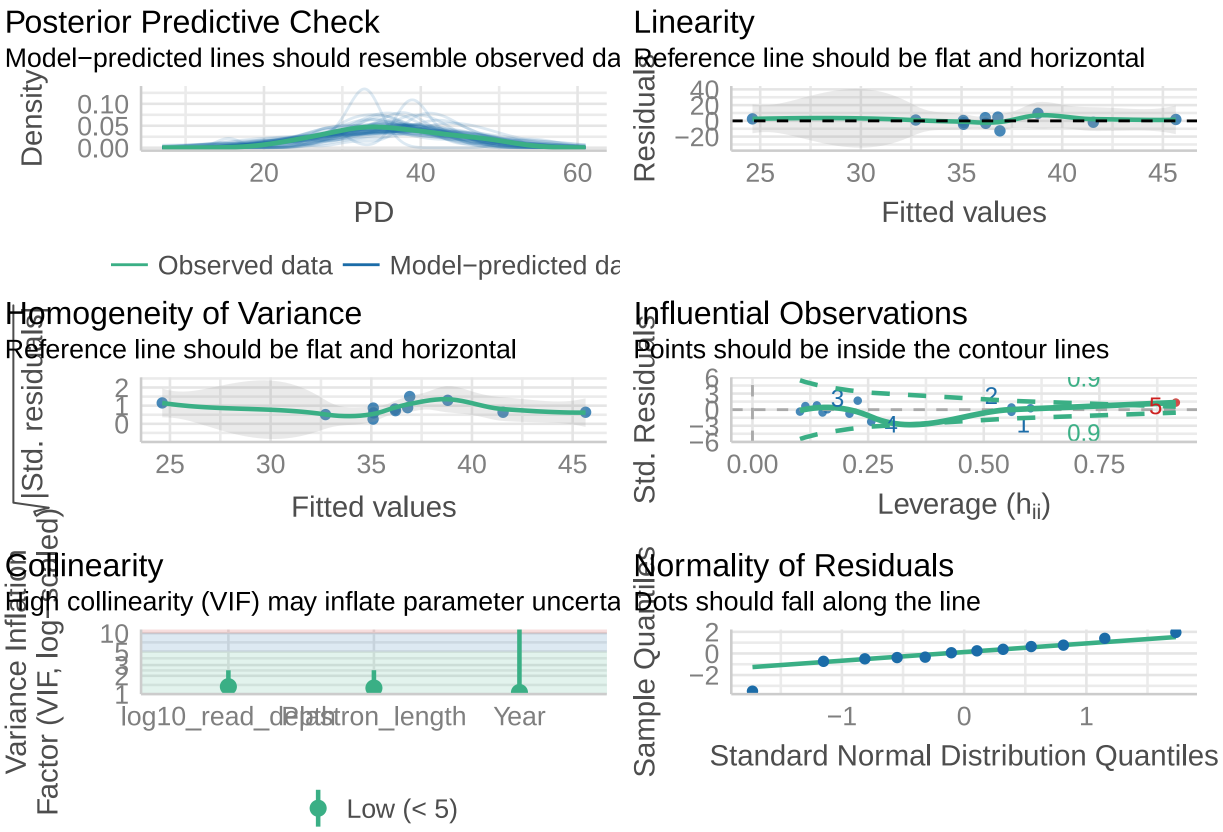


```r
plot(pl.PD.PSCO, which = 1)
```

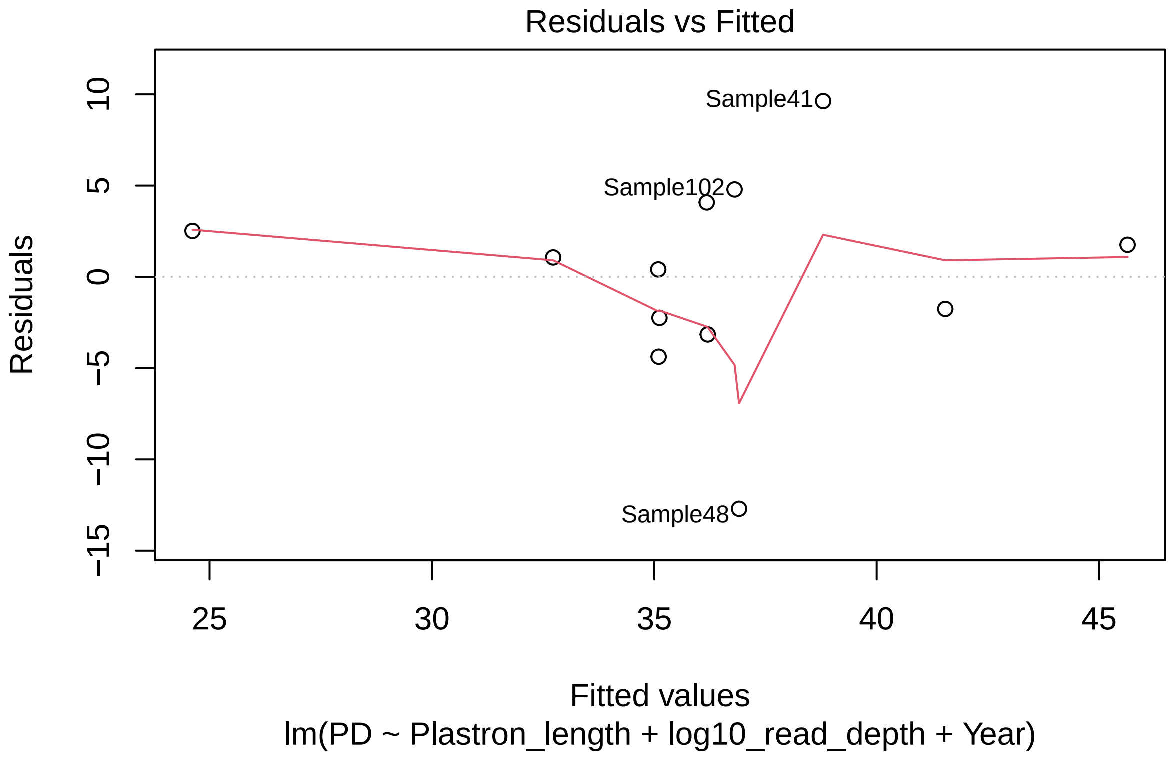

```r
plot(pl.PD.PSCO, which = 2)
```

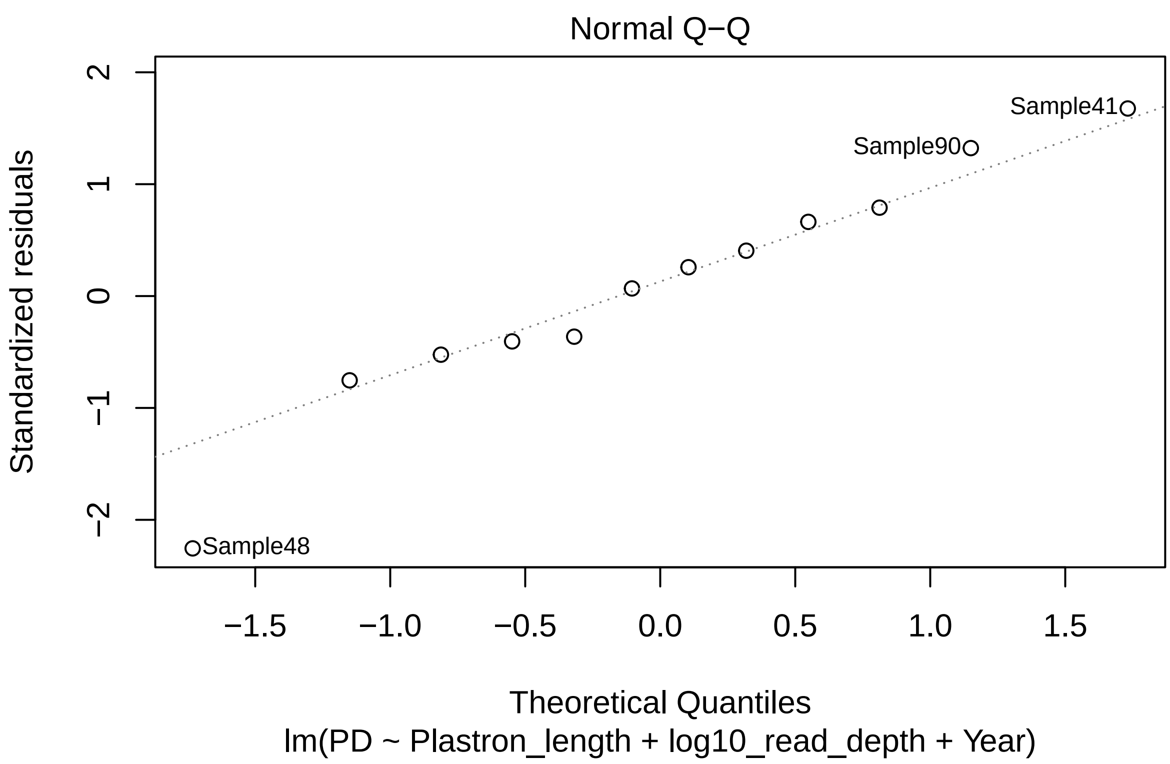


```r
#check for effects in STCA
pl.PD.STCA <- lm(PD~Plastron_length+Sex+log10_read_depth+Year,data=STCA)

summary(pl.PD.STCA)
```

```
##
## Call:
## lm(formula = PD ~ Plastron_length + Sex + log10_read_depth +
##     Year, data = STCA)
##
## Residuals:
##   Sample55   Sample62   Sample63   Sample64   Sample69   Sample70   Sample71
##  2.960e+00 -2.783e+00  5.813e+00 -1.253e+01  1.443e-15  5.044e+00  6.108e+00
##   Sample72
## -4.608e+00
##
## Coefficients: (1 not defined because of singularities)
##                   Estimate Std. Error t value Pr(>|t|)
## (Intercept)      -704.26921  412.34118  -1.708    0.163
## Plastron_length    -0.05036    0.32641  -0.154    0.885
## SexM               -9.92321   13.09754  -0.758    0.491
## log10_read_depth  155.25547   88.39243   1.756    0.154
## Year                     NA         NA      NA       NA
##
## Residual standard error: 8.535 on 4 degrees of freedom
## Multiple R-squared:  0.495,  Adjusted R-squared:  0.1162
## F-statistic: 1.307 on 3 and 4 DF,  p-value: 0.3879
```

```r
confint(pl.PD.STCA)
```

```
##                             2.5 %      97.5 %
## (Intercept)        -1849.1118445 440.5734328
## Plastron_length       -0.9566084   0.8558886
## SexM                 -46.2878065  26.4413795
## log10_read_depth     -90.1612563 400.6721913
## Year                          NA          NA
```

```r
check_model(pl.PD.STCA)
```

```
## Model matrix is rank deficient. VIFs may not be sensible.
```

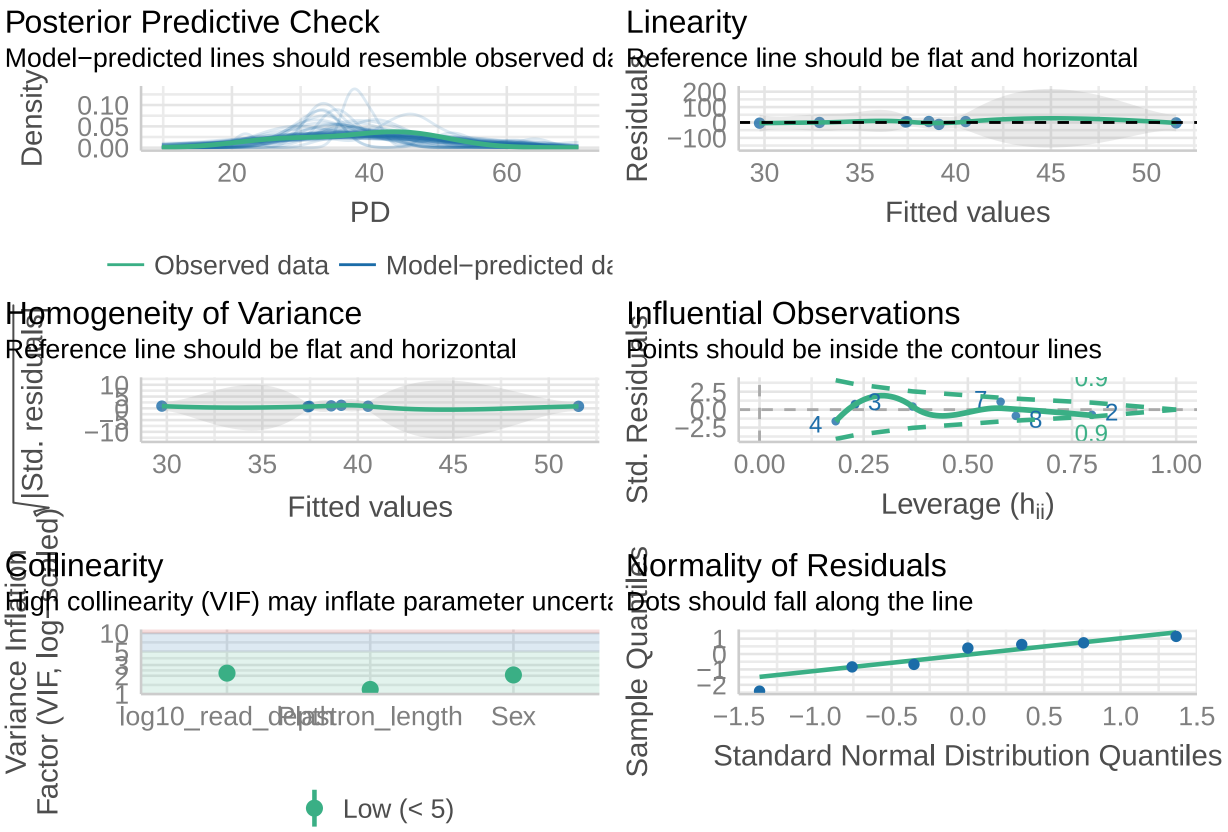


```r
plot(pl.PD.STCA, which = 1)
```

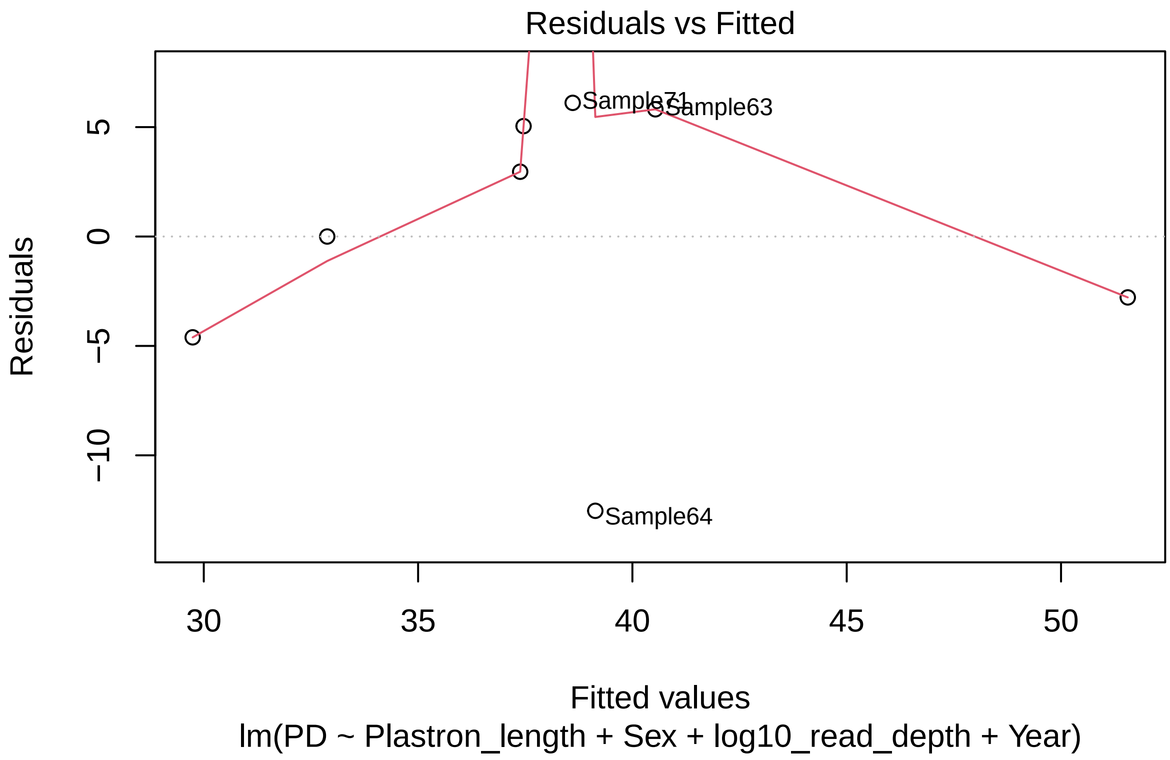


```r
plot(pl.PD.STCA, which = 2)
```

```
## Warning: not plotting observations with leverage one:
##   5
```

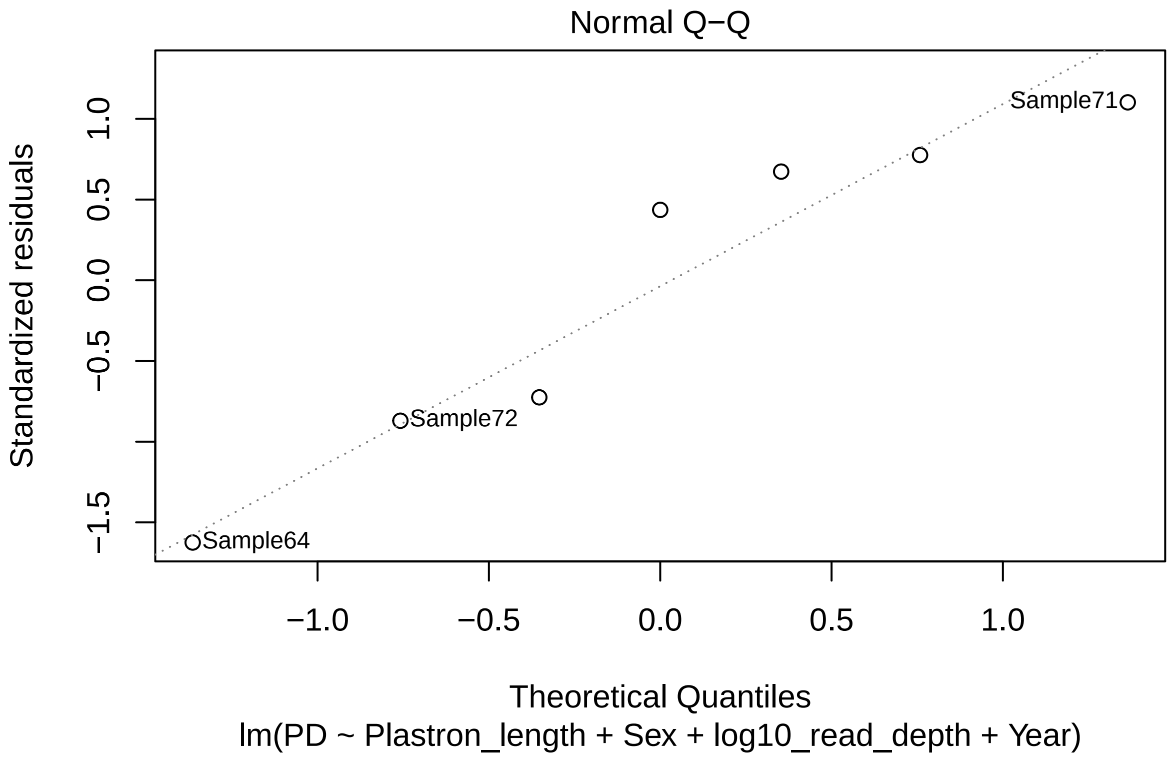

```r
#check for effects in STOD
pl.PD.STOD <- lm(PD~Plastron_length+Sex+log10_read_depth+Year,data=STOD)

summary(pl.PD.STOD)
```

```
##
## Call:
## lm(formula = PD ~ Plastron_length + Sex + log10_read_depth +
##     Year, data = STOD)
##
## Residuals:
##     Min      1Q  Median      3Q     Max
## -13.064  -3.568  -1.148   5.584  11.220
##
## Coefficients:
##                   Estimate Std. Error t value Pr(>|t|)
## (Intercept)      -2.317e+03  8.805e+03  -0.263  0.79525
## Plastron_length  -5.306e-02  2.379e-01  -0.223  0.82586
## SexJ              2.994e+00  1.036e+01   0.289  0.77580
## SexM              2.974e+00  4.021e+00   0.740  0.46854
## log10_read_depth  5.989e+01  1.735e+01   3.452  0.00267 **
## Year              1.025e+00  4.385e+00   0.234  0.81759
## ---
## Signif. codes:  0 '***' 0.001 '**' 0.01 '*' 0.05 '.' 0.1 ' ' 1
##
## Residual standard error: 7.2 on 19 degrees of freedom
## Multiple R-squared:  0.6517, Adjusted R-squared:  0.5601
## F-statistic: 7.111 on 5 and 19 DF,  p-value: 0.0006688
```

```r
confint(pl.PD.STOD)
```

```
##                          2.5 %       97.5 %
## (Intercept)      -2.074562e+04 1.611139e+04
## Plastron_length  -5.509627e-01 4.448328e-01
## SexJ             -1.869910e+01 2.468736e+01
## SexM             -5.442000e+00 1.139059e+01
## log10_read_depth  2.357640e+01 9.621021e+01
## Year             -8.152247e+00 1.020318e+01
```

```r
check_model(pl.PD.STOD)
```

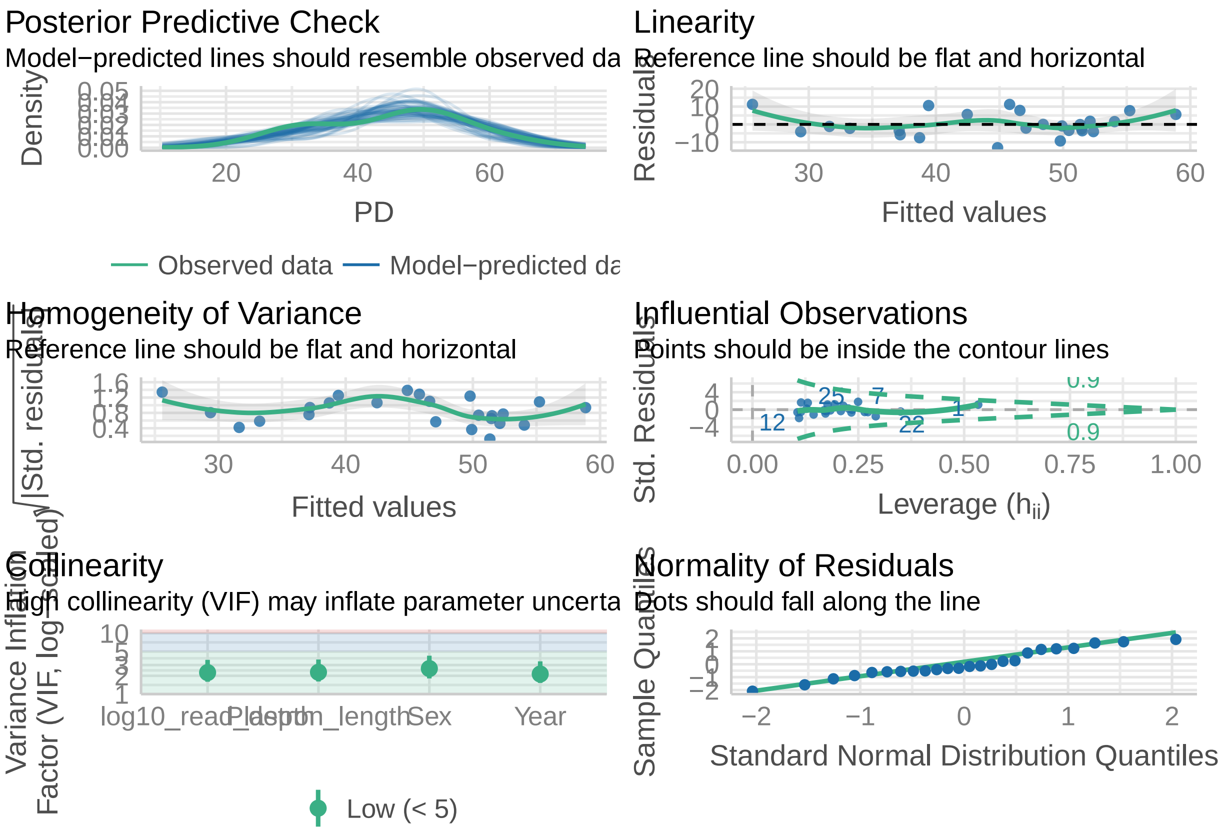


```r
plot(pl.PD.STOD, which = 1)
```

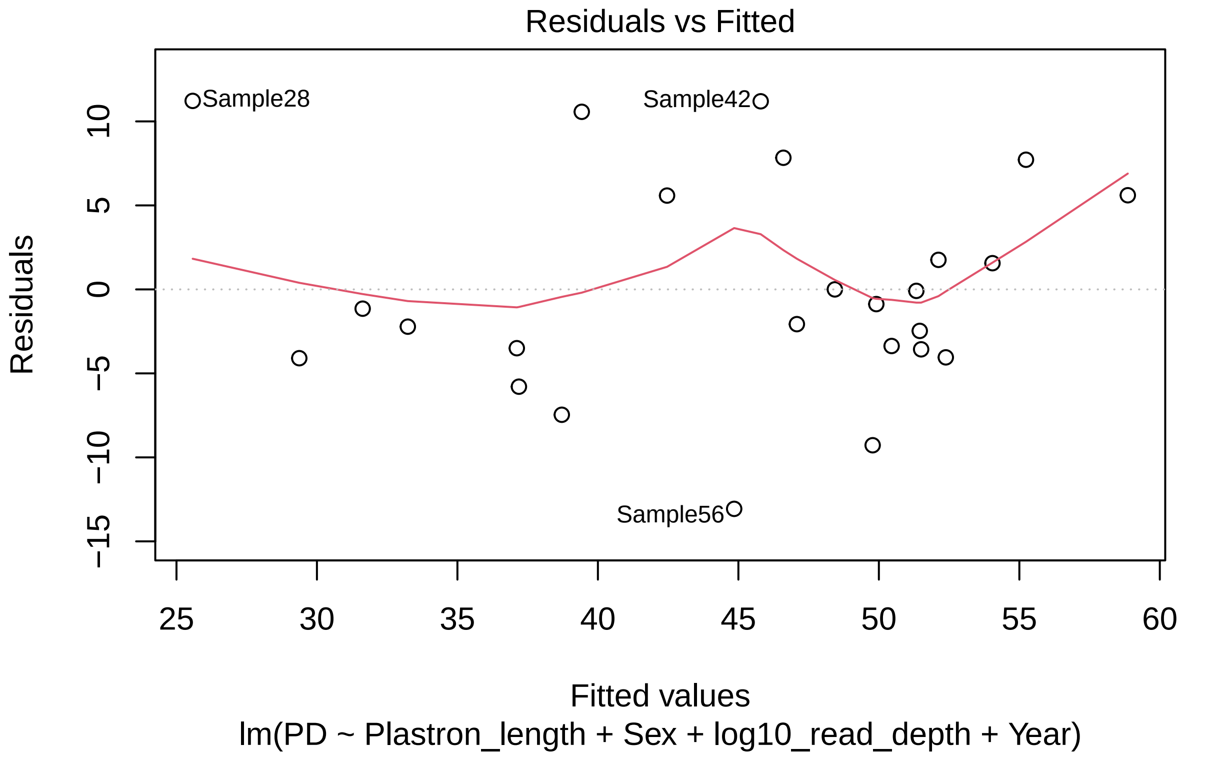

```r
plot(pl.PD.STOD, which = 2)
```

```
## Warning: not plotting observations with leverage one:
##   3
```

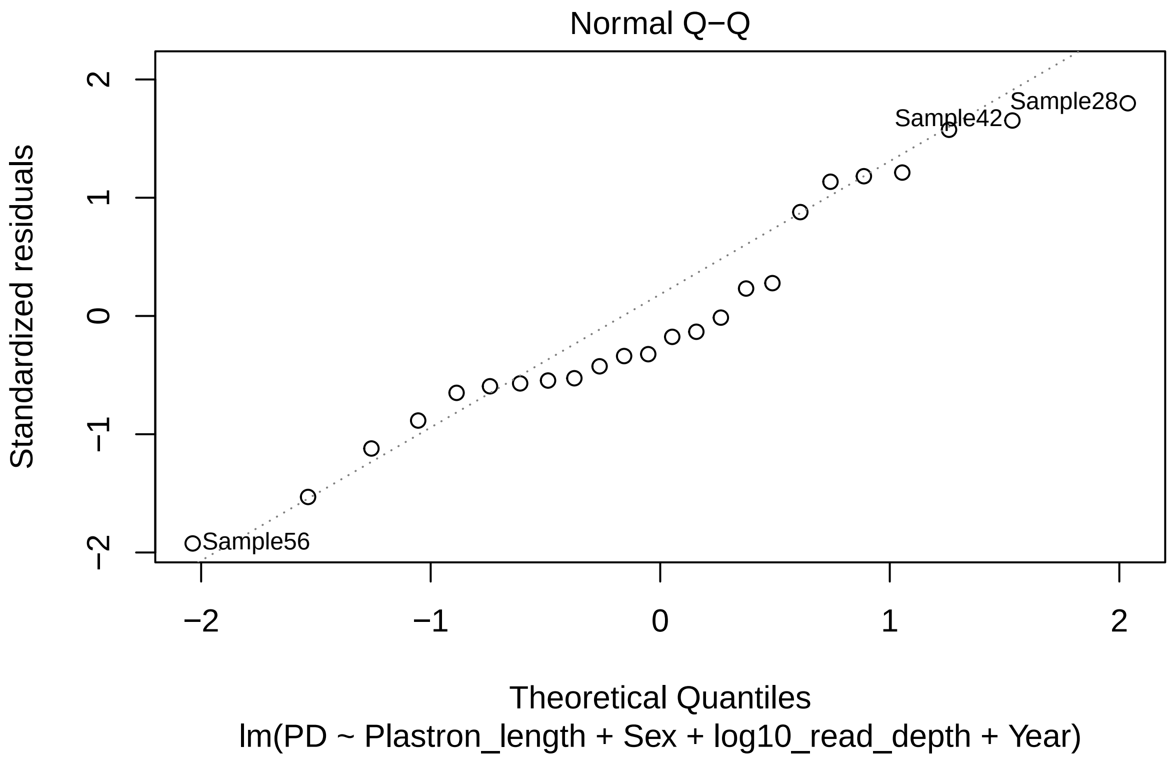


```r
#check for effects in TRSC
pl.PD.TRSC <- lm(PD~Plastron_length+Sex+log10_read_depth+Year,data=TRSC)

summary(pl.PD.TRSC)
```

```
##
## Call:
## lm(formula = PD ~ Plastron_length + Sex + log10_read_depth +
##     Year, data = TRSC)
##
## Residuals:
##      Min       1Q   Median       3Q      Max
## -12.1802  -2.6579   0.9087   3.7149  12.3023
##
## Coefficients:
##                    Estimate Std. Error t value Pr(>|t|)
## (Intercept)      -2.575e+04  1.251e+04  -2.059   0.0562 .
## Plastron_length   7.800e-02  7.106e-02   1.098   0.2886
## SexJ              4.481e+00  1.012e+01   0.443   0.6639
## SexM             -1.429e+00  3.580e+00  -0.399   0.6951
## log10_read_depth -1.997e+01  2.532e+01  -0.788   0.4419
## Year              1.280e+01  6.236e+00   2.053   0.0568 .
## ---
```

```
## Signif. codes:  0 '***' 0.001 '**' 0.01 '*' 0.05 '.' 0.1 ' ' 1
##
## Residual standard error: 7.033 on 16 degrees of freedom
## Multiple R-squared:  0.3891, Adjusted R-squared:  0.1982
## F-statistic: 2.039 on 5 and 16 DF,  p-value: 0.1275
```

```r
confint(pl.PD.TRSC)
```

```
##                           2.5 %      97.5 %
## (Intercept)       -5.227048e+04 767.4114615
## Plastron_length   -7.265244e-02   0.2286435
## SexJ              -1.697955e+01  25.9421310
## SexM              -9.017764e+00   6.1606969
## log10_read_depth  -7.364869e+01  33.7158267
## Year              -4.181013e-01  26.0226581
```

```r
check_model(pl.PD.TRSC)
```

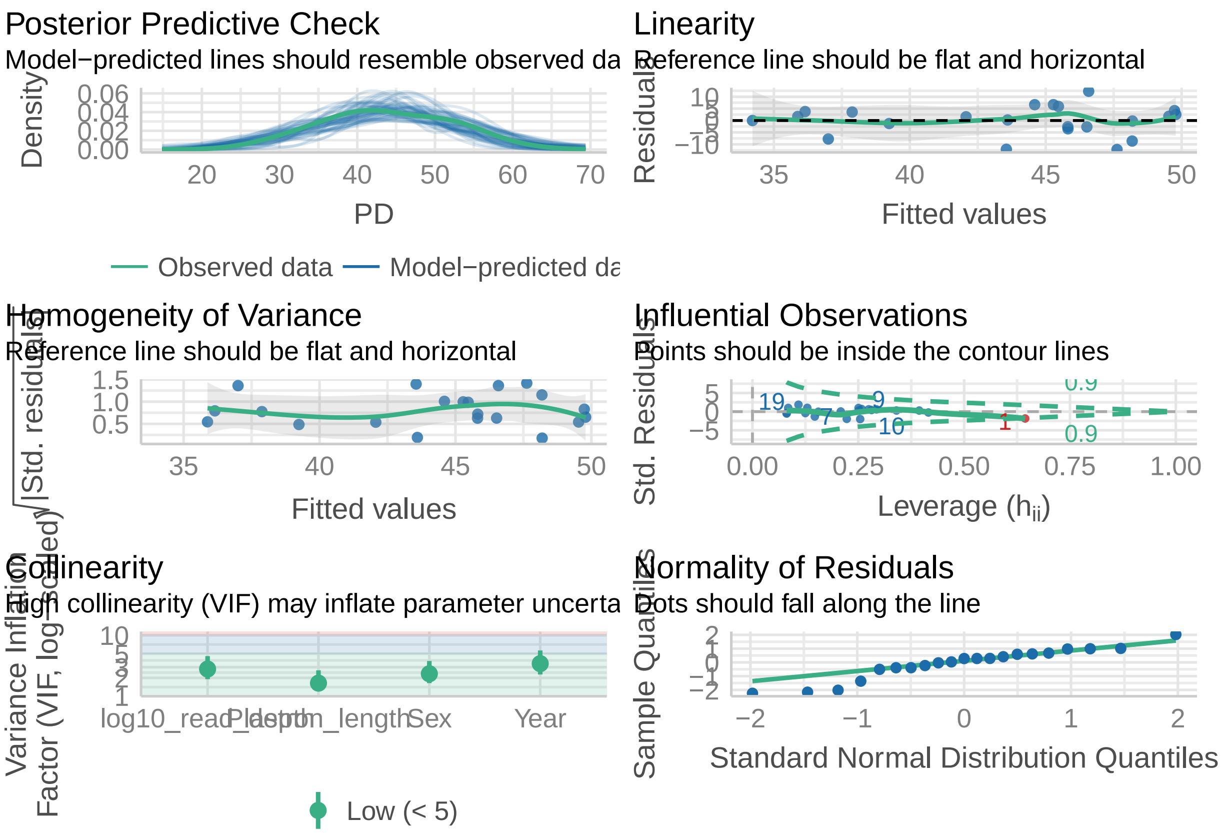

```r
plot(pl.PD.TRSC, which = 1)
```

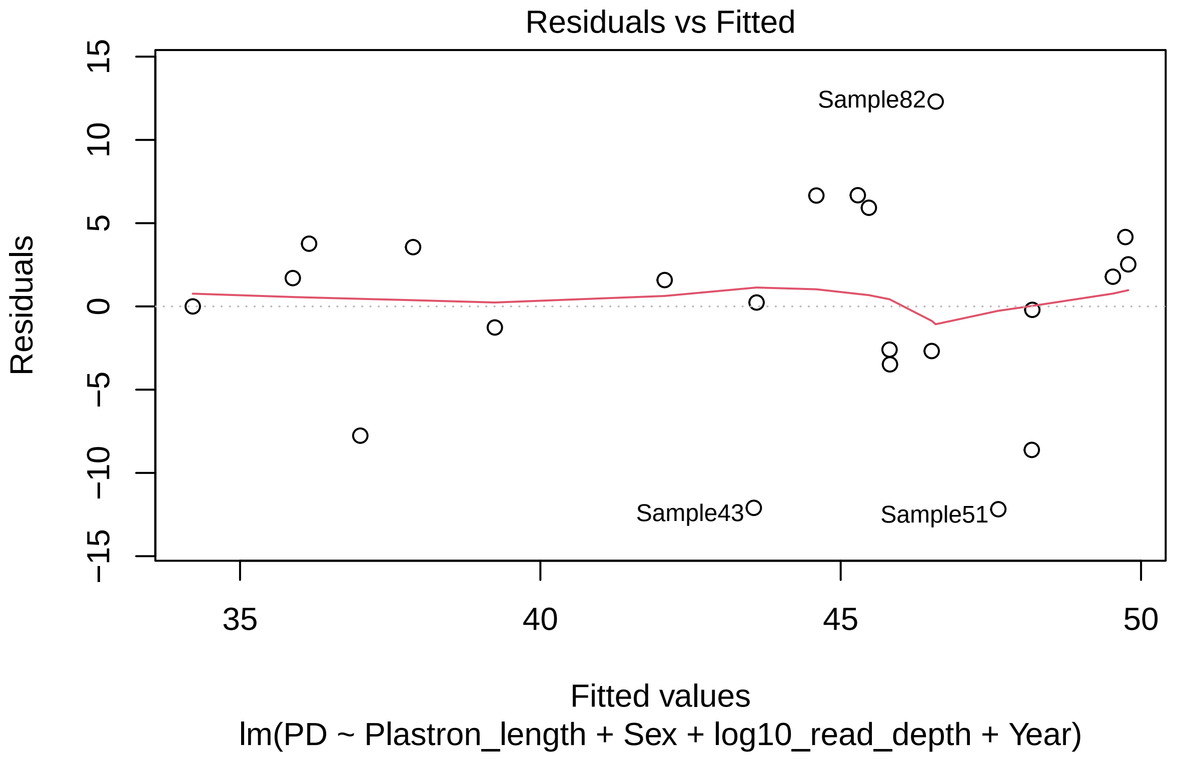


```r
plot(pl.PD.TRSC, which = 2)
```

```
## Warning: not plotting observations with leverage one:
##   6
```

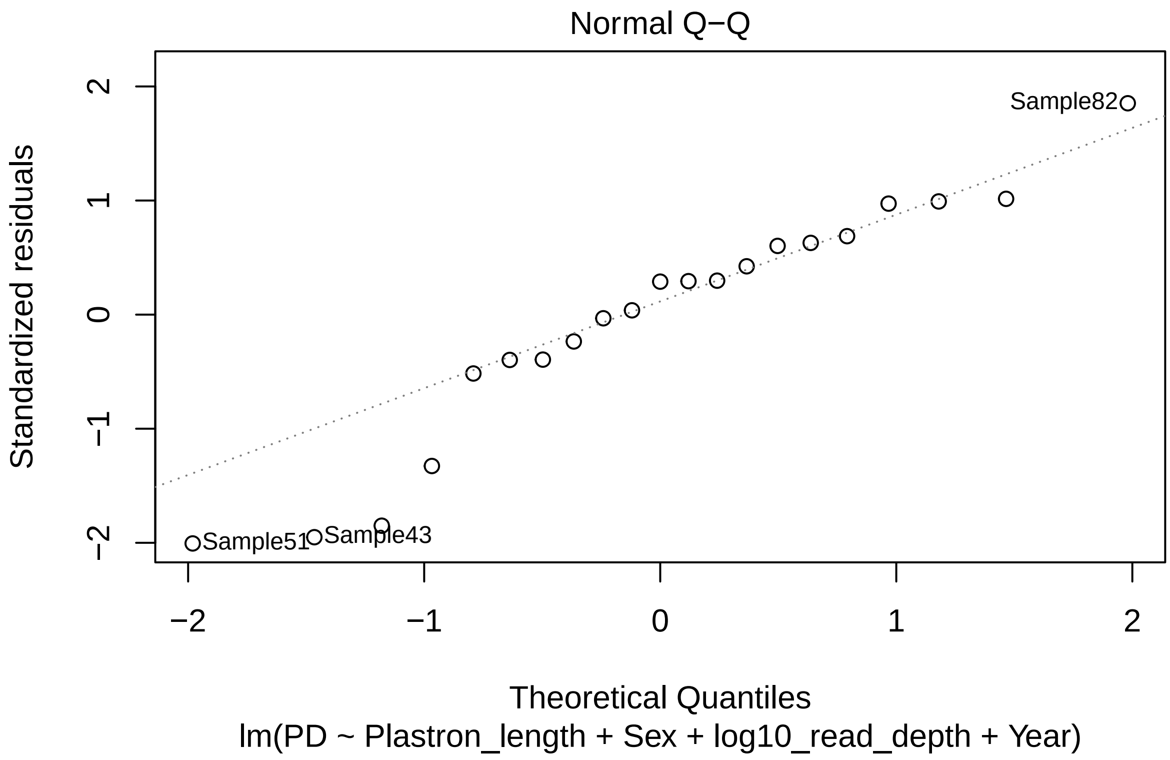

Generate heat maps for different taxonomic levels:

The loop commands here will be used to generate taxonomy-based heat maps for turtle species/collection site and collection site/turtle species combination, at the taxonomic levels of ASV, genus, family, order, class and phylum

```
phylo.heat <- function(phyloseq.object = phylo, data, ID.col = "sample.ID", order1 = NULL, order2 = NULL

  phylo.temp <- prune_samples(x = phyloseq.object,samples = data[,which(colnames(data) == ID.col)]) #su
  phylo.temp  = filter_taxa(phylo.temp , function(x) mean(x) > 0, TRUE)

  data$order <- paste(data[,which(colnames(data) == order1)], #make ordering column
                      data[,which(colnames(data) == order2)],
                      data[,which(colnames(data) == order3)],
                      data[,which(colnames(data) == order4)])

  data.i <- data.i[match(sample_data(phylo.temp)$sample.ID,data.i$sample.ID),]

  sample_data(phylo.temp)$grouping <- data$order

  data <- data[order(data$order),] # order data

  plot <- plot_heatmap(phylo.temp,sample.order = data[,which(colnames(data) == ID.col)],sample.label =


  print(plot)
}
```

```r
phylo.heat.loop <- function(phyloseq.object.list = phylo.list, data, ID.col = "sample.ID", order1 = NULL

  for(i in 1:length(phyloseq.object.list )){

    phyloseq.object <- phyloseq.object.list[[i]]
    taxa.label <- taxa.label.list[[i]]

    data.i <- data

    phylo.temp <- prune_samples(x = phyloseq.object,samples = data.i[,which(colnames(data.i) == ID.col)]
    phylo.temp  = filter_taxa(phylo.temp , function(x) mean(x) > 0, TRUE)

    data.i$order <- paste(data.i[,which(colnames(data.i) == order1)], #make ordering column
                          data.i[,which(colnames(data.i) == order2)],
                          data.i[,which(colnames(data.i) == order3)],
                          data.i[,which(colnames(data.i) == order4)])


    data.i <- data.i[match(sample_data(phylo.temp)$sample.ID,data.i$sample.ID),]

    sample_data(phylo.temp)$grouping <- data.i$order

    data.i <- data.i[order(data.i$order),] # order data.i

    plot <- plot_heatmap(phylo.temp,sample.order = data.i[,which(colnames(data.i) == ID.col)],sample.lab


    print(plot)}

}
```

Here taxonomic heat maps are generated for turtle species/collection site and collection site/turtle species combinations, across the following taxonomic levels: ASV, genus, family, order, class and phylum.

```r
phylo.heat.loop(phylo.list, data = carapace , ID.col = "sample.ID", order1 = "Species", order2="Site",t
```

```
## Warning: Transformation introduced infinite values in discrete y-axis
```

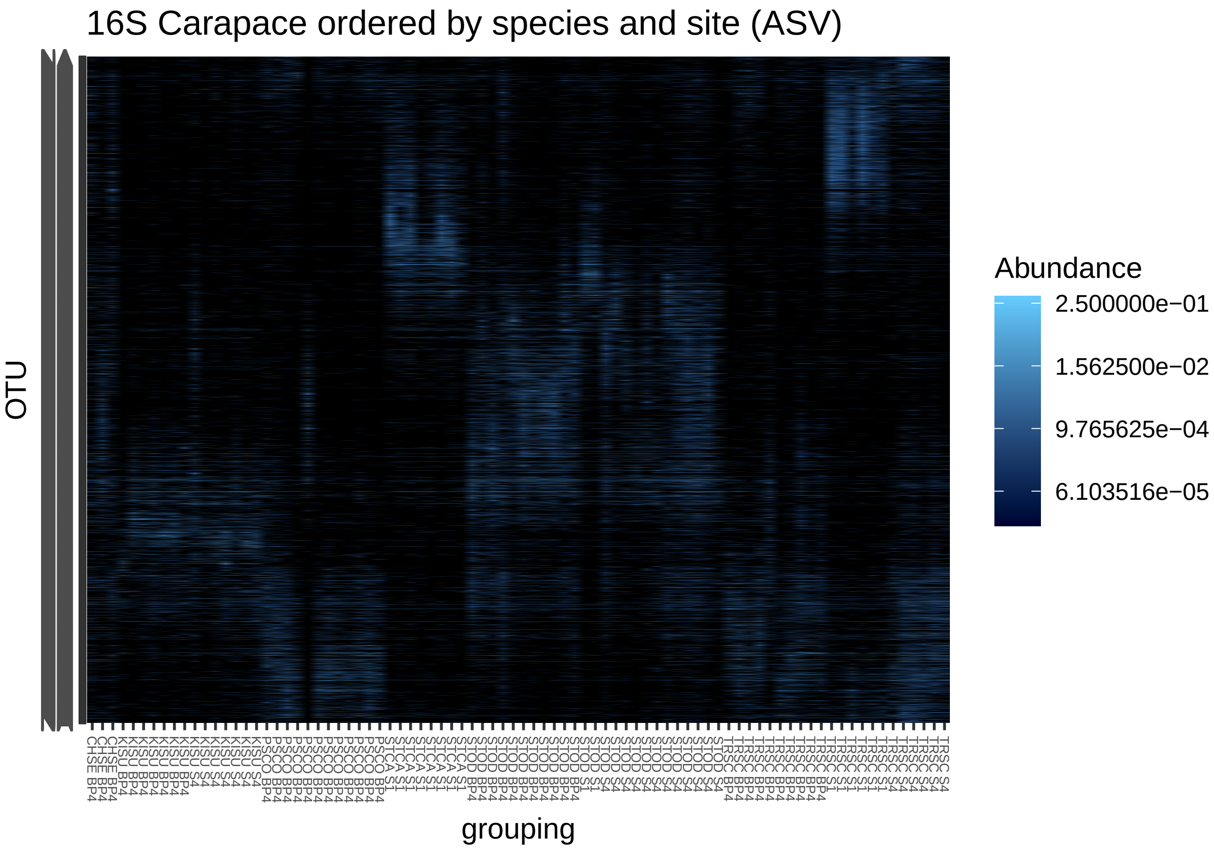


```
## Warning: Transformation introduced infinite values in discrete y-axis
```

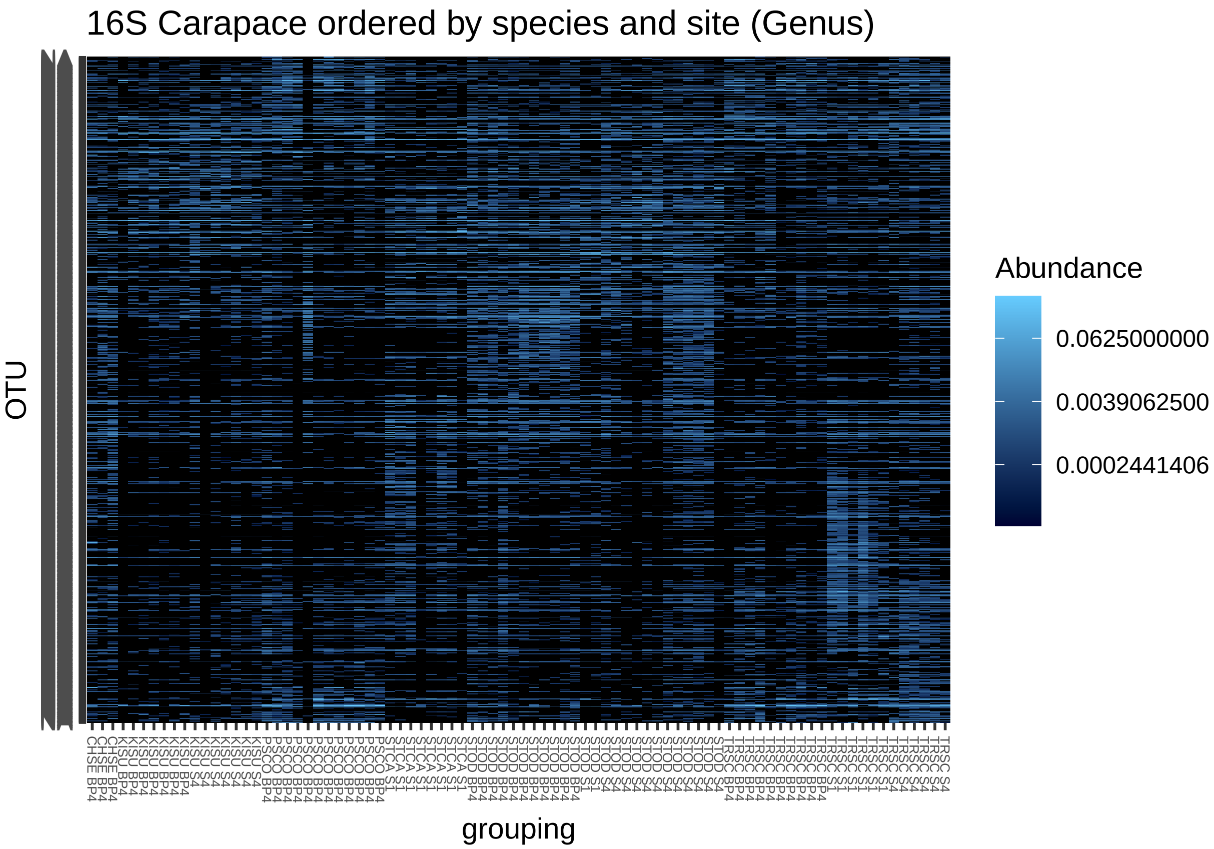

Warning: Transformation introduced infinite values in discrete y-axis

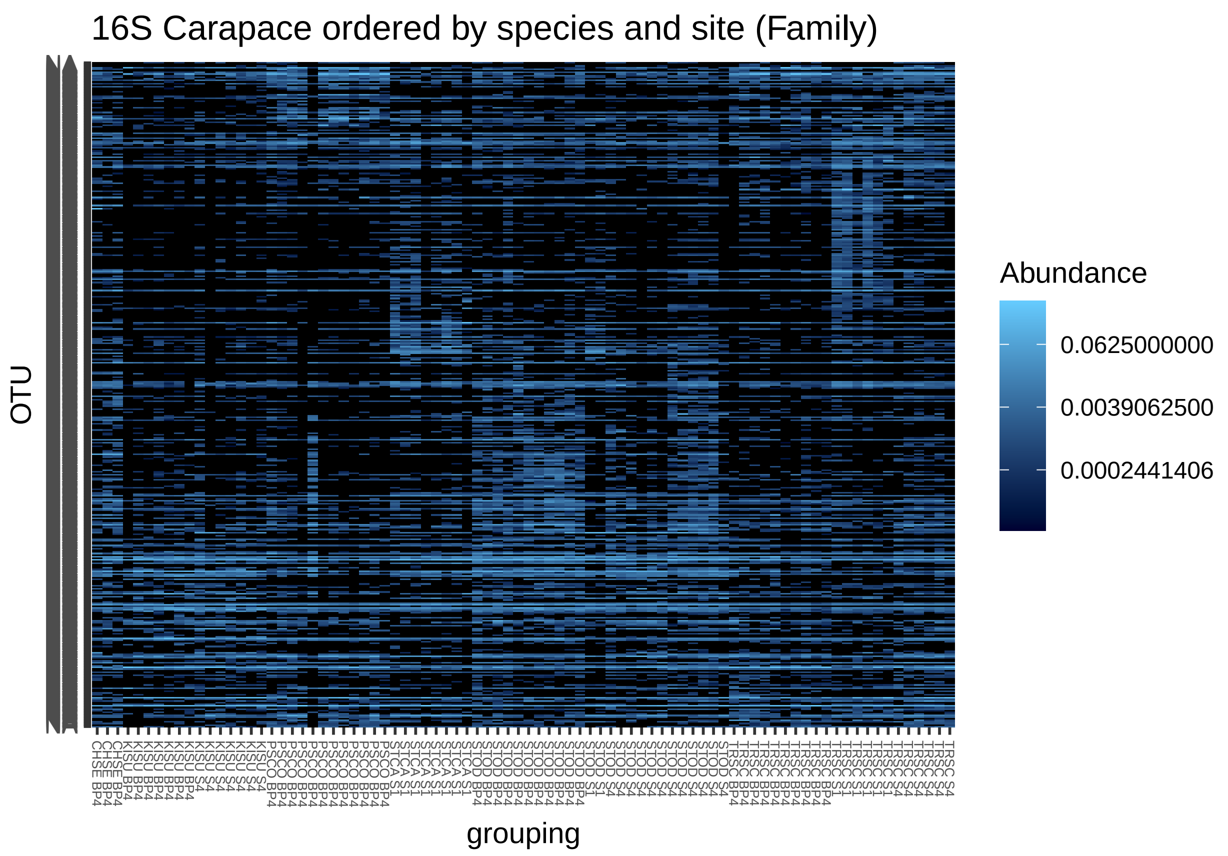

Warning: Transformation introduced infinite values in discrete y-axis

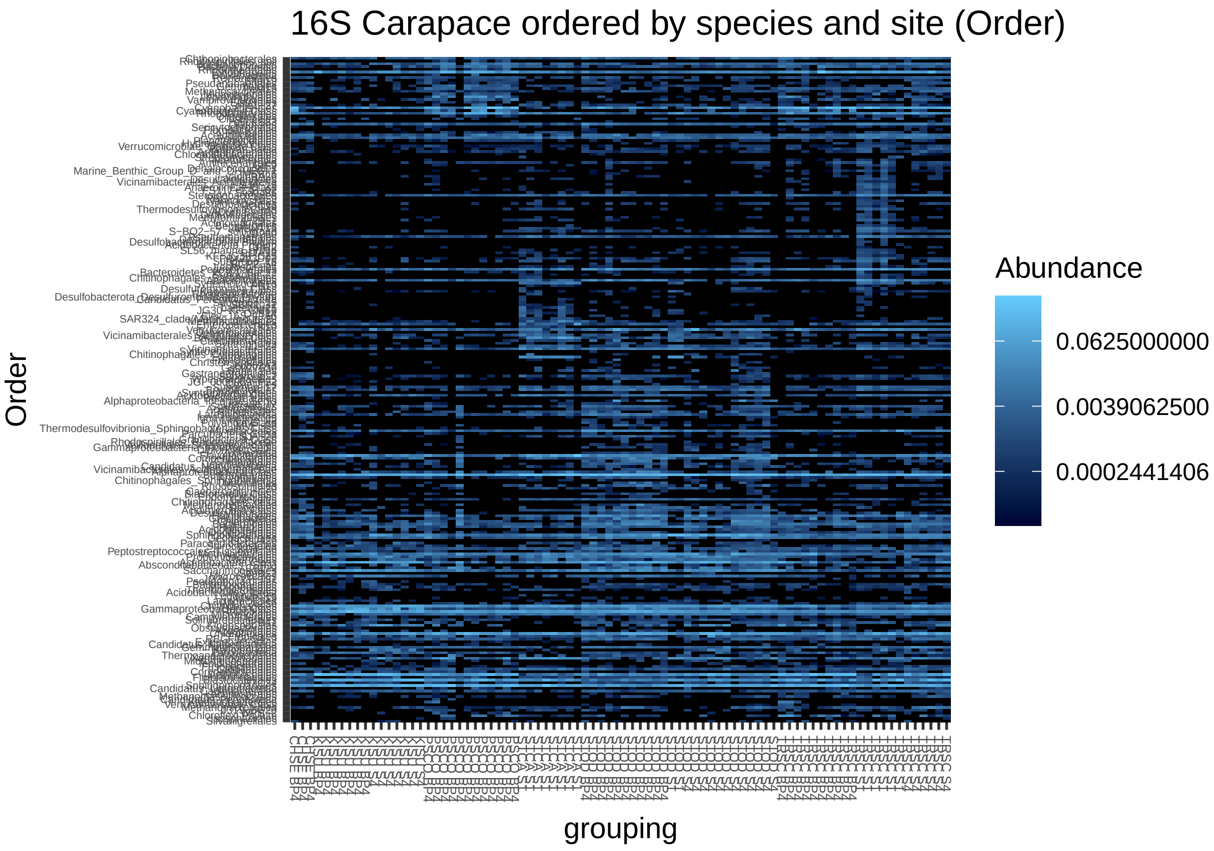


```
## Warning: Transformation introduced infinite values in discrete y-axis
```

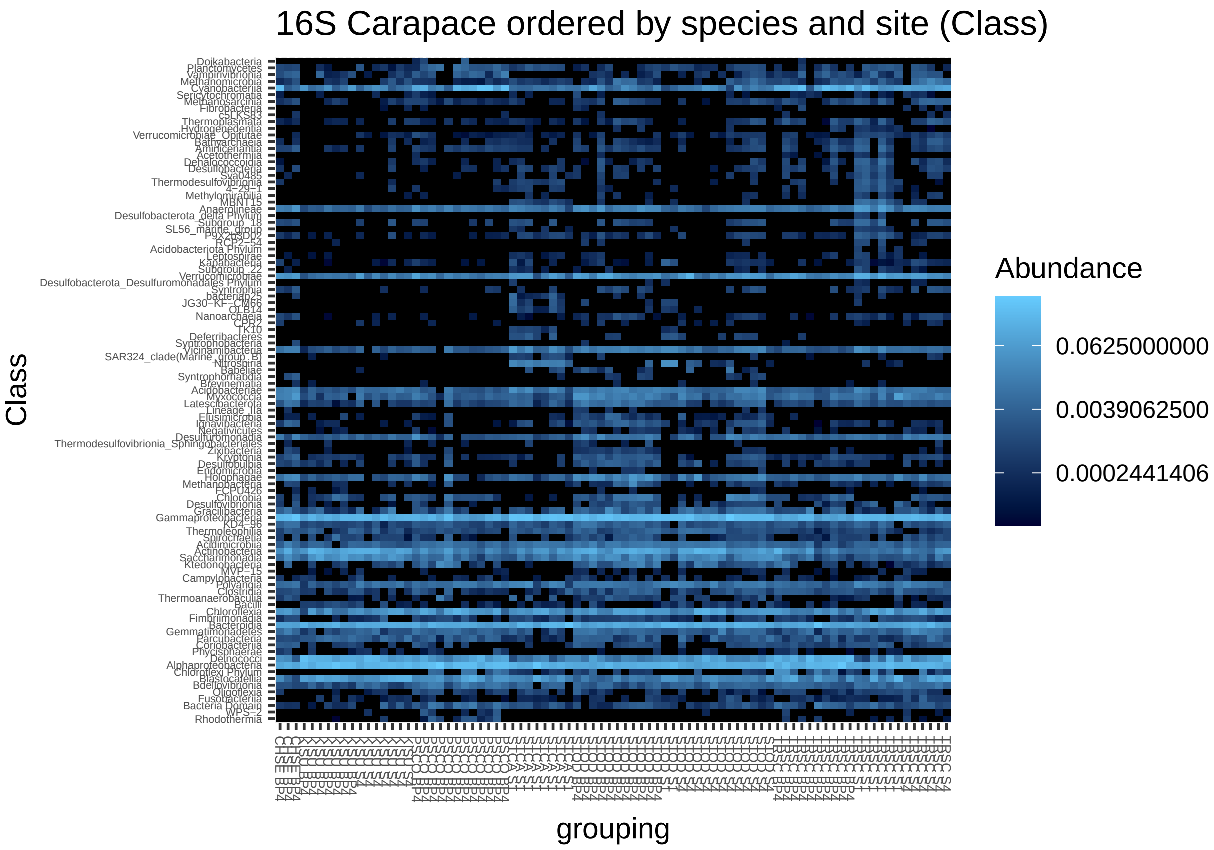


```
## Warning: Transformation introduced infinite values in discrete y-axis
```

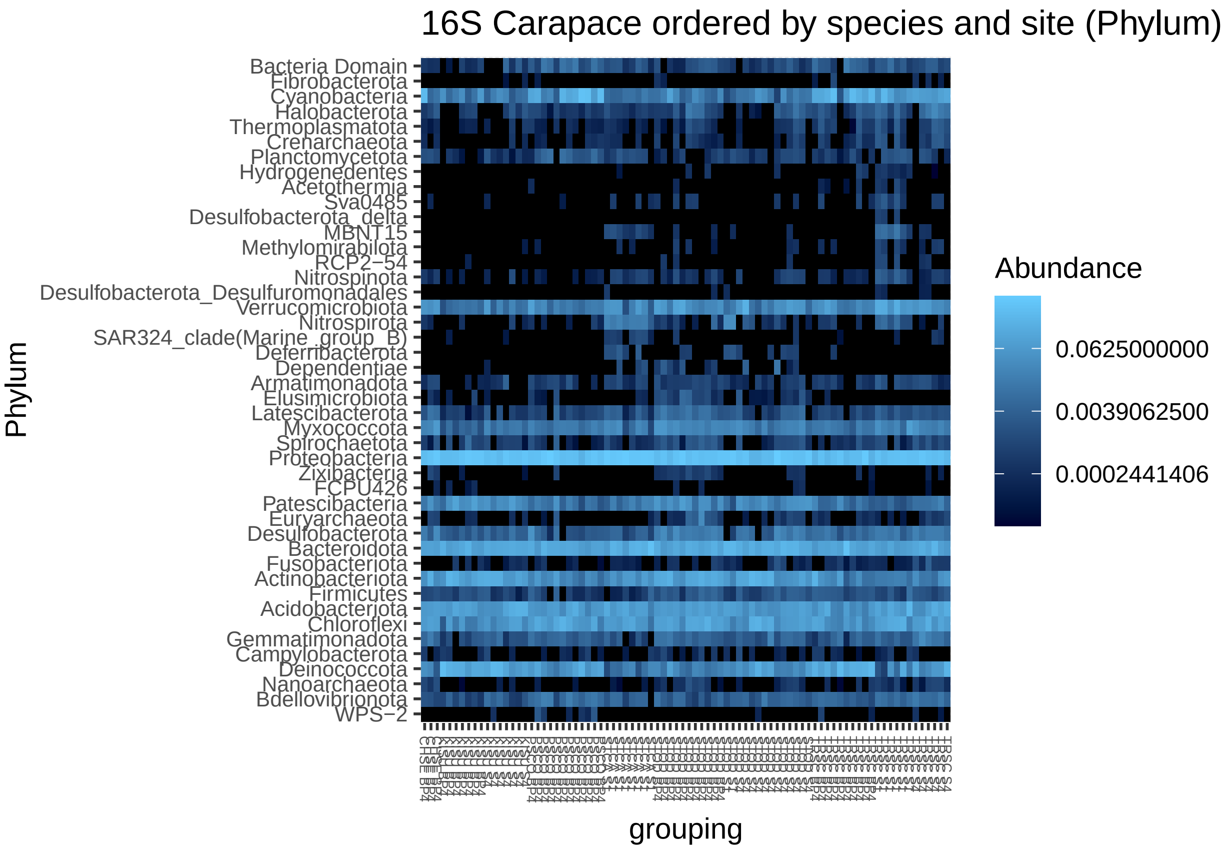


```
phylo.heat.loop(phylo.list, data = carapace , ID.col = "sample.ID", order1 = "Site", order2="Species",t
```

```
## Warning: Transformation introduced infinite values in discrete y-axis
```

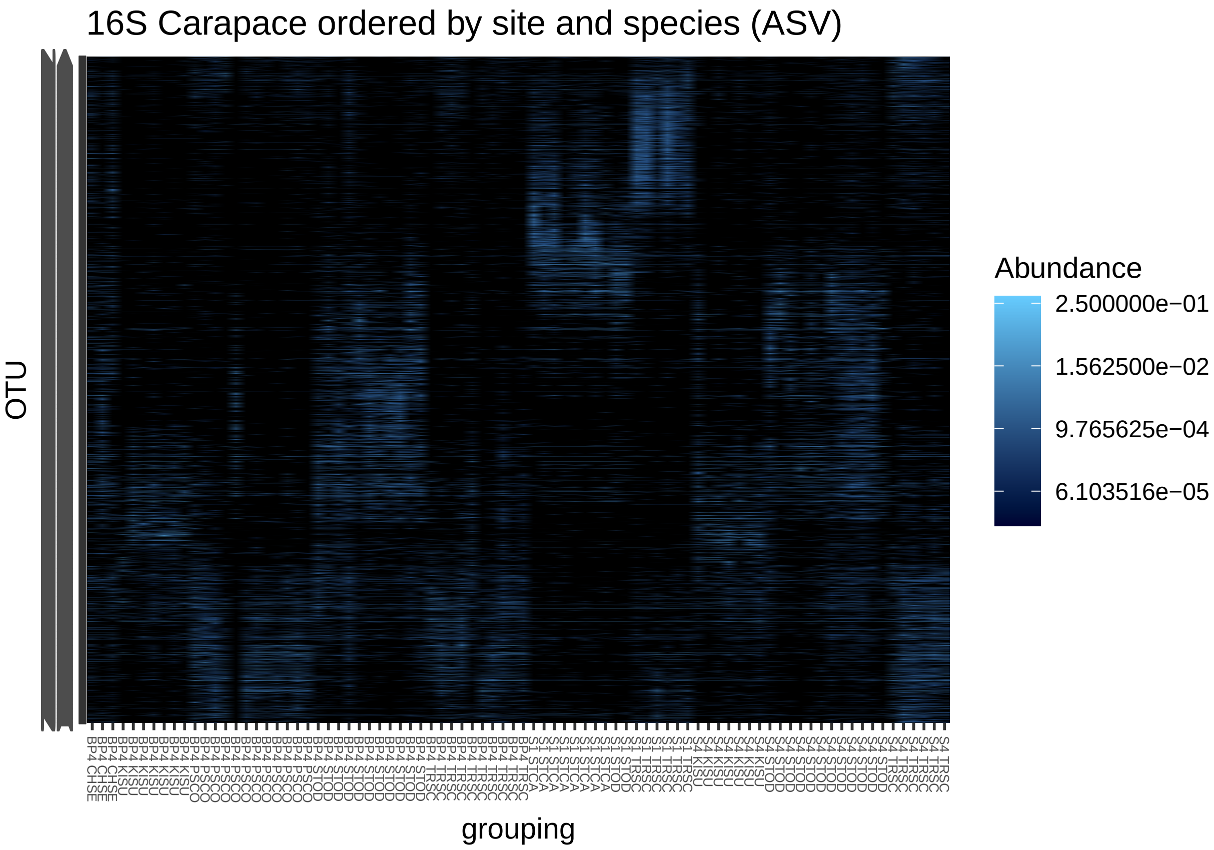


```
## Warning: Transformation introduced infinite values in discrete y-axis
```

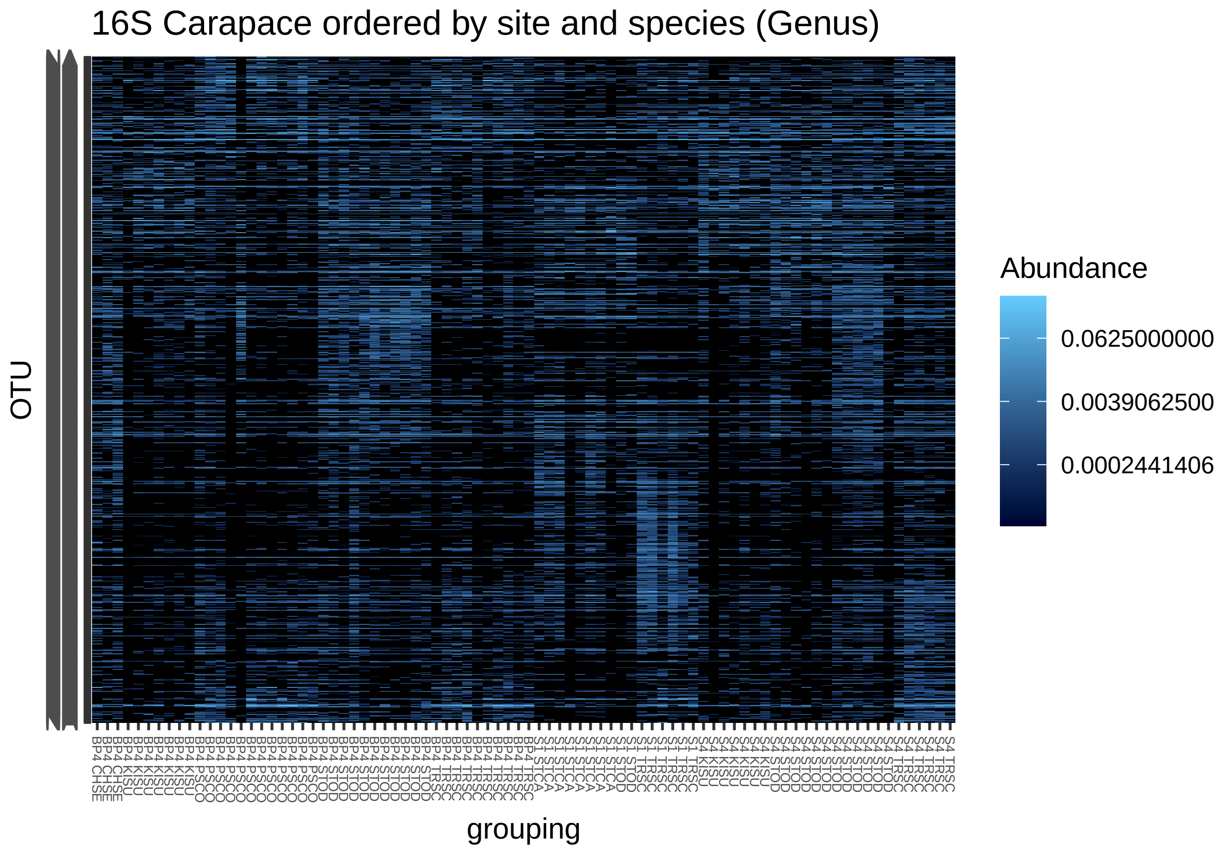


```
## Warning: Transformation introduced infinite values in discrete y-axis
```

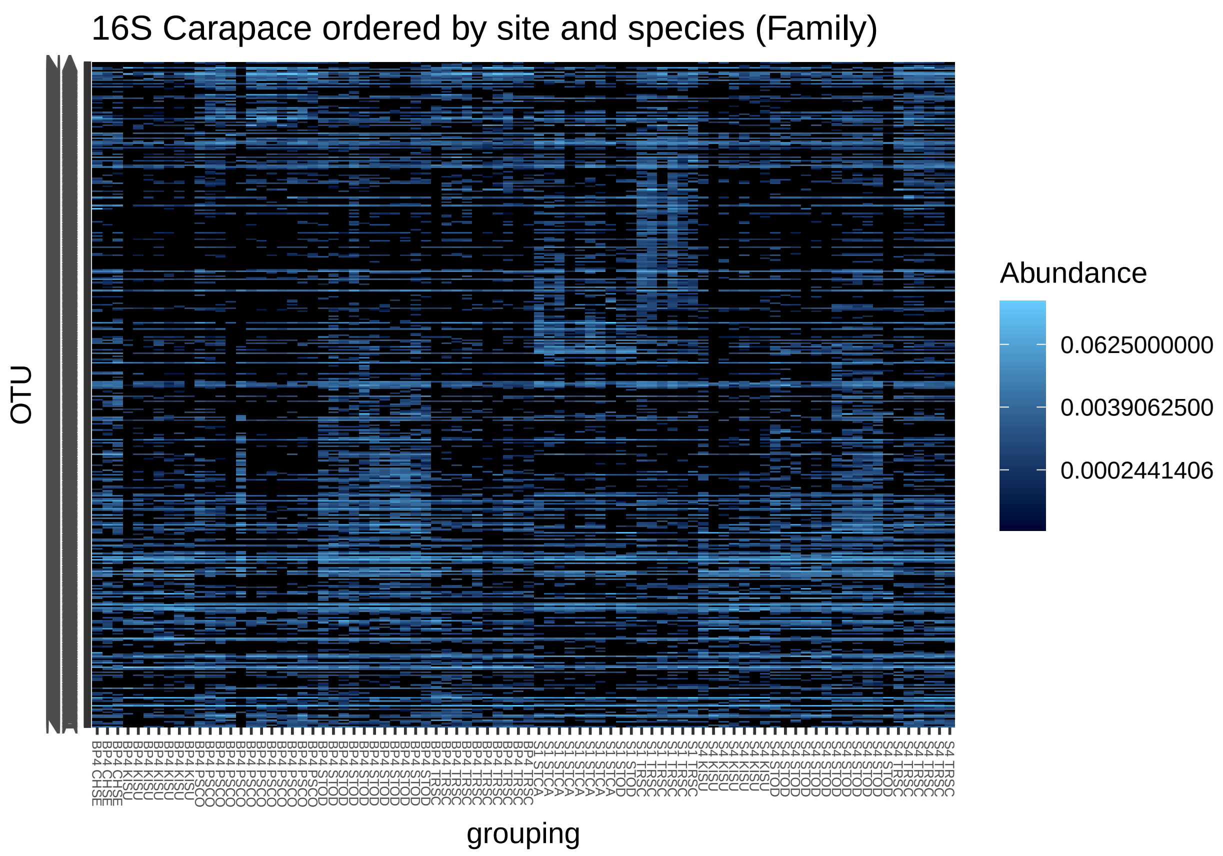


```
## Warning: Transformation introduced infinite values in discrete y-axis
```

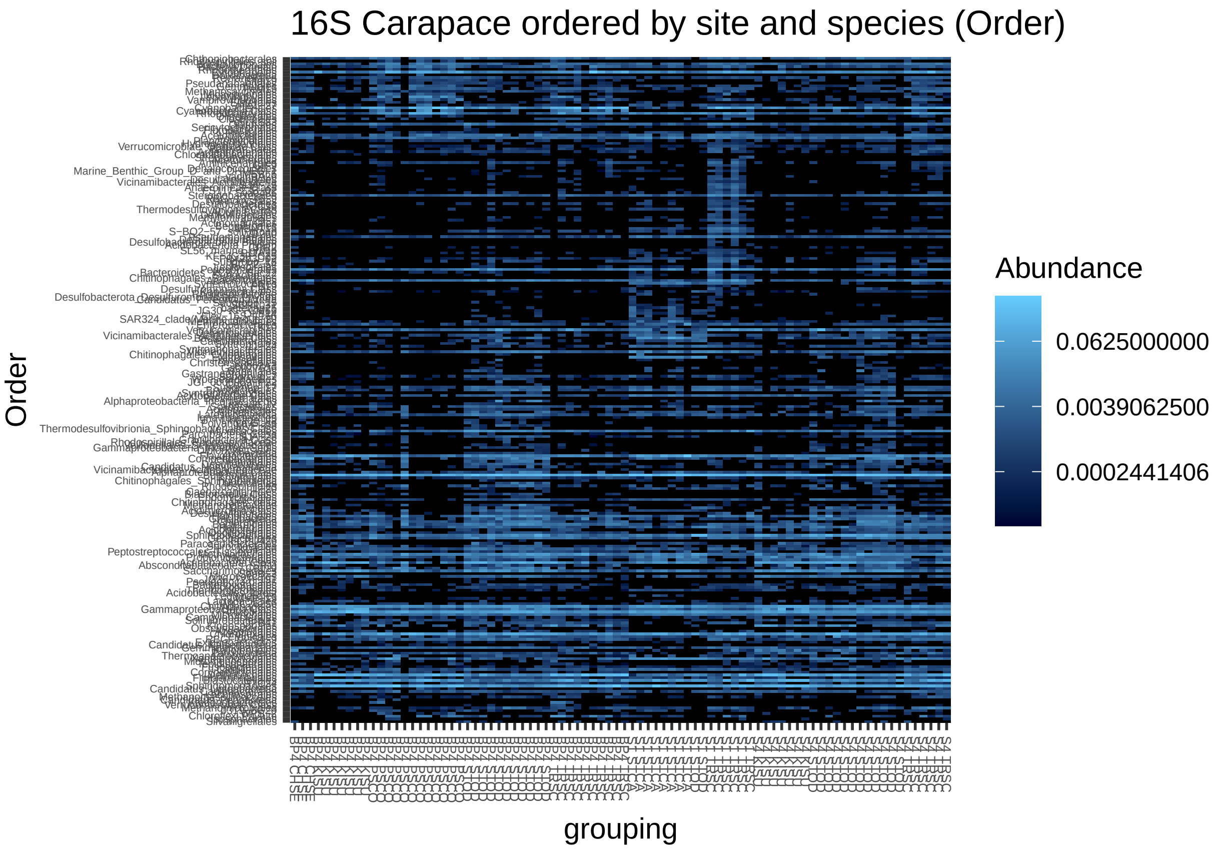


```
## Warning: Transformation introduced infinite values in discrete y-axis
```

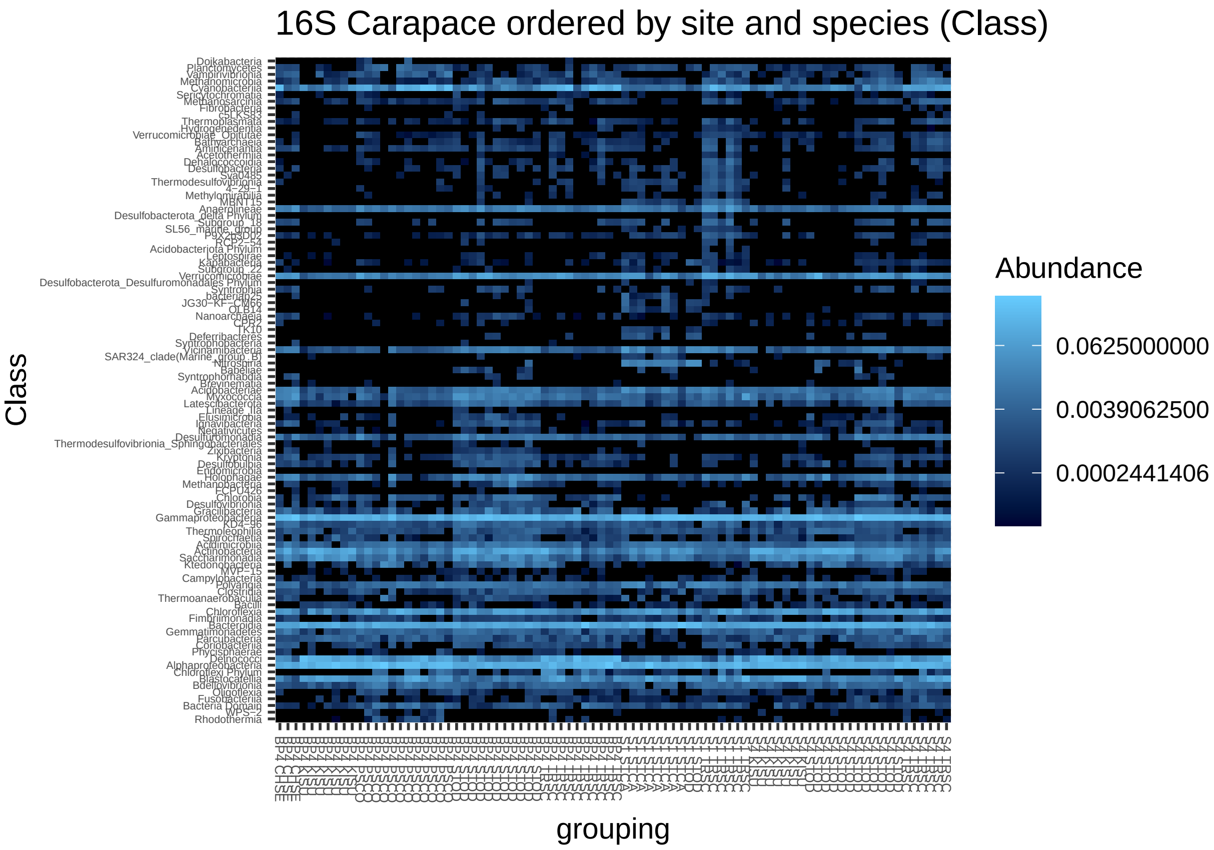


```
## Warning: Transformation introduced infinite values in discrete y-axis
```

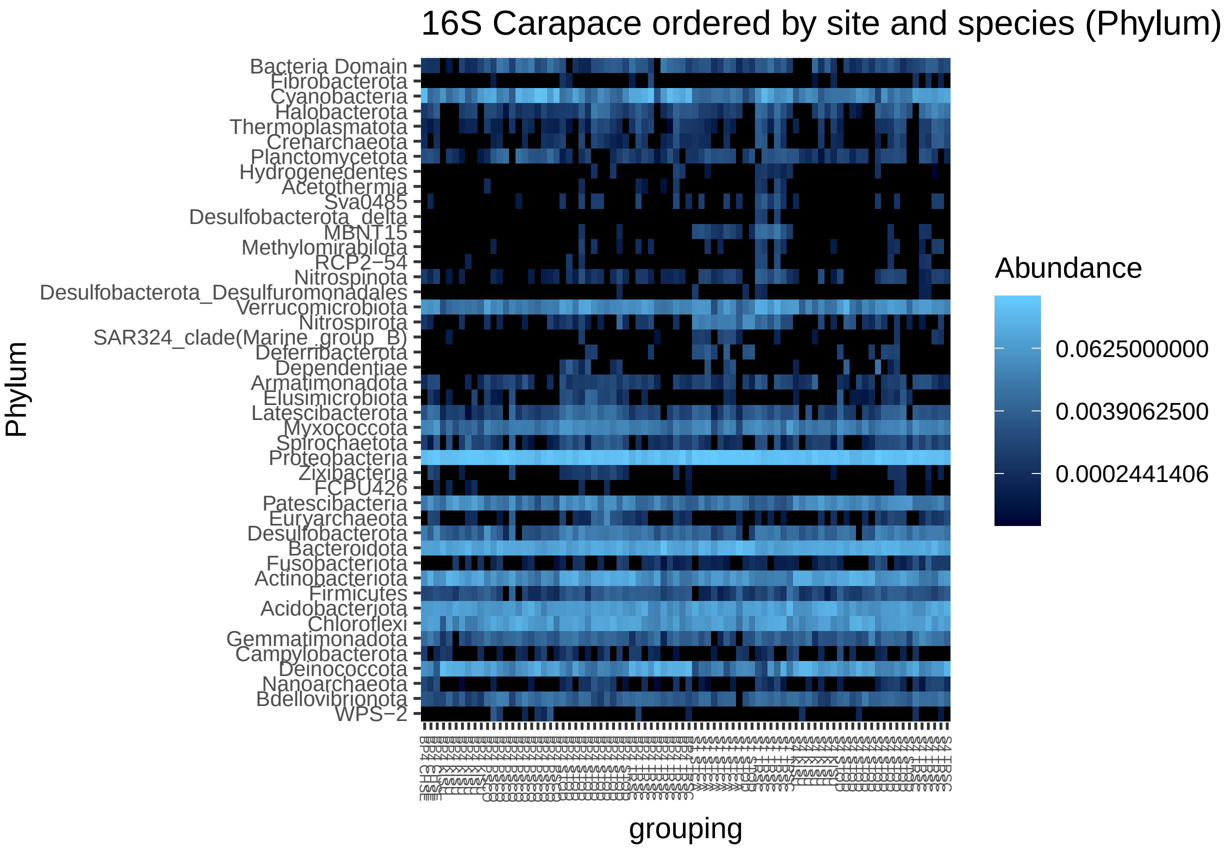

Beta diversity comparisons

The sections below feature beta diversity analyses. First, beta diversity matrices are calculated and stored in a data frame.

```
# Calculate distance matrices in vegan
carapace_bray <- phyloseq::distance(phylo_carapace, method = "bray")
carapace_jacc <- phyloseq::distance(phylo_carapace, method = "jaccard")
carapace_uni <- phyloseq::distance(phylo_carapace, method = "unifrac")
carapace_wuni <- phyloseq::distance(phylo_carapace, method = "wunifrac")

# make a data frame from the sample_data
sampledf <- data.frame(sample_data(phylo_carapace))
```

Beta dispersion testing by species and site

The below commands are used to generate comparisons of beta dispersion by species and by site, and to generate ordination plots for beta diversity by species and by site.

```
betadisper_bray_species <- betadisper(carapace_bray, sampledf$Species)
betadisper_jacc_species <- betadisper(carapace_jacc, sampledf$Species)
betadisper_uni_species <- betadisper(carapace_uni, sampledf$Species)
betadisper_wuni_species <- betadisper(carapace_wuni, sampledf$Species)

betadisper_bray_site <- betadisper(carapace_bray, sampledf$Site)
```

```r
betadisper_jacc_site <- betadisper(carapace_jacc, sampledf$Site)
betadisper_uni_site <- betadisper(carapace_uni, sampledf$Site)
betadisper_wuni_site <- betadisper(carapace_wuni, sampledf$Site)


plot(betadisper_bray_species, hull = FALSE, ellipse = TRUE)
```

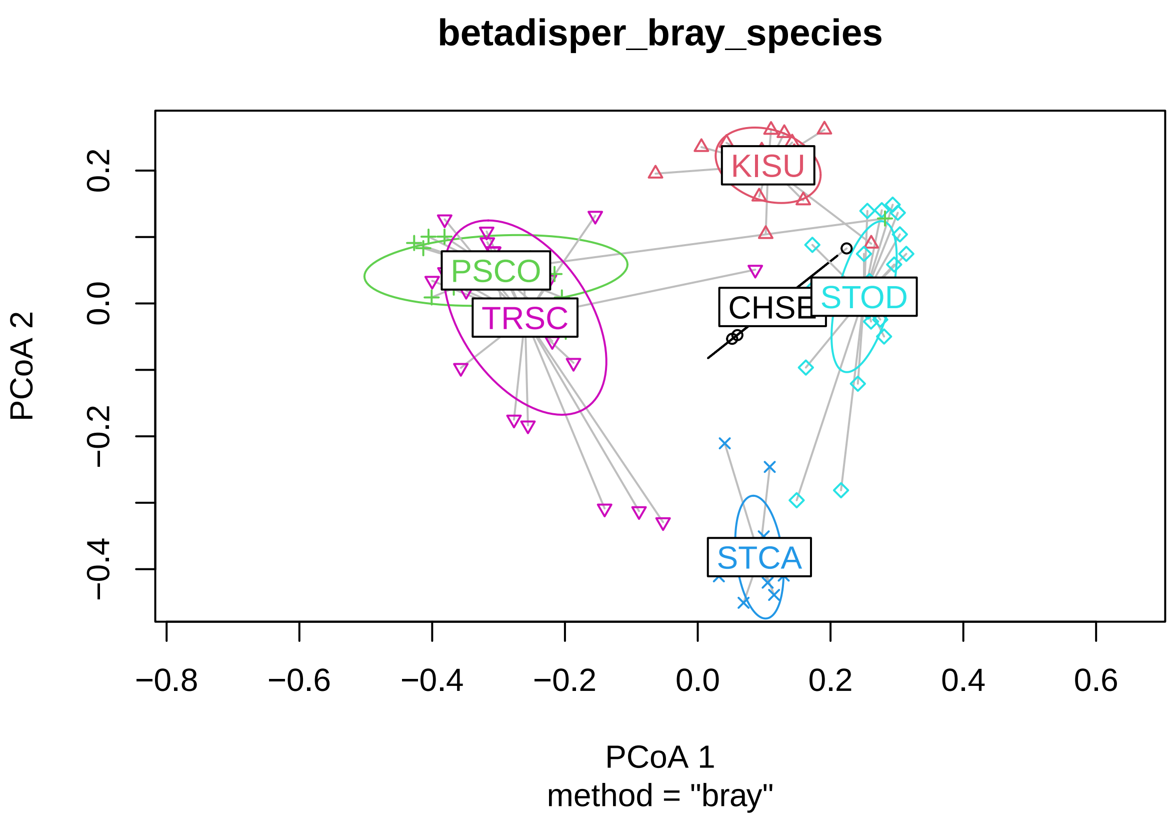


```r
print(betadisper_bray_species, digits = max(3, getOption("digits") - 3), neigen = 2)
```

```
##
##  Homogeneity of multivariate dispersions
##
## Call: betadisper(d = carapace_bray, group = sampledf$Species)
##
## No. of Positive Eigenvalues: 83
## No. of Negative Eigenvalues: 0
##
## Average distance to median:
##   CHSE   KISU   PSCO   STCA   STOD   TRSC
## 0.4043 0.4647 0.4830 0.4135 0.4963 0.5148
##
## Eigenvalues for PCoA axes:
## (Showing 2 of 83 eigenvalues)
## PCoA1 PCoA2
## 4.920 2.614
```

```r
plot(betadisper_bray_site, hull = FALSE, ellipse = TRUE)
```

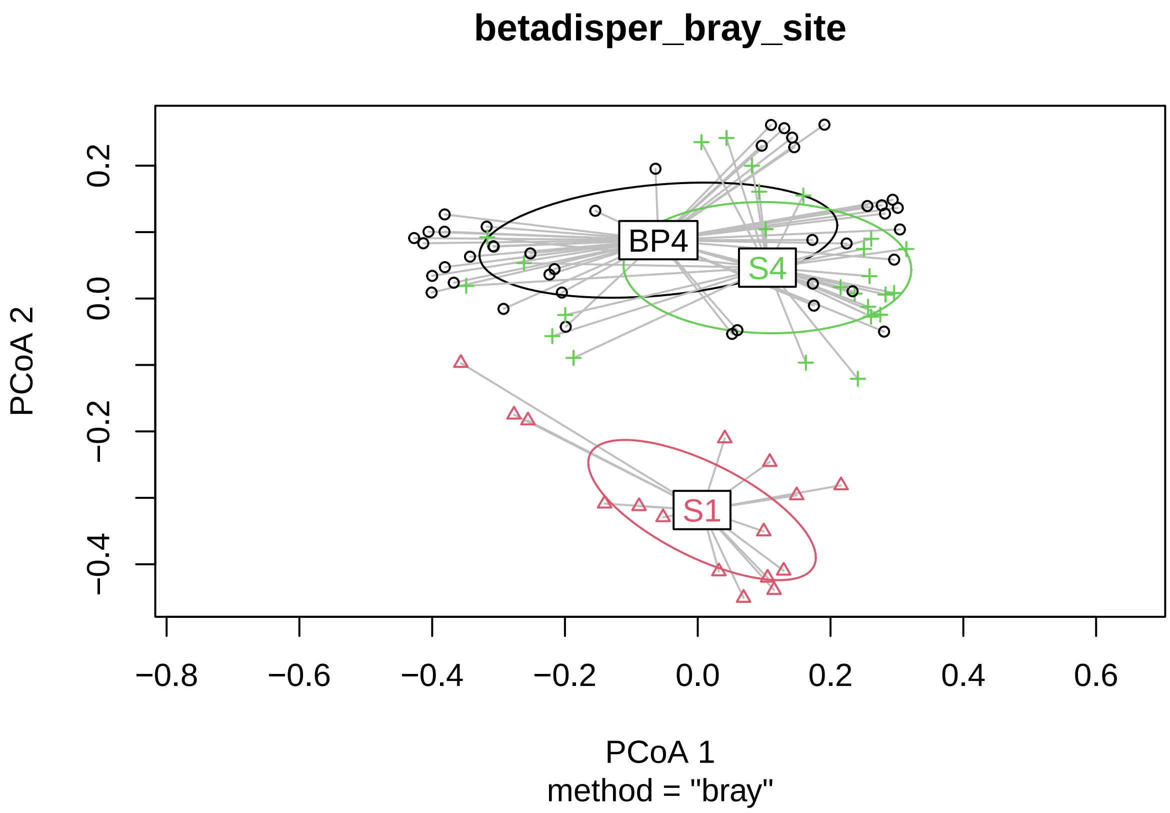


```r
print(betadisper_bray_site, digits = max(3, getOption("digits") - 3), neigen = 2)
```

```
##
##  Homogeneity of multivariate dispersions
##
## Call: betadisper(d = carapace_bray, group = sampledf$Site)
##
## No. of Positive Eigenvalues: 83
## No. of Negative Eigenvalues: 0
##
## Average distance to median:
##    BP4     S1     S4
## 0.5749 0.5098 0.5312
##
## Eigenvalues for PCoA axes:
## (Showing 2 of 83 eigenvalues)
## PCoA1 PCoA2
## 4.920 2.614
```

```r
plot(betadisper_jacc_species, hull = FALSE, ellipse = TRUE)
```

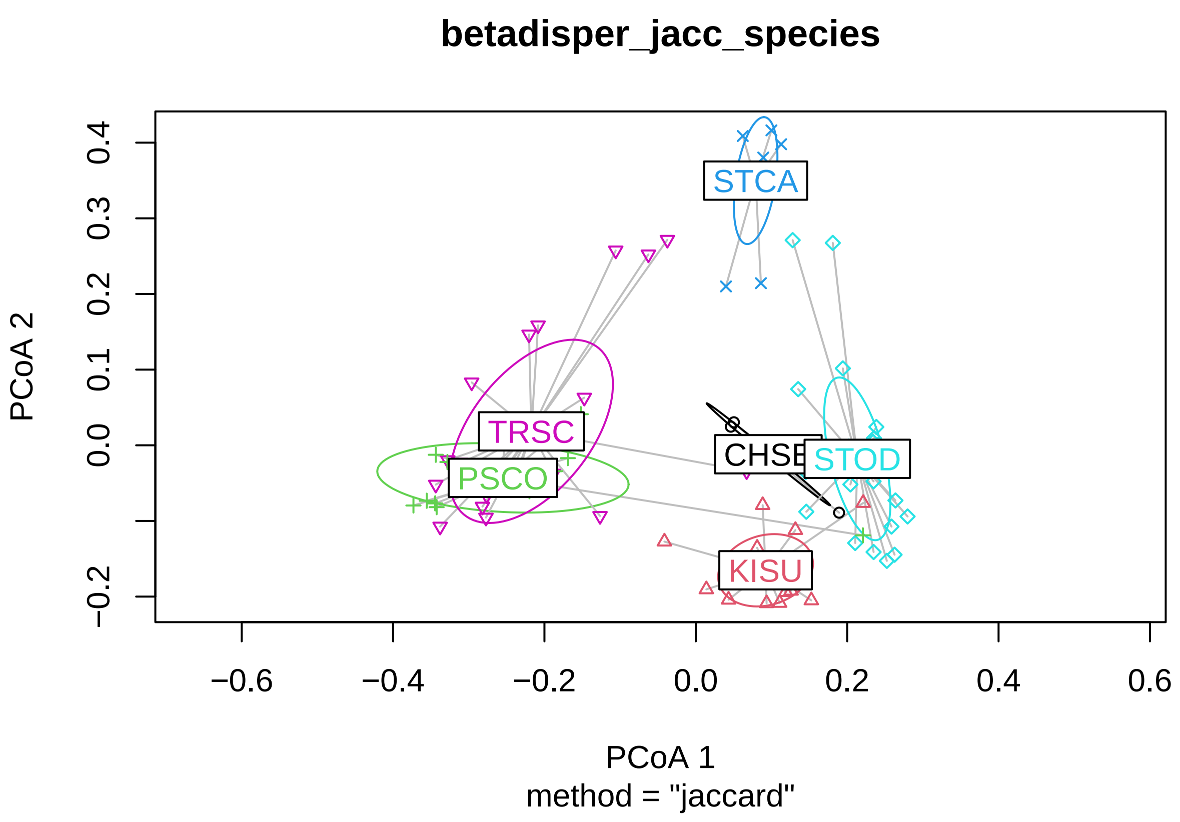


```r
print(betadisper_jacc_species, digits = max(3, getOption("digits") - 3), neigen = 2)
```

```
##
##  Homogeneity of multivariate dispersions
##
## Call: betadisper(d = carapace_jacc, group = sampledf$Species)
##
## No. of Positive Eigenvalues: 83
## No. of Negative Eigenvalues: 0
##
## Average distance to median:
##   CHSE   KISU   PSCO   STCA   STOD   TRSC
## 0.4756 0.5507 0.5584 0.5048 0.5756 0.5865
##
## Eigenvalues for PCoA axes:
## (Showing 2 of 83 eigenvalues)
## PCoA1 PCoA2
## 3.612 2.032
```

```r
plot(betadisper_jacc_site, hull = FALSE, ellipse = TRUE)
```

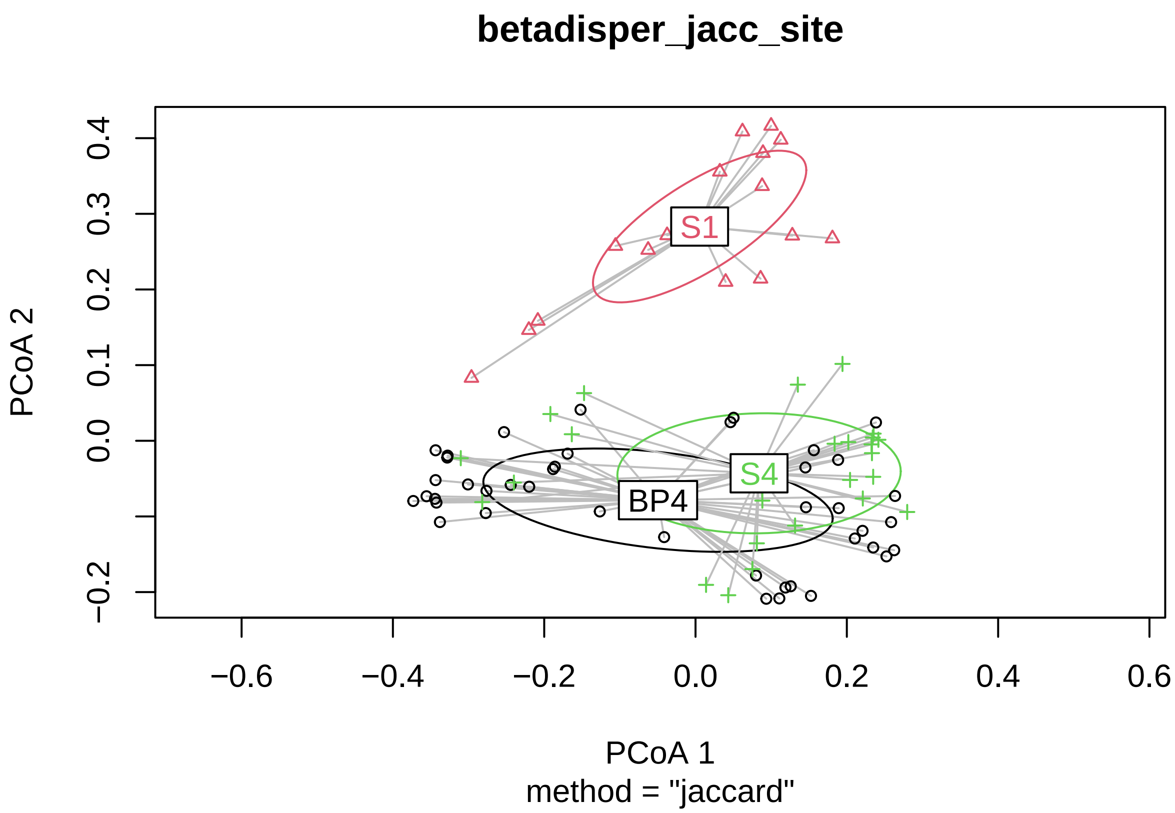


```r
print(betadisper_jacc_site, digits = max(3, getOption("digits") - 3), neigen = 2)
```

```
##
##  Homogeneity of multivariate dispersions
##
## Call: betadisper(d = carapace_jacc, group = sampledf$Site)
##
## No. of Positive Eigenvalues: 83
## No. of Negative Eigenvalues: 0
##
## Average distance to median:
##    BP4     S1     S4
## 0.6264 0.5788 0.5975
##
## Eigenvalues for PCoA axes:
## (Showing 2 of 83 eigenvalues)
## PCoA1 PCoA2
## 3.612 2.032
```

```r
plot(betadisper_uni_species, hull = FALSE, ellipse = TRUE)
```

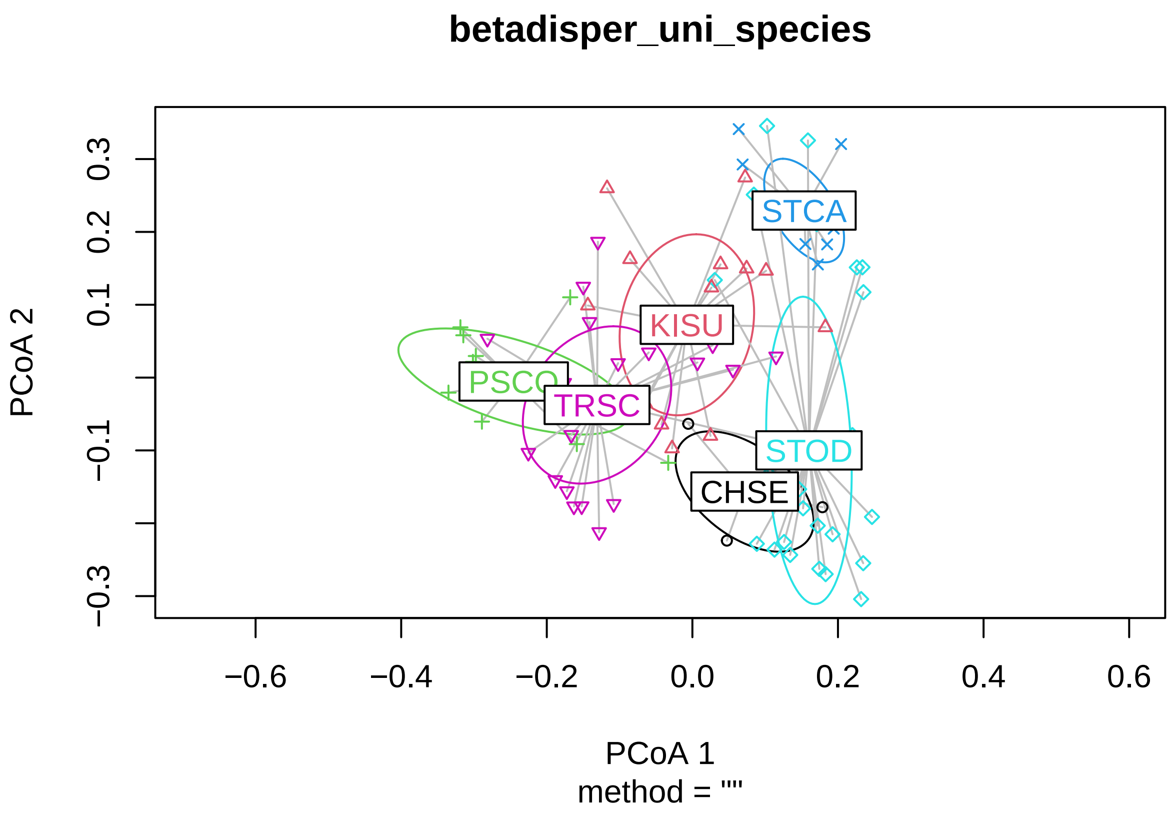


```
print(betadisper_uni_species, digits = max(3, getOption("digits") - 3), neigen = 2)
```

```
##
##  Homogeneity of multivariate dispersions
##
## Call: betadisper(d = carapace_uni, group = sampledf$Species)
##
## No. of Positive Eigenvalues: 83
## No. of Negative Eigenvalues: 0
##
## Average distance to median:
##   CHSE   KISU   PSCO   STCA   STOD   TRSC
## 0.3826 0.5041 0.4271 0.4135 0.4891 0.4827
##
## Eigenvalues for PCoA axes:
## (Showing 2 of 83 eigenvalues)
## PCoA1 PCoA2
## 2.447 2.358
```

```
plot(betadisper_uni_site, hull = FALSE, ellipse = TRUE)
```

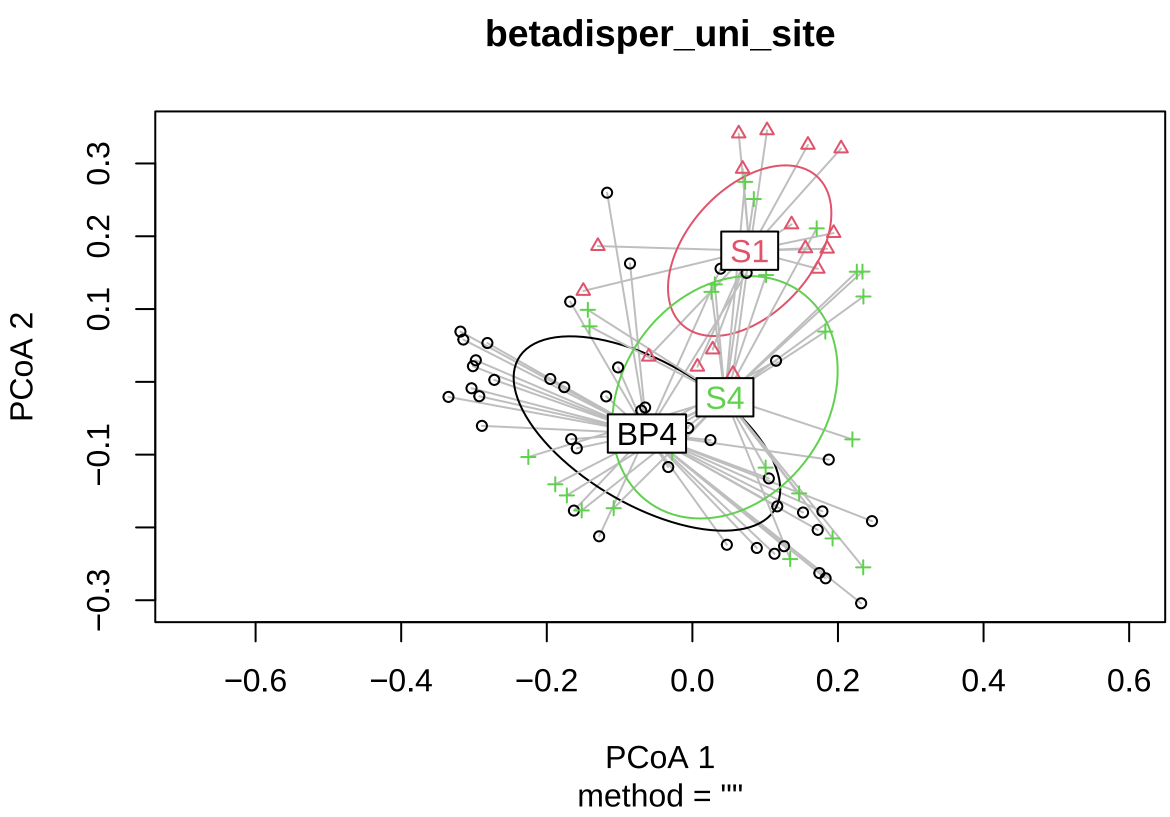


```r
print(betadisper_uni_site, digits = max(3, getOption("digits") - 3), neigen = 2)
```

```
##
##  Homogeneity of multivariate dispersions
##
## Call: betadisper(d = carapace_uni, group = sampledf$Site)
##
## No. of Positive Eigenvalues: 83
## No. of Negative Eigenvalues: 0
##
## Average distance to median:
##    BP4     S1     S4
## 0.5083 0.4831 0.5141
##
## Eigenvalues for PCoA axes:
## (Showing 2 of 83 eigenvalues)
## PCoA1 PCoA2
## 2.447 2.358
```

```r
plot(betadisper_wuni_species, hull = FALSE, ellipse = TRUE)
```

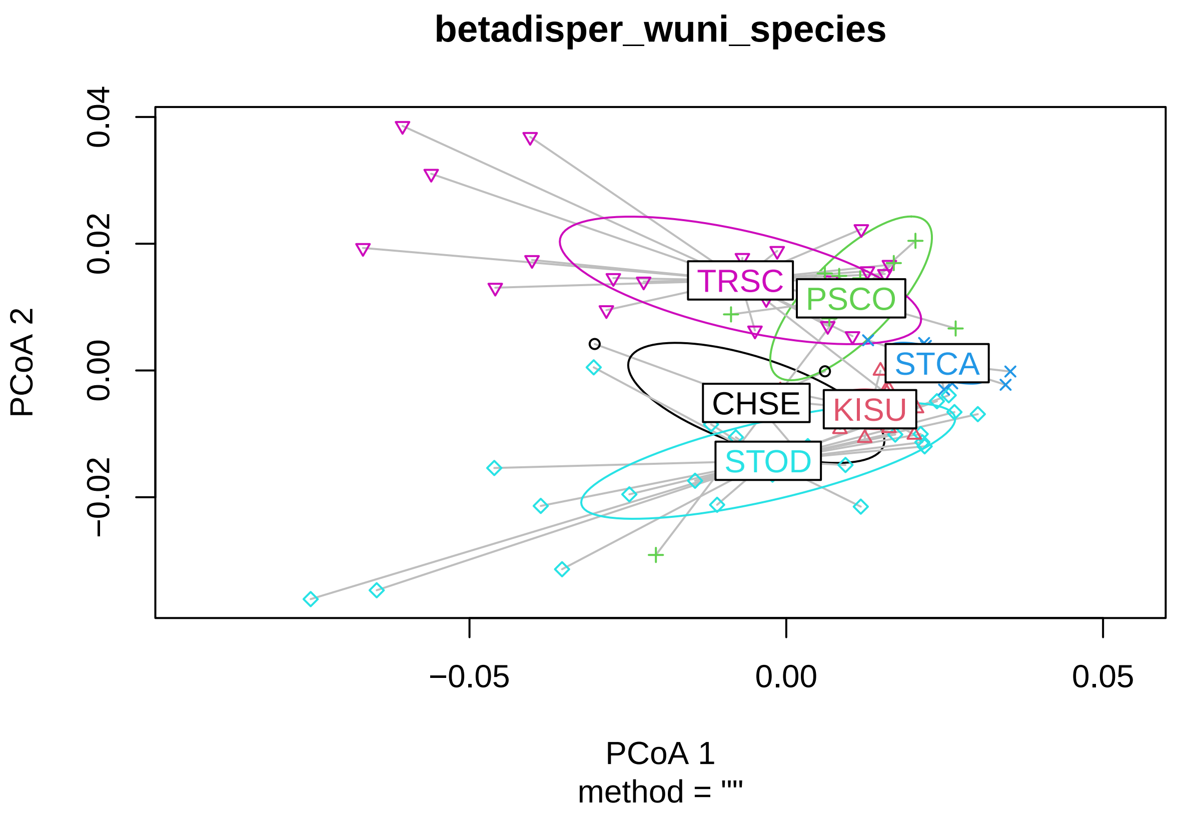


```r
print(betadisper_wuni_species, digits = max(3, getOption("digits") - 3), neigen = 2)
```

```
##
##  Homogeneity of multivariate dispersions
##
## Call: betadisper(d = carapace_wuni, group = sampledf$Species)
##
## No. of Positive Eigenvalues: 70
## No. of Negative Eigenvalues: 13
##
## Average distance to median:
##    CHSE    KISU    PSCO    STCA    STOD    TRSC
## 0.03449 0.02203 0.03180 0.02682 0.04268 0.04238
##
## Eigenvalues for PCoA axes:
## (Showing 2 of 83 eigenvalues)
##   PCoA1   PCoA2
## 0.05359 0.01909
```

```r
plot(betadisper_wuni_site, hull = FALSE, ellipse = TRUE)
```

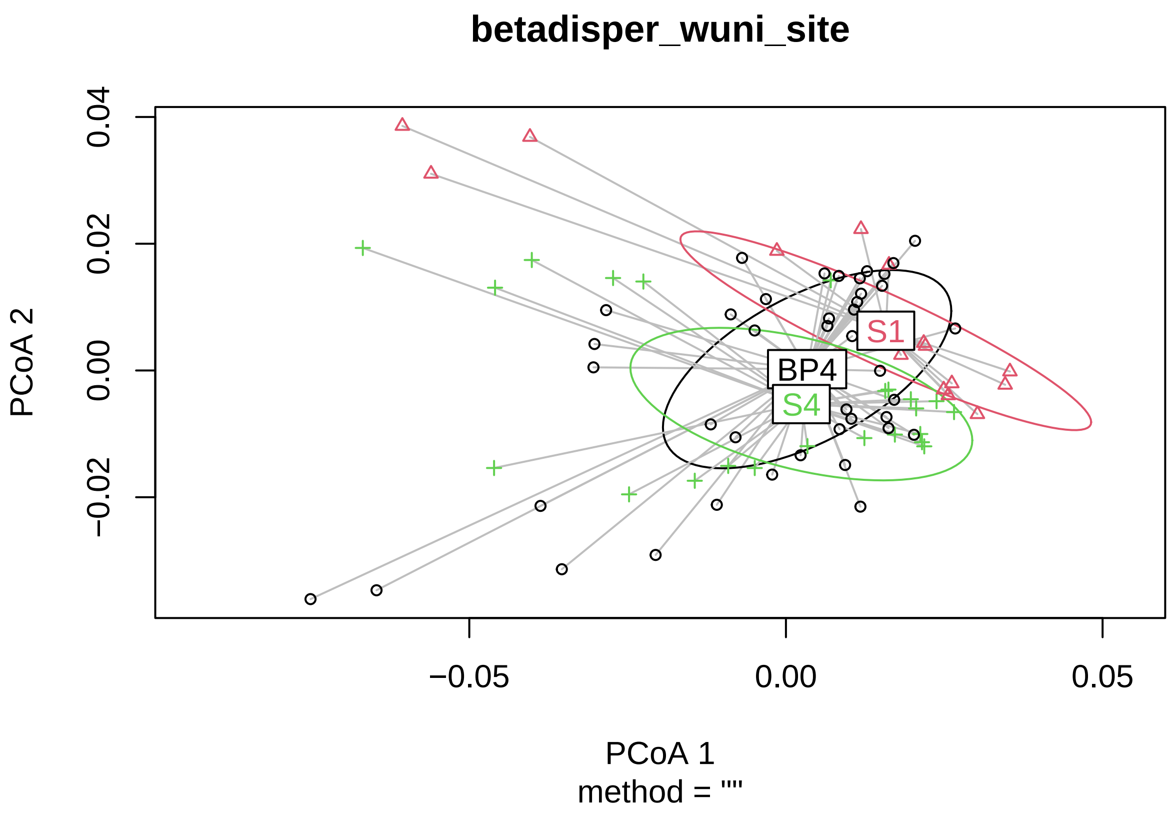


```r
print(betadisper_wuni_site, digits = max(3, getOption("digits") - 3), neigen = 2)
```

```
##
##  Homogeneity of multivariate dispersions
##
## Call: betadisper(d = carapace_wuni, group = sampledf$Site)
##
## No. of Positive Eigenvalues: 70
## No. of Negative Eigenvalues: 13
##
## Average distance to median:
##     BP4      S1      S4
## 0.03903 0.04173 0.03932
##
## Eigenvalues for PCoA axes:
## (Showing 2 of 83 eigenvalues)
##   PCoA1   PCoA2
## 0.05359 0.01909
```

In the following, ANOVA is used to test for significant differences in beta dispersion by site and by species, across four different beta diversity metrics (Bray, Jaccsard, UniFrac, weighted UniFrac). If results are significant, we can reject the null hypothesis that the species have the same dispersions. Pairwise comparisons with Tukey-corrected p-values are also presented.

```r
betadisper_bray_species.anova <- anova(betadisper_bray_species, permutations = 999)
betadisper_bray_species.anova
```

```
## Analysis of Variance Table
##
## Response: Distances
##           Df  Sum Sq   Mean Sq F value  Pr(>F)
## Groups     5 0.08860 0.0177190  2.4731 0.03922 *
## Residuals 78 0.55885 0.0071647
## ---
## Signif. codes:  0 '***' 0.001 '**' 0.01 '*' 0.05 '.' 0.1 ' ' 1
```

```
# test significance for beta dispersion between each group with p-values adjusted by Tukey HSD
betadisper_bray_species.TukeyHSD <- TukeyHSD(betadisper_bray_species)
betadisper_bray_species.TukeyHSD
```

```
##   Tukey multiple comparisons of means
##     95% family-wise confidence level
##
## Fit: aov(formula = distances ~ group, data = df)
##
## $group
##                   diff           lwr        upr     p adj
## KISU-CHSE  0.060372885 -0.0969692075 0.21771498 0.8712029
## PSCO-CHSE  0.078647747 -0.0809914316 0.23828693 0.7030267
## STCA-CHSE  0.009233152 -0.1581978312 0.17666413 0.9999843
## STOD-CHSE  0.091990158 -0.0591199404 0.24310026 0.4853207
## TRSC-CHSE  0.110533357 -0.0416766277 0.26274334 0.2871119
## PSCO-KISU  0.018274862 -0.0790171393 0.11556686 0.9938687
## STCA-KISU -0.051139733 -0.1607490405 0.05846957 0.7485723
## STOD-KISU  0.031617274 -0.0509377273 0.11417227 0.8720894
## TRSC-KISU  0.050160472 -0.0343909657 0.13471191 0.5143864
## STCA-PSCO -0.069414595 -0.1822965409 0.04346735 0.4738724
## STOD-PSCO  0.013342411 -0.0735106918 0.10019551 0.9976235
## TRSC-PSCO  0.031885610 -0.0568672999 0.12063852 0.8993646
## STOD-STCA  0.082757007 -0.0177015833 0.18321560 0.1666005
## TRSC-STCA  0.101300205 -0.0008053566 0.20340577 0.0530612
## TRSC-STOD  0.018543199 -0.0537525384 0.09083894 0.9748976
```

```
betadisper_bray_site.anova <- anova(betadisper_bray_site, permutations = 999)
betadisper_bray_site.anova
```

```
## Analysis of Variance Table
##
## Response: Distances
##           Df   Sum Sq   Mean Sq F value    Pr(>F)
## Groups     2 0.061292 0.0306461  12.348 2.086e-05 ***
## Residuals 81 0.201034 0.0024819
## ---
## Signif. codes:  0 '***' 0.001 '**' 0.01 '*' 0.05 '.' 0.1 ' ' 1
```

```
# test significance for beta dispersion between each group with p-values adjusted by Tukey HSD
betadisper_bray_site.TukeyHSD <- TukeyHSD(betadisper_bray_site)
betadisper_bray_site.TukeyHSD
```

```
##   Tukey multiple comparisons of means
##     95% family-wise confidence level
##
## Fit: aov(formula = distances ~ group, data = df)
##
## $group
##               diff         lwr         upr     p adj
## S1-BP4 -0.0650644 -0.09989618 -0.03023263 0.0000771
## S4-BP4 -0.0437063 -0.07362165 -0.01379096 0.0022538
## S4-S1   0.0213581 -0.01672267  0.05943887 0.3778832
```

```
betadisper_jacc_species.anova <- anova(betadisper_jacc_species, permutations = 999)
betadisper_jacc_species.anova
```

```
## Analysis of Variance Table
##
## Response: Distances
##           Df   Sum Sq   Mean Sq F value   Pr(>F)
## Groups     5 0.068345 0.0136690  4.0696 0.002464 **
## Residuals 78 0.261986 0.0033588
## ---
## Signif. codes:  0 '***' 0.001 '**' 0.01 '*' 0.05 '.' 0.1 ' ' 1
```

```
# test significance for beta dispersion between each group with p-values adjusted by Tukey HSD
betadisper_jacc_species.TukeyHSD <- TukeyHSD(betadisper_jacc_species)
betadisper_jacc_species.TukeyHSD
```

```
##   Tukey multiple comparisons of means
##     95% family-wise confidence level
##
## Fit: aov(formula = distances ~ group, data = df)
##
## $group
##                   diff          lwr        upr     p adj
## KISU-CHSE  0.075189743 -0.032540217 0.18291970 0.3301336
## PSCO-CHSE  0.082813808 -0.026488936 0.19211655 0.2433972
## STCA-CHSE  0.029294591 -0.085343094 0.14393228 0.9753015
## STOD-CHSE  0.100039778 -0.003423223 0.20350278 0.0639425
## TRSC-CHSE  0.110997559  0.006781481 0.21521364 0.0300726
## PSCO-KISU  0.007624065 -0.058990427 0.07423856 0.9994252
## STCA-KISU -0.045895152 -0.120943133 0.02915283 0.4801491
## STOD-KISU  0.024850035 -0.031674236 0.08137431 0.7925474
## TRSC-KISU  0.035807815 -0.022083388 0.09369902 0.4672157
## STCA-PSCO -0.053519217 -0.130807928 0.02376949 0.3388861
## STOD-PSCO  0.017225970 -0.042241152 0.07669309 0.9576946
## TRSC-PSCO  0.028183751 -0.032584143 0.08895164 0.7532506
## STOD-STCA  0.070745187  0.001962576 0.13952780 0.0401663
## TRSC-STCA  0.081702967  0.011792697 0.15161324 0.0125715
## TRSC-STOD  0.010957781 -0.038542113 0.06045767 0.9869641
```

```
betadisper_jacc_site.anova <- anova(betadisper_jacc_site, permutations = 999)
betadisper_jacc_site.anova
```

```
## Analysis of Variance Table
##
## Response: Distances
##           Df   Sum Sq   Mean Sq F value    Pr(>F)
## Groups     2 0.030939 0.0154697  14.725 3.512e-06 ***
## Residuals 81 0.085098 0.0010506
## ---
## Signif. codes:  0 '***' 0.001 '**' 0.01 '*' 0.05 '.' 0.1 ' ' 1
```

```
# test significance for beta dispersion between each group with p-values adjusted by Tukey HSD
betadisper_jacc_site.TukeyHSD <- TukeyHSD(betadisper_jacc_site)
betadisper_jacc_site.TukeyHSD
```

```
##   Tukey multiple comparisons of means
##     95% family-wise confidence level
##
## Fit: aov(formula = distances ~ group, data = df)
##
## $group
##               diff          lwr          upr     p adj
## S1-BP4 -0.04760656 -0.070268709 -0.024944403 0.0000091
## S4-BP4 -0.02893841 -0.048401854 -0.009474974 0.0018485
## S4-S1   0.01866814 -0.006107865  0.043444149 0.1763905
```

```
betadisper_uni_species.anova <- anova(betadisper_uni_species, permutations = 999)
betadisper_uni_species.anova
```

```
## Analysis of Variance Table
##
## Response: Distances
##           Df  Sum Sq   Mean Sq F value  Pr(>F)
## Groups     5 0.09952 0.0199035  3.2429 0.01033 *
## Residuals 78 0.47873 0.0061375
## ---
## Signif. codes:  0 '***' 0.001 '**' 0.01 '*' 0.05 '.' 0.1 ' ' 1
```

```
# test significance for beta dispersion between each group with p-values adjusted by Tukey HSD
betadisper_uni_species.TukeyHSD <- TukeyHSD(betadisper_uni_species)
betadisper_uni_species.TukeyHSD
```

```
##   Tukey multiple comparisons of means
##     95% family-wise confidence level
##
## Fit: aov(formula = distances ~ group, data = df)
##
## $group
##                   diff         lwr        upr     p adj
## KISU-CHSE  0.121477327 -0.02414923 0.26710388 0.1563839
## PSCO-CHSE  0.044481024 -0.10327158 0.19223363 0.9503079
## STCA-CHSE  0.030889593 -0.12407465 0.18585383 0.9919260
## STOD-CHSE  0.106513005 -0.03334559 0.24637160 0.2382720
## TRSC-CHSE  0.100119529 -0.04075705 0.24099611 0.3102951
```

```
## PSCO-KISU -0.076996303 -0.16704404 0.01305143 0.1372621
## STCA-KISU -0.090587734 -0.19203564 0.01086017 0.1072616
## STOD-KISU -0.014964322 -0.09137236 0.06144372 0.9925577
## TRSC-KISU -0.021357797 -0.09961362 0.05689802 0.9671533
## STCA-PSCO -0.013591431 -0.11806830 0.09088544 0.9989301
## STOD-PSCO  0.062031981 -0.01835413 0.14241809 0.2254043
## TRSC-PSCO  0.055638505 -0.02650595 0.13778296 0.3636434
## STOD-STCA  0.075623412 -0.01735513 0.16860196 0.1773806
## TRSC-STCA  0.069229937 -0.02527295 0.16373282 0.2778814
## TRSC-STOD -0.006393475 -0.07330614 0.06051919 0.9997622
```

```
betadisper_uni_site.anova <- anova(betadisper_uni_site, permutations = 999)
betadisper_uni_site.anova
```

```
## Analysis of Variance Table
##
## Response: Distances
##           Df   Sum Sq   Mean Sq F value Pr(>F)
## Groups     2 0.010198 0.0050989  1.5164 0.2257
## Residuals 81 0.272362 0.0033625
```

```
# test significance for beta dispersion between each group with p-values adjusted by Tukey HSD
betadisper_uni_site.TukeyHSD <- TukeyHSD(betadisper_uni_site)
betadisper_uni_site.TukeyHSD
```

```
##   Tukey multiple comparisons of means
##     95% family-wise confidence level
##
## Fit: aov(formula = distances ~ group, data = df)
##
## $group
##               diff         lwr        upr     p adj
## S1-BP4 -0.02514432 -0.06568721 0.01539856 0.3054772
## S4-BP4  0.00586578 -0.02895457 0.04068613 0.9147929
## S4-S1   0.03101010 -0.01331449 0.07533470 0.2228068
```

```
betadisper_wuni_species.anova <- anova(betadisper_wuni_species, permutations = 999)
betadisper_wuni_species.anova
```

```
## Analysis of Variance Table
##
## Response: Distances
##           Df    Sum Sq    Mean Sq F value    Pr(>F)
## Groups     5 0.0056337 0.00112673   5.996 9.586e-05 ***
## Residuals 78 0.0146573 0.00018791
## ---
## Signif. codes:  0 '***' 0.001 '**' 0.01 '*' 0.05 '.' 0.1 ' ' 1
```

```
# test significance for beta dispersion between each group with p-values adjusted by Tukey HSD
betadisper_wuni_species.TukeyHSD <- TukeyHSD(betadisper_wuni_species)
betadisper_wuni_species.TukeyHSD
```

```
##   Tukey multiple comparisons of means
##     95% family-wise confidence level
##
## Fit: aov(formula = distances ~ group, data = df)
##
## $group
##                    diff           lwr        upr     p adj
## KISU-CHSE -0.0124648872 -0.0379463174 0.01301654 0.7092484
## PSCO-CHSE -0.0026947302 -0.0285481717 0.02315871 0.9996359
## STCA-CHSE -0.0076770514 -0.0347923697 0.01943827 0.9615872
## STOD-CHSE  0.0081883345 -0.0162838292 0.03266050 0.9237076
## TRSC-CHSE  0.0078836271 -0.0167666622 0.03253392 0.9363349
## PSCO-KISU  0.0097701570 -0.0059862075 0.02552652 0.4643800
## STCA-KISU  0.0047878358 -0.0129633069 0.02253898 0.9687783
## STOD-KISU  0.0206532216  0.0072835031 0.03402294 0.0003127
## TRSC-KISU  0.0203485143  0.0066554743 0.03404155 0.0005830
## STCA-PSCO -0.0049823212 -0.0232634651 0.01329882 0.9673512
## STOD-PSCO  0.0108830647 -0.0031827283 0.02494886 0.2228506
## TRSC-PSCO  0.0105783573 -0.0037951078 0.02495182 0.2730449
## STOD-STCA  0.0158653859 -0.0004038051 0.03213458 0.0601808
## TRSC-STCA  0.0155606785 -0.0009752383 0.03209660 0.0770366
## TRSC-STOD -0.0003047074 -0.0120129461 0.01140353 0.9999996
```

```
betadisper_wuni_site.anova <- anova(betadisper_wuni_site, permutations = 999)
betadisper_wuni_site.anova
```

```
## Analysis of Variance Table
##
## Response: Distances
##           Df    Sum Sq    Mean Sq F value Pr(>F)
## Groups     2 0.0000884 4.4207e-05   0.147 0.8636
## Residuals 81 0.0243666 3.0082e-04
```

```
# test significance for beta dispersion between each group with p-values adjusted by Tukey HSD
betadisper_wuni_site.TukeyHSD <- TukeyHSD(betadisper_wuni_site)
betadisper_wuni_site.TukeyHSD
```

```
##   Tukey multiple comparisons of means
##     95% family-wise confidence level
##
## Fit: aov(formula = distances ~ group, data = df)
##
## $group
##                diff          lwr        upr     p adj
## S1-BP4  0.0027015580 -0.009425039 0.01482816 0.8559146
## S4-BP4  0.0002986019 -0.010116353 0.01071356 0.9974201
## S4-S1  -0.0024029560 -0.015660684 0.01085477 0.9020622
```

Testing for differences in centroid location

In this section, PERMANOVA (through Adonis2) is used to test for significant differences in centroid locations for each beta diversity metric, for both species and sites. If Adonis2 PERMANOVA result is signifi-

cant, we can reject the null hypothesis that the species have the same centroid. Pairwise comparisons with Benjamini-Hochberg-corrected p-values are also presented.

```
adonis2(carapace_bray ~ Species+Site+Sex+log10(Read_depth)+Year, data = sampledf, by="margin", permutat
```

```
## Permutation test for adonis under reduced model
## Marginal effects of terms
## Permutation: free
## Number of permutations: 999
##
## adonis2(formula = carapace_bray ~ Species + Site + Sex + log10(Read_depth) + Year, data = sampledf, p
##                   Df SumOfSqs      R2      F Pr(>F)
## Species            5   6.6191 0.22907 5.8970  0.001 ***
## Site               2   1.8719 0.06478 4.1693  0.001 ***
## Sex                2   0.8447 0.02923 1.8815  0.005 **
## log10(Read_depth)  1   0.4446 0.01539 1.9803  0.006 **
## Year               1   0.6404 0.02216 2.8526  0.002 **
## Residual          72  16.1633 0.55937
## Total             83  28.8957 1.00000
## ---
## Signif. codes:  0 '***' 0.001 '**' 0.01 '*' 0.05 '.' 0.1 ' ' 1
```

```
adonis2(carapace_jacc ~ Species+Site+Sex+log10(Read_depth)+Year, data = sampledf, by="margin", permutat
```

```
## Permutation test for adonis under reduced model
## Marginal effects of terms
## Permutation: free
## Number of permutations: 999
##
## adonis2(formula = carapace_jacc ~ Species + Site + Sex + log10(Read_depth) + Year, data = sampledf, p
##                   Df SumOfSqs      R2      F Pr(>F)
## Species            5    5.700 0.16776 3.5967  0.001 ***
## Site               2    1.747 0.05142 2.7563  0.001 ***
## Sex                2    0.960 0.02826 1.5150  0.003 **
## log10(Read_depth)  1    0.470 0.01383 1.4825  0.016 *
## Year               1    0.637 0.01876 2.0112  0.001 ***
## Residual          72   22.822 0.67165
## Total             83   33.978 1.00000
## ---
## Signif. codes:  0 '***' 0.001 '**' 0.01 '*' 0.05 '.' 0.1 ' ' 1
```

```
adonis2(carapace_uni ~ Species+Site+Sex+log10(Read_depth)+Year, data = sampledf, by="margin", permutati
```

```
## Permutation test for adonis under reduced model
## Marginal effects of terms
## Permutation: free
## Number of permutations: 999
##
## adonis2(formula = carapace_uni ~ Species + Site + Sex + log10(Read_depth) + Year, data = sampledf, pe
##                   Df SumOfSqs      R2      F Pr(>F)
## Species            5   3.8762 0.15861 3.6422  0.001 ***
## Site               2   1.7128 0.07009 4.0236  0.001 ***
```

```
## Sex                 2   0.5772 0.02362 1.3558  0.035 *
## log10(Read_depth)   1   0.4817 0.01971 2.2631  0.001 ***
## Year                1   0.6459 0.02643 3.0346  0.001 ***
## Residual           72  15.3248 0.62710
## Total              83  24.4377 1.00000
## ---
## Signif. codes:  0 '***' 0.001 '**' 0.01 '*' 0.05 '.' 0.1 ' ' 1
```

```
adonis2(carapace_wuni ~ Species+Site+Sex+log10(Read_depth)+Year, data = sampledf, by="margin", permutat
```

```
## Permutation test for adonis under reduced model
## Marginal effects of terms
## Permutation: free
## Number of permutations: 999
##
## adonis2(formula = carapace_wuni ~ Species + Site + Sex + log10(Read_depth) + Year, data = sampledf, 
##                   Df SumOfSqs      R2      F Pr(>F)
## Species            5 0.033546 0.20051 4.8124  0.001 ***
## Site               2 0.010817 0.06465 3.8793  0.002 **
## Sex                2 0.005353 0.03199 1.9197  0.024 *
## log10(Read_depth)  1 0.001696 0.01014 1.2167  0.234
## Year               1 0.006249 0.03735 4.4826  0.001 ***
## Residual          72 0.100378 0.59997
## Total             83 0.167305 1.00000
## ---
## Signif. codes:  0 '***' 0.001 '**' 0.01 '*' 0.05 '.' 0.1 ' ' 1
```

```
#Pairwise comparisons for beta diversity for species and site
pairwise.adonis2(carapace_bray ~ Species, data = sampledf, p.adjust.m="BH")
```

```
## $parent_call
## [1] "carapace_bray ~ Species , strata = Null , permutations 999"
##
## $KISU_vs_STOD
##          Df SumOfSqs      R2     F Pr(>F)
## Species   1   1.7945 0.16153 7.128  0.001 ***
## Residual 37   9.3146 0.83847
## Total    38  11.1091 1.00000
## ---
## Signif. codes:  0 '***' 0.001 '**' 0.01 '*' 0.05 '.' 0.1 ' ' 1
##
## $KISU_vs_TRSC
##          Df SumOfSqs      R2      F Pr(>F)
## Species   1   2.3497 0.20649 8.8477  0.001 ***
## Residual 34   9.0296 0.79351
## Total    35  11.3794 1.00000
## ---
## Signif. codes:  0 '***' 0.001 '**' 0.01 '*' 0.05 '.' 0.1 ' ' 1
##
## $KISU_vs_PSCO
##          Df SumOfSqs      R2      F Pr(>F)
## Species   1   1.9626 0.24541 7.8055  0.001 ***
## Residual 24   6.0346 0.75459
```

```
## Total    25   7.9972 1.00000
## ---
## Signif. codes:  0 '***' 0.001 '**' 0.01 '*' 0.05 '.' 0.1 ' ' 1
##
## $KISU_vs_STCA
##          Df SumOfSqs      R2     F Pr(>F)
## Species   1   1.9732 0.30445 8.754  0.001 ***
## Residual 20   4.5082 0.69555
## Total    21   6.4815 1.00000
## ---
## Signif. codes:  0 '***' 0.001 '**' 0.01 '*' 0.05 '.' 0.1 ' ' 1
##
## $KISU_vs_CHSE
##          Df SumOfSqs      R2      F Pr(>F)
## Species   1   0.8762 0.19731 3.6871  0.002 **
## Residual 15   3.5644 0.80269
## Total    16   4.4405 1.00000
## ---
## Signif. codes:  0 '***' 0.001 '**' 0.01 '*' 0.05 '.' 0.1 ' ' 1
##
## $STOD_vs_TRSC
##          Df SumOfSqs      R2      F Pr(>F)
## Species   1   3.1726 0.20642 11.705  0.001 ***
## Residual 45  12.1974 0.79358
## Total    46  15.3700 1.00000
## ---
## Signif. codes:  0 '***' 0.001 '**' 0.01 '*' 0.05 '.' 0.1 ' ' 1
##
## $STOD_vs_PSCO
##          Df SumOfSqs      R2      F Pr(>F)
## Species   1   2.4892 0.21291 9.4673  0.001 ***
## Residual 35   9.2024 0.78709
## Total    36  11.6916 1.00000
## ---
## Signif. codes:  0 '***' 0.001 '**' 0.01 '*' 0.05 '.' 0.1 ' ' 1
##
## $STOD_vs_STCA
##          Df SumOfSqs      R2      F Pr(>F)
## Species   1   1.6411 0.17614 6.6276  0.001 ***
## Residual 31   7.6760 0.82386
## Total    32   9.3171 1.00000
## ---
## Signif. codes:  0 '***' 0.001 '**' 0.01 '*' 0.05 '.' 0.1 ' ' 1
##
## $STOD_vs_CHSE
##          Df SumOfSqs      R2      F Pr(>F)
## Species   1   0.5959 0.08132 2.3014  0.004 **
## Residual 26   6.7322 0.91868
## Total    27   7.3280 1.00000
## ---
## Signif. codes:  0 '***' 0.001 '**' 0.01 '*' 0.05 '.' 0.1 ' ' 1
##
## $TRSC_vs_PSCO
##          Df SumOfSqs      R2      F Pr(>F)
```

```
## Species   1   0.5970 0.06275 2.1425  0.004 **
## Residual 32   8.9174 0.93725
## Total    33   9.5144 1.00000
## ---
## Signif. codes:  0 '***' 0.001 '**' 0.01 '*' 0.05 '.' 0.1 ' ' 1
##
## $TRSC_vs_STCA
##          Df SumOfSqs      R2      F Pr(>F)
## Species   1    1.888 0.20347 7.1526  0.001 ***
## Residual 28    7.391 0.79653
## Total    29    9.279 1.00000
## ---
## Signif. codes:  0 '***' 0.001 '**' 0.01 '*' 0.05 '.' 0.1 ' ' 1
##
## $TRSC_vs_CHSE
##          Df SumOfSqs     R2      F Pr(>F)
## Species   1   0.8025 0.1107 2.8629  0.001 ***
## Residual 23   6.4471 0.8893
## Total    24   7.2496 1.0000
## ---
## Signif. codes:  0 '***' 0.001 '**' 0.01 '*' 0.05 '.' 0.1 ' ' 1
##
## $PSCO_vs_STCA
##          Df SumOfSqs      R2      F Pr(>F)
## Species   1   1.8552 0.29678 7.5966  0.001 ***
## Residual 18   4.3959 0.70322
## Total    19   6.2512 1.00000
## ---
## Signif. codes:  0 '***' 0.001 '**' 0.01 '*' 0.05 '.' 0.1 ' ' 1
##
## $PSCO_vs_CHSE
##          Df SumOfSqs      R2      F Pr(>F)
## Species   1   0.9103 0.20867 3.4281  0.005 **
## Residual 13   3.4521 0.79133
## Total    14   4.3624 1.00000
## ---
## Signif. codes:  0 '***' 0.001 '**' 0.01 '*' 0.05 '.' 0.1 ' ' 1
##
## $STCA_vs_CHSE
##          Df SumOfSqs      R2      F Pr(>F)
## Species   1  0.93341 0.32647 4.3624  0.009 **
## Residual  9  1.92572 0.67353
## Total    10  2.85913 1.00000
## ---
## Signif. codes:  0 '***' 0.001 '**' 0.01 '*' 0.05 '.' 0.1 ' ' 1
##
## attr(,"class")
## [1] "pwadstrata" "list"
```

```
pairwise.adonis2(carapace_bray ~ Site, data = sampledf, p.adjust.m="BH")
```

```
## $parent_call
## [1] "carapace_bray ~ Site , strata = Null , permutations 999"
##
```

```
## $BP4_vs_S4
##          Df SumOfSqs      R2      F Pr(>F)
## Site      1   1.1106 0.04937 3.4275  0.001 ***
## Residual 66  21.3867 0.95063
## Total    67  22.4973 1.00000
## ---
## Signif. codes:  0 '***' 0.001 '**' 0.01 '*' 0.05 '.' 0.1 ' ' 1
##
## $BP4_vs_S1
##          Df SumOfSqs      R2      F Pr(>F)
## Site      1   2.2087 0.10676 6.8128  0.001 ***
## Residual 57  18.4791 0.89324
## Total    58  20.6878 1.00000
## ---
## Signif. codes:  0 '***' 0.001 '**' 0.01 '*' 0.05 '.' 0.1 ' ' 1
##
## $S4_vs_S1
##          Df SumOfSqs      R2      F Pr(>F)
## Site      1   1.7402 0.13318 5.9921  0.001 ***
## Residual 39  11.3261 0.86682
## Total    40  13.0663 1.00000
## ---
## Signif. codes:  0 '***' 0.001 '**' 0.01 '*' 0.05 '.' 0.1 ' ' 1
##
## attr(,"class")
## [1] "pwadstrata" "list"
```

```r
pairwise.adonis2(carapace_jacc ~ Species, data = sampledf, p.adjust.m="BH")
```

```
## $parent_call
## [1] "carapace_jacc ~ Species , strata = Null , permutations 999"
##
## $KISU_vs_STOD
##          Df SumOfSqs      R2      F Pr(>F)
## Species   1   1.5091 0.10702 4.4344  0.001 ***
## Residual 37  12.5920 0.89298
## Total    38  14.1012 1.00000
## ---
## Signif. codes:  0 '***' 0.001 '**' 0.01 '*' 0.05 '.' 0.1 ' ' 1
##
## $KISU_vs_TRSC
##          Df SumOfSqs      R2      F Pr(>F)
## Species   1   1.8576 0.13508 5.3102  0.001 ***
## Residual 34  11.8940 0.86492
## Total    35  13.7517 1.00000
## ---
## Signif. codes:  0 '***' 0.001 '**' 0.01 '*' 0.05 '.' 0.1 ' ' 1
##
## $KISU_vs_PSCO
##          Df SumOfSqs      R2      F Pr(>F)
## Species   1   1.6104 0.16604 4.7784  0.001 ***
## Residual 24   8.0887 0.83396
## Total    25   9.6991 1.00000
## ---
```

```
## Signif. codes:  0 '***' 0.001 '**' 0.01 '*' 0.05 '.' 0.1 ' ' 1
##
## $KISU_vs_STCA
##          Df SumOfSqs      R2      F Pr(>F)
## Species   1   1.6156 0.20285 5.0894  0.001 ***
## Residual 20   6.3490 0.79715
## Total    21   7.9646 1.00000
## ---
## Signif. codes:  0 '***' 0.001 '**' 0.01 '*' 0.05 '.' 0.1 ' ' 1
##
## $KISU_vs_CHSE
##          Df SumOfSqs      R2      F Pr(>F)
## Species   1   0.7923 0.13796 2.4005  0.004 **
## Residual 15   4.9505 0.86204
## Total    16   5.7428 1.00000
## ---
## Signif. codes:  0 '***' 0.001 '**' 0.01 '*' 0.05 '.' 0.1 ' ' 1
##
## $STOD_vs_TRSC
##          Df SumOfSqs     R2      F Pr(>F)
## Species   1   2.4055 0.1311 6.7899  0.001 ***
## Residual 45  15.9424 0.8689
## Total    46  18.3479 1.0000
## ---
## Signif. codes:  0 '***' 0.001 '**' 0.01 '*' 0.05 '.' 0.1 ' ' 1
##
## $STOD_vs_PSCO
##          Df SumOfSqs      R2      F Pr(>F)
## Species   1    1.944 0.13806 5.6059  0.001 ***
## Residual 35   12.137 0.86194
## Total    36   14.081 1.00000
## ---
## Signif. codes:  0 '***' 0.001 '**' 0.01 '*' 0.05 '.' 0.1 ' ' 1
##
## $STOD_vs_STCA
##          Df SumOfSqs      R2      F Pr(>F)
## Species   1   1.4182 0.12003 4.2286  0.001 ***
## Residual 31  10.3973 0.87997
## Total    32  11.8156 1.00000
## ---
## Signif. codes:  0 '***' 0.001 '**' 0.01 '*' 0.05 '.' 0.1 ' ' 1
##
## $STOD_vs_CHSE
##          Df SumOfSqs      R2      F Pr(>F)
## Species   1   0.5958 0.06209 1.7213  0.009 **
## Residual 26   8.9989 0.93791
## Total    27   9.5946 1.00000
## ---
## Signif. codes:  0 '***' 0.001 '**' 0.01 '*' 0.05 '.' 0.1 ' ' 1
##
## $TRSC_vs_PSCO
##          Df SumOfSqs      R2      F Pr(>F)
## Species   1   0.6283 0.05207 1.7578  0.006 **
## Residual 32  11.4390 0.94793
```

```
## Total    33  12.0674 1.00000
## ---
## Signif. codes:  0 '***' 0.001 '**' 0.01 '*' 0.05 '.' 0.1 ' ' 1
##
## $TRSC_vs_STCA
##          Df SumOfSqs     R2      F Pr(>F)
## Species   1   1.5633 0.1388 4.5128  0.001 ***
## Residual 28   9.6993 0.8612
## Total    29  11.2626 1.0000
## ---
## Signif. codes:  0 '***' 0.001 '**' 0.01 '*' 0.05 '.' 0.1 ' ' 1
##
## $TRSC_vs_CHSE
##          Df SumOfSqs      R2      F Pr(>F)
## Species   1   0.7384 0.08168 2.0458  0.001 ***
## Residual 23   8.3009 0.91832
## Total    24   9.0393 1.00000
## ---
## Signif. codes:  0 '***' 0.001 '**' 0.01 '*' 0.05 '.' 0.1 ' ' 1
##
## $PSCO_vs_STCA
##          Df SumOfSqs      R2      F Pr(>F)
## Species   1   1.5368 0.20682 4.6935  0.001 ***
## Residual 18   5.8940 0.79318
## Total    19   7.4308 1.00000
## ---
## Signif. codes:  0 '***' 0.001 '**' 0.01 '*' 0.05 '.' 0.1 ' ' 1
##
## $PSCO_vs_CHSE
##          Df SumOfSqs      R2      F Pr(>F)
## Species   1   0.8146 0.15341 2.3557  0.004 **
## Residual 13   4.4955 0.84659
## Total    14   5.3102 1.00000
## ---
## Signif. codes:  0 '***' 0.001 '**' 0.01 '*' 0.05 '.' 0.1 ' ' 1
##
## $STCA_vs_CHSE
##          Df SumOfSqs      R2      F Pr(>F)
## Species   1   0.8460 0.23488 2.7628  0.003 **
## Residual  9   2.7558 0.76512
## Total    10   3.6018 1.00000
## ---
## Signif. codes:  0 '***' 0.001 '**' 0.01 '*' 0.05 '.' 0.1 ' ' 1
##
## attr(,"class")
## [1] "pwadstrata" "list"
```

```
pairwise.adonis2(carapace_jacc ~ Site, data = sampledf, p.adjust.m="BH")
```

```
## $parent_call
## [1] "carapace_jacc ~ Site , strata = Null , permutations 999"
##
## $BP4_vs_S4
##          Df SumOfSqs      R2      F Pr(>F)
```

```
## Site      1   1.0013 0.03729 2.5564  0.002 **
## Residual 66  25.8496 0.96271
## Total    67  26.8509 1.00000
## ---
## Signif. codes:  0 '***' 0.001 '**' 0.01 '*' 0.05 '.' 0.1 ' ' 1
##
## $BP4_vs_S1
##          Df SumOfSqs      R2      F Pr(>F)
## Site      1   1.7468 0.07269 4.4684  0.001 ***
## Residual 57  22.2833 0.92731
## Total    58  24.0301 1.00000
## ---
## Signif. codes:  0 '***' 0.001 '**' 0.01 '*' 0.05 '.' 0.1 ' ' 1
##
## $S4_vs_S1
##          Df SumOfSqs      R2      F Pr(>F)
## Site      1   1.4486 0.09178 3.9412  0.001 ***
## Residual 39  14.3343 0.90822
## Total    40  15.7829 1.00000
## ---
## Signif. codes:  0 '***' 0.001 '**' 0.01 '*' 0.05 '.' 0.1 ' ' 1
##
## attr(,"class")
## [1] "pwadstrata" "list"
```

```
pairwise.adonis2(carapace_uni ~ Species, data = sampledf, p.adjust.m="BH")
```

```
## $parent_call
## [1] "carapace_uni ~ Species , strata = Null , permutations 999"
##
## $KISU_vs_STOD
##          Df SumOfSqs      R2      F Pr(>F)
## Species   1   1.1586 0.10653 4.4114  0.001 ***
## Residual 37   9.7173 0.89347
## Total    38  10.8759 1.00000
## ---
## Signif. codes:  0 '***' 0.001 '**' 0.01 '*' 0.05 '.' 0.1 ' ' 1
##
## $KISU_vs_TRSC
##          Df SumOfSqs      R2      F Pr(>F)
## Species   1   1.2406 0.12388 4.8073  0.001 ***
## Residual 34   8.7743 0.87612
## Total    35  10.0148 1.00000
## ---
## Signif. codes:  0 '***' 0.001 '**' 0.01 '*' 0.05 '.' 0.1 ' ' 1
##
## $KISU_vs_PSCO
##          Df SumOfSqs      R2      F Pr(>F)
## Species   1   1.0383 0.14947 4.2178  0.001 ***
## Residual 24   5.9078 0.85053
## Total    25   6.9461 1.00000
## ---
## Signif. codes:  0 '***' 0.001 '**' 0.01 '*' 0.05 '.' 0.1 ' ' 1
##
```

```
## $KISU_vs_STCA
##          Df SumOfSqs      R2    F Pr(>F)
## Species   1   1.4201 0.22118 5.68  0.001 ***
## Residual 20   5.0003 0.77882
## Total    21   6.4204 1.00000
## ---
## Signif. codes:  0 '***' 0.001 '**' 0.01 '*' 0.05 '.' 0.1 ' ' 1
##
## $KISU_vs_CHSE
##          Df SumOfSqs      R2      F Pr(>F)
## Species   1   0.5417 0.11846 2.0158  0.001 ***
## Residual 15   4.0309 0.88154
## Total    16   4.5725 1.00000
## ---
## Signif. codes:  0 '***' 0.001 '**' 0.01 '*' 0.05 '.' 0.1 ' ' 1
##
## $STOD_vs_TRSC
##          Df SumOfSqs      R2      F Pr(>F)
## Species   1   1.5136 0.11804 6.0227  0.001 ***
## Residual 45  11.3089 0.88196
## Total    46  12.8225 1.00000
## ---
## Signif. codes:  0 '***' 0.001 '**' 0.01 '*' 0.05 '.' 0.1 ' ' 1
##
## $STOD_vs_PSCO
##          Df SumOfSqs      R2      F Pr(>F)
## Species   1   1.4448 0.14613 5.9898  0.001 ***
## Residual 35   8.4425 0.85387
## Total    36   9.8873 1.00000
## ---
## Signif. codes:  0 '***' 0.001 '**' 0.01 '*' 0.05 '.' 0.1 ' ' 1
##
## $STOD_vs_STCA
##          Df SumOfSqs      R2      F Pr(>F)
## Species   1   1.3077 0.14788 5.3799  0.001 ***
## Residual 31   7.5350 0.85212
## Total    32   8.8426 1.00000
## ---
## Signif. codes:  0 '***' 0.001 '**' 0.01 '*' 0.05 '.' 0.1 ' ' 1
##
## $STOD_vs_CHSE
##          Df SumOfSqs      R2     F Pr(>F)
## Species   1   0.3447 0.04988 1.365  0.056 .
## Residual 26   6.5655 0.95012
## Total    27   6.9102 1.00000
## ---
## Signif. codes:  0 '***' 0.001 '**' 0.01 '*' 0.05 '.' 0.1 ' ' 1
##
## $TRSC_vs_PSCO
##          Df SumOfSqs      R2      F Pr(>F)
## Species   1   0.6248 0.07691 2.6661  0.001 ***
## Residual 32   7.4994 0.92309
## Total    33   8.1242 1.00000
## ---
```

```
## Signif. codes:  0 '***' 0.001 '**' 0.01 '*' 0.05 '.' 0.1 ' ' 1
##
## $TRSC_vs_STCA
##          Df SumOfSqs      R2      F Pr(>F)
## Species   1   1.3190 0.16673 5.6027  0.001 ***
## Residual 28   6.5919 0.83327
## Total    29   7.9110 1.00000
## ---
## Signif. codes:  0 '***' 0.001 '**' 0.01 '*' 0.05 '.' 0.1 ' ' 1
##
## $TRSC_vs_CHSE
##          Df SumOfSqs      R2      F Pr(>F)
## Species   1   0.4282 0.07076 1.7514  0.011 *
## Residual 23   5.6225 0.92924
## Total    24   6.0506 1.00000
## ---
## Signif. codes:  0 '***' 0.001 '**' 0.01 '*' 0.05 '.' 0.1 ' ' 1
##
## $PSCO_vs_STCA
##          Df SumOfSqs      R2      F Pr(>F)
## Species   1   1.4637 0.28206 7.0719  0.001 ***
## Residual 18   3.7255 0.71794
## Total    19   5.1892 1.00000
## ---
## Signif. codes:  0 '***' 0.001 '**' 0.01 '*' 0.05 '.' 0.1 ' ' 1
##
## $PSCO_vs_CHSE
##          Df SumOfSqs      R2      F Pr(>F)
## Species   1   0.5261 0.16028 2.4814   0.01 **
## Residual 13   2.7560 0.83972
## Total    14   3.2821 1.00000
## ---
## Signif. codes:  0 '***' 0.001 '**' 0.01 '*' 0.05 '.' 0.1 ' ' 1
##
## $STCA_vs_CHSE
##          Df SumOfSqs      R2      F Pr(>F)
## Species   1  0.71967 0.28022 3.5039  0.006 **
## Residual  9  1.84854 0.71978
## Total    10  2.56821 1.00000
## ---
## Signif. codes:  0 '***' 0.001 '**' 0.01 '*' 0.05 '.' 0.1 ' ' 1
##
## attr(,"class")
## [1] "pwadstrata" "list"
```

```
pairwise.adonis2(carapace_uni ~ Site, data = sampledf, p.adjust.m="BH")
```

```
## $parent_call
## [1] "carapace_uni ~ Site , strata = Null , permutations 999"
##
## $BP4_vs_S4
##          Df SumOfSqs      R2      F Pr(>F)
## Site      1   0.7595 0.04071 2.8007  0.001 ***
## Residual 66  17.8978 0.95929
```

```
## Total    67  18.6573 1.00000
## ---
## Signif. codes:  0 '***' 0.001 '**' 0.01 '*' 0.05 '.' 0.1 ' ' 1
##
## $BP4_vs_S1
##          Df SumOfSqs      R2      F Pr(>F)
## Site      1   2.0012 0.11759 7.5959  0.001 ***
## Residual 57  15.0172 0.88241
## Total    58  17.0184 1.00000
## ---
## Signif. codes:  0 '***' 0.001 '**' 0.01 '*' 0.05 '.' 0.1 ' ' 1
##
## $S4_vs_S1
##          Df SumOfSqs     R2      F Pr(>F)
## Site      1   1.4848 0.1242 5.5305  0.001 ***
## Residual 39  10.4701 0.8758
## Total    40  11.9549 1.0000
## ---
## Signif. codes:  0 '***' 0.001 '**' 0.01 '*' 0.05 '.' 0.1 ' ' 1
##
## attr(,"class")
## [1] "pwadstrata" "list"
```

```
pairwise.adonis2(carapace_wuni ~ Species, data = sampledf, p.adjust.m="BH")
```

```
## $parent_call
## [1] "carapace_wuni ~ Species , strata = Null , permutations 999"
##
## $KISU_vs_STOD
##          Df SumOfSqs      R2      F Pr(>F)
## Species   1 0.006663 0.10348 4.2709  0.002 **
## Residual 37 0.057725 0.89652
## Total    38 0.064389 1.00000
## ---
## Signif. codes:  0 '***' 0.001 '**' 0.01 '*' 0.05 '.' 0.1 ' ' 1
##
## $KISU_vs_TRSC
##          Df SumOfSqs      R2      F Pr(>F)
## Species   1 0.011910 0.18604 7.7713  0.001 ***
## Residual 34 0.052107 0.81396
## Total    35 0.064017 1.00000
## ---
## Signif. codes:  0 '***' 0.001 '**' 0.01 '*' 0.05 '.' 0.1 ' ' 1
##
## $KISU_vs_PSCO
##          Df  SumOfSqs      R2      F Pr(>F)
## Species   1 0.0048265 0.19167 5.6907  0.001 ***
## Residual 24 0.0203553 0.80833
## Total    25 0.0251818 1.00000
## ---
## Signif. codes:  0 '***' 0.001 '**' 0.01 '*' 0.05 '.' 0.1 ' ' 1
##
## $KISU_vs_STCA
##          Df  SumOfSqs      R2      F Pr(>F)
```

```
## Species   1 0.0060251 0.31706 9.2853  0.001 ***
## Residual 20 0.0129778 0.68294
## Total    21 0.0190029 1.00000
## ---
## Signif. codes:  0 '***' 0.001 '**' 0.01 '*' 0.05 '.' 0.1 ' ' 1
##
## $KISU_vs_CHSE
##          Df  SumOfSqs      R2      F Pr(>F)
## Species   1 0.0038731 0.26564 5.4259  0.001 ***
## Residual 15 0.0107073 0.73436
## Total    16 0.0145804 1.00000
## ---
## Signif. codes:  0 '***' 0.001 '**' 0.01 '*' 0.05 '.' 0.1 ' ' 1
##
## $STOD_vs_TRSC
##          Df SumOfSqs      R2      F Pr(>F)
## Species   1 0.012837 0.11804 6.0227  0.001 ***
## Residual 45 0.095918 0.88196
## Total    46 0.108755 1.00000
## ---
## Signif. codes:  0 '***' 0.001 '**' 0.01 '*' 0.05 '.' 0.1 ' ' 1
##
## $STOD_vs_PSCO
##          Df SumOfSqs      R2     F Pr(>F)
## Species   1 0.010015 0.13501 5.463  0.001 ***
## Residual 35 0.064166 0.86499
## Total    36 0.074181 1.00000
## ---
## Signif. codes:  0 '***' 0.001 '**' 0.01 '*' 0.05 '.' 0.1 ' ' 1
##
## $STOD_vs_STCA
##          Df SumOfSqs      R2      F Pr(>F)
## Species   1 0.011470 0.16804 6.2614  0.001 ***
## Residual 31 0.056788 0.83196
## Total    32 0.068259 1.00000
## ---
## Signif. codes:  0 '***' 0.001 '**' 0.01 '*' 0.05 '.' 0.1 ' ' 1
##
## $STOD_vs_CHSE
##          Df SumOfSqs      R2      F Pr(>F)
## Species   1 0.002868 0.04998 1.3679  0.182
## Residual 26 0.054518 0.95002
## Total    27 0.057386 1.00000
##
## $TRSC_vs_PSCO
##          Df SumOfSqs      R2      F Pr(>F)
## Species   1 0.006635 0.10179 3.6262  0.004 **
## Residual 32 0.058548 0.89821
## Total    33 0.065182 1.00000
## ---
## Signif. codes:  0 '***' 0.001 '**' 0.01 '*' 0.05 '.' 0.1 ' ' 1
##
## $TRSC_vs_STCA
##          Df SumOfSqs      R2     F Pr(>F)
```

```
## Species   1 0.013882 0.21339 7.596  0.001 ***
## Residual 28 0.051170 0.78661
## Total    29 0.065052 1.00000
## ---
## Signif. codes:  0 '***' 0.001 '**' 0.01 '*' 0.05 '.' 0.1 ' ' 1
##
## $TRSC_vs_CHSE
##          Df SumOfSqs      R2      F Pr(>F)
## Species   1 0.003792 0.07196 1.7834  0.082 .
## Residual 23 0.048900 0.92804
## Total    24 0.052691 1.00000
## ---
## Signif. codes:  0 '***' 0.001 '**' 0.01 '*' 0.05 '.' 0.1 ' ' 1
##
## $PSCO_vs_STCA
##          Df  SumOfSqs      R2      F Pr(>F)
## Species   1 0.0080797 0.29383 7.4895  0.001 ***
## Residual 18 0.0194184 0.70617
## Total    19 0.0274980 1.00000
## ---
## Signif. codes:  0 '***' 0.001 '**' 0.01 '*' 0.05 '.' 0.1 ' ' 1
##
## $PSCO_vs_CHSE
##          Df  SumOfSqs     R2      F Pr(>F)
## Species   1 0.0039807 0.1884 3.0178  0.011 *
## Residual 13 0.0171479 0.8116
## Total    14 0.0211286 1.0000
## ---
## Signif. codes:  0 '***' 0.001 '**' 0.01 '*' 0.05 '.' 0.1 ' ' 1
##
## $STCA_vs_CHSE
##          Df  SumOfSqs      R2     F Pr(>F)
## Species   1 0.0056907 0.36807 5.242  0.011 *
## Residual  9 0.0097704 0.63193
## Total    10 0.0154610 1.00000
## ---
## Signif. codes:  0 '***' 0.001 '**' 0.01 '*' 0.05 '.' 0.1 ' ' 1
##
## attr(,"class")
## [1] "pwadstrata" "list"
```

```
pairwise.adonis2(carapace_wuni ~ Site, data = sampledf, p.adjust.m="BH")
```

```
## $parent_call
## [1] "carapace_wuni ~ Site , strata = Null , permutations 999"
##
## $BP4_vs_S4
##          Df SumOfSqs      R2      F Pr(>F)
## Site      1 0.003852 0.03147 2.1445  0.032 *
## Residual 66 0.118561 0.96853
## Total    67 0.122414 1.00000
## ---
## Signif. codes:  0 '***' 0.001 '**' 0.01 '*' 0.05 '.' 0.1 ' ' 1
##
```

```
## $BP4_vs_S1
##          Df SumOfSqs      R2      F Pr(>F)
## Site      1 0.010276 0.08609 5.3696  0.002 **
## Residual 57 0.109086 0.91391
## Total    58 0.119362 1.00000
## ---
## Signif. codes:  0 '***' 0.001 '**' 0.01 '*' 0.05 '.' 0.1 ' ' 1
##
## $S4_vs_S1
##          Df SumOfSqs      R2      F Pr(>F)
## Site      1 0.008670 0.10024 4.3448  0.002 **
## Residual 39 0.077827 0.89976
## Total    40 0.086497 1.00000
## ---
## Signif. codes:  0 '***' 0.001 '**' 0.01 '*' 0.05 '.' 0.1 ' ' 1
##
## attr(,"class")
## [1] "pwadstrata" "list"
```

ANOSIM: analysis of similarity testing

For anosim tests, values range from -1 to +1. Values closer to +1 indicate stronger similarities within groups compared to between groups. Values close to zero indicates no difference between groups. Anosim tests are done for each beta diversity metric, first for turtle species and then by collection site.

```
anosim(carapace_bray, sampledf$Species, permutations = 999)
```

```
##
## Call:
## anosim(x = carapace_bray, grouping = sampledf$Species, permutations = 999)
## Dissimilarity: bray
##
## ANOSIM statistic R: 0.7189
##       Significance: 0.001
##
## Permutation: free
## Number of permutations: 999
```

```
anosim(carapace_jacc, sampledf$Species, permutations = 999)
```

```
##
## Call:
## anosim(x = carapace_jacc, grouping = sampledf$Species, permutations = 999)
## Dissimilarity: jaccard
##
## ANOSIM statistic R: 0.7189
##       Significance: 0.001
##
## Permutation: free
## Number of permutations: 999
```

```r
anosim(carapace_uni, sampledf$Species, permutations = 999)
```

```
##
## Call:
## anosim(x = carapace_uni, grouping = sampledf$Species, permutations = 999)
## Dissimilarity:
##
## ANOSIM statistic R: 0.4678
##       Significance: 0.001
##
## Permutation: free
## Number of permutations: 999
```

```r
anosim(carapace_wuni, sampledf$Species, permutations = 999)
```

```
##
## Call:
## anosim(x = carapace_wuni, grouping = sampledf$Species, permutations = 999)
## Dissimilarity:
##
## ANOSIM statistic R: 0.1867
##       Significance: 0.001
##
## Permutation: free
## Number of permutations: 999
```

```r
anosim(carapace_bray, sampledf$Site, permutations = 999)
```

```
##
## Call:
## anosim(x = carapace_bray, grouping = sampledf$Site, permutations = 999)
## Dissimilarity: bray
##
## ANOSIM statistic R: 0.2388
##       Significance: 0.001
##
## Permutation: free
## Number of permutations: 999
```

```r
anosim(carapace_jacc, sampledf$Site, permutations = 999)
```

```
##
## Call:
## anosim(x = carapace_jacc, grouping = sampledf$Site, permutations = 999)
## Dissimilarity: jaccard
##
## ANOSIM statistic R: 0.2388
##       Significance: 0.001
##
## Permutation: free
## Number of permutations: 999
```

```
anosim(carapace_uni, sampledf$Site, permutations = 999)
```

```
##
## Call:
## anosim(x = carapace_uni, grouping = sampledf$Site, permutations = 999)
## Dissimilarity:
##
## ANOSIM statistic R: 0.3759
##       Significance: 0.001
##
## Permutation: free
## Number of permutations: 999
```

```
anosim(carapace_wuni, sampledf$Site, permutations = 999)
```

```
##
## Call:
## anosim(x = carapace_wuni, grouping = sampledf$Site, permutations = 999)
## Dissimilarity:
##
## ANOSIM statistic R: 0.1707
##       Significance: 0.002
##
## Permutation: free
## Number of permutations: 999
```

generate UpSetR plots

UpSetR plots are generated in place of Venn diagrams to enable effective comparison between all six turtle species. UpSetR plots for collection sites are also generated.

```
library(UpSetR)
library(gridExtra)
library(methods)
library(grDevices)
library(mltools)
library(data.table)

setwd("/Users/mparks10/Desktop/atoka.2021_2022.combined/phyloseq")
data = read.csv('16S.OTU_table.0_1.txt', na.strings="Not Applicable")
newdata <- one_hot(as.data.table(data))
#with numbers over bars
upset(newdata, nsets=6, order.by="freq", mainbar.y.label = "# ASV in Intersection", sets.x.label = "# AS
```

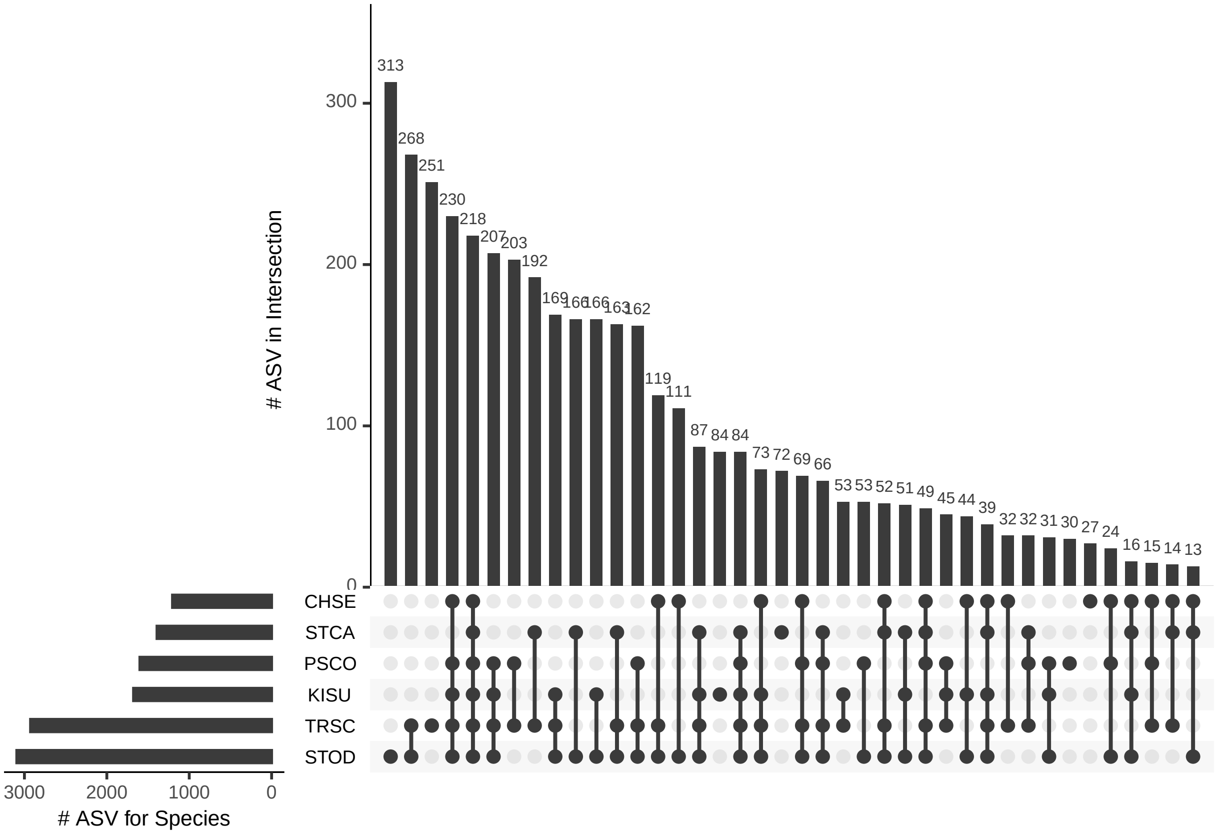


```
#without numbers over bars
#upset(newdata, nsets=6, order.by="freq", mainbar.y.label = "# ASV in Intersection", sets.x.label = "# A

data = read.csv('16S.OTU_site_table.0_1.txt', na.strings="Not Applicable")#install packages (can skip i
newdata <- one_hot(as.data.table(data))#create the visualization
#with numbers over bars
upset(newdata, nsets=3, order.by="freq", mainbar.y.label = "# ASV in Intersection", sets.x.label = "# AS
```

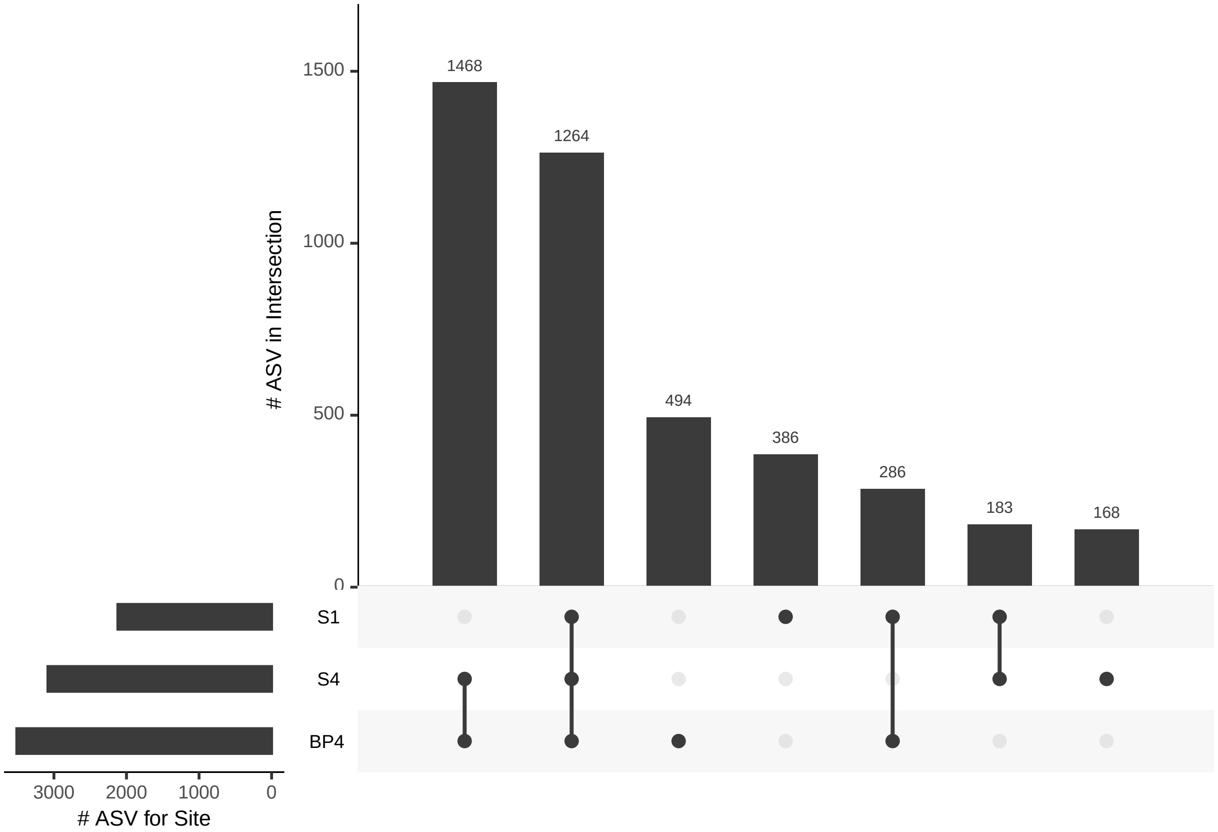


```
#without numbers over bars
#upset(newdata, nsets=3, order.by="freq", mainbar.y.label = "# ASV in Intersection", sets.x.label = "#
```

18S rRNA gene analyses

Load libraries for analysis

Here all necessary libraries are loaded for statistical analyses, with the exception of UpSet Plot-related libraries. The latter may cause problems with the initial set of libraries, so they are loaded and UpSet plots are generated at the end of the R markdown. The library easystats may be updated as necessary along with other libraries.

library(vegan) library(ggplot2) library(usedist) library(car) library(performance) library(emmeans) library(plyr) library(lme4) library(splitstackshape) library(kableExtra) library(ggpubr) library(gtable) library(grid) library(gridExtra) #easystats::easystats_update() library(easystats) library(see) library(phyloseq) library(microViz) library(pairwiseAdonis) library(btools) library(olsrr)

Add simplifying code

This code snippet simplifies subsequent code.

```
#simplification coding:
#wrapper function with settings used for tables throughout. This simplifies the code elsewhere
# x is the object o be turned into a kable
# caption is the caption
```

```r
# row.names - determines whether to include row names (NA is default for kbl)
kable.wrap <- function(x,caption,row.names=NA){
  kable_classic(full_width = F,html_font = "Calibri",font_size = 18,kbl(x,align="c",caption=caption,row
}
```

Set the working directory and phyloseq objects

The code below sets working directory and assigns phyloseq objects. Working directory would need to be adjusted for other users/computers.

```r
#setting up directory and clarifying metadata
setwd("/Users/mparks10/Desktop/atoka.2021_2022.combined/phyloseq")
meta <- read.csv("Atoka_2021_2022.18S.metadata.w_morphology.csv")

meta$Carapace_length <- as.numeric(as.character(meta$Carapace_length))
```

```
## Warning: NAs introduced by coercion
```

```r
meta$Plastron_length <- as.numeric(as.character(meta$Plastron_length))
```

```
## Warning: NAs introduced by coercion
```

```r
meta$Mass <- as.numeric(as.character(meta$Mass))
```

```
## Warning: NAs introduced by coercion
```

```r
#sapply(meta, class)

rownames(meta) <- meta$sample.ID
meta.phylo <- sample_data(meta)

# function for normalizing to proportions
prop.trans <- function(x){x/sum(x)}

data <- read.csv("18S.asv.table.filter.cleaned.csv")
rownames(data) <- data[,1]
data <- data[,2:(ncol(data)-1)]
data <- as.matrix(data)

data <- apply(data,2,prop.trans) #convert to proportions
asv <- otu_table(data,taxa_are_rows = T)

#reading in numerical data (i.e., integer counts rather than proportions)

data_np <- read.csv("18S.asv.table.filter.cleaned.csv") #note suffice 'np' means 'not proportions', ind
rownames(data_np) <- data_np[,1]
data_np <- data_np[,2:(ncol(data_np)-1)]
data_np <- as.matrix(data_np)
asv_np <- otu_table(data_np,taxa_are_rows = T)
```

```
#reading in taxon information
#in this file labels for 'unknown' have been replaced with "unknown_ ..." where "..." = the lowest taxo
taxa <- read.csv("18S.taxa_filter.unknown.cleaned.csv")
rownames(taxa) <- taxa[,1]
taxa <- as.matrix(taxa[,-1])
colnames(taxa)[7] <- "ASV"
taxa <- tax_table(taxa)

# load rooted tree file and trim
tree <- read_tree("18S.rooted.tree.nwk")
tree <- prune_taxa(rownames(asv),tree)

# convert to phyloseq, tax_fix can be used to adjust any non-conforming taxon names
phylo <- merge_phyloseq(taxa,asv,tree,meta.phylo)
phylo_np <- merge_phyloseq(taxa,asv_np,tree,meta.phylo)
phylo<-tax_fix(phylo)
phylo_np<-tax_fix(phylo_np)
```

Estimating alpha diversity

The code below calculates different measures of alpha diversity to be used in later analysis.

```
richness <- estimate_richness(phylo_np,measures=c("Observed","Chao1","ACE","Shannon","Simpson","InvSimps
richness
```

```
##          Observed     Chao1   se.chao1       ACE    se.ACE      Shannon
## Sample2         8   8.00000 0.00000000   8.00000 1.2247449 1.804599e+00
## Sample13       30  30.00000 0.00000000  30.00000 2.3166067 2.599252e+00
## Sample20       87  87.00000 0.00000000  87.00000 3.8536443 2.125789e+00
## Sample22       66  66.00000 0.00000000  66.00000 3.2310051 1.441145e+00
## Sample33       40  40.00000 0.00000000  40.00000 2.5298221 3.064440e+00
## Sample36       30  30.00000 0.00000000  30.00000 2.3166067 9.935898e-01
## Sample37       36  36.25000 0.73503387  37.43548 2.7473800 1.726192e+00
## Sample38       47  47.50000 1.29447205  47.54976 3.2996717 2.318752e+00
## Sample3         6   6.00000 0.00000000       NaN       NaN 1.626200e+00
## Sample18      101 101.00000 0.09950372 101.23279 4.3163863 1.669817e+00
## Sample39       87  87.00000 0.49711813  87.16877 3.7479232 2.689470e+00
## Sample19      104 104.00000 0.16586345 104.29909 3.7524782 2.080386e+00
## Sample24       43  43.00000 0.49415185  43.24206 2.6298078 1.763660e+00
## Sample21       73  73.00000 0.12414088  73.27597 3.7330098 2.076156e+00
## Sample23       64  64.00000 0.00000000  64.00000 2.6457513 1.211299e+00
## Sample14       57  57.00000 0.00000000  57.00000 2.7529888 1.591640e+00
## Sample25       37  37.00000 0.49319696  37.21770 2.6703005 1.769365e+00
## Sample4        14  14.00000 0.00000000  14.00000 1.5352989 1.339764e-05
## Sample5        14  14.00000 0.00000000  14.00000 1.5352989 1.339764e-05
## Sample26       47  48.00000 2.32737334  48.14325 2.5443733 1.070759e+00
## Sample15       71  71.33333 0.92480028  71.86764 3.1068834 1.060197e+00
## Sample27       20  20.00000 0.00000000  20.00000 2.0493902 3.938978e-01
## Sample16       87  87.00000 0.12427953  87.31590 4.1006093 1.676226e+00
## Sample28       51  51.00000 0.00000000  51.00000 3.0292690 7.346174e-01
## Sample29       40  41.00000 2.32431474  40.82662 2.6006887 7.065450e-01
## Sample30       52  52.00000 0.00000000  52.00000 2.9450088 1.069891e+00
```

```
## Sample6          17  17.00000 0.00000000  17.00000 1.5718105 9.199626e-01
## Sample7          17  17.00000 0.00000000  17.00000 1.5718105 9.199626e-01
## Sample31         37  37.00000 0.00000000  37.00000 2.7013510 5.567955e-01
## Sample34         95  95.33333 0.92517711  95.60860 4.2108246 2.839401e+00
## Sample35         48  48.00000 0.00000000  48.00000 2.7041635 1.020304e+00
## Sample40         81  81.00000 0.24845200  81.18806 3.5468448 3.026836e+00
## Sample32         70  70.50000 1.29596345  70.51641 3.6797438 2.290767e+00
## Sample17         90  90.00000 0.00000000  90.00000 4.0124805 2.292587e+00
## Sample9          28  28.00000 0.00000000  28.00000 2.2912878 1.610036e+00
## Sample8          11  11.00000 0.00000000  11.00000 1.6514456 1.739963e+00
## Sample10          8   8.00000 0.00000000   8.00000 1.3693064 1.098719e+00
## Sample11          5   5.00000 0.00000000   5.00000 0.8944272 1.255970e+00
## Sample12          2   2.00000 0.00000000   2.00000 0.7071068 1.351198e-02
## Sample67         14  14.00000 0.00000000       NaN       NaN 2.501977e-01
## Sample104        14  14.00000 0.00000000       NaN       NaN 2.501977e-01
## Sample41        101 101.00000 0.16583953 101.23092 3.8129251 1.933277e+00
## Sample42         18  18.00000 0.00000000  18.00000 1.5811388 4.208576e-01
## Sample43         89  89.00000 0.00000000  89.00000 3.6226503 2.409729e+00
## Sample44         18  18.00000 0.00000000  18.00000 1.5811388 1.331851e+00
## Sample45         69  69.00000 0.49636359  69.24119 3.4773422 1.656993e+00
## Sample46         51  51.00000 0.00000000  51.00000 3.3665016 1.067337e+00
## Sample47         74  74.00000 0.00000000  74.00000 4.0269363 2.554079e+00
## Sample48         49  52.00000 4.62175042  50.63324 2.5165187 2.201667e+00
## Sample49         70  70.00000 0.00000000  70.00000 2.6619005 2.688619e+00
## Sample50         60  60.00000 0.00000000  60.00000 2.4866309 1.246383e+00
## Sample51         42  42.00000 0.00000000  42.00000 2.4152295 1.410278e+00
## Sample52         53  53.00000 0.00000000  53.00000 3.3981127 4.261084e-01
## Sample53         40  40.00000 0.00000000  40.00000 2.8240042 2.233383e+00
## Sample54         70  70.50000 1.29596345  70.61881 3.4664859 1.416831e+00
## Sample55         22  22.00000 0.00000000  22.00000 1.6096301 1.372771e+00
## Sample56         31  31.00000 0.00000000  31.00000 2.1997067 1.730620e+00
## Sample57        120 123.00000 4.64600537 120.95765 3.7448337 3.190298e+00
## Sample58        123 123.00000 0.00000000 123.00000 4.4630370 3.096025e+00
## Sample59        107 107.00000 0.49765807 107.18781 3.2238978 2.233356e+00
## Sample60        130 130.00000 0.09961464 130.28081 3.9134132 3.102306e+00
## Sample61        104 104.00000 0.49759035 104.18670 3.6477404 2.985968e+00
## Sample62         36  36.00000 0.00000000  36.00000 2.5980762 1.778615e+00
## Sample63         52  52.00000 0.00000000  52.00000 2.9450088 2.439459e+00
## Sample64         13  13.00000 0.00000000  13.00000 0.9607689 9.009753e-01
## Sample65         29  29.00000 0.00000000  29.00000 2.6781363 1.003663e+00
## Sample66         26  26.00000 0.00000000  26.00000 2.0095924 1.117192e+00
## Sample68         74  74.00000 0.00000000  74.00000 3.0602067 2.687905e+00
## Sample69         47  48.00000 2.32737334  47.64394 3.0123045 2.089426e+00
## Sample70         31  31.00000 0.00000000  31.00000 2.5272706 1.884655e+00
## Sample71         27  27.00000 0.00000000  27.00000 2.2771002 1.746269e+00
## Sample72         15  15.00000 0.00000000  15.00000 1.7126977 2.074205e+00
## Sample73         88  88.00000 0.00000000  88.00000 4.0620192 1.077388e+00
## Sample74         29  29.00000 0.00000000  29.00000 2.4068867 1.304946e+00
## Sample75         43  43.00000 0.00000000  43.00000 2.2721775 2.044749e+00
## Sample76         35  35.00000 0.00000000  35.00000 2.4842360 1.543661e+00
## Sample77         45  45.00000 0.00000000  45.00000 2.9664794 1.227475e+00
## Sample78         18  18.00000 0.48591266  18.27242 2.0849273 9.315579e-01
## Sample79         32  32.00000 0.00000000  32.00000 2.6220221 1.326497e+00
## Sample80         16  16.00000 0.00000000  16.00000 1.7320508 3.824934e-01
```

```
## Sample81        44  44.00000 0.49428553  44.20106 2.8451758 4.382773e-01
## Sample82        74  74.00000 0.24830507  74.27013 3.2430240 1.473129e+00
## Sample83        70  70.00000 0.00000000  70.00000 3.5132403 6.658754e-01
## Sample84        49  49.00000 0.00000000  49.00000 3.1622777 9.600715e-01
## Sample85        51  52.00000 2.32875008  51.69756 2.8880009 5.410910e-01
## Sample86         9   9.00000 0.00000000   9.00000 1.4142136 6.040455e-01
## Sample87        13  13.00000 0.00000000  13.00000 1.7541160 8.840481e-01
## Sample88        17  17.00000 0.00000000  17.00000 1.8786729 6.009443e-01
## Sample89        16  16.00000 0.00000000  16.00000 1.9364917 9.944296e-01
## Sample90        32  32.00000 0.00000000  32.00000 2.7386128 5.758058e-01
## Sample91        58  58.00000 0.16522364  58.40422 3.2437336 2.140760e+00
## Sample92        66  66.00000 0.09923953  66.27089 3.8579628 8.519245e-01
## Sample93        49  49.00000 0.16495722  49.25740 3.1360163 6.125096e-01
## Sample94        63  63.50000 1.29562407  63.40589 3.6797889 4.821666e-01
## Sample95        38  38.00000 0.00000000  38.00000 2.5131234 1.441316e+00
## Sample96         9   9.00000 0.00000000   9.00000 1.4142136 1.855970e-01
## Sample97        24  24.00000 0.00000000  24.00000 2.4409698 1.202992e+00
## Sample98        14  14.00000 0.00000000  14.00000 1.5352989 8.028782e-01
## Sample99        26  27.00000 2.31340698  26.57709 2.5316679 7.141670e-01
## Sample100       12  12.00000 0.00000000  12.00000 1.5000000 1.939317e-02
## Sample101       20  20.00000 0.00000000  20.00000 1.9364917 1.308178e-01
## Sample102       32  32.50000 1.29235920  32.61896 2.8108829 1.176881e+00
## Sample103       39  39.00000 0.49354812  39.20972 3.0081273 1.006804e+00
##                Simpson InvSimpson
## Sample2   8.062601e-01   5.161559
## Sample13  8.714683e-01   7.780185
## Sample20  7.265054e-01   3.656380
## Sample22  4.670017e-01   1.876179
## Sample33  9.182640e-01  12.234517
## Sample36  3.942086e-01   1.650733
## Sample37  6.779211e-01   3.104829
## Sample38  8.298166e-01   5.876013
## Sample3   7.780214e-01   4.504938
## Sample18  6.248716e-01   2.665754
## Sample39  8.510617e-01   6.714189
## Sample19  7.016622e-01   3.351906
## Sample24  7.679359e-01   4.309154
## Sample21  7.067076e-01   3.409567
## Sample23  4.555819e-01   1.836824
## Sample14  6.679648e-01   3.011729
## Sample25  7.108941e-01   3.458940
## Sample4   1.576558e-06   1.000002
## Sample5   1.576558e-06   1.000002
## Sample26  4.645454e-01   1.867572
## Sample15  3.457556e-01   1.528481
## Sample27  1.429102e-01   1.166739
## Sample16  6.287627e-01   2.693695
## Sample28  2.628979e-01   1.356664
## Sample29  2.496440e-01   1.332701
## Sample30  4.393947e-01   1.783786
## Sample6   5.463829e-01   2.204502
## Sample7   5.463829e-01   2.204502
## Sample31  1.940048e-01   1.240702
## Sample34  8.601863e-01   7.152372
```

```
## Sample35  3.341887e-01   1.501927
## Sample40  9.101736e-01  11.132579
## Sample32  8.019268e-01   5.048638
## Sample17  7.988467e-01   4.971332
## Sample9   6.484020e-01   2.844158
## Sample8   7.728209e-01   4.401813
## Sample10  5.074679e-01   2.030325
## Sample11  6.433986e-01   2.804251
## Sample12  3.700265e-03   1.003714
## Sample67  1.278364e-01   1.146574
## Sample104 1.278364e-01   1.146574
## Sample41  6.678934e-01   3.011081
## Sample42  1.613124e-01   1.192339
## Sample43  8.199319e-01   5.553454
## Sample44  6.005542e-01   2.503469
## Sample45  6.983508e-01   3.315109
## Sample46  4.905636e-01   1.962954
## Sample47  8.802455e-01   8.350419
## Sample48  8.281661e-01   5.819574
## Sample49  8.597991e-01   7.132622
## Sample50  4.507075e-01   1.820524
## Sample51  6.021265e-01   2.513362
## Sample52  1.528109e-01   1.180374
## Sample53  8.304479e-01   5.897894
## Sample54  5.464230e-01   2.204697
## Sample55  5.181920e-01   2.075516
## Sample56  7.446940e-01   3.916869
## Sample57  9.285254e-01  13.990983
## Sample58  8.929221e-01   9.338995
## Sample59  7.354226e-01   3.779612
## Sample60  9.056968e-01  10.604094
## Sample61  9.076411e-01  10.827324
## Sample62  7.380069e-01   3.816895
## Sample63  8.688152e-01   7.622837
## Sample64  4.152040e-01   1.709998
## Sample65  5.265309e-01   2.112070
## Sample66  4.992768e-01   1.997111
## Sample68  8.784086e-01   8.224267
## Sample69  7.299744e-01   3.703352
## Sample70  6.781252e-01   3.106798
## Sample71  7.456487e-01   3.931571
## Sample72  8.044368e-01   5.113438
## Sample73  3.405533e-01   1.516423
## Sample74  6.862611e-01   3.187364
## Sample75  8.034328e-01   5.087319
## Sample76  7.306737e-01   3.712969
## Sample77  5.409614e-01   2.178466
## Sample78  4.960903e-01   1.984483
## Sample79  6.021315e-01   2.513393
## Sample80  1.380397e-01   1.160146
## Sample81  1.256427e-01   1.143697
## Sample82  6.605398e-01   2.945853
## Sample83  2.003896e-01   1.250609
## Sample84  5.396824e-01   2.172413
```

```
## Sample85  1.744127e-01   1.211259
## Sample86  2.757723e-01   1.380781
## Sample87  4.685735e-01   1.881728
## Sample88  2.805198e-01   1.389892
## Sample89  5.447086e-01   2.196396
## Sample90  1.897201e-01   1.234141
## Sample91  7.777776e-01   4.499997
## Sample92  3.237776e-01   1.478803
## Sample93  2.226131e-01   1.286361
## Sample94  1.304188e-01   1.149979
## Sample95  6.476798e-01   2.838327
## Sample96  6.465566e-02   1.069125
## Sample97  6.434045e-01   2.804298
## Sample98  5.139106e-01   2.057235
## Sample99  3.874779e-01   1.632594
## Sample100 4.268749e-03   1.004287
## Sample101 3.447583e-02   1.035707
## Sample102 5.927415e-01   2.455443
## Sample103 4.284526e-01   1.749636
```

```r
estimate_pd(phylo)
```

```
## Calculating Faiths PD-index...
```

```
##                  PD  SR
## Sample2    3.201427   8
## Sample13   6.672275  30
## Sample20  12.376283  87
## Sample22  12.815638  66
## Sample33   7.395668  40
## Sample36   7.861920  30
## Sample37   6.719728  36
## Sample38   9.088148  47
## Sample3    3.015810   6
## Sample18  15.333446 101
## Sample39  11.835606  87
## Sample19  14.256343 104
## Sample24   8.283373  43
## Sample21  11.011768  73
## Sample23   9.931335  64
## Sample14   7.348046  57
## Sample25   6.285341  37
## Sample4    3.894408  14
## Sample5    3.894408  14
## Sample26   9.359082  47
## Sample15  11.157167  71
## Sample27   6.525874  20
## Sample16  13.281604  87
## Sample28   9.300845  51
## Sample29   5.557910  40
## Sample30   9.446650  52
## Sample6    4.412710  17
## Sample7    4.412710  17
```

```
## Sample31   7.759993  37
## Sample34  11.633775  95
## Sample35   8.968064  48
## Sample40  11.530097  81
## Sample32  10.723880  70
## Sample17  12.572860  90
## Sample9    6.679084  28
## Sample8    3.697611  11
## Sample10   1.796189   8
## Sample11   1.331946   5
## Sample12   1.216489   2
## Sample67   4.024763  14
## Sample104  4.024763  14
## Sample41  14.810360 101
## Sample42   4.799912  18
## Sample43  13.266494  89
## Sample44   4.834226  18
## Sample45  12.010712  69
## Sample46   9.429812  51
## Sample47  10.970226  74
## Sample48   8.950246  49
## Sample49   8.056302  70
## Sample50   8.911418  60
## Sample51   6.854829  42
## Sample52  10.169400  53
## Sample53   6.346057  40
## Sample54  14.528626  70
## Sample55   5.127487  22
## Sample56   7.254231  31
## Sample57  18.063455 120
## Sample58  18.286383 123
## Sample59  19.429675 107
## Sample60  19.536637 130
## Sample61  18.437202 104
## Sample62   7.189657  36
## Sample63   8.981257  52
## Sample64   4.818412  13
## Sample65   7.113173  29
## Sample66   6.316807  26
## Sample68  12.779562  74
## Sample69   8.199459  47
## Sample70   7.688080  31
## Sample71   7.001914  27
## Sample72   5.356128  15
## Sample73  13.578638  88
## Sample74   6.530145  29
## Sample75   8.666320  43
## Sample76   8.275582  35
## Sample77   8.065433  45
## Sample78   5.258696  18
## Sample79   8.420291  32
## Sample80   4.359322  16
## Sample81   9.620990  44
## Sample82  12.822346  74
```

```
## Sample83  11.706936  70
## Sample84  10.483936  49
## Sample85   8.666369  51
## Sample86   2.921782   9
## Sample87   4.255595  13
## Sample88   4.708311  17
## Sample89   5.276630  16
## Sample90   7.402705  32
## Sample91   9.267598  58
## Sample92   9.002706  66
## Sample93   8.358703  49
## Sample94  10.223890  63
## Sample95   7.008309  38
## Sample96   2.674692   9
## Sample97   6.059613  24
## Sample98   3.796045  14
## Sample99   7.035426  26
## Sample100  4.953537  12
## Sample101  5.531198  20
## Sample102  7.628880  32
## Sample103  8.249660  39
```

Setting phyloseq objects to different taxonomic levels

This code sets phyloseq objects consisting of different taxonomic levels, for both the proportional and numeric versions of counts data.

```
phylo.gen <- tax_glom(phylo, "Genus")
phylo_np.gen <- tax_glom(phylo_np, "Genus")

phylo.fam <- tax_glom(phylo, "Family")
phylo_np.fam <- tax_glom(phylo_np, "Family")

phylo.ord <- tax_glom(phylo, "Order")
phylo_np.ord <- tax_glom(phylo_np, "Order")

phylo.class <- tax_glom(phylo, "Class")
phylo_np.class <- tax_glom(phylo_np, "Class")

phylo.phy <- tax_glom(phylo, "Phylum")
phylo_np.phy <- tax_glom(phylo_np, "Phylum")


phylo.list <- list(phylo,phylo.gen,phylo.fam,phylo.ord,phylo.class,phylo.phy)
phylo_np.list <- list(phylo_np,phylo_np.gen,phylo_np.fam,phylo_np.ord,phylo_np.class,phylo_np.phy)

label.list <- list("ASV","Genus","Family","Order","Class","Phylum")
```

Isolate carapace samples for each turtle species

The code below isolates each species' carapace samples and double-checks that the commands worked. These data subsets are used later to check for any effects of plastron length within each species

```r
carapace <- meta[meta$Substrate == "carapace",]
summary(carapace)
```

```
##   sample.ID          LibraryName          Read_depth     ProjectName       
##  Length:84          Length:84          Min.   : 43496   Length:84         
##  Class :character   Class :character   1st Qu.: 70900   Class :character  
##  Mode  :character   Mode  :character   Median : 88460   Mode  :character  
##                                        Mean   : 87626                     
##                                        3rd Qu.:103493                     
##                                        Max.   :174528                     
##     Region          Sample_date             Year          Site          
##  Length:84          Length:84          Min.   :2021   Length:84         
##  Class :character   Class :character   1st Qu.:2021   Class :character  
##  Mode  :character   Mode  :character   Median :2022   Mode  :character  
##                                        Mean   :2022                     
##                                        3rd Qu.:2022                     
##                                        Max.   :2022                     
##  Sample_type         Substrate           Species          Species_substrate 
##  Length:84          Length:84          Length:84          Length:84         
##  Class :character   Class :character   Class :character   Class :character  
##  Mode  :character   Mode  :character   Mode  :character   Mode  :character  
##                                                                             
##                                                                             
##                                                                             
##  Species_site          Sorter              Sex            Carapace_length
##  Length:84          Length:84          Length:84          Min.   : 49.4  
##  Class :character   Class :character   Class :character   1st Qu.: 87.0  
##  Mode  :character   Mode  :character   Mode  :character   Median : 98.0  
##                                                           Mean   :124.8  
##                                                           3rd Qu.:165.7  
##                                                           Max.   :308.0  
##  Plastron_length      Mass         Sample_number       Sex_notes        
##  Min.   : 35.8   Min.   :  25.0   Length:84          Length:84         
##  1st Qu.: 65.0   1st Qu.: 110.0   Class :character   Class :character  
##  Median : 83.3   Median : 170.0   Mode  :character   Mode  :character  
##  Mean   :104.0   Mean   : 492.7                                        
##  3rd Qu.:152.0   3rd Qu.: 682.5                                        
##  Max.   :239.0   Max.   :6600.0                                        
##   PCR1_date         extraction_date       Observed          Chao1       
##  Length:84          Min.   :    2021   Min.   :  9.00   Min.   :  9.00  
##  Class :character   1st Qu.:    2021   1st Qu.: 29.00   1st Qu.: 29.00  
##  Mode  :character   Median :20220620   Median : 47.00   Median : 47.75  
##                     Mean   :14925274   Mean   : 51.45   Mean   : 51.61  
##                     3rd Qu.:20220623   3rd Qu.: 70.25   3rd Qu.: 70.71  
##                     Max.   :20220624   Max.   :130.00   Max.   :130.00  
##     se.chao1           ACE             se.ACE          Shannon      
##  Min.   :0.0000   Min.   :  9.00   Min.   :0.9608   Min.   :0.0194  
##  1st Qu.:0.0000   1st Qu.: 29.00   1st Qu.:2.3166   1st Qu.:0.9243  
##  Median :0.0000   Median : 47.60   Median :2.8174   Median :1.4150  
##  Mean   :0.3934   Mean   : 51.63   Mean   :2.8402   Mean   :1.5234  
##  3rd Qu.:0.4877   3rd Qu.: 70.93   3rd Qu.:3.4863   3rd Qu.:2.1325  
##  Max.   :4.6460   Max.   :130.28   Max.   :4.4630   Max.   :3.1900  
##     Simpson          InvSimpson     log10_read_depth       PD       
```

```
##  Min.   :0.00427   Min.   : 1.004   Min.   :4.638    Min.   : 2.675
##  1st Qu.:0.40975   1st Qu.: 1.695   1st Qu.:4.851    1st Qu.: 6.637
##  Median :0.62700   Median : 2.680   Median :4.947    Median : 8.389
##  Mean   :0.57270   Mean   : 3.671   Mean   :4.926    Mean   : 9.162
##  3rd Qu.:0.78325   3rd Qu.: 4.618   3rd Qu.:5.015    3rd Qu.:11.250
##  Max.   :0.92900   Max.   :13.991   Max.   :5.242    Max.   :19.537
```

```
CHSE<-carapace[carapace$Species == "CHSE",]
KISU<-carapace[carapace$Species == "KISU",]
PSCO<-carapace[carapace$Species == "PSCO",]
STCA<-carapace[carapace$Species == "STCA",]
STOD<-carapace[carapace$Species == "STOD",]
TRSC<-carapace[carapace$Species == "TRSC",]
CHSE
```

```
##           sample.ID
## Sample99   Sample99
## Sample100 Sample100
## Sample101 Sample101
##
## Sample99  ZI.CHSE_M_L2-R9_BP4_02-06-2022_71.AGAA_GTAA.18S.R1.adapter_trimmed.fq.gz,ZI.CHSE_M_L2-R9_BP
## Sample100     ZJ.CHSE_F_L9_BP4_02-06-2022_72.AGAA_GAAAG.18S.R1.adapter_trimmed.fq.gz,ZJ.CHSE_F_L9_BP4
## Sample101   ZK.CHSE_M_L9_BP4_02-06-2022_73.AGAA_TCCTGA.18S.R1.adapter_trimmed.fq.gz,ZK.CHSE_M_L9_BP4_
##           Read_depth  ProjectName  Region Sample_date Year Site Sample_type
## Sample99       80715 Atoka_turtle 18SV8V9    20220602 2022  BP4      turtle
## Sample100      72636 Atoka_turtle 18SV8V9    20220602 2022  BP4      turtle
## Sample101      71903 Atoka_turtle 18SV8V9    20220602 2022  BP4      turtle
##           Substrate Species Species_substrate Species_site Sorter Sex
## Sample99   carapace    CHSE     CHSE_carapace     CHSE_BP4     78   M
## Sample100  carapace    CHSE     CHSE_carapace     CHSE_BP4     79   F
## Sample101  carapace    CHSE     CHSE_carapace     CHSE_BP4     80   M
##           Carapace_length Plastron_length Mass Sample_number Sex_notes
## Sample99              308             221 6600            71        na
## Sample100             231             179 2525            72        na
## Sample101             209             152 2050            73        na
##           PCR1_date extraction_date Observed Chao1 se.chao1      ACE   se.ACE
## Sample99       2022        20220624       26    27 2.313407 26.57709 2.531668
## Sample100      2022        20220624       12    12 0.000000 12.00000 1.500000
## Sample101      2022        20220624       20    20 0.000000 20.00000 1.936492
##           Shannon Simpson InvSimpson log10_read_depth       PD
## Sample99   0.7140 0.38700   1.632594         4.906954 7.035426
## Sample100  0.0194 0.00427   1.004287         4.861152 4.953537
## Sample101  0.1310 0.03450   1.035707         4.856747 5.531198
```

```
KISU
```

```
##          sample.ID
## Sample13  Sample13
## Sample14  Sample14
## Sample15  Sample15
## Sample16  Sample16
## Sample17  Sample17
## Sample45  Sample45
```

```
## Sample46  Sample46
## Sample49  Sample49
## Sample53  Sample53
## Sample74  Sample74
## Sample79  Sample79
## Sample80  Sample80
## Sample81  Sample81
## Sample96  Sample96
##
## Sample13                                    KISU-20210525-01-C-BP4.GA_GTAA.18S.R1.adapter_trimmed
## Sample14                                  KISU-20210525-01-C-S4.TGT_TCGTA.18S.R1.adapter_trimmed.
## Sample15                                KISU-20210525-02-C-S4.CTTT_GAAAG.18S.R1.adapter_trimmed.f
## Sample16                                      KISU-20210525-03-C-S4.CTTT_GC.18S.R1.adapter_trimme
## Sample17                            KISU-20210526-01-C-BP4.TTTCA_TCGTA.18S.R1.adapter_trimmed.fq.
## Sample45    EA.KISU_F_L2-R3-9-10_BP4_01-06-2022_5.GA_TCCTGA.18S.R1.adapter_trimmed.fq.gz,EA.KISU_F_
## Sample46              FA.KISU_M_L2-R-9-11_BP4_01-06-2022_6.GA_GC.18S.R1.adapter_trimmed.fq.gz,FA.KI
## Sample49 IA.KISU_M_L3-10-11-R10-11_BP4_01-06-2022_9.TGT_AA.18S.R1.adapter_trimmed.fq.gz,IA.KISU_M_L3
## Sample53       MA.KISU_F_L7-8-11_BP4_01-06-2022_13.TGT_TCCTGA.18S.R1.adapter_trimmed.fq.gz,MA.KISU_F
## Sample74 YJ.KISU_M_L3-10-11-R2_S4_02-06-2022_45.AAAGAA_TGG.18S.R1.adapter_trimmed.fq.gz,YJ.KISU_M_L3
## Sample79 YO.KISU_M_L3-10-11-R3_S4_02-06-2022_50.AAAGAA_ATC.18S.R1.adapter_trimmed.fq.gz,YO.KISU_M_L3
## Sample80   YP.KISU_F_L3-8-9-R3_S4_02-06-2022_51.AAAGAA_CACG.18S.R1.adapter_trimmed.fq.gz,YP.KISU_F_L
## Sample81           YQ.KISU_M_L3-10-11-R8_S4_02-06-2022_52.AT_AA.18S.R1.adapter_trimmed.fq.gz,YQ.KISU
## Sample96   ZF.KISU_M_L3-10-R1-10_BP4_02-06-2022_68.GTC_CACG.18S.R1.adapter_trimmed.fq.gz,ZF.KISU_M_L
##          Read_depth  ProjectName  Region Sample_date Year Site Sample_type
## Sample13      43496 Atoka_turtle 18SV8V9    20210525 2021  BP4      turtle
## Sample14     104268 Atoka_turtle 18SV8V9    20210525 2021   S4      turtle
## Sample15     109823 Atoka_turtle 18SV8V9    20210525 2021   S4      turtle
## Sample16     122855 Atoka_turtle 18SV8V9    20210525 2021   S4      turtle
## Sample17     115720 Atoka_turtle 18SV8V9    20210526 2021  BP4      turtle
## Sample45      78121 Atoka_turtle 18SV8V9    20220601 2022  BP4      turtle
## Sample46      60420 Atoka_turtle 18SV8V9    20220601 2022  BP4      turtle
## Sample49      92965 Atoka_turtle 18SV8V9    20220601 2022  BP4      turtle
## Sample53      74371 Atoka_turtle 18SV8V9    20220601 2022  BP4      turtle
## Sample74      85823 Atoka_turtle 18SV8V9    20220602 2022   S4      turtle
## Sample79      78216 Atoka_turtle 18SV8V9    20220602 2022   S4      turtle
## Sample80      64787 Atoka_turtle 18SV8V9    20220602 2022   S4      turtle
## Sample81      83406 Atoka_turtle 18SV8V9    20220602 2022   S4      turtle
## Sample96      51481 Atoka_turtle 18SV8V9    20220602 2022  BP4      turtle
##          Substrate Species Species_substrate Species_site Sorter Sex
## Sample13  carapace    KISU     KISU_carapace     KISU_BP4     na   M
## Sample14  carapace    KISU     KISU_carapace      KISU_S4      7   F
## Sample15  carapace    KISU     KISU_carapace      KISU_S4     11   F
## Sample16  carapace    KISU     KISU_carapace      KISU_S4     13   F
## Sample17  carapace    KISU     KISU_carapace     KISU_BP4     19   F
## Sample45  carapace    KISU     KISU_carapace     KISU_BP4     24   F
## Sample46  carapace    KISU     KISU_carapace     KISU_BP4     25   M
## Sample49  carapace    KISU     KISU_carapace     KISU_BP4     31   M
## Sample53  carapace    KISU     KISU_carapace     KISU_BP4     32   F
## Sample74  carapace    KISU     KISU_carapace      KISU_S4     52   M
## Sample79  carapace    KISU     KISU_carapace      KISU_S4     57   M
## Sample80  carapace    KISU     KISU_carapace      KISU_S4     59   F
## Sample81  carapace    KISU     KISU_carapace      KISU_S4     58   M
## Sample96  carapace    KISU     KISU_carapace     KISU_BP4     76   M
##          Carapace_length Plastron_length Mass Sample_number Sex_notes
```

```
## Sample13           68.5            59.7   60             1         na
## Sample14           90.0            84.9  175            15         na
## Sample15          102.5            97.3  245            18         na
## Sample16           87.7            86.8  145            20         na
## Sample17           86.8            80.6  115            35         na
## Sample45           69.0            60.5   53             5         na
## Sample46           75.0            66.0   76             6         na
## Sample49           77.0            66.0   80             9         na
## Sample53           90.0            72.0  120            13         na
## Sample74           93.0            72.0  130            45         na
## Sample79           73.0            65.0   69            50         na
## Sample80           95.0            91.0  190            51         na
## Sample81           94.0            76.0  130            52         na
## Sample96           65.0            56.0   49            68         na
##            PCR1_date extraction_date Observed    Chao1  se.chao1      ACE
## Sample13 15_17_June            2021       30 30.00000 0.0000000 30.00000
## Sample14 17_22_June            2021       57 57.00000 0.0000000 57.00000
## Sample15 23_25_June            2021       71 71.33333 0.9248003 71.86764
## Sample16 23_25_June            2021       87 87.00000 0.1242795 87.31590
## Sample17 25_28_June            2021       90 90.00000 0.0000000 90.00000
## Sample45       2022        20220617       69 69.00000 0.4963636 69.24119
## Sample46       2022        20220617       51 51.00000 0.0000000 51.00000
## Sample49       2022        20220617       70 70.00000 0.0000000 70.00000
## Sample53       2022        20220617       40 40.00000 0.0000000 40.00000
## Sample74       2022        20220623       29 29.00000 0.0000000 29.00000
## Sample79       2022        20220623       32 32.00000 0.0000000 32.00000
## Sample80       2022        20220623       16 16.00000 0.0000000 16.00000
## Sample81       2022        20220623       44 44.00000 0.4942855 44.20106
## Sample96       2022        20220624        9  9.00000 0.0000000  9.00000
##            se.ACE Shannon Simpson InvSimpson log10_read_depth        PD
## Sample13 2.316607   2.600  0.8710   7.780185         4.638449  6.672275
## Sample14 2.752989   1.590  0.6680   3.011729         5.018151  7.348046
## Sample15 3.106883   1.060  0.3460   1.528481         5.040693 11.157167
## Sample16 4.100609   1.680  0.6290   2.693695         5.089393 13.281604
## Sample17 4.012480   2.290  0.7990   4.971332         5.063408 12.572860
## Sample45 3.477342   1.660  0.6980   3.315109         4.892768 12.010712
## Sample46 3.366502   1.070  0.4910   1.962954         4.781181  9.429812
## Sample49 2.661900   2.690  0.8600   7.132622         4.968319  8.056302
## Sample53 2.824004   2.230  0.8300   5.897894         4.871404  6.346057
## Sample74 2.406887   1.300  0.6860   3.187364         4.933604  6.530145
## Sample79 2.622022   1.330  0.6020   2.513393         4.893296  8.420291
## Sample80 1.732051   0.382  0.1380   1.160146         4.811488  4.359322
## Sample81 2.845176   0.438  0.1260   1.143697         4.921197  9.620990
## Sample96 1.414214   0.186  0.0647   1.069125         4.711647  2.674692
```

```
PSCO
```

```
##            sample.ID
## Sample18    Sample18
## Sample19    Sample19
## Sample41    Sample41
## Sample48    Sample48
## Sample90    Sample90
## Sample91    Sample91
```

```
## Sample92   Sample92
## Sample93   Sample93
## Sample94   Sample94
## Sample95   Sample95
## Sample102 Sample102
## Sample103 Sample103
##
## Sample18                                PSCO-20210525-01-C-BP4.TGT_GTAA.18S.R1.adapter_trimmed.fq.g
## Sample19                            PSCO-20210525-02-C-BP4.TGT_TCCTGA.18S.R1.adapter_trimmed.fq.gz
## Sample41            AA.PSCO_J_L2-10-R10_BP4_01-06-2022_1.GA_AA.18S.R1.adapter_trimmed.fq.gz,AA.PSCO_
## Sample48          HA.PSCO_J_L2-10-R8_BP4_01-06-2022_8.GA_CACG.18S.R1.adapter_trimmed.fq.gz,HA.PSCO_J
## Sample90      YZ.STOD_M_L2-9-R1-3_BP4_02-06-2022_62.GTC_TGG.18S.R1.adapter_trimmed.fq.gz,YZ.STOD_M_L
## Sample91    ZA.PSCO_M_L2-10-R11_BP4_02-06-2022_63.GTC_GTAA.18S.R1.adapter_trimmed.fq.gz,ZA.PSCO_M_L2
## Sample92  ZB.PSCO_J_L2-10-R12_BP4_02-06-2022_64.GTC_GAAAG.18S.R1.adapter_trimmed.fq.gz,ZB.PSCO_J_L2-
## Sample93  ZC.PSCO_J_L2-11-R8_BP4_02-06-2022_65.GTC_TCCTGA.18S.R1.adapter_trimmed.fq.gz,ZC.PSCO_J_L2-
## Sample94          ZD.PSCO_M_L2-11-R9_BP4_02-06-2022_66.GTC_GC.18S.R1.adapter_trimmed.fq.gz,ZD.PSCO_M
## Sample95      ZE.PSCO_J_L2-11-R10_BP4_02-06-2022_67.GTC_ATC.18S.R1.adapter_trimmed.fq.gz,ZE.PSCO_J_L
## Sample102     ZL.PSCO_J_L2-10-R10_BP4_02-06-2022_74.AGAA_GC.18S.R1.adapter_trimmed.fq.gz,ZL.PSCO_J_L
## Sample103       ZM.PSCO_F_L2-R1-8_BP4_02-06-2022_75.AGAA_ATC.18S.R1.adapter_trimmed.fq.gz,ZM.PSCO_F_
##           Read_depth  ProjectName  Region Sample_date Year Site Sample_type
## Sample18      110828 Atoka_turtle 18SV8V9    20210525 2021  BP4      turtle
## Sample19      117605 Atoka_turtle 18SV8V9    20210525 2021  BP4      turtle
## Sample41       89534 Atoka_turtle 18SV8V9    20220601 2022  BP4      turtle
## Sample48       60361 Atoka_turtle 18SV8V9    20220601 2022  BP4      turtle
## Sample90       79884 Atoka_turtle 18SV8V9    20220602 2022  BP4      turtle
## Sample91       93640 Atoka_turtle 18SV8V9    20220602 2022  BP4      turtle
## Sample92       58625 Atoka_turtle 18SV8V9    20220602 2022  BP4      turtle
## Sample93       51468 Atoka_turtle 18SV8V9    20220602 2022  BP4      turtle
## Sample94       59736 Atoka_turtle 18SV8V9    20220602 2022  BP4      turtle
## Sample95       58180 Atoka_turtle 18SV8V9    20220602 2022  BP4      turtle
## Sample102      53266 Atoka_turtle 18SV8V9    20220602 2022  BP4      turtle
## Sample103      48819 Atoka_turtle 18SV8V9    20220602 2022  BP4      turtle
##           Substrate Species Species_substrate Species_site Sorter Sex
## Sample18   carapace    PSCO     PSCO_carapace     PSCO_BP4      4   M
## Sample19   carapace    PSCO     PSCO_carapace     PSCO_BP4      5   J
## Sample41   carapace    PSCO     PSCO_carapace     PSCO_BP4     20   J
## Sample48   carapace    PSCO     PSCO_carapace     PSCO_BP4     27   J
## Sample90   carapace    PSCO     PSCO_carapace     PSCO_BP4     69   M
## Sample91   carapace    PSCO     PSCO_carapace     PSCO_BP4     70   M
## Sample92   carapace    PSCO     PSCO_carapace     PSCO_BP4     71   J
## Sample93   carapace    PSCO     PSCO_carapace     PSCO_BP4     72   J
## Sample94   carapace    PSCO     PSCO_carapace     PSCO_BP4     73   M
## Sample95   carapace    PSCO     PSCO_carapace     PSCO_BP4     74   J
## Sample102  carapace    PSCO     PSCO_carapace     PSCO_BP4     81   J
## Sample103  carapace    PSCO     PSCO_carapace     PSCO_BP4     82   F
##           Carapace_length Plastron_length Mass Sample_number Sex_notes
## Sample18            202.3           170.7  900             9        na
## Sample19            133.4           122.0  335            11        na
## Sample41            131.0           120.5  350             1        na
## Sample48             86.0            79.5  110             8        na
## Sample90             84.0            60.0   92            62        na
## Sample91            188.0           166.0  740            63        na
## Sample92            143.0           132.0  425            64        na
## Sample93            137.0           126.0  360            65        na
```

```
## Sample94           212.0           186.0 1050            66        na
## Sample95           100.0            93.0  170            67        na
## Sample102          131.0           120.0  320            74        na
## Sample103          260.0           239.0 2400            75        na
##            PCR1_date extraction_date Observed Chao1   se.chao1       ACE
## Sample18  17_22_June            2021      101 101.0 0.09950372 101.23279
## Sample19  17_22_June            2021      104 104.0 0.16586345 104.29909
## Sample41        2022        20220617      101 101.0 0.16583953 101.23092
## Sample48        2022        20220617       49  52.0 4.62175042  50.63324
## Sample90        2022        20220624       32  32.0 0.00000000  32.00000
## Sample91        2022        20220624       58  58.0 0.16522364  58.40422
## Sample92        2022        20220624       66  66.0 0.09923953  66.27089
## Sample93        2022        20220624       49  49.0 0.16495722  49.25740
## Sample94        2022        20220624       63  63.5 1.29562407  63.40589
## Sample95        2022        20220624       38  38.0 0.00000000  38.00000
## Sample102       2022        20220624       32  32.5 1.29235920  32.61896
## Sample103       2022        20220624       39  39.0 0.49354812  39.20972
##             se.ACE Shannon Simpson InvSimpson log10_read_depth        PD
## Sample18  4.316386   1.670   0.625   2.665754         5.044649 15.333446
## Sample19  3.752478   2.080   0.702   3.351906         5.070426 14.256343
## Sample41  3.812925   1.930   0.668   3.011081         4.951988 14.810360
## Sample48  2.516519   2.200   0.828   5.819574         4.780756  8.950246
## Sample90  2.738613   0.576   0.190   1.234141         4.902460  7.402705
## Sample91  3.243734   2.140   0.778   4.499997         4.971461  9.267598
## Sample92  3.857963   0.852   0.324   1.478803         4.768083  9.002706
## Sample93  3.136016   0.613   0.223   1.286361         4.711537  8.358703
## Sample94  3.679789   0.482   0.130   1.149979         4.776236 10.223890
## Sample95  2.513123   1.440   0.648   2.838327         4.764774  7.008309
## Sample102 2.810883   1.180   0.593   2.455443         4.726450  7.628880
## Sample103 3.008127   1.010   0.428   1.749636         4.688589  8.249660
```

```
STCA
```

```
##          sample.ID
## Sample55  Sample55
## Sample62  Sample62
## Sample63  Sample63
## Sample64  Sample64
## Sample69  Sample69
## Sample70  Sample70
## Sample71  Sample71
## Sample72  Sample72
##
## Sample55              OA.STCA_M_L3-R8-11_S1_01-06-2022_15.TGT_ATC.18S.R1.adapter_trimmed.fq.gz,OA.S
## Sample62              VA.STCA_M_L3-R9-10_S1_01-06-2022_30.CTTT_GC.18S.R1.adapter_trimmed.fq.gz,VA.S
## Sample63            WA.STCA_M_L3-R9-11_S1_01-06-2022_31.CTTT_ATC.18S.R1.adapter_trimmed.fq.gz,WA.ST
## Sample64            XA.STCA_M_L1-3-R3_S1_01-06-2022_32.CTTT_CACG.18S.R1.adapter_trimmed.fq.gz,XA.ST
## Sample69 YE.STCA_F_L3-R10-11_S1_02-06-2022_40.TTTCAA_TCCTGA.18S.R1.adapter_trimmed.fq.gz,YE.STCA_F_L
## Sample70            YF.STCA_M_L2-3-11_S1_02-06-2022_41.TTTCAA_GC.18S.R1.adapter_trimmed.fq.gz,YF.ST
## Sample71          YG.STCA_M_L8-9-10_S1_02-06-2022_42.TTTCAA_ATC.18S.R1.adapter_trimmed.fq.gz,YG.STC
## Sample72        YH.STCA_M_L8-9-11_S1_02-06-2022_43.TTTCAA_CACG.18S.R1.adapter_trimmed.fq.gz,YH.STCA
##          Read_depth  ProjectName  Region Sample_date Year Site Sample_type
## Sample55      53907 Atoka_turtle 18SV8V9    20220601 2022   S1      turtle
## Sample62     107293 Atoka_turtle 18SV8V9    20220601 2022   S1      turtle
```

```
## Sample63      95768 Atoka_turtle 18SV8V9    20220601 2022   S1      turtle
## Sample64      88635 Atoka_turtle 18SV8V9    20220601 2022   S1      turtle
## Sample69      87262 Atoka_turtle 18SV8V9    20220602 2022   S1      turtle
## Sample70      60318 Atoka_turtle 18SV8V9    20220602 2022   S1      turtle
## Sample71      49327 Atoka_turtle 18SV8V9    20220602 2022   S1      turtle
## Sample72      46939 Atoka_turtle 18SV8V9    20220602 2022   S1      turtle
##           Substrate Species Species_substrate Species_site Sorter Sex
## Sample55  carapace    STCA     STCA_carapace      STCA_S1     35   M
## Sample62  carapace    STCA     STCA_carapace      STCA_S1     41   M
## Sample63  carapace    STCA     STCA_carapace      STCA_S1     42   M
## Sample64  carapace    STCA     STCA_carapace      STCA_S1     43   M
## Sample69  carapace    STCA     STCA_carapace      STCA_S1     47   F
## Sample70  carapace    STCA     STCA_carapace      STCA_S1     48   M
## Sample71  carapace    STCA     STCA_carapace      STCA_S1     49   M
## Sample72  carapace    STCA     STCA_carapace      STCA_S1     50   M
##           Carapace_length Plastron_length Mass Sample_number Sex_notes PCR1_date
## Sample55              136            90.0  360            15        na      2022
## Sample62              122            80.0  240            30        na      2022
## Sample63              131            87.0  340            31        na      2022
## Sample64              124            84.0  220            32        na      2022
## Sample69              108            80.0  170            40        na      2022
## Sample70              129            85.0  305            41        na      2022
## Sample71               88            60.5   92            42        na      2022
## Sample72               94            64.0  105            43        na      2022
##           extraction_date Observed Chao1 se.chao1      ACE    se.ACE Shannon
## Sample55         20220617       22    22 0.000000 22.00000 1.6096301   1.370
## Sample62         20220623       36    36 0.000000 36.00000 2.5980762   1.780
## Sample63         20220623       52    52 0.000000 52.00000 2.9450088   2.440
## Sample64         20220623       13    13 0.000000 13.00000 0.9607689   0.901
## Sample69         20220623       47    48 2.327373 47.64394 3.0123045   2.090
## Sample70         20220623       31    31 0.000000 31.00000 2.5272706   1.880
## Sample71         20220623       27    27 0.000000 27.00000 2.2771002   1.750
## Sample72         20220623       15    15 0.000000 15.00000 1.7126977   2.070
##           Simpson InvSimpson log10_read_depth       PD
## Sample55    0.518   2.075516         4.731645 5.127487
## Sample62    0.738   3.816895         5.030571 7.189657
## Sample63    0.869   7.622837         4.981220 8.981257
## Sample64    0.415   1.709998         4.947605 4.818412
## Sample69    0.730   3.703352         4.940825 8.199459
## Sample70    0.678   3.106798         4.780447 7.688080
## Sample71    0.746   3.931571         4.693085 7.001914
## Sample72    0.804   5.113438         4.671534 5.356128
```

```
STOD
```

```
##           sample.ID
## Sample20  Sample20
## Sample21  Sample21
## Sample24  Sample24
## Sample25  Sample25
## Sample26  Sample26
## Sample27  Sample27
## Sample28  Sample28
## Sample30  Sample30
```

```
## Sample32  Sample32
## Sample42  Sample42
## Sample44  Sample44
## Sample56  Sample56
## Sample65  Sample65
## Sample66  Sample66
## Sample68  Sample68
## Sample75  Sample75
## Sample76  Sample76
## Sample77  Sample77
## Sample78  Sample78
## Sample86  Sample86
## Sample87  Sample87
## Sample88  Sample88
## Sample89  Sample89
## Sample97  Sample97
## Sample98  Sample98
##
## Sample20                                          STOD-20210525-01-C-BP4.GA_GAAAG.18S.R1.adapter_trimm
## Sample21                                              STOD-20210525-01-C-S4.TGT_ATC.18S.R1.adapter_tri
## Sample24                                              STOD-20210525-02-C-BP4.TGT_GC.18S.R1.adapter_tri
## Sample25                                        STOD-20210525-02-C-S4.TGT_ACTAAT.18S.R1.adapter_trimme
## Sample26                                          STOD-20210525-03-C-S4.CTTT_GTAA.18S.R1.adapter_trimm
## Sample27                                      STOD-20210525-04-C-S4.CTTT_TCCTGA.18S.R1.adapter_trimmed
## Sample28                                          STOD-20210525-05-C-S4.CTTT_CACG.18S.R1.adapter_trimm
## Sample30                                      STOD-20210525-06-C-S4.CTTT_ACTAAT.18S.R1.adapter_trimmed
## Sample32                                      STOD-20210526-01-C-BP4.TTTCA_CACG.18S.R1.adapter_trimmed
## Sample42                BA.STOD_F_L1-8-9-R11_BP4_01-06-2022_2.GA_TGG.18S.R1.adapter_trimmed.fq.gz,BA.S
## Sample44          DA.STOD_F_L2-9-10-R11_BP4_01-06-2022_4.GA_GAAAG.18S.R1.adapter_trimmed.fq.gz,DA.STOD
## Sample56            PA.STOD_F_L2-9-10-R2_S1_01-06-2022_16.TGT_CACG.18S.R1.adapter_trimmed.fq.gz,PA.STO
## Sample65          YA.STOD_F_L2-9-10-R3_S4_01-06-2022_33.TTTCAA_AA.18S.R1.adapter_trimmed.fq.gz,YA.STOD
## Sample66          YB.STOD_M_L1-8-9-R2_S4_01-06-2022_34.TTTCAA_TGG.18S.R1.adapter_trimmed.fq.gz,YB.STOD
## Sample68    YD.STOD_F_L1-8-11-R8_S1_02-06-2022_39.TTTCAA_GAAAG.18S.R1.adapter_trimmed.fq.gz,YD.STOD_F_
## Sample75      YK.STOD_M_L2-9-10-R8_S4_02-06-2022_46.AAAGAA_GTAA.18S.R1.adapter_trimmed.fq.gz,YK.STOD_M
## Sample76    YL.STOD_M_L2-9-11-R2_S4_02-06-2022_47.AAAGAA_GAAAG.18S.R1.adapter_trimmed.fq.gz,YL.STOD_M_
## Sample77 YM.STOD_M_L2-9-11-R8_S4_02-06-2022_48.AAAGAA_TCCTGA.18S.R1.adapter_trimmed.fq.gz,YM.STOD_M_L
## Sample78          YN.STOD_F_L2-9-11-R3_S4_02-06-2022_49.AAAGAA_GC.18S.R1.adapter_trimmed.fq.gz,YN.STOD
## Sample86                YV.STOD_M_L2-9-11-R9_BP4_02-06-2022_58.AT_GC.18S.R1.adapter_trimmed.fq.gz,YV.S
## Sample87              YW.STOD_M_L2-9-R1-10_BP4_02-06-2022_59.AT_ATC.18S.R1.adapter_trimmed.fq.gz,YW.ST
## Sample88            YX.STOD_M_L2-9-R1-11_BP4_02-06-2022_60.AT_CACG.18S.R1.adapter_trimmed.fq.gz,YX.STO
## Sample89                YY.STOD_M_L2-9-R1-2_BP4_02-06-2022_61.GTC_AA.18S.R1.adapter_trimmed.fq.gz,YY.S
## Sample97              ZG.STOD_F_L2-9-R1-8_BP4_02-06-2022_69.AGAA_AA.18S.R1.adapter_trimmed.fq.gz,ZG.ST
## Sample98            ZH.STOD_F_L1-8-9-10_BP4_02-06-2022_70.AGAA_TGG.18S.R1.adapter_trimmed.fq.gz,ZH.STO
##          Read_depth  ProjectName  Region Sample_date Year Site Sample_type
## Sample20      71200 Atoka_turtle 18SV8V9    20210525 2021  BP4      turtle
## Sample21     123909 Atoka_turtle 18SV8V9    20210525 2021   S4      turtle
## Sample24     127865 Atoka_turtle 18SV8V9    20210525 2021  BP4      turtle
## Sample25     106339 Atoka_turtle 18SV8V9    20210525 2021   S4      turtle
## Sample26     119590 Atoka_turtle 18SV8V9    20210525 2021   S4      turtle
## Sample27     124894 Atoka_turtle 18SV8V9    20210525 2021   S4      turtle
## Sample28     103269 Atoka_turtle 18SV8V9    20210525 2021   S4      turtle
## Sample30      99933 Atoka_turtle 18SV8V9    20210525 2021   S4      turtle
## Sample32     125270 Atoka_turtle 18SV8V9    20210526 2021  BP4      turtle
## Sample42      97616 Atoka_turtle 18SV8V9    20220601 2022  BP4      turtle
```

```
## Sample44      91258 Atoka_turtle 18SV8V9    20220601 2022  BP4      turtle
## Sample56      67222 Atoka_turtle 18SV8V9    20220601 2022   S1      turtle
## Sample65     108884 Atoka_turtle 18SV8V9    20220601 2022   S4      turtle
## Sample66     103389 Atoka_turtle 18SV8V9    20220601 2022   S4      turtle
## Sample68     174528 Atoka_turtle 18SV8V9    20220602 2022   S1      turtle
## Sample75     103805 Atoka_turtle 18SV8V9    20220602 2022   S4      turtle
## Sample76     103817 Atoka_turtle 18SV8V9    20220602 2022   S4      turtle
## Sample77      99597 Atoka_turtle 18SV8V9    20220602 2022   S4      turtle
## Sample78      93872 Atoka_turtle 18SV8V9    20220602 2022   S4      turtle
## Sample86      94842 Atoka_turtle 18SV8V9    20220602 2022  BP4      turtle
## Sample87      91106 Atoka_turtle 18SV8V9    20220602 2022  BP4      turtle
## Sample88      69999 Atoka_turtle 18SV8V9    20220602 2022  BP4      turtle
## Sample89     107745 Atoka_turtle 18SV8V9    20220602 2022  BP4      turtle
## Sample97      95535 Atoka_turtle 18SV8V9    20220602 2022  BP4      turtle
## Sample98      64970 Atoka_turtle 18SV8V9    20220602 2022  BP4      turtle
##          Substrate Species Species_substrate Species_site Sorter Sex
## Sample20  carapace    STOD     STOD_carapace     STOD_BP4     na   M
## Sample21  carapace    STOD     STOD_carapace      STOD_S4      8   M
## Sample24  carapace    STOD     STOD_carapace     STOD_BP4      6   J
## Sample25  carapace    STOD     STOD_carapace      STOD_S4      9   M
## Sample26  carapace    STOD     STOD_carapace      STOD_S4     10   F
## Sample27  carapace    STOD     STOD_carapace      STOD_S4     12   F
## Sample28  carapace    STOD     STOD_carapace      STOD_S4     14   F
## Sample30  carapace    STOD     STOD_carapace      STOD_S4     15   F
## Sample32  carapace    STOD     STOD_carapace     STOD_BP4     18   M
## Sample42  carapace    STOD     STOD_carapace     STOD_BP4     21   F
## Sample44  carapace    STOD     STOD_carapace     STOD_BP4     23   F
## Sample56  carapace    STOD     STOD_carapace      STOD_S1     33   F
## Sample65  carapace    STOD     STOD_carapace      STOD_S4     45   F
## Sample66  carapace    STOD     STOD_carapace      STOD_S4     44   M
## Sample68  carapace    STOD     STOD_carapace      STOD_S1     46   F
## Sample75  carapace    STOD     STOD_carapace      STOD_S4     53   M
## Sample76  carapace    STOD     STOD_carapace      STOD_S4     54   M
## Sample77  carapace    STOD     STOD_carapace      STOD_S4     56   M
## Sample78  carapace    STOD     STOD_carapace      STOD_S4     55   F
## Sample86  carapace    STOD     STOD_carapace     STOD_BP4     65   M
## Sample87  carapace    STOD     STOD_carapace     STOD_BP4     66   M
## Sample88  carapace    STOD     STOD_carapace     STOD_BP4     67   M
## Sample89  carapace    STOD     STOD_carapace     STOD_BP4     68   M
## Sample97  carapace    STOD     STOD_carapace     STOD_BP4     75   F
## Sample98  carapace    STOD     STOD_carapace     STOD_BP4     77   F
##          Carapace_length Plastron_length  Mass Sample_number
## Sample20            53.3            35.8  25.0             2
## Sample21            87.8            59.5 110.0            13
## Sample24            49.4            36.6  30.0            12
## Sample25            94.8            62.3 135.0            16
## Sample26            81.1            59.8 105.0            17
## Sample27            89.6            70.6 145.0            19
## Sample28            85.2            63.5 110.0            21
## Sample30            94.6            65.7 140.0            23
## Sample32            75.4            51.8  60.0            34
## Sample42            82.0            65.0 110.0             2
## Sample44            89.0            65.0 130.0             4
## Sample56            93.0            65.0 105.0            16
```

```
## Sample65            87.0            71.0 130.0            33
## Sample66            87.0            63.0 120.0            34
## Sample68            91.5            66.0 125.0            39
## Sample75            68.0            49.0  50.5            46
## Sample76            91.0            65.0 120.0            47
## Sample77            96.0            62.0 140.0            48
## Sample78            86.0            66.0 105.0            49
## Sample86            88.0            60.0 110.0            58
## Sample87           105.0            65.0 160.0            59
## Sample88            68.0            46.0  48.0            60
## Sample89            82.0            57.0  92.0            61
## Sample97            84.0            63.0 110.0            69
## Sample98            87.0            68.0 120.0            70
##                                                    Sex_notes  PCR1_date
## Sample20 waiting for confirmation on juvenile status from Ethan 15_17_June
## Sample21                                                  na 17_22_June
## Sample24                                                 J/F 17_22_June
## Sample25                                                  na 17_22_June
## Sample26                                                  na 23-25_June
## Sample27                                                  na 23-25_June
## Sample28                                                  na 23-25_June
## Sample30                                                  na 23-25_June
## Sample32                                                  na 25-28_June
## Sample42                                                  na       2022
## Sample44                                                  na       2022
## Sample56                                                  na       2022
## Sample65                                                  na       2022
## Sample66                                                  na       2022
## Sample68                                                  na       2022
## Sample75                                                  na       2022
## Sample76                                                  na       2022
## Sample77                                                  na       2022
## Sample78                                                  na       2022
## Sample86                                                  na       2022
## Sample87                                                  na       2022
## Sample88                                                  na       2022
## Sample89                                                  na       2022
## Sample97                                                  na       2022
## Sample98                                                  na       2022
##          extraction_date Observed Chao1  se.chao1      ACE   se.ACE Shannon
## Sample20            2021       87  87.0 0.0000000 87.00000 3.853644   2.130
## Sample21            2021       73  73.0 0.1241409 73.27597 3.733010   2.080
## Sample24            2021       43  43.0 0.4941519 43.24206 2.629808   1.760
## Sample25            2021       37  37.0 0.4931970 37.21770 2.670301   1.770
## Sample26            2021       47  48.0 2.3273733 48.14325 2.544373   1.070
## Sample27            2021       20  20.0 0.0000000 20.00000 2.049390   0.394
## Sample28            2021       51  51.0 0.0000000 51.00000 3.029269   0.735
## Sample30            2021       52  52.0 0.0000000 52.00000 2.945009   1.070
## Sample32            2021       70  70.5 1.2959634 70.51641 3.679744   2.290
## Sample42        20220617       18  18.0 0.0000000 18.00000 1.581139   0.421
## Sample44        20220617       18  18.0 0.0000000 18.00000 1.581139   1.330
## Sample56        20220617       31  31.0 0.0000000 31.00000 2.199707   1.730
## Sample65        20220623       29  29.0 0.0000000 29.00000 2.678136   1.000
## Sample66        20220623       26  26.0 0.0000000 26.00000 2.009592   1.120
```

```
## Sample68        20220623       74  74.0 0.0000000 74.00000 3.060207   2.690
## Sample75        20220623       43  43.0 0.0000000 43.00000 2.272178   2.040
## Sample76        20220623       35  35.0 0.0000000 35.00000 2.484236   1.540
## Sample77        20220623       45  45.0 0.0000000 45.00000 2.966479   1.230
## Sample78        20220623       18  18.0 0.4859127 18.27242 2.084927   0.932
## Sample86        20220624        9   9.0 0.0000000  9.00000 1.414214   0.604
## Sample87        20220624       13  13.0 0.0000000 13.00000 1.754116   0.884
## Sample88        20220624       17  17.0 0.0000000 17.00000 1.878673   0.601
## Sample89        20220624       16  16.0 0.0000000 16.00000 1.936492   0.994
## Sample97        20220624       24  24.0 0.0000000 24.00000 2.440970   1.200
## Sample98        20220624       14  14.0 0.0000000 14.00000 1.535299   0.803
##          Simpson InvSimpson log10_read_depth        PD
## Sample20   0.727   3.656380         4.852480 12.376283
## Sample21   0.707   3.409567         5.093103 11.011768
## Sample24   0.768   4.309154         5.106752  8.283373
## Sample25   0.711   3.458940         5.026693  6.285341
## Sample26   0.465   1.867572         5.077695  9.359082
## Sample27   0.143   1.166739         5.096542  6.525874
## Sample28   0.263   1.356664         5.013970  9.300845
## Sample30   0.439   1.783786         4.999709  9.446650
## Sample32   0.802   5.048638         5.097847 10.723880
## Sample42   0.161   1.192339         4.989521  4.799912
## Sample44   0.601   2.503469         4.960271  4.834226
## Sample56   0.745   3.916869         4.827511  7.254231
## Sample65   0.527   2.112070         5.036964  7.113173
## Sample66   0.499   1.997111         5.014474  6.316807
## Sample68   0.878   8.224267         5.241865 12.779562
## Sample75   0.803   5.087319         5.016218  8.666320
## Sample76   0.731   3.712969         5.016268  8.275582
## Sample77   0.541   2.178466         4.998246  8.065433
## Sample78   0.496   1.984483         4.972536  5.258696
## Sample86   0.276   1.380781         4.977001  2.921782
## Sample87   0.469   1.881728         4.959547  4.255595
## Sample88   0.281   1.389892         4.845092  4.708311
## Sample89   0.545   2.196396         5.032397  5.276630
## Sample97   0.643   2.804298         4.980163  6.059613
## Sample98   0.514   2.057235         4.812713  3.796045
```

```
TRSC
```

```
##          sample.ID
## Sample33  Sample33
## Sample34  Sample34
## Sample36  Sample36
## Sample38  Sample38
## Sample39  Sample39
## Sample40  Sample40
## Sample43  Sample43
## Sample47  Sample47
## Sample50  Sample50
## Sample51  Sample51
## Sample52  Sample52
## Sample54  Sample54
## Sample57  Sample57
```

```
## Sample58  Sample58
## Sample59  Sample59
## Sample60  Sample60
## Sample61  Sample61
## Sample73  Sample73
## Sample82  Sample82
## Sample83  Sample83
## Sample84  Sample84
## Sample85  Sample85
##
## Sample33                                          TRSC-20210525-01-C-BP4.GA_GC.18S.R1.adapter_trimm
## Sample34                                    TRSC-20210525-01-C-S4.TTTCA_TCCTGA.18S.R1.adapter_trimmed.fq.
## Sample36                                          TRSC-20210525-02-C-BP4.GA_ATC.18S.R1.adapter_trimme
## Sample38                                       TRSC-20210525-03-C-BP4.GA_ACTAAT.18S.R1.adapter_trimmed.f
## Sample39                                       TRSC-20210525-04-C-BP4.TGT_GAAAG.18S.R1.adapter_trimmed.f
## Sample40                                       TRSC-20210526-01-C-BP4.TTTCA_ATC.18S.R1.adapter_trimmed.f
## Sample43              CA.TRSC_M_L3-4-7-8_BP4_01-06-2022_3.GA_GTAA.18S.R1.adapter_trimmed.fq.gz,CA.TRS
## Sample47          GA.TRSC_M_L4-7-8-9-10_BP4_01-06-2022_7.GA_ATC.18S.R1.adapter_trimmed.fq.gz,GA.TRSC_
## Sample50        JA.TRSC_F_L1-3-9-R12_BP4_01-06-2022_10.TGT_TGG.18S.R1.adapter_trimmed.fq.gz,JA.TRSC_F
## Sample51      KA.TRSC_F_L1-7-8-R10_BP4_01-06-2022_11.TGT_GTAA.18S.R1.adapter_trimmed.fq.gz,KA.TRSC_F_
## Sample52      LA.TRSC_M_L1-9-R3-9_BP4_01-06-2022_12.TGT_GAAAG.18S.R1.adapter_trimmed.fq.gz,LA.TRSC_M_
## Sample54            NA.TRSC_M_L1-9-R8-10_S1_01-06-2022_14.TGT_GC.18S.R1.adapter_trimmed.fq.gz,NA.TRSC
## Sample57          QA.TRSC_M_L1-9-R8-11_S1_01-06-2022_25.CTTT_AA.18S.R1.adapter_trimmed.fq.gz,QA.TRSC_
## Sample58        RA.TRSC_M_L1-9-R8-12_S1_01-06-2022_26.CTTT_TGG.18S.R1.adapter_trimmed.fq.gz,RA.TRSC_M
## Sample59          SA.TRSC_M_L9-10-R7_S1_01-06-2022_27.CTTT_GTAA.18S.R1.adapter_trimmed.fq.gz,SA.TRSC_
## Sample60    TA.TRSC_M_L1-9-R9-10_S1_01-06-2022_28.CTTT_GAAAG.18S.R1.adapter_trimmed.fq.gz,TA.TRSC_M_L
## Sample61 UA.TRSC_M_L1-9-R9-11_S1_01-06-2022_29.CTTT_TCCTGA.18S.R1.adapter_trimmed.fq.gz,UA.TRSC_M_L1
## Sample73        YI.TRSC_M_L11-R7-10_S4_02-06-2022_44.AAAGAA_AA.18S.R1.adapter_trimmed.fq.gz,YI.TRSC_M
## Sample82                YR.TRSC_M_L11-R2-8_S4_02-06-2022_53.AT_TGG.18S.R1.adapter_trimmed.fq.gz,YR.TR
## Sample83      YS.TRSC_F_L10-11-12-R3_S4_02-06-2022_54.AT_GTAA.18S.R1.adapter_trimmed.fq.gz,YS.TRSC_F_
## Sample84    YT.TRSC_F_L10-11-12-R8_S4_02-06-2022_55.AT_GAAAG.18S.R1.adapter_trimmed.fq.gz,YT.TRSC_F_L
## Sample85          YU.TRSC_M_L11-R1-7_S4_02-06-2022_56.AT_TCCTGA.18S.R1.adapter_trimmed.fq.gz,YU.TRSC_
##          Read_depth  ProjectName  Region Sample_date Year Site Sample_type
## Sample33      80442 Atoka_turtle 18SV8V9    20210525 2021  BP4      turtle
## Sample34     124804 Atoka_turtle 18SV8V9    20210525 2021   S4      turtle
## Sample36      79246 Atoka_turtle 18SV8V9    20210525 2021  BP4      turtle
## Sample38      56636 Atoka_turtle 18SV8V9    20210525 2021  BP4      turtle
## Sample39     104397 Atoka_turtle 18SV8V9    20210525 2021  BP4      turtle
## Sample40     126749 Atoka_turtle 18SV8V9    20210526 2021  BP4      turtle
## Sample43      83381 Atoka_turtle 18SV8V9    20220601 2022  BP4      turtle
## Sample47      58730 Atoka_turtle 18SV8V9    20220601 2022  BP4      turtle
## Sample50      91385 Atoka_turtle 18SV8V9    20220601 2022  BP4      turtle
## Sample51      88285 Atoka_turtle 18SV8V9    20220601 2022  BP4      turtle
## Sample52      87622 Atoka_turtle 18SV8V9    20220601 2022  BP4      turtle
## Sample54      61401 Atoka_turtle 18SV8V9    20220601 2022   S1      turtle
## Sample57      94494 Atoka_turtle 18SV8V9    20220601 2022   S1      turtle
## Sample58      80585 Atoka_turtle 18SV8V9    20220601 2022   S1      turtle
## Sample59      85768 Atoka_turtle 18SV8V9    20220601 2022   S1      turtle
## Sample60      75694 Atoka_turtle 18SV8V9    20220601 2022   S1      turtle
## Sample61      95127 Atoka_turtle 18SV8V9    20220601 2022   S1      turtle
## Sample73      87884 Atoka_turtle 18SV8V9    20220602 2022   S4      turtle
## Sample82      85829 Atoka_turtle 18SV8V9    20220602 2022   S4      turtle
## Sample83      91165 Atoka_turtle 18SV8V9    20220602 2022   S4      turtle
## Sample84      98615 Atoka_turtle 18SV8V9    20220602 2022   S4      turtle
```

```
## Sample85      89516 Atoka_turtle 18SV8V9    20220602 2022   S4      turtle
##          Substrate Species Species_substrate Species_site Sorter Sex
## Sample33  carapace    TRSC     TRSC_carapace     TRSC_BP4     na   F
## Sample34  carapace    TRSC     TRSC_carapace      TRSC_S4     16   M
## Sample36  carapace    TRSC     TRSC_carapace     TRSC_BP4      1   F
## Sample38  carapace    TRSC     TRSC_carapace     TRSC_BP4      2   M
## Sample39  carapace    TRSC     TRSC_carapace     TRSC_BP4      3   F
## Sample40  carapace    TRSC     TRSC_carapace     TRSC_BP4     17   J
## Sample43  carapace    TRSC     TRSC_carapace     TRSC_BP4     22   M
## Sample47  carapace    TRSC     TRSC_carapace     TRSC_BP4     26   M
## Sample50  carapace    TRSC     TRSC_carapace     TRSC_BP4     28   F
## Sample51  carapace    TRSC     TRSC_carapace     TRSC_BP4     29   F
## Sample52  carapace    TRSC     TRSC_carapace     TRSC_BP4     30   M
## Sample54  carapace    TRSC     TRSC_carapace      TRSC_S1     34   M
## Sample57  carapace    TRSC     TRSC_carapace      TRSC_S1     36   M
## Sample58  carapace    TRSC     TRSC_carapace      TRSC_S1     37   M
## Sample59  carapace    TRSC     TRSC_carapace      TRSC_S1     38   M
## Sample60  carapace    TRSC     TRSC_carapace      TRSC_S1     39   M
## Sample61  carapace    TRSC     TRSC_carapace      TRSC_S1     40   M
## Sample73  carapace    TRSC     TRSC_carapace      TRSC_S4     51   M
## Sample82  carapace    TRSC     TRSC_carapace      TRSC_S4     60   M
## Sample83  carapace    TRSC     TRSC_carapace      TRSC_S4     61   F
## Sample84  carapace    TRSC     TRSC_carapace      TRSC_S4     62   F
## Sample85  carapace    TRSC     TRSC_carapace      TRSC_S4     63   M
##          Carapace_length Plastron_length Mass Sample_number Sex_notes
## Sample33           117.1           109.6  250             4        na
## Sample34           170.7           156.9  775            25        na
## Sample36           177.8           171.4  850             6        na
## Sample38           203.7           181.5 1225             8        na
## Sample39           153.9           142.8  560            10        na
## Sample40            91.2            82.6  125            33       J/M
## Sample43           171.0           152.0  710             3        na
## Sample47           187.5           170.0 1075             7        na
## Sample50           164.0           149.0  680            10        na
## Sample51           204.0           183.0 1250            11        na
## Sample52           163.0           151.0  715            12        na
## Sample54           199.0           180.0 1200            14        na
## Sample57           160.0           152.0  610            25        na
## Sample58           155.0           142.0  510            26        na
## Sample59           188.0           168.0  915            27        na
## Sample60           179.0           161.0  750            28        na
## Sample61           178.0           159.0  690            29        na
## Sample73           174.0           155.0  610            44        na
## Sample82           176.0           152.0  695            53        na
## Sample83           204.0           196.0 1250            54        na
## Sample84           201.0           186.0 1200            55        na
## Sample85           113.0           101.0  210            56        na
##           PCR1_date extraction_date Observed    Chao1   se.chao1       ACE
## Sample33 15_17_June            2021       40  40.00000 0.00000000  40.00000
## Sample34 25-28_June            2021       95  95.33333 0.92517711  95.60860
## Sample36 15_17_June            2021       30  30.00000 0.00000000  30.00000
## Sample38 15_17_June            2021       47  47.50000 1.29447205  47.54976
## Sample39 17_22_June            2021       87  87.00000 0.49711813  87.16877
## Sample40 25-28_June            2021       81  81.00000 0.24845200  81.18806
```

```
## Sample43       2022        20220617       89  89.00000 0.00000000  89.00000
## Sample47       2022        20220617       74  74.00000 0.00000000  74.00000
## Sample50       2022        20220617       60  60.00000 0.00000000  60.00000
## Sample51       2022        20220617       42  42.00000 0.00000000  42.00000
## Sample52       2022        20220617       53  53.00000 0.00000000  53.00000
## Sample54       2022        20220617       70  70.50000 1.29596345  70.61881
## Sample57       2022        20220617      120 123.00000 4.64600537 120.95765
## Sample58       2022        20220617      123 123.00000 0.00000000 123.00000
## Sample59       2022        20220617      107 107.00000 0.49765807 107.18781
## Sample60       2022        20220617      130 130.00000 0.09961464 130.28081
## Sample61       2022        20220623      104 104.00000 0.49759035 104.18670
## Sample73       2022        20220623       88  88.00000 0.00000000  88.00000
## Sample82       2022        20220623       74  74.00000 0.24830507  74.27013
## Sample83       2022        20220623       70  70.00000 0.00000000  70.00000
## Sample84       2022        20220624       49  49.00000 0.00000000  49.00000
## Sample85       2022        20220624       51  52.00000 2.32875008  51.69756
##            se.ACE Shannon Simpson InvSimpson log10_read_depth        PD
## Sample33 2.529822   3.060   0.918  12.234517         4.905483  7.395668
## Sample34 4.210825   2.840   0.860   7.152372         5.096229 11.633775
## Sample36 2.316607   0.994   0.394   1.650733         4.898977  7.861920
## Sample38 3.299672   2.320   0.830   5.876013         4.753093  9.088148
## Sample39 3.747923   2.690   0.851   6.714189         5.018688 11.835606
## Sample40 3.546845   3.030   0.910  11.132579         5.102945 11.530097
## Sample43 3.622650   2.410   0.820   5.553454         4.921067 13.266494
## Sample47 4.026936   2.550   0.880   8.350419         4.768860 10.970226
## Sample50 2.486631   1.250   0.451   1.820524         4.960875  8.911418
## Sample51 2.415230   1.410   0.602   2.513362         4.945887  6.854829
## Sample52 3.398113   0.426   0.153   1.180374         4.942613 10.169400
## Sample54 3.466486   1.420   0.546   2.204697         4.788175 14.528626
## Sample57 3.744834   3.190   0.929  13.990983         4.975404 18.063455
## Sample58 4.463037   3.100   0.893   9.338995         4.906254 18.286383
## Sample59 3.223898   2.230   0.735   3.779612         4.933325 19.429675
## Sample60 3.913413   3.100   0.906  10.604094         4.879061 19.536637
## Sample61 3.647740   2.990   0.908  10.827324         4.978304 18.437202
## Sample73 4.062019   1.080   0.341   1.516423         4.943910 13.578638
## Sample82 3.243024   1.470   0.661   2.945853         4.933634 12.822346
## Sample83 3.513240   0.666   0.200   1.250609         4.959828 11.706936
## Sample84 3.162278   0.960   0.540   2.172413         4.993943 10.483936
## Sample85 2.888001   0.541   0.174   1.211259         4.951901  8.666369
```

Making stacked barplots for taxonomy

This code generates stacked taxonomy barplots for turtle-species/site combinations levels at the taxonomic rank of family. Only the top 17 most common taxa are indicated for each panel of plots. Warnings are disabled as use of microDecon to adjust data for contamination introduced fractional counts less than one.

```
phylo_carapace<-subset_samples(phylo,Substrate=="carapace")
p1 <- phyloseq::merge_samples(phylo_carapace, group = "Species_site") %>%
  comp_barplot(
    tax_level = "Family", n_taxa = 17, merge_other=FALSE, bar_outline_colour = "grey50",
    #sample_order = c( "TRSC", "STOD", "STCA", "PSCO", "KISU", "CHSE"),
    sample_order = c("TRSC_S4", "TRSC_S1", "TRSC_BP4", "STOD_S4", "STOD_S1", "STOD_BP4", "STCA_S1", "PS
    bar_width = 0.8
```

```
  ) +
  coord_flip() + labs(x = NULL, y = NULL)
p1
```

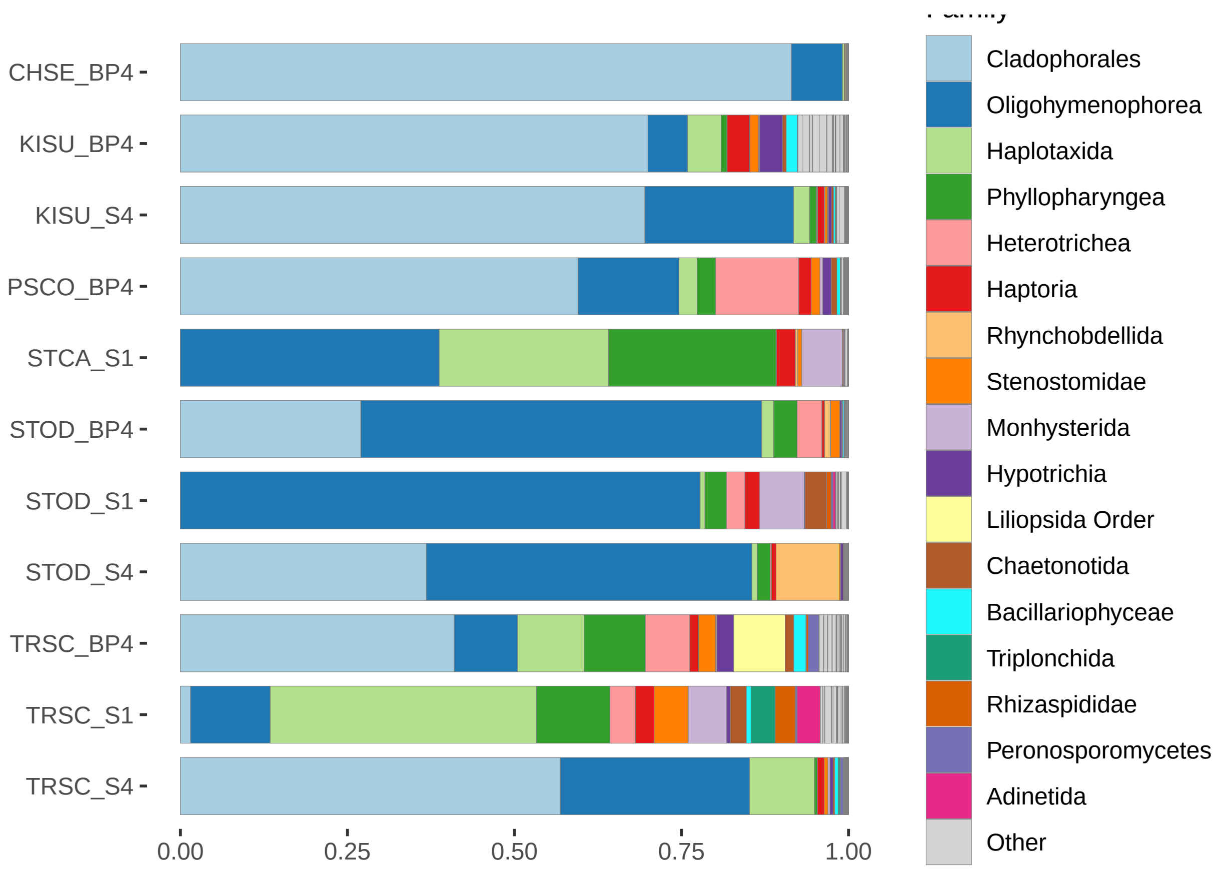

Extracting abundances for several taxa shared with Parks et al. (2020)

This codes includes merging ASVs into shared genera, and merging all samples from a turtle species into a single representative sample for the turtle species. The resulting abundances are for several taxa recovered in carapace microbiomes shared in common to those recovered by Parks et al. (2020) in sampling of Trachemys scripta shell microbial communities.

```
phylo_carapace
```

```
## phyloseq-class experiment-level object
## otu_table()   OTU Table:         [ 513 taxa and 84 samples ]
## sample_data() Sample Data:       [ 84 samples by 32 sample variables ]
## tax_table()   Taxonomy Table:    [ 513 taxa by 7 taxonomic ranks ]
## phy_tree()    Phylogenetic Tree: [ 513 tips and 509 internal nodes ]
```

```
genus = tax_glom(phylo_carapace, taxrank = "Genus")

genus <- merge_samples(genus, "Species")

# Use psmelt to obtain a long-format data.frame
```

```r
genus_merged <- genus %>% tax_glom(taxrank = "Genus") %>% transform_sample_counts(function(x) {x/sum(x)
chse <- subset(genus_merged, Sample == "CHSE")
chse
```

```
##                                  OTU Sample    Abundance sample.ID LibraryName
## 596 b67d6ea01a3e536a54482ca7a0981684   CHSE 9.144884e-01        NA          NA
## 104 209c361a75dc875d21c73373c80aecd6   CHSE 7.582290e-02        NA          NA
## 518 a26e880299a19c2b85beab3bbba55623   CHSE 2.636365e-03        NA          NA
## 373 6dc8b15f0fc7611c071144212bff62f3   CHSE 1.564382e-03        NA          NA
## 155 2e30e2fdb8f5fac8b67a95395cdcb2f5   CHSE 8.939623e-04        NA          NA
## 688 cea8b7fb6c36db1330f4e9764e791077   CHSE 5.610249e-04        NA          NA
## 466 97e8908767c64292554bb350129d80d1   CHSE 4.611164e-04        NA          NA
## 358 695f3bc8518a9fdb8d9fa513b6ec765d   CHSE 4.470334e-04        NA          NA
## 816 ee4de42ed18d06fb70b73e219f0fd91c   CHSE 3.554101e-04        NA          NA
## 547 acde1ac7566d5b6e93a64bb9f73218a4   CHSE 3.140833e-04        NA          NA
## 752 e4279e681a284a86d4884a0f35a7e579   CHSE 2.929974e-04        NA          NA
## 844 f595b71d4ab4cb0209b8788b82fc5c60   CHSE 2.727894e-04        NA          NA
## 544 ac4274a3f58b992ed6c1cbbcf0005f59   CHSE 2.304139e-04        NA          NA
## 763 e4c439f92e51482a4ffdef8c84537431   CHSE 1.860912e-04        NA          NA
## 771 e53717c4df25eed26207b0acdab69e42   CHSE 1.537055e-04        NA          NA
## 330 5b18f4a2e63b865298307685ee1e2a76   CHSE 1.478091e-04        NA          NA
## 309 57639963d6e65c618307604736176612   CHSE 1.460202e-04        NA          NA
## 392 7c86b3982d831b10270365d8bde64d5e   CHSE 1.435556e-04        NA          NA
## 353 66ab6ab16c6d87dec704df0790c04aed   CHSE 1.321142e-04        NA          NA
## 775 e549fd51fd9571c0c77bbabb0ff13a73   CHSE 1.239803e-04        NA          NA
## 848 f5b5e56300c25d417240199c4a12d132   CHSE 1.239803e-04        NA          NA
## 782 e98c4e4a6a56a9a0a7df09bdb223d07b   CHSE 1.074496e-04        NA          NA
## 204 3879cd2e8809cece9402833c8665af87   CHSE 6.916745e-05        NA          NA
## 386 7a47718a7b3879a3f548460e8bd9e8b4   CHSE 5.939069e-05        NA          NA
## 174 3131defa0456216f7aa4d5474fb5ed5b   CHSE 5.170542e-05        NA          NA
## 529 a6e1e80e43fcee8651098c1415823591   CHSE 4.611164e-05        NA          NA
## 444 8ffd2a82beadbdd8011c21f0580a0477   CHSE 4.132675e-05        NA          NA
## 501 a067af8801c15610261c02fdbbcbeaee   CHSE 3.842636e-05        NA          NA
## 48  0cc2931f5c4f3c66baf77ba7421dbd39   CHSE 3.447028e-05        NA          NA
## 860 f742b6f1d4bac4744069ac0400552db0   CHSE 3.074109e-05        NA          NA
## 218 3ccc464b14a04d4513d763517f78d9d6   CHSE 2.305582e-05        NA          NA
## 6   003c5ee1b5a5dfe4ef306013d5486d40   CHSE 0.000000e+00        NA          NA
## 8   00dfcabb2bf805ab24ccc63585e26df0   CHSE 0.000000e+00        NA          NA
## 17  0323e63ea23934e9059f57ce10b57767   CHSE 0.000000e+00        NA          NA
## 20  05e0685e1392f500b5efe42eb62c6926   CHSE 0.000000e+00        NA          NA
## 26  0776b00b47df7f4c79ed760543673acf   CHSE 0.000000e+00        NA          NA
## 36  077f93d2e5833a31e924533cc90f0e4e   CHSE 0.000000e+00        NA          NA
## 37  08d60da72f2e93595bcf7957d3a00be3   CHSE 0.000000e+00        NA          NA
## 54  0d6ad349acac052cc7f5b29b87337927   CHSE 0.000000e+00        NA          NA
## 60  14610001ebd204b349f393854f5a42d4   CHSE 0.000000e+00        NA          NA
## 62  14a103111ece5d8981023ebd315f0b25   CHSE 0.000000e+00        NA          NA
## 70  19565c6992d65726026556348f3d470a   CHSE 0.000000e+00        NA          NA
## 74  1c15885a63f2647fe673d38048b504ca   CHSE 0.000000e+00        NA          NA
## 80  1d7bd6e0a7eea343cf22496e6deea260   CHSE 0.000000e+00        NA          NA
## 90  1fdb643b5117c7343b8759d68b6a5db4   CHSE 0.000000e+00        NA          NA
## 94  1fed30d21b0de26a20f21bd6af322332   CHSE 0.000000e+00        NA          NA
## 102 201f16c1593a734c0f01f800b1e58748   CHSE 0.000000e+00        NA          NA
## 112 233610153215429b8356dfcc5e6d8087   CHSE 0.000000e+00        NA          NA
```

```
## 120 23477c5405ac4e36ce3c2e98a64358e2   CHSE 0.000000e+00         NA           NA
## 123 24180eaf7f850109d1a63ff258a67052   CHSE 0.000000e+00         NA           NA
## 131 244947dcdc8e49b5e30fcc3c736c3e32   CHSE 0.000000e+00         NA           NA
## 133 25ba93f0919412d21ef3344faae98893   CHSE 0.000000e+00         NA           NA
## 144 27fe63dbe2fa00139e0df2e33720c73e   CHSE 0.000000e+00         NA           NA
## 147 2b4d960b73500d33769b45c74990d26d   CHSE 0.000000e+00         NA           NA
## 162 2f1d48f838b36de1d989a22e12c31085   CHSE 0.000000e+00         NA           NA
## 165 30d9cfac4dee44a5db7f3f209fc16684   CHSE 0.000000e+00         NA           NA
## 177 32cd714da38aefbf6c8eec97b598dfd6   CHSE 0.000000e+00         NA           NA
## 185 36004e0b08d5028e689db8ad034bcdcf   CHSE 0.000000e+00         NA           NA
## 192 372198a0c5add35ab81b5e92076853fb   CHSE 0.000000e+00         NA           NA
## 196 380b0df4b57d900524366fbfeda2b855   CHSE 0.000000e+00         NA           NA
## 209 3ab2cfd86fbd3291e902a1177ce0d30d   CHSE 0.000000e+00         NA           NA
## 212 3b9a7de7b5c3828202d5cbbbd88fc73e   CHSE 0.000000e+00         NA           NA
## 228 3d18af72e65e18d8246da2530e09a15d   CHSE 0.000000e+00         NA           NA
## 234 3e2c687294ee598fd93560fa1012cfc2   CHSE 0.000000e+00         NA           NA
## 237 3eb875c3fd763b395c417440009be743   CHSE 0.000000e+00         NA           NA
## 242 47b78d9566a2539d232173accefd3002   CHSE 0.000000e+00         NA           NA
## 249 47c904f2773998574258444025b9638b   CHSE 0.000000e+00         NA           NA
## 254 47ebbdcb2337e8f1c663d9642115e5ce   CHSE 0.000000e+00         NA           NA
## 261 48cf0b33aca8d8dbb795b3c86e978031   CHSE 0.000000e+00         NA           NA
## 265 48e4adbcff483ff866ea39bb88cd9eef   CHSE 0.000000e+00         NA           NA
## 273 4b99550ebd65ec0d7ec914b73626b202   CHSE 0.000000e+00         NA           NA
## 279 4bae15d017e7498a7a95efee32adce81   CHSE 0.000000e+00         NA           NA
## 287 4d7de46af0e80f64fa125f817a111bc2   CHSE 0.000000e+00         NA           NA
## 294 5197dfdc25ba83865f6b9671e145d331   CHSE 0.000000e+00         NA           NA
## 300 55d90bbccdc906eb1ffd05c5dde48cc6   CHSE 0.000000e+00         NA           NA
## 304 564abc0948938adda161516c1c944daa   CHSE 0.000000e+00         NA           NA
## 315 585696e92b06d09ab7a52fff38042a36   CHSE 0.000000e+00         NA           NA
## 321 58c3d160fc28779aa43623922d8c9cd9   CHSE 0.000000e+00         NA           NA
## 336 606ef3ba6711a592609f4f1f324b2d02   CHSE 0.000000e+00         NA           NA
## 340 659d0c32e826b429d9c6319f01bd8e78   CHSE 0.000000e+00         NA           NA
## 343 65f5f578c8d646d283c13506eae6a130   CHSE 0.000000e+00         NA           NA
## 366 6a89bf70acf0ad89d7daf130654c0c3c   CHSE 0.000000e+00         NA           NA
## 372 6c89739fd891cabe74d5b28d5f11e949   CHSE 0.000000e+00         NA           NA
## 383 6f56b5db3008b39573175140284a4ab1   CHSE 0.000000e+00         NA           NA
## 401 80189ee4870bf141414ebb583ccb543b   CHSE 0.000000e+00         NA           NA
## 407 80244512fa02a84aeecfb3680bbf141f   CHSE 0.000000e+00         NA           NA
## 410 80a21a230c73ec96166d9d48a685eb3f   CHSE 0.000000e+00         NA           NA
## 417 82d6d9b7335776eb480b3556469ea2b7   CHSE 0.000000e+00         NA           NA
## 424 8504dfd1fc0ab4891d9301b95161e7d6   CHSE 0.000000e+00         NA           NA
## 429 8eddda1918a4bcf5c60ce2230fd6867d   CHSE 0.000000e+00         NA           NA
## 435 8f129c2b2d3ca00fed4978cccb4dcf81   CHSE 0.000000e+00         NA           NA
## 446 903bdf51482c709f9cc0b084098195eb   CHSE 0.000000e+00         NA           NA
## 456 95335e65bd761d7854f7a948791ca883   CHSE 0.000000e+00         NA           NA
## 459 97297518574a421782aaa957d916d231   CHSE 0.000000e+00         NA           NA
## 471 985641653102f94d3db3ea748c2a062a   CHSE 0.000000e+00         NA           NA
## 477 9872b6306cf41a87059c45bb15cf007c   CHSE 0.000000e+00         NA           NA
## 483 991376a5684a3ae6521ae0aea4de6dc7   CHSE 0.000000e+00         NA           NA
## 488 9bb331ab2b207efb293397a3c9e99ee7   CHSE 0.000000e+00         NA           NA
## 495 9d2d0f126622459bf77ea636719474ab   CHSE 0.000000e+00         NA           NA
## 508 a0f759f600b79ae5aa311a872f9779ec   CHSE 0.000000e+00         NA           NA
## 513 a211ab477ead37b3a94583d53466502d   CHSE 0.000000e+00         NA           NA
## 524 a4b24564fe7b6a3d90c8a0f0946d4c77   CHSE 0.000000e+00         NA           NA
```

```
## 536 ab84230be5240e5fcba940dfdd14565d   CHSE 0.000000e+00        NA          NA
## 553 ad8a103129c19403fc997a17149c941e   CHSE 0.000000e+00        NA          NA
## 560 afc8a4c48ce3660c5ed2b2cea41cf5de   CHSE 0.000000e+00        NA          NA
## 566 aff536faed83252c4c07fc7fa5a05c0a   CHSE 0.000000e+00        NA          NA
## 576 b00e74fbf5f4da43f7c0799df8667c27   CHSE 0.000000e+00        NA          NA
## 578 b12c5d6eabe4d50013e63de57f4e4621   CHSE 0.000000e+00        NA          NA
## 584 b2d556b9c8220cec28d32761abd03a41   CHSE 0.000000e+00        NA          NA
## 591 b427c51feb44612a975b0ce30e5a14ff   CHSE 0.000000e+00        NA          NA
## 602 b6c0c40ac312b9305a99c6d56e3dc92c   CHSE 0.000000e+00        NA          NA
## 611 b6da70d5930e42d1ae13c7a513fa9579   CHSE 0.000000e+00        NA          NA
## 616 b8de421ae421d404c74477cd629d9da9   CHSE 0.000000e+00        NA          NA
## 621 b90aa4694de6f7ef3ba6cf52dc0df264   CHSE 0.000000e+00        NA          NA
## 627 b976340ad78040bd3734f62f3a33ad86   CHSE 0.000000e+00        NA          NA
## 636 ba84ac31c6b4d4fe2a748b7be19ccf22   CHSE 0.000000e+00        NA          NA
## 639 bac106077343175e9231902dab6fd6a1   CHSE 0.000000e+00        NA          NA
## 645 be9e06529505146e17aaf503a483b44b   CHSE 0.000000e+00        NA          NA
## 654 c10521b7aca26286e3a1af8f5aa6b21b   CHSE 0.000000e+00        NA          NA
## 656 c2041d468fb08ed7f3a5902456bd27c9   CHSE 0.000000e+00        NA          NA
## 662 c41a85f9cf4af92d3b20fa6394bf39ca   CHSE 0.000000e+00        NA          NA
## 672 c42c72079e4b3cdf650e00134005d876   CHSE 0.000000e+00        NA          NA
## 676 ca631eaa8021c9bd35043f3b5d3a8f78   CHSE 0.000000e+00        NA          NA
## 684 cbbcaf8df153b84e21e8141a5a738c8d   CHSE 0.000000e+00        NA          NA
## 696 cef116d69a2a12ba692fbcbe8798cbe0   CHSE 0.000000e+00        NA          NA
## 702 d1ba86ca1673ceb52bda2dd35470aa6d   CHSE 0.000000e+00        NA          NA
## 703 d1c03b58a44789bdf3b85abc1723a01d   CHSE 0.000000e+00        NA          NA
## 713 d1fcd1bcab533267713070d71ac90136   CHSE 0.000000e+00        NA          NA
## 719 dbd7f00941ee192db4642e331b67ca9b   CHSE 0.000000e+00        NA          NA
## 726 dc14c243314b825eb156b610f04cf3b3   CHSE 0.000000e+00        NA          NA
## 732 dc81b34715e4fb35b1f60449222591cf   CHSE 0.000000e+00        NA          NA
## 734 dcf70bfac8bd4bff480f29449a88b7c6   CHSE 0.000000e+00        NA          NA
## 741 dd1f0eb0be205a4097523eed0ebd3345   CHSE 0.000000e+00        NA          NA
## 747 dd665b6076ccf9b5abb58794f6cbb13e   CHSE 0.000000e+00        NA          NA
## 760 e4b645e03851eefb01df284dca6e7fd7   CHSE 0.000000e+00        NA          NA
## 790 e9c233e7f840d5c5d5297c188c7dba2f   CHSE 0.000000e+00        NA          NA
## 798 ed1a43eeb73b5c6c20d74b82f85600b3   CHSE 0.000000e+00        NA          NA
## 804 ed36144a332440d3d0bfefbebde4788b   CHSE 0.000000e+00        NA          NA
## 805 ed387d17e8d99ea6d7a9ca5e83b44122   CHSE 0.000000e+00        NA          NA
## 817 f0fe389378462680c28ef31ba99b62e0   CHSE 0.000000e+00        NA          NA
## 825 f146be138f993deaa4abc34d3bbcb847   CHSE 0.000000e+00        NA          NA
## 830 f228fcbd4b99bfbce61137ccbad936b9   CHSE 0.000000e+00        NA          NA
## 838 f46ed5eda4674d3f44d318a4b6f3e5c1   CHSE 0.000000e+00        NA          NA
## 857 f6a9d48b89e830fc6867ed61c3f2b083   CHSE 0.000000e+00        NA          NA
## 869 f8a8223b1a4f0fb02e5de874e86bc01e   CHSE 0.000000e+00        NA          NA
## 875 fae159b942c1639acaa3b4e26e91a154   CHSE 0.000000e+00        NA          NA
## 882 fc9a9c6d302bd1c80550b1511436dc77   CHSE 0.000000e+00        NA          NA
## 888 fce04bfd74657cd39ed56756a127de98   CHSE 0.000000e+00        NA          NA
## 890 ff13969b63a73ce3ae0c69f161d66864   CHSE 0.000000e+00        NA          NA
##     Read_depth ProjectName Region Sample_date Year Site Sample_type Substrate
## 596   75084.67          NA     NA    20220602 2022   NA          NA        NA
## 104   75084.67          NA     NA    20220602 2022   NA          NA        NA
## 518   75084.67          NA     NA    20220602 2022   NA          NA        NA
## 373   75084.67          NA     NA    20220602 2022   NA          NA        NA
## 155   75084.67          NA     NA    20220602 2022   NA          NA        NA
## 688   75084.67          NA     NA    20220602 2022   NA          NA        NA
```

```
## 466   75084.67          NA     NA    20220602 2022   NA          NA        NA
## 358   75084.67          NA     NA    20220602 2022   NA          NA        NA
## 816   75084.67          NA     NA    20220602 2022   NA          NA        NA
## 547   75084.67          NA     NA    20220602 2022   NA          NA        NA
## 752   75084.67          NA     NA    20220602 2022   NA          NA        NA
## 844   75084.67          NA     NA    20220602 2022   NA          NA        NA
## 544   75084.67          NA     NA    20220602 2022   NA          NA        NA
## 763   75084.67          NA     NA    20220602 2022   NA          NA        NA
## 771   75084.67          NA     NA    20220602 2022   NA          NA        NA
## 330   75084.67          NA     NA    20220602 2022   NA          NA        NA
## 309   75084.67          NA     NA    20220602 2022   NA          NA        NA
## 392   75084.67          NA     NA    20220602 2022   NA          NA        NA
## 353   75084.67          NA     NA    20220602 2022   NA          NA        NA
## 775   75084.67          NA     NA    20220602 2022   NA          NA        NA
## 848   75084.67          NA     NA    20220602 2022   NA          NA        NA
## 782   75084.67          NA     NA    20220602 2022   NA          NA        NA
## 204   75084.67          NA     NA    20220602 2022   NA          NA        NA
## 386   75084.67          NA     NA    20220602 2022   NA          NA        NA
## 174   75084.67          NA     NA    20220602 2022   NA          NA        NA
## 529   75084.67          NA     NA    20220602 2022   NA          NA        NA
## 444   75084.67          NA     NA    20220602 2022   NA          NA        NA
## 501   75084.67          NA     NA    20220602 2022   NA          NA        NA
## 48    75084.67          NA     NA    20220602 2022   NA          NA        NA
## 860   75084.67          NA     NA    20220602 2022   NA          NA        NA
## 218   75084.67          NA     NA    20220602 2022   NA          NA        NA
## 6     75084.67          NA     NA    20220602 2022   NA          NA        NA
## 8     75084.67          NA     NA    20220602 2022   NA          NA        NA
## 17    75084.67          NA     NA    20220602 2022   NA          NA        NA
## 20    75084.67          NA     NA    20220602 2022   NA          NA        NA
## 26    75084.67          NA     NA    20220602 2022   NA          NA        NA
## 36    75084.67          NA     NA    20220602 2022   NA          NA        NA
## 37    75084.67          NA     NA    20220602 2022   NA          NA        NA
## 54    75084.67          NA     NA    20220602 2022   NA          NA        NA
## 60    75084.67          NA     NA    20220602 2022   NA          NA        NA
## 62    75084.67          NA     NA    20220602 2022   NA          NA        NA
## 70    75084.67          NA     NA    20220602 2022   NA          NA        NA
## 74    75084.67          NA     NA    20220602 2022   NA          NA        NA
## 80    75084.67          NA     NA    20220602 2022   NA          NA        NA
## 90    75084.67          NA     NA    20220602 2022   NA          NA        NA
## 94    75084.67          NA     NA    20220602 2022   NA          NA        NA
## 102   75084.67          NA     NA    20220602 2022   NA          NA        NA
## 112   75084.67          NA     NA    20220602 2022   NA          NA        NA
## 120   75084.67          NA     NA    20220602 2022   NA          NA        NA
## 123   75084.67          NA     NA    20220602 2022   NA          NA        NA
## 131   75084.67          NA     NA    20220602 2022   NA          NA        NA
## 133   75084.67          NA     NA    20220602 2022   NA          NA        NA
## 144   75084.67          NA     NA    20220602 2022   NA          NA        NA
## 147   75084.67          NA     NA    20220602 2022   NA          NA        NA
## 162   75084.67          NA     NA    20220602 2022   NA          NA        NA
## 165   75084.67          NA     NA    20220602 2022   NA          NA        NA
## 177   75084.67          NA     NA    20220602 2022   NA          NA        NA
## 185   75084.67          NA     NA    20220602 2022   NA          NA        NA
## 192   75084.67          NA     NA    20220602 2022   NA          NA        NA
## 196   75084.67          NA     NA    20220602 2022   NA          NA        NA
```

```
## 209   75084.67            NA      NA     20220602 2022   NA           NA         NA
## 212   75084.67            NA      NA     20220602 2022   NA           NA         NA
## 228   75084.67            NA      NA     20220602 2022   NA           NA         NA
## 234   75084.67            NA      NA     20220602 2022   NA           NA         NA
## 237   75084.67            NA      NA     20220602 2022   NA           NA         NA
## 242   75084.67            NA      NA     20220602 2022   NA           NA         NA
## 249   75084.67            NA      NA     20220602 2022   NA           NA         NA
## 254   75084.67            NA      NA     20220602 2022   NA           NA         NA
## 261   75084.67            NA      NA     20220602 2022   NA           NA         NA
## 265   75084.67            NA      NA     20220602 2022   NA           NA         NA
## 273   75084.67            NA      NA     20220602 2022   NA           NA         NA
## 279   75084.67            NA      NA     20220602 2022   NA           NA         NA
## 287   75084.67            NA      NA     20220602 2022   NA           NA         NA
## 294   75084.67            NA      NA     20220602 2022   NA           NA         NA
## 300   75084.67            NA      NA     20220602 2022   NA           NA         NA
## 304   75084.67            NA      NA     20220602 2022   NA           NA         NA
## 315   75084.67            NA      NA     20220602 2022   NA           NA         NA
## 321   75084.67            NA      NA     20220602 2022   NA           NA         NA
## 336   75084.67            NA      NA     20220602 2022   NA           NA         NA
## 340   75084.67            NA      NA     20220602 2022   NA           NA         NA
## 343   75084.67            NA      NA     20220602 2022   NA           NA         NA
## 366   75084.67            NA      NA     20220602 2022   NA           NA         NA
## 372   75084.67            NA      NA     20220602 2022   NA           NA         NA
## 383   75084.67            NA      NA     20220602 2022   NA           NA         NA
## 401   75084.67            NA      NA     20220602 2022   NA           NA         NA
## 407   75084.67            NA      NA     20220602 2022   NA           NA         NA
## 410   75084.67            NA      NA     20220602 2022   NA           NA         NA
## 417   75084.67            NA      NA     20220602 2022   NA           NA         NA
## 424   75084.67            NA      NA     20220602 2022   NA           NA         NA
## 429   75084.67            NA      NA     20220602 2022   NA           NA         NA
## 435   75084.67            NA      NA     20220602 2022   NA           NA         NA
## 446   75084.67            NA      NA     20220602 2022   NA           NA         NA
## 456   75084.67            NA      NA     20220602 2022   NA           NA         NA
## 459   75084.67            NA      NA     20220602 2022   NA           NA         NA
## 471   75084.67            NA      NA     20220602 2022   NA           NA         NA
## 477   75084.67            NA      NA     20220602 2022   NA           NA         NA
## 483   75084.67            NA      NA     20220602 2022   NA           NA         NA
## 488   75084.67            NA      NA     20220602 2022   NA           NA         NA
## 495   75084.67            NA      NA     20220602 2022   NA           NA         NA
## 508   75084.67            NA      NA     20220602 2022   NA           NA         NA
## 513   75084.67            NA      NA     20220602 2022   NA           NA         NA
## 524   75084.67            NA      NA     20220602 2022   NA           NA         NA
## 536   75084.67            NA      NA     20220602 2022   NA           NA         NA
## 553   75084.67            NA      NA     20220602 2022   NA           NA         NA
## 560   75084.67            NA      NA     20220602 2022   NA           NA         NA
## 566   75084.67            NA      NA     20220602 2022   NA           NA         NA
## 576   75084.67            NA      NA     20220602 2022   NA           NA         NA
## 578   75084.67            NA      NA     20220602 2022   NA           NA         NA
## 584   75084.67            NA      NA     20220602 2022   NA           NA         NA
## 591   75084.67            NA      NA     20220602 2022   NA           NA         NA
## 602   75084.67            NA      NA     20220602 2022   NA           NA         NA
## 611   75084.67            NA      NA     20220602 2022   NA           NA         NA
## 616   75084.67            NA      NA     20220602 2022   NA           NA         NA
## 621   75084.67            NA      NA     20220602 2022   NA           NA         NA
```

```
## 627   75084.67          NA     NA    20220602 2022   NA          NA        NA
## 636   75084.67          NA     NA    20220602 2022   NA          NA        NA
## 639   75084.67          NA     NA    20220602 2022   NA          NA        NA
## 645   75084.67          NA     NA    20220602 2022   NA          NA        NA
## 654   75084.67          NA     NA    20220602 2022   NA          NA        NA
## 656   75084.67          NA     NA    20220602 2022   NA          NA        NA
## 662   75084.67          NA     NA    20220602 2022   NA          NA        NA
## 672   75084.67          NA     NA    20220602 2022   NA          NA        NA
## 676   75084.67          NA     NA    20220602 2022   NA          NA        NA
## 684   75084.67          NA     NA    20220602 2022   NA          NA        NA
## 696   75084.67          NA     NA    20220602 2022   NA          NA        NA
## 702   75084.67          NA     NA    20220602 2022   NA          NA        NA
## 703   75084.67          NA     NA    20220602 2022   NA          NA        NA
## 713   75084.67          NA     NA    20220602 2022   NA          NA        NA
## 719   75084.67          NA     NA    20220602 2022   NA          NA        NA
## 726   75084.67          NA     NA    20220602 2022   NA          NA        NA
## 732   75084.67          NA     NA    20220602 2022   NA          NA        NA
## 734   75084.67          NA     NA    20220602 2022   NA          NA        NA
## 741   75084.67          NA     NA    20220602 2022   NA          NA        NA
## 747   75084.67          NA     NA    20220602 2022   NA          NA        NA
## 760   75084.67          NA     NA    20220602 2022   NA          NA        NA
## 790   75084.67          NA     NA    20220602 2022   NA          NA        NA
## 798   75084.67          NA     NA    20220602 2022   NA          NA        NA
## 804   75084.67          NA     NA    20220602 2022   NA          NA        NA
## 805   75084.67          NA     NA    20220602 2022   NA          NA        NA
## 817   75084.67          NA     NA    20220602 2022   NA          NA        NA
## 825   75084.67          NA     NA    20220602 2022   NA          NA        NA
## 830   75084.67          NA     NA    20220602 2022   NA          NA        NA
## 838   75084.67          NA     NA    20220602 2022   NA          NA        NA
## 857   75084.67          NA     NA    20220602 2022   NA          NA        NA
## 869   75084.67          NA     NA    20220602 2022   NA          NA        NA
## 875   75084.67          NA     NA    20220602 2022   NA          NA        NA
## 882   75084.67          NA     NA    20220602 2022   NA          NA        NA
## 888   75084.67          NA     NA    20220602 2022   NA          NA        NA
## 890   75084.67          NA     NA    20220602 2022   NA          NA        NA
##     Species Species_substrate Species_site Sorter Sex Carapace_length
## 596      NA                NA           NA     79  NA        249.3333
## 104      NA                NA           NA     79  NA        249.3333
## 518      NA                NA           NA     79  NA        249.3333
## 373      NA                NA           NA     79  NA        249.3333
## 155      NA                NA           NA     79  NA        249.3333
## 688      NA                NA           NA     79  NA        249.3333
## 466      NA                NA           NA     79  NA        249.3333
## 358      NA                NA           NA     79  NA        249.3333
## 816      NA                NA           NA     79  NA        249.3333
## 547      NA                NA           NA     79  NA        249.3333
## 752      NA                NA           NA     79  NA        249.3333
## 844      NA                NA           NA     79  NA        249.3333
## 544      NA                NA           NA     79  NA        249.3333
## 763      NA                NA           NA     79  NA        249.3333
## 771      NA                NA           NA     79  NA        249.3333
## 330      NA                NA           NA     79  NA        249.3333
## 309      NA                NA           NA     79  NA        249.3333
## 392      NA                NA           NA     79  NA        249.3333
```

```
## 353      NA                NA           NA     79  NA        249.3333
## 775      NA                NA           NA     79  NA        249.3333
## 848      NA                NA           NA     79  NA        249.3333
## 782      NA                NA           NA     79  NA        249.3333
## 204      NA                NA           NA     79  NA        249.3333
## 386      NA                NA           NA     79  NA        249.3333
## 174      NA                NA           NA     79  NA        249.3333
## 529      NA                NA           NA     79  NA        249.3333
## 444      NA                NA           NA     79  NA        249.3333
## 501      NA                NA           NA     79  NA        249.3333
## 48       NA                NA           NA     79  NA        249.3333
## 860      NA                NA           NA     79  NA        249.3333
## 218      NA                NA           NA     79  NA        249.3333
## 6        NA                NA           NA     79  NA        249.3333
## 8        NA                NA           NA     79  NA        249.3333
## 17       NA                NA           NA     79  NA        249.3333
## 20       NA                NA           NA     79  NA        249.3333
## 26       NA                NA           NA     79  NA        249.3333
## 36       NA                NA           NA     79  NA        249.3333
## 37       NA                NA           NA     79  NA        249.3333
## 54       NA                NA           NA     79  NA        249.3333
## 60       NA                NA           NA     79  NA        249.3333
## 62       NA                NA           NA     79  NA        249.3333
## 70       NA                NA           NA     79  NA        249.3333
## 74       NA                NA           NA     79  NA        249.3333
## 80       NA                NA           NA     79  NA        249.3333
## 90       NA                NA           NA     79  NA        249.3333
## 94       NA                NA           NA     79  NA        249.3333
## 102      NA                NA           NA     79  NA        249.3333
## 112      NA                NA           NA     79  NA        249.3333
## 120      NA                NA           NA     79  NA        249.3333
## 123      NA                NA           NA     79  NA        249.3333
## 131      NA                NA           NA     79  NA        249.3333
## 133      NA                NA           NA     79  NA        249.3333
## 144      NA                NA           NA     79  NA        249.3333
## 147      NA                NA           NA     79  NA        249.3333
## 162      NA                NA           NA     79  NA        249.3333
## 165      NA                NA           NA     79  NA        249.3333
## 177      NA                NA           NA     79  NA        249.3333
## 185      NA                NA           NA     79  NA        249.3333
## 192      NA                NA           NA     79  NA        249.3333
## 196      NA                NA           NA     79  NA        249.3333
## 209      NA                NA           NA     79  NA        249.3333
## 212      NA                NA           NA     79  NA        249.3333
## 228      NA                NA           NA     79  NA        249.3333
## 234      NA                NA           NA     79  NA        249.3333
## 237      NA                NA           NA     79  NA        249.3333
## 242      NA                NA           NA     79  NA        249.3333
## 249      NA                NA           NA     79  NA        249.3333
## 254      NA                NA           NA     79  NA        249.3333
## 261      NA                NA           NA     79  NA        249.3333
## 265      NA                NA           NA     79  NA        249.3333
## 273      NA                NA           NA     79  NA        249.3333
## 279      NA                NA           NA     79  NA        249.3333
```

```
## 287       NA                  NA            NA     79  NA         249.3333
## 294       NA                  NA            NA     79  NA         249.3333
## 300       NA                  NA            NA     79  NA         249.3333
## 304       NA                  NA            NA     79  NA         249.3333
## 315       NA                  NA            NA     79  NA         249.3333
## 321       NA                  NA            NA     79  NA         249.3333
## 336       NA                  NA            NA     79  NA         249.3333
## 340       NA                  NA            NA     79  NA         249.3333
## 343       NA                  NA            NA     79  NA         249.3333
## 366       NA                  NA            NA     79  NA         249.3333
## 372       NA                  NA            NA     79  NA         249.3333
## 383       NA                  NA            NA     79  NA         249.3333
## 401       NA                  NA            NA     79  NA         249.3333
## 407       NA                  NA            NA     79  NA         249.3333
## 410       NA                  NA            NA     79  NA         249.3333
## 417       NA                  NA            NA     79  NA         249.3333
## 424       NA                  NA            NA     79  NA         249.3333
## 429       NA                  NA            NA     79  NA         249.3333
## 435       NA                  NA            NA     79  NA         249.3333
## 446       NA                  NA            NA     79  NA         249.3333
## 456       NA                  NA            NA     79  NA         249.3333
## 459       NA                  NA            NA     79  NA         249.3333
## 471       NA                  NA            NA     79  NA         249.3333
## 477       NA                  NA            NA     79  NA         249.3333
## 483       NA                  NA            NA     79  NA         249.3333
## 488       NA                  NA            NA     79  NA         249.3333
## 495       NA                  NA            NA     79  NA         249.3333
## 508       NA                  NA            NA     79  NA         249.3333
## 513       NA                  NA            NA     79  NA         249.3333
## 524       NA                  NA            NA     79  NA         249.3333
## 536       NA                  NA            NA     79  NA         249.3333
## 553       NA                  NA            NA     79  NA         249.3333
## 560       NA                  NA            NA     79  NA         249.3333
## 566       NA                  NA            NA     79  NA         249.3333
## 576       NA                  NA            NA     79  NA         249.3333
## 578       NA                  NA            NA     79  NA         249.3333
## 584       NA                  NA            NA     79  NA         249.3333
## 591       NA                  NA            NA     79  NA         249.3333
## 602       NA                  NA            NA     79  NA         249.3333
## 611       NA                  NA            NA     79  NA         249.3333
## 616       NA                  NA            NA     79  NA         249.3333
## 621       NA                  NA            NA     79  NA         249.3333
## 627       NA                  NA            NA     79  NA         249.3333
## 636       NA                  NA            NA     79  NA         249.3333
## 639       NA                  NA            NA     79  NA         249.3333
## 645       NA                  NA            NA     79  NA         249.3333
## 654       NA                  NA            NA     79  NA         249.3333
## 656       NA                  NA            NA     79  NA         249.3333
## 662       NA                  NA            NA     79  NA         249.3333
## 672       NA                  NA            NA     79  NA         249.3333
## 676       NA                  NA            NA     79  NA         249.3333
## 684       NA                  NA            NA     79  NA         249.3333
## 696       NA                  NA            NA     79  NA         249.3333
## 702       NA                  NA            NA     79  NA         249.3333
```

```
## 703      NA                 NA            NA      79  NA         249.3333
## 713      NA                 NA            NA      79  NA         249.3333
## 719      NA                 NA            NA      79  NA         249.3333
## 726      NA                 NA            NA      79  NA         249.3333
## 732      NA                 NA            NA      79  NA         249.3333
## 734      NA                 NA            NA      79  NA         249.3333
## 741      NA                 NA            NA      79  NA         249.3333
## 747      NA                 NA            NA      79  NA         249.3333
## 760      NA                 NA            NA      79  NA         249.3333
## 790      NA                 NA            NA      79  NA         249.3333
## 798      NA                 NA            NA      79  NA         249.3333
## 804      NA                 NA            NA      79  NA         249.3333
## 805      NA                 NA            NA      79  NA         249.3333
## 817      NA                 NA            NA      79  NA         249.3333
## 825      NA                 NA            NA      79  NA         249.3333
## 830      NA                 NA            NA      79  NA         249.3333
## 838      NA                 NA            NA      79  NA         249.3333
## 857      NA                 NA            NA      79  NA         249.3333
## 869      NA                 NA            NA      79  NA         249.3333
## 875      NA                 NA            NA      79  NA         249.3333
## 882      NA                 NA            NA      79  NA         249.3333
## 888      NA                 NA            NA      79  NA         249.3333
## 890      NA                 NA            NA      79  NA         249.3333
##     Plastron_length Mass Sample_number Sex_notes PCR1_date extraction_date
## 596             184 3725            72        NA      2022        20220624
## 104             184 3725            72        NA      2022        20220624
## 518             184 3725            72        NA      2022        20220624
## 373             184 3725            72        NA      2022        20220624
## 155             184 3725            72        NA      2022        20220624
## 688             184 3725            72        NA      2022        20220624
## 466             184 3725            72        NA      2022        20220624
## 358             184 3725            72        NA      2022        20220624
## 816             184 3725            72        NA      2022        20220624
## 547             184 3725            72        NA      2022        20220624
## 752             184 3725            72        NA      2022        20220624
## 844             184 3725            72        NA      2022        20220624
## 544             184 3725            72        NA      2022        20220624
## 763             184 3725            72        NA      2022        20220624
## 771             184 3725            72        NA      2022        20220624
## 330             184 3725            72        NA      2022        20220624
## 309             184 3725            72        NA      2022        20220624
## 392             184 3725            72        NA      2022        20220624
## 353             184 3725            72        NA      2022        20220624
## 775             184 3725            72        NA      2022        20220624
## 848             184 3725            72        NA      2022        20220624
## 782             184 3725            72        NA      2022        20220624
## 204             184 3725            72        NA      2022        20220624
## 386             184 3725            72        NA      2022        20220624
## 174             184 3725            72        NA      2022        20220624
## 529             184 3725            72        NA      2022        20220624
## 444             184 3725            72        NA      2022        20220624
## 501             184 3725            72        NA      2022        20220624
## 48              184 3725            72        NA      2022        20220624
## 860             184 3725            72        NA      2022        20220624
```

```
## 218                 184 3725            72         NA      2022         20220624
## 6                   184 3725            72         NA      2022         20220624
## 8                   184 3725            72         NA      2022         20220624
## 17                  184 3725            72         NA      2022         20220624
## 20                  184 3725            72         NA      2022         20220624
## 26                  184 3725            72         NA      2022         20220624
## 36                  184 3725            72         NA      2022         20220624
## 37                  184 3725            72         NA      2022         20220624
## 54                  184 3725            72         NA      2022         20220624
## 60                  184 3725            72         NA      2022         20220624
## 62                  184 3725            72         NA      2022         20220624
## 70                  184 3725            72         NA      2022         20220624
## 74                  184 3725            72         NA      2022         20220624
## 80                  184 3725            72         NA      2022         20220624
## 90                  184 3725            72         NA      2022         20220624
## 94                  184 3725            72         NA      2022         20220624
## 102                 184 3725            72         NA      2022         20220624
## 112                 184 3725            72         NA      2022         20220624
## 120                 184 3725            72         NA      2022         20220624
## 123                 184 3725            72         NA      2022         20220624
## 131                 184 3725            72         NA      2022         20220624
## 133                 184 3725            72         NA      2022         20220624
## 144                 184 3725            72         NA      2022         20220624
## 147                 184 3725            72         NA      2022         20220624
## 162                 184 3725            72         NA      2022         20220624
## 165                 184 3725            72         NA      2022         20220624
## 177                 184 3725            72         NA      2022         20220624
## 185                 184 3725            72         NA      2022         20220624
## 192                 184 3725            72         NA      2022         20220624
## 196                 184 3725            72         NA      2022         20220624
## 209                 184 3725            72         NA      2022         20220624
## 212                 184 3725            72         NA      2022         20220624
## 228                 184 3725            72         NA      2022         20220624
## 234                 184 3725            72         NA      2022         20220624
## 237                 184 3725            72         NA      2022         20220624
## 242                 184 3725            72         NA      2022         20220624
## 249                 184 3725            72         NA      2022         20220624
## 254                 184 3725            72         NA      2022         20220624
## 261                 184 3725            72         NA      2022         20220624
## 265                 184 3725            72         NA      2022         20220624
## 273                 184 3725            72         NA      2022         20220624
## 279                 184 3725            72         NA      2022         20220624
## 287                 184 3725            72         NA      2022         20220624
## 294                 184 3725            72         NA      2022         20220624
## 300                 184 3725            72         NA      2022         20220624
## 304                 184 3725            72         NA      2022         20220624
## 315                 184 3725            72         NA      2022         20220624
## 321                 184 3725            72         NA      2022         20220624
## 336                 184 3725            72         NA      2022         20220624
## 340                 184 3725            72         NA      2022         20220624
## 343                 184 3725            72         NA      2022         20220624
## 366                 184 3725            72         NA      2022         20220624
## 372                 184 3725            72         NA      2022         20220624
## 383                 184 3725            72         NA      2022         20220624
```

```
## 401             184 3725            72        NA      2022        20220624
## 407             184 3725            72        NA      2022        20220624
## 410             184 3725            72        NA      2022        20220624
## 417             184 3725            72        NA      2022        20220624
## 424             184 3725            72        NA      2022        20220624
## 429             184 3725            72        NA      2022        20220624
## 435             184 3725            72        NA      2022        20220624
## 446             184 3725            72        NA      2022        20220624
## 456             184 3725            72        NA      2022        20220624
## 459             184 3725            72        NA      2022        20220624
## 471             184 3725            72        NA      2022        20220624
## 477             184 3725            72        NA      2022        20220624
## 483             184 3725            72        NA      2022        20220624
## 488             184 3725            72        NA      2022        20220624
## 495             184 3725            72        NA      2022        20220624
## 508             184 3725            72        NA      2022        20220624
## 513             184 3725            72        NA      2022        20220624
## 524             184 3725            72        NA      2022        20220624
## 536             184 3725            72        NA      2022        20220624
## 553             184 3725            72        NA      2022        20220624
## 560             184 3725            72        NA      2022        20220624
## 566             184 3725            72        NA      2022        20220624
## 576             184 3725            72        NA      2022        20220624
## 578             184 3725            72        NA      2022        20220624
## 584             184 3725            72        NA      2022        20220624
## 591             184 3725            72        NA      2022        20220624
## 602             184 3725            72        NA      2022        20220624
## 611             184 3725            72        NA      2022        20220624
## 616             184 3725            72        NA      2022        20220624
## 621             184 3725            72        NA      2022        20220624
## 627             184 3725            72        NA      2022        20220624
## 636             184 3725            72        NA      2022        20220624
## 639             184 3725            72        NA      2022        20220624
## 645             184 3725            72        NA      2022        20220624
## 654             184 3725            72        NA      2022        20220624
## 656             184 3725            72        NA      2022        20220624
## 662             184 3725            72        NA      2022        20220624
## 672             184 3725            72        NA      2022        20220624
## 676             184 3725            72        NA      2022        20220624
## 684             184 3725            72        NA      2022        20220624
## 696             184 3725            72        NA      2022        20220624
## 702             184 3725            72        NA      2022        20220624
## 703             184 3725            72        NA      2022        20220624
## 713             184 3725            72        NA      2022        20220624
## 719             184 3725            72        NA      2022        20220624
## 726             184 3725            72        NA      2022        20220624
## 732             184 3725            72        NA      2022        20220624
## 734             184 3725            72        NA      2022        20220624
## 741             184 3725            72        NA      2022        20220624
## 747             184 3725            72        NA      2022        20220624
## 760             184 3725            72        NA      2022        20220624
## 790             184 3725            72        NA      2022        20220624
## 798             184 3725            72        NA      2022        20220624
## 804             184 3725            72        NA      2022        20220624
```

```
## 805            184 3725           72         NA      2022        20220624
## 817            184 3725           72         NA      2022        20220624
## 825            184 3725           72         NA      2022        20220624
## 830            184 3725           72         NA      2022        20220624
## 838            184 3725           72         NA      2022        20220624
## 857            184 3725           72         NA      2022        20220624
## 869            184 3725           72         NA      2022        20220624
## 875            184 3725           72         NA      2022        20220624
## 882            184 3725           72         NA      2022        20220624
## 888            184 3725           72         NA      2022        20220624
## 890            184 3725           72         NA      2022        20220624
##     Observed    Chao1  se.chao1     ACE   se.ACE   Shannon   Simpson InvSimpson
## 596 19.33333 19.66667 0.7711357 19.5257 1.989387 0.2881333 0.1419233   1.224196
## 104 19.33333 19.66667 0.7711357 19.5257 1.989387 0.2881333 0.1419233   1.224196
## 518 19.33333 19.66667 0.7711357 19.5257 1.989387 0.2881333 0.1419233   1.224196
## 373 19.33333 19.66667 0.7711357 19.5257 1.989387 0.2881333 0.1419233   1.224196
## 155 19.33333 19.66667 0.7711357 19.5257 1.989387 0.2881333 0.1419233   1.224196
## 688 19.33333 19.66667 0.7711357 19.5257 1.989387 0.2881333 0.1419233   1.224196
## 466 19.33333 19.66667 0.7711357 19.5257 1.989387 0.2881333 0.1419233   1.224196
## 358 19.33333 19.66667 0.7711357 19.5257 1.989387 0.2881333 0.1419233   1.224196
## 816 19.33333 19.66667 0.7711357 19.5257 1.989387 0.2881333 0.1419233   1.224196
## 547 19.33333 19.66667 0.7711357 19.5257 1.989387 0.2881333 0.1419233   1.224196
## 752 19.33333 19.66667 0.7711357 19.5257 1.989387 0.2881333 0.1419233   1.224196
## 844 19.33333 19.66667 0.7711357 19.5257 1.989387 0.2881333 0.1419233   1.224196
## 544 19.33333 19.66667 0.7711357 19.5257 1.989387 0.2881333 0.1419233   1.224196
## 763 19.33333 19.66667 0.7711357 19.5257 1.989387 0.2881333 0.1419233   1.224196
## 771 19.33333 19.66667 0.7711357 19.5257 1.989387 0.2881333 0.1419233   1.224196
## 330 19.33333 19.66667 0.7711357 19.5257 1.989387 0.2881333 0.1419233   1.224196
## 309 19.33333 19.66667 0.7711357 19.5257 1.989387 0.2881333 0.1419233   1.224196
## 392 19.33333 19.66667 0.7711357 19.5257 1.989387 0.2881333 0.1419233   1.224196
## 353 19.33333 19.66667 0.7711357 19.5257 1.989387 0.2881333 0.1419233   1.224196
## 775 19.33333 19.66667 0.7711357 19.5257 1.989387 0.2881333 0.1419233   1.224196
## 848 19.33333 19.66667 0.7711357 19.5257 1.989387 0.2881333 0.1419233   1.224196
## 782 19.33333 19.66667 0.7711357 19.5257 1.989387 0.2881333 0.1419233   1.224196
## 204 19.33333 19.66667 0.7711357 19.5257 1.989387 0.2881333 0.1419233   1.224196
## 386 19.33333 19.66667 0.7711357 19.5257 1.989387 0.2881333 0.1419233   1.224196
## 174 19.33333 19.66667 0.7711357 19.5257 1.989387 0.2881333 0.1419233   1.224196
## 529 19.33333 19.66667 0.7711357 19.5257 1.989387 0.2881333 0.1419233   1.224196
## 444 19.33333 19.66667 0.7711357 19.5257 1.989387 0.2881333 0.1419233   1.224196
## 501 19.33333 19.66667 0.7711357 19.5257 1.989387 0.2881333 0.1419233   1.224196
## 48  19.33333 19.66667 0.7711357 19.5257 1.989387 0.2881333 0.1419233   1.224196
## 860 19.33333 19.66667 0.7711357 19.5257 1.989387 0.2881333 0.1419233   1.224196
## 218 19.33333 19.66667 0.7711357 19.5257 1.989387 0.2881333 0.1419233   1.224196
## 6   19.33333 19.66667 0.7711357 19.5257 1.989387 0.2881333 0.1419233   1.224196
## 8   19.33333 19.66667 0.7711357 19.5257 1.989387 0.2881333 0.1419233   1.224196
## 17  19.33333 19.66667 0.7711357 19.5257 1.989387 0.2881333 0.1419233   1.224196
## 20  19.33333 19.66667 0.7711357 19.5257 1.989387 0.2881333 0.1419233   1.224196
## 26  19.33333 19.66667 0.7711357 19.5257 1.989387 0.2881333 0.1419233   1.224196
## 36  19.33333 19.66667 0.7711357 19.5257 1.989387 0.2881333 0.1419233   1.224196
## 37  19.33333 19.66667 0.7711357 19.5257 1.989387 0.2881333 0.1419233   1.224196
## 54  19.33333 19.66667 0.7711357 19.5257 1.989387 0.2881333 0.1419233   1.224196
## 60  19.33333 19.66667 0.7711357 19.5257 1.989387 0.2881333 0.1419233   1.224196
## 62  19.33333 19.66667 0.7711357 19.5257 1.989387 0.2881333 0.1419233   1.224196
## 70  19.33333 19.66667 0.7711357 19.5257 1.989387 0.2881333 0.1419233   1.224196
```

```
## 74  19.33333 19.66667 0.7711357 19.5257 1.989387 0.2881333 0.1419233   1.224196
## 80  19.33333 19.66667 0.7711357 19.5257 1.989387 0.2881333 0.1419233   1.224196
## 90  19.33333 19.66667 0.7711357 19.5257 1.989387 0.2881333 0.1419233   1.224196
## 94  19.33333 19.66667 0.7711357 19.5257 1.989387 0.2881333 0.1419233   1.224196
## 102 19.33333 19.66667 0.7711357 19.5257 1.989387 0.2881333 0.1419233   1.224196
## 112 19.33333 19.66667 0.7711357 19.5257 1.989387 0.2881333 0.1419233   1.224196
## 120 19.33333 19.66667 0.7711357 19.5257 1.989387 0.2881333 0.1419233   1.224196
## 123 19.33333 19.66667 0.7711357 19.5257 1.989387 0.2881333 0.1419233   1.224196
## 131 19.33333 19.66667 0.7711357 19.5257 1.989387 0.2881333 0.1419233   1.224196
## 133 19.33333 19.66667 0.7711357 19.5257 1.989387 0.2881333 0.1419233   1.224196
## 144 19.33333 19.66667 0.7711357 19.5257 1.989387 0.2881333 0.1419233   1.224196
## 147 19.33333 19.66667 0.7711357 19.5257 1.989387 0.2881333 0.1419233   1.224196
## 162 19.33333 19.66667 0.7711357 19.5257 1.989387 0.2881333 0.1419233   1.224196
## 165 19.33333 19.66667 0.7711357 19.5257 1.989387 0.2881333 0.1419233   1.224196
## 177 19.33333 19.66667 0.7711357 19.5257 1.989387 0.2881333 0.1419233   1.224196
## 185 19.33333 19.66667 0.7711357 19.5257 1.989387 0.2881333 0.1419233   1.224196
## 192 19.33333 19.66667 0.7711357 19.5257 1.989387 0.2881333 0.1419233   1.224196
## 196 19.33333 19.66667 0.7711357 19.5257 1.989387 0.2881333 0.1419233   1.224196
## 209 19.33333 19.66667 0.7711357 19.5257 1.989387 0.2881333 0.1419233   1.224196
## 212 19.33333 19.66667 0.7711357 19.5257 1.989387 0.2881333 0.1419233   1.224196
## 228 19.33333 19.66667 0.7711357 19.5257 1.989387 0.2881333 0.1419233   1.224196
## 234 19.33333 19.66667 0.7711357 19.5257 1.989387 0.2881333 0.1419233   1.224196
## 237 19.33333 19.66667 0.7711357 19.5257 1.989387 0.2881333 0.1419233   1.224196
## 242 19.33333 19.66667 0.7711357 19.5257 1.989387 0.2881333 0.1419233   1.224196
## 249 19.33333 19.66667 0.7711357 19.5257 1.989387 0.2881333 0.1419233   1.224196
## 254 19.33333 19.66667 0.7711357 19.5257 1.989387 0.2881333 0.1419233   1.224196
## 261 19.33333 19.66667 0.7711357 19.5257 1.989387 0.2881333 0.1419233   1.224196
## 265 19.33333 19.66667 0.7711357 19.5257 1.989387 0.2881333 0.1419233   1.224196
## 273 19.33333 19.66667 0.7711357 19.5257 1.989387 0.2881333 0.1419233   1.224196
## 279 19.33333 19.66667 0.7711357 19.5257 1.989387 0.2881333 0.1419233   1.224196
## 287 19.33333 19.66667 0.7711357 19.5257 1.989387 0.2881333 0.1419233   1.224196
## 294 19.33333 19.66667 0.7711357 19.5257 1.989387 0.2881333 0.1419233   1.224196
## 300 19.33333 19.66667 0.7711357 19.5257 1.989387 0.2881333 0.1419233   1.224196
## 304 19.33333 19.66667 0.7711357 19.5257 1.989387 0.2881333 0.1419233   1.224196
## 315 19.33333 19.66667 0.7711357 19.5257 1.989387 0.2881333 0.1419233   1.224196
## 321 19.33333 19.66667 0.7711357 19.5257 1.989387 0.2881333 0.1419233   1.224196
## 336 19.33333 19.66667 0.7711357 19.5257 1.989387 0.2881333 0.1419233   1.224196
## 340 19.33333 19.66667 0.7711357 19.5257 1.989387 0.2881333 0.1419233   1.224196
## 343 19.33333 19.66667 0.7711357 19.5257 1.989387 0.2881333 0.1419233   1.224196
## 366 19.33333 19.66667 0.7711357 19.5257 1.989387 0.2881333 0.1419233   1.224196
## 372 19.33333 19.66667 0.7711357 19.5257 1.989387 0.2881333 0.1419233   1.224196
## 383 19.33333 19.66667 0.7711357 19.5257 1.989387 0.2881333 0.1419233   1.224196
## 401 19.33333 19.66667 0.7711357 19.5257 1.989387 0.2881333 0.1419233   1.224196
## 407 19.33333 19.66667 0.7711357 19.5257 1.989387 0.2881333 0.1419233   1.224196
## 410 19.33333 19.66667 0.7711357 19.5257 1.989387 0.2881333 0.1419233   1.224196
## 417 19.33333 19.66667 0.7711357 19.5257 1.989387 0.2881333 0.1419233   1.224196
## 424 19.33333 19.66667 0.7711357 19.5257 1.989387 0.2881333 0.1419233   1.224196
## 429 19.33333 19.66667 0.7711357 19.5257 1.989387 0.2881333 0.1419233   1.224196
## 435 19.33333 19.66667 0.7711357 19.5257 1.989387 0.2881333 0.1419233   1.224196
## 446 19.33333 19.66667 0.7711357 19.5257 1.989387 0.2881333 0.1419233   1.224196
## 456 19.33333 19.66667 0.7711357 19.5257 1.989387 0.2881333 0.1419233   1.224196
## 459 19.33333 19.66667 0.7711357 19.5257 1.989387 0.2881333 0.1419233   1.224196
## 471 19.33333 19.66667 0.7711357 19.5257 1.989387 0.2881333 0.1419233   1.224196
## 477 19.33333 19.66667 0.7711357 19.5257 1.989387 0.2881333 0.1419233   1.224196
```

```
## 483 19.33333 19.66667 0.7711357 19.5257 1.989387 0.2881333 0.1419233   1.224196
## 488 19.33333 19.66667 0.7711357 19.5257 1.989387 0.2881333 0.1419233   1.224196
## 495 19.33333 19.66667 0.7711357 19.5257 1.989387 0.2881333 0.1419233   1.224196
## 508 19.33333 19.66667 0.7711357 19.5257 1.989387 0.2881333 0.1419233   1.224196
## 513 19.33333 19.66667 0.7711357 19.5257 1.989387 0.2881333 0.1419233   1.224196
## 524 19.33333 19.66667 0.7711357 19.5257 1.989387 0.2881333 0.1419233   1.224196
## 536 19.33333 19.66667 0.7711357 19.5257 1.989387 0.2881333 0.1419233   1.224196
## 553 19.33333 19.66667 0.7711357 19.5257 1.989387 0.2881333 0.1419233   1.224196
## 560 19.33333 19.66667 0.7711357 19.5257 1.989387 0.2881333 0.1419233   1.224196
## 566 19.33333 19.66667 0.7711357 19.5257 1.989387 0.2881333 0.1419233   1.224196
## 576 19.33333 19.66667 0.7711357 19.5257 1.989387 0.2881333 0.1419233   1.224196
## 578 19.33333 19.66667 0.7711357 19.5257 1.989387 0.2881333 0.1419233   1.224196
## 584 19.33333 19.66667 0.7711357 19.5257 1.989387 0.2881333 0.1419233   1.224196
## 591 19.33333 19.66667 0.7711357 19.5257 1.989387 0.2881333 0.1419233   1.224196
## 602 19.33333 19.66667 0.7711357 19.5257 1.989387 0.2881333 0.1419233   1.224196
## 611 19.33333 19.66667 0.7711357 19.5257 1.989387 0.2881333 0.1419233   1.224196
## 616 19.33333 19.66667 0.7711357 19.5257 1.989387 0.2881333 0.1419233   1.224196
## 621 19.33333 19.66667 0.7711357 19.5257 1.989387 0.2881333 0.1419233   1.224196
## 627 19.33333 19.66667 0.7711357 19.5257 1.989387 0.2881333 0.1419233   1.224196
## 636 19.33333 19.66667 0.7711357 19.5257 1.989387 0.2881333 0.1419233   1.224196
## 639 19.33333 19.66667 0.7711357 19.5257 1.989387 0.2881333 0.1419233   1.224196
## 645 19.33333 19.66667 0.7711357 19.5257 1.989387 0.2881333 0.1419233   1.224196
## 654 19.33333 19.66667 0.7711357 19.5257 1.989387 0.2881333 0.1419233   1.224196
## 656 19.33333 19.66667 0.7711357 19.5257 1.989387 0.2881333 0.1419233   1.224196
## 662 19.33333 19.66667 0.7711357 19.5257 1.989387 0.2881333 0.1419233   1.224196
## 672 19.33333 19.66667 0.7711357 19.5257 1.989387 0.2881333 0.1419233   1.224196
## 676 19.33333 19.66667 0.7711357 19.5257 1.989387 0.2881333 0.1419233   1.224196
## 684 19.33333 19.66667 0.7711357 19.5257 1.989387 0.2881333 0.1419233   1.224196
## 696 19.33333 19.66667 0.7711357 19.5257 1.989387 0.2881333 0.1419233   1.224196
## 702 19.33333 19.66667 0.7711357 19.5257 1.989387 0.2881333 0.1419233   1.224196
## 703 19.33333 19.66667 0.7711357 19.5257 1.989387 0.2881333 0.1419233   1.224196
## 713 19.33333 19.66667 0.7711357 19.5257 1.989387 0.2881333 0.1419233   1.224196
## 719 19.33333 19.66667 0.7711357 19.5257 1.989387 0.2881333 0.1419233   1.224196
## 726 19.33333 19.66667 0.7711357 19.5257 1.989387 0.2881333 0.1419233   1.224196
## 732 19.33333 19.66667 0.7711357 19.5257 1.989387 0.2881333 0.1419233   1.224196
## 734 19.33333 19.66667 0.7711357 19.5257 1.989387 0.2881333 0.1419233   1.224196
## 741 19.33333 19.66667 0.7711357 19.5257 1.989387 0.2881333 0.1419233   1.224196
## 747 19.33333 19.66667 0.7711357 19.5257 1.989387 0.2881333 0.1419233   1.224196
## 760 19.33333 19.66667 0.7711357 19.5257 1.989387 0.2881333 0.1419233   1.224196
## 790 19.33333 19.66667 0.7711357 19.5257 1.989387 0.2881333 0.1419233   1.224196
## 798 19.33333 19.66667 0.7711357 19.5257 1.989387 0.2881333 0.1419233   1.224196
## 804 19.33333 19.66667 0.7711357 19.5257 1.989387 0.2881333 0.1419233   1.224196
## 805 19.33333 19.66667 0.7711357 19.5257 1.989387 0.2881333 0.1419233   1.224196
## 817 19.33333 19.66667 0.7711357 19.5257 1.989387 0.2881333 0.1419233   1.224196
## 825 19.33333 19.66667 0.7711357 19.5257 1.989387 0.2881333 0.1419233   1.224196
## 830 19.33333 19.66667 0.7711357 19.5257 1.989387 0.2881333 0.1419233   1.224196
## 838 19.33333 19.66667 0.7711357 19.5257 1.989387 0.2881333 0.1419233   1.224196
## 857 19.33333 19.66667 0.7711357 19.5257 1.989387 0.2881333 0.1419233   1.224196
## 869 19.33333 19.66667 0.7711357 19.5257 1.989387 0.2881333 0.1419233   1.224196
## 875 19.33333 19.66667 0.7711357 19.5257 1.989387 0.2881333 0.1419233   1.224196
## 882 19.33333 19.66667 0.7711357 19.5257 1.989387 0.2881333 0.1419233   1.224196
## 888 19.33333 19.66667 0.7711357 19.5257 1.989387 0.2881333 0.1419233   1.224196
## 890 19.33333 19.66667 0.7711357 19.5257 1.989387 0.2881333 0.1419233   1.224196
##     log10_read_depth       PD    Domain                            Phylum
```

```
## 596         4.874951 5.840054 Eukaryota                       Chlorophyta
## 104         4.874951 5.840054 Eukaryota                        Ciliophora
## 518         4.874951 5.840054 Eukaryota                          Annelida
## 373         4.874951 5.840054 Eukaryota                          Annelida
## 155         4.874951 5.840054 Eukaryota                   Platyhelminthes
## 688         4.874951 5.840054 Eukaryota                Peronosporomycetes
## 466         4.874951 5.840054 Eukaryota                       Chlorophyta
## 358         4.874951 5.840054 Eukaryota                        Ciliophora
## 816         4.874951 5.840054 Eukaryota                        Ciliophora
## 547         4.874951 5.840054 Eukaryota                          Annelida
## 752         4.874951 5.840054 Eukaryota                        Ciliophora
## 844         4.874951 5.840054 Eukaryota                          Diatomea
## 544         4.874951 5.840054 Eukaryota                      Gastrotricha
## 763         4.874951 5.840054 Eukaryota                         Nematozoa
## 771         4.874951 5.840054 Eukaryota                          Cercozoa
## 330         4.874951 5.840054 Eukaryota                         Nematozoa
## 309         4.874951 5.840054 Eukaryota                        Ciliophora
## 392         4.874951 5.840054 Eukaryota                       Chlorophyta
## 353         4.874951 5.840054 Eukaryota                        Ochrophyta
## 775         4.874951 5.840054 Eukaryota                          Diatomea
## 848         4.874951 5.840054 Eukaryota                        Ciliophora
## 782         4.874951 5.840054 Eukaryota                          Diatomea
## 204         4.874951 5.840054 Eukaryota                         Amoebozoa
## 386         4.874951 5.840054 Eukaryota                       Chlorophyta
## 174         4.874951 5.840054 Eukaryota                   Platyhelminthes
## 529         4.874951 5.840054 Eukaryota                          Mollusca
## 444         4.874951 5.840054 Eukaryota                   Platyhelminthes
## 501         4.874951 5.840054 Eukaryota                          Diatomea
## 48          4.874951 5.840054 Eukaryota                        Ciliophora
## 860         4.874951 5.840054 Eukaryota                          Cercozoa
## 218         4.874951 5.840054 Eukaryota                        Ciliophora
## 6           4.874951 5.840054 Eukaryota                        Ascomycota
## 8           4.874951 5.840054 Eukaryota                        Ciliophora
## 17          4.874951 5.840054 Eukaryota                         Amoebozoa
## 20          4.874951 5.840054 Eukaryota                        Ciliophora
## 26          4.874951 5.840054 Eukaryota                          Cercozoa
## 36          4.874951 5.840054 Eukaryota                   Platyhelminthes
## 37          4.874951 5.840054 Eukaryota                          Diatomea
## 54          4.874951 5.840054 Eukaryota                Peronosporomycetes
## 60          4.874951 5.840054 Eukaryota                        Ciliophora
## 62          4.874951 5.840054 Eukaryota                       Apicomplexa
## 70          4.874951 5.840054 Eukaryota                        Ciliophora
## 74          4.874951 5.840054 Eukaryota                      Centrohelida
## 80          4.874951 5.840054 Eukaryota                          Diatomea
## 90          4.874951 5.840054 Eukaryota                        Ciliophora
## 94          4.874951 5.840054 Eukaryota                  Chytridiomycota
## 102         4.874951 5.840054 Eukaryota                Blastocladiomycota
## 112         4.874951 5.840054 Eukaryota                         Amoebozoa
## 120         4.874951 5.840054 Eukaryota                         Amoebozoa
## 123         4.874951 5.840054 Eukaryota                        Ciliophora
## 131         4.874951 5.840054 Eukaryota                   Platyhelminthes
## 133         4.874951 5.840054 Eukaryota                          Cercozoa
## 144         4.874951 5.840054 Eukaryota Nucleariidae_and_Fonticula_group
## 147         4.874951 5.840054 Eukaryota                        Ciliophora
```

```
## 162         4.874951 5.840054 Eukaryota                        Ciliophora
## 165         4.874951 5.840054 Eukaryota                        Ciliophora
## 177         4.874951 5.840054 Eukaryota                      Gastrotricha
## 185         4.874951 5.840054 Eukaryota                        Ciliophora
## 192         4.874951 5.840054 Eukaryota                Phragmoplastophyta
## 196         4.874951 5.840054 Eukaryota                        Ciliophora
## 209         4.874951 5.840054 Eukaryota                        Ciliophora
## 212         4.874951 5.840054 Eukaryota                       Chlorophyta
## 228         4.874951 5.840054 Eukaryota                Phragmoplastophyta
## 234         4.874951 5.840054 Eukaryota                        Ascomycota
## 237         4.874951 5.840054 Eukaryota                        Ciliophora
## 242         4.874951 5.840054 Eukaryota                        Ciliophora
## 249         4.874951 5.840054 Eukaryota                          Cercozoa
## 254         4.874951 5.840054 Eukaryota                        Ciliophora
## 261         4.874951 5.840054 Eukaryota                        Ciliophora
## 265         4.874951 5.840054 Eukaryota                       Chlorophyta
## 273         4.874951 5.840054 Eukaryota                        Ciliophora
## 279         4.874951 5.840054 Eukaryota                        Ciliophora
## 287         4.874951 5.840054 Eukaryota                          Annelida
## 294         4.874951 5.840054 Eukaryota                        Ciliophora
## 300         4.874951 5.840054 Eukaryota                         Nematozoa
## 304         4.874951 5.840054 Eukaryota                        Ascomycota
## 315         4.874951 5.840054 Eukaryota                        Ciliophora
## 321         4.874951 5.840054 Eukaryota                        Ciliophora
## 336         4.874951 5.840054 Eukaryota                        Ciliophora
## 340         4.874951 5.840054 Eukaryota                        Ciliophora
## 343         4.874951 5.840054 Eukaryota                        Ciliophora
## 366         4.874951 5.840054 Eukaryota                          Diatomea
## 372         4.874951 5.840054 Eukaryota                        Ciliophora
## 383         4.874951 5.840054 Eukaryota                        Ochrophyta
## 401         4.874951 5.840054 Eukaryota                          Cercozoa
## 407         4.874951 5.840054 Eukaryota                        Ciliophora
## 410         4.874951 5.840054 Eukaryota                         Amoebozoa
## 417         4.874951 5.840054 Eukaryota                           Bryozoa
## 424         4.874951 5.840054 Eukaryota                        Ciliophora
## 429         4.874951 5.840054 Eukaryota                          Diatomea
## 435         4.874951 5.840054 Eukaryota                     Protalveolata
## 446         4.874951 5.840054 Eukaryota                          Rotifera
## 456         4.874951 5.840054 Eukaryota                        Ciliophora
## 459         4.874951 5.840054 Eukaryota                        Ciliophora
## 471         4.874951 5.840054 Eukaryota                        Ciliophora
## 477         4.874951 5.840054 Eukaryota                        Ciliophora
## 483         4.874951 5.840054 Eukaryota                    Dinoflagellata
## 488         4.874951 5.840054 Eukaryota                    Dinoflagellata
## 495         4.874951 5.840054 Eukaryota                        Ciliophora
## 508         4.874951 5.840054 Eukaryota                          Cercozoa
## 513         4.874951 5.840054 Eukaryota                          Porifera
## 524         4.874951 5.840054 Eukaryota                        Ciliophora
## 536         4.874951 5.840054 Eukaryota                        Ciliophora
## 553         4.874951 5.840054 Eukaryota                        Ciliophora
## 560         4.874951 5.840054 Eukaryota                        Ascomycota
## 566         4.874951 5.840054 Eukaryota                       Apicomplexa
## 576         4.874951 5.840054 Eukaryota                Phragmoplastophyta
## 578         4.874951 5.840054 Eukaryota                        Ascomycota
```

```
## 584         4.874951 5.840054 Eukaryota                 Eukaryota Domain
## 591         4.874951 5.840054 Eukaryota                       Ciliophora
## 602         4.874951 5.840054 Eukaryota                         Diatomea
## 611         4.874951 5.840054 Eukaryota                       Ciliophora
## 616         4.874951 5.840054 Eukaryota                       Ciliophora
## 621         4.874951 5.840054 Eukaryota                       Ciliophora
## 627         4.874951 5.840054 Eukaryota                       Ciliophora
## 636         4.874951 5.840054 Eukaryota                       Ciliophora
## 639         4.874951 5.840054 Eukaryota                       Ciliophora
## 645         4.874951 5.840054 Eukaryota                       Ciliophora
## 654         4.874951 5.840054 Eukaryota               Phragmoplastophyta
## 656         4.874951 5.840054 Eukaryota                      Chlorophyta
## 662         4.874951 5.840054 Eukaryota                       Ciliophora
## 672         4.874951 5.840054 Eukaryota                       Ciliophora
## 676         4.874951 5.840054 Eukaryota                       Ciliophora
## 684         4.874951 5.840054 Eukaryota                       Ciliophora
## 696         4.874951 5.840054 Eukaryota                    Cryptophyceae
## 702         4.874951 5.840054 Eukaryota                       Ciliophora
## 703         4.874951 5.840054 Eukaryota                    Basidiomycota
## 713         4.874951 5.840054 Eukaryota                 Eukaryota Domain
## 719         4.874951 5.840054 Eukaryota                       Ciliophora
## 726         4.874951 5.840054 Eukaryota                  Platyhelminthes
## 732         4.874951 5.840054 Eukaryota               Phragmoplastophyta
## 734         4.874951 5.840054 Eukaryota                       Ciliophora
## 741         4.874951 5.840054 Eukaryota                       Ciliophora
## 747         4.874951 5.840054 Eukaryota                       Ciliophora
## 760         4.874951 5.840054 Eukaryota                       Ciliophora
## 790         4.874951 5.840054 Eukaryota                         Diatomea
## 798         4.874951 5.840054 Eukaryota                       Ascomycota
## 804         4.874951 5.840054 Eukaryota               Peronosporomycetes
## 805         4.874951 5.840054 Eukaryota                         Diatomea
## 817         4.874951 5.840054 Eukaryota                       Ciliophora
## 825         4.874951 5.840054 Eukaryota                       Ochrophyta
## 830         4.874951 5.840054 Eukaryota                       Ciliophora
## 838         4.874951 5.840054 Eukaryota                       Ciliophora
## 857         4.874951 5.840054 Eukaryota                       Rigifilida
## 869         4.874951 5.840054 Eukaryota                       Ciliophora
## 875         4.874951 5.840054 Eukaryota                         Cercozoa
## 882         4.874951 5.840054 Eukaryota                       Ascomycota
## 888         4.874951 5.840054 Eukaryota                       Ciliophora
## 890         4.874951 5.840054 Eukaryota                       Ciliophora
##                              Class                             Order
## 596                    Ulvophyceae                      Cladophorales
## 104             Intramacronucleata                          Conthreep
## 518                     Clitellata                        Haplotaxida
## 373                     Clitellata                    Rhynchobdellida
## 155                     Catenulida                         Catenulida
## 688             Peronosporomycetes                 Peronosporomycetes
## 466              Chlorophyta Phylum                Chlorophyta Phylum
## 358         Postciliodesmatophora                     Heterotrichea
## 816             Intramacronucleata                          Conthreep
## 547                     Clitellata                  Clitellata Class
## 752             Intramacronucleata                          Conthreep
## 844             Bacillariophyceae                  Bacillariophyceae
```

```
## 544                      Gastrotricha                        Chaetonotida
## 763                       Chromadorea                        Monhysterida
## 771                      Thecofilosea                        Cryomonadida
## 330                           Enoplea                        Triplonchida
## 309            Postciliodesmatophora                       Heterotrichea
## 392                     Chlorophyceae                Chlorophyceae Class
## 353                     Chrysophyceae                      Ochromonadales
## 775                 Bacillariophyceae                   Bacillariophyceae
## 848                Intramacronucleata                           Conthreep
## 782                 Bacillariophyceae                   Bacillariophyceae
## 204                         Tubulinea                         Arcellinida
## 386                       Ulvophyceae                        Cladophorales
## 174                    Rhabditophora                         Rhabdocoela
## 529                        Gastropoda                      Heterobranchia
## 444                    Rhabditophora                        Macrostomida
## 501                 Bacillariophyceae                   Bacillariophyceae
## 48                 Intramacronucleata                           Conthreep
## 860                     Vampyrellidae                       Vampyrellidae
## 218                Intramacronucleata                           Conthreep
## 6                      Eurotiomycetes                          Onygenales
## 8                  Intramacronucleata                           Conthreep
## 17                           Discosea                        Centramoebida
## 20                 Intramacronucleata                           Conthreep
## 26                         Imbricatea                      Spongomonadida
## 36                          Monogenea                   Monopisthocotylea
## 37               Coscinodiscophytina                        Fragilariales
## 54                 Peronosporomycetes                  Peronosporomycetes
## 60                 Intramacronucleata                        Spirotrichea
## 62                         Conoidasida                        Gregarinasina
## 70              Postciliodesmatophora                       Heterotrichea
## 74                       Centrohelida                        Centrohelida
## 80                  Bacillariophyceae                   Bacillariophyceae
## 90                 Intramacronucleata                           Conthreep
## 94                  Chytridiomycetes                       Rhizophydiales
## 102              Blastocladiomycetes                      Blastocladiales
## 112                         Tubulinea                          Euamoebida
## 120                         Tubulinea                        Echinamoebida
## 123                Intramacronucleata                           Conthreep
## 131            Platyhelminthes Phylum               Platyhelminthes Phylum
## 133                      Thecofilosea                       Incertae_Sedis
## 144 Nucleariidae_and_Fonticula_group Nucleariidae_and_Fonticula_group
## 147                Intramacronucleata                        Spirotrichea
## 162             Postciliodesmatophora                       Karyorelictea
## 165                Intramacronucleata                           Conthreep
## 177                      Gastrotricha                        Chaetonotida
## 185                Intramacronucleata                           Conthreep
## 192                       Embryophyta                          Liliopsida
## 196                Intramacronucleata                         Litostomatea
## 209                Intramacronucleata                        Spirotrichea
## 212                 Trebouxiophyceae               Trebouxiophyceae Class
## 228                       Embryophyta                          Liliopsida
## 234                   Dothideomycetes                         Pleosporales
## 237                Intramacronucleata                         Litostomatea
## 242                Intramacronucleata                        Spirotrichea
```

```
## 249              Vampyrellidae             Vampyrellidae
## 254        Intramacronucleata                 Conthreep
## 261        Intramacronucleata                 Conthreep
## 265               Ulvophyceae       Scotinosphaerales
## 273        Intramacronucleata              Spirotrichea
## 279        Intramacronucleata                 Conthreep
## 287           Annelida Phylum           Annelida Phylum
## 294        Intramacronucleata              Spirotrichea
## 300                   Enoplea               Dorylaimia
## 304           Dothideomycetes               Capnodiales
## 315        Intramacronucleata              Litostomatea
## 321        Intramacronucleata                 Conthreep
## 336        Intramacronucleata                Armophorea
## 340        Intramacronucleata              Spirotrichea
## 343        Intramacronucleata              Litostomatea
## 366         Bacillariophyceae         Bacillariophyceae
## 372        Intramacronucleata              Spirotrichea
## 383             Chrysophyceae             Chromulinales
## 401                Imbricatea                Imbricatea
## 407        Intramacronucleata              Spirotrichea
## 410                  Discosea                  Discosea
## 417           Phylactolaemata               Plumatellida
## 424        Intramacronucleata                 Conthreep
## 429           Diatomea Phylum           Diatomea Phylum
## 435               Perkinsidae         Perkinsidae Class
## 446                Bdelloidea                 Adinetida
## 456        Intramacronucleata              Spirotrichea
## 459        Intramacronucleata                 Conthreep
## 471        Intramacronucleata              Litostomatea
## 477        Intramacronucleata                 Conthreep
## 483               Dinophyceae         Dinophyceae Class
## 488      Dinoflagellata Phylum     Dinoflagellata Phylum
## 495        Intramacronucleata                Armophorea
## 508                Imbricatea                Imbricatea
## 513              Demospongiae                Spongillida
## 524        Intramacronucleata              Litostomatea
## 536        Intramacronucleata              Spirotrichea
## 553        Intramacronucleata                Armophorea
## 560           Dothideomycetes               Dothideales
## 566               Conoidasida              Gregarinasina
## 576               Embryophyta               Salviniales
## 578           Dothideomycetes               Capnodiales
## 584       Labyrinthulomycetes       Labyrinthulomycetes
## 591        Intramacronucleata                 Conthreep
## 602         Bacillariophyceae         Bacillariophyceae
## 611        Intramacronucleata                 Conthreep
## 616        Intramacronucleata                 Conthreep
## 621        Intramacronucleata                 Conthreep
## 627        Intramacronucleata              Litostomatea
## 636        Intramacronucleata                 Conthreep
## 639      Postciliodesmatophora            Heterotrichea
## 645        Intramacronucleata              Litostomatea
## 654               Embryophyta         Embryophyta Class
## 656             Chlorophyceae            Chaetophorales
```

```
## 662              Intramacronucleata                      Litostomatea
## 672              Intramacronucleata                         Conthreep
## 676              Intramacronucleata                         Conthreep
## 684              Intramacronucleata                         Conthreep
## 696                   Cryptophyceae                   Cryptomonadales
## 702              Intramacronucleata                      Spirotrichea
## 703               Malasseziomycetes                    Malasseziales
## 713                 Eukaryota Domain                  Eukaryota Domain
## 719              Intramacronucleata                         Conthreep
## 726                   Rhabditophora                       Rhabdocoela
## 732                     Embryophyta                         Asterales
## 734              Intramacronucleata                         Conthreep
## 741              Intramacronucleata                      Litostomatea
## 747              Intramacronucleata                      Spirotrichea
## 760              Intramacronucleata                         Conthreep
## 790               Bacillariophyceae                 Bacillariophyceae
## 798                 Dothideomycetes                      Pleosporales
## 804              Peronosporomycetes                Peronosporomycetes
## 805               Bacillariophyceae                 Bacillariophyceae
## 817              Intramacronucleata                      Spirotrichea
## 825                   Chrysophyceae               Chrysophyceae Class
## 830              Intramacronucleata                      Spirotrichea
## 838              Intramacronucleata                         Conthreep
## 857                      Rigifilida                        Rigifilida
## 869              Intramacronucleata                         Conthreep
## 875                  Novel_Clade_12                    Novel_Clade_12
## 882                 Dothideomycetes                      Pleosporales
## 888              Intramacronucleata                      Spirotrichea
## 890              Intramacronucleata                      Spirotrichea
##                     Family                        Genus
## 596          Cladophorales                  Aegagropila
## 104      Oligohymenophorea               Telotrochidium
## 518            Haplotaxida                  Haplotaxida
## 373        Rhynchobdellida              Rhynchobdellida
## 155          Stenostomidae                  Stenostomum
## 688     Peronosporomycetes                      Pythium
## 466     Chlorophyta Phylum           Chlorophyta Phylum
## 358          Heterotrichea                      Stentor
## 816      Oligohymenophorea     Oligohymenophorea Family
## 547       Clitellata Class             Clitellata Class
## 752        Phyllopharyngea                   Heliophrya
## 844      Bacillariophyceae                   Gomphonema
## 544           Chaetonotida                 Chaetonotida
## 763           Monhysterida                 Monhysterida
## 771          Rhizaspididae                   Rhogostoma
## 330           Triplonchida                 Triplonchida
## 309          Heterotrichea         Heterotrichea Family
## 392    Chlorophyceae Class          Chlorophyceae Class
## 353         Ochromonadales                   Ochromonas
## 775      Bacillariophyceae                    Nitzschia
## 848      Oligohymenophorea                   Vorticella
## 782      Bacillariophyceae                   Pinnularia
## 204            Arcellinida                  Arcellinida
## 386          Cladophorales         Cladophorales Family
```

```
## 174          Neodalyellida                     Gieysztoria
## 529         Heterobranchia                  Heterobranchia
## 444           Macrostomida                    Macrostomida
## 501      Bacillariophyceae                        Navicula
## 48       Oligohymenophorea                        Stokesia
## 860          Vampyrellidae                   Vampyrellidae
## 218         Phyllopharyngea              Pseudochilodonopsis
## 6              Onygenaceae              Onygenaceae Family
## 8        Oligohymenophorea              Vorticella_ciliate
## 17           Centramoebida                   Centramoebida
## 20       Oligohymenophorea          Telotrochidium_ciliate
## 26          Spongomonadida                     Spongomonas
## 36    Pseudodactylogyridae            Pseudodactylogyridae
## 37           Fragilariales                         Ulnaria
## 54      Peronosporomycetes       Peronosporomycetes Family
## 60             Hypotrichia                         Meseres
## 62          Eugregarinorida                   Elev-18S-1089
## 70           Heterotrichea                     Spirostomum
## 74          Acanthocystidae                     Pterocystis
## 80  Bacillariophyceae Order         Bacillariophyceae Order
## 90       Oligohymenophorea               Oligohymenophorea
## 94    Rhizophydiales Order            Rhizophydiales Order
## 102   Blastocladiales Order           Blastocladiales Order
## 112              Euamoebida         BOLA868_Hartmannellidae
## 120           Echinamoebida                   Echinamoebida
## 123      Oligohymenophorea                     Opercularia
## 131  Platyhelminthes Phylum          Platyhelminthes Phylum
## 133           Incertae_Sedis                      Lecythium
## 144            Nucleariidae                       Nuclearia
## 147             Hypotrichia              Hypotrichia Family
## 162           Karyorelictea                         Loxodes
## 165      Oligohymenophorea                        Ophrydium
## 177            Chaetonotida                     Chaetonotus
## 185      Oligohymenophorea                      Ophryoglena
## 192         Liliopsida Order                 Liliopsida Order
## 196                Haptoria                        Didinium
## 209             Hypotrichia                    Paruroleptus
## 212  Trebouxiophyceae Class          Trebouxiophyceae Class
## 228             Alismatales                       Spirodela
## 234       Phaeosphaeriaceae                 Ophiosphaerella
## 237                Haptoria                      Amphileptus
## 242   Choreotrichia_ciliate Choreotrichia_ciliate Family
## 249           Vampyrellidae                        Penardia
## 254      Oligohymenophorea                       Paramecium
## 261             Prostomatea                          Coleps
## 265      Scotinosphaerales                  Scotinosphaera
## 273           Choreotrichia            Choreotrichia Family
## 279         Conthreep Order                 Conthreep Order
## 287          Annelida Phylum                 Annelida Phylum
## 294             Hypotrichia                        Halteria
## 300             Dorylaimida                     Dorylaimida
## 304         Cladosporiaceae                     Cladosporium
## 315                Haptoria                        Dileptus
## 321      Oligohymenophorea                  Histiobalantium
```

```
## 336             Armophorida                   Brachonella
## 340             Hypotrichia                   Hypotrichia
## 343                Haptoria                      Haptoria
## 366       Bacillariophyceae         Gomphonema_Gomphonema
## 372             Hypotrichia               Halteria_ciliate
## 383           Chromulinales                        JBNA46
## 401              Euglyphida                      Euglypha
## 407             Hypotrichia                Pseudouroleptus
## 410               Discosea                      Discosea
## 417            Plumatellida                  Plumatellida
## 424               Colpodea                   Platyophrya
## 429          Diatomea Phylum               Diatomea Phylum
## 435       Perkinsidae Class                  A31_alveolate
## 446               Adinetida                     Adinetida
## 456             Hypotrichia            Halteria_alveolate
## 459               Colpodea               Colpodea Family
## 471                Haptoria                      Phialina
## 477       Oligohymenophorea                   Dexiotricha
## 483       Dinophyceae Class             Dinophyceae Class
## 488   Dinoflagellata Phylum         Dinoflagellata Phylum
## 495              Armophorea                    Armophorea
## 508              Euglyphida             Euglyphida Family
## 513             Spongillida                   Spongillida
## 524                Haptoria                     Epiphyllum
## 536            Oligotrichia           Oligotrichia Family
## 553             Armophorida                   Armophorida
## 560       Aureobasidiaceae     Aureobasidiaceae Family
## 566         Eugregarinorida                     Syncystis
## 576             Salviniales                        Azolla
## 578       Capnodiales Order             Capnodiales Order
## 584     Labyrinthulomycetes    Sorodiplophrys_Eimeriidae
## 591             Plagiopylea            Plagiopyla_ciliate
## 602       Bacillariophyceae    Bacillariophyceae Family
## 611             Prostomatea                Prorodon_marine
## 616       Oligohymenophorea                   Peritrichia
## 621       Oligohymenophorea                     Epistylis
## 627                Haptoria               Haptoria Family
## 636       Oligohymenophorea              CV1-2A-17_ciliate
## 639           Heterotrichea                 Heterotrichea
## 645                Haptoria                    Hemiophrys
## 654       Embryophyta Class             Embryophyta Class
## 656          Chaetophorales         Chaetophorales Family
## 662                Haptoria                    Trachelius
## 672       Oligohymenophorea                     Lembadion
## 676       Oligohymenophorea                     Cothurnia
## 684       Oligohymenophorea                  Epicarchesium
## 696          Cryptomonadales                   Cryptomonas
## 702             Hypotrichia                     Uroleptus
## 703          Malasseziaceae                    Malassezia
## 713         Eukaryota Domain               Eukaryota Domain
## 719          Phyllopharyngea                     Tokophrya
## 726           Neodalyellida                 Neodalyellida
## 732               Asterales                    Helianthus
## 734       Oligohymenophorea                     Frontonia
```

```
## 741                Haptoria      Arcuospathidium_ciliate
## 747                Euplotia                   Aspidisca
## 760                Colpodea                    Bursaria
## 790       Bacillariophyceae                     Eunotia
## 798           Didymellaceae                    Boeremia
## 804      Peronosporomycetes                 Aphanomyces
## 805       Bacillariophyceae            Pleurosigma_marine
## 817            Oligotrichia                  Strombidium
## 825     Chrysophyceae Class         Chrysophyceae Class
## 830                Euplotia                    Euplotes
## 838         Phyllopharyngea       Phyllopharyngea Family
## 857              Rigifilida                  Rigifilida
## 869             Prostomatea                    Prorodon
## 875           Novel_Clade_12    Novel_Clade_12_freshwater
## 882           Didymellaceae                   Ascochyta
## 888             Hypotrichia                  Stylonychia
## 890             Hypotrichia             Neourostylopsis
```

```
chse_epistylis <- subset(chse, Genus == "Epistylis")
chse_epistylis
```

```
##                                  OTU Sample Abundance sample.ID LibraryName
## 621 b90aa4694de6f7ef3ba6cf52dc0df264   CHSE         0        NA          NA
##     Read_depth ProjectName Region Sample_date Year Site Sample_type Substrate
## 621   75084.67          NA     NA    20220602 2022   NA          NA        NA
##     Species Species_substrate Species_site Sorter Sex Carapace_length
## 621      NA                NA           NA     79  NA        249.3333
##     Plastron_length Mass Sample_number Sex_notes PCR1_date extraction_date
## 621             184 3725            72        NA      2022        20220624
##     Observed    Chao1  se.chao1     ACE   se.ACE   Shannon   Simpson InvSimpson
## 621 19.33333 19.66667 0.7711357 19.5257 1.989387 0.2881333 0.1419233   1.224196
##     log10_read_depth       PD    Domain     Phylum               Class     Order
## 621         4.874951 5.840054 Eukaryota Ciliophora Intramacronucleata Conthreep
##                Family     Genus
## 621 Oligohymenophorea Epistylis
```

```
chse_tokophyra <- subset(chse, Genus == "Tokophrya")
chse_tokophyra
```

```
##                                  OTU Sample Abundance sample.ID LibraryName
## 719 dbd7f00941ee192db4642e331b67ca9b   CHSE         0        NA          NA
##     Read_depth ProjectName Region Sample_date Year Site Sample_type Substrate
## 719   75084.67          NA     NA    20220602 2022   NA          NA        NA
##     Species Species_substrate Species_site Sorter Sex Carapace_length
## 719      NA                NA           NA     79  NA        249.3333
##     Plastron_length Mass Sample_number Sex_notes PCR1_date extraction_date
## 719             184 3725            72        NA      2022        20220624
##     Observed    Chao1  se.chao1     ACE   se.ACE   Shannon   Simpson InvSimpson
## 719 19.33333 19.66667 0.7711357 19.5257 1.989387 0.2881333 0.1419233   1.224196
##     log10_read_depth       PD    Domain     Phylum               Class     Order
## 719         4.874951 5.840054 Eukaryota Ciliophora Intramacronucleata Conthreep
##              Family     Genus
## 719 Phyllopharyngea Tokophrya
```

```r
chse_heliophyra <- subset(chse, Genus == "Heliophrya")
chse_heliophyra
```

```
##                                  OTU Sample    Abundance sample.ID LibraryName
## 752 e4279e681a284a86d4884a0f35a7e579   CHSE 0.0002929974        NA          NA
##     Read_depth ProjectName Region Sample_date Year Site Sample_type Substrate
## 752   75084.67          NA     NA    20220602 2022   NA          NA        NA
##     Species Species_substrate Species_site Sorter Sex Carapace_length
## 752      NA                NA           NA     79  NA        249.3333
##     Plastron_length Mass Sample_number Sex_notes PCR1_date extraction_date
## 752             184 3725            72        NA      2022        20220624
##     Observed    Chao1  se.chao1     ACE   se.ACE   Shannon   Simpson InvSimpson
## 752 19.33333 19.66667 0.7711357 19.5257 1.989387 0.2881333 0.1419233   1.224196
##     log10_read_depth       PD    Domain     Phylum              Class     Order
## 752         4.874951 5.840054 Eukaryota Ciliophora Intramacronucleata Conthreep
##               Family      Genus
## 752 Phyllopharyngea Heliophrya
```

```r
kisu <- subset(genus_merged, Sample == "KISU")
kisu_epistylis <- subset(kisu, Genus == "Epistylis")
kisu_epistylis
```

```
##                                  OTU Sample    Abundance sample.ID LibraryName
## 619 b90aa4694de6f7ef3ba6cf52dc0df264   KISU 3.995567e-05        NA          NA
##     Read_depth ProjectName Region Sample_date     Year Site Sample_type
## 619      83268          NA     NA    20217003 2021.643   NA          NA
##     Substrate Species Species_substrate Species_site Sorter Sex Carapace_length
## 619        NA      NA                NA           NA     NA  NA        83.32143
##     Plastron_length     Mass Sample_number Sex_notes PCR1_date extraction_date
## 619        73.84286 116.9286      27.71429        NA        NA        12999692
##     Observed    Chao1  se.chao1      ACE   se.ACE  Shannon   Simpson InvSimpson
## 619 49.64286 49.66667 0.1456949 49.75899 2.831405 1.464714 0.5577643   3.383409
##     log10_read_depth       PD    Domain     Phylum              Class     Order
## 619           4.9025 8.462877 Eukaryota Ciliophora Intramacronucleata Conthreep
##                Family     Genus
## 619 Oligohymenophorea Epistylis
```

```r
kisu_tokophyra <- subset(kisu, Genus == "Tokophrya")
kisu_tokophyra
```

```
##                                  OTU Sample Abundance sample.ID LibraryName
## 717 dbd7f00941ee192db4642e331b67ca9b   KISU         0        NA          NA
##     Read_depth ProjectName Region Sample_date     Year Site Sample_type
## 717      83268          NA     NA    20217003 2021.643   NA          NA
##     Substrate Species Species_substrate Species_site Sorter Sex Carapace_length
## 717        NA      NA                NA           NA     NA  NA        83.32143
##     Plastron_length     Mass Sample_number Sex_notes PCR1_date extraction_date
## 717        73.84286 116.9286      27.71429        NA        NA        12999692
##     Observed    Chao1  se.chao1      ACE   se.ACE  Shannon   Simpson InvSimpson
## 717 49.64286 49.66667 0.1456949 49.75899 2.831405 1.464714 0.5577643   3.383409
##     log10_read_depth       PD    Domain     Phylum              Class     Order
```

```
## 717           4.9025 8.462877 Eukaryota Ciliophora Intramacronucleata Conthreep
##               Family     Genus
## 717 Phyllopharyngea Tokophrya
```

```
kisu_heliophyra <- subset(kisu, Genus == "Heliophrya")
kisu_heliophyra
```

```
##                                  OTU Sample   Abundance sample.ID LibraryName
## 756 e4279e681a284a86d4884a0f35a7e579   KISU 0.003960183        NA          NA
##     Read_depth ProjectName Region Sample_date     Year Site Sample_type
## 756      83268          NA     NA    20217003 2021.643   NA          NA
##     Substrate Species Species_substrate Species_site Sorter Sex Carapace_length
## 756        NA      NA                NA           NA     NA  NA        83.32143
##     Plastron_length     Mass Sample_number Sex_notes PCR1_date extraction_date
## 756        73.84286 116.9286      27.71429        NA        NA        12999692
##     Observed    Chao1  se.chao1      ACE    se.ACE  Shannon   Simpson InvSimpson
## 756 49.64286 49.66667 0.1456949 49.75899 2.831405 1.464714 0.5577643   3.383409
##     log10_read_depth       PD    Domain     Phylum              Class     Order
## 756           4.9025 8.462877 Eukaryota Ciliophora Intramacronucleata Conthreep
##               Family      Genus
## 756 Phyllopharyngea Heliophrya
```

```
psco <- subset(genus_merged, Sample == "PSCO")
psco_epistylis <- subset(psco, Genus == "Epistylis")
psco_epistylis
```

```
##                                  OTU Sample  Abundance sample.ID LibraryName
## 620 b90aa4694de6f7ef3ba6cf52dc0df264   PSCO 0.01068831        NA          NA
##     Read_depth ProjectName Region Sample_date     Year Site Sample_type
## 620    73495.5          NA     NA    20218922 2021.833   NA          NA
##     Substrate Species Species_substrate Species_site Sorter Sex Carapace_length
## 620        NA      NA                NA           NA     54  NA        150.6417
##     Plastron_length     Mass Sample_number Sex_notes PCR1_date extraction_date
## 620        134.5583 604.3333      47.08333        NA        NA        16850856
##     Observed    Chao1  se.chao1      ACE    se.ACE Shannon   Simpson InvSimpson
## 620       61 61.33333 0.7136591 61.38026 3.282213 1.34775 0.5114167   2.628417
##     log10_read_depth       PD    Domain     Phylum              Class     Order
## 620         4.846451 10.04107 Eukaryota Ciliophora Intramacronucleata Conthreep
##                 Family     Genus
## 620 Oligohymenophorea Epistylis
```

```
psco_tokophyra <- subset(psco, Genus == "Tokophrya")
psco_tokophyra
```

```
##                                  OTU Sample   Abundance sample.ID LibraryName
## 715 dbd7f00941ee192db4642e331b67ca9b   PSCO 0.001099535        NA          NA
##     Read_depth ProjectName Region Sample_date     Year Site Sample_type
## 715    73495.5          NA     NA    20218922 2021.833   NA          NA
##     Substrate Species Species_substrate Species_site Sorter Sex Carapace_length
## 715        NA      NA                NA           NA     54  NA        150.6417
##     Plastron_length     Mass Sample_number Sex_notes PCR1_date extraction_date
## 715        134.5583 604.3333      47.08333        NA        NA        16850856
```

```
##     Observed    Chao1  se.chao1      ACE   se.ACE Shannon   Simpson InvSimpson
## 715       61 61.33333 0.7136591 61.38026 3.282213 1.34775 0.5114167   2.628417
##     log10_read_depth       PD    Domain     Phylum              Class     Order
## 715         4.846451 10.04107 Eukaryota Ciliophora Intramacronucleata Conthreep
##               Family     Genus
## 715 Phyllopharyngea Tokophrya
```

```r
psco_heliophyra <- subset(psco, Genus == "Heliophrya")
psco_heliophyra
```

```
##                                  OTU Sample  Abundance sample.ID LibraryName
## 754 e4279e681a284a86d4884a0f35a7e579   PSCO 0.00524215        NA          NA
##     Read_depth ProjectName Region Sample_date     Year Site Sample_type
## 754    73495.5          NA     NA    20218922 2021.833   NA          NA
##     Substrate Species Species_substrate Species_site Sorter Sex Carapace_length
## 754        NA      NA                NA           NA     54  NA        150.6417
##     Plastron_length     Mass Sample_number Sex_notes PCR1_date extraction_date
## 754        134.5583 604.3333      47.08333        NA        NA        16850856
##     Observed    Chao1  se.chao1      ACE   se.ACE Shannon   Simpson InvSimpson
## 754       61 61.33333 0.7136591 61.38026 3.282213 1.34775 0.5114167   2.628417
##     log10_read_depth       PD    Domain     Phylum              Class     Order
## 754         4.846451 10.04107 Eukaryota Ciliophora Intramacronucleata Conthreep
##               Family      Genus
## 754 Phyllopharyngea Heliophrya
```

```r
stca <- subset(genus_merged, Sample == "STCA")
stca_epistylis <- subset(stca, Genus == "Epistylis")
stca_epistylis
```

```
##                                  OTU Sample Abundance sample.ID LibraryName
## 624 b90aa4694de6f7ef3ba6cf52dc0df264   STCA 0.2431826        NA          NA
##     Read_depth ProjectName Region Sample_date Year Site Sample_type Substrate
## 624   73681.12          NA     NA    20220602 2022   NA          NA        NA
##     Species Species_substrate Species_site Sorter Sex Carapace_length
## 624      NA                NA           NA 44.375  NA           116.5
##     Plastron_length Mass Sample_number Sex_notes PCR1_date extraction_date
## 624         78.8125  229         34.25        NA      2022        20220622
##     Observed Chao1  se.chao1      ACE   se.ACE  Shannon Simpson InvSimpson
## 624   30.375  30.5 0.2909217 30.45549 2.205357 1.785125 0.68725   3.885051
##     log10_read_depth       PD    Domain     Phylum              Class     Order
## 624         4.847117 6.795299 Eukaryota Ciliophora Intramacronucleata Conthreep
##                 Family     Genus
## 624 Oligohymenophorea Epistylis
```

```r
stca_tokophyra <- subset(stca, Genus == "Tokophrya")
stca_tokophyra
```

```
##                                  OTU Sample   Abundance sample.ID LibraryName
## 720 dbd7f00941ee192db4642e331b67ca9b   STCA 0.002107471        NA          NA
##     Read_depth ProjectName Region Sample_date Year Site Sample_type Substrate
## 720   73681.12          NA     NA    20220602 2022   NA          NA        NA
##     Species Species_substrate Species_site Sorter Sex Carapace_length
```

```
## 720      NA                NA            NA 44.375  NA           116.5
##     Plastron_length Mass Sample_number Sex_notes PCR1_date extraction_date
## 720         78.8125  229         34.25        NA      2022        20220622
##     Observed Chao1  se.chao1      ACE   se.ACE  Shannon Simpson InvSimpson
## 720   30.375  30.5 0.2909217 30.45549 2.205357 1.785125 0.68725   3.885051
##     log10_read_depth       PD    Domain     Phylum              Class     Order
## 720         4.847117 6.795299 Eukaryota Ciliophora Intramacronucleata Conthreep
##              Family     Genus
## 720 Phyllopharyngea Tokophrya
```

```
stca_heliophyra <- subset(stca, Genus == "Heliophrya")
stca_heliophyra
```

```
##                                  OTU Sample Abundance sample.ID LibraryName
## 755 e4279e681a284a86d4884a0f35a7e579   STCA 0.2307659        NA          NA
##     Read_depth ProjectName Region Sample_date Year Site Sample_type Substrate
## 755   73681.12          NA     NA    20220602 2022   NA          NA        NA
##     Species Species_substrate Species_site Sorter Sex Carapace_length
## 755      NA                NA            NA 44.375  NA           116.5
##     Plastron_length Mass Sample_number Sex_notes PCR1_date extraction_date
## 755         78.8125  229         34.25        NA      2022        20220622
##     Observed Chao1  se.chao1      ACE   se.ACE  Shannon Simpson InvSimpson
## 755   30.375  30.5 0.2909217 30.45549 2.205357 1.785125 0.68725   3.885051
##     log10_read_depth       PD    Domain     Phylum              Class     Order
## 755         4.847117 6.795299 Eukaryota Ciliophora Intramacronucleata Conthreep
##              Family      Genus
## 755 Phyllopharyngea Heliophrya
```

```
stod <- subset(genus_merged, Sample == "STOD")
stod_epistylis <- subset(stod, Genus == "Epistylis")
stod_epistylis
```

```
##                                  OTU Sample Abundance sample.ID LibraryName
## 622 b90aa4694de6f7ef3ba6cf52dc0df264   STOD 0.2370674        NA          NA
##     Read_depth ProjectName Region Sample_date    Year Site Sample_type
## 622   102818.2          NA     NA    20216974 2021.64   NA          NA
##     Substrate Species Species_substrate Species_site Sorter Sex Carapace_length
## 622        NA      NA                NA           NA     NA  NA          83.828
##     Plastron_length   Mass Sample_number Sex_notes PCR1_date extraction_date
## 622          60.064 105.42         34.08        NA        NA        12941926
##     Observed Chao1  se.chao1      ACE   se.ACE Shannon Simpson InvSimpson
## 622     36.4 36.46 0.2088296 36.50671 2.440482 1.29672  0.5494   2.827085
##     log10_read_depth       PD    Domain     Phylum              Class     Order
## 622         5.001823 7.347801 Eukaryota Ciliophora Intramacronucleata Conthreep
##                Family     Genus
## 622 Oligohymenophorea Epistylis
```

```
stod_tokophyra <- subset(stod, Genus == "Tokophrya")
stod_tokophyra
```

```
##                                  OTU Sample   Abundance sample.ID LibraryName
## 718 dbd7f00941ee192db4642e331b67ca9b   STOD 0.003148123        NA          NA
```

```
##     Read_depth ProjectName Region Sample_date    Year Site Sample_type
## 718   102818.2          NA     NA    20216974 2021.64   NA          NA
##     Substrate Species Species_substrate Species_site Sorter Sex Carapace_length
## 718        NA      NA                NA           NA     NA  NA          83.828
##     Plastron_length   Mass Sample_number Sex_notes PCR1_date extraction_date
## 718          60.064 105.42         34.08        NA        NA        12941926
##     Observed Chao1  se.chao1      ACE   se.ACE Shannon Simpson InvSimpson
## 718     36.4 36.46 0.2088296 36.50671 2.440482 1.29672  0.5494   2.827085
##     log10_read_depth       PD    Domain     Phylum              Class     Order
## 718         5.001823 7.347801 Eukaryota Ciliophora Intramacronucleata Conthreep
##             Family    Genus
## 718 Phyllopharyngea Tokophrya
```

```r
stod_heliophyra <- subset(stod, Genus == "Heliophrya")
stod_heliophyra
```

```
##                                  OTU Sample  Abundance sample.ID LibraryName
## 753 e4279e681a284a86d4884a0f35a7e579   STOD 0.02214673        NA          NA
##     Read_depth ProjectName Region Sample_date    Year Site Sample_type
## 753   102818.2          NA     NA    20216974 2021.64   NA          NA
##     Substrate Species Species_substrate Species_site Sorter Sex Carapace_length
## 753        NA      NA                NA           NA     NA  NA          83.828
##     Plastron_length   Mass Sample_number Sex_notes PCR1_date extraction_date
## 753          60.064 105.42         34.08        NA        NA        12941926
##     Observed Chao1  se.chao1      ACE   se.ACE Shannon Simpson InvSimpson
## 753     36.4 36.46 0.2088296 36.50671 2.440482 1.29672  0.5494   2.827085
##     log10_read_depth       PD    Domain     Phylum              Class     Order
## 753         5.001823 7.347801 Eukaryota Ciliophora Intramacronucleata Conthreep
##             Family     Genus
## 753 Phyllopharyngea Heliophrya
```

```r
trsc <- subset(genus_merged, Sample == "TRSC")
trsc_epistylis <- subset(trsc, Genus == "Epistylis")
trsc_epistylis
```

```
##                                  OTU Sample  Abundance sample.ID LibraryName
## 623 b90aa4694de6f7ef3ba6cf52dc0df264   TRSC 0.03742871        NA          NA
##     Read_depth ProjectName Region Sample_date     Year Site Sample_type
## 623   87625.23          NA     NA    20217853 2021.727   NA          NA
##     Substrate Species Species_substrate Species_site Sorter Sex Carapace_length
## 623        NA      NA                NA           NA     NA  NA        169.5864
##     Plastron_length     Mass Sample_number Sex_notes PCR1_date extraction_date
## 623        154.6273 766.1364      24.54545        NA        NA        14706456
##     Observed    Chao1  se.chao1      ACE   se.ACE  Shannon   Simpson InvSimpson
## 623 76.54545 76.78788 0.5717776 76.75976 3.405874 1.987591 0.6591818   5.637309
##     log10_read_depth       PD    Domain     Phylum              Class     Order
## 623         4.934475 12.50263 Eukaryota Ciliophora Intramacronucleata Conthreep
##               Family     Genus
## 623 Oligohymenophorea Epistylis
```

```r
trsc_tokophyra <- subset(trsc, Genus == "Tokophrya")
trsc_tokophyra
```

```
##                                  OTU Sample   Abundance sample.ID LibraryName
## 716 dbd7f00941ee192db4642e331b67ca9b   TRSC 0.004610589        NA          NA
##     Read_depth ProjectName Region Sample_date     Year Site Sample_type
## 716   87625.23          NA     NA    20217853 2021.727   NA          NA
##     Substrate Species Species_substrate Species_site Sorter Sex Carapace_length
## 716        NA      NA                NA           NA     NA  NA        169.5864
##     Plastron_length     Mass Sample_number Sex_notes PCR1_date extraction_date
## 716        154.6273 766.1364      24.54545        NA        NA        14706456
##     Observed    Chao1  se.chao1      ACE   se.ACE  Shannon   Simpson InvSimpson
## 716 76.54545 76.78788 0.5717776 76.75976 3.405874 1.987591 0.6591818   5.637309
##     log10_read_depth       PD    Domain     Phylum               Class     Order
## 716         4.934475 12.50263 Eukaryota Ciliophora Intramacronucleata Conthreep
##              Family     Genus
## 716 Phyllopharyngea Tokophrya
```

```
trsc_heliophyra <- subset(trsc, Genus == "Heliophrya")
trsc_heliophyra
```

```
##                                  OTU Sample Abundance sample.ID LibraryName
## 751 e4279e681a284a86d4884a0f35a7e579   TRSC 0.0109697        NA          NA
##     Read_depth ProjectName Region Sample_date     Year Site Sample_type
## 751   87625.23          NA     NA    20217853 2021.727   NA          NA
##     Substrate Species Species_substrate Species_site Sorter Sex Carapace_length
## 751        NA      NA                NA           NA     NA  NA        169.5864
##     Plastron_length     Mass Sample_number Sex_notes PCR1_date extraction_date
## 751        154.6273 766.1364      24.54545        NA        NA        14706456
##     Observed    Chao1  se.chao1      ACE   se.ACE  Shannon   Simpson InvSimpson
## 751 76.54545 76.78788 0.5717776 76.75976 3.405874 1.987591 0.6591818   5.637309
##     log10_read_depth       PD    Domain     Phylum               Class     Order
## 751         4.934475 12.50263 Eukaryota Ciliophora Intramacronucleata Conthreep
##              Family      Genus
## 751 Phyllopharyngea Heliophrya
```

Statistical testing for alpha diversity significance

ANOVA Testing is done first for Shannon diversity with all carapace samples and then again after removing standardized residual outliers. Factors tested are turtle species, collection site, turtle sex and (log10) read depth. This is repeated for (log10) observed features and Faith's Phylogenetic Diversity.

Turtle species and plastron length showed consistently high to moderate levels of collinearity. Because of this, plastron length was not used as a factor, but was subsequently checked for any effects within individual species (see next section).

Q-Q and residual plots, among other diagnostics, are used to assess assumptions of normality. P-values are Tukey-adjusted for pairwise comparisons in ANOVA analyses.

```
#Testing for Shannon diversity, all samples included
#NOTE: Reordering the factors in the below model does not change the resulting p-values
pl.shannon.lm <- lm(Shannon~Species+Site+Plastron_length+Sex+log10_read_depth+Year,data=carapace)
#NOTE: running the above model, both Species and Plastron_length have high or moderate levels of collin
#Because of this, Plastron_length is taken out of the model and is checked later for any effects within
pl.shannon.lm <- lm(Shannon~Species+Site+Sex+log10_read_depth+Year,data=carapace)

summary(pl.shannon.lm)
```

```
##
## Call:
## lm(formula = Shannon ~ Species + Site + Sex + log10_read_depth +
##     Year, data = carapace)
##
## Residuals:
##     Min      1Q  Median      3Q     Max
## -1.3662 -0.3914  0.1005  0.3827  1.1336
##
## Coefficients:
##                   Estimate Std. Error t value Pr(>|t|)
## (Intercept)      1223.6527   346.2485   3.534 0.000719 ***
## SpeciesKISU         1.2152     0.3927   3.095 0.002806 **
## SpeciesPSCO         0.8026     0.4178   1.921 0.058687 .
## SpeciesSTCA         0.5044     0.4805   1.050 0.297352
## SpeciesSTOD         0.7959     0.3810   2.089 0.040271 *
## SpeciesTRSC         1.3003     0.3823   3.401 0.001099 **
## SiteS1              0.9695     0.2588   3.747 0.000358 ***
## SiteS4             -0.4909     0.1735  -2.830 0.006020 **
## SexJ                0.5162     0.2932   1.761 0.082551 .
## SexM                0.3095     0.1491   2.076 0.041509 *
## log10_read_depth    1.4881     0.6793   2.191 0.031724 *
## Year               -0.6087     0.1707  -3.566 0.000649 ***
## ---
## Signif. codes:  0 '***' 0.001 '**' 0.01 '*' 0.05 '.' 0.1 ' ' 1
##
## Residual standard error: 0.5955 on 72 degrees of freedom
## Multiple R-squared:  0.5382, Adjusted R-squared:  0.4676
## F-statistic: 7.628 on 11 and 72 DF,  p-value: 1.526e-08
```

```
confint(pl.shannon.lm)
```

```
##                         2.5 %       97.5 %
## (Intercept)      533.41898877 1913.8864832
## SpeciesKISU        0.43241752    1.9980691
## SpeciesPSCO       -0.03026085    1.6355291
## SpeciesSTCA       -0.45347690    1.4622986
## SpeciesSTOD        0.03628809    1.5554498
## SpeciesTRSC        0.53810944    2.0624123
## SiteS1             0.45368312    1.4853576
## SiteS4            -0.83671035   -0.1451770
## SexJ              -0.06826918    1.1006477
## SexM               0.01224046    0.6067761
## log10_read_depth   0.13387689    2.8423235
## Year              -0.94902571   -0.2684079
```

```
check_model(pl.shannon.lm)
```

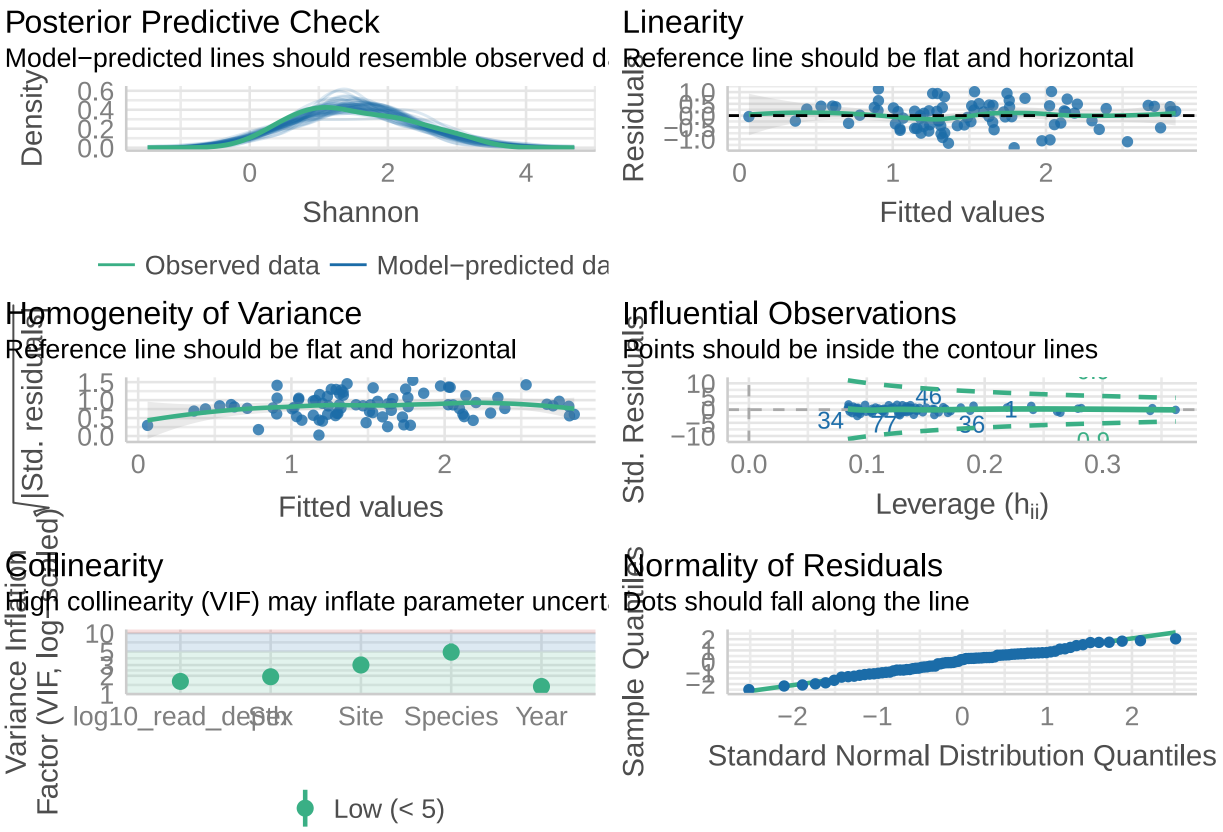


```r
plot(pl.shannon.lm, which = 1)
```

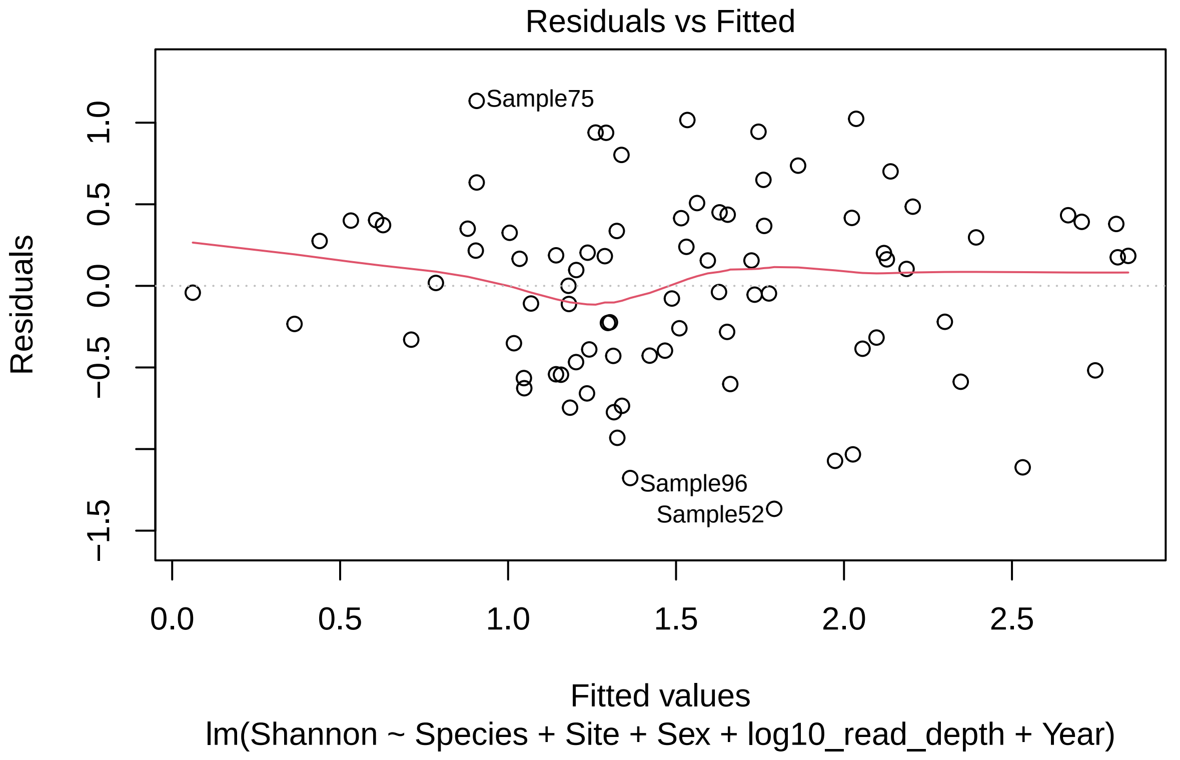

```r
plot(pl.shannon.lm, which = 2)
```

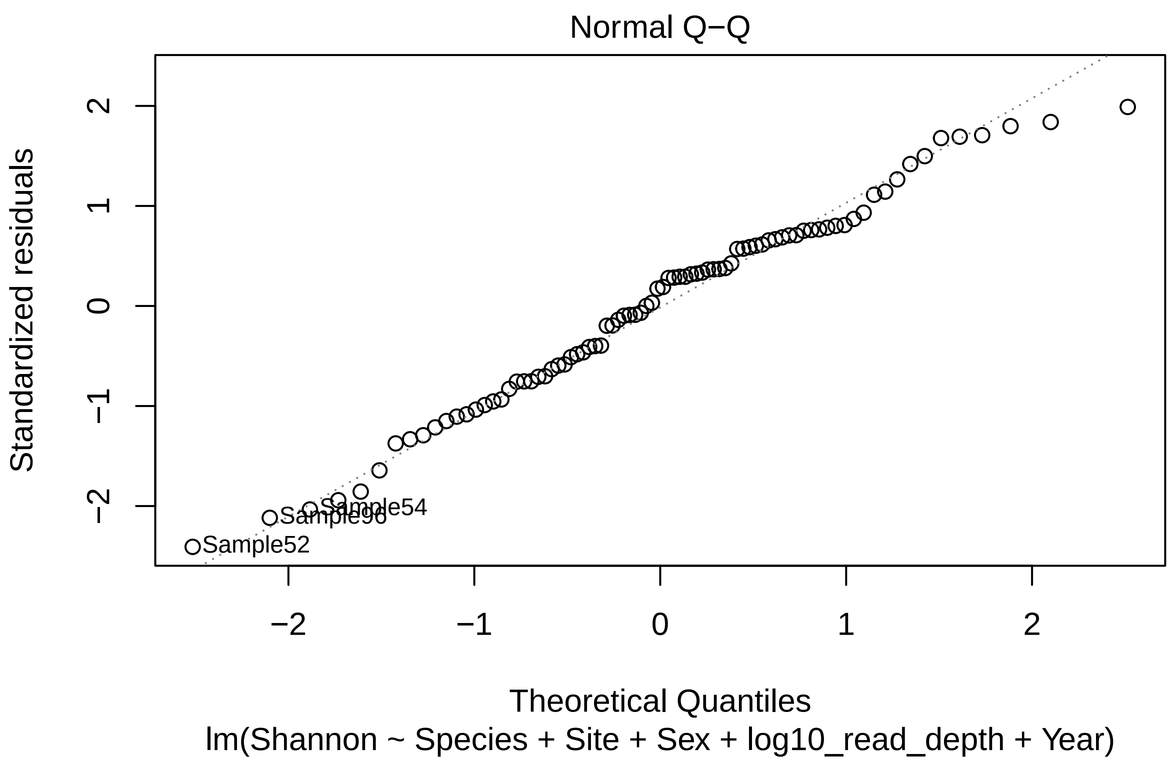


```r
ols_plot_resid_stand(pl.shannon.lm)
```

Table 7: Result: ANOVA comparing Shannon diversity of turtle species by different factors

	Sum Sq	Df	F value	Pr(>F)
Species	8.268924	5	4.664143	0.0009598
Site	10.930988	2	15.414244	0.0000027
Sex	1.978578	2	2.790075	0.0680687
log10_read_depth	1.701404	1	4.798444	0.0317242
Year	4.508247	1	12.714536	0.0006494
Residuals	25.529346	72	NA	NA

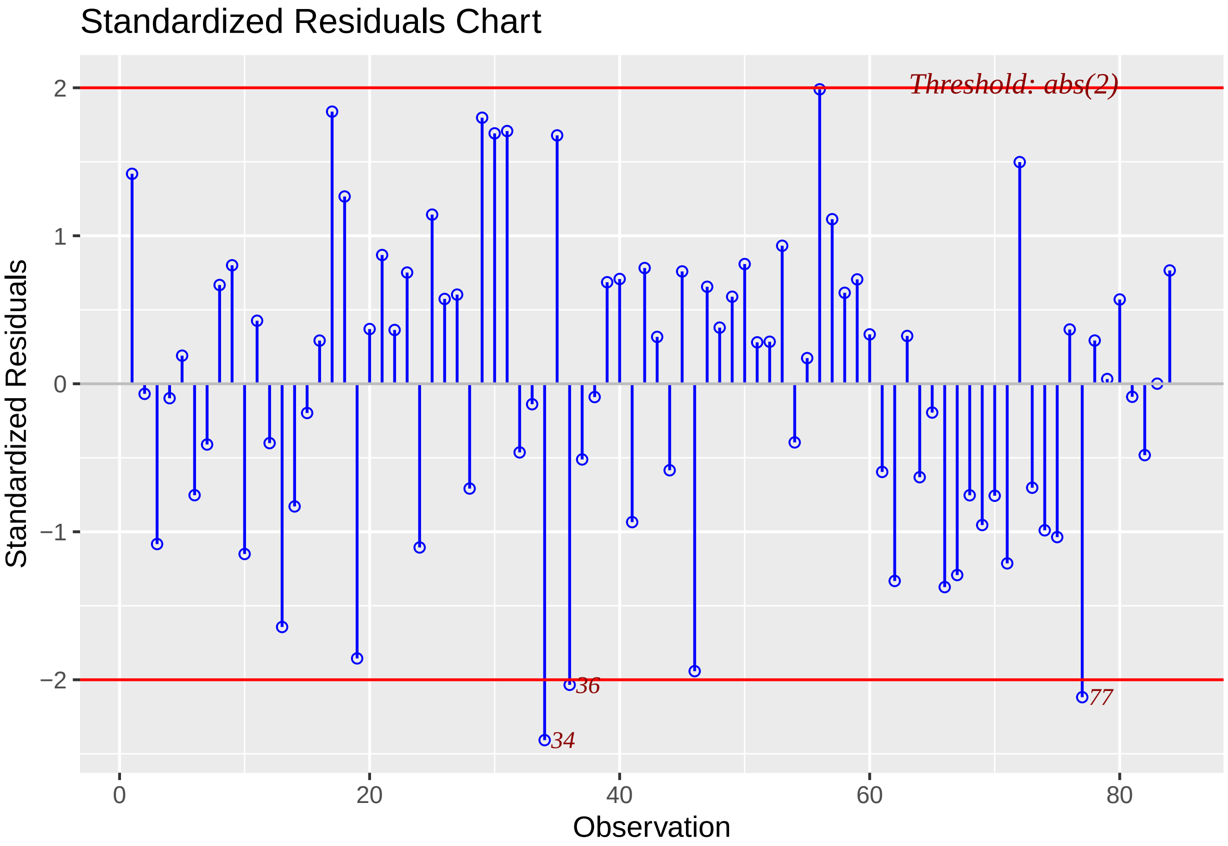


```
#ANOVA with pairwise comparisons (Tukey adjustment for pairwise comparisons):
kable.wrap(Anova(pl.shannon.lm),"Result: ANOVA comparing Shannon diversity of turtle species by differe
```

```
emmeans(pl.shannon.lm, list(pairwise ~ Species), adjust = "tukey")
```

```
## $`emmeans of Species`
##  Species emmean    SE df lower.CL upper.CL
##  CHSE     0.897 0.386 72    0.127     1.67
##  KISU     2.112 0.205 72    1.705     2.52
```

```
##  PSCO     1.700 0.227 72    1.247     2.15
##  STCA     1.402 0.280 72    0.843     1.96
##  STOD     1.693 0.172 72    1.349     2.04
##  TRSC     2.197 0.158 72    1.883     2.51
##
## Results are averaged over the levels of: Site, Sex, Year
## Confidence level used: 0.95
##
## $`pairwise differences of Species`
##  1           estimate    SE df t.ratio p.value
##  CHSE - KISU -1.21524 0.393 72  -3.095  0.0322
##  CHSE - PSCO -0.80263 0.418 72  -1.921  0.3980
##  CHSE - STCA -0.50441 0.481 72  -1.050  0.8992
##  CHSE - STOD -0.79587 0.381 72  -2.089  0.3048
##  CHSE - TRSC -1.30026 0.382 72  -3.401  0.0135
##  KISU - PSCO  0.41261 0.295 72   1.396  0.7290
##  KISU - STCA  0.71083 0.359 72   1.981  0.3634
##  KISU - STOD  0.41937 0.210 72   1.993  0.3564
##  KISU - TRSC -0.08502 0.218 72  -0.390  0.9988
##  PSCO - STCA  0.29822 0.396 72   0.753  0.9744
##  PSCO - STOD  0.00677 0.281 72   0.024  1.0000
##  PSCO - TRSC -0.49763 0.276 72  -1.802  0.4709
##  STCA - STOD -0.29146 0.341 72  -0.856  0.9556
##  STCA - TRSC -0.79585 0.306 72  -2.600  0.1106
##  STOD - TRSC -0.50439 0.186 72  -2.717  0.0843
##
## Results are averaged over the levels of: Site, Sex, Year
## P value adjustment: tukey method for comparing a family of 6 estimates
```

```
emmeans(pl.shannon.lm, list(pairwise ~ Site), adjust = "tukey")
```

```
## $`emmeans of Site`
##  Site emmean    SE df lower.CL upper.CL
##  BP4    1.51 0.127 72    1.255     1.76
##  S1     2.48 0.235 72    2.008     2.95
##  S4     1.02 0.172 72    0.673     1.36
##
## Results are averaged over the levels of: Species, Sex, Year
## Confidence level used: 0.95
##
## $`pairwise differences of Site`
##  1        estimate    SE df t.ratio p.value
##  BP4 - S1   -0.970 0.259 72  -3.747  0.0010
##  BP4 - S4    0.491 0.173 72   2.830  0.0164
##  S1 - S4     1.460 0.266 72   5.484  <.0001
##
## Results are averaged over the levels of: Species, Sex, Year
## P value adjustment: tukey method for comparing a family of 3 estimates
```

```
#Rerun above analysis, but with standardized residual outliers removed
carapace_shannon_no_outliers<-carapace[-c(6,25,34,36,75),]
summary(carapace_shannon_no_outliers)
```

```
##   sample.ID         LibraryName          Read_depth     ProjectName
```

```
##  Length:79          Length:79          Min.   : 43496   Length:79
##  Class :character   Class :character   1st Qu.: 71552   Class :character
##  Mode  :character   Mode  :character   Median : 89516   Mode  :character
##                                        Mean   : 88071
##                                        3rd Qu.:103597
##                                        Max.   :174528
##     Region          Sample_date             Year          Site
##  Length:79          Length:79          Min.   :2021   Length:79
##  Class :character   Class :character   1st Qu.:2021   Class :character
##  Mode  :character   Mode  :character   Median :2022   Mode  :character
##                                        Mean   :2022
##                                        3rd Qu.:2022
##                                        Max.   :2022
##  Sample_type         Substrate           Species          Species_substrate
##  Length:79          Length:79          Length:79          Length:79
##  Class :character   Class :character   Class :character   Class :character
##  Mode  :character   Mode  :character   Mode  :character   Mode  :character
##
##
##
##  Species_site          Sorter              Sex            Carapace_length
##  Length:79          Length:79          Length:79          Min.   : 49.4
##  Class :character   Class :character   Class :character   1st Qu.: 86.9
##  Mode  :character   Mode  :character   Mode  :character   Median : 94.8
##                                                           Mean   :120.7
##                                                           3rd Qu.:154.4
##                                                           Max.   :308.0
##  Plastron_length       Mass      Sample_number       Sex_notes
##  Min.   : 35.80   Min.   :  25   Length:79          Length:79
##  1st Qu.: 64.50   1st Qu.: 110   Class :character   Class :character
##  Median : 80.00   Median : 145   Mode  :character   Mode  :character
##  Mean   : 99.93   Mean   : 466
##  3rd Qu.:142.40   3rd Qu.: 535
##  Max.   :239.00   Max.   :6600
##   PCR1_date         extraction_date       Observed          Chao1
##  Length:79          Min.   :    2021   Min.   :  9.00   Min.   :  9.00
##  Class :character   1st Qu.:    2021   1st Qu.: 28.00   1st Qu.: 28.00
##  Mode  :character   Median :20220623   Median : 44.00   Median : 44.00
##                     Mean   :14846057   Mean   : 49.95   Mean   : 50.10
##                     3rd Qu.:20220623   3rd Qu.: 70.00   3rd Qu.: 70.25
##                     Max.   :20220624   Max.   :130.00   Max.   :130.00
##     se.chao1           ACE             se.ACE          Shannon
##  Min.   :0.0000   Min.   :  9.00   Min.   :0.9608   Min.   :0.0194
##  1st Qu.:0.0000   1st Qu.: 28.00   1st Qu.:2.2969   1st Qu.:0.9460
##  Median :0.0000   Median : 44.20   Median :2.7386   Median :1.4100
##  Mean   :0.3842   Mean   : 50.12   Mean   :2.7860   Mean   :1.5388
##  3rd Qu.:0.3672   3rd Qu.: 70.26   3rd Qu.:3.3331   3rd Qu.:2.1350
##  Max.   :4.6460   Max.   :130.28   Max.   :4.4630   Max.   :3.1900
##     Simpson          InvSimpson     log10_read_depth       PD
##  Min.   :0.00427   Min.   : 1.004   Min.   :4.638    Min.   : 2.675
##  1st Qu.:0.42150   1st Qu.: 1.730   1st Qu.:4.855    1st Qu.: 6.528
##  Median :0.64300   Median : 2.804   Median :4.952    Median : 8.276
##  Mean   :0.58016   Mean   : 3.742   Mean   :4.928    Mean   : 8.938
##  3rd Qu.:0.78850   3rd Qu.: 4.736   3rd Qu.:5.015    3rd Qu.:10.991
```

```
##  Max.   :0.92900   Max.   :13.991   Max.   :5.242    Max.   :19.537
```

```
pl.shannon_no_outliers.lm <- lm(Shannon~Species+Site+Sex+log10_read_depth+Year,data=carapace_shannon_no_
summary(pl.shannon_no_outliers.lm)
```

```
##
## Call:
## lm(formula = Shannon ~ Species + Site + Sex + log10_read_depth +
##     Year, data = carapace_shannon_no_outliers)
##
## Residuals:
##      Min       1Q   Median       3Q      Max
## -1.22323 -0.33795  0.09298  0.34975  1.13278
##
## Coefficients:
##                   Estimate Std. Error t value Pr(>|t|)
## (Intercept)      1295.5453   338.7456   3.825 0.000290 ***
## SpeciesKISU         1.2327     0.3699   3.332 0.001405 **
## SpeciesPSCO         1.0362     0.4226   2.452 0.016809 *
## SpeciesSTCA         0.4009     0.4628   0.866 0.389368
## SpeciesSTOD         0.8172     0.3592   2.275 0.026090 *
## SpeciesTRSC         1.3904     0.3660   3.799 0.000316 ***
## SiteS1              1.0584     0.2627   4.029 0.000146 ***
## SiteS4             -0.5274     0.1672  -3.155 0.002407 **
## SexJ                0.3540     0.2995   1.182 0.241309
## SexM                0.3744     0.1434   2.611 0.011141 *
## log10_read_depth    1.4478     0.6637   2.181 0.032678 *
## Year               -0.6442     0.1671  -3.856 0.000262 ***
## ---
## Signif. codes:  0 '***' 0.001 '**' 0.01 '*' 0.05 '.' 0.1 ' ' 1
##
## Residual standard error: 0.5595 on 67 degrees of freedom
## Multiple R-squared:  0.5978, Adjusted R-squared:  0.5318
## F-statistic: 9.054 on 11 and 67 DF,  p-value: 1.198e-09
```

```
confint(pl.shannon_no_outliers.lm)
```

```
##                          2.5 %       97.5 %
## (Intercept)      619.40631338 1971.6842605
## SpeciesKISU        0.49434707    1.9711207
## SpeciesPSCO        0.19278008    1.8796655
## SpeciesSTCA       -0.52273281    1.3245802
## SpeciesSTOD        0.10032388    1.5340986
## SpeciesTRSC        0.65985382    2.1208942
## SiteS1             0.53403629    1.5827146
## SiteS4            -0.86115392   -0.1937053
## SexJ              -0.24370557    0.9517399
## SexM               0.08813276    0.6605709
## log10_read_depth   0.12295590    2.7725499
## Year              -0.97769451   -0.3106976
```

```
check_model(pl.shannon_no_outliers.lm)
```

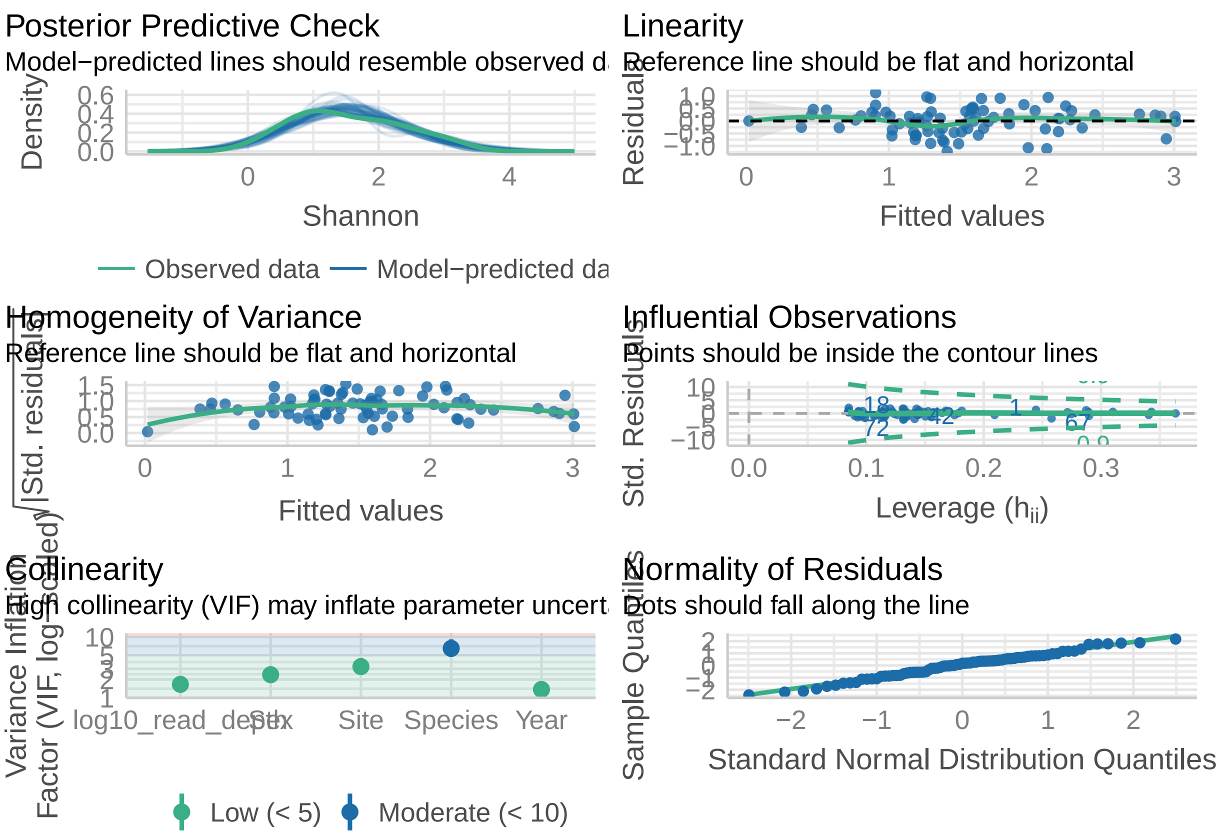


```
plot(pl.shannon_no_outliers.lm, which = 1)
```

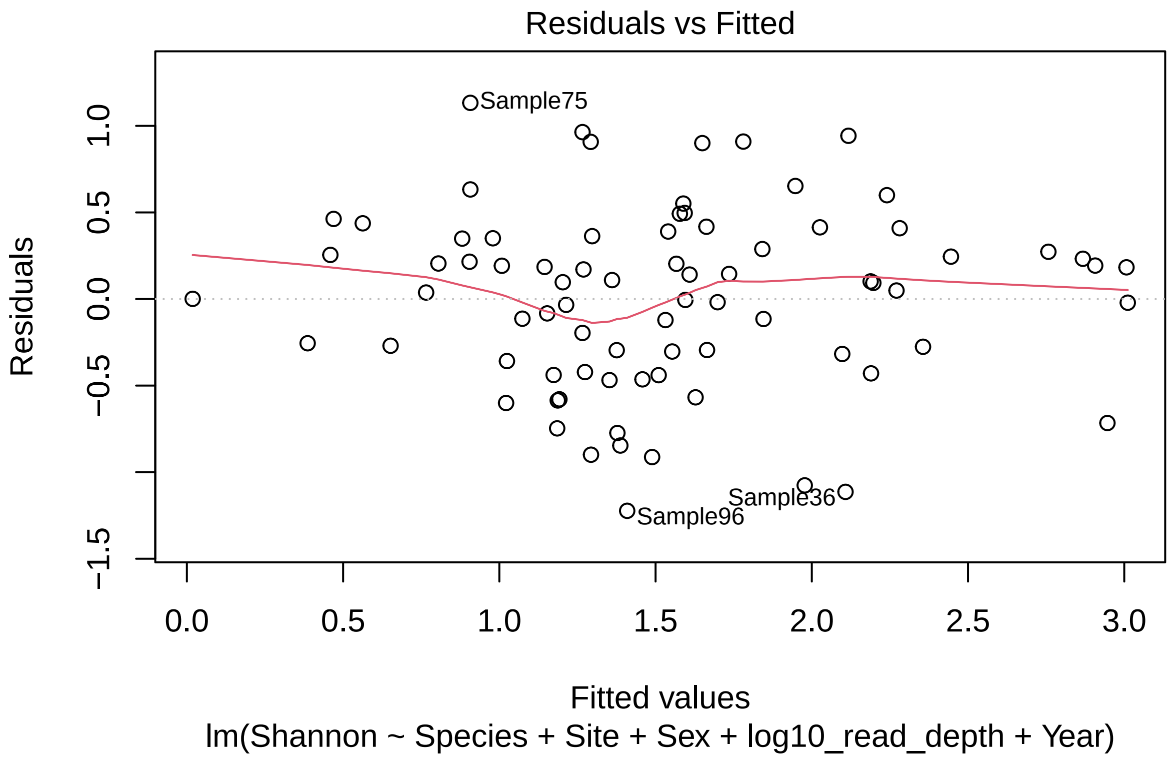


```r
plot(pl.shannon_no_outliers.lm, which = 2)
```

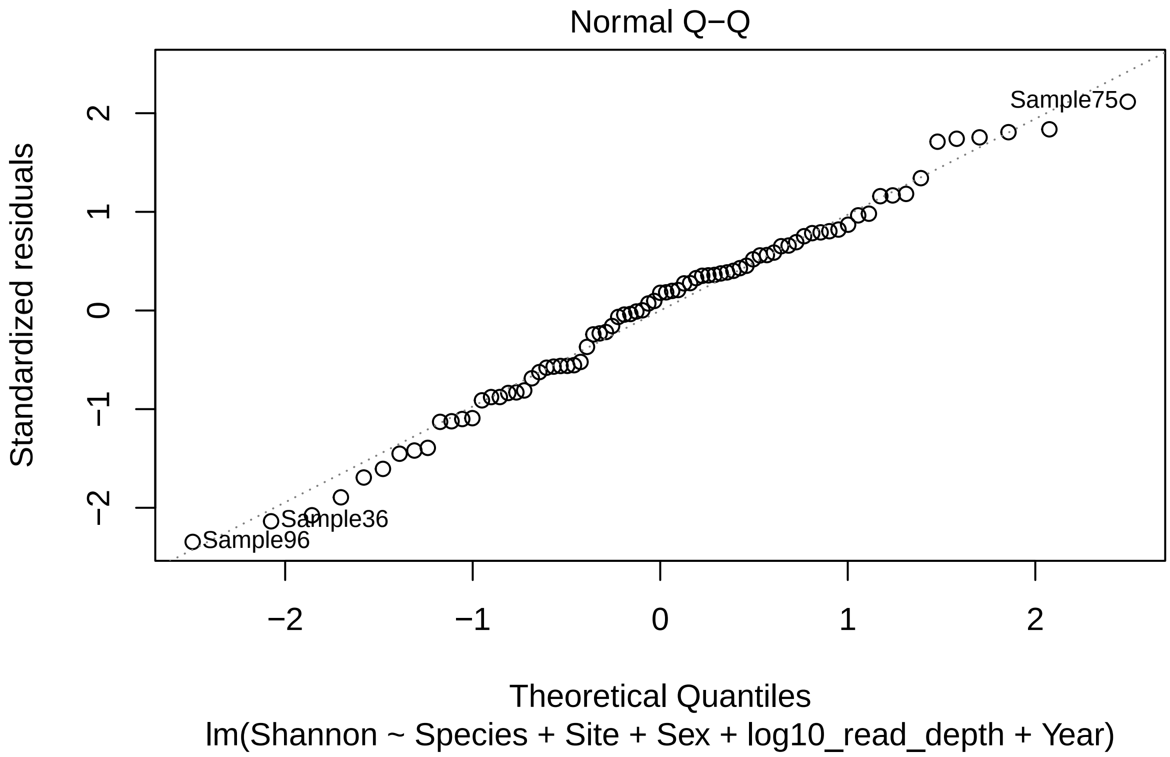


```r
#ANOVA with pairwise comparisons (Tukey adjustment for pairwise comparisons):
kable.wrap(Anova(pl.shannon_no_outliers.lm),"Result: ANOVA comparing Shannon diversity of turtle specie
```

Table 8: Result: ANOVA comparing Shannon diversity of turtle species by different factors, standardized residual outliers removed

	Sum Sq	Df	F value	Pr(>F)
Species	9.417897	5	6.016352	0.0001175
Site	12.045798	2	19.237776	0.0000002
Sex	2.235037	2	3.569472	0.0336474
log10_read_depth	1.489586	1	4.757896	0.0326779
Year	4.653974	1	14.865285	0.0002615
Residuals	20.976138	67	NA	NA

```
emmeans(pl.shannon_no_outliers.lm, list(pairwise ~ Species), adjust = "tukey")
```

```
## $`emmeans of Species`
##  Species emmean    SE df lower.CL upper.CL
##  CHSE     0.857 0.369 67    0.121     1.59
##  KISU     2.090 0.198 67    1.695     2.49
##  PSCO     1.894 0.249 67    1.396     2.39
##  STCA     1.258 0.271 67    0.717     1.80
##  STOD     1.675 0.170 67    1.336     2.01
##  TRSC     2.248 0.158 67    1.933     2.56
##
## Results are averaged over the levels of: Site, Sex, Year
## Confidence level used: 0.95
##
## $`pairwise differences of Species`
##  1            estimate    SE df t.ratio p.value
##  CHSE - KISU   -1.233 0.370 67  -3.332  0.0169
##  CHSE - PSCO   -1.036 0.423 67  -2.452  0.1536
##  CHSE - STCA   -0.401 0.463 67  -0.866  0.9532
##  CHSE - STOD   -0.817 0.359 67  -2.275  0.2187
##  CHSE - TRSC   -1.390 0.366 67  -3.799  0.0041
##  KISU - PSCO    0.197 0.319 67   0.615  0.9895
##  KISU - STCA    0.832 0.348 67   2.392  0.1738
##  KISU - STOD    0.416 0.198 67   2.098  0.3010
##  KISU - TRSC   -0.158 0.210 67  -0.752  0.9744
##  PSCO - STCA    0.635 0.412 67   1.543  0.6381
##  PSCO - STOD    0.219 0.309 67   0.708  0.9803
##  PSCO - TRSC   -0.354 0.309 67  -1.148  0.8593
##  STCA - STOD   -0.416 0.333 67  -1.251  0.8100
##  STCA - TRSC   -0.989 0.301 67  -3.283  0.0195
##  STOD - TRSC   -0.573 0.180 67  -3.176  0.0263
##
## Results are averaged over the levels of: Site, Sex, Year
## P value adjustment: tukey method for comparing a family of 6 estimates
```

```r
emmeans(pl.shannon_no_outliers.lm, list(pairwise ~ Site), adjust = "tukey")
```

```
## $`emmeans of Site`
##  Site emmean    SE df lower.CL upper.CL
##  BP4    1.493 0.122 67     1.25     1.74
##  S1     2.552 0.234 67     2.08     3.02
##  S4     0.966 0.163 67     0.64     1.29
##
## Results are averaged over the levels of: Species, Sex, Year
## Confidence level used: 0.95
##
## $`pairwise differences of Site`
##  1        estimate    SE df t.ratio p.value
##  BP4 - S1   -1.058 0.263 67  -4.029  0.0004
##  BP4 - S4    0.527 0.167 67   3.155  0.0067
##  S1 - S4     1.586 0.262 67   6.060  <.0001
##
## Results are averaged over the levels of: Species, Sex, Year
## P value adjustment: tukey method for comparing a family of 3 estimates
```

```r
#Testing for observed features, all samples included
#NOTE: Reordering the factors in the below model does not change the resulting p-values
#also, log10 of observed features counts does seem to improve the model fit
pl.observed.lm <- lm(log10(Observed)~Species+Site+Sex+log10_read_depth+Year,data=carapace)

summary(pl.observed.lm)
```

```
##
## Call:
## lm(formula = log10(Observed) ~ Species + Site + Sex + log10_read_depth +
##     Year, data = carapace)
##
## Residuals:
##      Min       1Q   Median       3Q      Max
## -0.46305 -0.07738 -0.00522  0.11348  0.48858
##
## Coefficients:
##                   Estimate Std. Error t value Pr(>|t|)
## (Intercept)      332.70970  105.98796   3.139 0.002457 **
## SpeciesKISU        0.26418    0.12021   2.198 0.031186 *
## SpeciesPSCO        0.45703    0.12789   3.574 0.000633 ***
## SpeciesSTCA       -0.13817    0.14709  -0.939 0.350663
## SpeciesSTOD        0.01536    0.11664   0.132 0.895570
## SpeciesTRSC        0.39099    0.11703   3.341 0.001326 **
## SiteS1             0.31911    0.07921   4.029 0.000137 ***
## SiteS4             0.04928    0.05309   0.928 0.356403
## SexJ               0.07622    0.08975   0.849 0.398543
## SexM               0.06951    0.04565   1.523 0.132197
## log10_read_depth   0.82742    0.20795   3.979 0.000163 ***
## Year              -0.16594    0.05226  -3.175 0.002202 **
## ---
## Signif. codes:  0 '***' 0.001 '**' 0.01 '*' 0.05 '.' 0.1 ' ' 1
```

```
##
## Residual standard error: 0.1823 on 72 degrees of freedom
## Multiple R-squared:  0.6429, Adjusted R-squared:  0.5883
## F-statistic: 11.78 on 11 and 72 DF,  p-value: 3.125e-12
```

```
confint(pl.observed.lm)
```

```
##                         2.5 %       97.5 %
## (Intercept)      121.42656648 543.99283594
## SpeciesKISU        0.02455281   0.50380461
## SpeciesPSCO        0.20208121   0.71198574
## SpeciesSTCA       -0.43138780   0.15503827
## SpeciesSTOD       -0.21714676   0.24787434
## SpeciesTRSC        0.15768786   0.62428268
## SiteS1             0.16121165   0.47701107
## SiteS4            -0.05655863   0.15512231
## SexJ              -0.10268600   0.25512385
## SexM              -0.02148570   0.16050390
## log10_read_depth   0.41289081   1.24195647
## Year              -0.27010684  -0.06176717
```

```
check_model(pl.observed.lm)
```

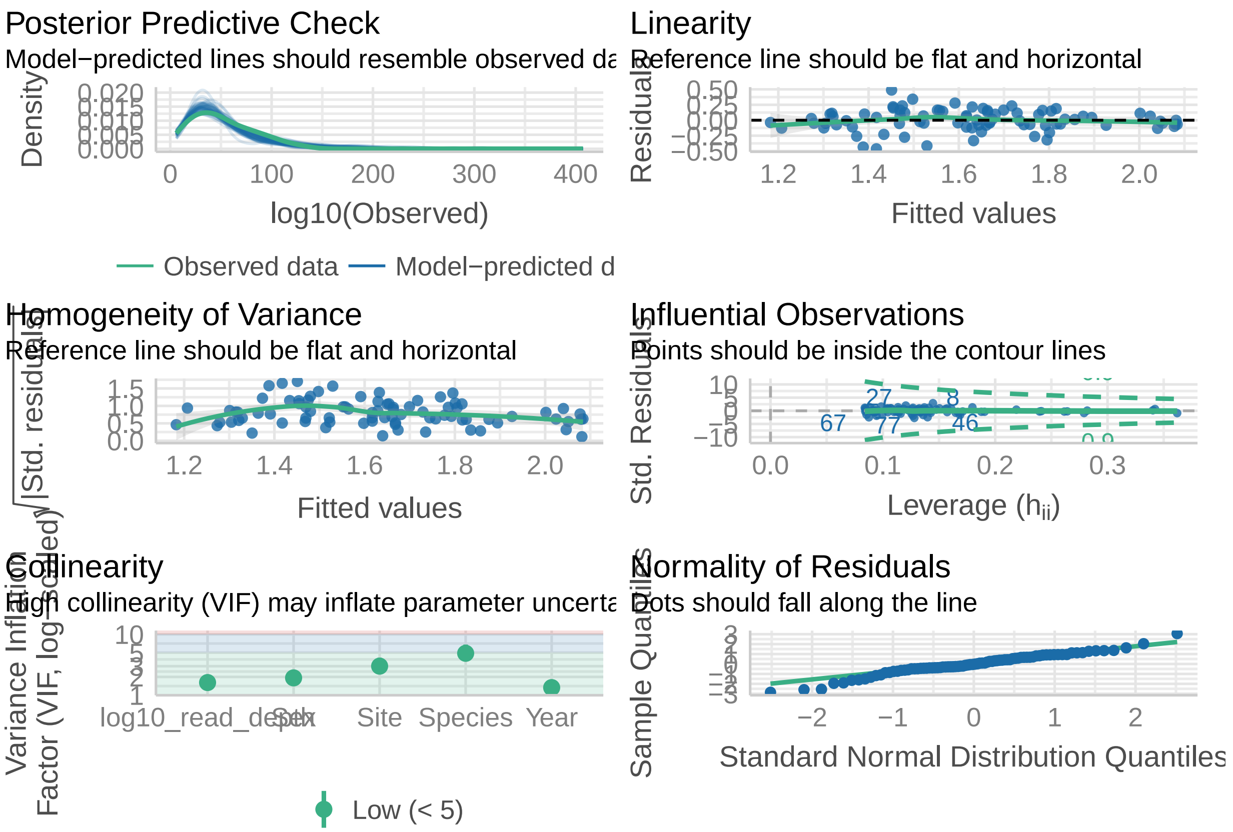


```
plot(pl.observed.lm, which = 1)
```

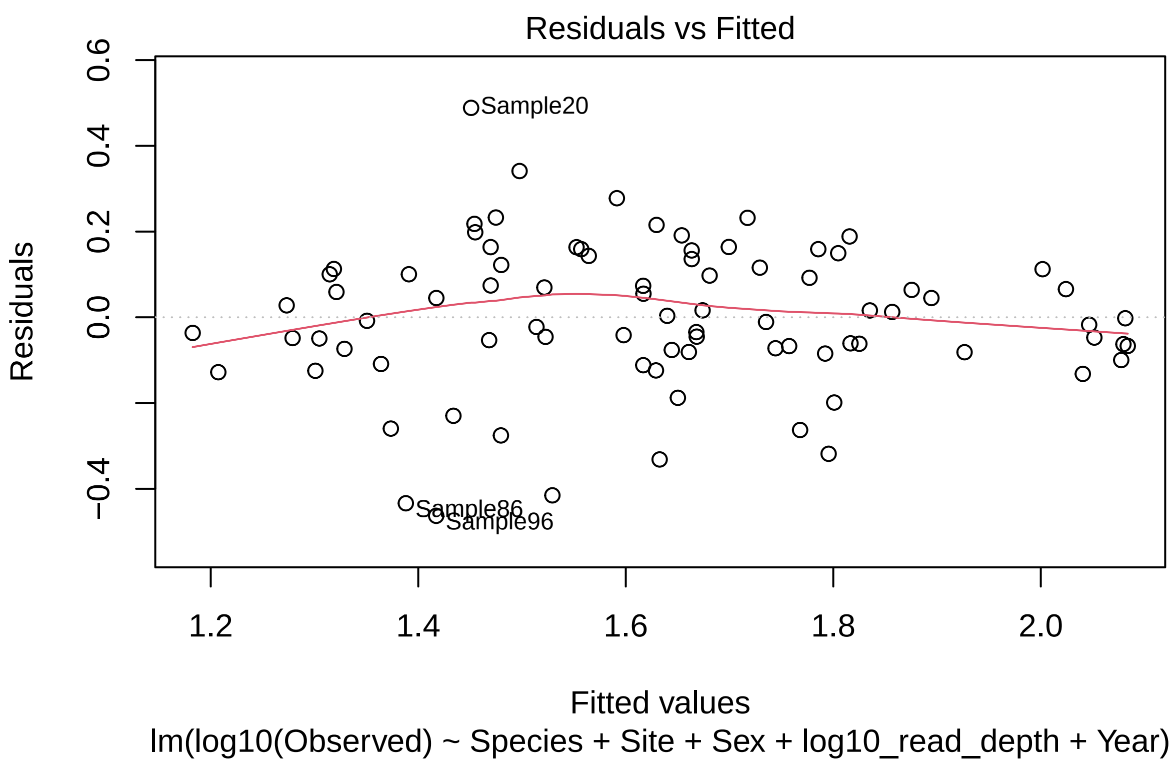


```
plot(pl.observed.lm, which = 2)
```

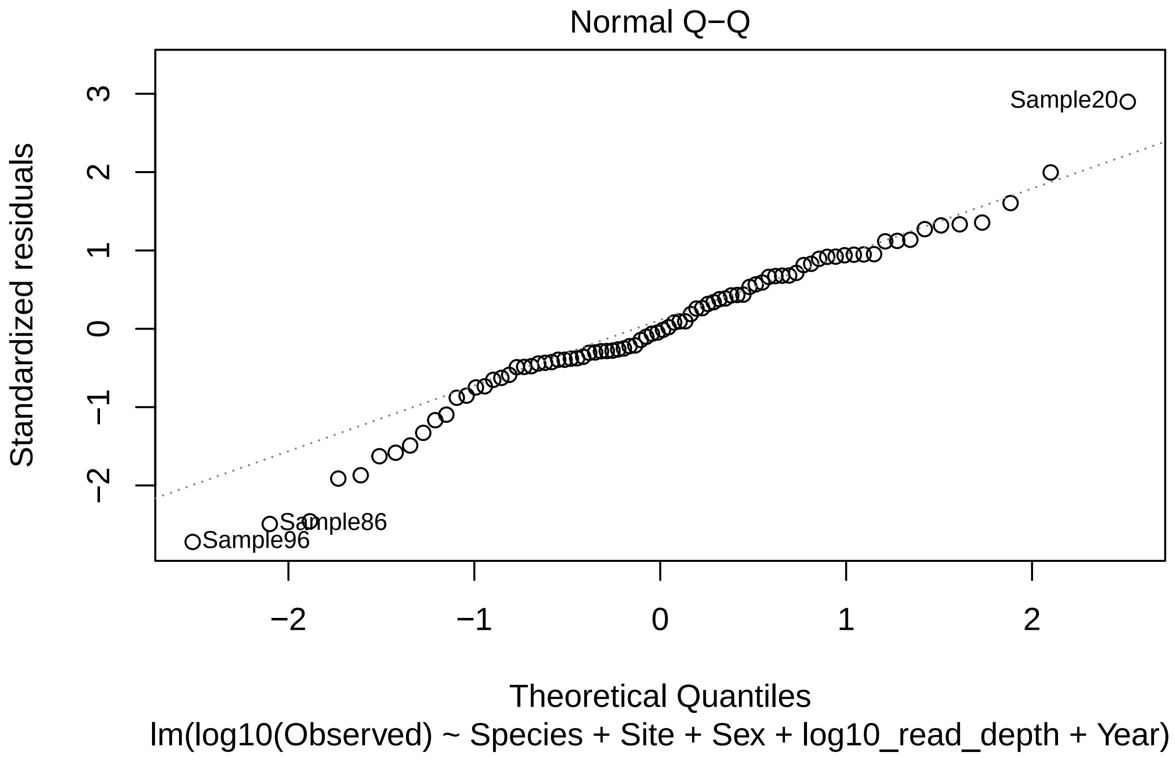


```
ols_plot_resid_stand(pl.observed.lm)
```

Table 9: Result: ANOVA comparing observed features diversity of turtle species by different factors

	Sum Sq	Df	F value	Pr(>F)
Species	2.8327797	5	17.052903	0.0000000
Site	0.5413980	2	8.147834	0.0006459
Sex	0.0812901	2	1.223385	0.3002748
log10_read_depth	0.5260161	1	15.832686	0.0001632
Year	0.3350145	1	10.083683	0.0022024
Residuals	2.3920870	72	NA	NA

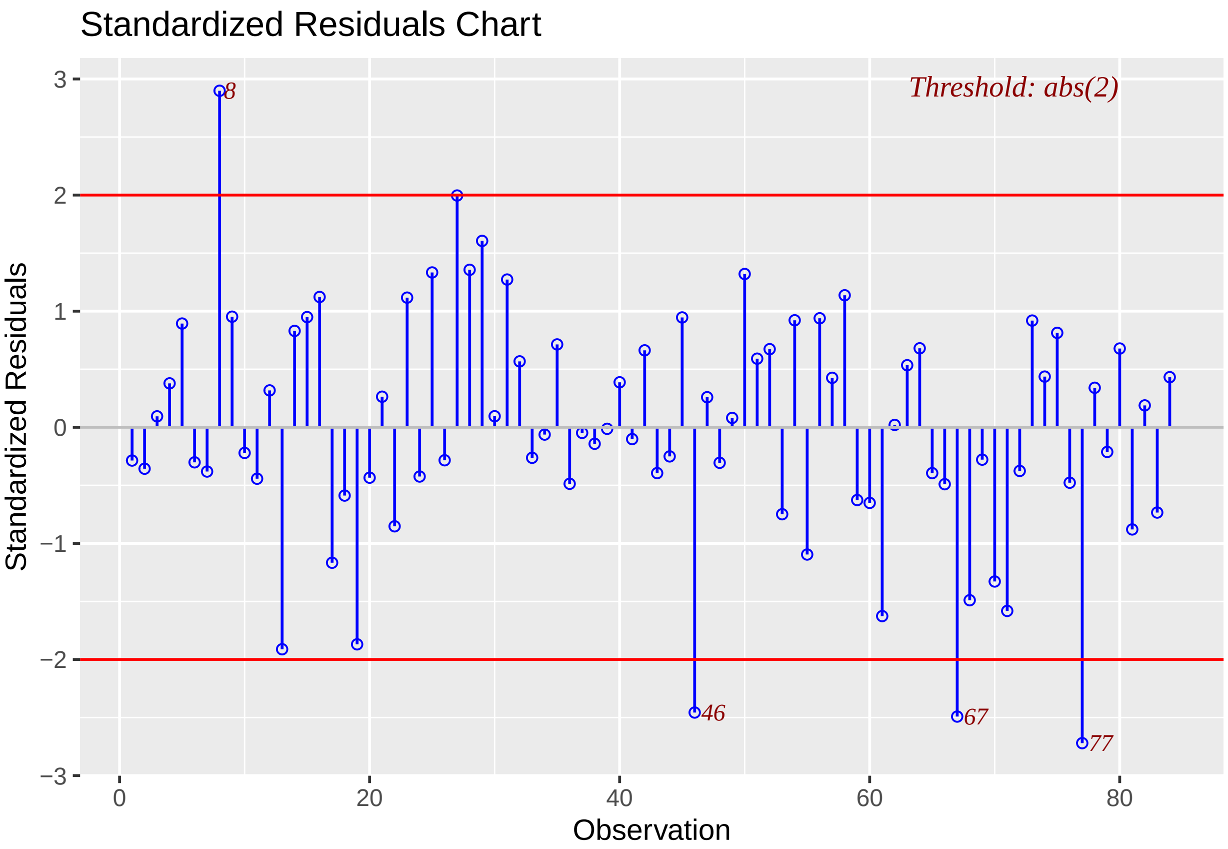


```
#ANOVA with pairwise comparisons (Tukey adjustment for pairwise comparisons):
kable.wrap(Anova(pl.observed.lm),"Result: ANOVA comparing observed features diversity of turtle species
```

```
emmeans(pl.observed.lm, list(pairwise ~ Species), adjust = "tukey")
```

```
## $`emmeans of Species`
##  Species emmean     SE df lower.CL upper.CL
##  CHSE      1.52 0.1183 72     1.28     1.75
##  KISU      1.78 0.0626 72     1.65     1.90
```

```
##  PSCO      1.97 0.0695 72     1.83     2.11
##  STCA      1.38 0.0858 72     1.21     1.55
##  STOD      1.53 0.0527 72     1.43     1.64
##  TRSC      1.91 0.0483 72     1.81     2.00
##
## Results are averaged over the levels of: Site, Sex, Year
## Results are given on the log10 (not the response) scale.
## Confidence level used: 0.95
##
## $`pairwise differences of Species`
##  1            estimate     SE df t.ratio p.value
##  CHSE - KISU  -0.2642 0.1202 72  -2.198  0.2516
##  CHSE - PSCO  -0.4570 0.1279 72  -3.574  0.0080
##  CHSE - STCA   0.1382 0.1471 72   0.939  0.9348
##  CHSE - STOD  -0.0154 0.1166 72  -0.132  1.0000
##  CHSE - TRSC  -0.3910 0.1170 72  -3.341  0.0161
##  KISU - PSCO  -0.1929 0.0904 72  -2.132  0.2827
##  KISU - STCA   0.4024 0.1099 72   3.663  0.0061
##  KISU - STOD   0.2488 0.0644 72   3.863  0.0032
##  KISU - TRSC  -0.1268 0.0667 72  -1.901  0.4100
##  PSCO - STCA   0.5952 0.1213 72   4.907  0.0001
##  PSCO - STOD   0.4417 0.0860 72   5.138  <.0001
##  PSCO - TRSC   0.0660 0.0845 72   0.781  0.9698
##  STCA - STOD  -0.1535 0.1043 72  -1.472  0.6826
##  STCA - TRSC  -0.5292 0.0937 72  -5.647  <.0001
##  STOD - TRSC  -0.3756 0.0568 72  -6.610  <.0001
##
## Results are averaged over the levels of: Site, Sex, Year
## Results are given on the log10 (not the response) scale.
## P value adjustment: tukey method for comparing a family of 6 estimates
```

```
emmeans(pl.observed.lm, list(pairwise ~ Site), adjust = "tukey")
```

```
## $`emmeans of Site`
##  Site emmean     SE df lower.CL upper.CL
##  BP4    1.56 0.0388 72     1.48     1.63
##  S1     1.88 0.0720 72     1.73     2.02
##  S4     1.61 0.0527 72     1.50     1.71
##
## Results are averaged over the levels of: Species, Sex, Year
## Results are given on the log10 (not the response) scale.
## Confidence level used: 0.95
##
## $`pairwise differences of Site`
##  1         estimate     SE df t.ratio p.value
##  BP4 - S1  -0.3191 0.0792 72  -4.029  0.0004
##  BP4 - S4  -0.0493 0.0531 72  -0.928  0.6244
##  S1 - S4    0.2698 0.0815 72   3.310  0.0041
##
## Results are averaged over the levels of: Species, Sex, Year
## Results are given on the log10 (not the response) scale.
## P value adjustment: tukey method for comparing a family of 3 estimates
```

```r
#Rerun above analysis, but with standardized residual outliers removed
carapace_observed_no_outliers<-carapace[-c(1,2,13,25,46),]
summary(carapace_observed_no_outliers)
```

```
##   sample.ID         LibraryName          Read_depth     ProjectName
##  Length:79          Length:79          Min.   : 46939   Length:79
##  Class :character   Class :character   1st Qu.: 70600   Class :character
##  Mode  :character   Mode  :character   Median : 88285   Mode  :character
##                                        Mean   : 87543
##                                        3rd Qu.:103329
##                                        Max.   :174528
##     Region          Sample_date             Year          Site
##  Length:79          Length:79          Min.   :2021   Length:79
##  Class :character   Class :character   1st Qu.:2022   Class :character
##  Mode  :character   Mode  :character   Median :2022   Mode  :character
##                                        Mean   :2022
##                                        3rd Qu.:2022
##                                        Max.   :2022
##  Sample_type         Substrate            Species          Species_substrate
##  Length:79          Length:79          Length:79          Length:79
##  Class :character   Class :character   Class :character   Class :character
##  Mode  :character   Mode  :character   Mode  :character   Mode  :character
##
##
##
##  Species_site          Sorter               Sex            Carapace_length
##  Length:79          Length:79          Length:79          Min.   : 49.4
##  Class :character   Class :character   Class :character   1st Qu.: 87.0
##  Mode  :character   Mode  :character   Mode  :character   Median :100.0
##                                                           Mean   :125.8
##                                                           3rd Qu.:167.3
##                                                           Max.   :308.0
##  Plastron_length      Mass         Sample_number       Sex_notes
##  Min.   : 35.8   Min.   :  25.0   Length:79          Length:79
##  1st Qu.: 65.0   1st Qu.: 110.0   Class :character   Class :character
##  Median : 82.6   Median : 170.0   Mode  :character   Mode  :character
##  Mean   :104.9   Mean   : 507.3
##  3rd Qu.:152.0   3rd Qu.: 685.0
##  Max.   :239.0   Max.   :6600.0
##   PCR1_date         extraction_date       Observed          Chao1
##  Length:79          Min.   :    2021   Min.   :  9.00   Min.   :  9.00
##  Class :character   1st Qu.:20220617   1st Qu.: 29.50   1st Qu.: 29.50
##  Mode  :character   Median :20220623   Median : 47.00   Median : 48.00
##                     Mean   :15357920   Mean   : 52.06   Mean   : 52.23
##                     3rd Qu.:20220624   3rd Qu.: 70.50   3rd Qu.: 70.92
##                     Max.   :20220624   Max.   :130.00   Max.   :130.00
##     se.chao1           ACE             se.ACE         Shannon
##  Min.   :0.0000   Min.   :  9.00   Min.   :1.414   Min.   :0.0194
##  1st Qu.:0.0000   1st Qu.: 29.50   1st Qu.:2.411   1st Qu.:0.9460
##  Median :0.0000   Median : 47.64   Median :2.845   Median :1.4100
##  Mean   :0.4183   Mean   : 52.25   Mean   :2.872   Mean   :1.5199
##  3rd Qu.:0.4934   3rd Qu.: 71.24   3rd Qu.:3.495   3rd Qu.:2.1100
##  Max.   :4.6460   Max.   :130.28   Max.   :4.463   Max.   :3.1900
```

```
##     Simpson           InvSimpson     log10_read_depth       PD
##  Min.   :0.00427   Min.   : 1.004   Min.   :4.672    Min.   : 2.675
##  1st Qu.:0.41100   1st Qu.: 1.700   1st Qu.:4.849    1st Qu.: 6.928
##  Median :0.62500   Median : 2.666   Median :4.946    Median : 8.666
##  Mean   :0.57202   Mean   : 3.660   Mean   :4.926    Mean   : 9.253
##  3rd Qu.:0.77300   3rd Qu.: 4.405   3rd Qu.:5.014    3rd Qu.:11.344
##  Max.   :0.92900   Max.   :13.991   Max.   :5.242    Max.   :19.537
```

```
#pl.observed_no_outliers.lm <- lm(Observed~Species+Site+Plastron_length+Sex+log10_read_depth+Year,data=
pl.observed_no_outliers.lm <- lm(Observed~Species+Site+Sex+log10_read_depth+Year,data=carapace_observed_

summary(pl.observed_no_outliers.lm)
```

```
##
## Call:
## lm(formula = Observed ~ Species + Site + Sex + log10_read_depth +
##     Year, data = carapace_observed_no_outliers)
##
## Residuals:
##     Min      1Q  Median      3Q     Max
## -37.238 -12.514   0.594   9.721  49.974
##
## Coefficients:
##                   Estimate Std. Error t value Pr(>|t|)
## (Intercept)      39973.685  10734.377   3.724 0.000404 ***
## SpeciesKISU         23.480     11.338   2.071 0.042219 *
## SpeciesPSCO         39.510     11.943   3.308 0.001512 **
## SpeciesSTCA        -29.958     14.036  -2.134 0.036475 *
## SpeciesSTOD         -3.394     10.930  -0.311 0.757132
## SpeciesTRSC         32.038     11.039   2.902 0.005011 **
## SiteS1              45.528      7.553   6.028 7.93e-08 ***
## SiteS4               1.587      5.091   0.312 0.756182
## SexJ                 7.849      8.401   0.934 0.353463
## SexM                 9.461      4.364   2.168 0.033740 *
## log10_read_depth    91.205     20.789   4.387 4.16e-05 ***
## Year               -19.983      5.289  -3.778 0.000338 ***
## ---
## Signif. codes:  0 '***' 0.001 '**' 0.01 '*' 0.05 '.' 0.1 ' ' 1
##
## Residual standard error: 17.01 on 67 degrees of freedom
## Multiple R-squared:  0.721,  Adjusted R-squared:  0.6752
## F-statistic: 15.74 on 11 and 67 DF,  p-value: 1.276e-14
```

```
confint(pl.observed_no_outliers.lm)
```

```
##                          2.5 %       97.5 %
## (Intercept)      18547.7779403 61399.591077
## SpeciesKISU          0.8496801    46.111107
## SpeciesPSCO         15.6724852    63.348124
## SpeciesSTCA        -57.9740034    -1.941978
## SpeciesSTOD        -25.2107065    18.422712
## SpeciesTRSC         10.0035540    54.072255
## SiteS1              30.4527545    60.602622
```

```
## SiteS4                 -8.5736477    11.747812
## SexJ                   -8.9183756    24.617202
## SexM                    0.7492520    18.172190
## log10_read_depth       49.7106110   132.699090
## Year                  -30.5401047    -9.425551
```

```
check_model(pl.observed_no_outliers.lm)
```

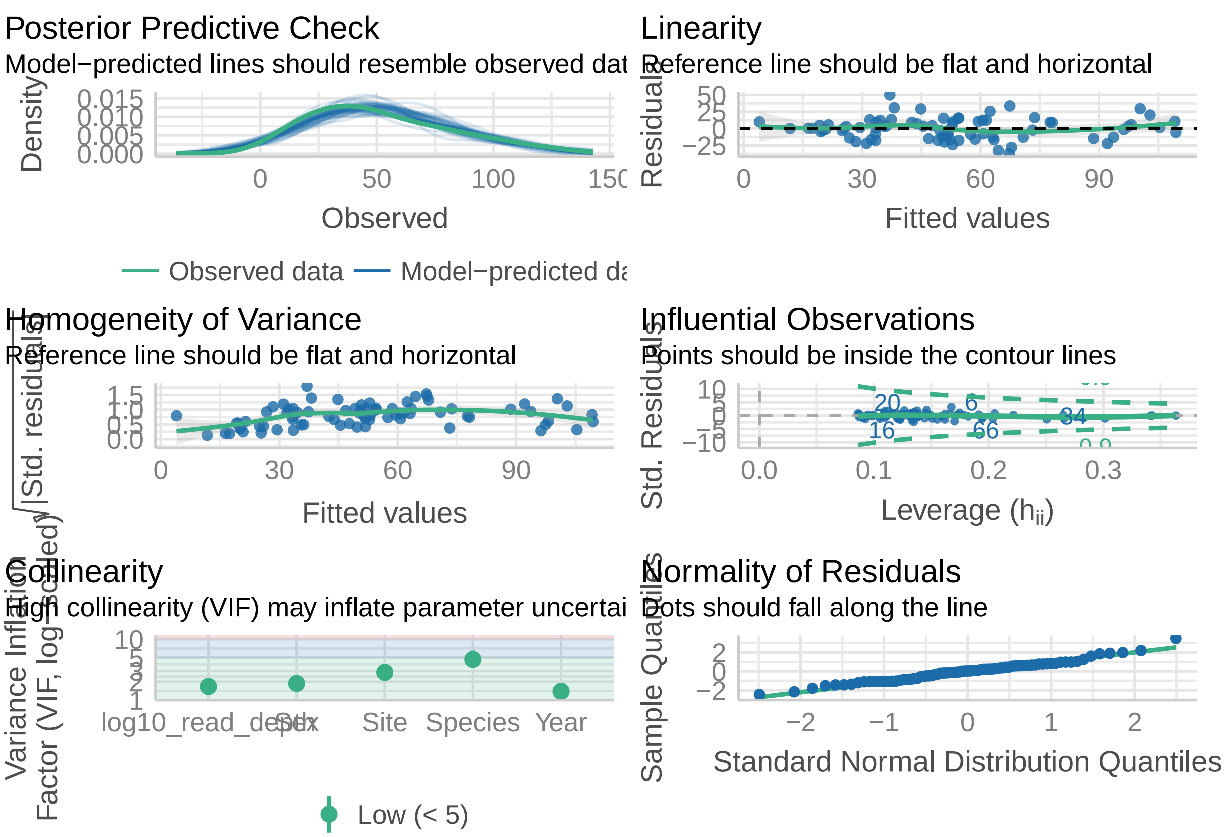


```
plot(pl.observed_no_outliers.lm, which = 1)
```

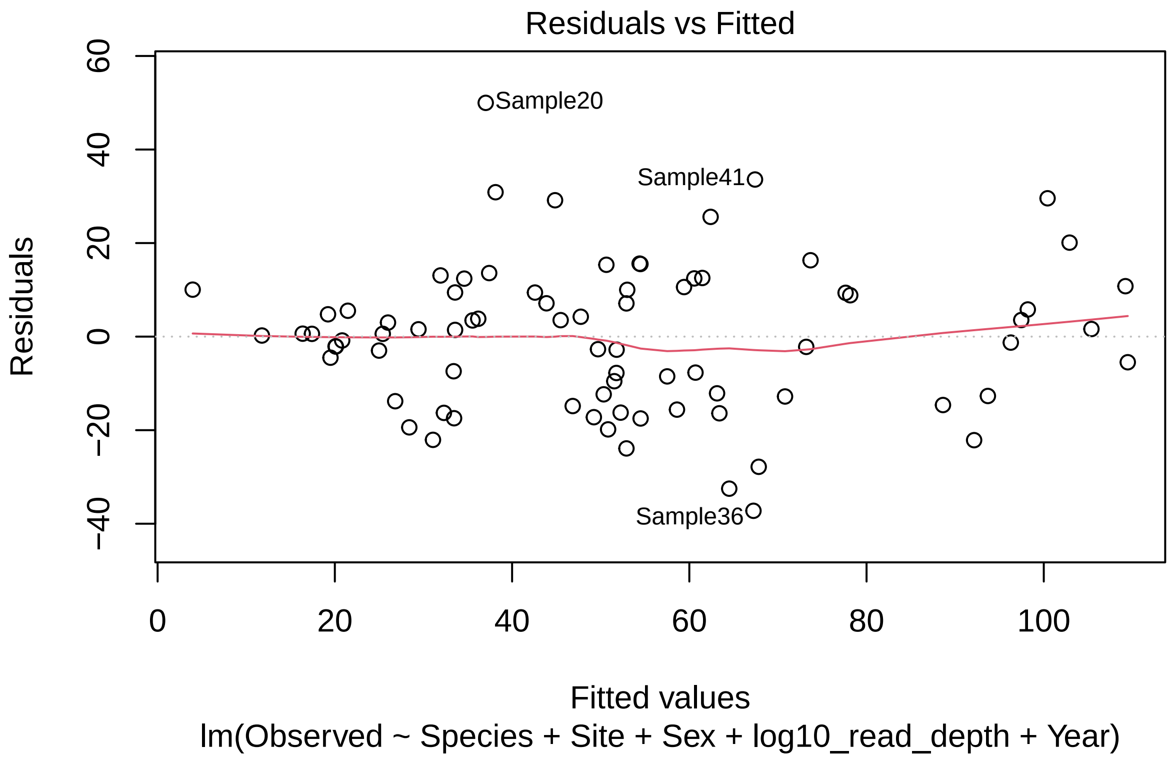


```
plot(pl.observed_no_outliers.lm, which = 2)
```

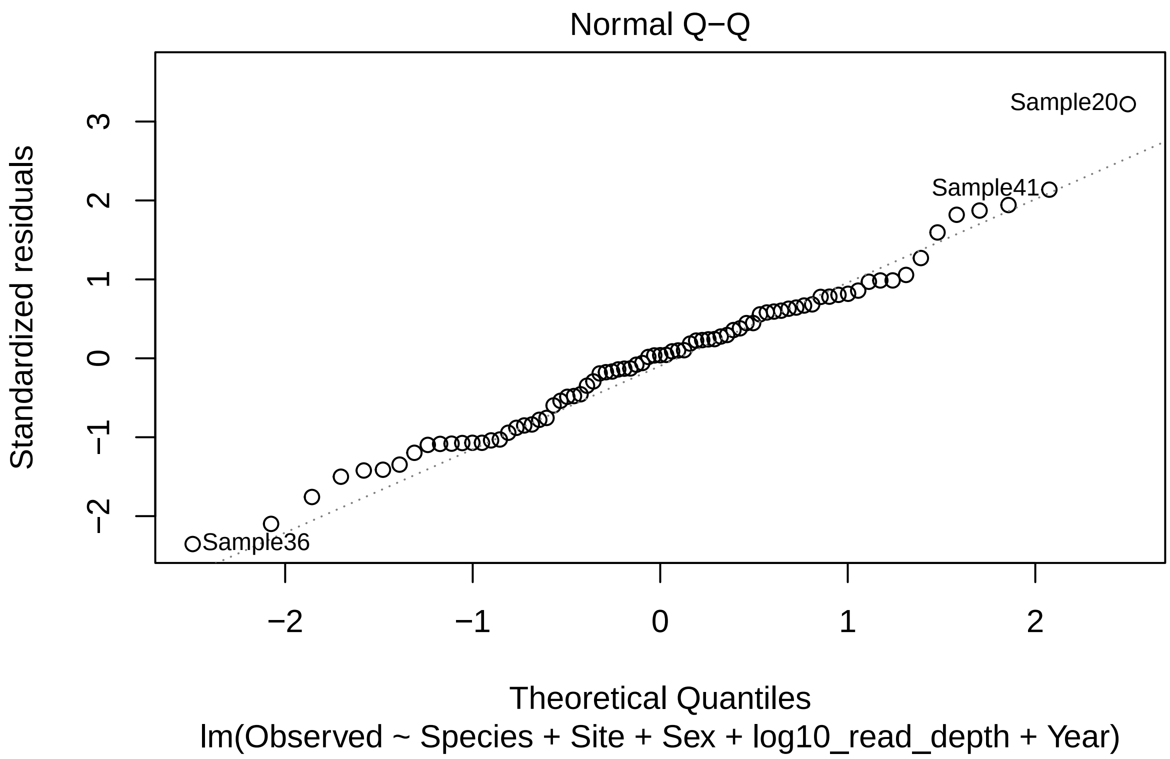


```
#ANOVA with pairwise comparisons (Tukey adjustment for pairwise comparisons):
kable.wrap(Anova(pl.observed_no_outliers.lm),"Result: ANOVA comparing observed features diversity of tu
```

Table 10: Result: ANOVA comparing observed features diversity of turtle species by different factors, standardized residual outliers removed

	Sum Sq	Df	F value	Pr(>F)
Species	29054.904	5	20.088468	0.0000000
Site	11319.169	2	19.565093	0.0000002
Sex	1371.622	2	2.370838	0.1011952
log10_read_depth	5567.851	1	19.247971	0.0000416
Year	4128.929	1	14.273641	0.0003384
Residuals	19381.055	67	NA	NA

```r
emmeans(pl.observed_no_outliers.lm, list(pairwise ~ Species), adjust = "tukey")
```

```
## $`emmeans of Species`
##  Species emmean    SE df lower.CL upper.CL
##  CHSE      49.2 11.14 67     26.9     71.4
##  KISU      72.7  6.27 67     60.1     85.2
##  PSCO      88.7  6.61 67     75.5    101.9
##  STCA      19.2  8.37 67      2.5     35.9
##  STOD      45.8  5.08 67     35.6     55.9
##  TRSC      81.2  4.55 67     72.1     90.3
##
## Results are averaged over the levels of: Site, Sex, Year
## Confidence level used: 0.95
##
## $`pairwise differences of Species`
##  1           estimate    SE df t.ratio p.value
##  CHSE - KISU   -23.48 11.34 67  -2.071  0.3149
##  CHSE - PSCO   -39.51 11.94 67  -3.308  0.0181
##  CHSE - STCA    29.96 14.04 67   2.134  0.2826
##  CHSE - STOD     3.39 10.93 67   0.311  0.9996
##  CHSE - TRSC   -32.04 11.04 67  -2.902  0.0542
##  KISU - PSCO   -16.03  8.66 67  -1.851  0.4408
##  KISU - STCA    53.44 10.81 67   4.944  0.0001
##  KISU - STOD    26.87  6.26 67   4.294  0.0008
##  KISU - TRSC    -8.56  6.56 67  -1.305  0.7811
##  PSCO - STCA    69.47 11.63 67   5.975  <.0001
##  PSCO - STOD    42.90  8.10 67   5.296  <.0001
##  PSCO - TRSC     7.47  8.02 67   0.932  0.9368
##  STCA - STOD   -26.56 10.18 67  -2.610  0.1089
##  STCA - TRSC   -62.00  9.11 67  -6.802  <.0001
##  STOD - TRSC   -35.43  5.44 67  -6.516  <.0001
##
## Results are averaged over the levels of: Site, Sex, Year
## P value adjustment: tukey method for comparing a family of 6 estimates
```

```r
emmeans(pl.observed_no_outliers.lm, list(pairwise ~ Site), adjust = "tukey")
```

```
## $`emmeans of Site`
##  Site emmean   SE df lower.CL upper.CL
##  BP4    43.7 3.70 67     36.4     51.1
##  S1     89.3 6.91 67     75.5    103.1
##  S4     45.3 5.07 67     35.2     55.5
##
## Results are averaged over the levels of: Species, Sex, Year
## Confidence level used: 0.95
##
## $`pairwise differences of Site`
##  1        estimate   SE df t.ratio p.value
##  BP4 - S1   -45.53 7.55 67  -6.028  <.0001
##  BP4 - S4    -1.59 5.09 67  -0.312  0.9479
##  S1 - S4     43.94 7.66 67   5.733  <.0001
##
## Results are averaged over the levels of: Species, Sex, Year
## P value adjustment: tukey method for comparing a family of 3 estimates
```

```r
#Testing for Faith's Phylogenetic Diversity, all samples included
#NOTE: Reordering the factors in the below model does not change the resulting p-values
pl.PD.lm <- lm(PD~Species+Site+Sex+log10_read_depth+Year,data=carapace)

summary(pl.PD.lm)
```

```
##
## Call:
## lm(formula = PD ~ Species + Site + Sex + log10_read_depth + Year,
##     data = carapace)
##
## Residuals:
##     Min      1Q  Median      3Q     Max
## -3.6302 -1.5025  0.0751  1.2854  5.5914
##
## Coefficients:
##                   Estimate Std. Error t value Pr(>|t|)
## (Intercept)      3531.1179  1218.2079   2.899 0.004964 **
## SpeciesKISU         1.5962     1.3816   1.155 0.251774
## SpeciesPSCO         4.1413     1.4700   2.817 0.006248 **
## SpeciesSTCA        -6.1436     1.6906  -3.634 0.000520 ***
## SpeciesSTOD        -1.0267     1.3406  -0.766 0.446290
## SpeciesTRSC         3.4946     1.3451   2.598 0.011366 *
## SiteS1              7.1018     0.9104   7.801 3.61e-11 ***
## SiteS4              0.6864     0.6103   1.125 0.264442
## SexJ                0.7775     1.0315   0.754 0.453444
## SexM                1.2537     0.5247   2.390 0.019492 *
## log10_read_depth    9.4874     2.3901   3.969 0.000169 ***
## Year               -1.7667     0.6006  -2.942 0.004390 **
## ---
## Signif. codes:  0 '***' 0.001 '**' 0.01 '*' 0.05 '.' 0.1 ' ' 1
##
```

```
## Residual standard error: 2.095 on 72 degrees of freedom
## Multiple R-squared:  0.7305, Adjusted R-squared:  0.6893
## F-statistic: 17.74 on 11 and 72 DF,  p-value: < 2.2e-16
```

```r
confint(pl.PD.lm)
```

```
##                         2.5 %       97.5 %
## (Intercept)      1102.6647972 5959.5709798
## SpeciesKISU        -1.1579833    4.3504569
## SpeciesPSCO         1.2109019    7.0716595
## SpeciesSTCA        -9.5137569   -2.7734735
## SpeciesSTOD        -3.6990885    1.6457864
## SpeciesTRSC         0.8131334    6.1760965
## SiteS1              5.2868850    8.9166309
## SiteS4             -0.5301490    1.9028764
## SexJ               -1.2787706    2.8338360
## SexM                0.2077866    2.2995447
## log10_read_depth    4.7228602   14.2520025
## Year               -2.9640586   -0.5694373
```

```r
check_model(pl.PD.lm)
```

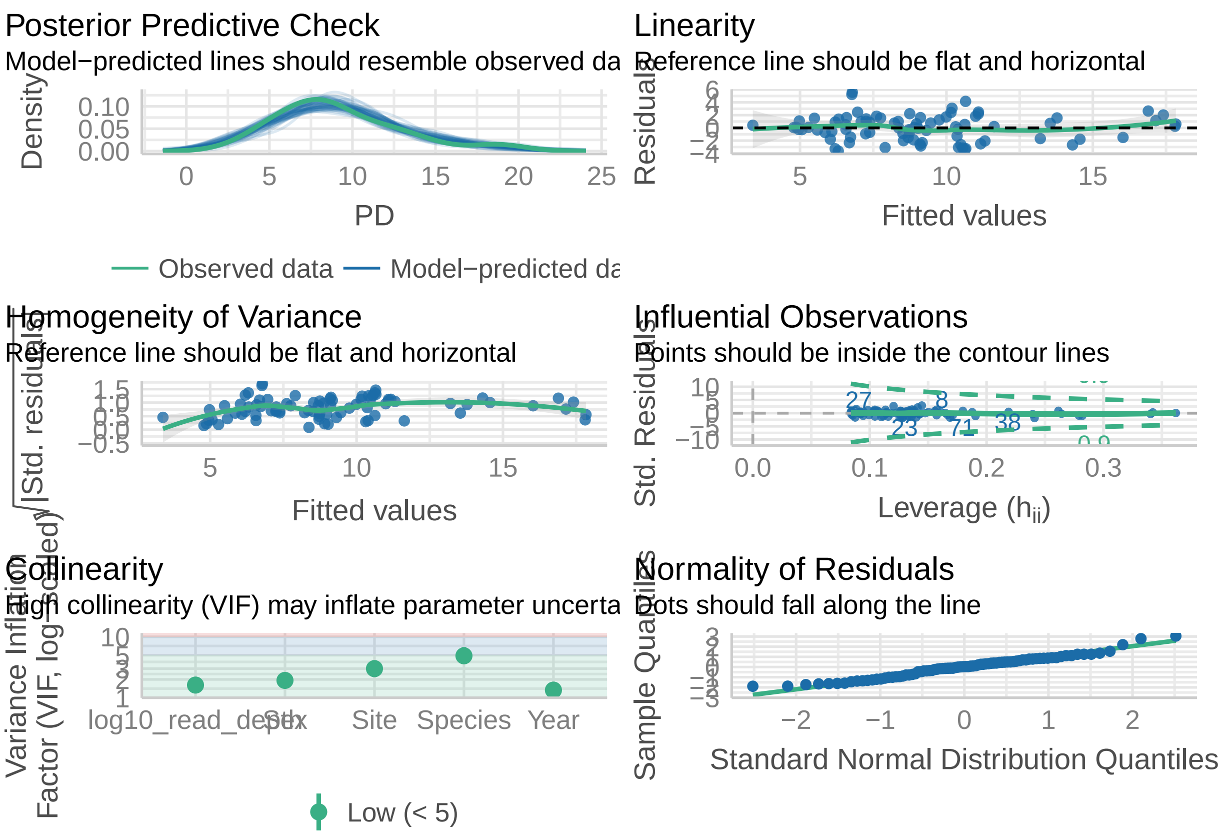


```r
plot(pl.PD.lm, which = 1)
```

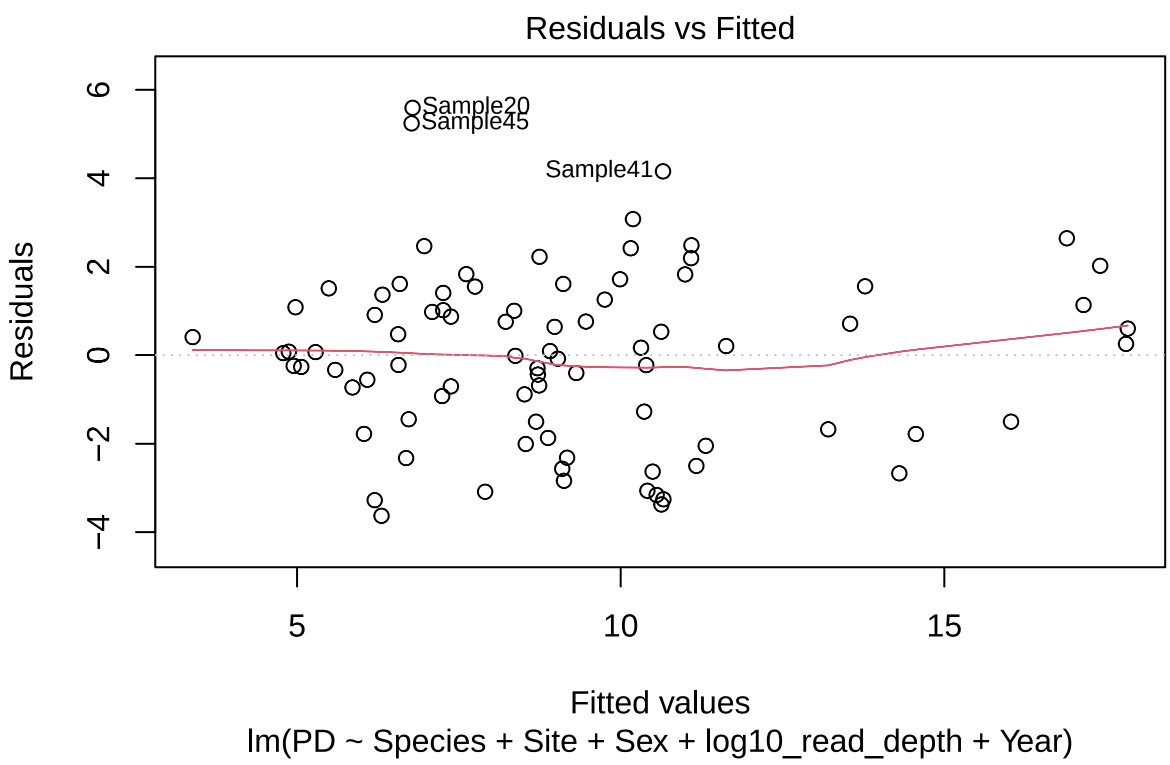


```
plot(pl.PD.lm, which = 2)
```

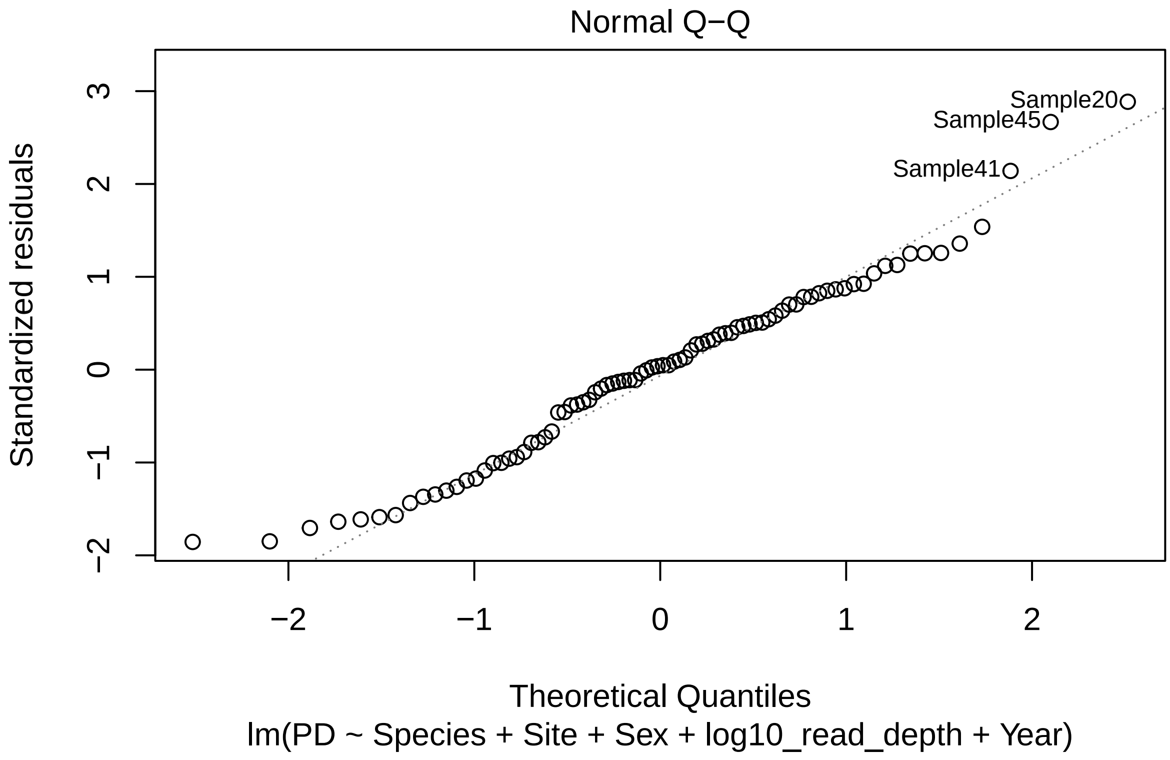


```
ols_plot_resid_stand(pl.PD.lm)#gives same outliers as resid_level, and more than resid_student, so will
```

Table 11: Result: Multiple regression comparing PD of turtle species by different factors

	Sum Sq	Df	F value	Pr(>F)
Species	569.37516	5	25.945035	0.0000000
Site	273.35397	2	31.140181	0.0000000
Sex	25.07314	2	2.856304	0.0640142
log10_read_depth	69.15769	1	15.756736	0.0001686
Year	37.97750	1	8.652709	0.0043900
Residuals	316.01431	72	NA	NA

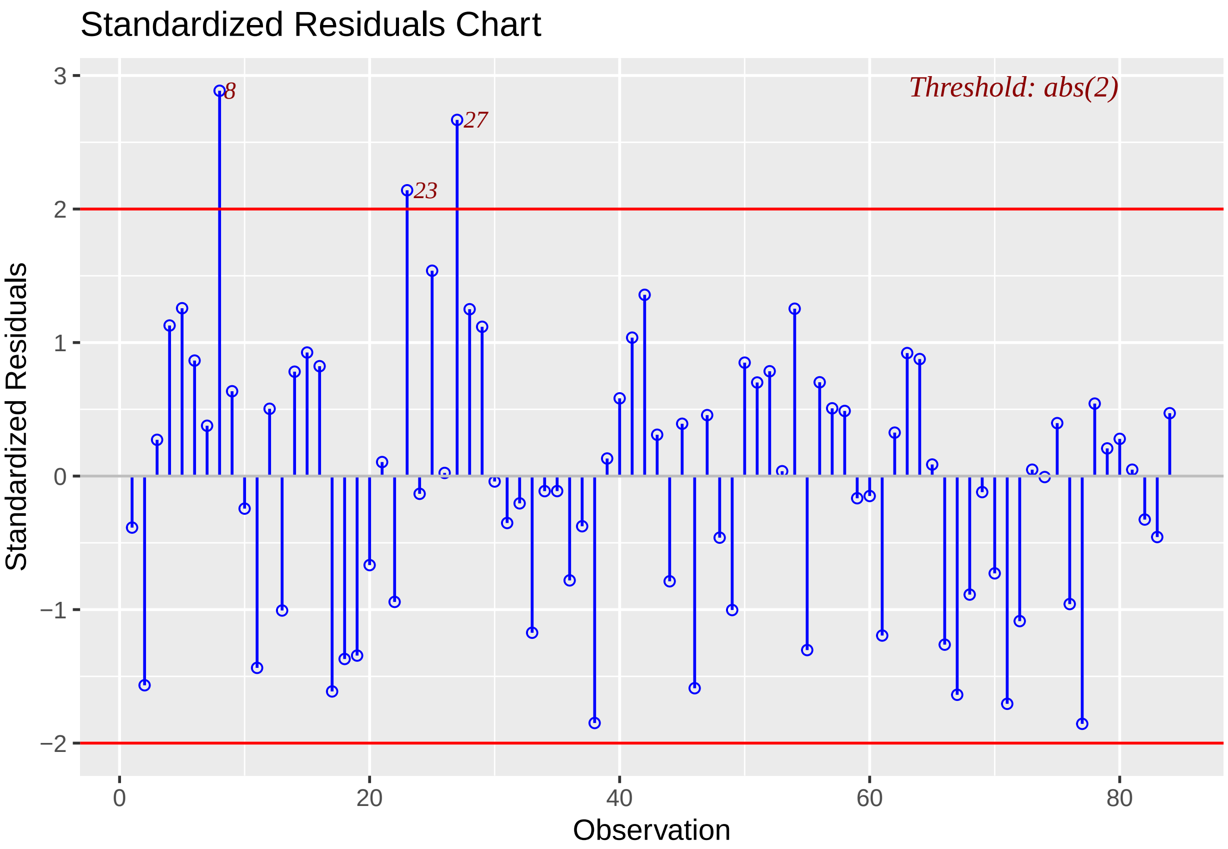


```
#ANOVA with pairwise comparisons (Tukey adjustment for pairwise comparisons):
kable.wrap(Anova(pl.PD.lm),"Result: Multiple regression comparing PD of turtle species by different fac
```

```
emmeans(pl.PD.lm, list(pairwise ~ Species), adjust = "tukey")
```

```
## $‘emmeans of Species‘
##  Species emmean    SE df lower.CL upper.CL
##  CHSE      9.65 1.360 72     6.94    12.36
##  KISU     11.24 0.720 72     9.81    12.68
```

```
##  PSCO     13.79 0.799 72    12.20    15.38
##  STCA      3.50 0.986 72     1.54     5.47
##  STOD      8.62 0.606 72     7.41     9.83
##  TRSC     13.14 0.555 72    12.03    14.25
##
## Results are averaged over the levels of: Site, Sex, Year
## Confidence level used: 0.95
##
## $`pairwise differences of Species`
##  1           estimate    SE df t.ratio p.value
##  CHSE - KISU   -1.596 1.382 72  -1.155  0.8562
##  CHSE - PSCO   -4.141 1.470 72  -2.817  0.0661
##  CHSE - STCA    6.144 1.691 72   3.634  0.0066
##  CHSE - STOD    1.027 1.341 72   0.766  0.9723
##  CHSE - TRSC   -3.495 1.345 72  -2.598  0.1110
##  KISU - PSCO   -2.545 1.040 72  -2.448  0.1538
##  KISU - STCA    7.740 1.263 72   6.130  <.0001
##  KISU - STOD    2.623 0.740 72   3.543  0.0088
##  KISU - TRSC   -1.898 0.767 72  -2.476  0.1450
##  PSCO - STCA   10.285 1.394 72   7.377  <.0001
##  PSCO - STOD    5.168 0.988 72   5.230  <.0001
##  PSCO - TRSC    0.647 0.972 72   0.666  0.9851
##  STCA - STOD   -5.117 1.199 72  -4.269  0.0008
##  STCA - TRSC   -9.638 1.077 72  -8.948  <.0001
##  STOD - TRSC   -4.521 0.653 72  -6.922  <.0001
##
## Results are averaged over the levels of: Site, Sex, Year
## P value adjustment: tukey method for comparing a family of 6 estimates
```

```
emmeans(pl.PD.lm, list(pairwise ~ Site), adjust = "tukey")
```

```
## $`emmeans of Site`
##  Site emmean    SE df lower.CL upper.CL
##  BP4    7.39 0.446 72     6.50     8.28
##  S1    14.50 0.827 72    12.85    16.14
##  S4     8.08 0.606 72     6.87     9.29
##
## Results are averaged over the levels of: Species, Sex, Year
## Confidence level used: 0.95
##
## $`pairwise differences of Site`
##  1        estimate    SE df t.ratio p.value
##  BP4 - S1   -7.102 0.910 72  -7.801  <.0001
##  BP4 - S4   -0.686 0.610 72  -1.125  0.5021
##  S1 - S4     6.415 0.937 72   6.847  <.0001
##
## Results are averaged over the levels of: Species, Sex, Year
## P value adjustment: tukey method for comparing a family of 3 estimates
```

```
#Rerun above analysis, but with standardized residual outliers removed
carapace_PD_no_outliers<-carapace[-c(25,71),]
summary(carapace_PD_no_outliers)
```

```
##   sample.ID         LibraryName          Read_depth     ProjectName
```

```
##  Length:82          Length:82          Min.   : 43496   Length:82
##  Class :character   Class :character   1st Qu.: 70299   Class :character
##  Mode  :character   Mode  :character   Median : 89076   Mode  :character
##                                        Mean   : 87772
##                                        3rd Qu.:103701
##                                        Max.   :174528
##     Region          Sample_date             Year          Site
##  Length:82          Length:82          Min.   :2021   Length:82
##  Class :character   Class :character   1st Qu.:2021   Class :character
##  Mode  :character   Mode  :character   Median :2022   Mode  :character
##                                        Mean   :2022
##                                        3rd Qu.:2022
##                                        Max.   :2022
##  Sample_type         Substrate           Species          Species_substrate
##  Length:82          Length:82          Length:82          Length:82
##  Class :character   Class :character   Class :character   Class :character
##  Mode  :character   Mode  :character   Mode  :character   Mode  :character
##
##
##
##  Species_site          Sorter               Sex           Carapace_length
##  Length:82          Length:82          Length:82          Min.   : 49.4
##  Class :character   Class :character   Class :character   1st Qu.: 87.0
##  Mode  :character   Mode  :character   Mode  :character   Median : 98.0
##                                                           Mean   :124.7
##                                                           3rd Qu.:163.8
##                                                           Max.   :308.0
##  Plastron_length      Mass        Sample_number       Sex_notes
##  Min.   : 35.8   Min.   :  25.0   Length:82          Length:82
##  1st Qu.: 65.0   1st Qu.: 110.0   Class :character   Class :character
##  Median : 83.3   Median : 170.0   Mode  :character   Mode  :character
##  Mean   :103.9   Mean   : 494.9
##  3rd Qu.:151.8   3rd Qu.: 662.5
##  Max.   :239.0   Max.   :6600.0
##   PCR1_date         extraction_date       Observed          Chao1
##  Length:82          Min.   :    2021   Min.   :  9.00   Min.   :  9.00
##  Class :character   1st Qu.:    2021   1st Qu.: 29.00   1st Qu.: 29.00
##  Mode  :character   Median :20220620   Median : 47.00   Median : 47.75
##                     Mean   :14796119   Mean   : 51.23   Mean   : 51.39
##                     3rd Qu.:20220623   3rd Qu.: 70.00   3rd Qu.: 70.50
##                     Max.   :20220624   Max.   :130.00   Max.   :130.00
##     se.chao1           ACE            se.ACE          Shannon
##  Min.   :0.0000   Min.   :  9.00   Min.   :0.9608   Min.   :0.0194
##  1st Qu.:0.0000   1st Qu.: 29.00   1st Qu.:2.3166   1st Qu.:0.9390
##  Median :0.0000   Median : 47.60   Median :2.8174   Median :1.4150
##  Mean   :0.4030   Mean   : 51.41   Mean   :2.8319   Mean   :1.5242
##  3rd Qu.:0.4914   3rd Qu.: 70.59   3rd Qu.:3.4746   3rd Qu.:2.1200
##  Max.   :4.6460   Max.   :130.28   Max.   :4.4630   Max.   :3.1900
##     Simpson          InvSimpson     log10_read_depth        PD
##  Min.   :0.00427   Min.   : 1.004   Min.   :4.638    Min.   : 2.675
##  1st Qu.:0.41825   1st Qu.: 1.720   1st Qu.:4.847    1st Qu.: 6.566
##  Median :0.62700   Median : 2.680   Median :4.950    Median : 8.389
##  Mean   :0.57435   Mean   : 3.678   Mean   :4.927    Mean   : 9.133
##  3rd Qu.:0.77550   3rd Qu.: 4.452   3rd Qu.:5.016    3rd Qu.:11.121
```

```
##  Max.   :0.92900   Max.   :13.991   Max.   :5.242    Max.   :19.537
```

```r
#pl.PD_no_outliers.lm <- lm(PD~Species+Site+Plastron_length+Sex+log10_read_depth+Year,data=carapace_PD_
pl.PD_no_outliers.lm <- lm(PD~Species+Site+Sex+log10_read_depth+Year,data=carapace_PD_no_outliers)

summary(pl.PD_no_outliers.lm)
```

```
##
## Call:
## lm(formula = PD ~ Species + Site + Sex + log10_read_depth + Year,
##     data = carapace_PD_no_outliers)
##
## Residuals:
##     Min      1Q  Median      3Q     Max
## -3.5449 -1.3452  0.0889  1.1622  5.6090
##
## Coefficients:
##                   Estimate Std. Error t value Pr(>|t|)
## (Intercept)      3638.7998  1197.1677   3.040 0.003331 **
## SpeciesKISU         1.5464     1.3519   1.144 0.256580
## SpeciesPSCO         4.7091     1.4811   3.179 0.002199 **
## SpeciesSTCA        -6.3012     1.6595  -3.797 0.000308 ***
## SpeciesSTOD        -1.0930     1.3116  -0.833 0.407474
## SpeciesTRSC         3.2794     1.3256   2.474 0.015793 *
## SiteS1              7.2655     0.9041   8.036 1.57e-11 ***
## SiteS4              0.7357     0.6021   1.222 0.225872
## SexJ                0.3464     1.0392   0.333 0.739879
## SexM                1.2534     0.5164   2.427 0.017802 *
## log10_read_depth    9.7035     2.3542   4.122 0.000102 ***
## Year               -1.8205     0.5902  -3.085 0.002918 **
## ---
## Signif. codes:  0 '***' 0.001 '**' 0.01 '*' 0.05 '.' 0.1 ' ' 1
##
## Residual standard error: 2.047 on 70 degrees of freedom
## Multiple R-squared:  0.7454, Adjusted R-squared:  0.7054
## F-statistic: 18.63 on 11 and 70 DF,  p-value: < 2.2e-16
```

```r
confint(pl.PD_no_outliers.lm)
```

```
##                         2.5 %       97.5 %
## (Intercept)      1251.1240909 6026.4755318
## SpeciesKISU        -1.1498903    4.2426193
## SpeciesPSCO         1.7550809    7.6631738
## SpeciesSTCA        -9.6109113   -2.9915658
## SpeciesSTOD        -3.7088222    1.5228146
## SpeciesTRSC         0.6355652    5.9232499
## SiteS1              5.4622245    9.0686905
## SiteS4             -0.4652096    1.9365964
## SexJ               -1.7262775    2.4190996
## SexM                0.2233807    2.2833432
## log10_read_depth    5.0080908   14.3988200
## Year               -2.9976531   -0.6433946
```

```r
check_model(pl.PD_no_outliers.lm)
```

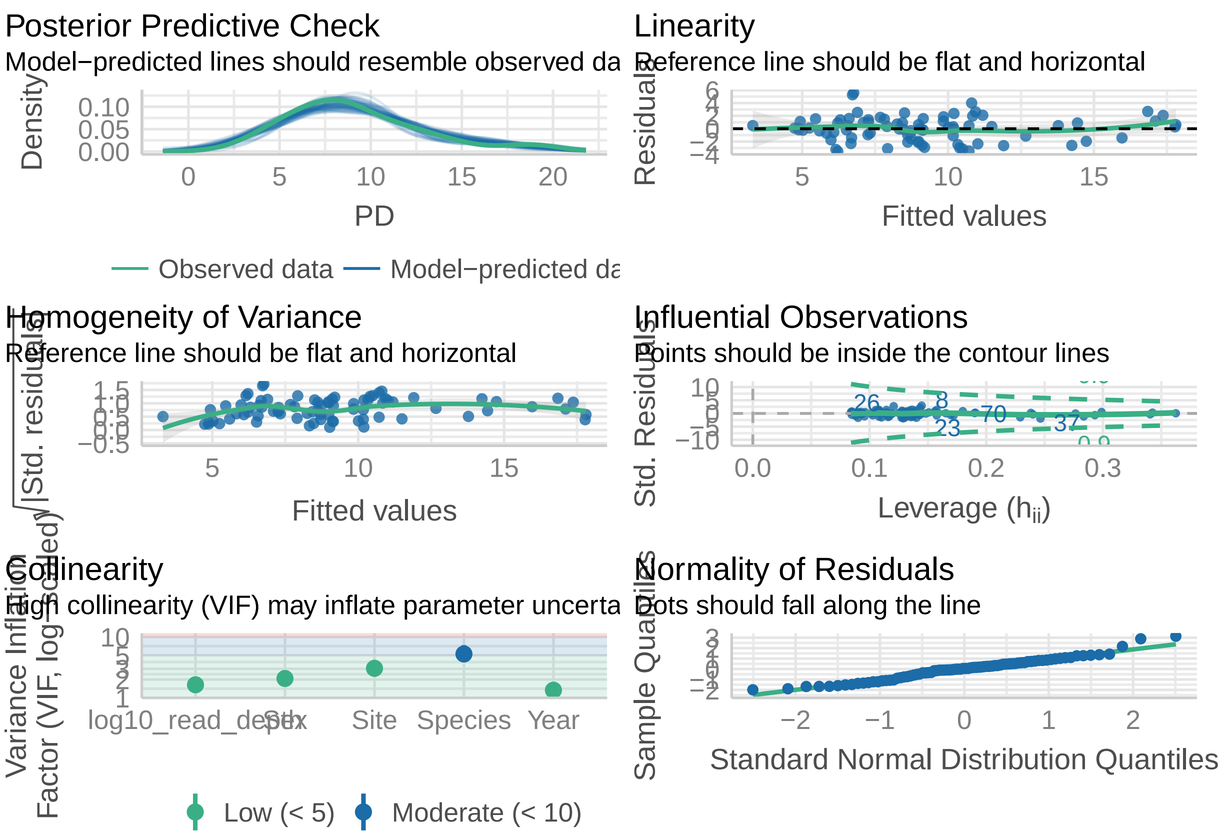


```r
plot(pl.PD_no_outliers.lm, which = 1)
```

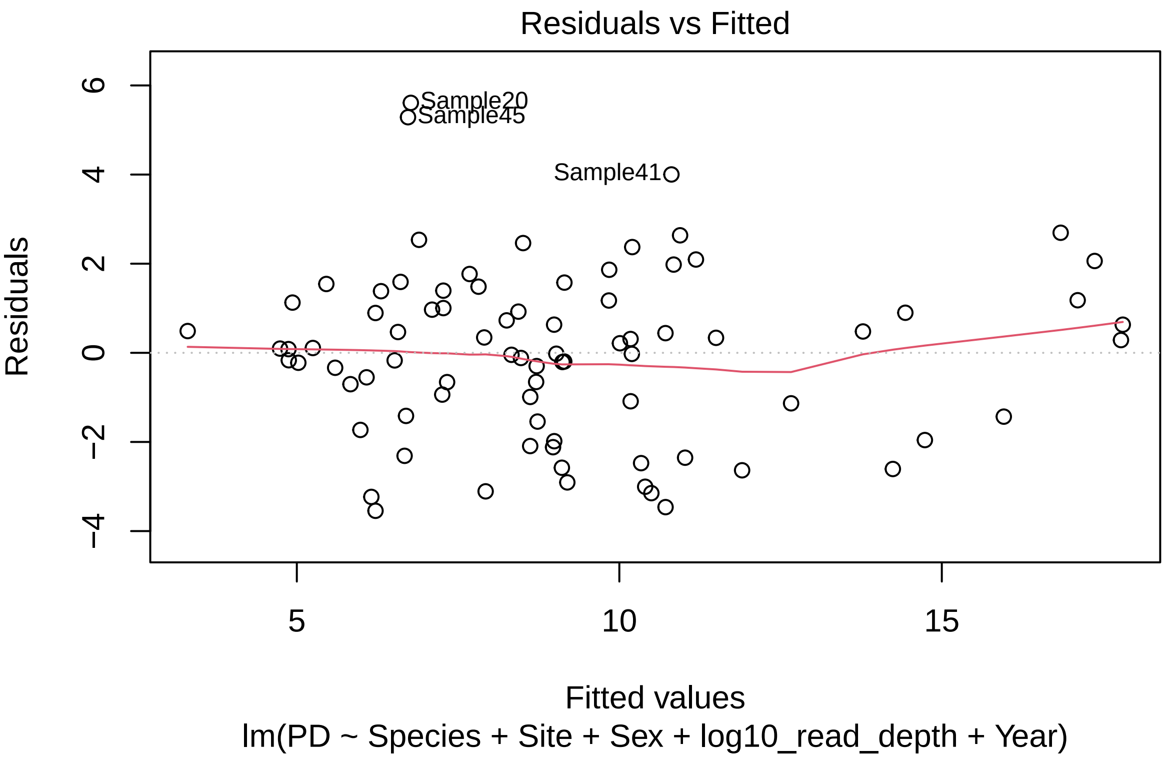


```
plot(pl.PD_no_outliers.lm, which = 2)
```

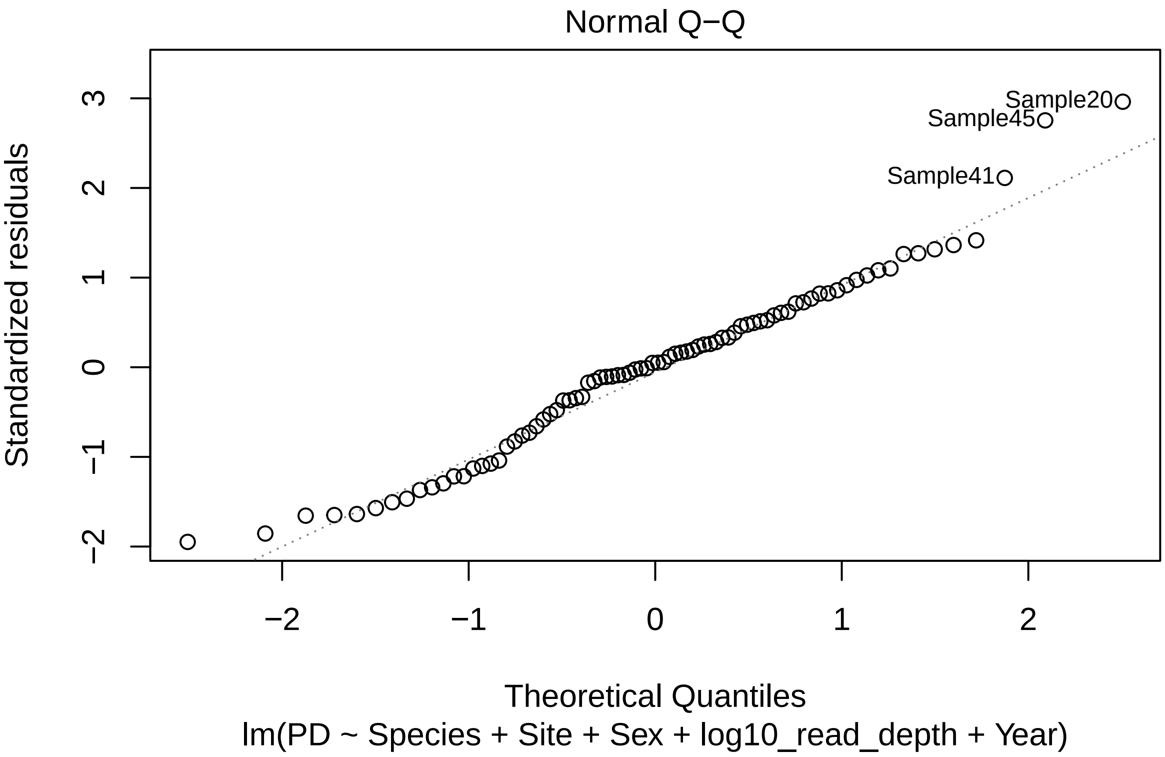


```
#ANOVA with pairwise comparisons (Tukey adjustment for pairwise comparisons):
kable.wrap(Anova(pl.PD_no_outliers.lm),"Result: Multiple regression comparing PD of turtle species by di
```

Table 12: Result: Multiple regression comparing PD of turtle species by different factors, standardized residual outliers removed

	Sum Sq	Df	F value	Pr(>F)
Species	566.26559	5	27.016661	0.0000000
Site	277.92613	2	33.149798	0.0000000
Sex	25.45238	2	3.035847	0.0544031
log10_read_depth	71.21531	1	16.988494	0.0001018
Year	39.88445	1	9.514481	0.0029177
Residuals	293.43812	70	NA	NA

```
emmeans(pl.PD_no_outliers.lm, list(pairwise ~ Species), adjust = "tukey")
```

```
## $`emmeans of Species`
##  Species emmean    SE df lower.CL upper.CL
##  CHSE      9.62 1.339 70     6.94    12.29
##  KISU     11.16 0.712 70     9.74    12.58
##  PSCO     14.32 0.820 70    12.69    15.96
##  STCA      3.31 0.968 70     1.38     5.24
##  STOD      8.52 0.604 70     7.32     9.73
##  TRSC     12.89 0.554 70    11.79    14.00
##
## Results are averaged over the levels of: Site, Sex, Year
## Confidence level used: 0.95
##
## $`pairwise differences of Species`
##  1            estimate    SE df t.ratio p.value
##  CHSE - KISU    -1.55 1.352 70  -1.144  0.8612
##  CHSE - PSCO    -4.71 1.481 70  -3.179  0.0257
##  CHSE - STCA     6.30 1.659 70   3.797  0.0040
##  CHSE - STOD     1.09 1.312 70   0.833  0.9603
##  CHSE - TRSC    -3.28 1.326 70  -2.474  0.1461
##  KISU - PSCO    -3.16 1.069 70  -2.958  0.0466
##  KISU - STCA     7.85 1.237 70   6.343  <.0001
##  KISU - STOD     2.64 0.724 70   3.648  0.0065
##  KISU - TRSC    -1.73 0.757 70  -2.290  0.2122
##  PSCO - STCA    11.01 1.404 70   7.845  <.0001
##  PSCO - STOD     5.80 1.026 70   5.658  <.0001
##  PSCO - TRSC     1.43 1.015 70   1.409  0.7217
##  STCA - STOD    -5.21 1.175 70  -4.432  0.0005
##  STCA - TRSC    -9.58 1.053 70  -9.099  <.0001
##  STOD - TRSC    -4.37 0.648 70  -6.750  <.0001
##
## Results are averaged over the levels of: Site, Sex, Year
## P value adjustment: tukey method for comparing a family of 6 estimates
```

```r
emmeans(pl.PD_no_outliers.lm, list(pairwise ~ Site), adjust = "tukey")
```

```
## $`emmeans of Site`
##  Site emmean    SE df lower.CL upper.CL
##  BP4    7.31 0.438 70     6.43     8.18
##  S1    14.57 0.818 70    12.94    16.20
##  S4     8.04 0.595 70     6.85     9.23
##
## Results are averaged over the levels of: Species, Sex, Year
## Confidence level used: 0.95
##
## $`pairwise differences of Site`
##  1        estimate    SE df t.ratio p.value
##  BP4 - S1   -7.265 0.904 70  -8.036  <.0001
##  BP4 - S4   -0.736 0.602 70  -1.222  0.4444
##  S1 - S4     6.530 0.919 70   7.103  <.0001
##
## Results are averaged over the levels of: Species, Sex, Year
## P value adjustment: tukey method for comparing a family of 3 estimates
```

Alpha diversity boxplots for carapace samples

The below plots show diversity distributions for carapace samples with standardized residual outliers removed, for Shannon diversity, observed species, and Faith's Phylogenetic Diversity. Plots are first by turtle species, then by site.

```r
ggplot(carapace_observed_no_outliers,aes(x=Species,y=Shannon))+
  geom_boxplot()+
  theme_classic()+
  theme(axis.text.x = element_text(angle = 90,hjust = 0.5),axis.title.x=element_blank())+
  ggtitle("18S Shannon diversity carapace samples (patterns by species) (standardized residual outliers
```

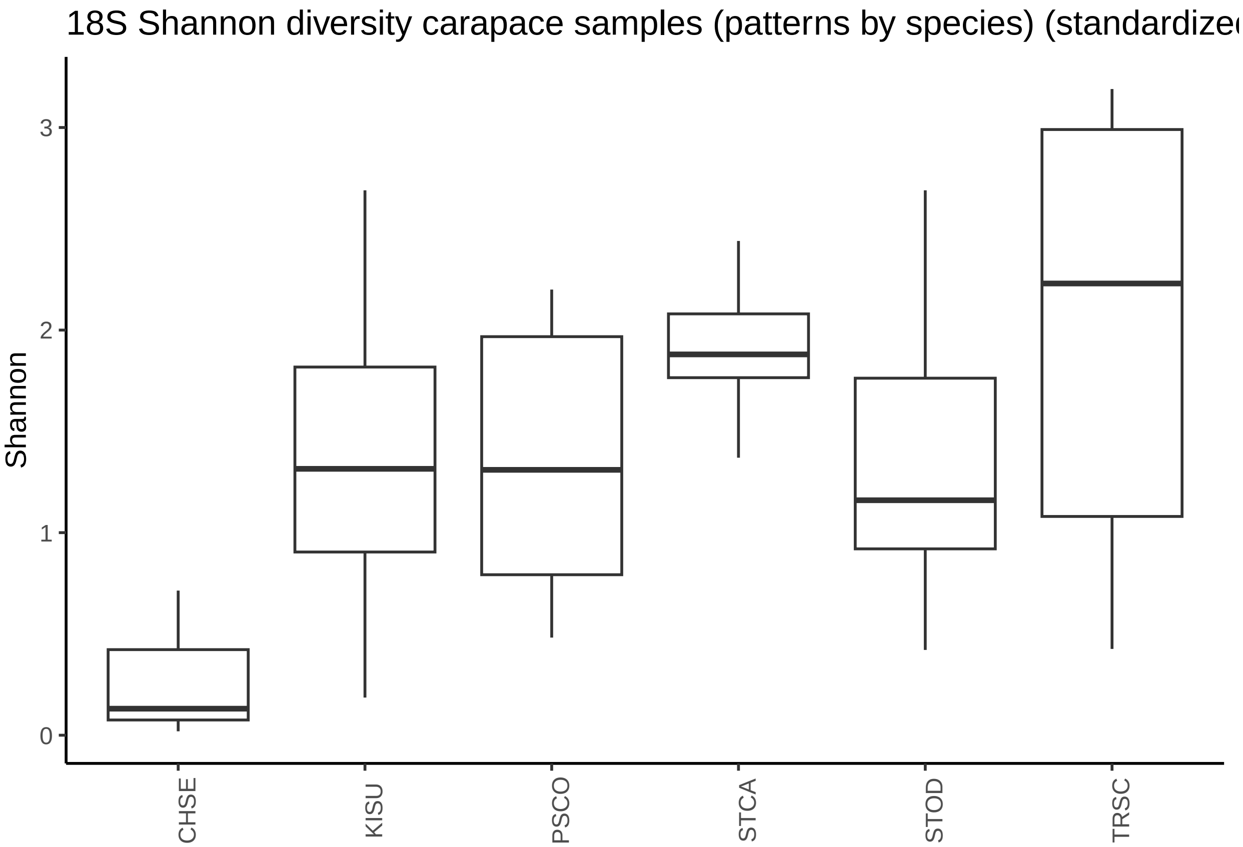


```
ggplot(carapace_shannon_no_outliers,aes(x=Species,y=Observed))+
  geom_boxplot()+
  theme_classic()+
  theme(axis.text.x = element_text(angle = 90,hjust = 0.5),axis.title.x=element_blank())+
  ggtitle("18S Observed features carapace samples (patterns by species) (standardized residual outliers
```

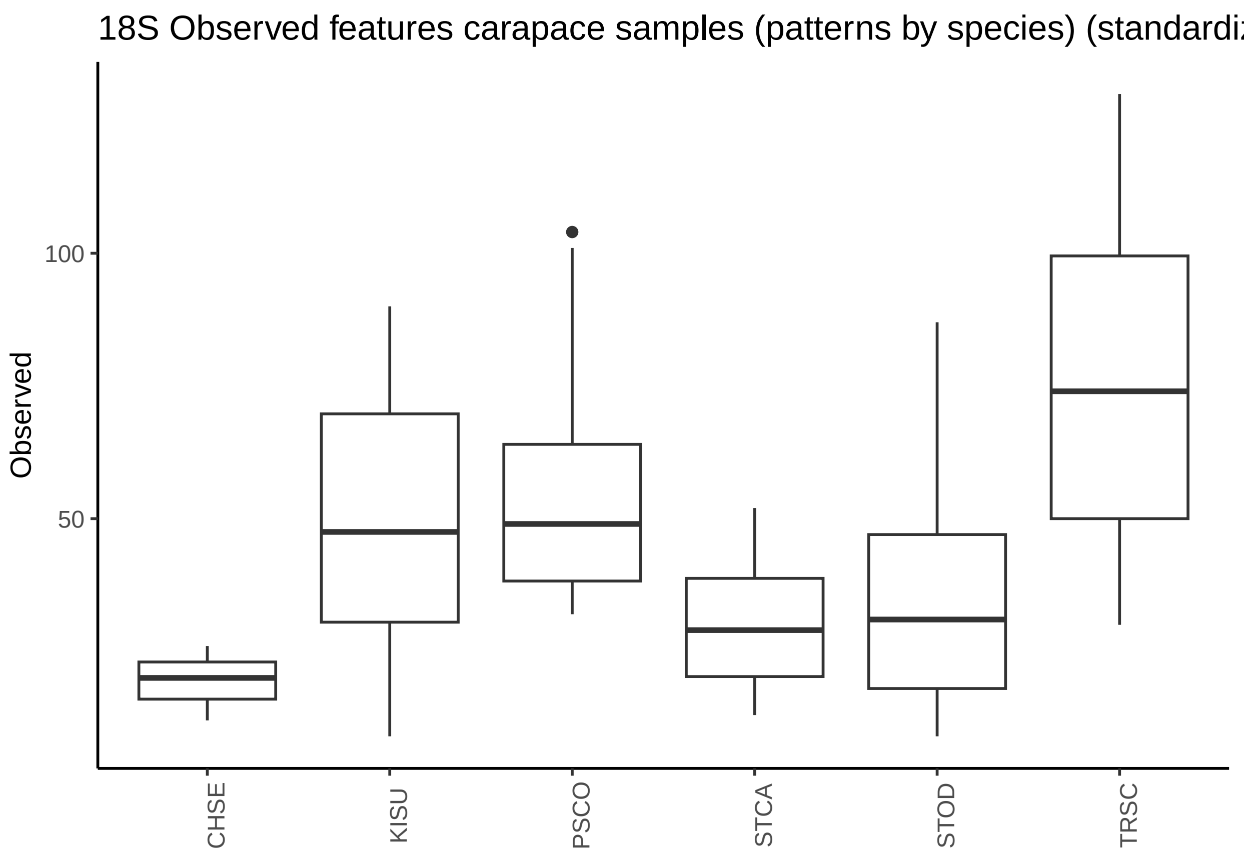


```
ggplot(carapace_PD_no_outliers,aes(x=Species,y=PD))+
  geom_boxplot()+
  theme_classic()+
  theme(axis.text.x = element_text(angle = 90,hjust = 0.5),axis.title.x=element_blank())+
  ggtitle("18S Faith's Phylogenetic Diversity carapace samples (patterns by species) (standardized resi
```

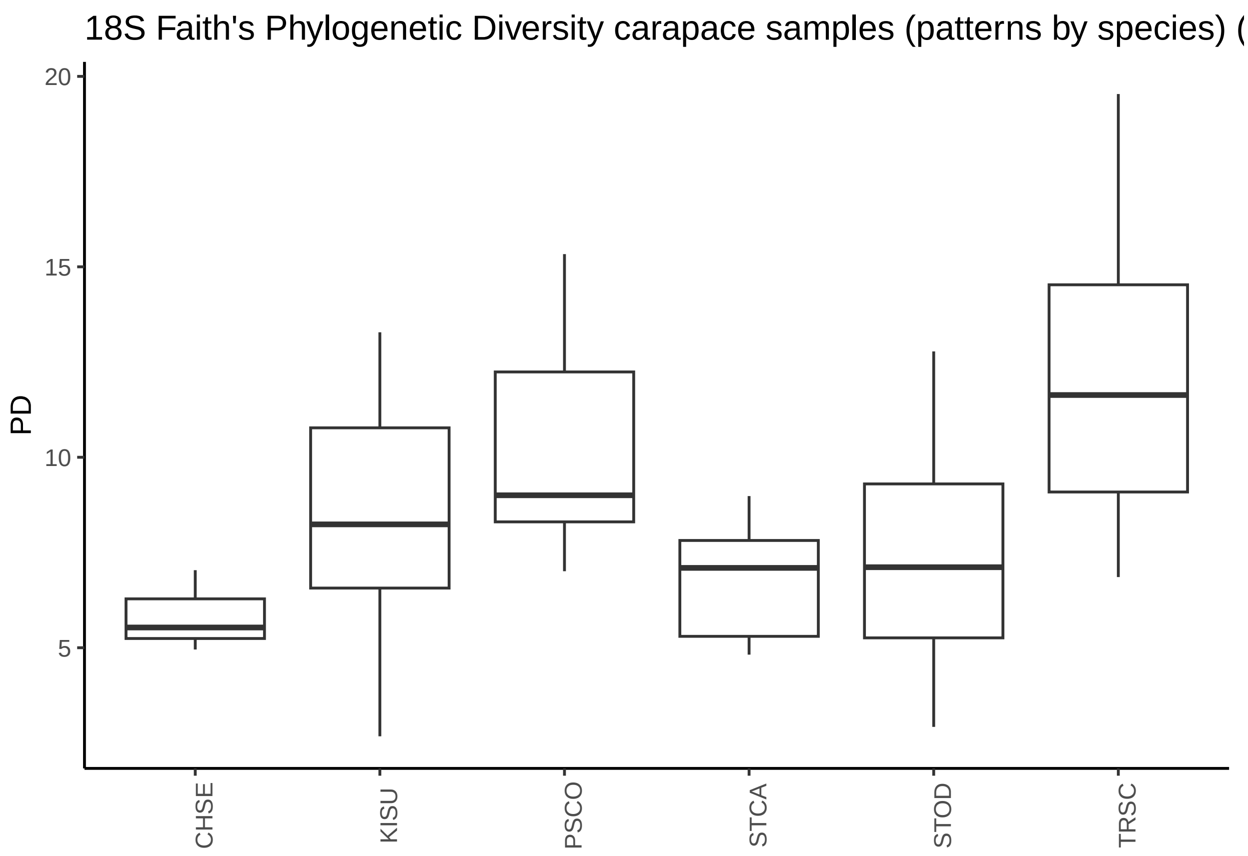


```
ggplot(carapace_observed_no_outliers,aes(x=Site,y=Shannon))+
  geom_boxplot()+
  theme_classic()+
  theme(axis.text.x = element_text(angle = 90,hjust = 0.5),axis.title.x=element_blank())+
  ggtitle("18S Shannon diversity carapace samples (patterns by site) (standardized residual outliers re
```

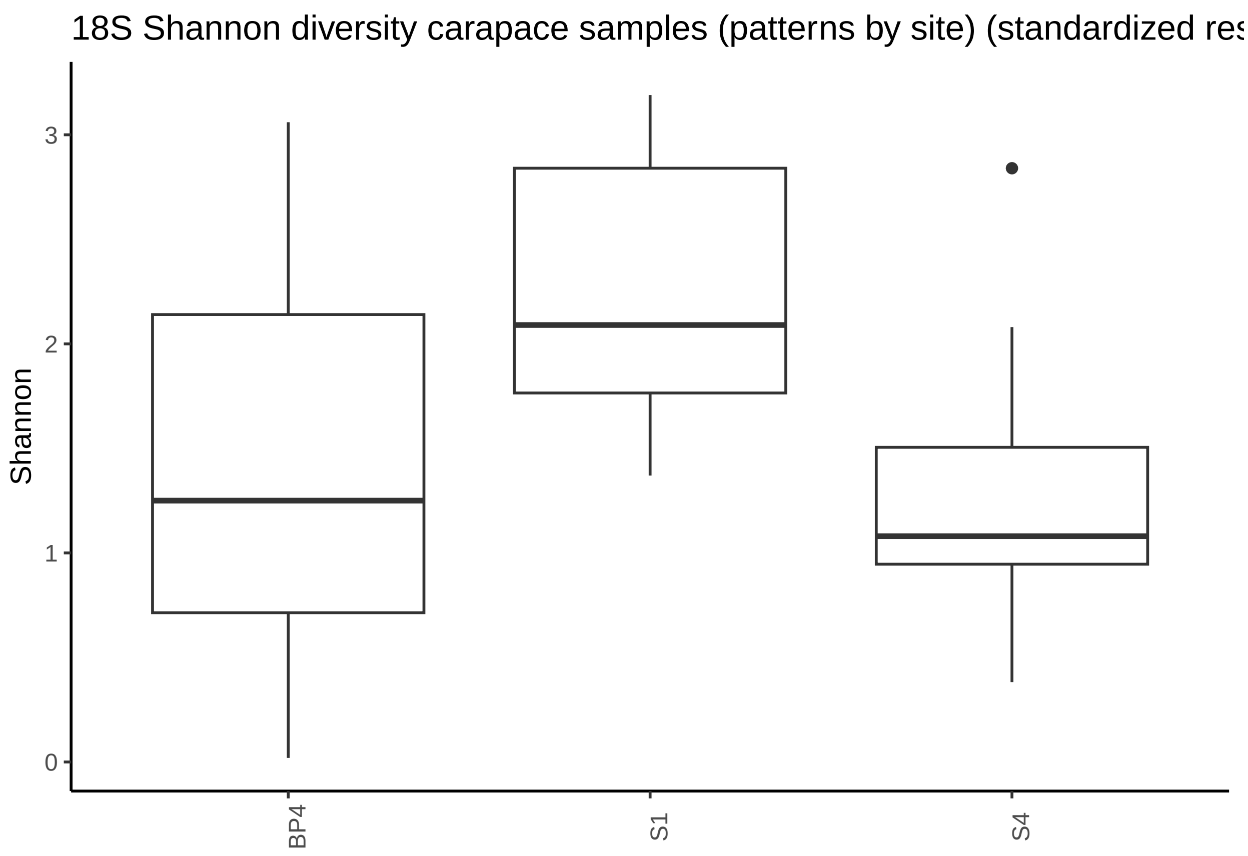


```
ggplot(carapace_shannon_no_outliers,aes(x=Site,y=Observed))+
  geom_boxplot()+
  theme_classic()+
  theme(axis.text.x = element_text(angle = 90,hjust = 0.5),axis.title.x=element_blank())+
  ggtitle("18S Observed features carapace samples (patterns by site) (standardized residual outliers rem
```

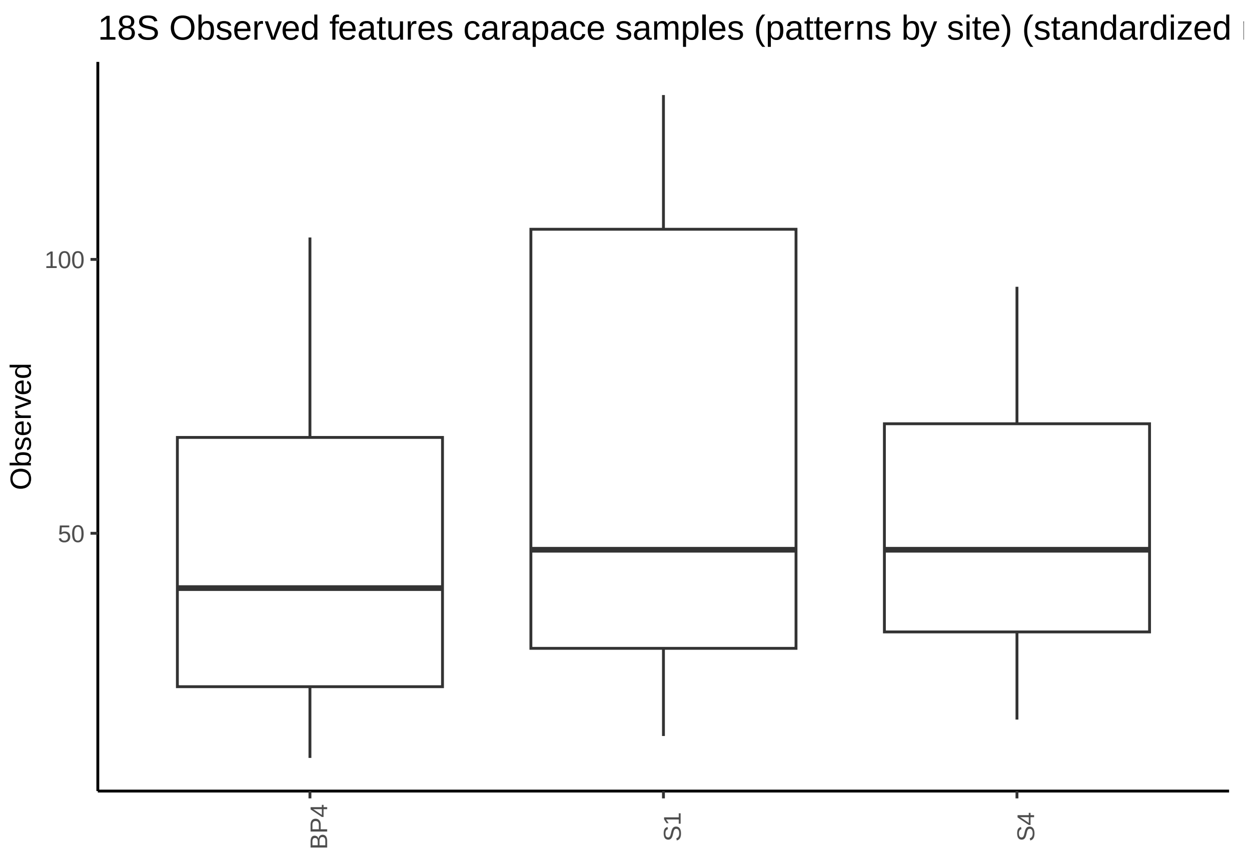


```
ggplot(carapace_PD_no_outliers,aes(x=Site,y=PD))+
  geom_boxplot()+
  theme_classic()+
  theme(axis.text.x = element_text(angle = 90,hjust = 0.5),axis.title.x=element_blank())+
  ggtitle("18S Faith's Phylogenetic Diversity carapace samples (patterns by site) (standardized residual
```

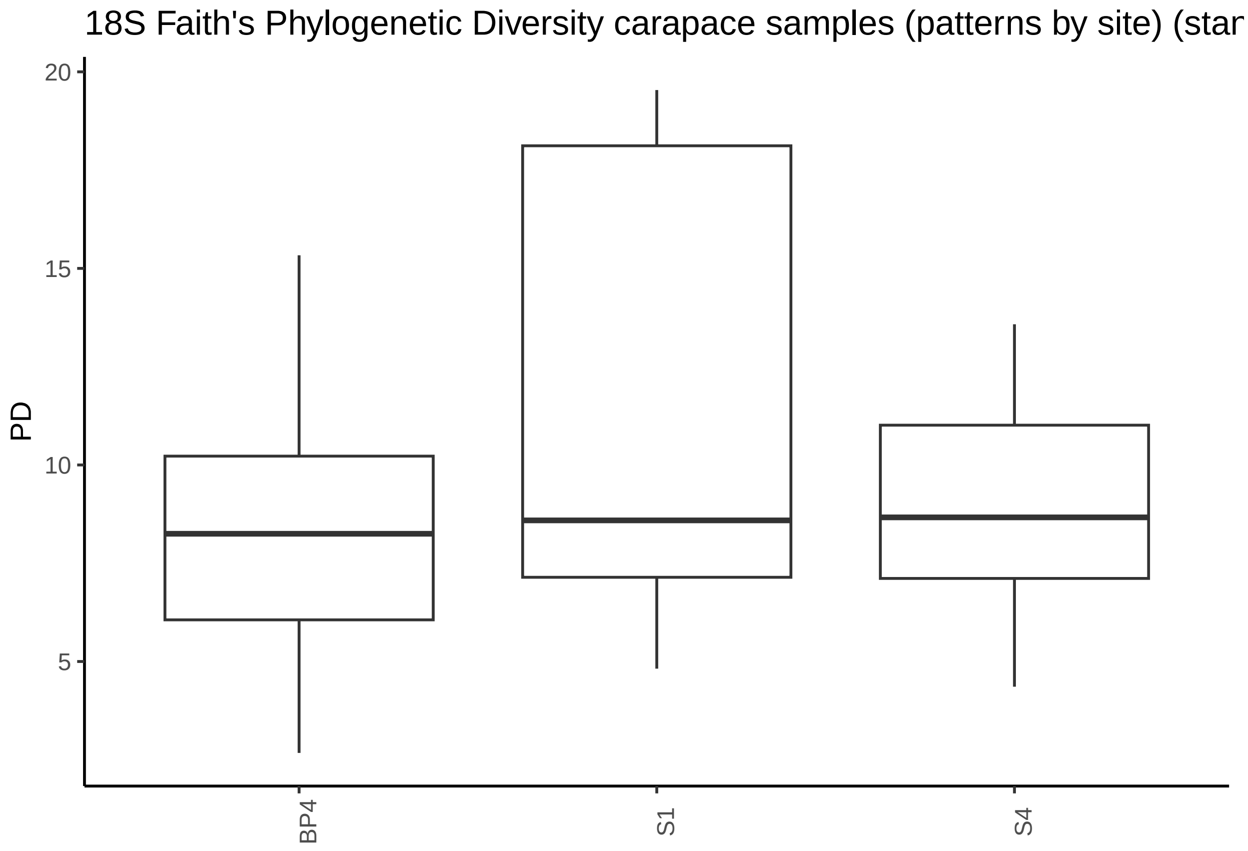

Check for effects of plastron length within each turtle species

In the below section, effect of plastron length is checked within each species, since plastron lengths are expected to differ between species and have moderate to high collinearity with turtle species in multiple regression analysis. CHSE is not included in this analysis, as the regression will not work with only three samples.

```
#Check Shannon diversity first
#check for effects in KISU
pl.shannon.KISU <- lm(Shannon~Plastron_length+Site+Sex+log10_read_depth+Year,data=KISU)

summary(pl.shannon.KISU)
```

```
##
## Call:
## lm(formula = Shannon ~ Plastron_length + Site + Sex + log10_read_depth +
##     Year, data = KISU)
##
## Residuals:
##     Min      1Q  Median      3Q     Max
## -1.2844 -0.3148 -0.0497  0.3165  0.7249
##
## Coefficients:
##                     Estimate Std. Error t value Pr(>|t|)
## (Intercept)       1421.91461  951.17685   1.495    0.173
```

```
## Plastron_length    -0.02318    0.03373  -0.687    0.511
## SiteS4             -0.74876    0.59401  -1.261    0.243
## SexM                0.02180    0.59106   0.037    0.971
## log10_read_depth    2.83046    1.99125   1.421    0.193
## Year               -0.70846    0.46963  -1.509    0.170
##
## Residual standard error: 0.703 on 8 degrees of freedom
## Multiple R-squared:  0.5283, Adjusted R-squared:  0.2336
## F-statistic: 1.792 on 5 and 8 DF,  p-value: 0.2206
```

```r
confint(pl.shannon.KISU)
```

```
##                          2.5 %       97.5 %
## (Intercept)       -771.5031473 3.615332e+03
## Plastron_length     -0.1009622 5.459981e-02
## SiteS4              -2.1185466 6.210289e-01
## SexM                -1.3411899 1.384784e+00
## log10_read_depth    -1.7613663 7.422279e+00
## Year                -1.7914169 3.744990e-01
```

```r
check_model(pl.shannon.KISU)
```

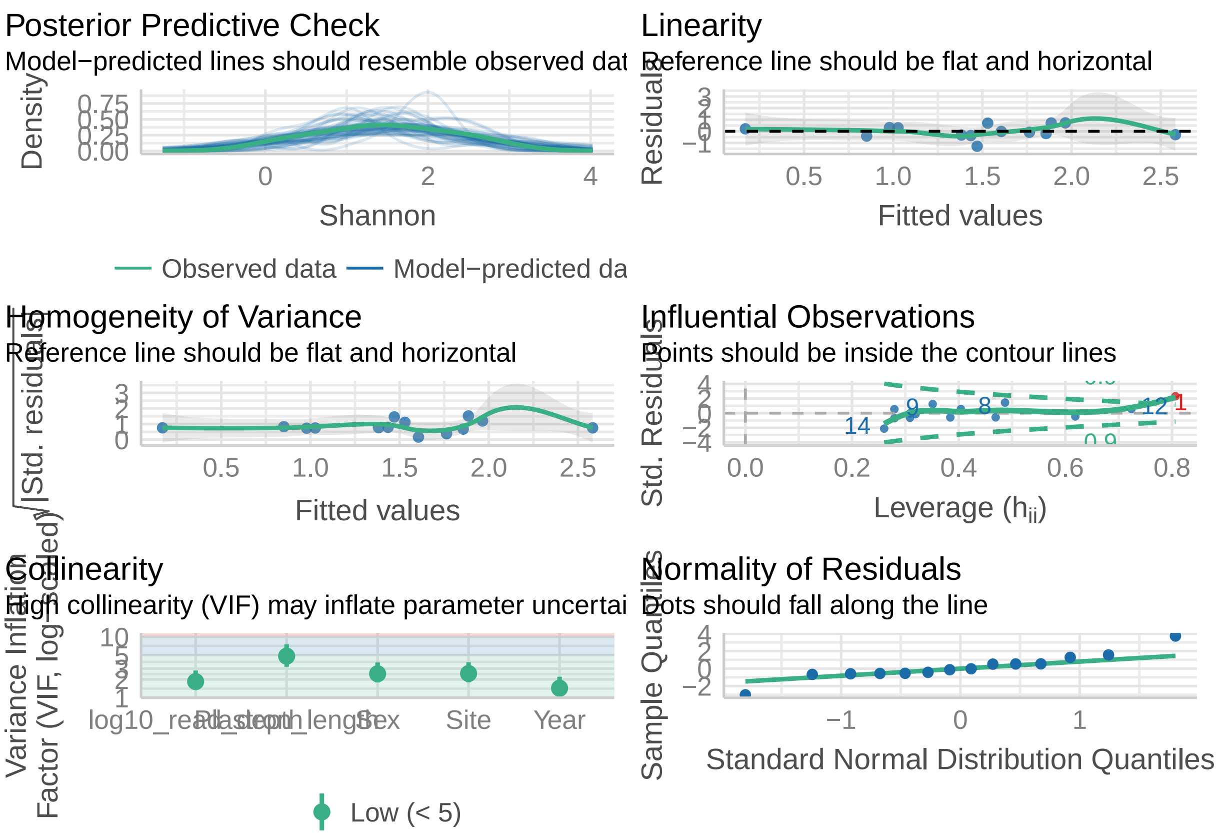


```r
plot(pl.shannon.KISU, which = 1)
```

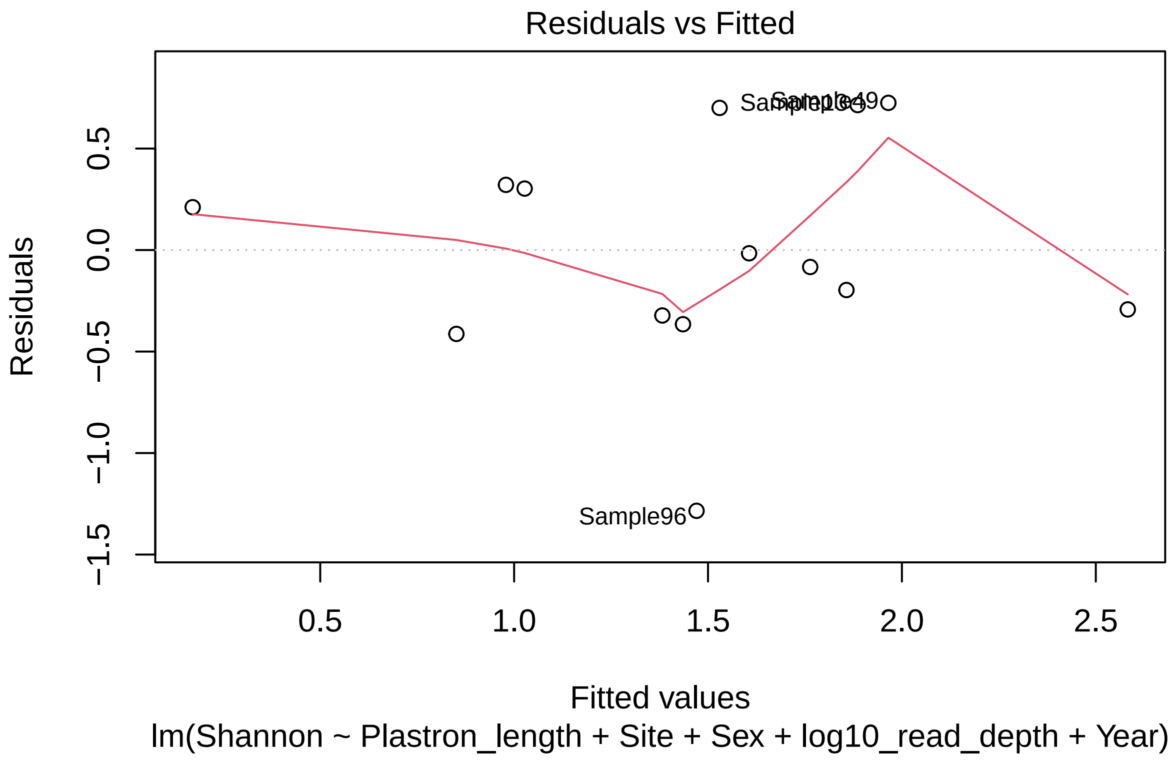


```
plot(pl.shannon.KISU, which = 2)
```

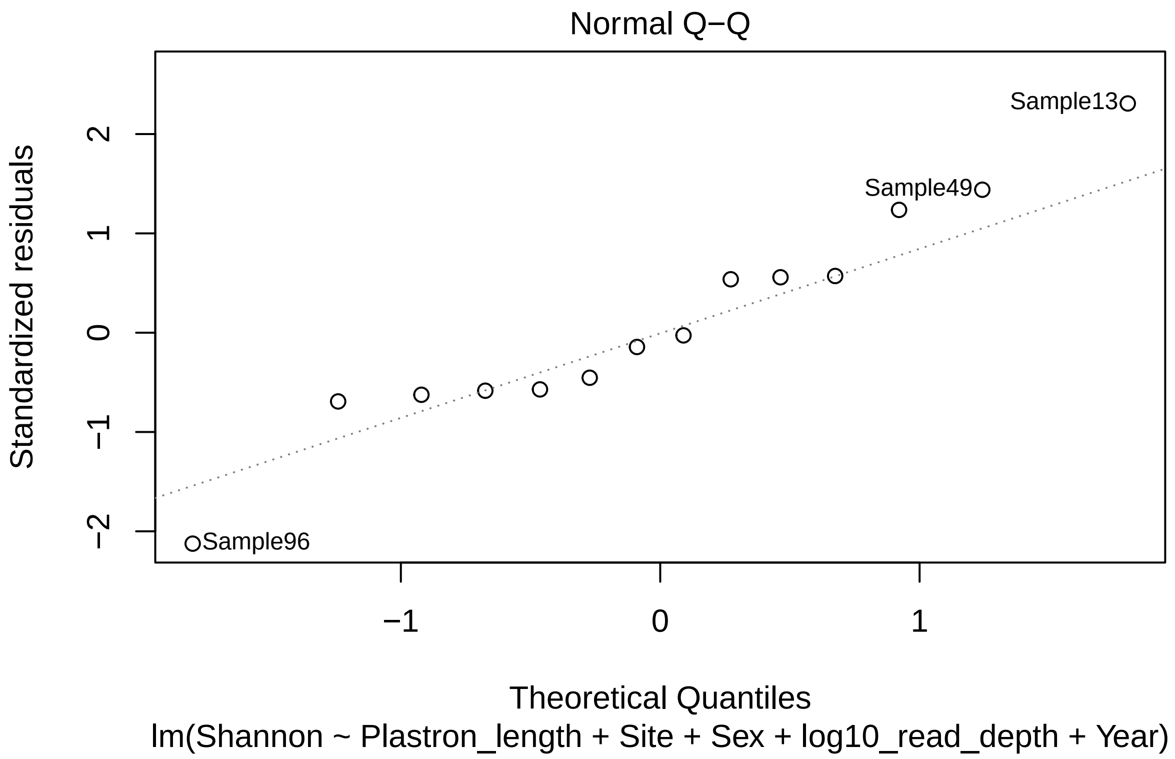


```
#check for effects in PSCO
#took out variable 'sex' from PSCO model for Shannon diversity, as sex and plastron_length were highly
pl.shannon.PSCO <- lm(Shannon~Plastron_length+log10_read_depth+Year,data=PSCO)
```

```r
summary(pl.shannon.PSCO)
```

```
##
## Call:
## lm(formula = Shannon ~ Plastron_length + log10_read_depth + Year,
##     data = PSCO)
##
## Residuals:
##     Min      1Q  Median      3Q     Max
## -1.0131 -0.2996  0.1564  0.2478  0.9916
##
## Coefficients:
##                    Estimate Std. Error t value Pr(>|t|)
## (Intercept)      -2.581e+02  1.484e+03  -0.174    0.866
## Plastron_length  -7.008e-04  3.936e-03  -0.178    0.863
## log10_read_depth  3.015e+00  2.112e+00   1.428    0.191
## Year              1.211e-01  7.303e-01   0.166    0.872
##
## Residual standard error: 0.6166 on 8 degrees of freedom
## Multiple R-squared:  0.3409, Adjusted R-squared:  0.09368
## F-statistic: 1.379 on 3 and 8 DF,  p-value: 0.3175
```

```r
confint(pl.shannon.PSCO)
```

```
##                          2.5 %       97.5 %
## (Intercept)      -3.681346e+03 3.165158e+03
## Plastron_length  -9.776478e-03 8.374794e-03
## log10_read_depth -1.854041e+00 7.884390e+00
## Year             -1.563031e+00 1.805310e+00
```

```r
check_model(pl.shannon.PSCO)
```

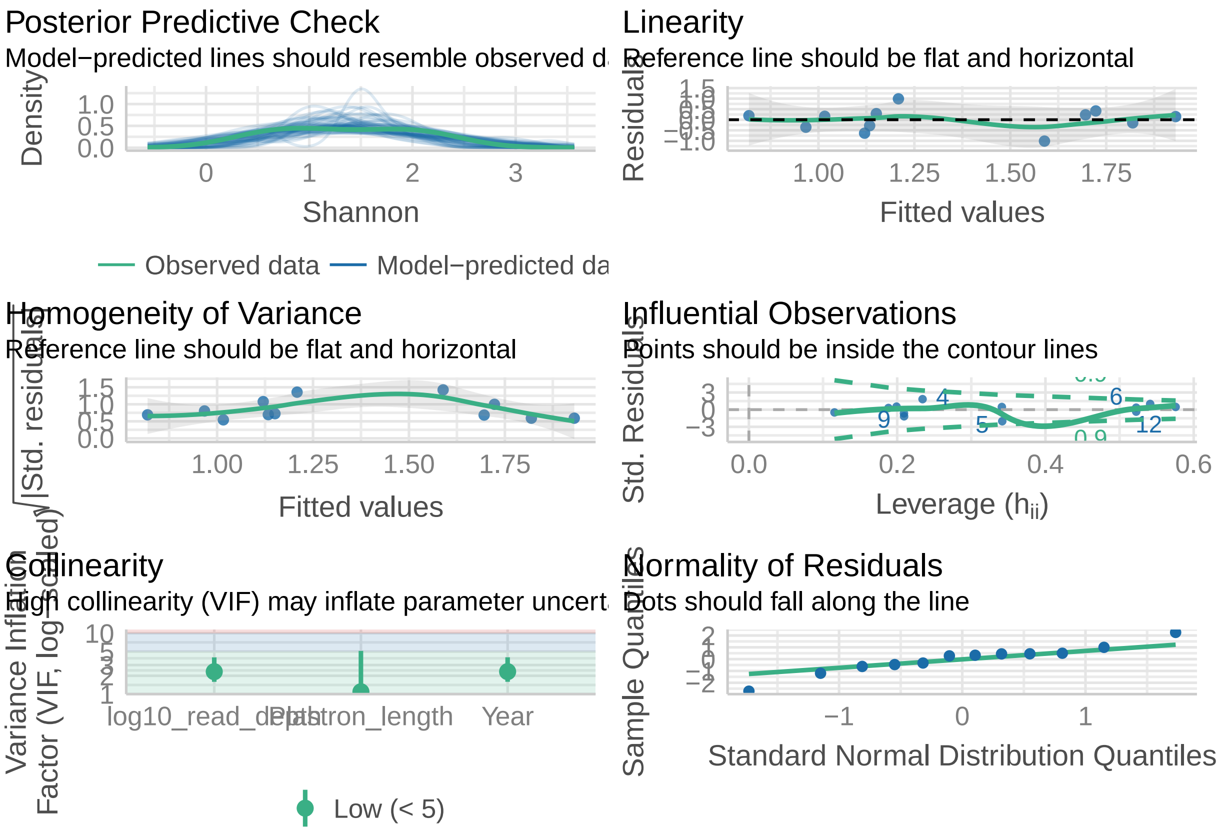


```r
plot(pl.shannon.PSCO, which = 1)
```

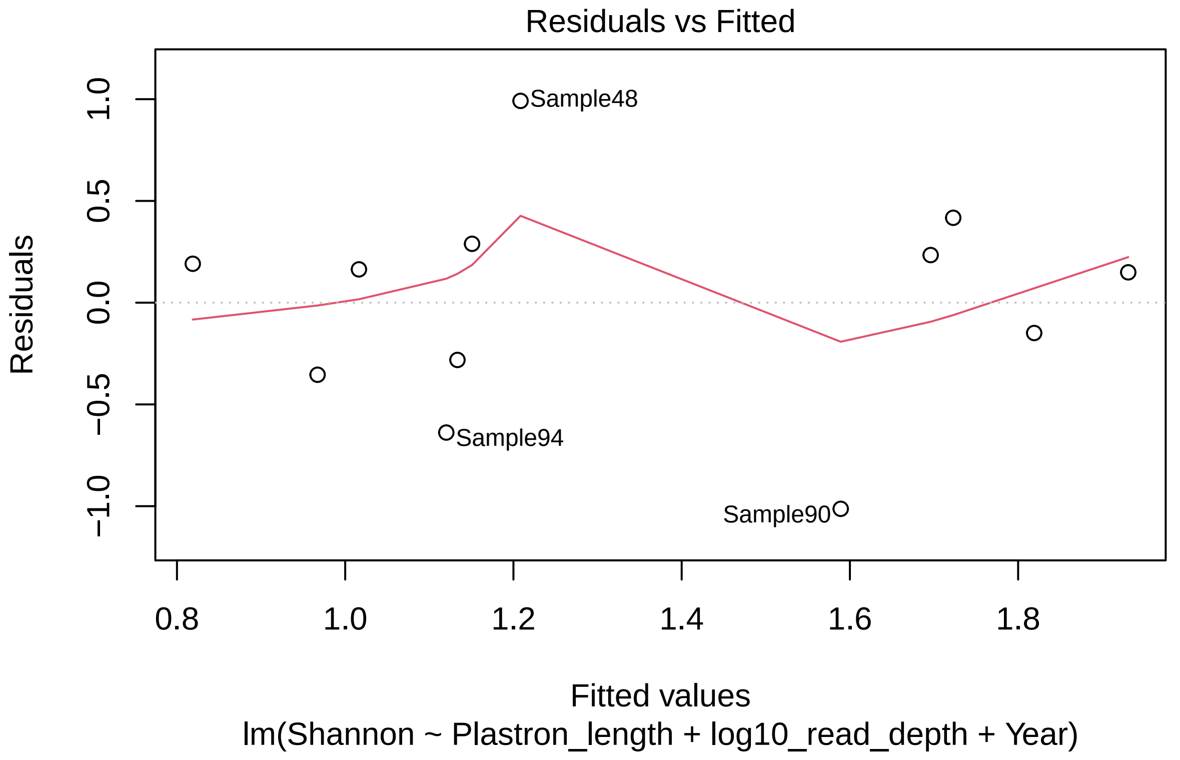

```r
plot(pl.shannon.PSCO, which = 2)
```

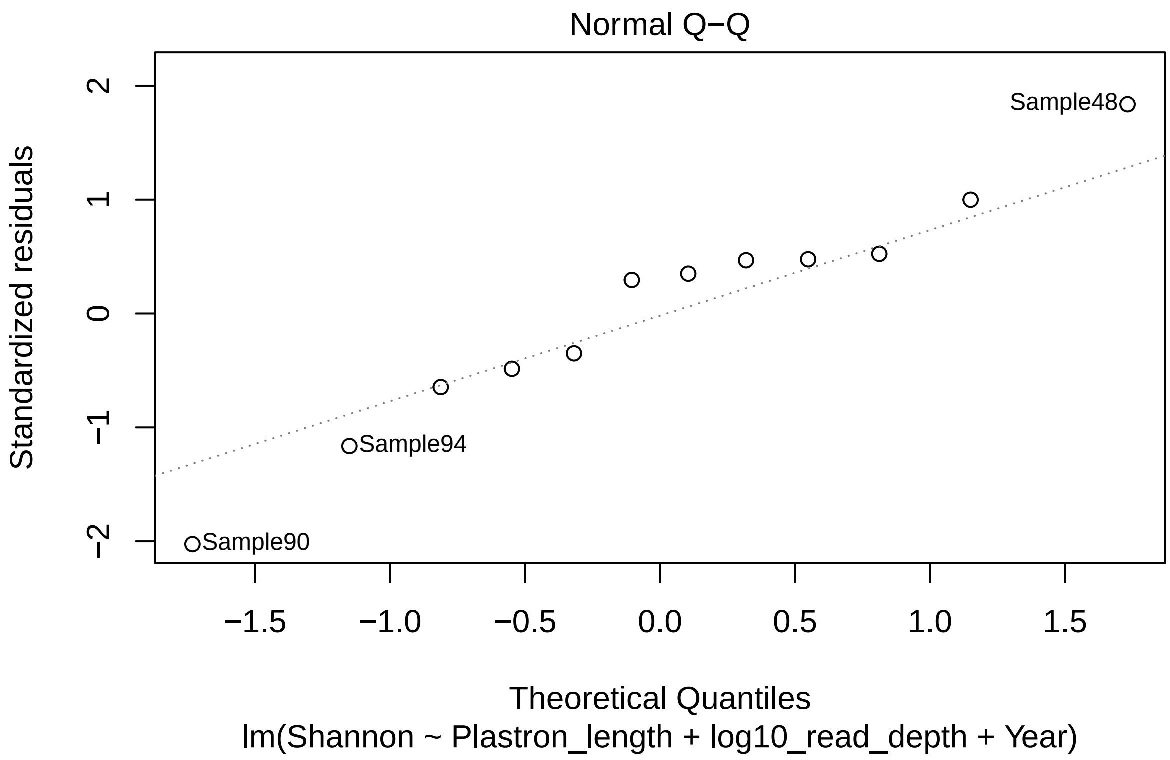


```r
#check for effects in STCA
pl.shannon.STCA <- lm(Shannon~Plastron_length+Sex+log10_read_depth+Year,data=STCA)

summary(pl.shannon.STCA)
```

```
##
## Call:
## lm(formula = Shannon ~ Plastron_length + Sex + log10_read_depth +
##     Year, data = STCA)
##
## Residuals:
##   Sample55   Sample62   Sample63   Sample64   Sample69   Sample70   Sample71
## -2.032e-01 -2.509e-02  7.332e-01 -8.260e-01 -1.041e-16  2.311e-01 -1.390e-01
##   Sample72
##  2.289e-01
##
## Coefficients: (1 not defined because of singularities)
##                  Estimate Std. Error t value Pr(>|t|)
## (Intercept)       1.01144    8.57524   0.118    0.912
## Plastron_length  -0.01123    0.02440  -0.460    0.669
## SexM             -0.32082    0.65725  -0.488    0.651
## log10_read_depth  0.40009    1.90943   0.210    0.844
## Year                   NA         NA      NA       NA
##
## Residual standard error: 0.5888 on 4 degrees of freedom
## Multiple R-squared:  0.115,  Adjusted R-squared:  -0.5487
## F-statistic: 0.1733 on 3 and 4 DF,  p-value: 0.9092
```

```r
confint(pl.shannon.STCA)
```

```
##                          2.5 %      97.5 %
## (Intercept)       -22.79723814 24.82012061
## Plastron_length    -0.07897269  0.05651785
## SexM               -2.14563055  1.50399368
## log10_read_depth   -4.90134896  5.70151982
## Year                        NA          NA
```

```r
check_model(pl.shannon.STCA)
```

```
## Model matrix is rank deficient. VIFs may not be sensible.
```

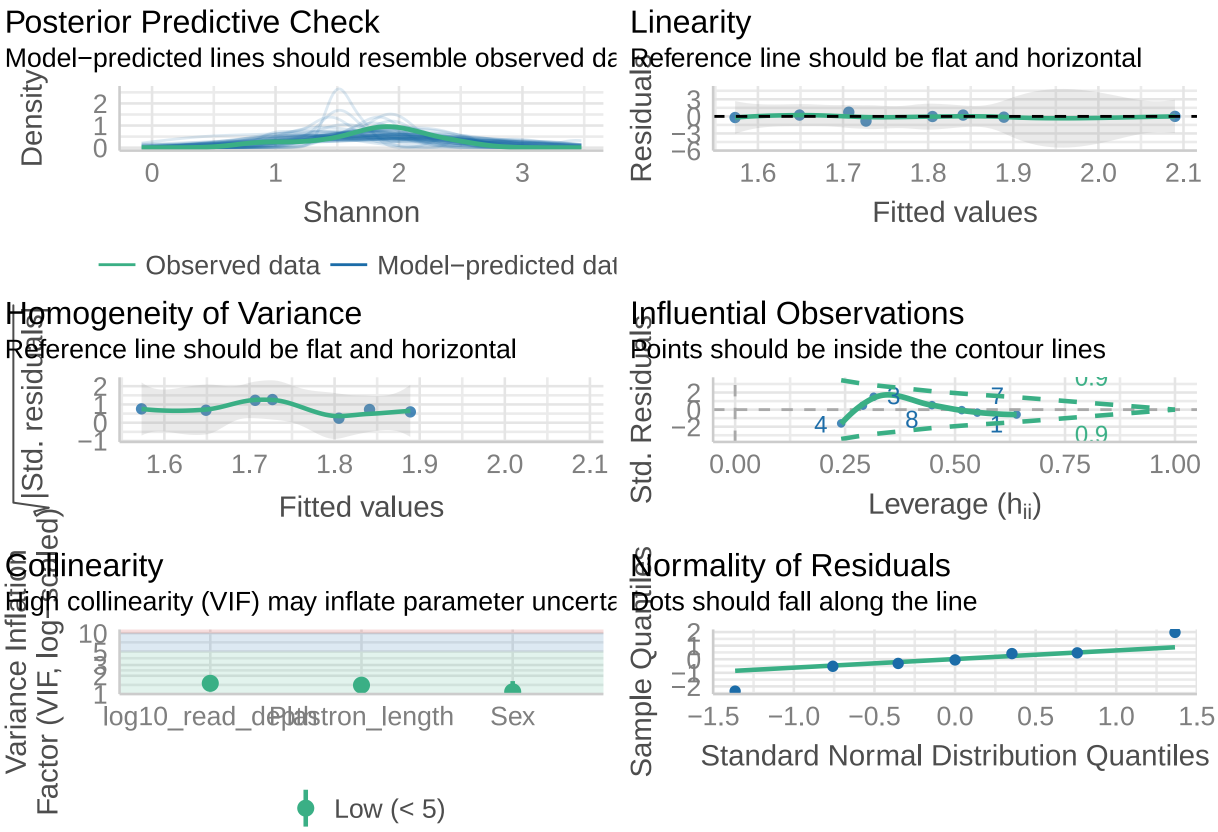


```r
plot(pl.shannon.STCA, which = 1)
```

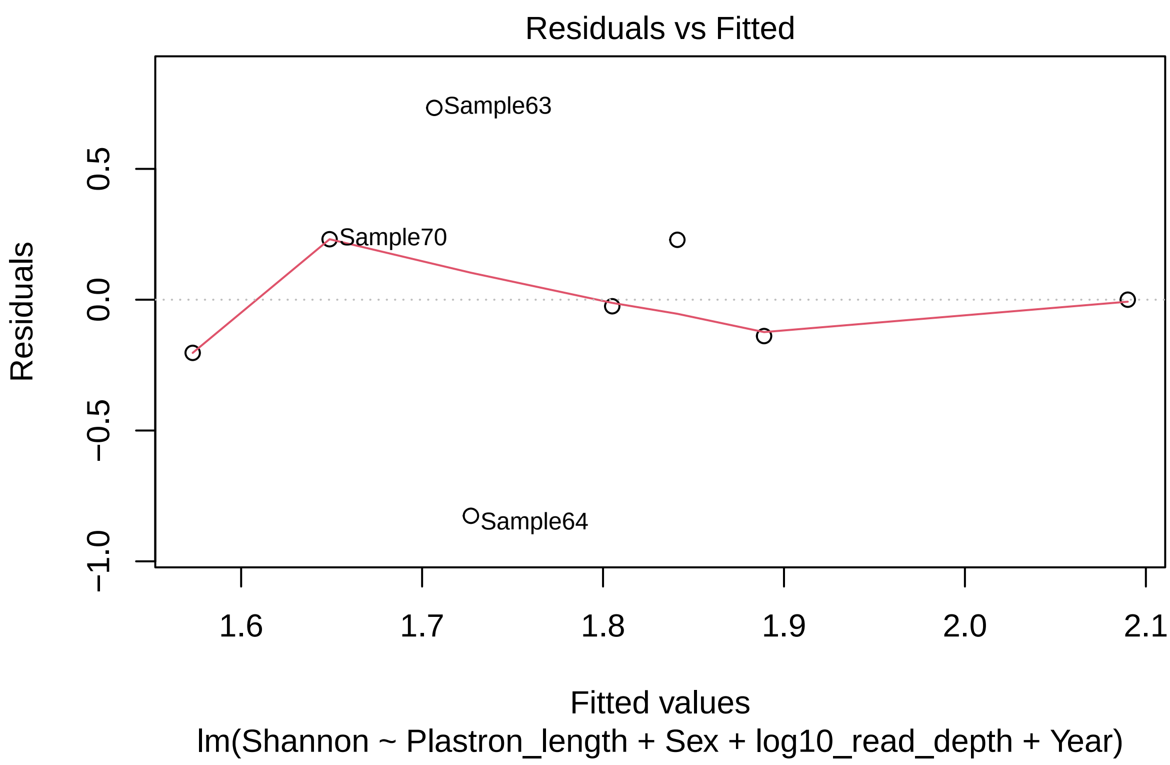


```
plot(pl.shannon.STCA, which = 2)
```

```
## Warning: not plotting observations with leverage one:
##   5
```

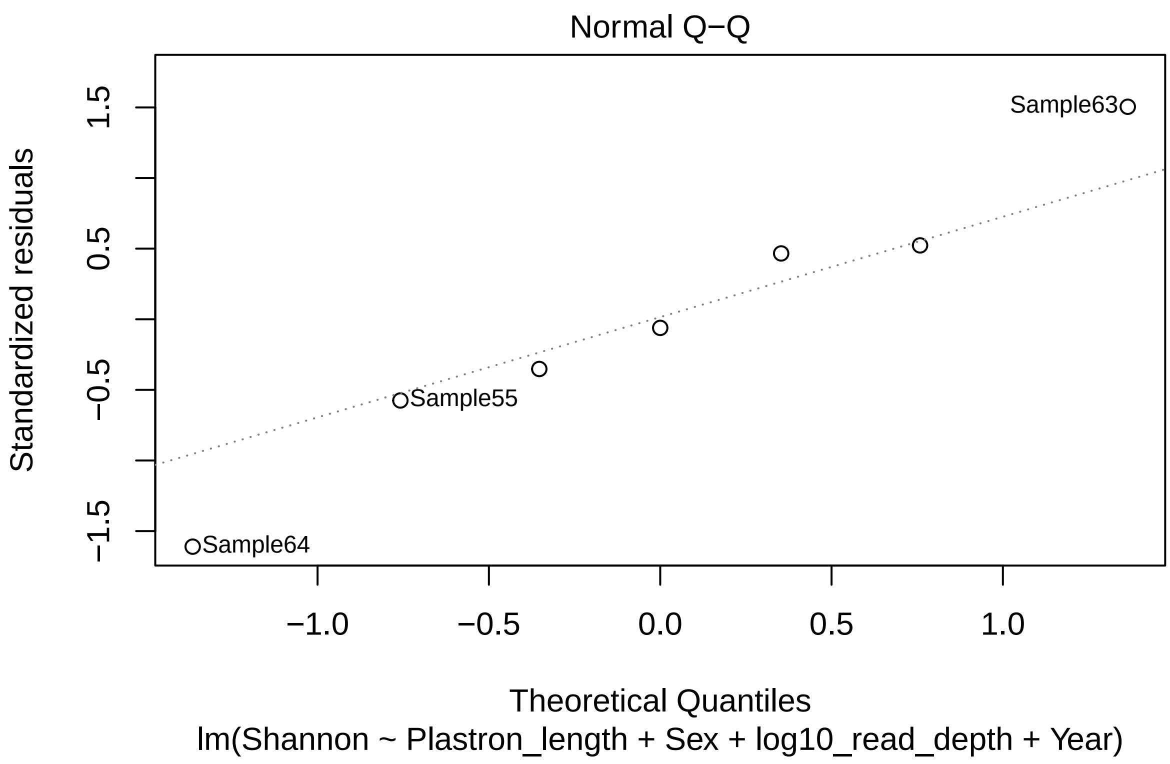

```r
#check for effects in STOD
pl.shannon.STOD <- lm(Shannon~Plastron_length+Sex+log10_read_depth+Year,data=STOD)

summary(pl.shannon.STOD)
```

```
##
## Call:
## lm(formula = Shannon ~ Plastron_length + Sex + log10_read_depth +
##     Year, data = STOD)
##
## Residuals:
##      Min       1Q   Median       3Q      Max
## -0.79969 -0.47023 -0.00615  0.34791  1.10325
##
## Coefficients:
##                    Estimate Std. Error t value Pr(>|t|)
## (Intercept)      -120.15185  554.21913  -0.217   0.8307
## Plastron_length    -0.03872    0.02023  -1.914   0.0708 .
## SexJ               -0.77232    0.84594  -0.913   0.3727
## SexM               -0.01577    0.29592  -0.053   0.9580
## log10_read_depth    3.07862    1.38693   2.220   0.0388 *
## Year                0.05363    0.27309   0.196   0.8464
## ---
## Signif. codes:  0 '***' 0.001 '**' 0.01 '*' 0.05 '.' 0.1 ' ' 1
##
## Residual standard error: 0.5644 on 19 degrees of freedom
## Multiple R-squared:  0.3428, Adjusted R-squared:  0.1699
## F-statistic: 1.983 on 5 and 19 DF,  p-value: 0.1276
```

```r
confint(pl.shannon.STOD)
```

```
##                          2.5 %       97.5 %
## (Intercept)      -1.280146e+03 1.039842e+03
## Plastron_length  -8.105858e-02 3.627823e-03
## SexJ             -2.542887e+00 9.982551e-01
## SexM             -6.351353e-01 6.035870e-01
## log10_read_depth  1.757507e-01 5.981491e+00
## Year             -5.179623e-01 6.252155e-01
```

```r
check_model(pl.shannon.STOD)
```

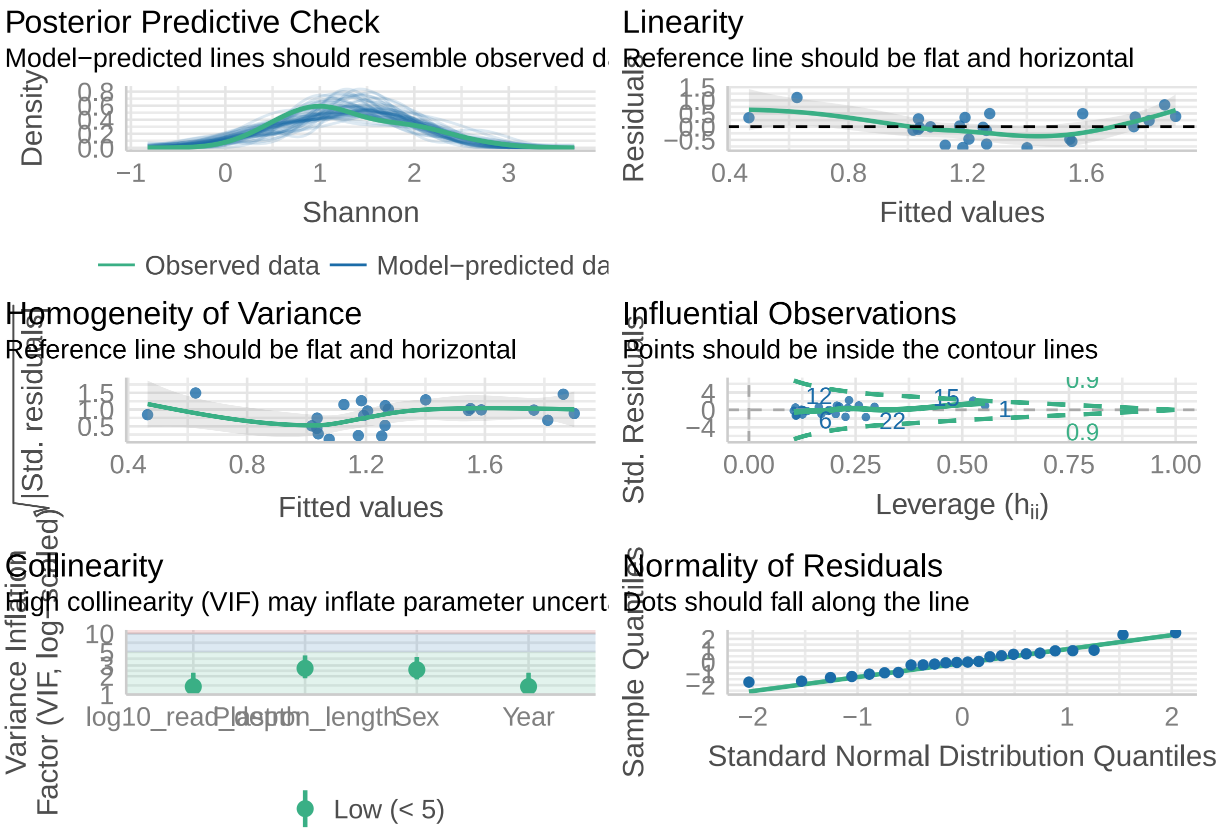


```r
plot(pl.shannon.STOD, which = 1)
```

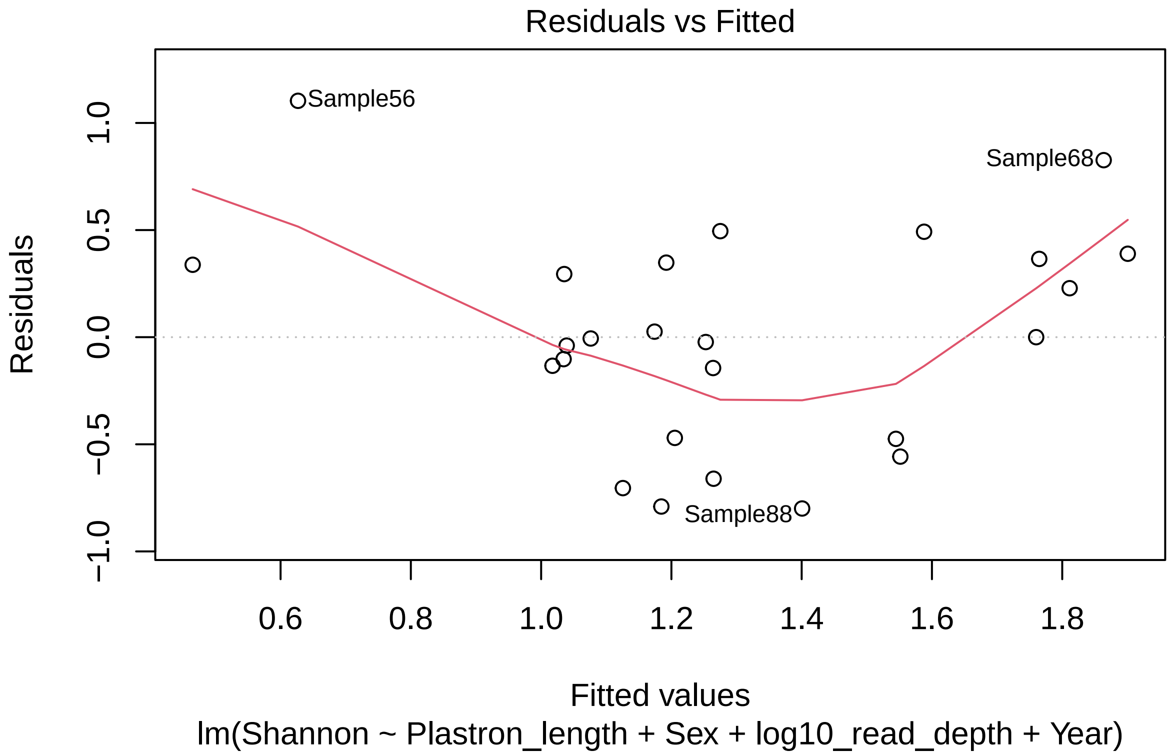

```r
plot(pl.shannon.STOD, which = 2)
```

```
## Warning: not plotting observations with leverage one:
##   3
```

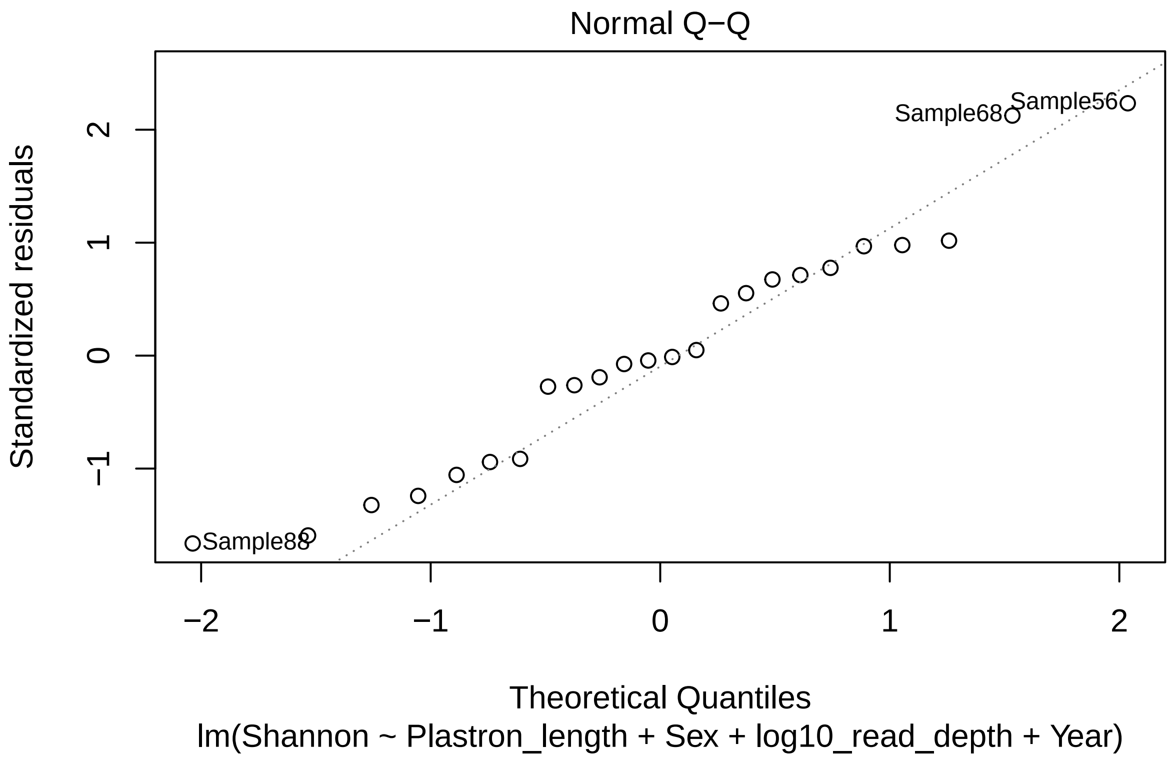


```r
#check for effects in TRSC
pl.shannon.TRSC <- lm(Shannon~Plastron_length+Sex+log10_read_depth+Year,data=TRSC)

summary(pl.shannon.TRSC)
```

```
##
## Call:
## lm(formula = Shannon ~ Plastron_length + Sex + log10_read_depth +
##     Year, data = TRSC)
##
## Residuals:
##     Min      1Q  Median      3Q     Max
## -1.6424 -0.4614 -0.0078  0.6218  1.1551
##
## Coefficients:
##                   Estimate Std. Error t value Pr(>|t|)
## (Intercept)       1.669e+03  1.062e+03   1.572    0.136
## Plastron_length  -3.072e-03  9.897e-03  -0.310    0.760
## SexJ              6.852e-01  1.290e+00   0.531    0.603
## SexM              7.734e-01  4.977e-01   1.554    0.140
## log10_read_depth  3.488e-01  2.859e+00   0.122    0.904
## Year             -8.256e-01  5.260e-01  -1.570    0.136
##
```

```
## Residual standard error: 0.9518 on 16 degrees of freedom
## Multiple R-squared:  0.2627, Adjusted R-squared:  0.03236
## F-statistic:  1.14 on 5 and 16 DF,  p-value: 0.3795
```

```
confint(pl.shannon.TRSC)
```

```
##                          2.5 %       97.5 %
## (Intercept)       -581.76884386 3.920474e+03
## Plastron_length     -0.02405286 1.790822e-02
## SexJ                -2.04925242 3.419738e+00
## SexM                -0.28168768 1.828498e+00
## log10_read_depth    -5.71106780 6.408582e+00
## Year                -1.94063413 2.894381e-01
```

```
check_model(pl.shannon.TRSC)
```

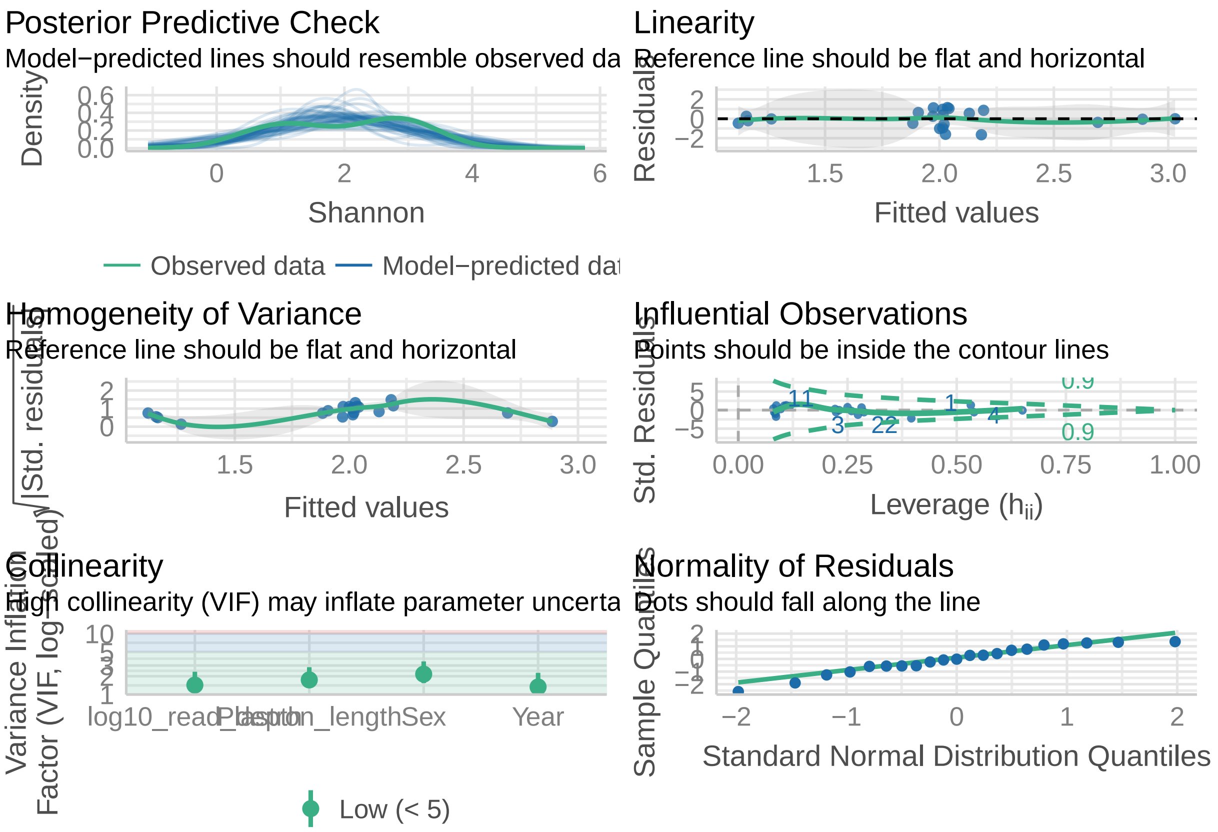


```
plot(pl.shannon.TRSC, which = 1)
```

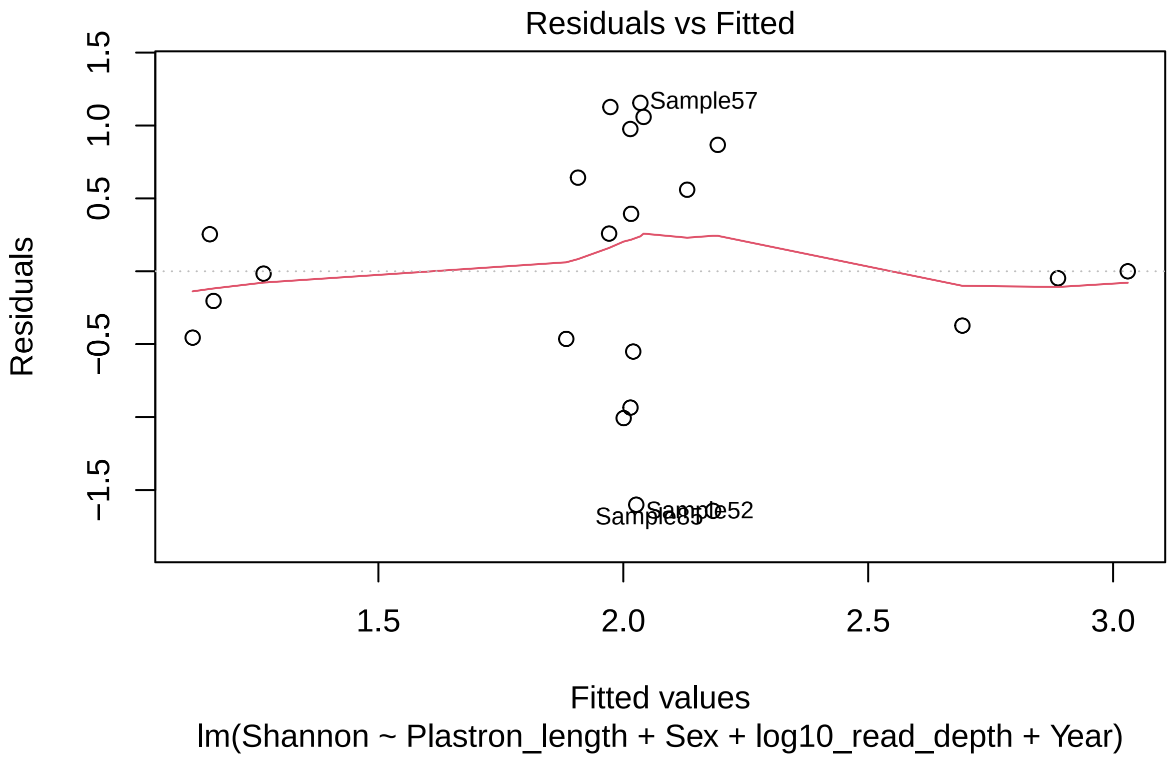


```r
plot(pl.shannon.TRSC, which = 2)
```

```
## Warning: not plotting observations with leverage one:
##   6
```

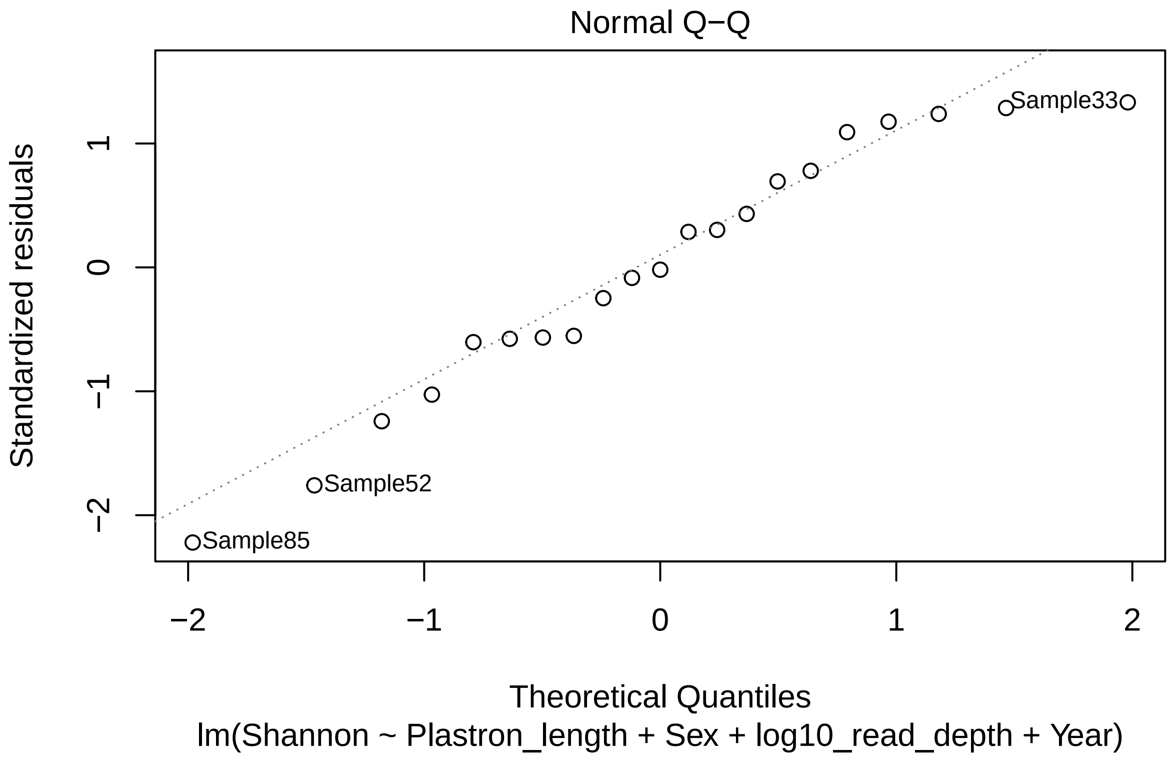

```r
#Check observed counts
#check for effects in KISU
pl.Observed.KISU <- lm(Observed~Plastron_length+Site+Sex+log10_read_depth+Year,data=KISU)

summary(pl.Observed.KISU)
```

```
##
## Call:
## lm(formula = Observed ~ Plastron_length + Site + Sex + log10_read_depth +
##     Year, data = KISU)
##
## Residuals:
##      Min       1Q   Median       3Q      Max
## -15.8657  -8.0994   0.5142   6.4207  14.4910
##
## Coefficients:
##                    Estimate Std. Error t value Pr(>|t|)
## (Intercept)       2.665e+04  1.631e+04   1.634  0.14093
## Plastron_length  -2.389e-02  5.784e-01  -0.041  0.96806
## SiteS4           -2.403e+01  1.019e+01  -2.359  0.04601 *
## SexM              1.847e+00  1.014e+01   0.182  0.85996
## log10_read_depth  1.745e+02  3.415e+01   5.109  0.00092 ***
## Year             -1.358e+01  8.054e+00  -1.686  0.13036
## ---
## Signif. codes:  0 '***' 0.001 '**' 0.01 '*' 0.05 '.' 0.1 ' ' 1
##
## Residual standard error: 12.06 on 8 degrees of freedom
## Multiple R-squared:  0.8609, Adjusted R-squared:  0.7739
## F-statistic: 9.899 on 5 and 8 DF,  p-value: 0.002835
```

```r
confint(pl.Observed.KISU)
```

```
##                          2.5 %        97.5 %
## (Intercept)      -10963.628380 64266.9720066
## Plastron_length      -1.357774     1.3099864
## SiteS4              -47.524437    -0.5429819
## SexM                -21.527301    25.2208973
## log10_read_depth     95.710143   253.2020672
## Year                -32.147150     4.9965167
```

```r
check_model(pl.Observed.KISU)
```

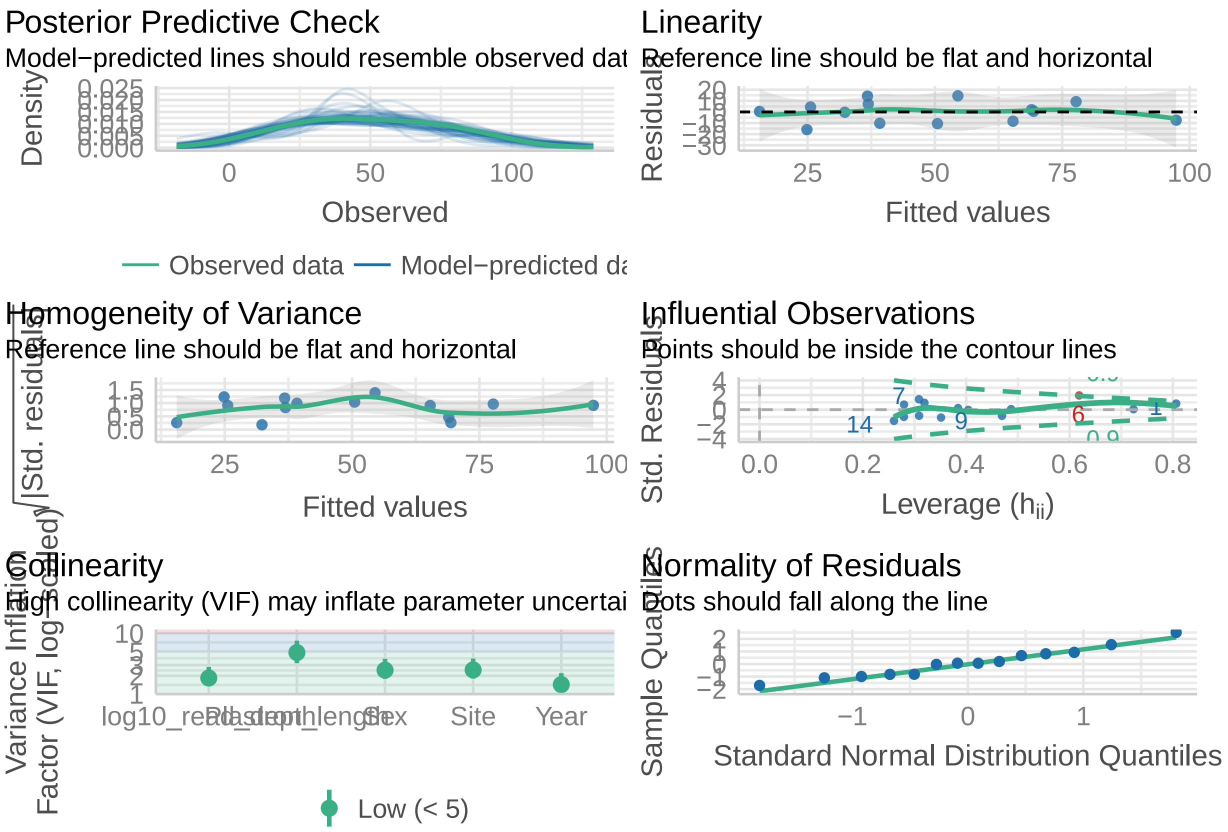


```r
plot(pl.Observed.KISU, which = 1)
```

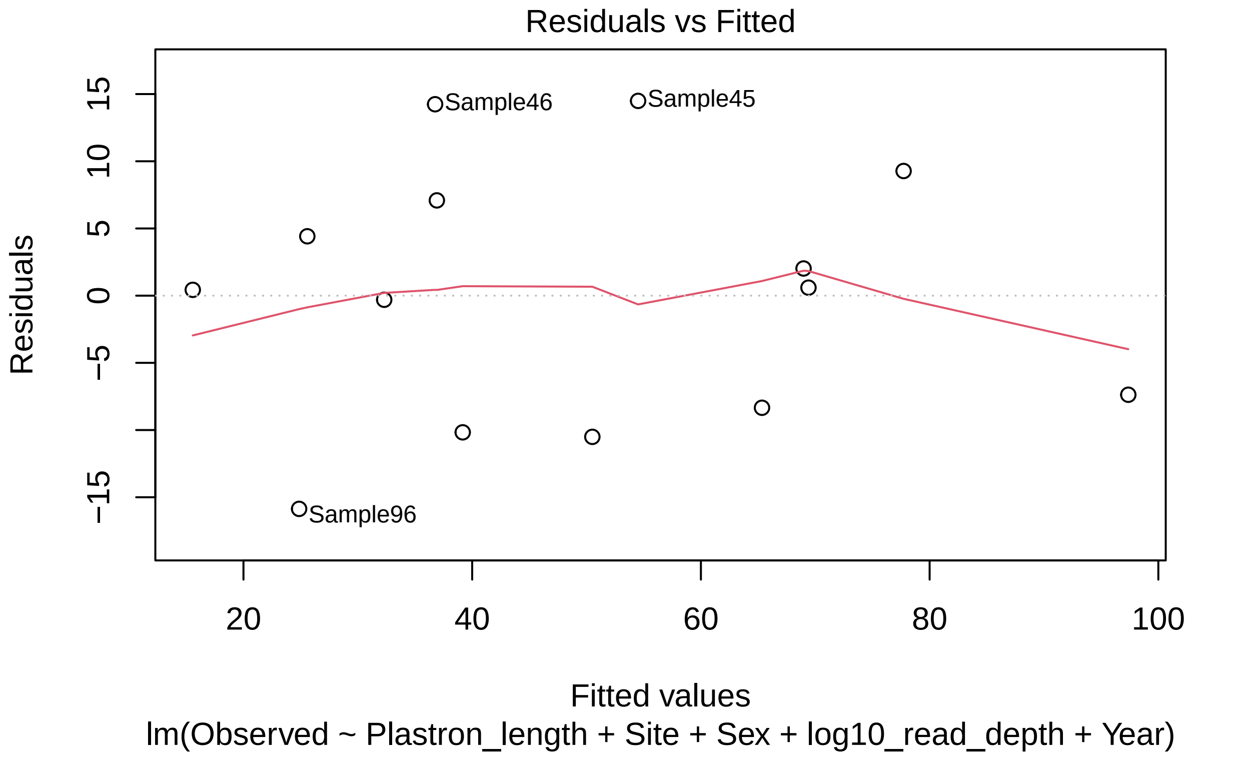

```r
plot(pl.Observed.KISU, which = 2)
```

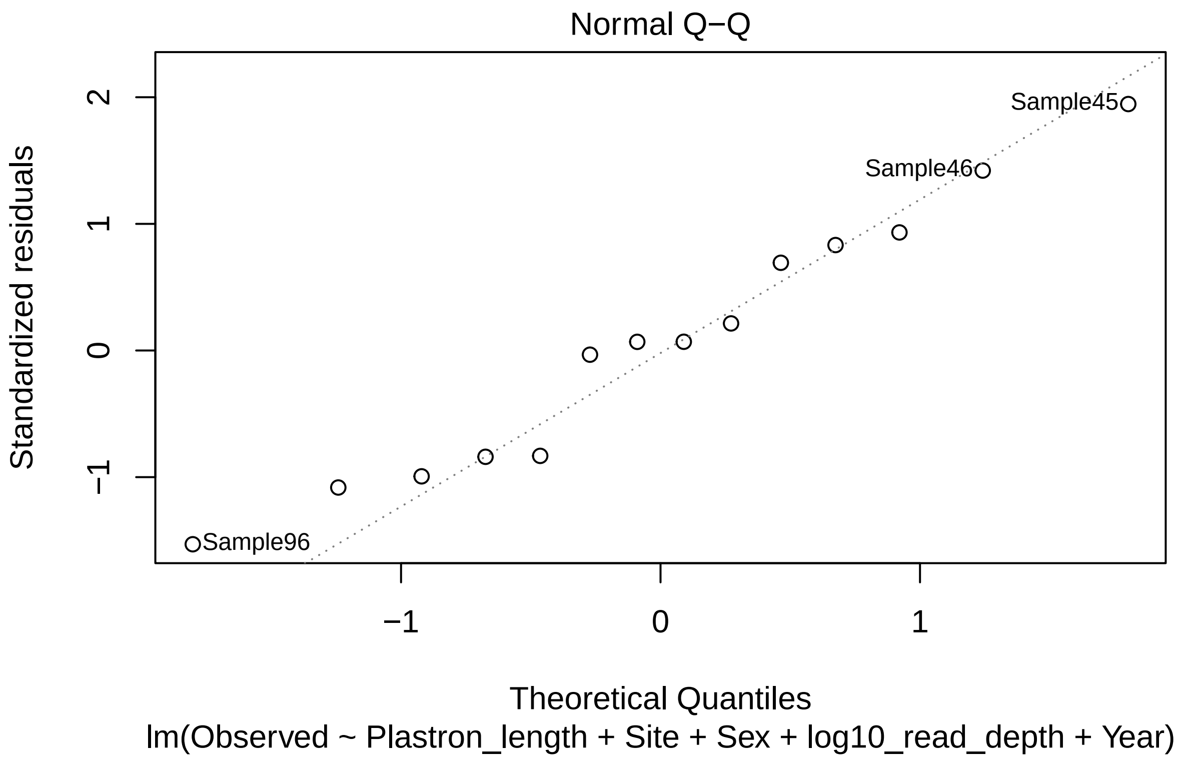


```r
#check for effects in PSCO
#took out variable 'sex' from PSCO model for observed features, as sex and plastron_length were highly
pl.Observed.PSCO <- lm(Observed~Plastron_length+log10_read_depth+Year,data=PSCO)

summary(pl.Observed.PSCO)
```

```
##
## Call:
## lm(formula = Observed ~ Plastron_length + log10_read_depth +
##     Year, data = PSCO)
##
## Residuals:
##    Min      1Q Median      3Q     Max
## -24.80 -10.48   0.00   7.91   32.09
##
## Coefficients:
##                   Estimate Std. Error t value Pr(>|t|)
## (Intercept)     36823.1481 44226.6406   0.833   0.4292
## Plastron_length     0.1036     0.1173   0.883   0.4028
## log10_read_depth  117.8906    62.9077   1.874   0.0978 .
## Year              -18.4721    21.7586  -0.849   0.4206
## ---
## Signif. codes:  0 '***' 0.001 '**' 0.01 '*' 0.05 '.' 0.1 ' ' 1
##
## Residual standard error: 18.37 on 8 degrees of freedom
## Multiple R-squared:  0.6655, Adjusted R-squared:   0.54
## F-statistic: 5.305 on 3 and 8 DF,  p-value: 0.02636
```

```r
confint(pl.Observed.PSCO)
```

```
##                          2.5 %       97.5 %
## (Intercept)       -6.516367e+04 1.388100e+05
## Plastron_length   -1.667973e-01 3.739723e-01
## log10_read_depth  -2.717495e+01 2.629561e+02
## Year              -6.864752e+01 3.170339e+01
```

```r
check_model(pl.Observed.PSCO)
```

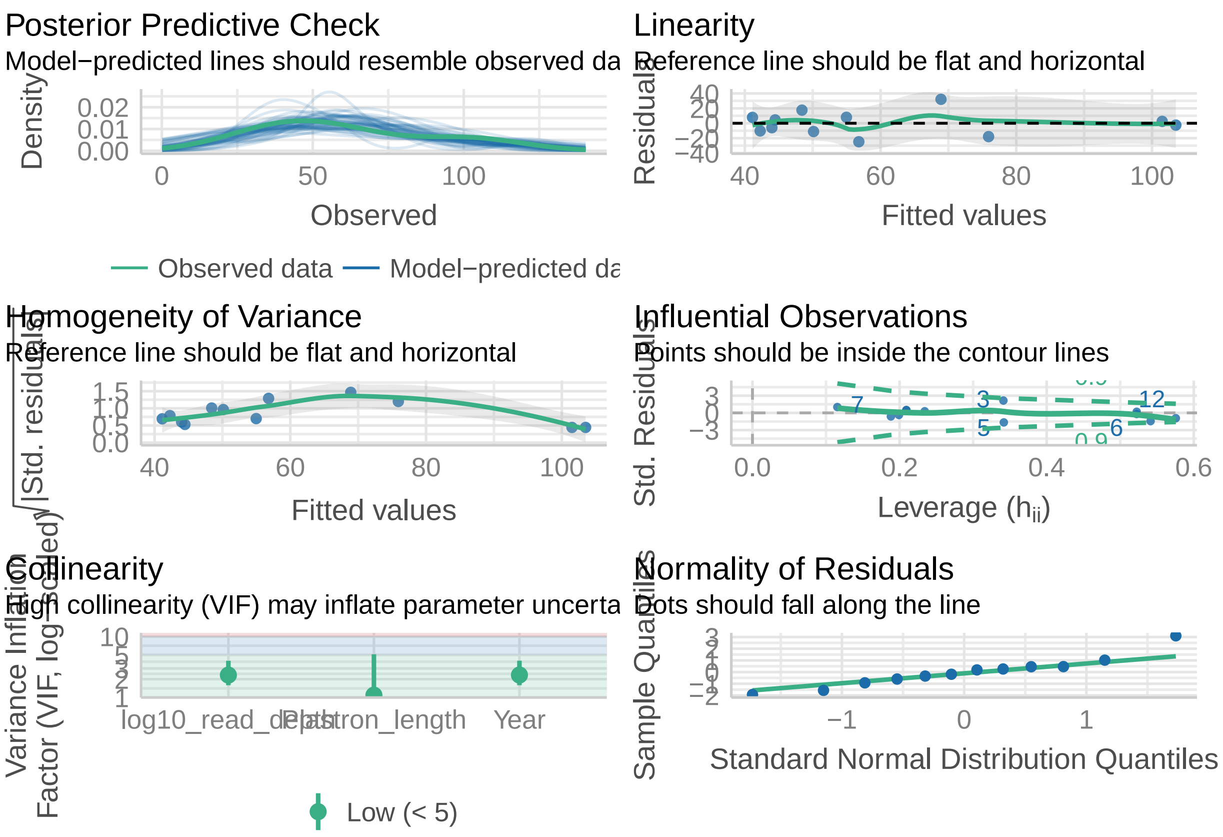


```r
plot(pl.Observed.PSCO, which = 1)
```

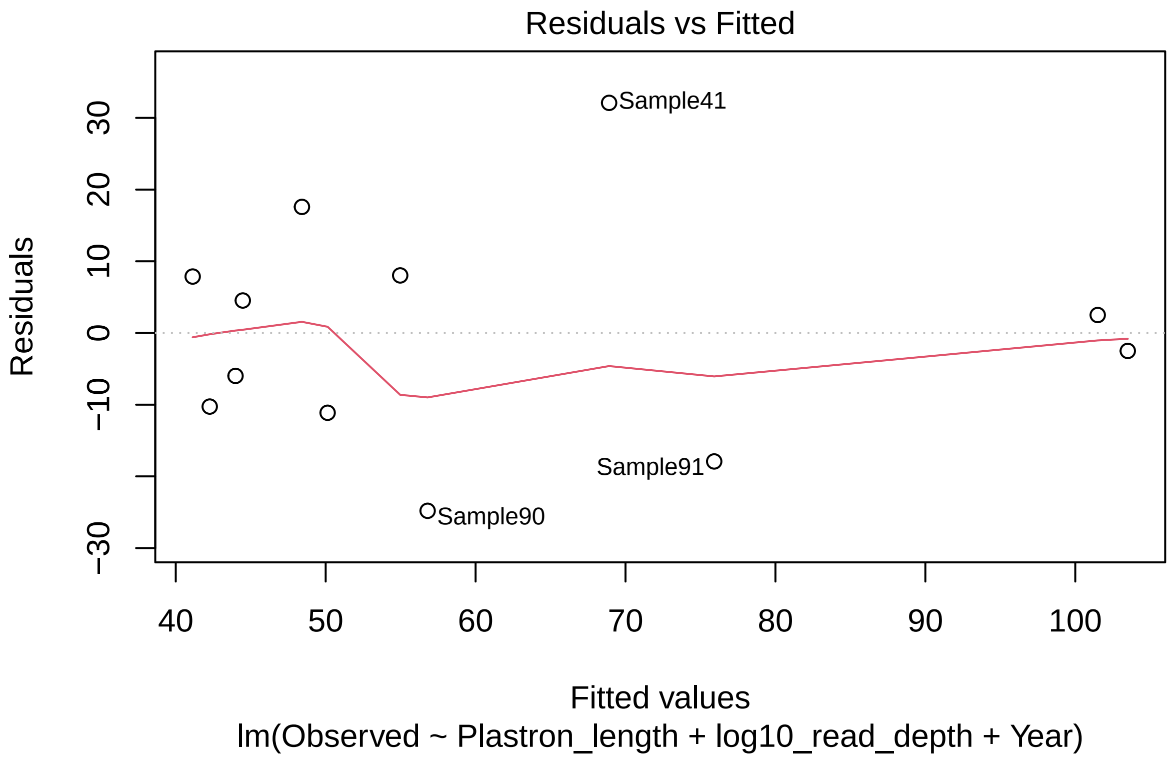


```
plot(pl.Observed.PSCO, which = 2)
```

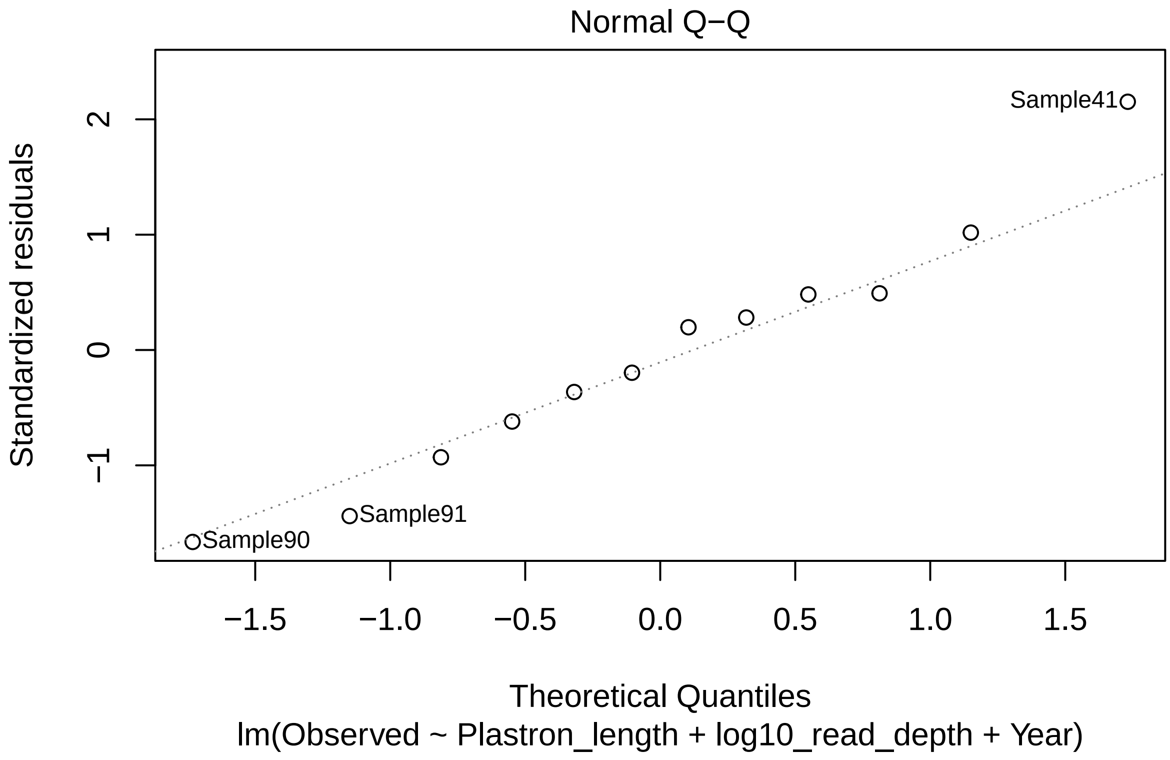


```
#check for effects in STCA
pl.Observed.STCA <- lm(Observed~Plastron_length+Sex+log10_read_depth+Year,data=STCA)
```

```r
summary(pl.Observed.STCA)
```

```
##
## Call:
## lm(formula = Observed ~ Plastron_length + Sex + log10_read_depth +
##     Year, data = STCA)
##
## Residuals:
##   Sample55   Sample62   Sample63   Sample64   Sample69   Sample70   Sample71
## -2.265e+00 -6.317e-01  1.708e+01 -2.027e+01 -7.772e-16  4.923e+00  6.223e+00
##   Sample72
## -5.056e+00
##
## Coefficients: (1 not defined because of singularities)
##                   Estimate Std. Error t value Pr(>|t|)
## (Intercept)      -172.48350  205.54635  -0.839    0.449
## Plastron_length     0.06145    0.58486   0.105    0.921
## SexM              -14.26572   15.75406  -0.906    0.416
## log10_read_depth   43.42744   45.76862   0.949    0.396
## Year                     NA         NA      NA       NA
##
## Residual standard error: 14.11 on 4 degrees of freedom
## Multiple R-squared:  0.4291, Adjusted R-squared:  0.0009518
## F-statistic: 1.002 on 3 and 4 DF,  p-value: 0.4782
```

```r
confint(pl.Observed.STCA)
```

```
##                        2.5 %     97.5 %
## (Intercept)      -743.171660 398.204664
## Plastron_length    -1.562386   1.685289
## SexM              -58.006014  29.474577
## log10_read_depth  -83.646633 170.501512
## Year                      NA         NA
```

```r
check_model(pl.Observed.STCA)
```

```
## Model matrix is rank deficient. VIFs may not be sensible.
```

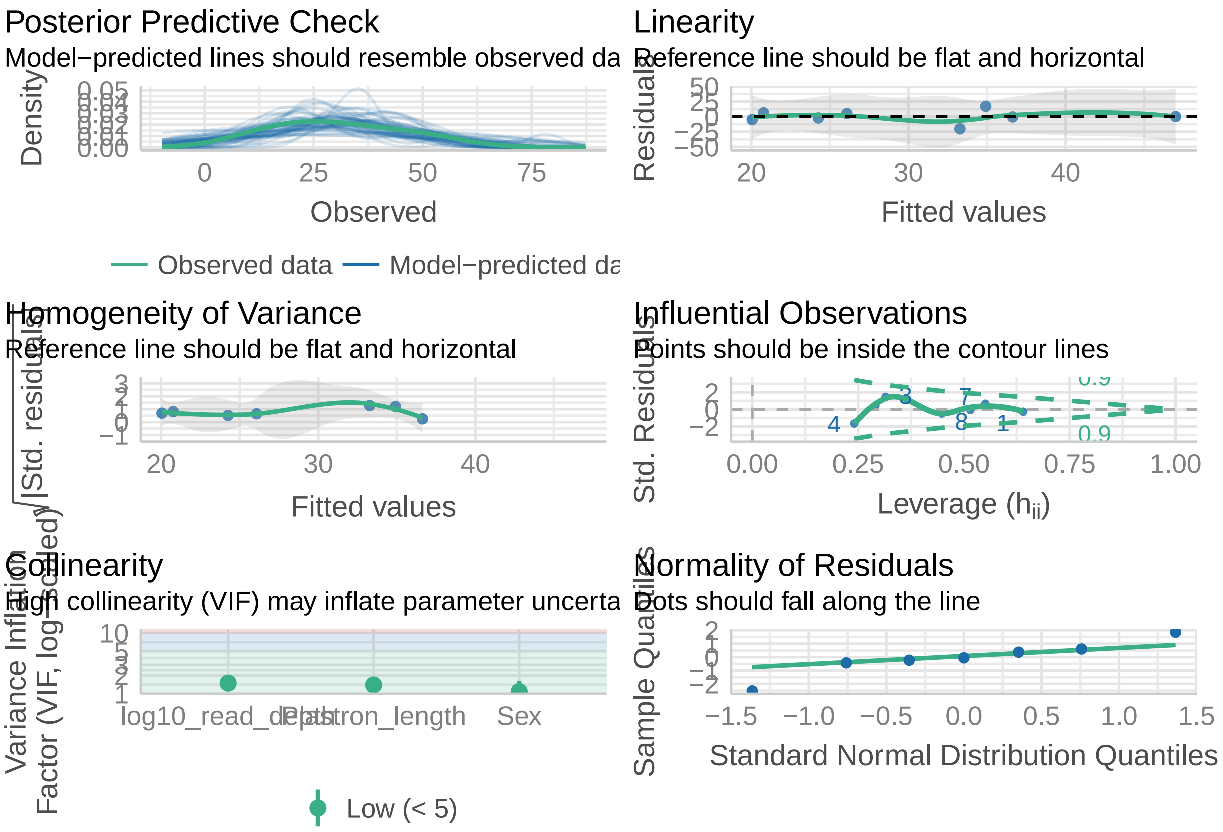


```r
plot(pl.Observed.STCA, which = 1)
```

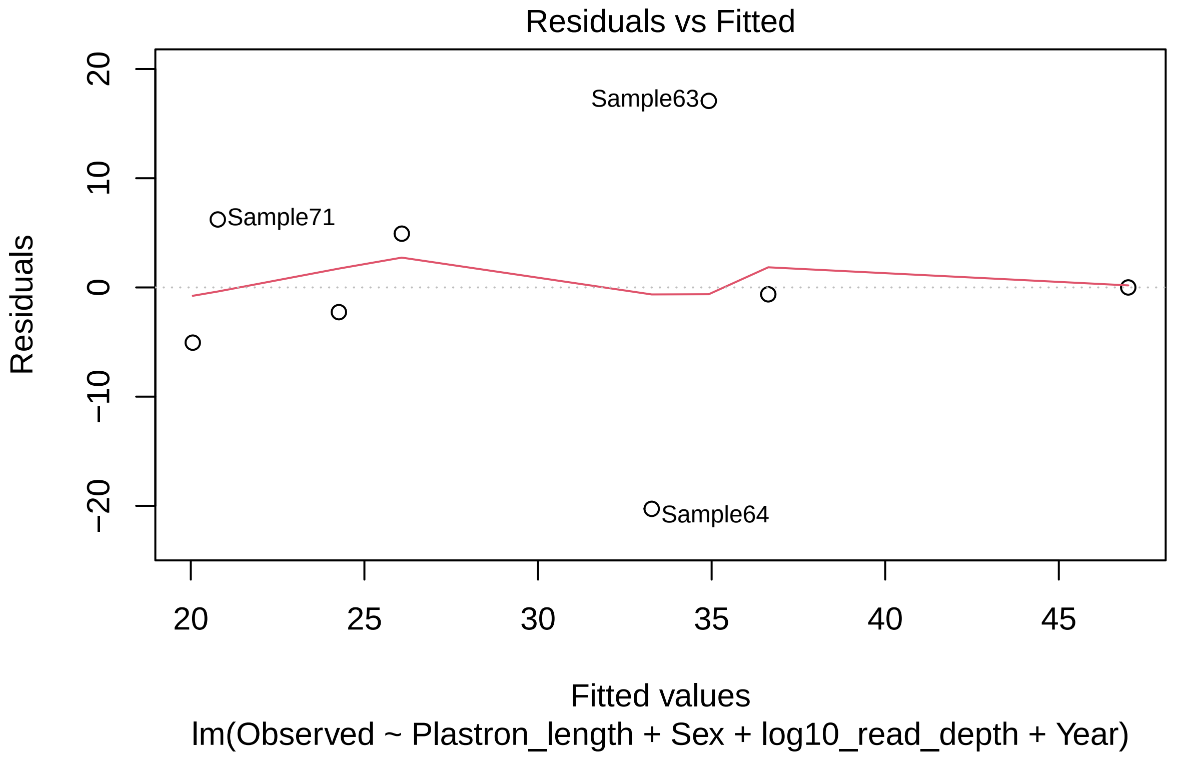

```r
plot(pl.Observed.STCA, which = 2)
```

```
## Warning: not plotting observations with leverage one:
##   5
```

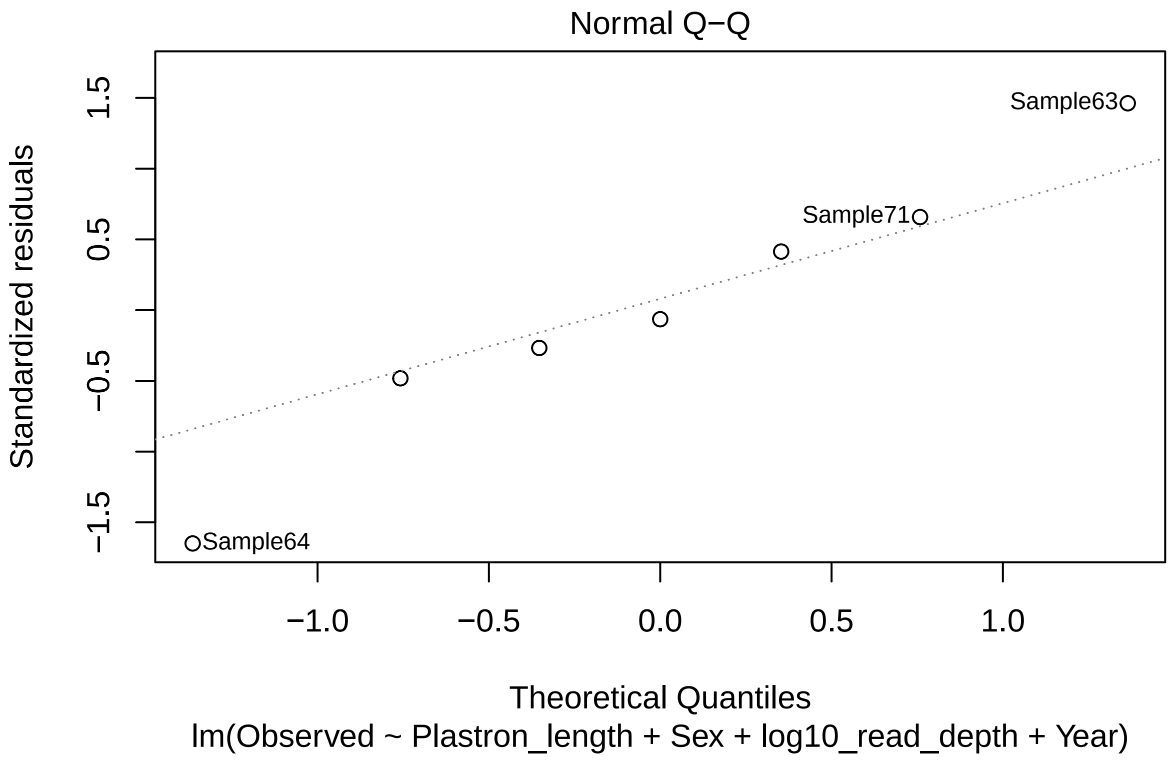


```r
#check for effects in STOD
pl.Observed.STOD <- lm(Observed~Plastron_length+Sex+log10_read_depth+Year,data=STOD)

summary(pl.Observed.STOD)
```

```
##
## Call:
## lm(formula = Observed ~ Plastron_length + Sex + log10_read_depth +
##     Year, data = STOD)
##
## Residuals:
##     Min      1Q  Median      3Q     Max
## -25.957  -7.435   0.000   8.484  23.738
##
## Coefficients:
##                   Estimate Std. Error t value Pr(>|t|)
## (Intercept)      34023.1035 15142.9712   2.247   0.0367 *
## Plastron_length     -1.5688     0.5528  -2.838   0.0105 *
## SexJ               -57.2849    23.1137  -2.478   0.0228 *
## SexM                -7.7707     8.0854  -0.961   0.3486
## log10_read_depth    96.9541    37.8951   2.558   0.0192 *
## Year               -17.0017     7.4617  -2.279   0.0344 *
## ---
```

```
## Signif. codes:  0 '***' 0.001 '**' 0.01 '*' 0.05 '.' 0.1 ' ' 1
##
## Residual standard error: 15.42 on 19 degrees of freedom
## Multiple R-squared:  0.6036, Adjusted R-squared:  0.4992
## F-statistic: 5.785 on 5 and 19 DF,  p-value: 0.002075
```

```
confint(pl.Observed.STOD)
```

```
##                          2.5 %        97.5 %
## (Intercept)        2328.500618 65717.7064568
## Plastron_length      -2.725698    -0.4118048
## SexJ               -105.662339    -8.9074348
## SexM                -24.693565     9.1521369
## log10_read_depth     17.638724   176.2694060
## Year                -32.619310    -1.3841759
```

```
check_model(pl.Observed.STOD)
```

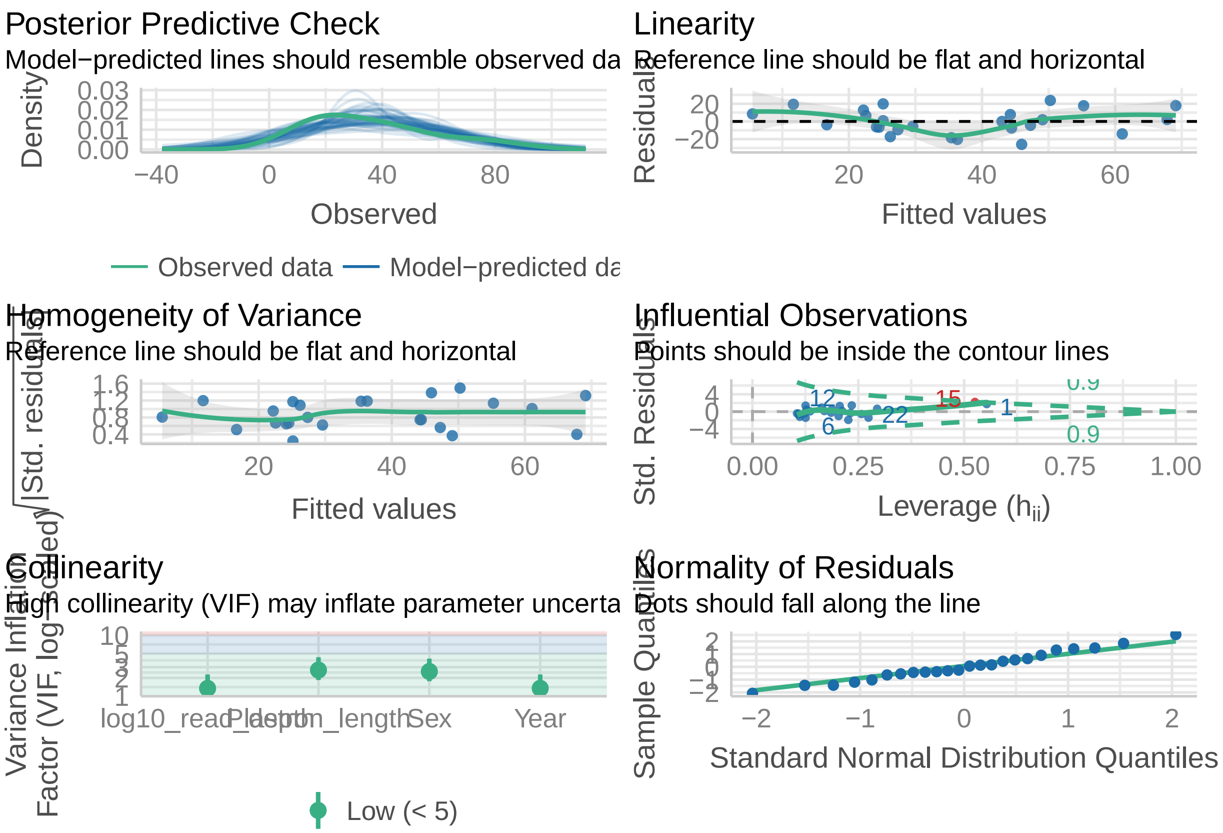

```r
plot(pl.Observed.STOD, which = 1)
```

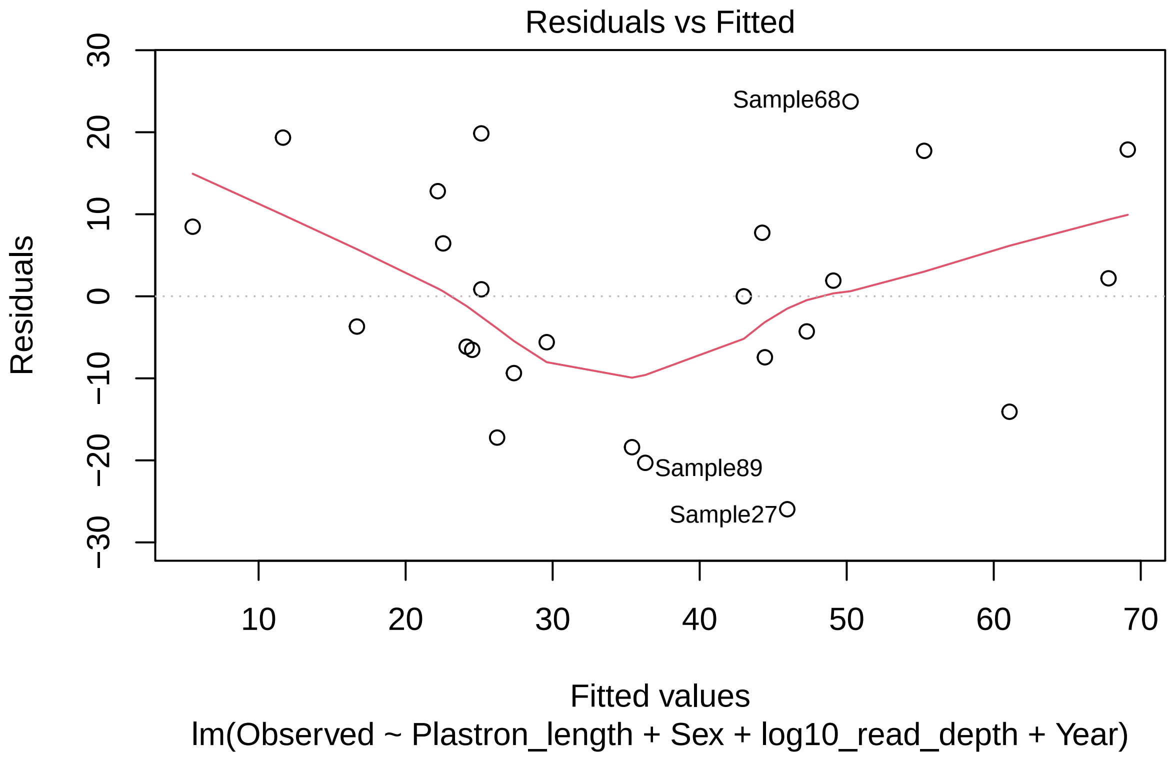


```r
plot(pl.Observed.STOD, which = 2)
```

```
## Warning: not plotting observations with leverage one:
##   3
```

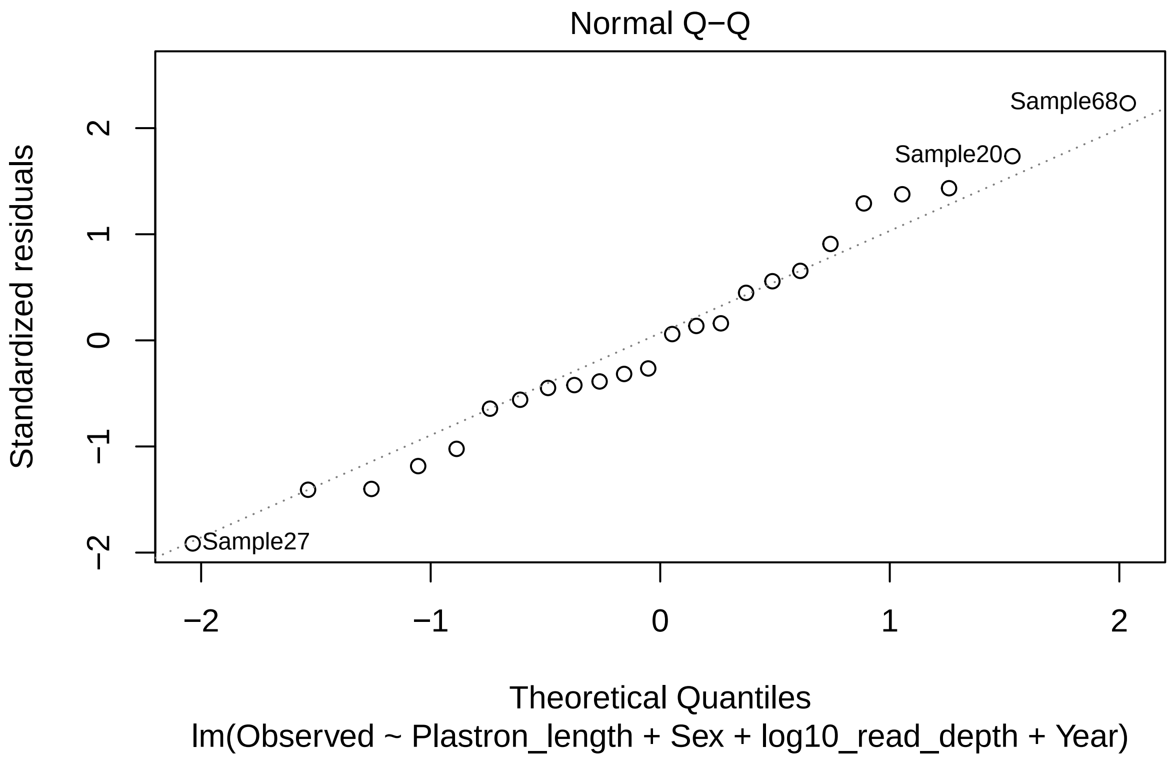


```r
#check for effects in TRSC
pl.Observed.TRSC <- lm(Observed~Plastron_length+Sex+log10_read_depth+Year,data=TRSC)

summary(pl.Observed.TRSC)
```

```
##
## Call:
## lm(formula = Observed ~ Plastron_length + Sex + log10_read_depth +
##     Year, data = TRSC)
##
## Residuals:
##     Min      1Q  Median      3Q     Max
## -39.271 -14.031  -0.234   6.680  45.049
##
## Coefficients:
##                   Estimate Std. Error t value Pr(>|t|)
## (Intercept)      -1.879e+04  2.720e+04  -0.691  0.49963
## Plastron_length   1.343e-01  2.535e-01   0.530  0.60357
## SexJ              2.267e+01  3.304e+01   0.686  0.50240
## SexM              3.764e+01  1.275e+01   2.952  0.00936 **
## log10_read_depth  1.363e+02  7.322e+01   1.862  0.08112 .
## Year              8.975e+00  1.347e+01   0.666  0.51477
## ---
## Signif. codes:  0 '***' 0.001 '**' 0.01 '*' 0.05 '.' 0.1 ' ' 1
##
## Residual standard error: 24.38 on 16 degrees of freedom
## Multiple R-squared:  0.4487, Adjusted R-squared:  0.2764
## F-statistic: 2.604 on 5 and 16 DF,  p-value: 0.06598
```

```r
confint(pl.Observed.TRSC)
```

```
##                         2.5 %       97.5 %
## (Intercept)       -7.644749e+04 3.887215e+04
## Plastron_length   -4.031047e-01 6.716791e-01
## SexJ              -4.736850e+01 9.271325e+01
## SexM               1.061247e+01 6.466240e+01
## log10_read_depth -1.890704e+01 2.915235e+02
## Year              -1.958491e+01 3.753576e+01
```

```r
check_model(pl.Observed.TRSC)
```

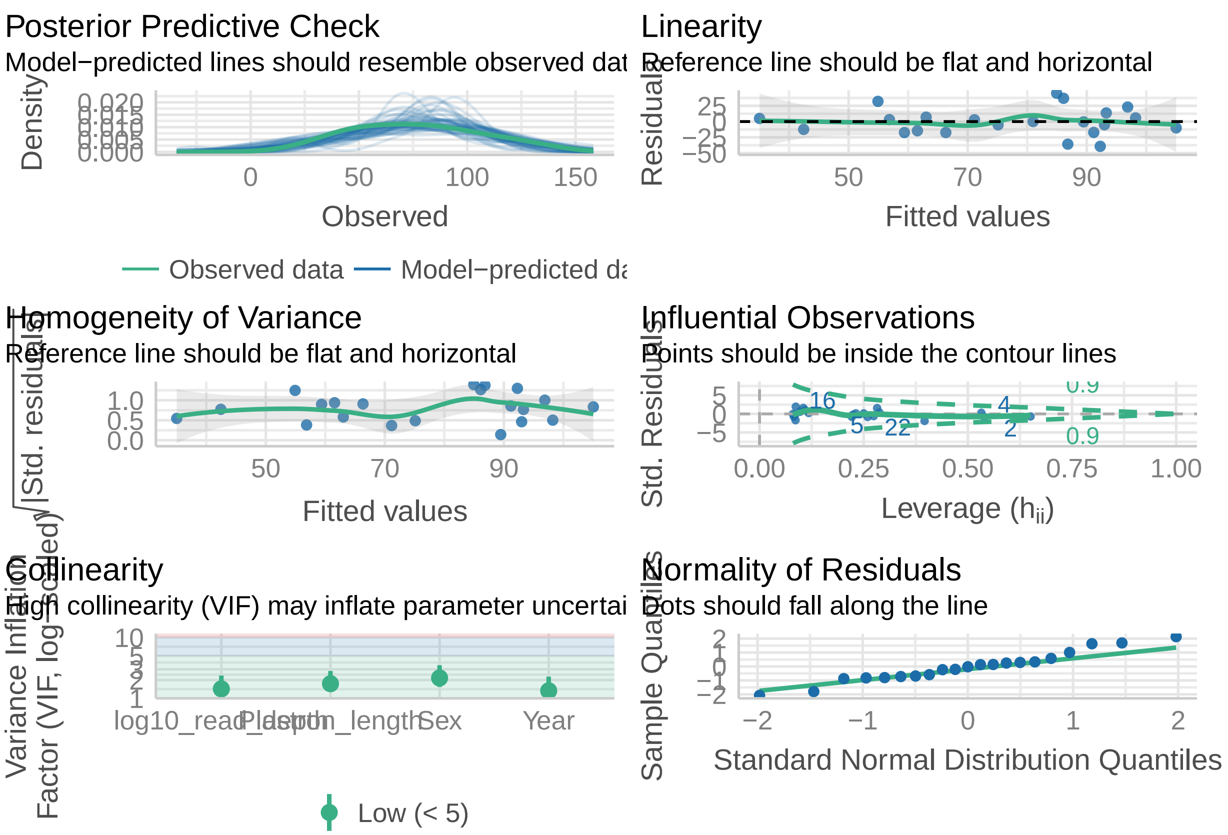


```r
plot(pl.Observed.TRSC, which = 1)
```

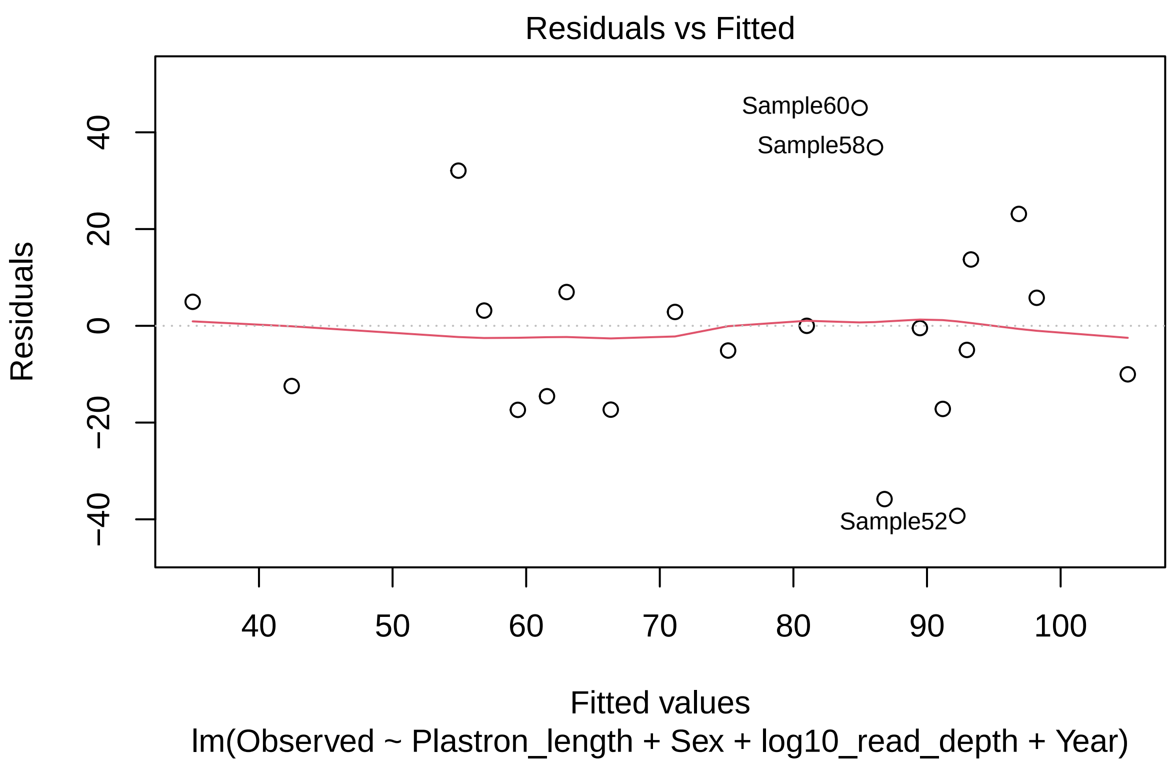


```
plot(pl.Observed.TRSC, which = 2)
```

```
## Warning: not plotting observations with leverage one:
##   6
```

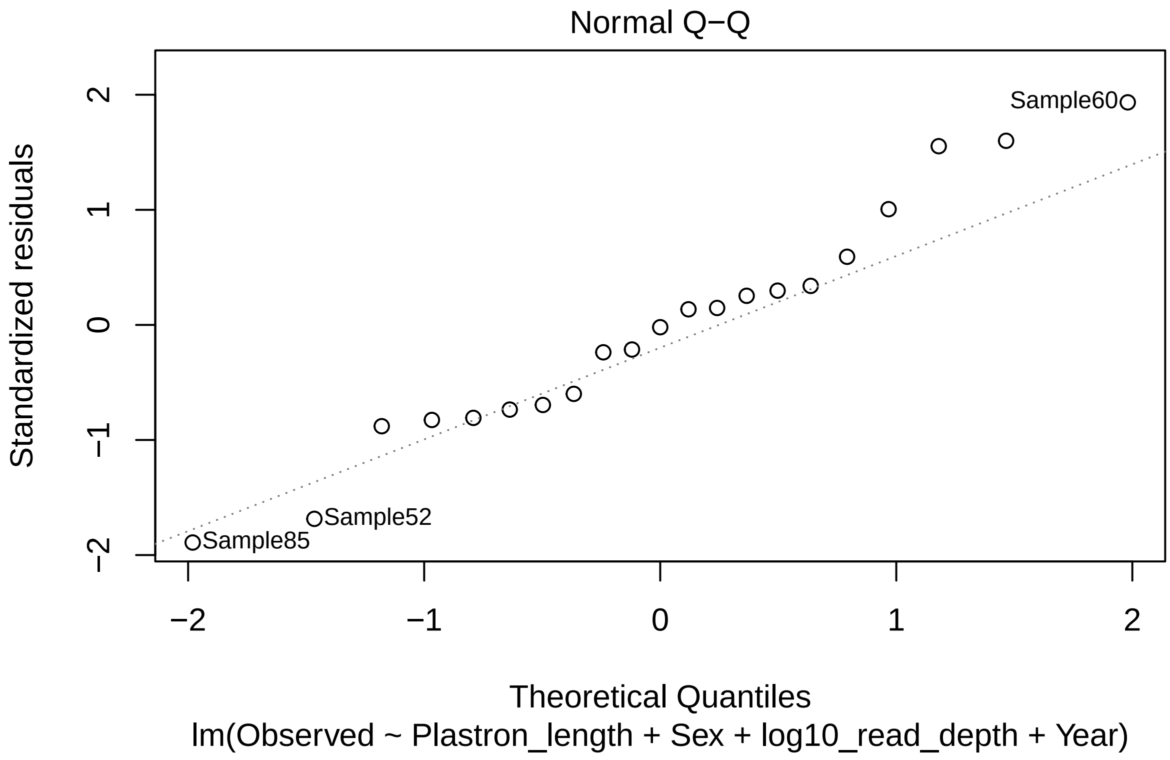

```r
#Check Faith's Phylogenetic Diversity
#check for effects in KISU
pl.PD.KISU <- lm(PD~Plastron_length+Site+Sex+log10_read_depth+Year,data=KISU)

summary(pl.PD.KISU)
```

```
##
## Call:
## lm(formula = PD ~ Plastron_length + Site + Sex + log10_read_depth +
##     Year, data = KISU)
##
## Residuals:
##     Min      1Q  Median      3Q     Max
## -3.2507 -1.6050  0.3474  1.3597  2.9534
##
## Coefficients:
##                    Estimate Std. Error t value Pr(>|t|)
## (Intercept)      3173.98706 3541.83577   0.896   0.3963
## Plastron_length    -0.06411    0.12560  -0.510   0.6235
## SiteS4             -0.85786    2.21187  -0.388   0.7082
## SexM               -0.24237    2.20089  -0.110   0.9150
## log10_read_depth   18.32210    7.41468   2.471   0.0386 *
## Year               -1.60764    1.74871  -0.919   0.3848
## ---
## Signif. codes:  0 '***' 0.001 '**' 0.01 '*' 0.05 '.' 0.1 ' ' 1
##
## Residual standard error: 2.618 on 8 degrees of freedom
## Multiple R-squared:  0.5628, Adjusted R-squared:  0.2896
## F-statistic:  2.06 on 5 and 8 DF,  p-value: 0.1736
```

```r
confint(pl.PD.KISU)
```

```
##                          2.5 %       97.5 %
## (Intercept)      -4993.5008768 1.134147e+04
## Plastron_length     -0.3537385 2.255178e-01
## SiteS4              -5.9584507 4.242730e+00
## SexM                -5.3176325 4.832901e+00
## log10_read_depth     1.2238235 3.542037e+01
## Year                -5.6401757 2.424905e+00
```

```r
check_model(pl.PD.KISU)
```

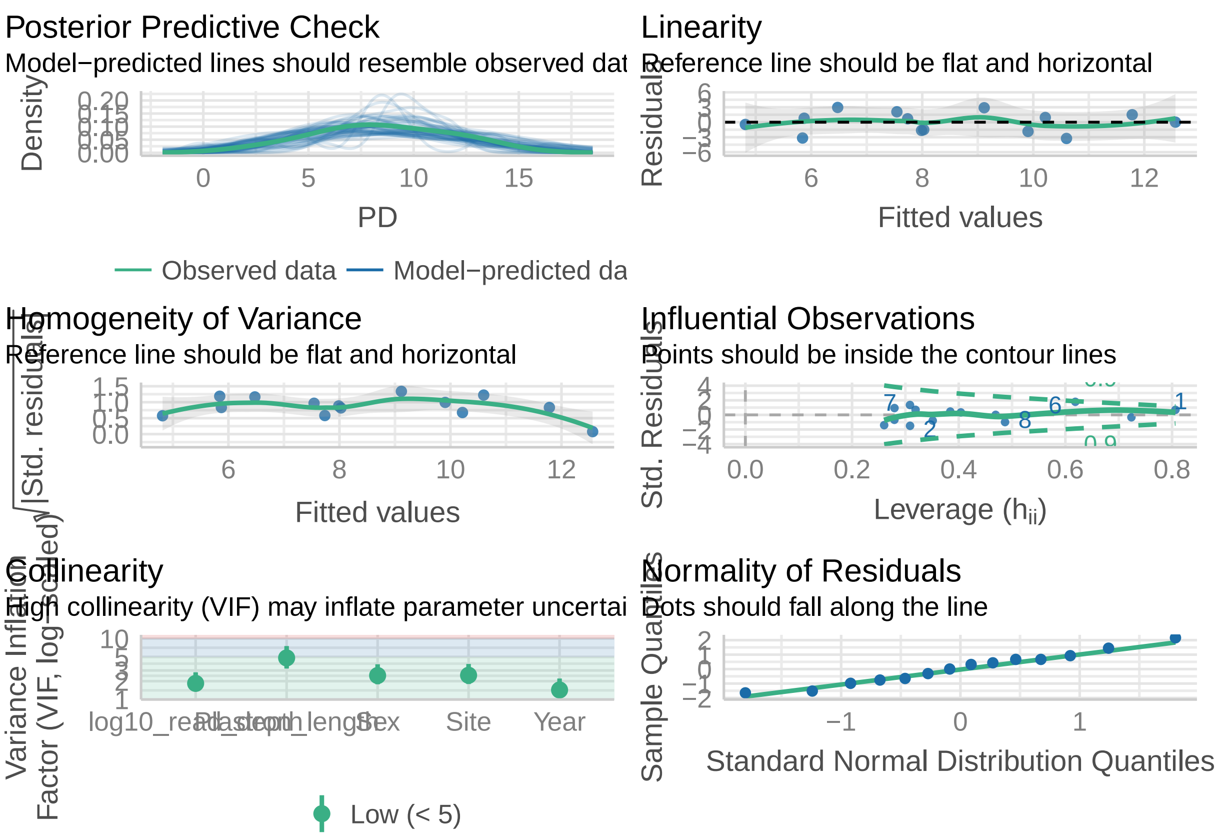


```r
plot(pl.PD.KISU, which = 1)
```

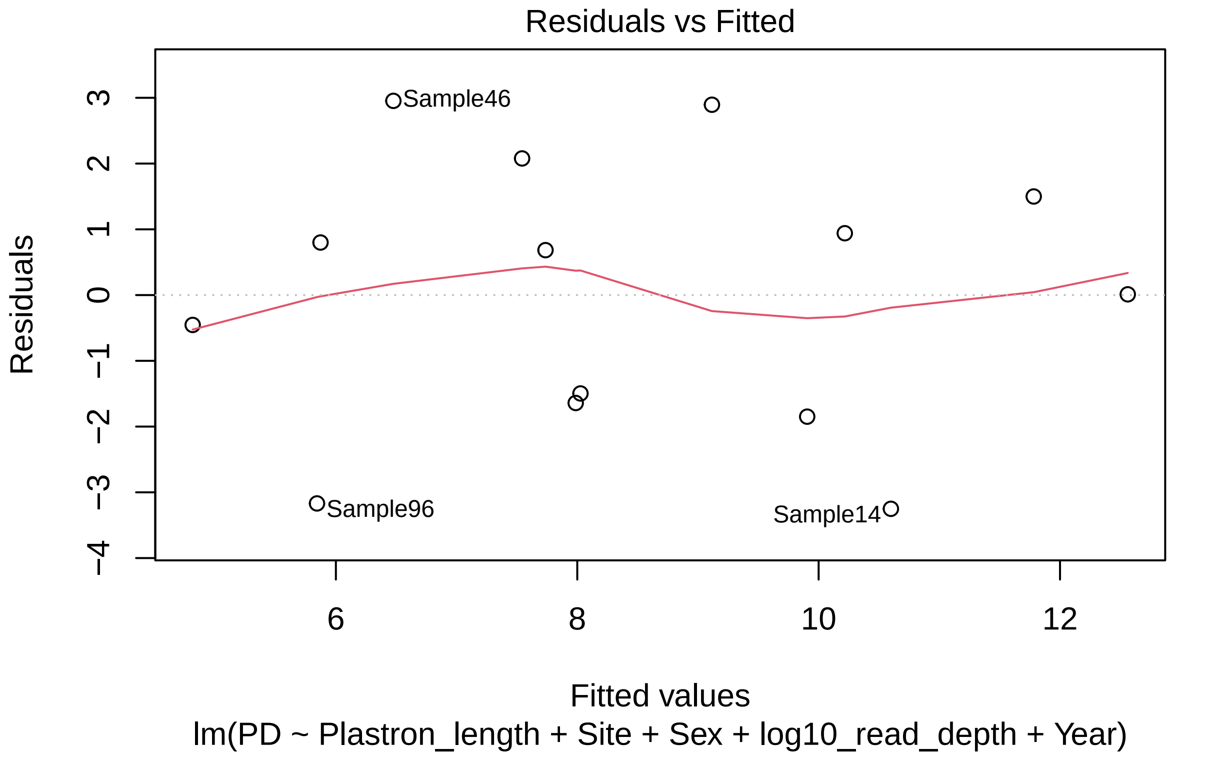

```r
plot(pl.PD.KISU, which = 2)
```

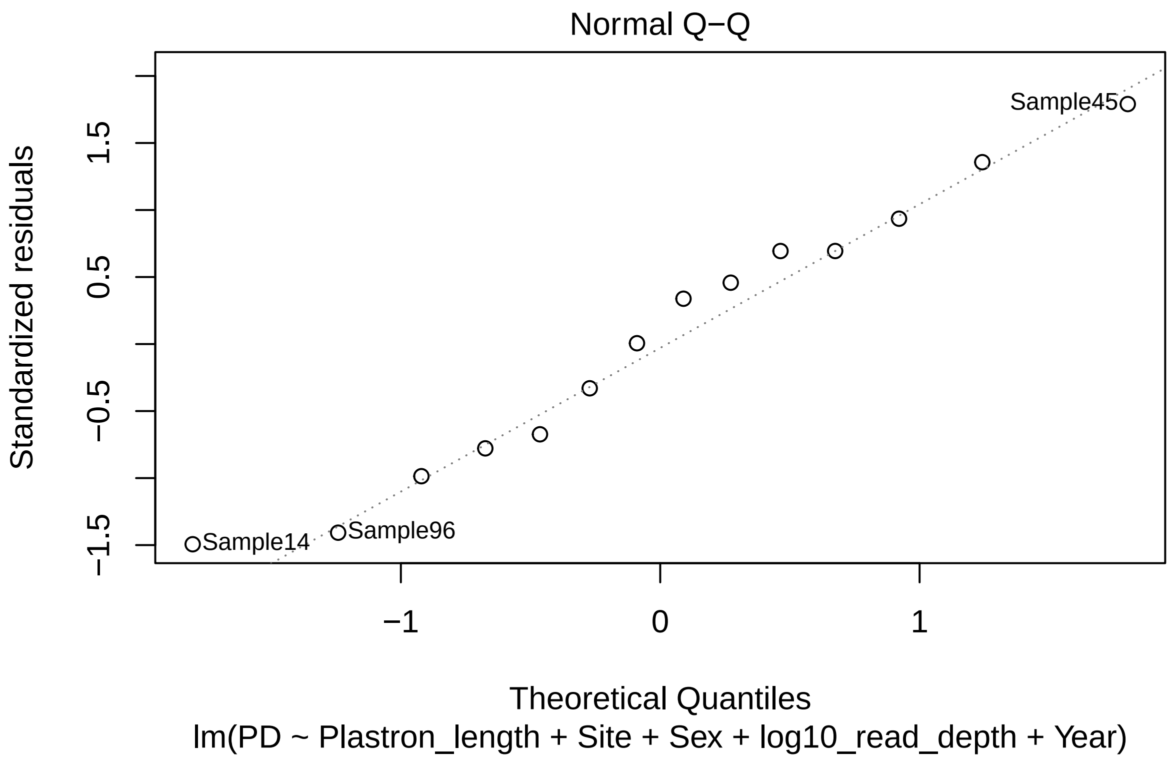


```r
#check for effects in PSCO
#took out variable 'sex' from PSCO model for PD, as sex and plastron_length were highly collinear
pl.PD.PSCO <- lm(PD~Plastron_length+log10_read_depth+Year,data=PSCO)

summary(pl.PD.PSCO)
```

```
##
## Call:
## lm(formula = PD ~ Plastron_length + log10_read_depth + Year,
##     data = PSCO)
##
## Residuals:
##     Min      1Q  Median      3Q     Max
## -2.4512 -0.8006  0.0518  0.6219  3.9381
##
## Coefficients:
##                    Estimate Std. Error t value Pr(>|t|)
## (Intercept)      4402.69939 4623.44791   0.952   0.3688
## Plastron_length     0.01300    0.01226   1.061   0.3197
## log10_read_depth   13.08996    6.57637   1.990   0.0817 .
## Year               -2.20485    2.27464  -0.969   0.3608
## ---
## Signif. codes:  0 '***' 0.001 '**' 0.01 '*' 0.05 '.' 0.1 ' ' 1
##
## Residual standard error: 1.92 on 8 degrees of freedom
## Multiple R-squared:  0.7033, Adjusted R-squared:  0.592
## F-statistic: 6.321 on 3 and 8 DF,  p-value: 0.01665
```

```r
confint(pl.PD.PSCO)
```

```
##                           2.5 %       97.5 %
## (Intercept)       -6.258991e+03 1.506439e+04
## Plastron_length   -1.526294e-02 4.126904e-02
## log10_read_depth  -2.075172e+00 2.825509e+01
## Year              -7.450190e+00 3.040482e+00
```

```r
check_model(pl.PD.PSCO)
```

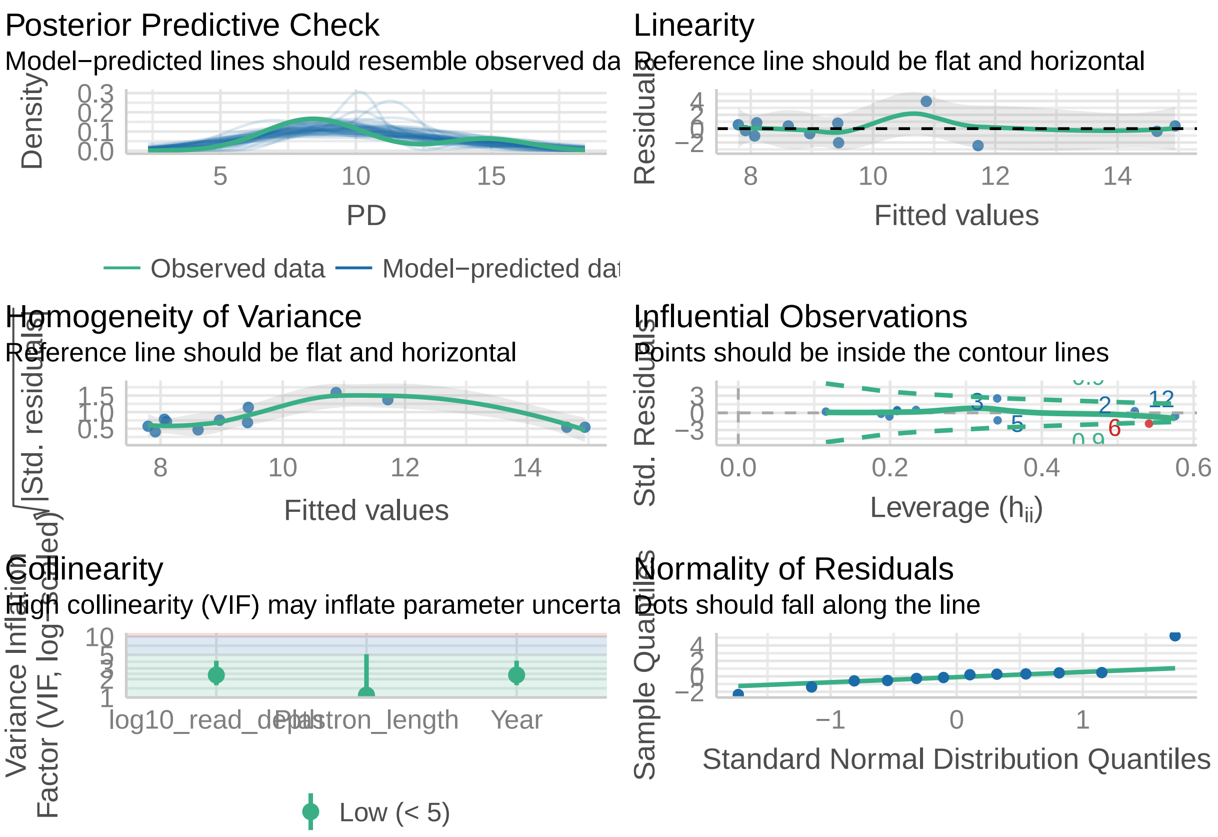


```r
plot(pl.PD.PSCO, which = 1)
```

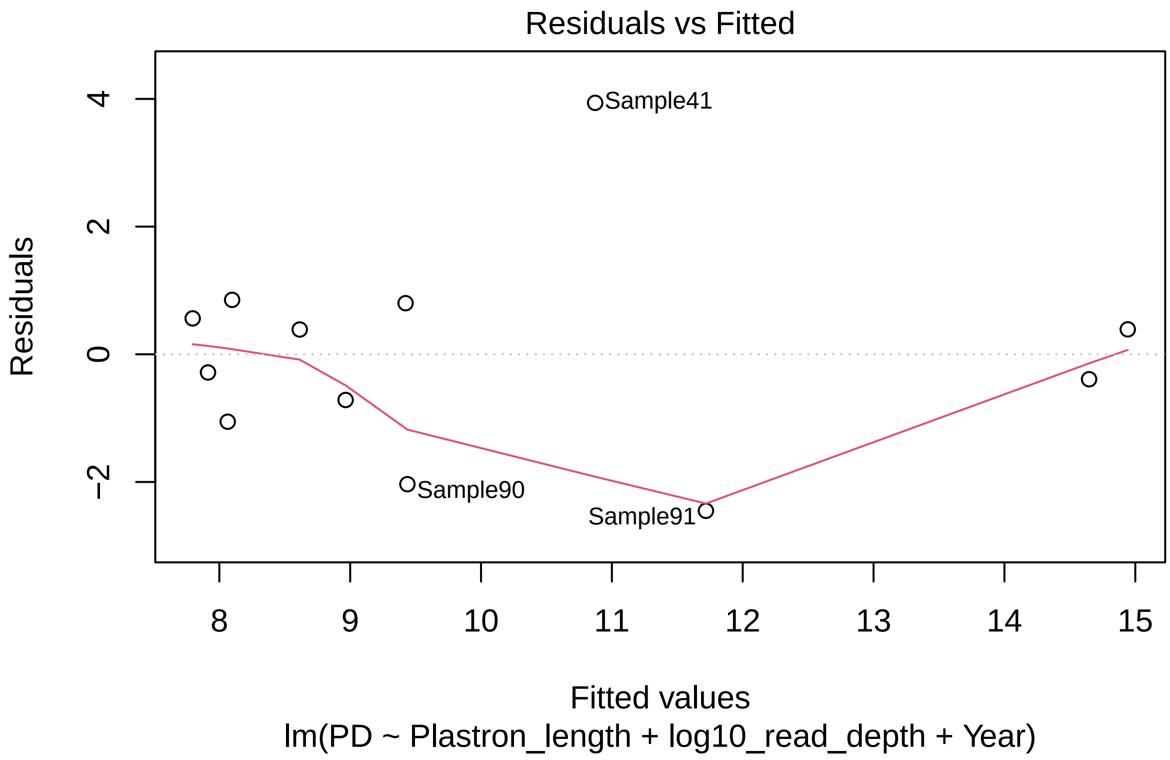


```
plot(pl.PD.PSCO, which = 2)
```

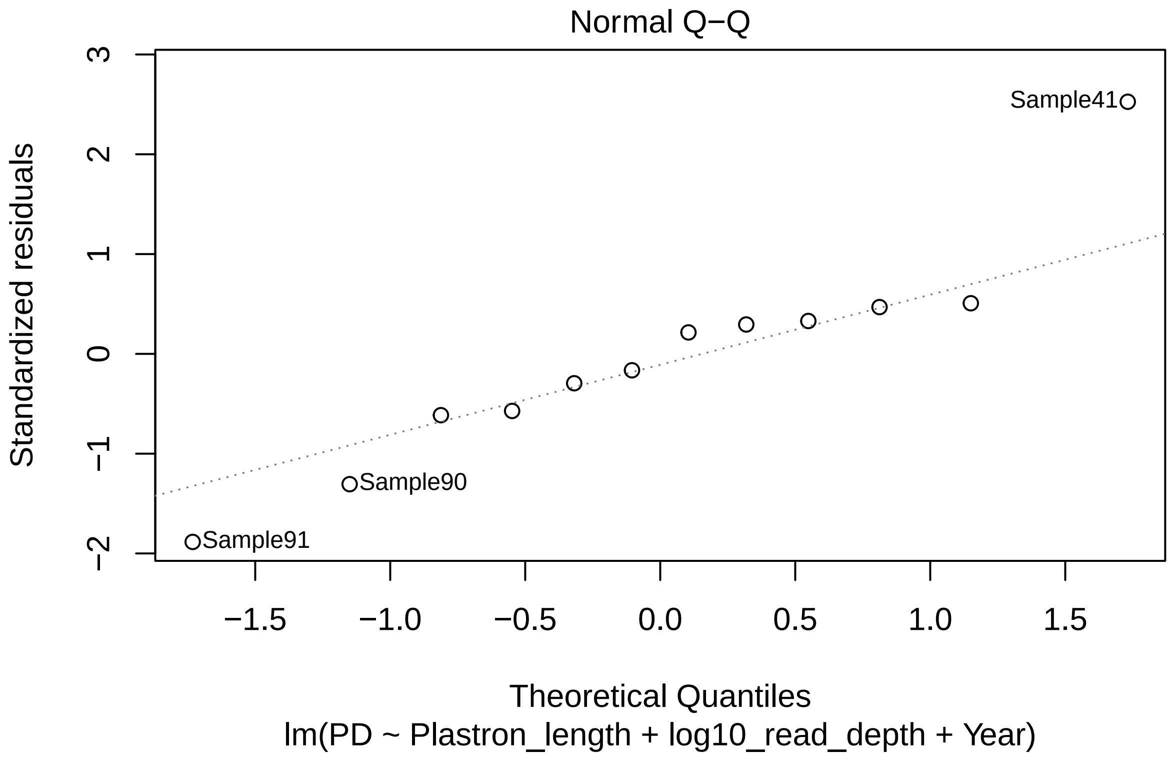


```
#check for effects in STCA
pl.PD.STCA <- lm(PD~Plastron_length+Sex+log10_read_depth+Year,data=STCA)
```

```r
summary(pl.PD.STCA)
```

```
##
## Call:
## lm(formula = PD ~ Plastron_length + Sex + log10_read_depth +
##     Year, data = STCA)
##
## Residuals:
##   Sample55   Sample62   Sample63   Sample64   Sample69   Sample70   Sample71
## -8.492e-01 -2.509e-01  1.862e+00 -2.198e+00  1.110e-16  1.422e+00  7.562e-01
##   Sample72
## -7.427e-01
##
## Coefficients: (1 not defined because of singularities)
##                   Estimate Std. Error t value Pr(>|t|)
## (Intercept)      -12.34802   25.46078  -0.485    0.653
## Plastron_length   -0.01487    0.07245  -0.205    0.847
## SexM              -1.15377    1.95144  -0.591    0.586
## log10_read_depth   4.39947    5.66930   0.776    0.481
## Year                    NA         NA      NA       NA
##
## Residual standard error: 1.748 on 4 degrees of freedom
## Multiple R-squared:  0.2593, Adjusted R-squared:  -0.2963
## F-statistic: 0.4666 on 3 and 4 DF,  p-value: 0.7213
```

```r
confint(pl.PD.STCA)
```

```
##                        2.5 %     97.5 %
## (Intercept)      -83.038487 58.3424381
## Plastron_length   -0.216012  0.1862736
## SexM              -6.571828  4.2642882
## log10_read_depth -11.341043 20.1399848
## Year                     NA         NA
```

```r
check_model(pl.PD.STCA)
```

```
## Model matrix is rank deficient. VIFs may not be sensible.
```

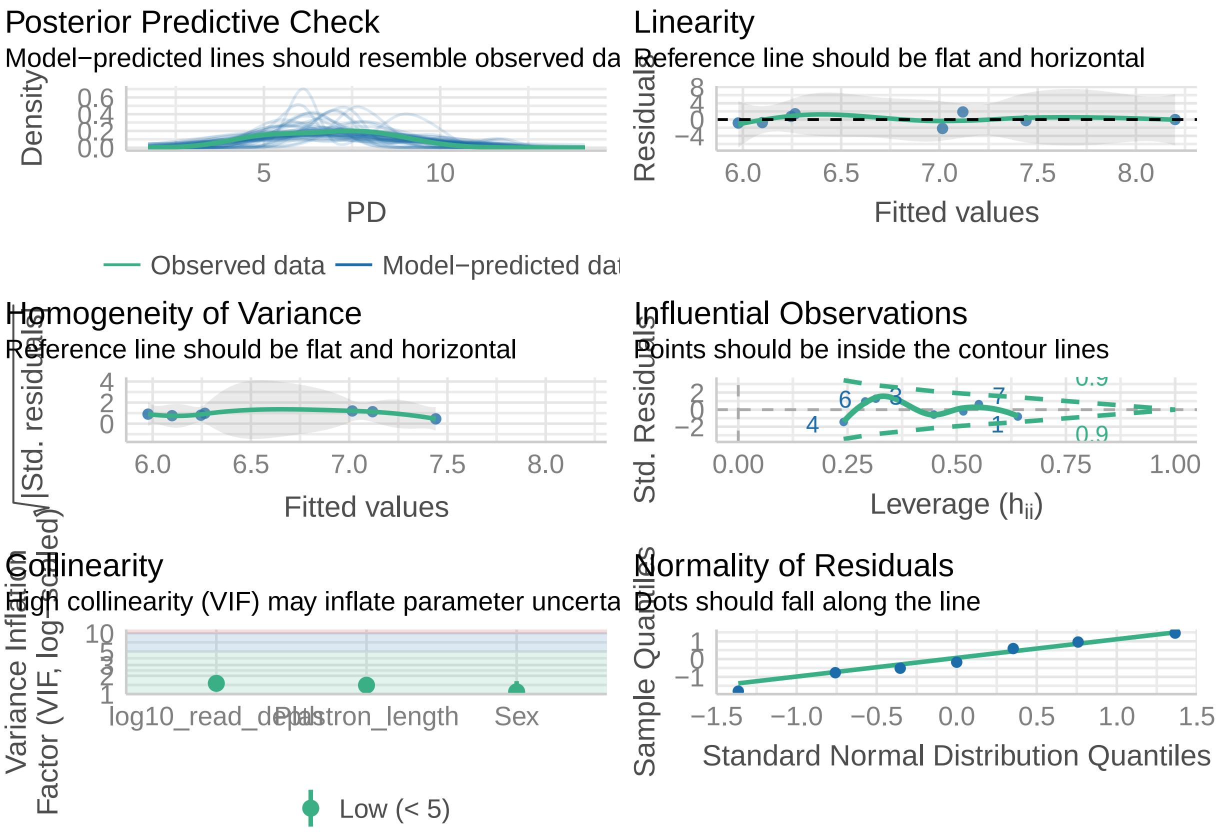


```
plot(pl.PD.STCA, which = 1)
```

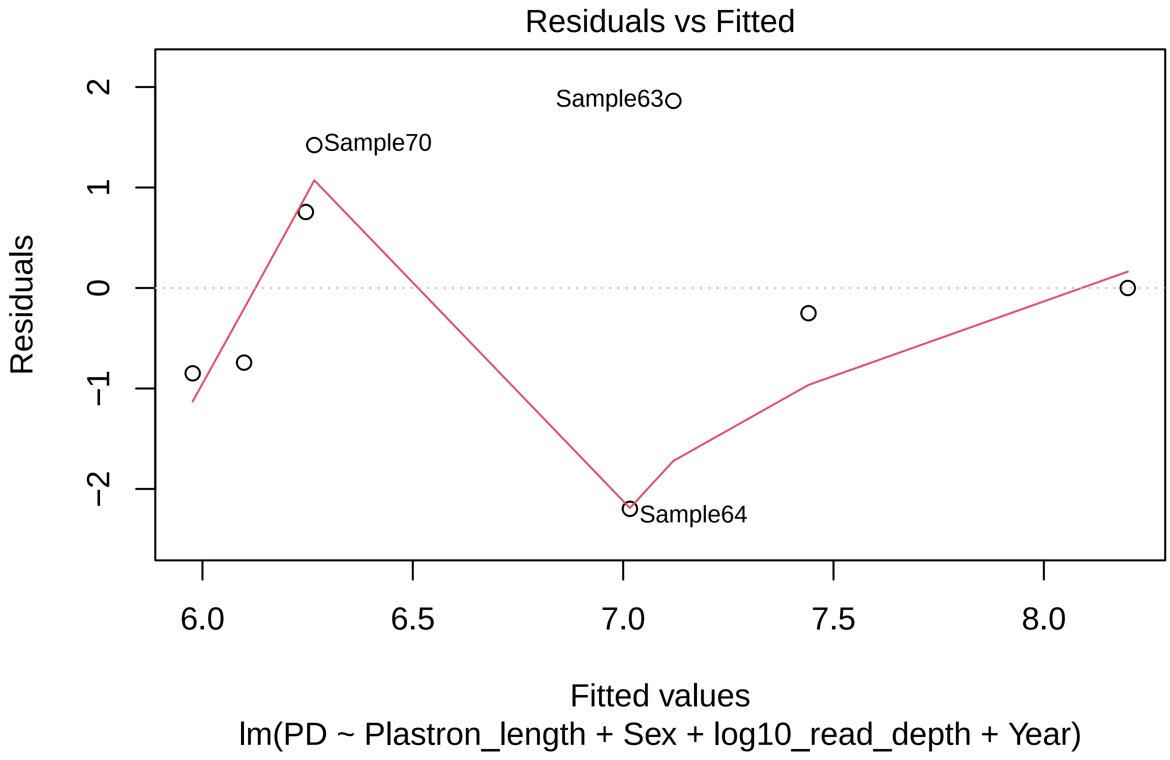

```r
plot(pl.PD.STCA, which = 2)
```

```
## Warning: not plotting observations with leverage one:
##   5
```

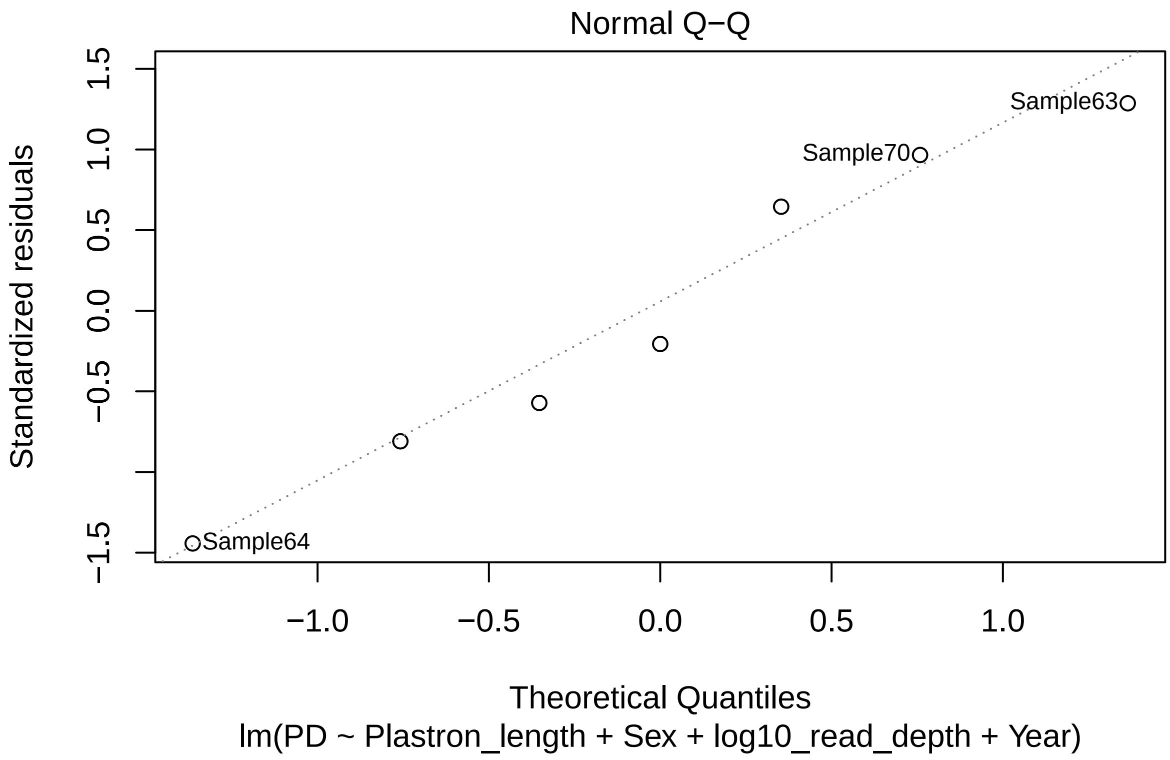


```r
#check for effects in STOD
pl.PD.STOD <- lm(PD~Plastron_length+Sex+log10_read_depth+Year,data=STOD)

summary(pl.PD.STOD)
```

```
##
## Call:
## lm(formula = PD ~ Plastron_length + Sex + log10_read_depth +
##     Year, data = STOD)
##
## Residuals:
##    Min     1Q Median     3Q    Max
## -2.990 -1.363  0.000  1.165  3.054
##
## Coefficients:
##                   Estimate Std. Error t value Pr(>|t|)
## (Intercept)      3231.4927  1893.0745   1.707   0.1041
## Plastron_length    -0.1915     0.0691  -2.771   0.0122 *
## SexJ               -7.1793     2.8895  -2.485   0.0225 *
## SexM               -1.4935     1.0108  -1.478   0.1559
## log10_read_depth   15.0358     4.7374   3.174   0.0050 **
## Year               -1.6258     0.9328  -1.743   0.0975 .
## ---
```

```
## Signif. codes:  0 '***' 0.001 '**' 0.01 '*' 0.05 '.' 0.1 ' ' 1
##
## Residual standard error: 1.928 on 19 degrees of freedom
## Multiple R-squared:  0.5862, Adjusted R-squared:  0.4773
## F-statistic: 5.383 on 5 and 19 DF,  p-value: 0.003005
```

```
confint(pl.PD.STOD)
```

```
##                           2.5 %        97.5 %
## (Intercept)       -730.7577994 7193.74330830
## Plastron_length     -0.3360995   -0.04683183
## SexJ               -13.2270896   -1.13142849
## SexM                -3.6091138    0.62205300
## log10_read_depth     5.1203054   24.95126863
## Year                -3.5782374    0.32657338
```

```
check_model(pl.PD.STOD)
```

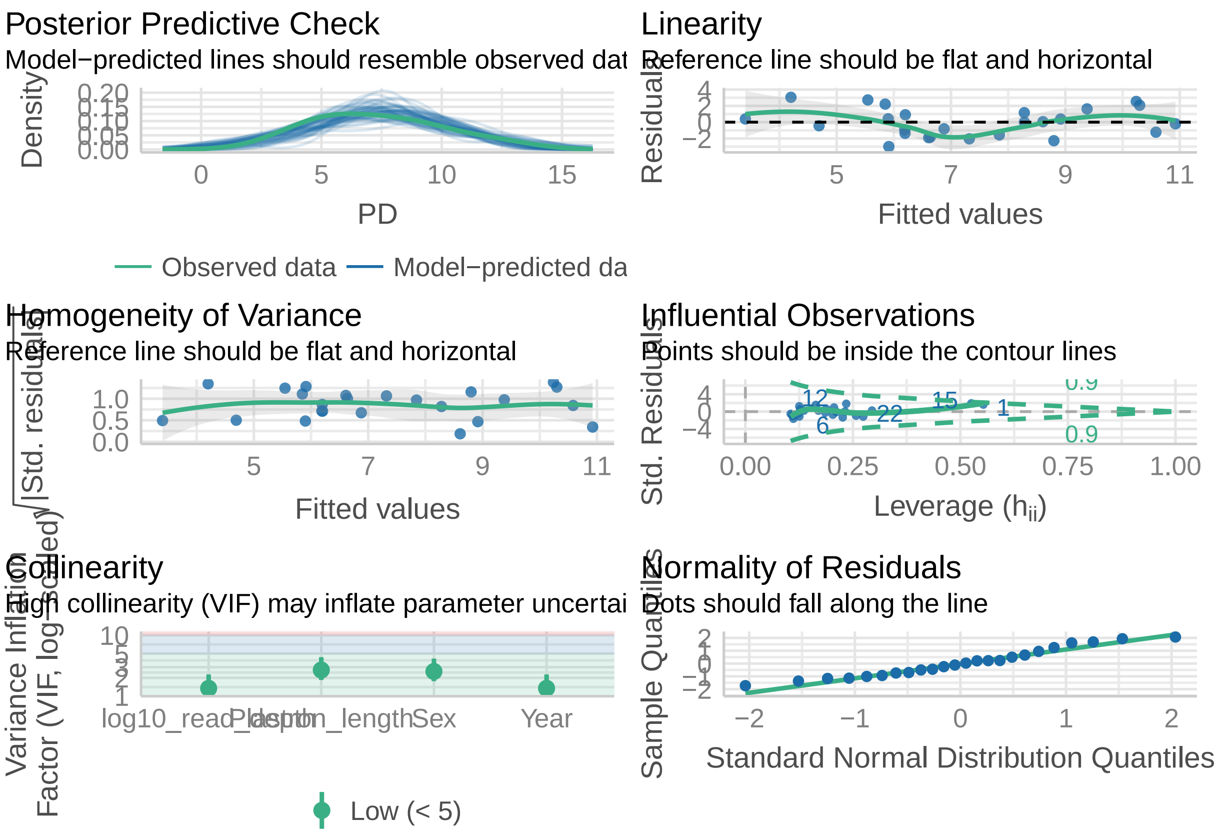

```r
plot(pl.PD.STOD, which = 1)
```

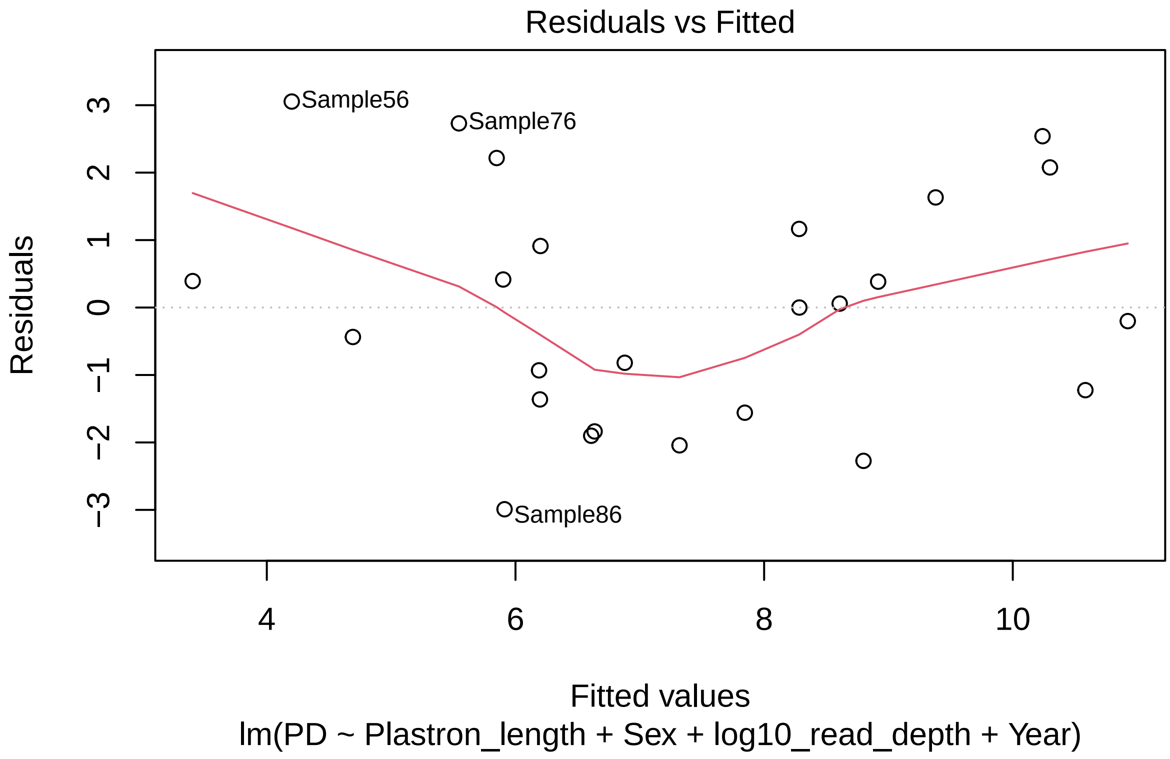


```r
plot(pl.PD.STOD, which = 2)
```

```
## Warning: not plotting observations with leverage one:
##   3
```

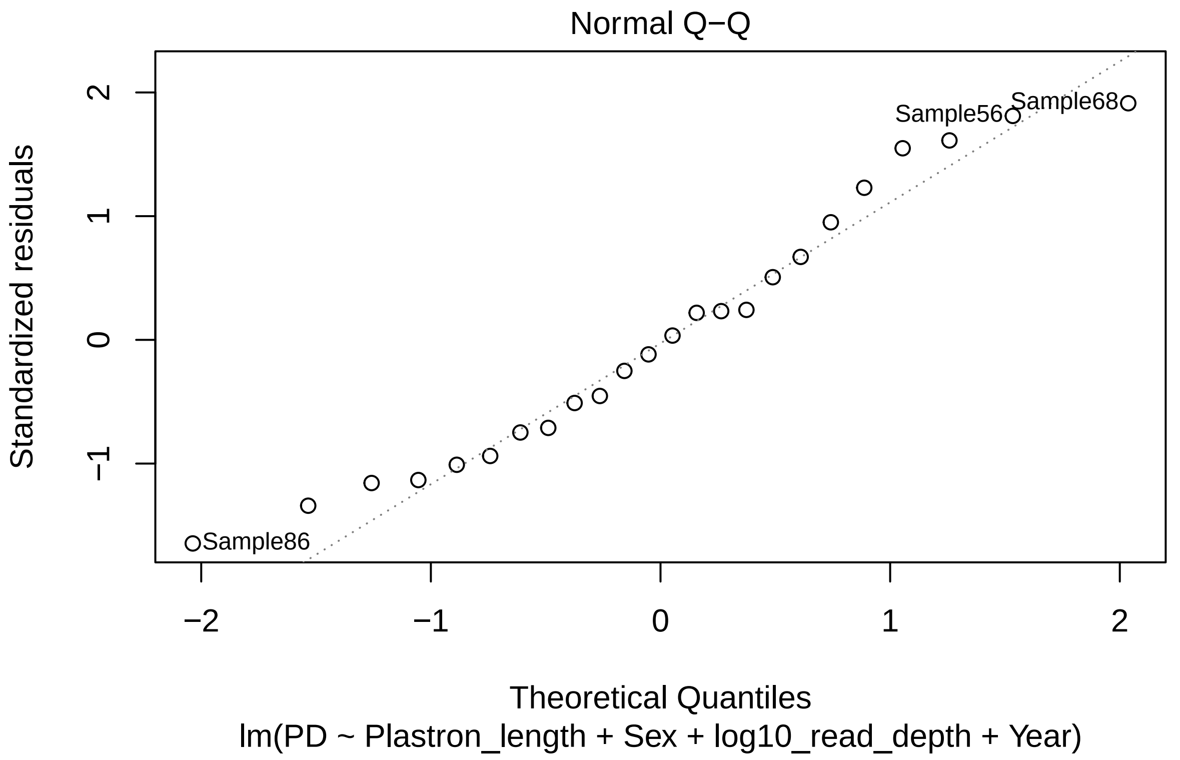


```
#check for effects in TRSC
pl.PD.TRSC <- lm(PD~Plastron_length+Sex+log10_read_depth+Year,data=TRSC)

summary(pl.PD.TRSC)
```

```
##
## Call:
## lm(formula = PD ~ Plastron_length + Sex + log10_read_depth +
##     Year, data = TRSC)
##
## Residuals:
##     Min      1Q  Median      3Q     Max
## -4.5188 -2.0869 -0.4475  2.6020  5.3098
##
## Coefficients:
##                   Estimate Std. Error t value Pr(>|t|)
## (Intercept)      -4.193e+03  3.767e+03  -1.113   0.2821
## Plastron_length   3.236e-02  3.511e-02   0.922   0.3704
## SexJ              4.163e+00  4.576e+00   0.910   0.3764
## SexM              5.043e+00  1.766e+00   2.856   0.0114 *
## log10_read_depth  1.235e+01  1.014e+01   1.218   0.2409
## Year              2.046e+00  1.866e+00   1.096   0.2891
## ---
## Signif. codes:  0 '***' 0.001 '**' 0.01 '*' 0.05 '.' 0.1 ' ' 1
##
## Residual standard error: 3.376 on 16 degrees of freedom
## Multiple R-squared:  0.4579, Adjusted R-squared:  0.2885
## F-statistic: 2.703 on 5 and 16 DF,  p-value: 0.05906
```

```r
confint(pl.PD.TRSC)
```

```
##                          2.5 %       97.5 %
## (Intercept)       -1.217883e+04 3792.6428442
## Plastron_length   -4.207069e-02    0.1067841
## SexJ              -5.537059e+00   13.8638998
## SexM               1.299712e+00    8.7854868
## log10_read_depth -9.146211e+00   33.8476118
## Year              -1.909634e+00    6.0014309
```

```r
check_model(pl.PD.TRSC)
```

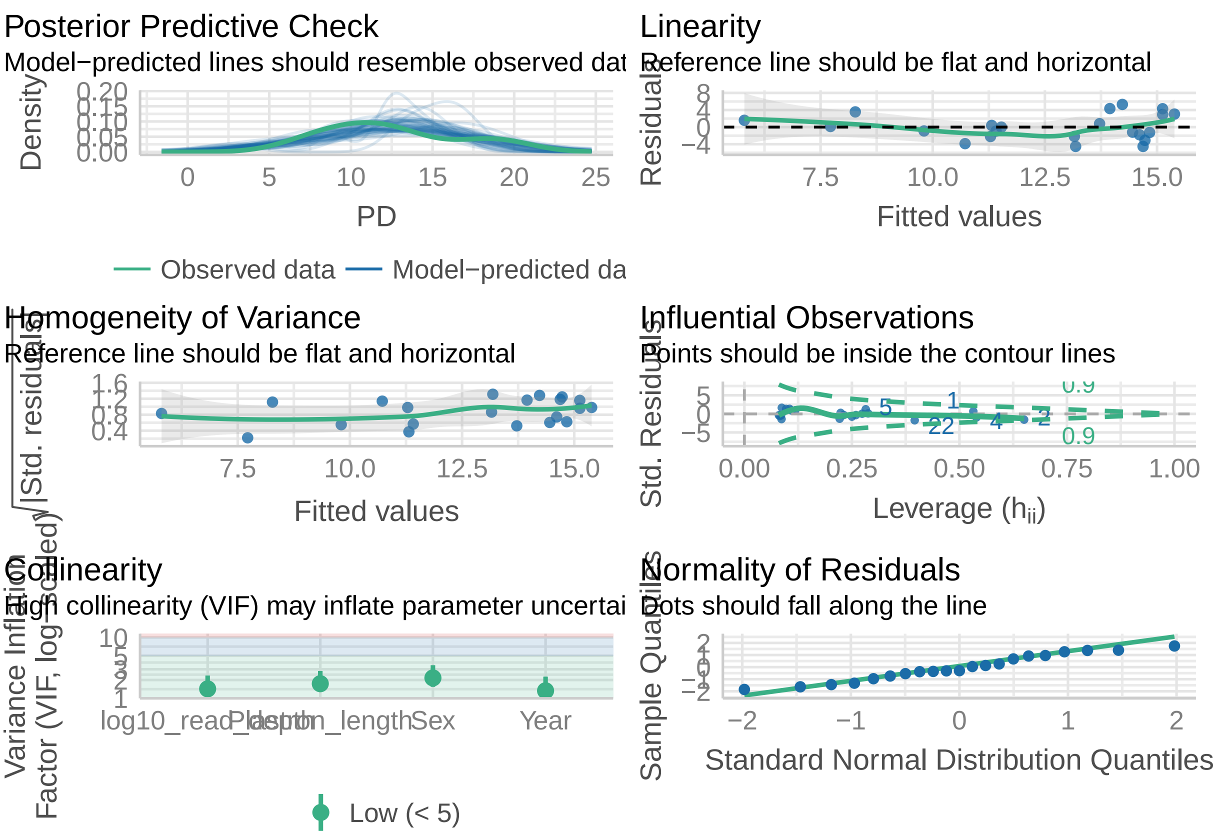


```r
plot(pl.PD.TRSC, which = 1)
```

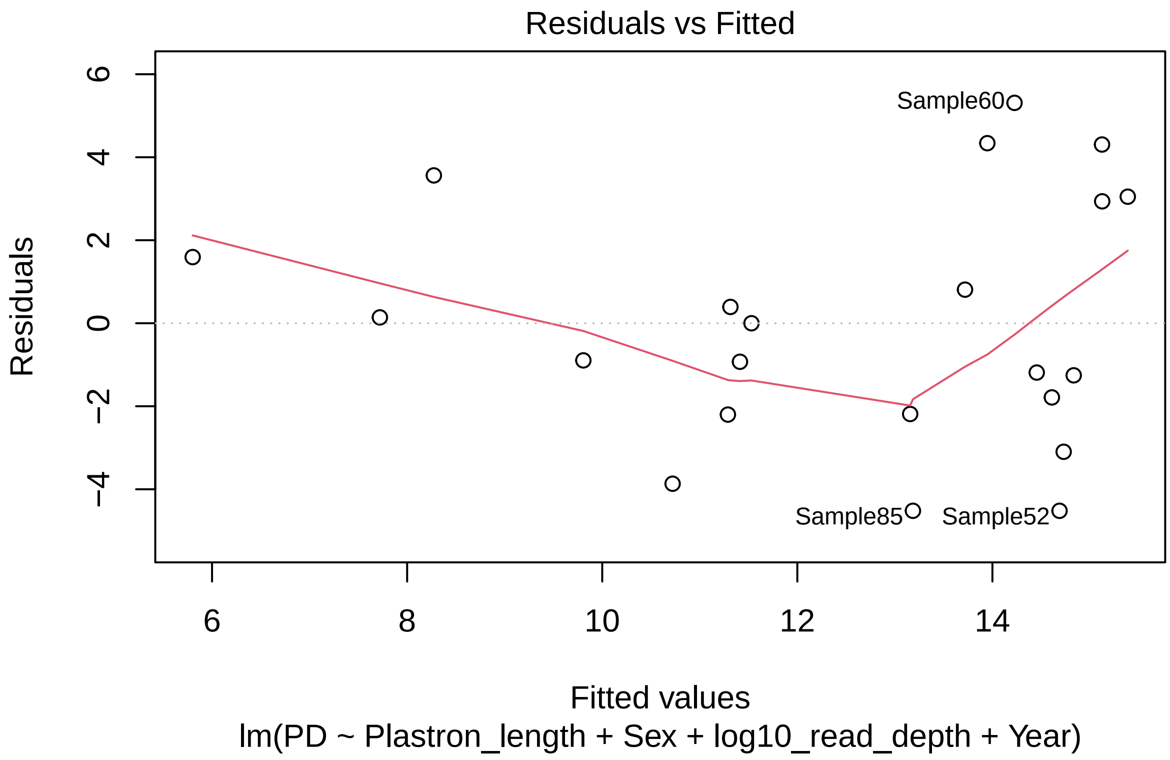


```r
plot(pl.PD.TRSC, which = 2)
```

```
## Warning: not plotting observations with leverage one:
##   6
```

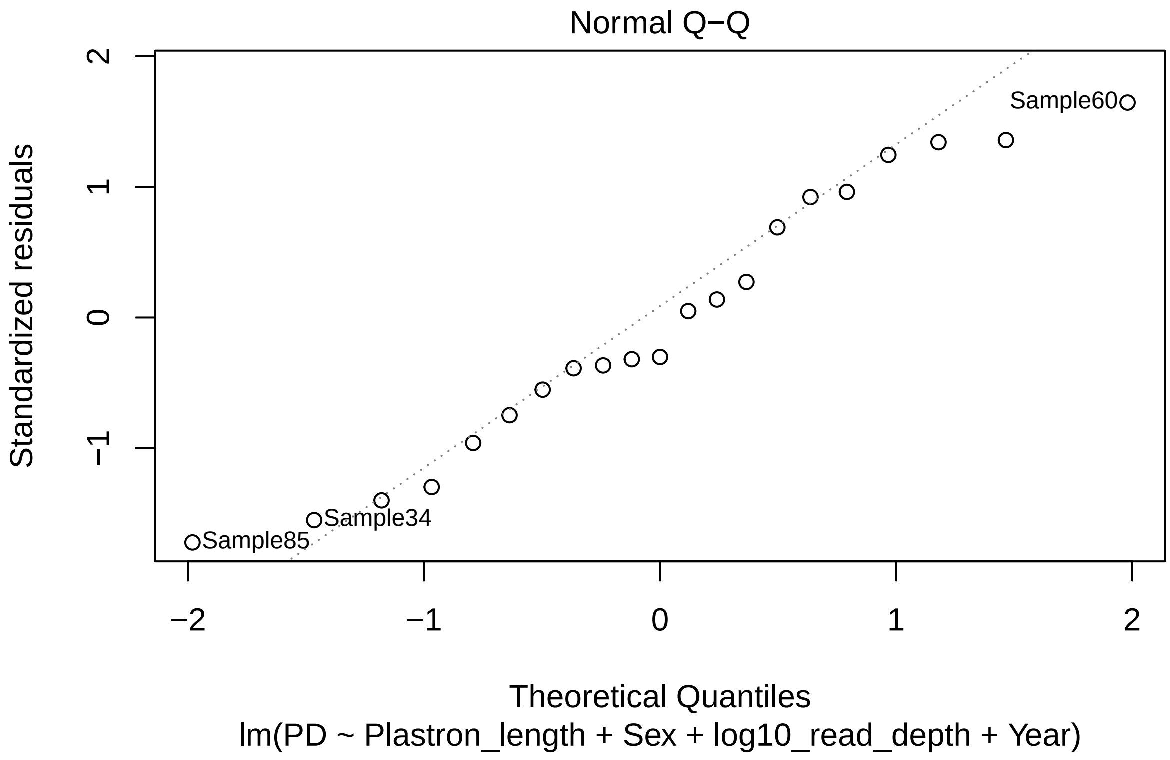

Generate heat maps for different taxonomic levels:

The loop commands here will be used to generate taxonomy-based heat maps for turtle species/collection site and collection site/turtle species combination, at the taxonomic levels of ASV, genus, family, order, class and phylum

```
phylo.heat <- function(phyloseq.object = phylo, data, ID.col = "sample.ID", order1 = NULL, order2 = NUL

  phylo.temp <- prune_samples(x = phyloseq.object,samples = data[,which(colnames(data) == ID.col)]) #su
  phylo.temp  = filter_taxa(phylo.temp , function(x) mean(x) > 0, TRUE)

  data$order <- paste(data[,which(colnames(data) == order1)], #make ordering column
                      data[,which(colnames(data) == order2)],
                      data[,which(colnames(data) == order3)],
                      data[,which(colnames(data) == order4)])

  data.i <- data.i[match(sample_data(phylo.temp)$sample.ID,data.i$sample.ID),]

  sample_data(phylo.temp)$grouping <- data$order

  data <- data[order(data$order),] # order data

  plot <- plot_heatmap(phylo.temp,sample.order = data[,which(colnames(data) == ID.col)],sample.label =


  print(plot)
}

phylo.heat.loop <- function(phyloseq.object.list = phylo.list, data, ID.col = "sample.ID", order1 = NUL

  for(i in 1:length(phyloseq.object.list )){

    phyloseq.object <- phyloseq.object.list[[i]]
    taxa.label <- taxa.label.list[[i]]

    data.i <- data

    phylo.temp <- prune_samples(x = phyloseq.object,samples = data.i[,which(colnames(data.i) == ID.col)
    phylo.temp  = filter_taxa(phylo.temp , function(x) mean(x) > 0, TRUE)

    data.i$order <- paste(data.i[,which(colnames(data.i) == order1)], #make ordering column
                          data.i[,which(colnames(data.i) == order2)],
                          data.i[,which(colnames(data.i) == order3)],
                          data.i[,which(colnames(data.i) == order4)])


    data.i <- data.i[match(sample_data(phylo.temp)$sample.ID,data.i$sample.ID),]

    sample_data(phylo.temp)$grouping <- data.i$order

    data.i <- data.i[order(data.i$order),] # order data.i

    plot <- plot_heatmap(phylo.temp,sample.order = data.i[,which(colnames(data.i) == ID.col)],sample.la
```

```
    print(plot)}

}
```

Here taxonomic heat maps are generated for turtle species/collection site and collection site/turtle species combinations, across the following taxonomic levels: ASV, genus, family, order, class and phylum.

```
phylo.heat.loop(phylo.list, data = carapace , ID.col = "sample.ID", order1 = "Species", order2="Site",t
```

```
## Warning: Transformation introduced infinite values in discrete y-axis
```

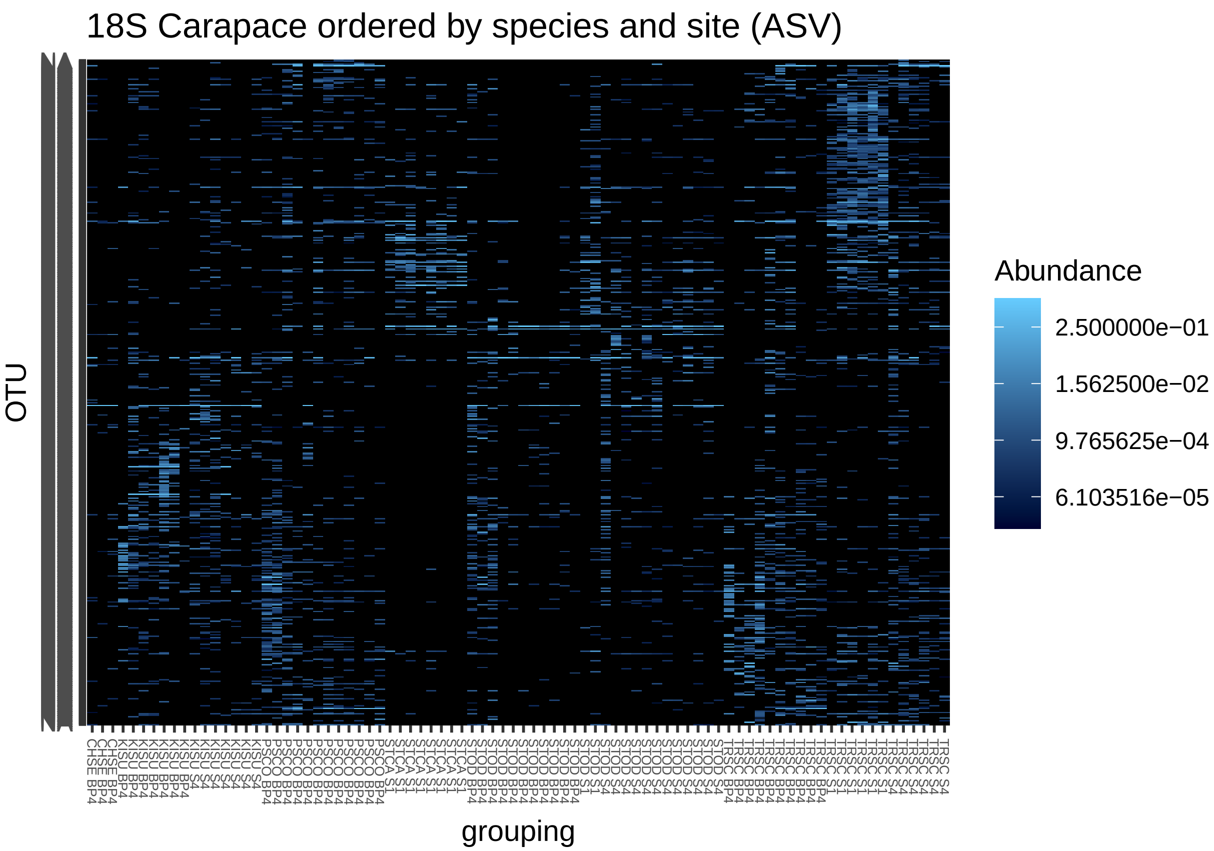


```
## Warning: Transformation introduced infinite values in discrete y-axis
```

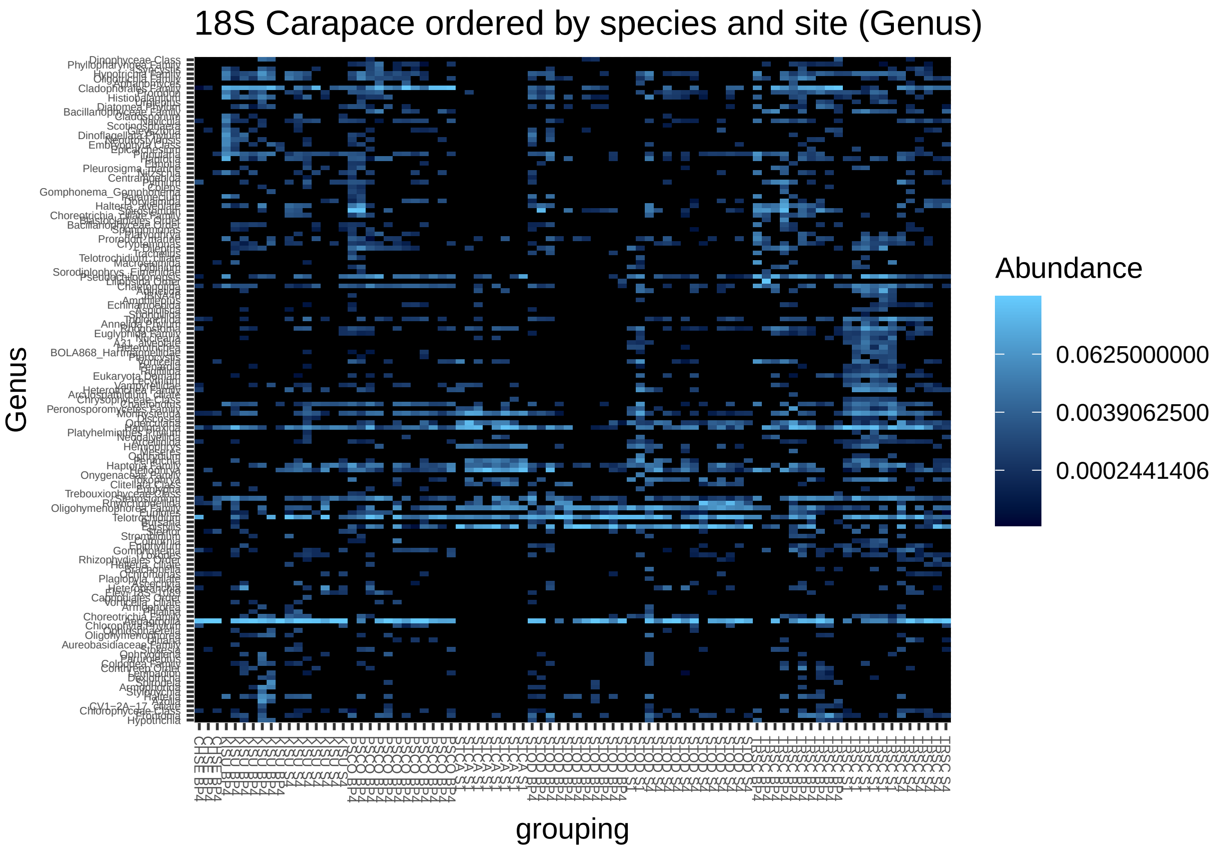


```
## Warning: Transformation introduced infinite values in discrete y-axis
```

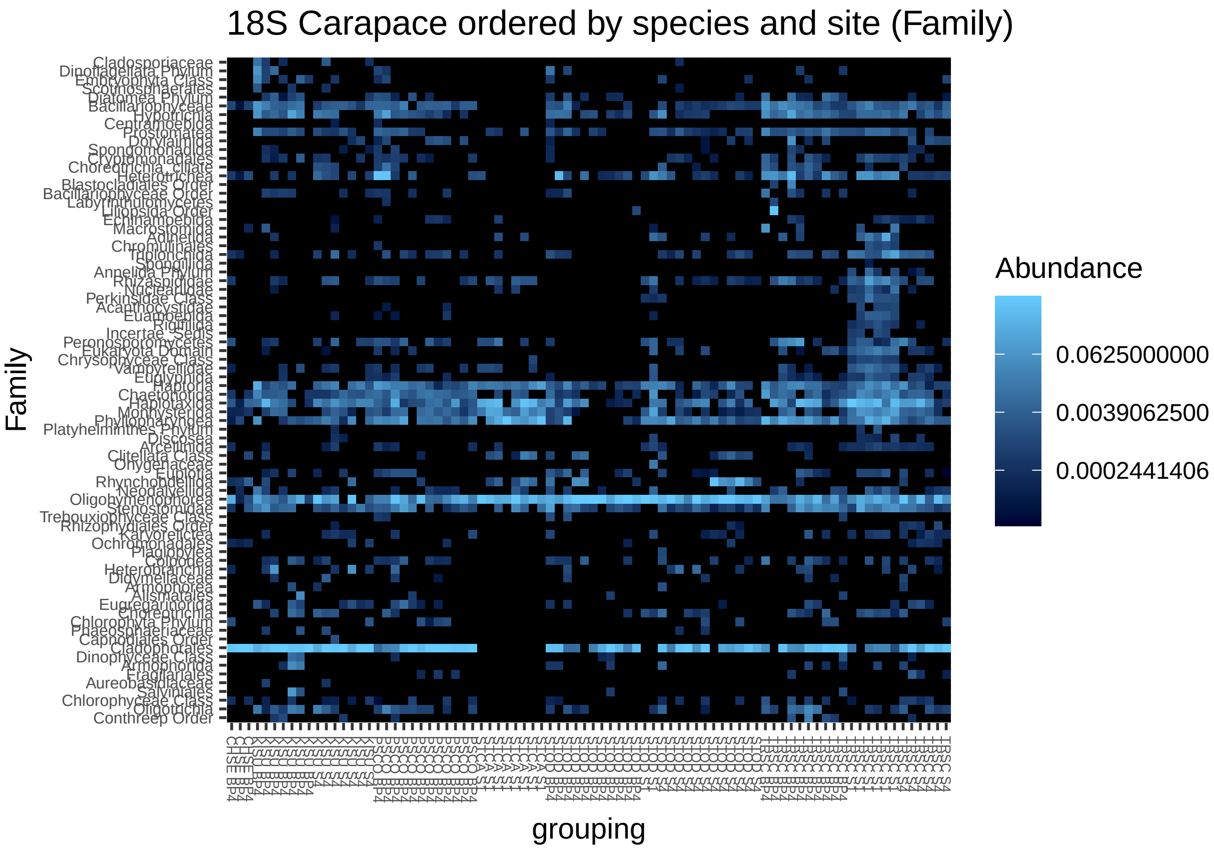


```
## Warning: Transformation introduced infinite values in discrete y-axis
```

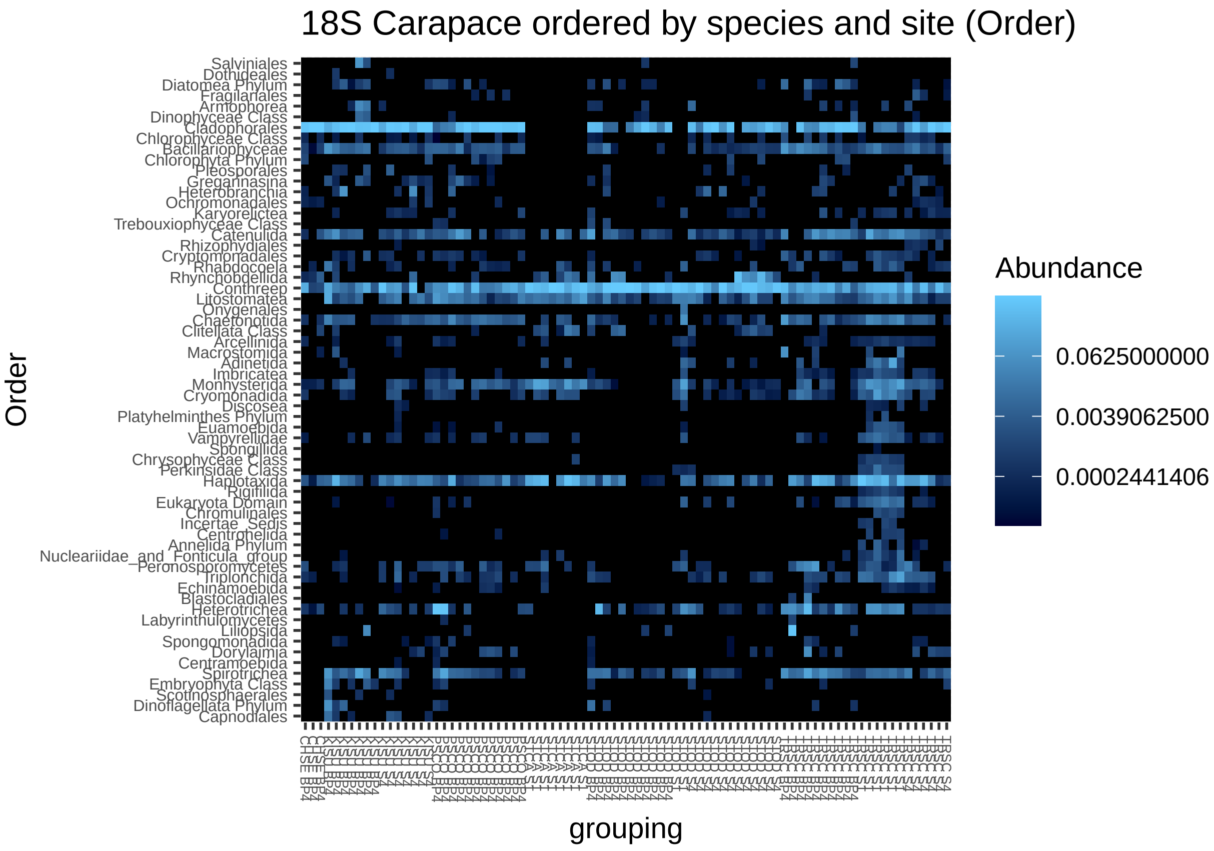


```
## Warning: Transformation introduced infinite values in discrete y-axis
```

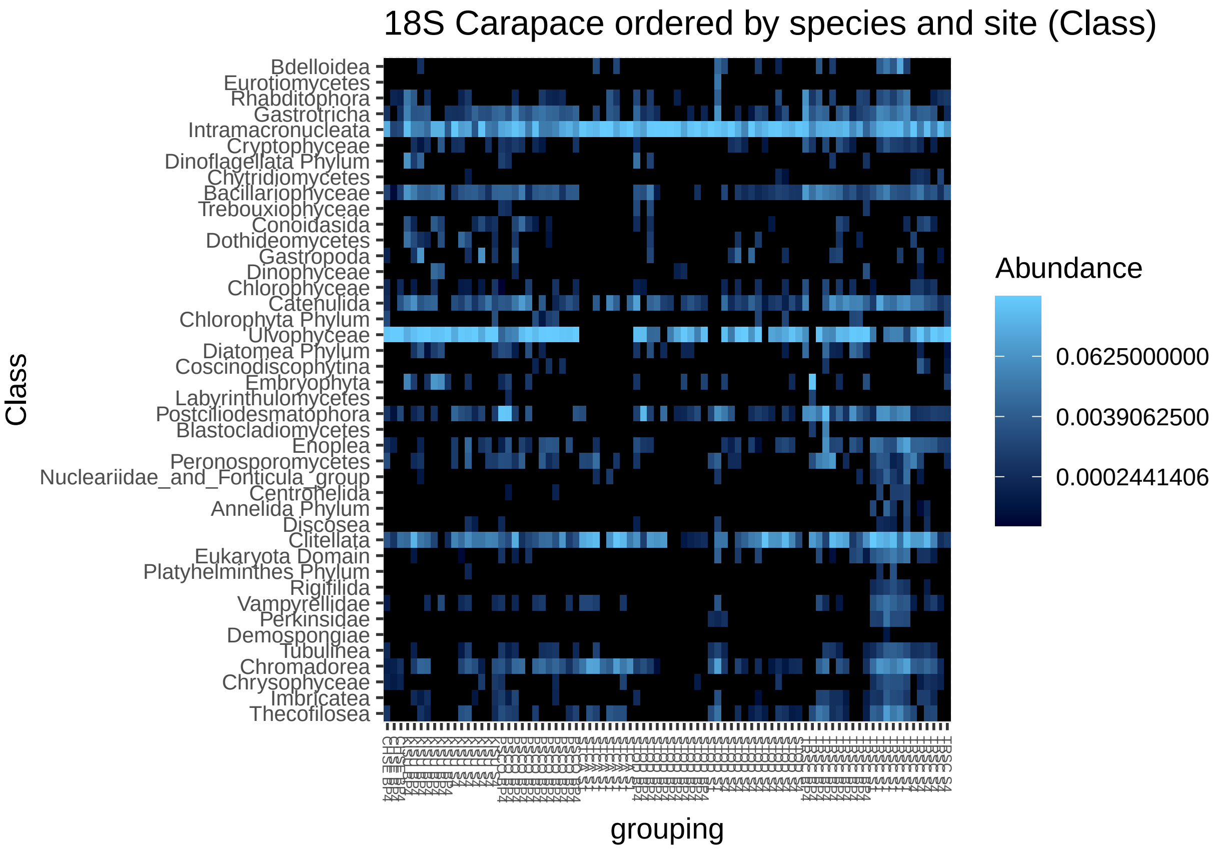


```
## Warning: Transformation introduced infinite values in discrete y-axis
```

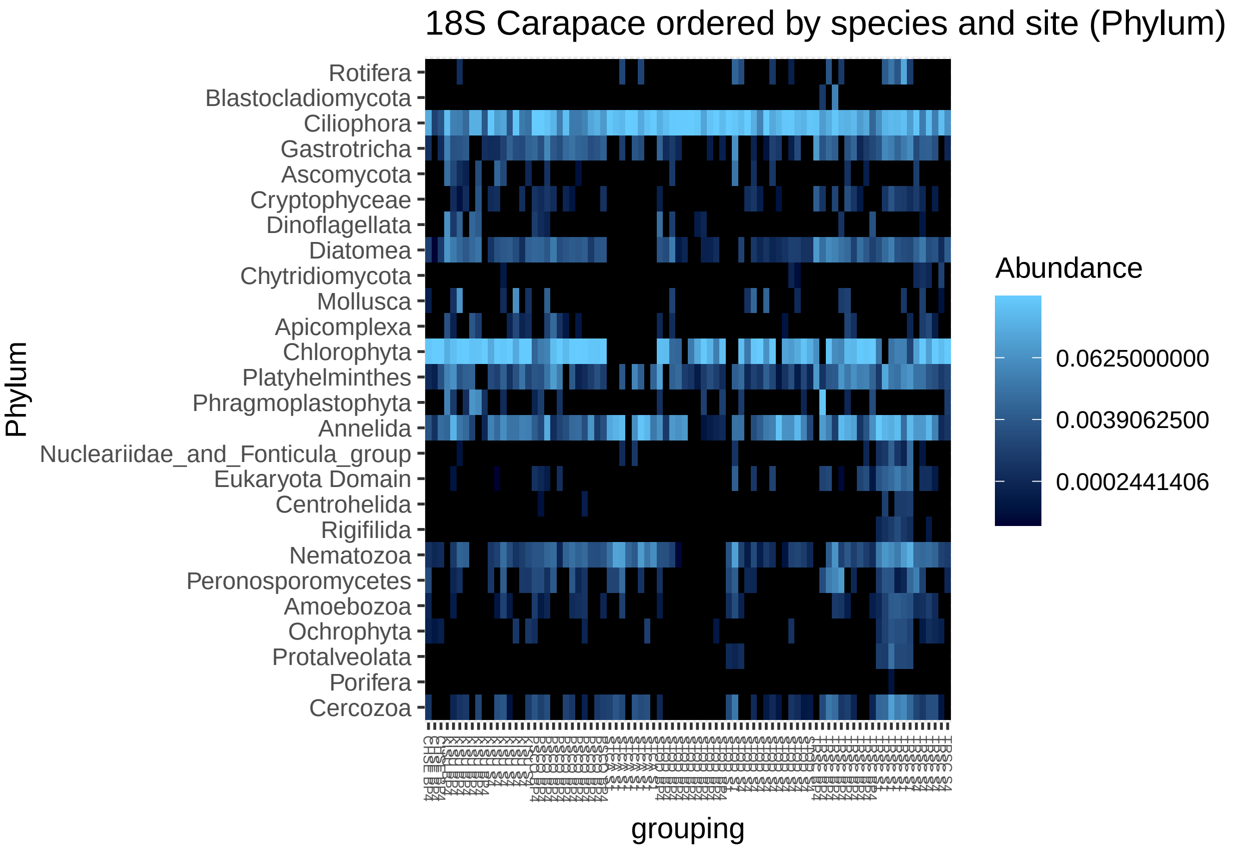


```
phylo.heat.loop(phylo.list, data = carapace , ID.col = "sample.ID", order1 = "Site", order2="Species",t
```

```
## Warning: Transformation introduced infinite values in discrete y-axis
```

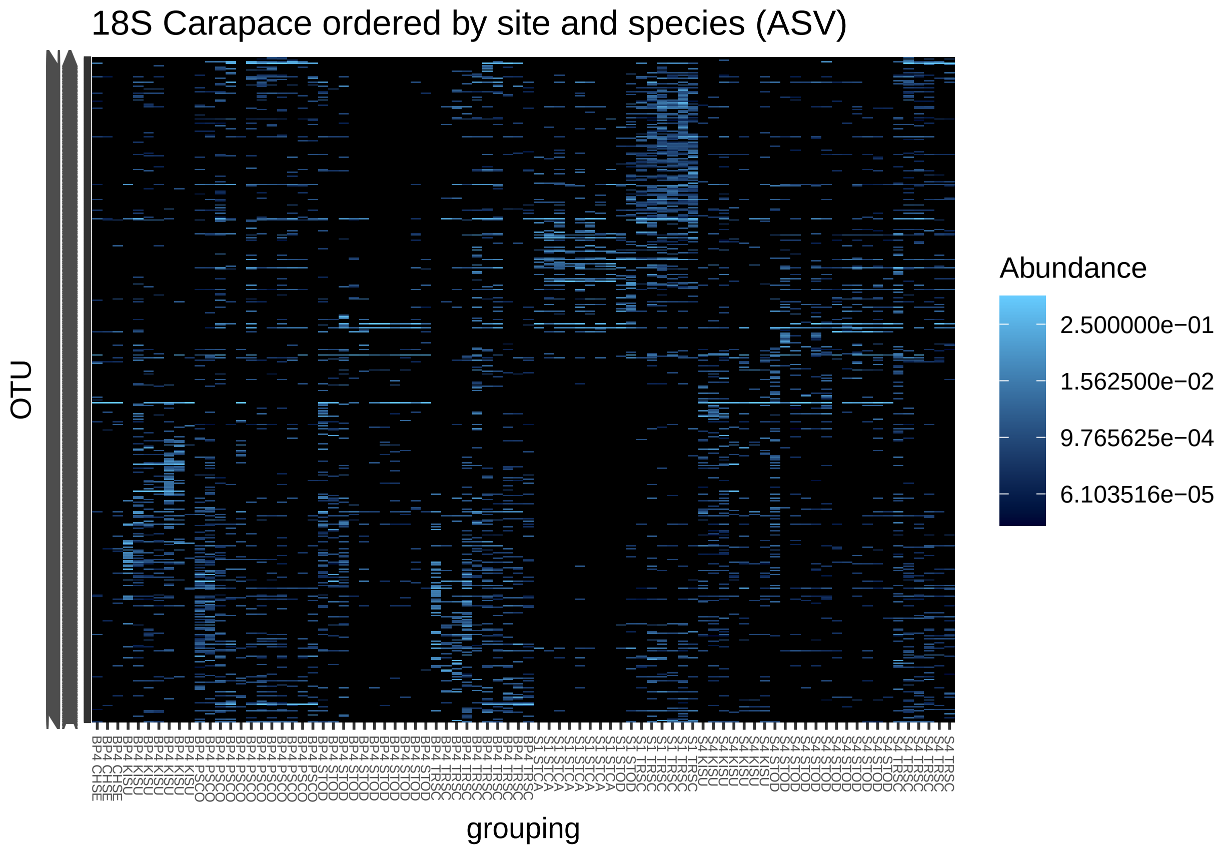


```
## Warning: Transformation introduced infinite values in discrete y-axis
```

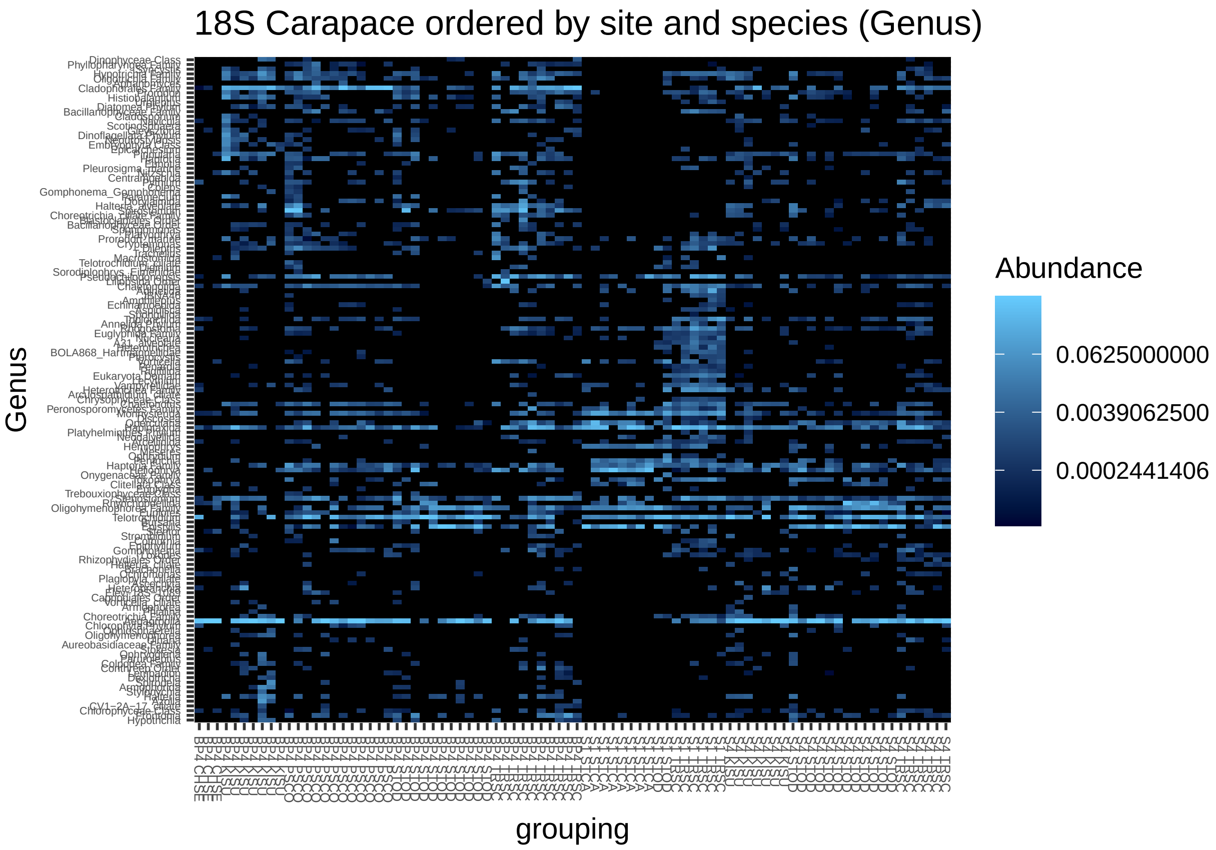


```
## Warning: Transformation introduced infinite values in discrete y-axis
```

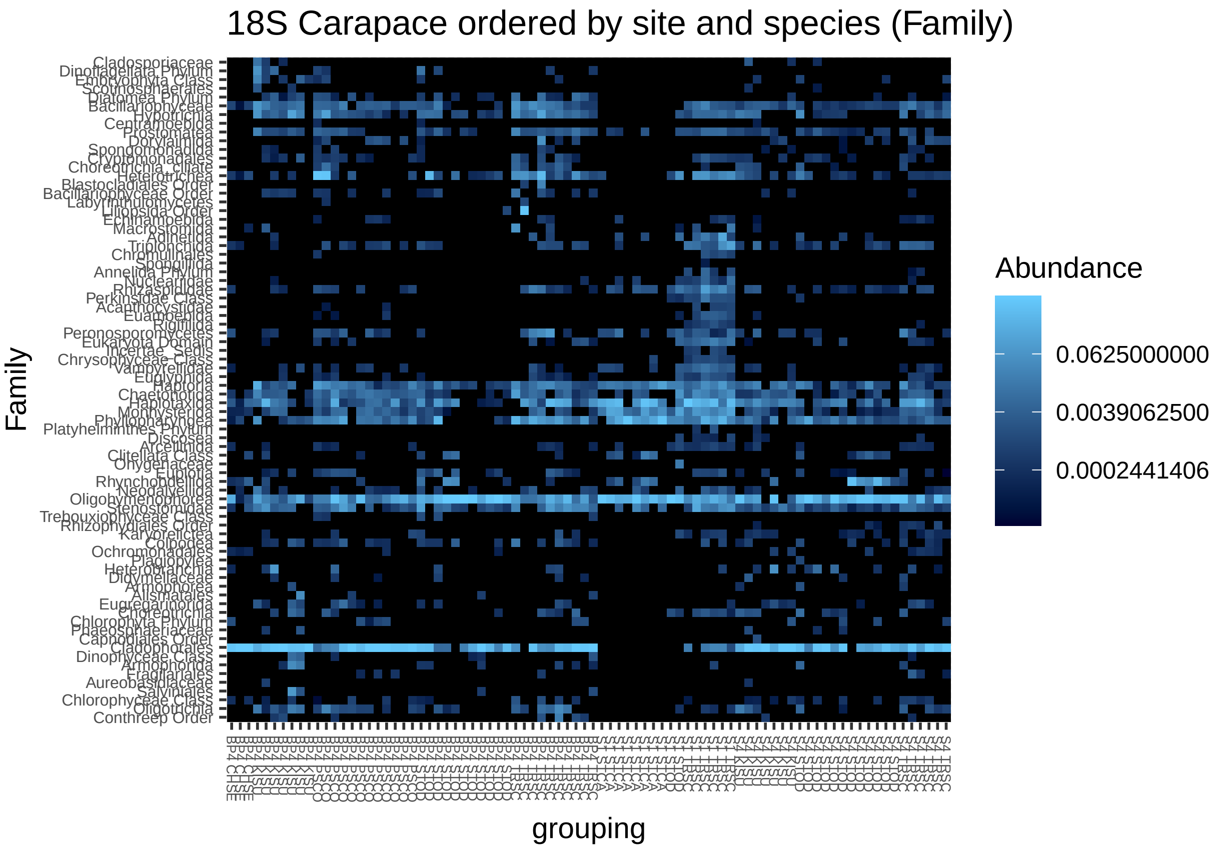


```
## Warning: Transformation introduced infinite values in discrete y-axis
```

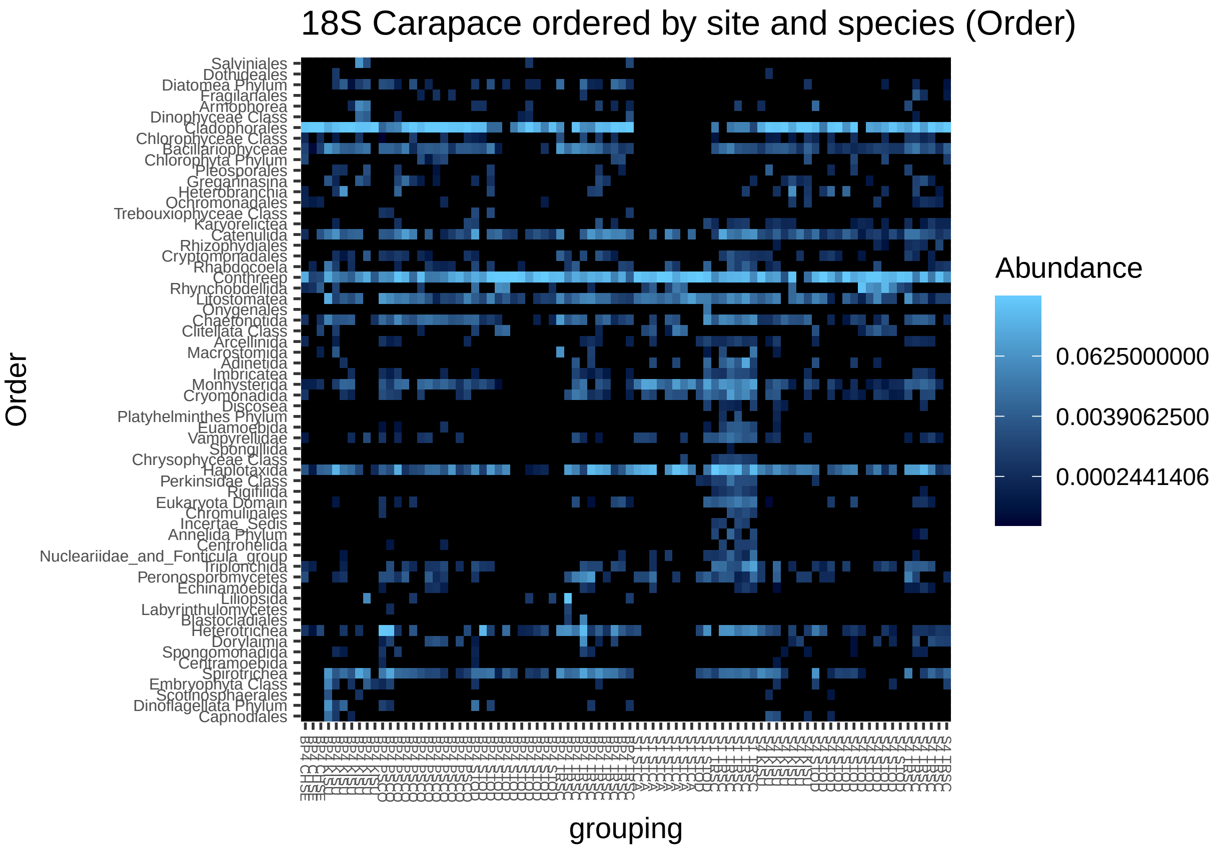


```
## Warning: Transformation introduced infinite values in discrete y-axis
```

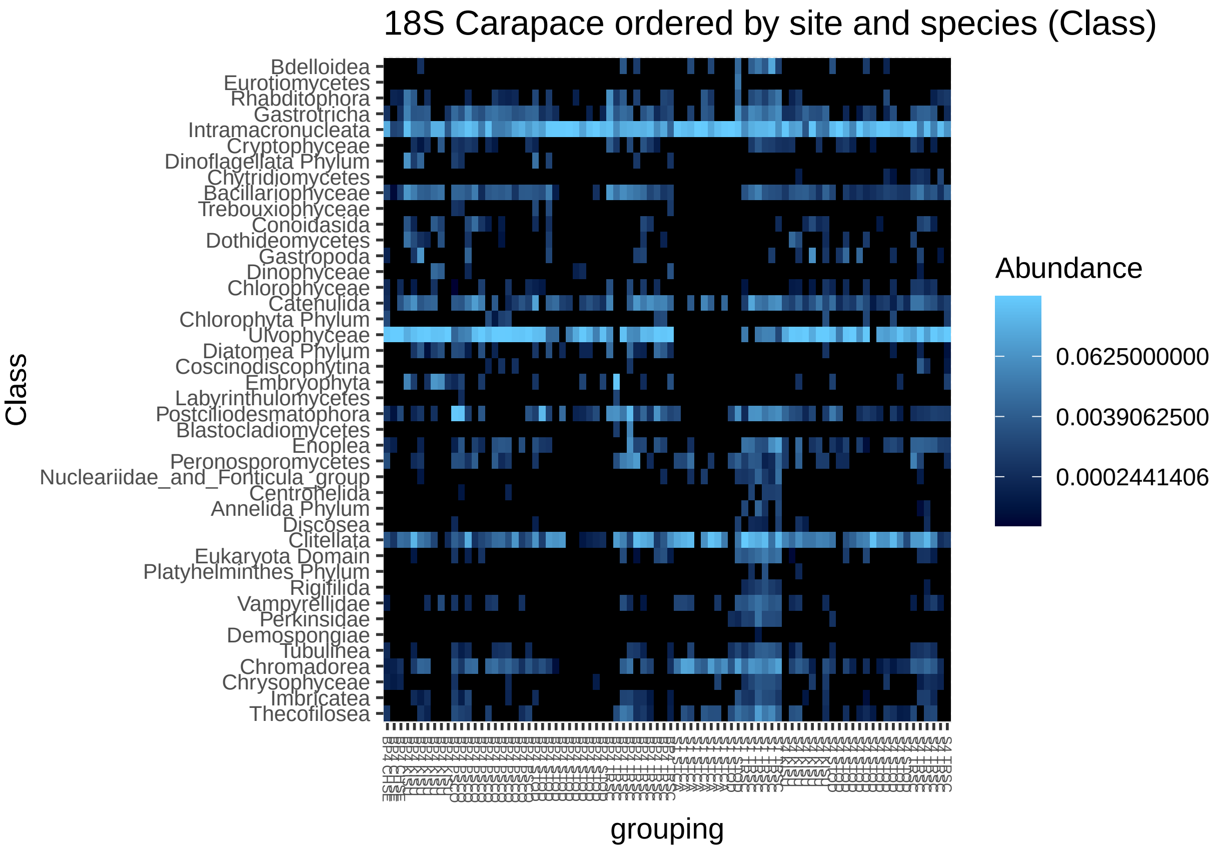


```
## Warning: Transformation introduced infinite values in discrete y-axis
```

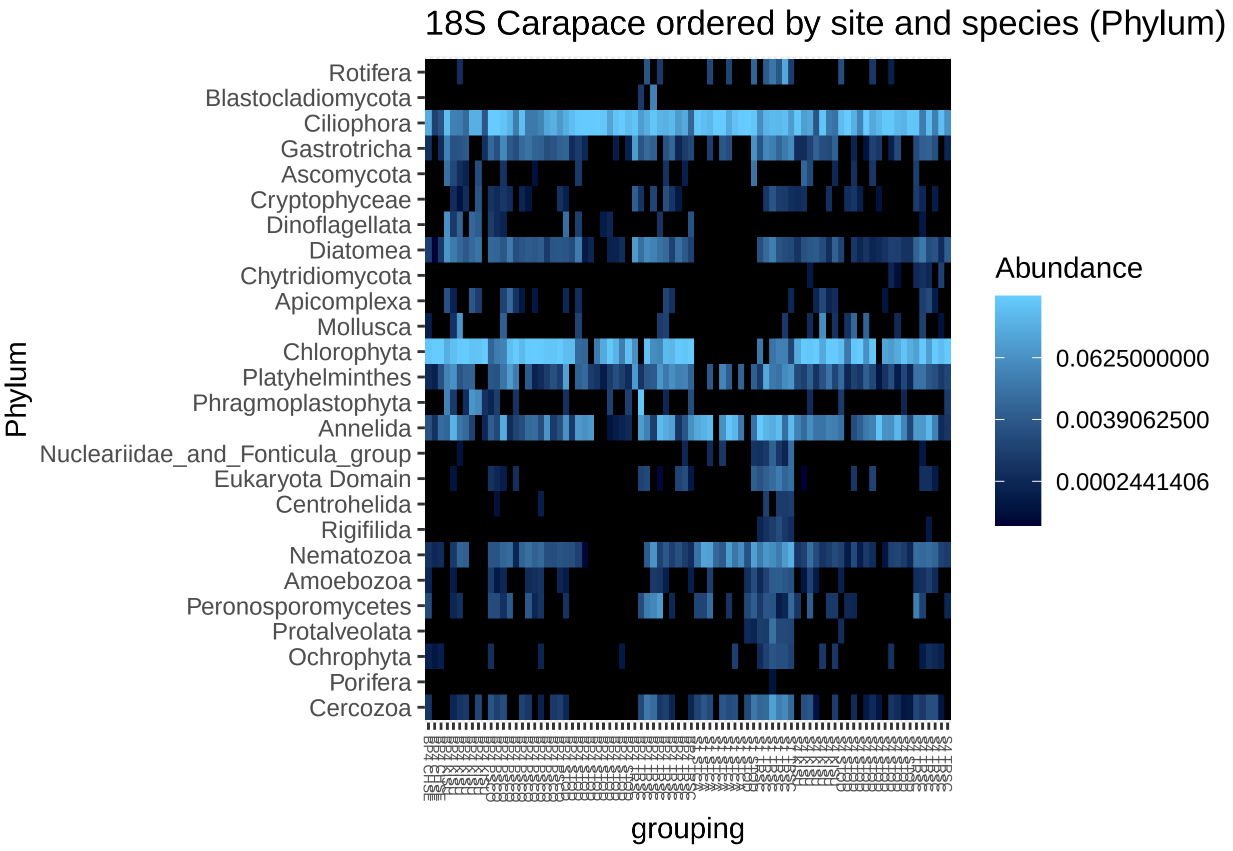

Beta diversity comparisons

The sections below feature beta diversity analyses. First, beta diversity matrices are calculated and stored in a data frame.

```r
# Calculate distance matrices in vegan
carapace_bray <- phyloseq::distance(phylo_carapace, method = "bray")
carapace_jacc <- phyloseq::distance(phylo_carapace, method = "jaccard")
carapace_uni <- phyloseq::distance(phylo_carapace, method = "unifrac")
carapace_wuni <- phyloseq::distance(phylo_carapace, method = "wunifrac")

# make a data frame from the sample_data
sampledf <- data.frame(sample_data(phylo_carapace))
```

Beta dispersion testing by species and site

The below commands are used to generate comparisons of beta dispersion by species and by site, and to generate ordination plots for beta diversity by species and by site.

```r
betadisper_bray_species <- betadisper(carapace_bray, sampledf$Species)
betadisper_jacc_species <- betadisper(carapace_jacc, sampledf$Species)
betadisper_uni_species <- betadisper(carapace_uni, sampledf$Species)
betadisper_wuni_species <- betadisper(carapace_wuni, sampledf$Species)
```

```
## Warning in betadisper(carapace_wuni, sampledf$Species): some squared distances
```

```
## are negative and changed to zero
```

```r
betadisper_bray_site <- betadisper(carapace_bray, sampledf$Site)
betadisper_jacc_site <- betadisper(carapace_jacc, sampledf$Site)
betadisper_uni_site <- betadisper(carapace_uni, sampledf$Site)
betadisper_wuni_site <- betadisper(carapace_wuni, sampledf$Site)
```

```
## Warning in betadisper(carapace_wuni, sampledf$Site): some squared distances are
## negative and changed to zero
```

```r
plot(betadisper_bray_species, hull = FALSE, ellipse = TRUE)
```

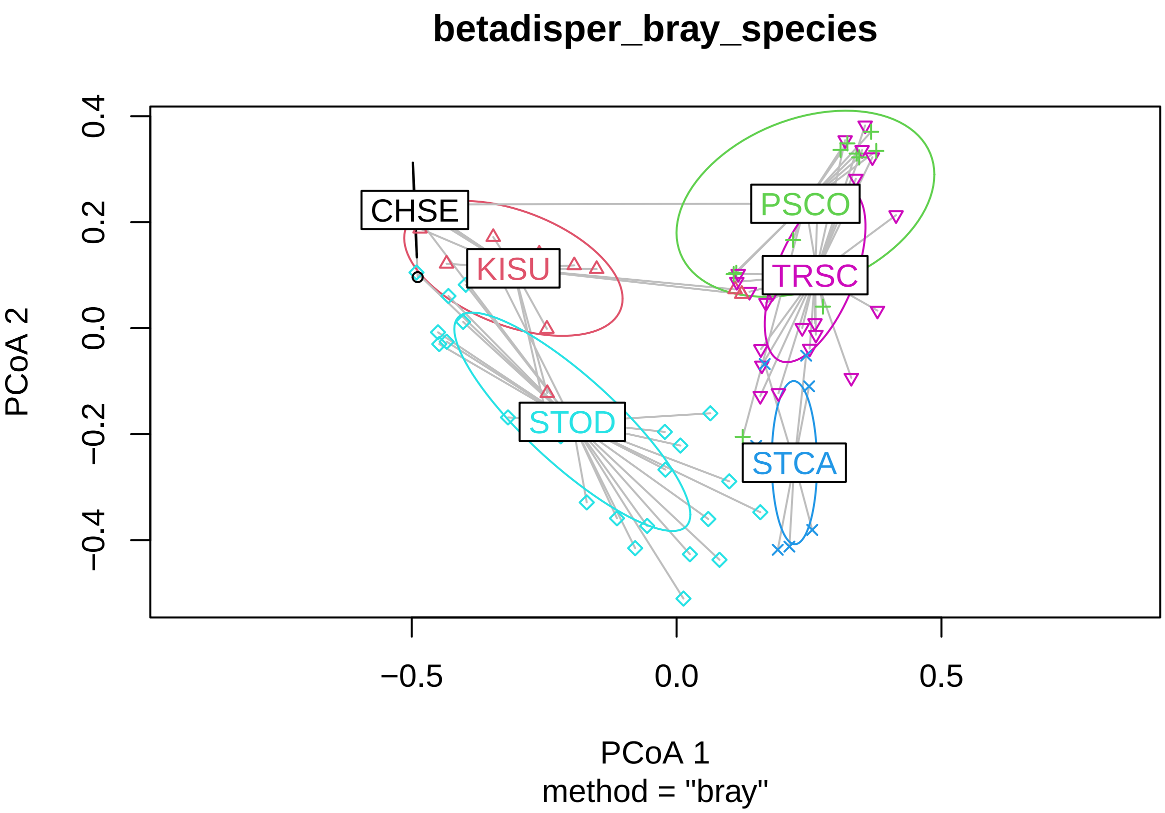


```r
print(betadisper_bray_species, digits = max(3, getOption("digits") - 3), neigen = 2)
```

```
##
##  Homogeneity of multivariate dispersions
##
## Call: betadisper(d = carapace_bray, group = sampledf$Species)
##
## No. of Positive Eigenvalues: 53
## No. of Negative Eigenvalues: 30
##
## Average distance to median:
##    CHSE    KISU    PSCO    STCA    STOD    TRSC
## 0.08685 0.48962 0.54000 0.44747 0.48532 0.59869
##
```

```
## Eigenvalues for PCoA axes:
## (Showing 2 of 83 eigenvalues)
## PCoA1 PCoA2
## 7.316 4.510
```

```r
plot(betadisper_bray_site, hull = FALSE, ellipse = TRUE)
```

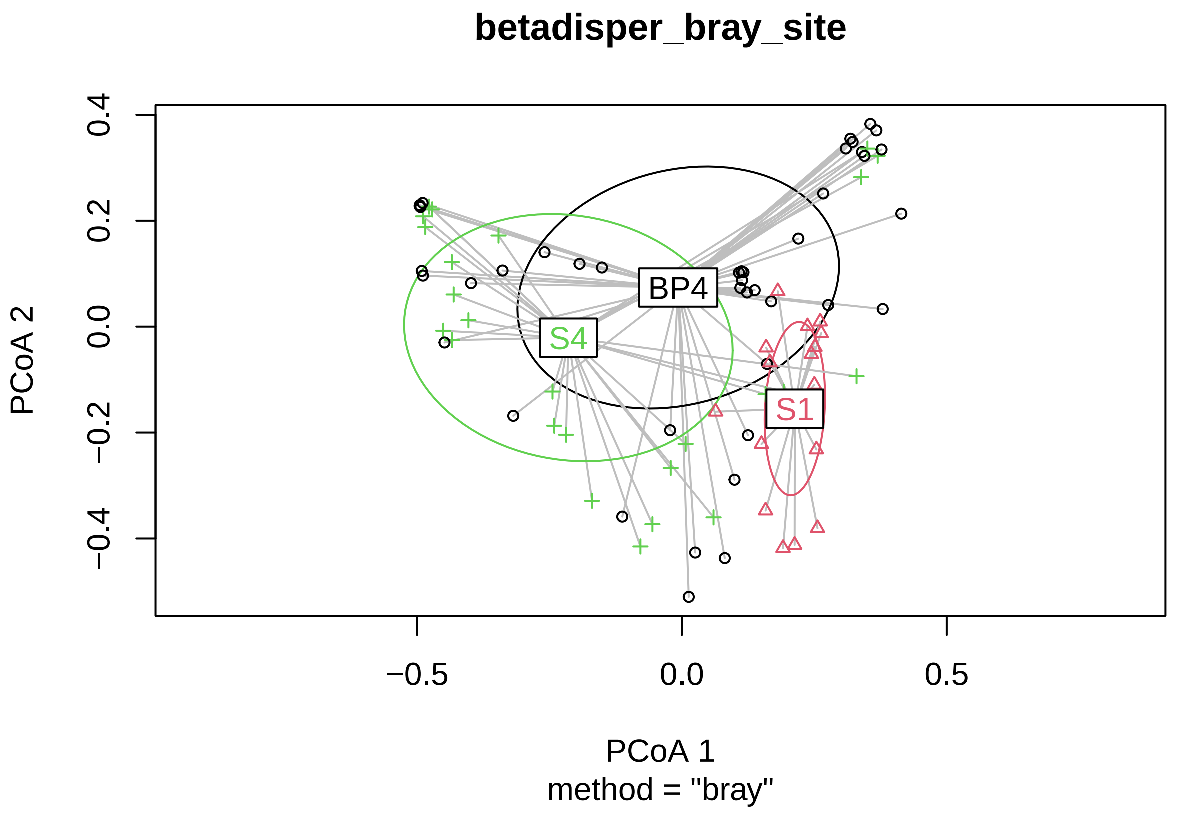


```r
print(betadisper_bray_site, digits = max(3, getOption("digits") - 3), neigen = 2)
```

```
##
##  Homogeneity of multivariate dispersions
##
## Call: betadisper(d = carapace_bray, group = sampledf$Site)
##
## No. of Positive Eigenvalues: 53
## No. of Negative Eigenvalues: 30
##
## Average distance to median:
##    BP4     S1     S4
## 0.6219 0.5434 0.5343
##
## Eigenvalues for PCoA axes:
## (Showing 2 of 83 eigenvalues)
## PCoA1 PCoA2
## 7.316 4.510
```

```r
plot(betadisper_jacc_species, hull = FALSE, ellipse = TRUE)
```

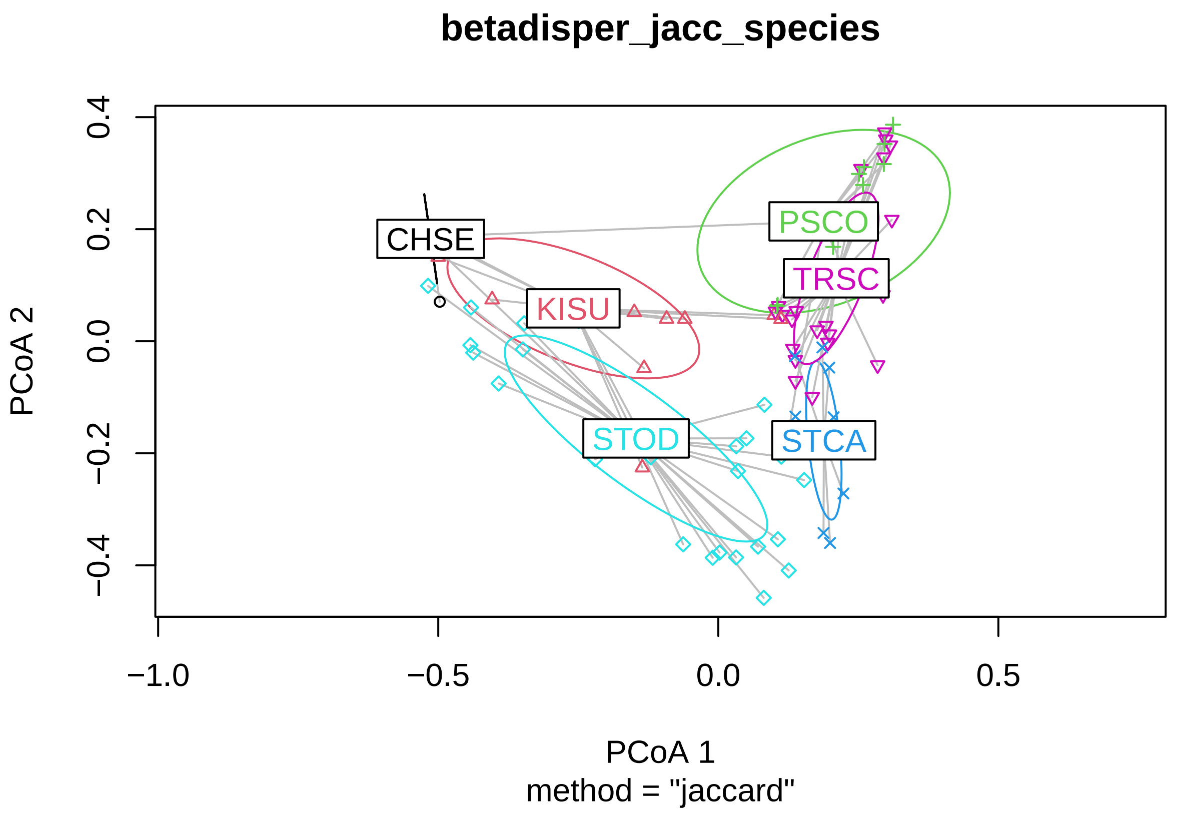


```r
print(betadisper_jacc_species, digits = max(3, getOption("digits") - 3), neigen = 2)
```

```
##
##  Homogeneity of multivariate dispersions
##
## Call: betadisper(d = carapace_jacc, group = sampledf$Species)
##
## No. of Positive Eigenvalues: 75
## No. of Negative Eigenvalues: 8
##
## Average distance to median:
##   CHSE   KISU   PSCO   STCA   STOD   TRSC
## 0.1413 0.5517 0.5843 0.5297 0.5555 0.6338
##
## Eigenvalues for PCoA axes:
## (Showing 2 of 83 eigenvalues)
## PCoA1 PCoA2
## 6.035 3.726
```

```r
plot(betadisper_jacc_site, hull = FALSE, ellipse = TRUE)
```

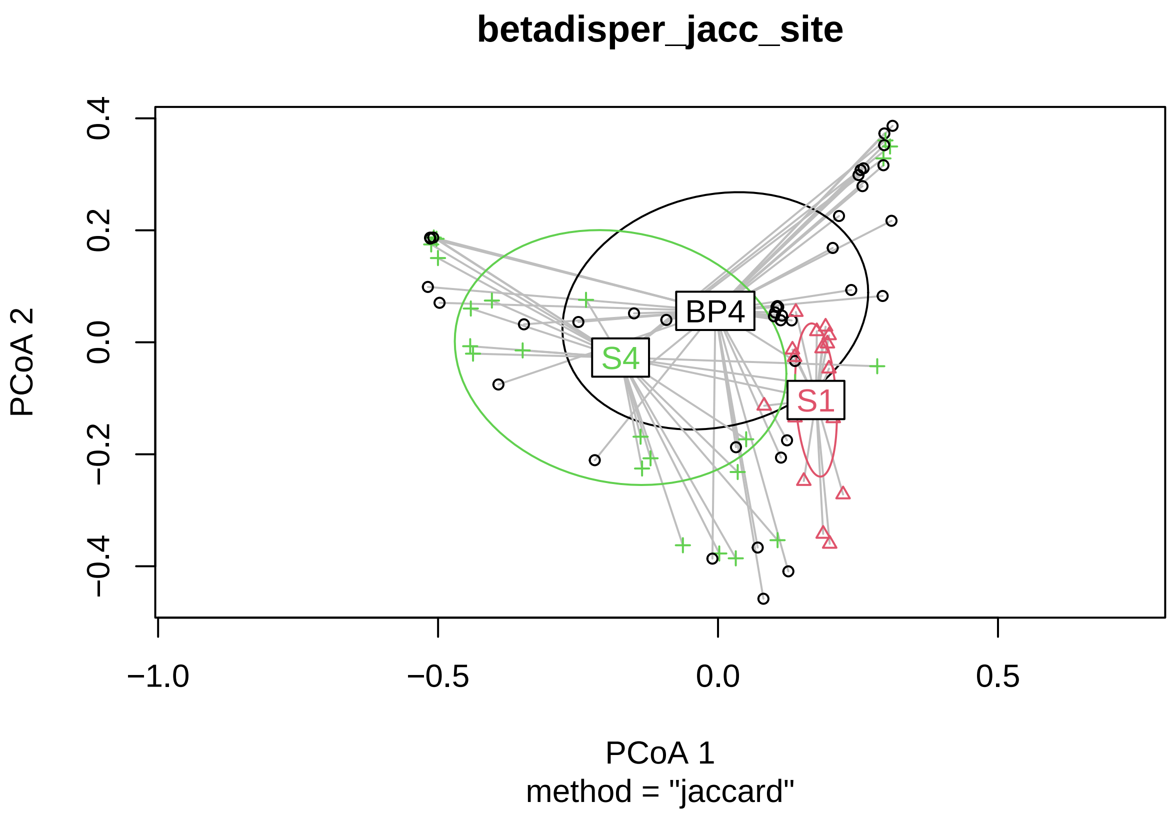


```r
print(betadisper_jacc_site, digits = max(3, getOption("digits") - 3), neigen = 2)
```

```
##
##  Homogeneity of multivariate dispersions
##
## Call: betadisper(d = carapace_jacc, group = sampledf$Site)
##
## No. of Positive Eigenvalues: 75
## No. of Negative Eigenvalues: 8
##
## Average distance to median:
##    BP4     S1     S4
## 0.6470 0.5996 0.5856
##
## Eigenvalues for PCoA axes:
## (Showing 2 of 83 eigenvalues)
## PCoA1 PCoA2
## 6.035 3.726
```

```r
plot(betadisper_uni_species, hull = FALSE, ellipse = TRUE)
```

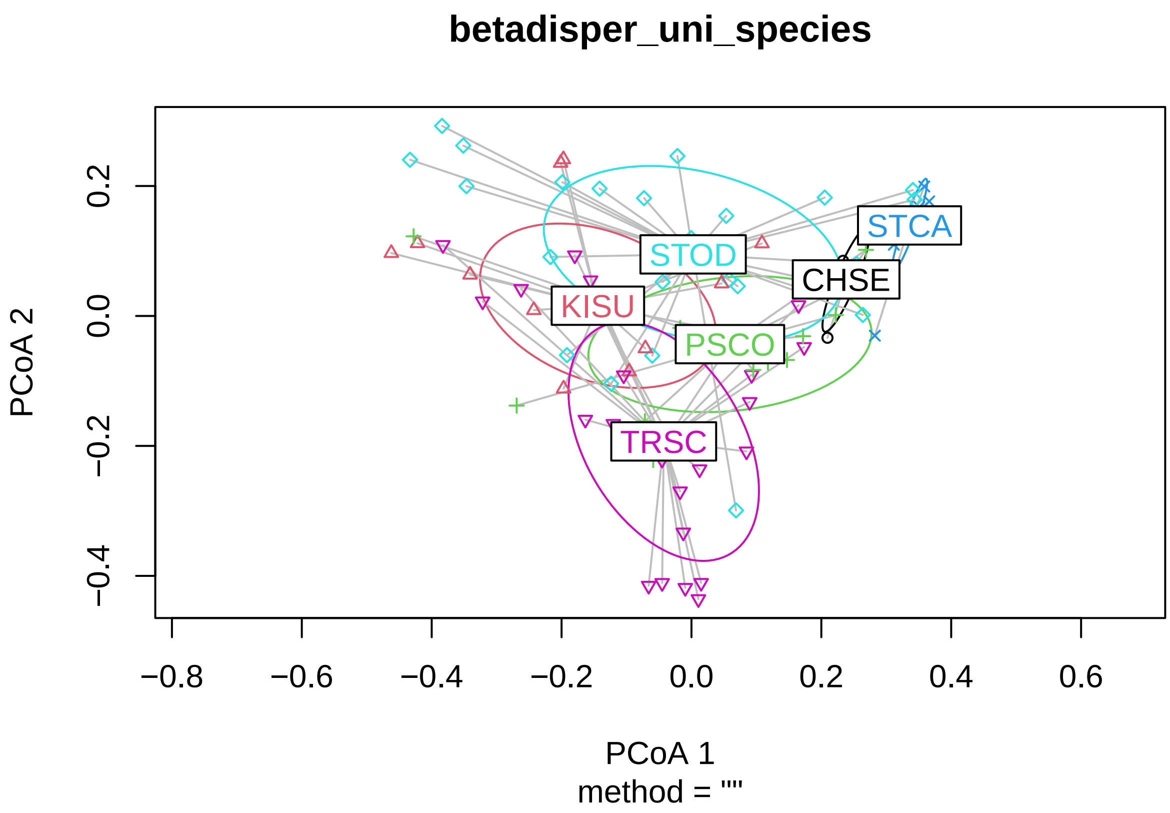


```r
print(betadisper_uni_species, digits = max(3, getOption("digits") - 3), neigen = 2)
```

```
##
##  Homogeneity of multivariate dispersions
##
## Call: betadisper(d = carapace_uni, group = sampledf$Species)
##
## No. of Positive Eigenvalues: 71
## No. of Negative Eigenvalues: 12
##
## Average distance to median:
##   CHSE   KISU   PSCO   STCA   STOD   TRSC
## 0.3396 0.5525 0.5038 0.3364 0.5612 0.5368
##
## Eigenvalues for PCoA axes:
## (Showing 2 of 83 eigenvalues)
## PCoA1 PCoA2
## 4.130 2.587
```

```r
plot(betadisper_uni_site, hull = FALSE, ellipse = TRUE)
```

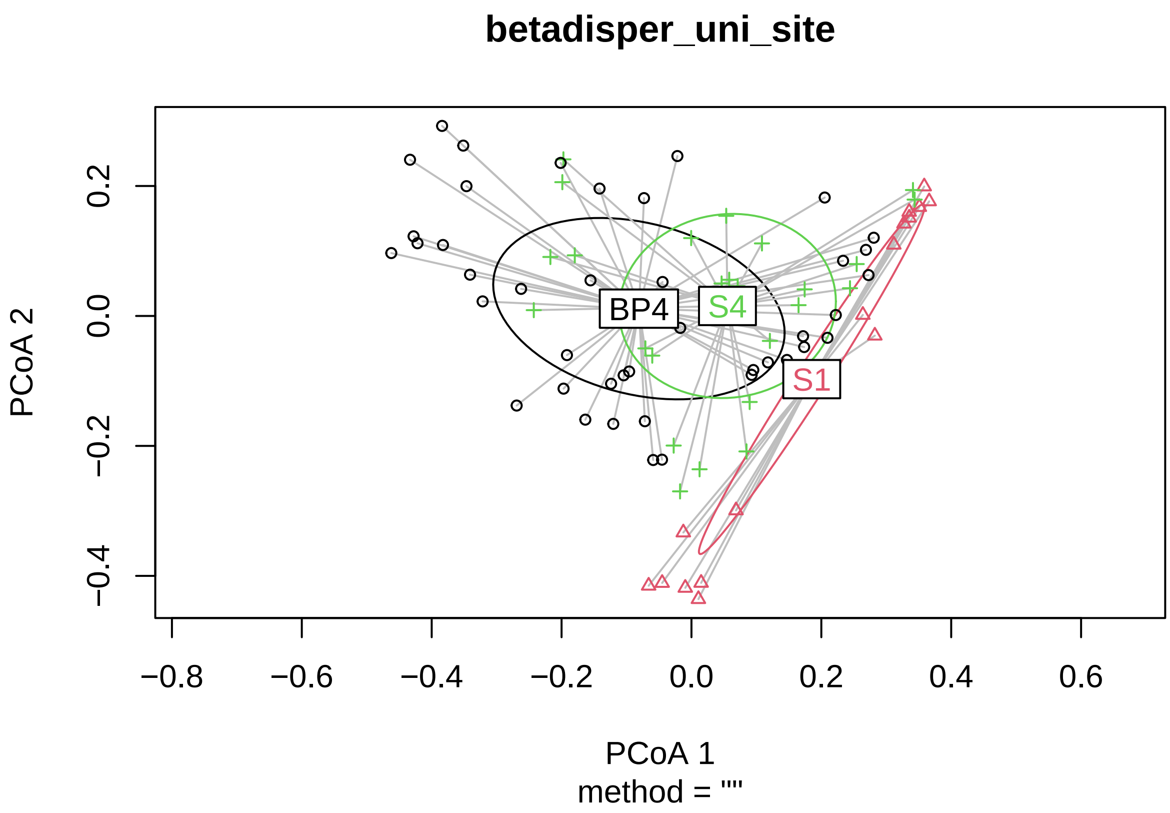


```r
print(betadisper_uni_site, digits = max(3, getOption("digits") - 3), neigen = 2)
```

```
##
##  Homogeneity of multivariate dispersions
##
## Call: betadisper(d = carapace_uni, group = sampledf$Site)
##
## No. of Positive Eigenvalues: 71
## No. of Negative Eigenvalues: 12
##
## Average distance to median:
##    BP4     S1     S4
## 0.5809 0.4767 0.5313
##
## Eigenvalues for PCoA axes:
## (Showing 2 of 83 eigenvalues)
## PCoA1 PCoA2
## 4.130 2.587
```

```r
plot(betadisper_wuni_species, hull = FALSE, ellipse = TRUE)
```

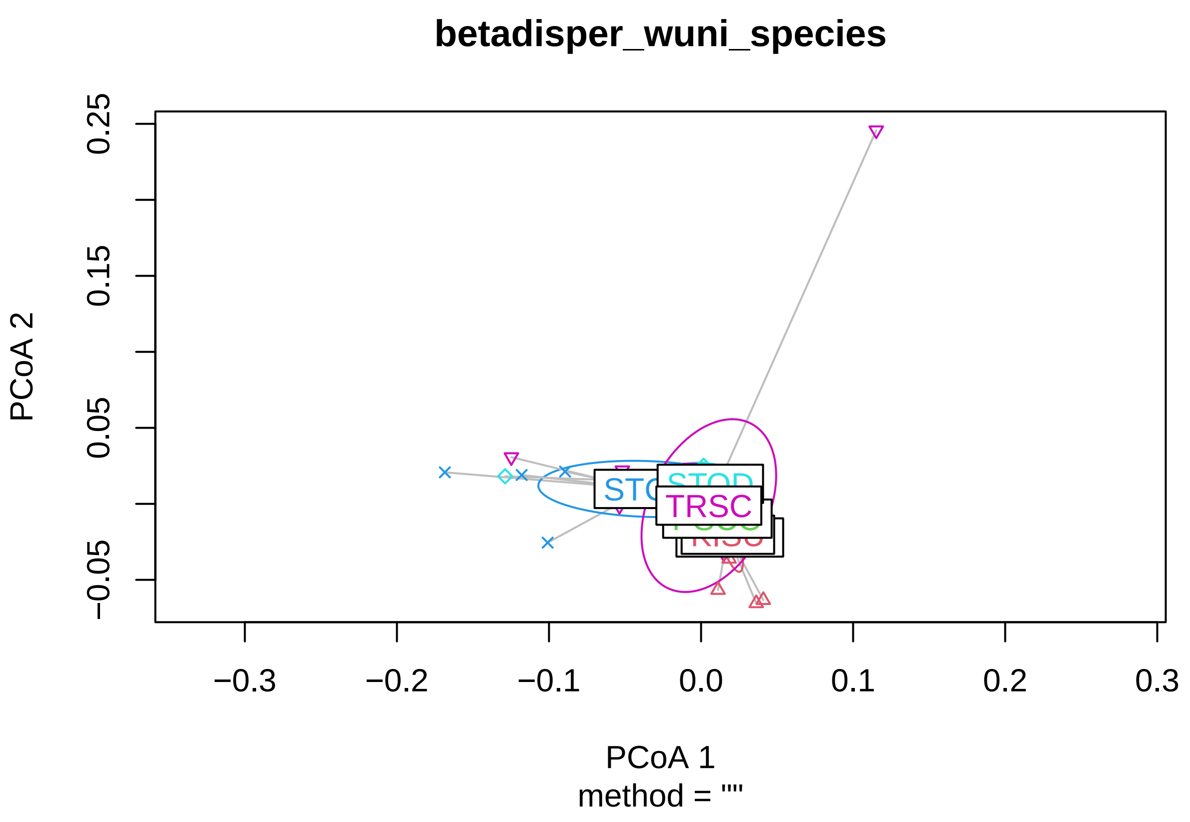


```r
print(betadisper_wuni_species, digits = max(3, getOption("digits") - 3), neigen = 2)
```

```
##
##  Homogeneity of multivariate dispersions
##
## Call: betadisper(d = carapace_wuni, group = sampledf$Species)
##
## No. of Positive Eigenvalues: 52
## No. of Negative Eigenvalues: 31
##
## Average distance to median:
##      CHSE      KISU      PSCO      STCA      STOD      TRSC
## 0.0006481 0.0335728 0.0114316 0.0545288 0.0120358 0.0449198
##
## Eigenvalues for PCoA axes:
## (Showing 2 of 83 eigenvalues)
##   PCoA1   PCoA2
## 0.12924 0.09851
```

```r
plot(betadisper_wuni_site, hull = FALSE, ellipse = TRUE)
```

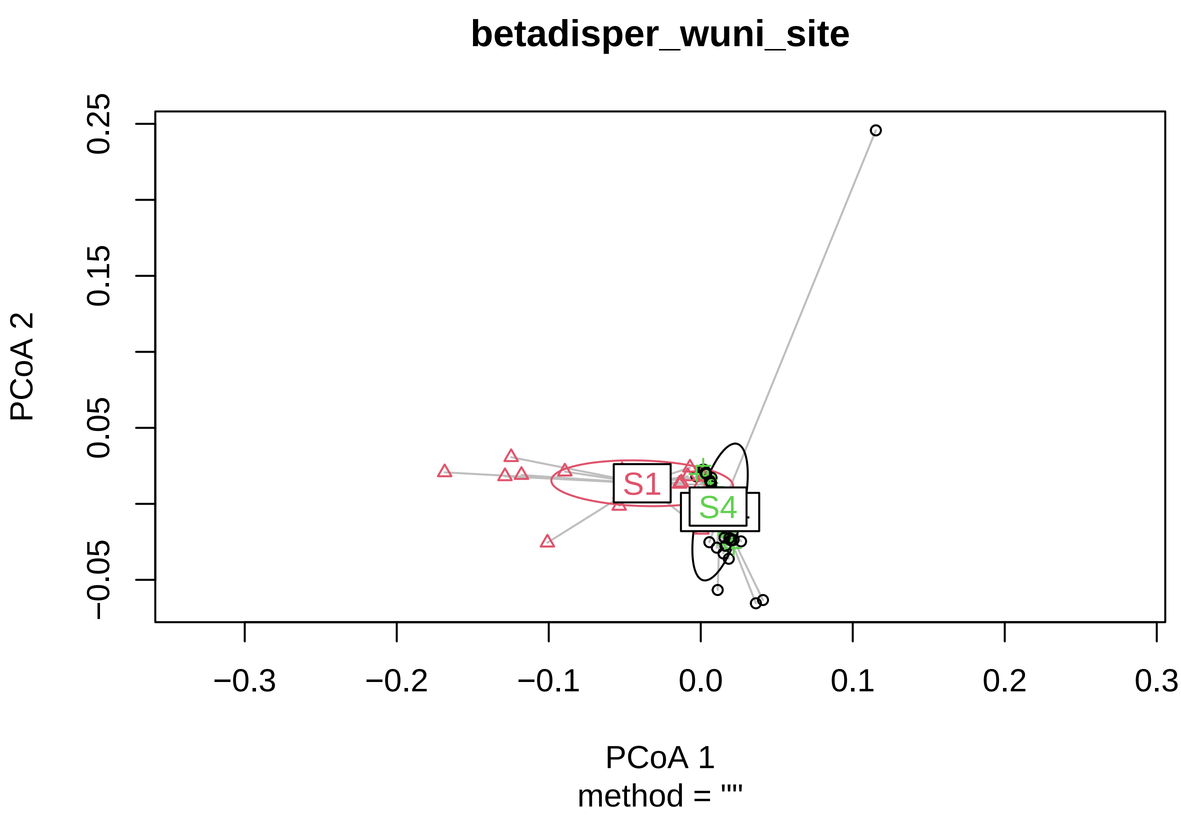


```
print(betadisper_wuni_site, digits = max(3, getOption("digits") - 3), neigen = 2)
```

```
##
##  Homogeneity of multivariate dispersions
##
## Call: betadisper(d = carapace_wuni, group = sampledf$Site)
##
## No. of Positive Eigenvalues: 52
## No. of Negative Eigenvalues: 31
##
## Average distance to median:
##     BP4      S1      S4
## 0.02506 0.05427 0.01074
##
## Eigenvalues for PCoA axes:
## (Showing 2 of 83 eigenvalues)
##   PCoA1   PCoA2
## 0.12924 0.09851
```

In the following, ANOVA is used to test for significant differences in beta dispersion by site and by species, across four different beta diversity metrics (Bray, Jaccsard, UniFrac, weighted UniFrac). If results are significant, we can reject the null hypothesis that the species have the same dispersions. Pairwise comparisons with Tukey-corrected p-values are also presented.

```
betadisper_bray_species.anova <- anova(betadisper_bray_species, permutations = 999)
betadisper_bray_species.anova
```

```
## Analysis of Variance Table
##
## Response: Distances
##           Df  Sum Sq  Mean Sq F value   Pr(>F)
## Groups     5 0.77178 0.154355  9.9621 2.14e-07 ***
## Residuals 78 1.20855 0.015494
## ---
## Signif. codes:  0 '***' 0.001 '**' 0.01 '*' 0.05 '.' 0.1 ' ' 1
```

```
# test significance for beta dispersion between each group with p-values adjusted by Tukey HSD
betadisper_bray_species.TukeyHSD <- TukeyHSD(betadisper_bray_species)
betadisper_bray_species.TukeyHSD
```

```
##   Tukey multiple comparisons of means
##     95% family-wise confidence level
##
## Fit: aov(formula = distances ~ group, data = df)
##
## $group
##                   diff          lwr        upr     p adj
## KISU-CHSE  0.402774977  0.171392610 0.63415734 0.0000355
## PSCO-CHSE  0.453151916  0.218391526 0.68791231 0.0000038
## STCA-CHSE  0.360623167  0.114404392 0.60684194 0.0007289
## STOD-CHSE  0.398470125  0.176252333 0.62068792 0.0000194
## TRSC-CHSE  0.511837717  0.288002468 0.73567297 0.0000000
## PSCO-KISU  0.050376939 -0.092697640 0.19345152 0.9068548
## STCA-KISU -0.042151811 -0.203339835 0.11903621 0.9726873
## STOD-KISU -0.004304852 -0.125707659 0.11709795 0.9999983
## TRSC-KISU  0.109062740 -0.015275964 0.23340144 0.1190705
## STCA-PSCO -0.092528750 -0.258529414 0.07347191 0.5824462
## STOD-PSCO -0.054681792 -0.182405253 0.07304167 0.8102274
## TRSC-PSCO  0.058685801 -0.071831456 0.18920306 0.7766137
## STOD-STCA  0.037846958 -0.109884306 0.18557822 0.9750269
## TRSC-STCA  0.151214551  0.001061300 0.30136780 0.0473875
## TRSC-STOD  0.113367592  0.007051739 0.21968345 0.0297695
```

```
betadisper_bray_site.anova <- anova(betadisper_bray_site, permutations = 999)
betadisper_bray_site.anova
```

```
## Analysis of Variance Table
##
## Response: Distances
##           Df  Sum Sq  Mean Sq F value  Pr(>F)
## Groups     2 0.14913 0.074565  6.7054 0.00202 **
## Residuals 81 0.90074 0.011120
## ---
## Signif. codes:  0 '***' 0.001 '**' 0.01 '*' 0.05 '.' 0.1 ' ' 1
```

```
# test significance for beta dispersion between each group with p-values adjusted by Tukey HSD
betadisper_bray_site.TukeyHSD <- TukeyHSD(betadisper_bray_site)
betadisper_bray_site.TukeyHSD
```

```
##   Tukey multiple comparisons of means
##     95% family-wise confidence level
##
## Fit: aov(formula = distances ~ group, data = df)
##
## $group
##                diff         lwr          upr     p adj
## S1-BP4 -0.078478947 -0.15220826 -0.004749636 0.0342508
## S4-BP4 -0.087636024 -0.15095861 -0.024313442 0.0040150
## S4-S1  -0.009157077 -0.08976362  0.071449470 0.9602781
```

```
betadisper_jacc_species.anova <- anova(betadisper_jacc_species, permutations = 999)
betadisper_jacc_species.anova
```

```
## Analysis of Variance Table
##
## Response: Distances
##           Df  Sum Sq Mean Sq F value    Pr(>F)
## Groups     5 0.66109 0.13222  14.372 5.786e-10 ***
## Residuals 78 0.71759 0.00920
## ---
## Signif. codes:  0 '***' 0.001 '**' 0.01 '*' 0.05 '.' 0.1 ' ' 1
```

```
# test significance for beta dispersion between each group with p-values adjusted by Tukey HSD
betadisper_jacc_species.TukeyHSD <- TukeyHSD(betadisper_jacc_species)
betadisper_jacc_species.TukeyHSD
```

```
##   Tukey multiple comparisons of means
##     95% family-wise confidence level
##
## Fit: aov(formula = distances ~ group, data = df)
##
## $group
##                   diff          lwr        upr     p adj
## KISU-CHSE  0.410386763  0.232093531 0.58867999 0.0000000
## PSCO-CHSE  0.443003079  0.262106889 0.62389927 0.0000000
## STCA-CHSE  0.388397440  0.198671914 0.57812297 0.0000009
## STOD-CHSE  0.414181456  0.242950048 0.58541286 0.0000000
## TRSC-CHSE  0.492449803  0.319972053 0.66492755 0.0000000
## PSCO-KISU  0.032616317 -0.077630758 0.14286339 0.9537849
## STCA-KISU -0.021989323 -0.146193835 0.10221519 0.9953500
## STOD-KISU  0.003794693 -0.089753052 0.09734244 0.9999966
## TRSC-KISU  0.082063041 -0.013746980 0.17787306 0.1359986
## STCA-PSCO -0.054605640 -0.182518563 0.07330728 0.8120734
## STOD-PSCO -0.028821624 -0.127239792 0.06959654 0.9557127
## TRSC-PSCO  0.049446724 -0.051124223 0.15001767 0.7048152
## STOD-STCA  0.025784016 -0.088051299 0.13961933 0.9855433
## TRSC-STCA  0.104052364 -0.011649229 0.21975396 0.1027776
## TRSC-STOD  0.078268348 -0.003654045 0.16019074 0.0695147
```

```
betadisper_jacc_site.anova <- anova(betadisper_jacc_site, permutations = 999)
betadisper_jacc_site.anova
```

```
## Analysis of Variance Table
##
## Response: Distances
##           Df  Sum Sq  Mean Sq F value   Pr(>F)
## Groups     2 0.06750 0.033750  5.8864 0.004103 **
## Residuals 81 0.46442 0.005734
## ---
## Signif. codes:  0 '***' 0.001 '**' 0.01 '*' 0.05 '.' 0.1 ' ' 1
```

```r
# test significance for beta dispersion between each group with p-values adjusted by Tukey HSD
betadisper_jacc_site.TukeyHSD <- TukeyHSD(betadisper_jacc_site)
betadisper_jacc_site.TukeyHSD
```

```
##   Tukey multiple comparisons of means
##     95% family-wise confidence level
##
## Fit: aov(formula = distances ~ group, data = df)
##
## $group
##               diff         lwr          upr     p adj
## S1-BP4 -0.04736721 -0.10030887  0.005574443 0.0889252
## S4-BP4 -0.06136485 -0.10683391 -0.015895794 0.0051576
## S4-S1  -0.01399764 -0.07187753  0.043882247 0.8325491
```

```r
betadisper_uni_species.anova <- anova(betadisper_uni_species, permutations = 999)
betadisper_uni_species.anova
```

```
## Analysis of Variance Table
##
## Response: Distances
##           Df  Sum Sq  Mean Sq F value    Pr(>F)
## Groups     5 0.43248 0.086495  9.7405 2.948e-07 ***
## Residuals 78 0.69263 0.008880
## ---
## Signif. codes:  0 '***' 0.001 '**' 0.01 '*' 0.05 '.' 0.1 ' ' 1
```

```r
# test significance for beta dispersion between each group with p-values adjusted by Tukey HSD
betadisper_uni_species.TukeyHSD <- TukeyHSD(betadisper_uni_species)
betadisper_uni_species.TukeyHSD
```

```
##   Tukey multiple comparisons of means
##     95% family-wise confidence level
##
## Fit: aov(formula = distances ~ group, data = df)
##
## $group
##                   diff         lwr        upr     p adj
## KISU-CHSE  0.212938529  0.03777286  0.38810420 0.0082713
## PSCO-CHSE  0.164206056 -0.01351691  0.34192902 0.0868451
## STCA-CHSE -0.003214202 -0.18961162  0.18318322 1.0000000
## STOD-CHSE  0.221583241  0.05335552  0.38981096 0.0031979
## TRSC-CHSE  0.197205374  0.02775317  0.36665757 0.0131224
```

```
## PSCO-KISU -0.048732473 -0.15704563  0.05958068 0.7761577
## STCA-KISU -0.216152730 -0.33817849 -0.09412697 0.0000250
## STOD-KISU  0.008644712 -0.08326205  0.10055147 0.9997799
## TRSC-KISU -0.015733154 -0.10986251  0.07839620 0.9964532
## STCA-PSCO -0.167420257 -0.29308937 -0.04175114 0.0027635
## STOD-PSCO  0.057377185 -0.03931456  0.15406893 0.5141052
## TRSC-PSCO  0.032999319 -0.06580744  0.13180608 0.9242662
## STOD-STCA  0.224797442  0.11295899  0.33663589 0.0000015
## TRSC-STCA  0.200419576  0.08674758  0.31409157 0.0000274
## TRSC-STOD -0.024377866 -0.10486320  0.05610747 0.9490411
```

```
betadisper_uni_site.anova <- anova(betadisper_uni_site, permutations = 999)
betadisper_uni_site.anova
```

```
## Analysis of Variance Table
##
## Response: Distances
##           Df  Sum Sq  Mean Sq F value    Pr(>F)
## Groups     2 0.13476 0.067379  9.4038 0.0002126 ***
## Residuals 81 0.58037 0.007165
## ---
## Signif. codes:  0 '***' 0.001 '**' 0.01 '*' 0.05 '.' 0.1 ' ' 1
```

```
# test significance for beta dispersion between each group with p-values adjusted by Tukey HSD
betadisper_uni_site.TukeyHSD <- TukeyHSD(betadisper_uni_site)
betadisper_uni_site.TukeyHSD
```

```
##   Tukey multiple comparisons of means
##     95% family-wise confidence level
##
## Fit: aov(formula = distances ~ group, data = df)
##
## $group
##               diff        lwr          upr     p adj
## S1-BP4 -0.10423747 -0.1634200 -0.045054921 0.0001959
## S4-BP4 -0.04968566 -0.1005147  0.001143402 0.0568000
## S4-S1   0.05455181 -0.0101511  0.119254724 0.1155727
```

```
betadisper_wuni_species.anova <- anova(betadisper_wuni_species, permutations = 999)
betadisper_wuni_species.anova
```

```
## Analysis of Variance Table
##
## Response: Distances
##           Df   Sum Sq   Mean Sq F value   Pr(>F)
## Groups     5 0.024269 0.0048539  3.5699 0.005851 **
## Residuals 78 0.106053 0.0013596
## ---
## Signif. codes:  0 '***' 0.001 '**' 0.01 '*' 0.05 '.' 0.1 ' ' 1
```

```r
# test significance for beta dispersion between each group with p-values adjusted by Tukey HSD
betadisper_wuni_species.TukeyHSD <- TukeyHSD(betadisper_wuni_species)
betadisper_wuni_species.TukeyHSD
```

```
##   Tukey multiple comparisons of means
##     95% family-wise confidence level
##
## Fit: aov(formula = distances ~ group, data = df)
##
## $group
##                     diff          lwr         upr     p adj
## KISU-CHSE  0.0329247053 -0.035617506 0.101466917 0.7248129
## PSCO-CHSE  0.0107835441 -0.058759337 0.080326425 0.9975152
## STCA-CHSE  0.0538807483 -0.019056441 0.126817937 0.2691464
## STOD-CHSE  0.0113877764 -0.054439621 0.077215174 0.9958287
## TRSC-CHSE  0.0442717227 -0.022034813 0.110578258 0.3798713
## PSCO-KISU -0.0221411613 -0.064524028 0.020241706 0.6485329
## STCA-KISU  0.0209560429 -0.026792556 0.068704641 0.7937241
## STOD-KISU -0.0215369290 -0.057499984 0.014426126 0.5038336
## TRSC-KISU  0.0113470173 -0.025485737 0.048179771 0.9453482
## STCA-PSCO  0.0430972042 -0.006077038 0.092271447 0.1196352
## STOD-PSCO  0.0006042323 -0.037231185 0.038439650 1.0000000
## TRSC-PSCO  0.0334881786 -0.005174843 0.072151200 0.1278692
## STOD-STCA -0.0424929719 -0.086255285 0.001269341 0.0620611
## TRSC-STCA -0.0096090256 -0.054088802 0.034870751 0.9883301
## TRSC-STOD  0.0328839463  0.001390087 0.064377806 0.0355391
```

```r
betadisper_wuni_site.anova <- anova(betadisper_wuni_site, permutations = 999)
betadisper_wuni_site.anova
```

```
## Analysis of Variance Table
##
## Response: Distances
##           Df   Sum Sq   Mean Sq F value    Pr(>F)
## Groups     2 0.018644 0.0093219   8.335 0.0005109 ***
## Residuals 81 0.090591 0.0011184
## ---
## Signif. codes:  0 '***' 0.001 '**' 0.01 '*' 0.05 '.' 0.1 ' ' 1
```

```r
# test significance for beta dispersion between each group with p-values adjusted by Tukey HSD
betadisper_wuni_site.TukeyHSD <- TukeyHSD(betadisper_wuni_site)
betadisper_wuni_site.TukeyHSD
```

```
##   Tukey multiple comparisons of means
##     95% family-wise confidence level
##
## Fit: aov(formula = distances ~ group, data = df)
##
## $group
##                diff          lwr         upr     p adj
## S1-BP4   0.02921779  0.005835706  0.05259988 0.0104134
## S4-BP4  -0.01432137 -0.034403130  0.00576038 0.2104093
## S4-S1   -0.04353917 -0.069102257 -0.01797607 0.0003212
```

Testing for differences in centroid location

In this section, PERMANOVA (through Adonis2) is used to test for significant differences in centroid locations for each beta diversity metric, for both species and sites. If Adonis2 PERMANOVA result is significant, we can reject the null hypothesis that the species have the same centroid. Pairwise comparisons with Benjamini-Hochberg-corrected p-values are also presented.

```
adonis2(carapace_bray ~ Species+Site+Sex+log10(Read_depth)+Year, data = sampledf, by="margin", permutati
```

```
## Permutation test for adonis under reduced model
## Marginal effects of terms
## Permutation: free
## Number of permutations: 999
##
## adonis2(formula = carapace_bray ~ Species + Site + Sex + log10(Read_depth) + Year, data = sampledf, p
##                   Df SumOfSqs      R2      F Pr(>F)
## Species            5    6.927 0.21091 5.1365  0.001 ***
## Site               2    1.867 0.05685 3.4615  0.001 ***
## Sex                2    0.514 0.01565 0.9531  0.502
## log10(Read_depth)  1    0.475 0.01447 1.7615  0.061 .
## Year               1    1.012 0.03082 3.7525  0.001 ***
## Residual          72   19.420 0.59128
## Total             83   32.843 1.00000
## ---
## Signif. codes:  0 '***' 0.001 '**' 0.01 '*' 0.05 '.' 0.1 ' ' 1
```

```
adonis2(carapace_jacc ~ Species+Site+Sex+log10(Read_depth)+Year, data = sampledf, by="margin", permutati
```

```
## Permutation test for adonis under reduced model
## Marginal effects of terms
## Permutation: free
## Number of permutations: 999
##
## adonis2(formula = carapace_jacc ~ Species + Site + Sex + log10(Read_depth) + Year, data = sampledf, p
##                   Df SumOfSqs      R2      F Pr(>F)
## Species            5    5.910 0.16614 3.5293  0.001 ***
## Site               2    1.653 0.04647 2.4680  0.001 ***
## Sex                2    0.599 0.01683 0.8940  0.628
## log10(Read_depth)  1    0.496 0.01395 1.4818  0.077 .
## Year               1    0.922 0.02592 2.7532  0.001 ***
## Residual          72   24.115 0.67789
## Total             83   35.574 1.00000
## ---
## Signif. codes:  0 '***' 0.001 '**' 0.01 '*' 0.05 '.' 0.1 ' ' 1
```

```
adonis2(carapace_uni ~ Species+Site+Sex+log10(Read_depth)+Year, data = sampledf, by="margin", permutatio
```

```
## Permutation test for adonis under reduced model
## Marginal effects of terms
## Permutation: free
## Number of permutations: 999
##
```

```
## adonis2(formula = carapace_uni ~ Species + Site + Sex + log10(Read_depth) + Year, data = sampledf, pe
##                   Df SumOfSqs      R2      F Pr(>F)
## Species            5   4.4729 0.15710 3.3659  0.001 ***
## Site               2   2.5087 0.08811 4.7196  0.001 ***
## Sex                2   0.6459 0.02269 1.2152  0.137
## log10(Read_depth)  1   0.3769 0.01324 1.4182  0.075 .
## Year               1   0.5432 0.01908 2.0437  0.005 **
## Residual          72  19.1362 0.67213
## Total             83  28.4711 1.00000
## ---
## Signif. codes:  0 '***' 0.001 '**' 0.01 '*' 0.05 '.' 0.1 ' ' 1
```

```
adonis2(carapace_wuni ~ Species+Site+Sex+log10(Read_depth)+Year, data = sampledf, by="margin", permutati
```

```
## Permutation test for adonis under reduced model
## Marginal effects of terms
## Permutation: free
## Number of permutations: 999
##
## adonis2(formula = carapace_wuni ~ Species + Site + Sex + log10(Read_depth) + Year, data = sampledf, p
##                   Df SumOfSqs      R2      F Pr(>F)
## Species            5 0.013296 0.05880 1.4885  0.170
## Site               2 0.029856 0.13202 8.3558  0.001 ***
## Sex                2 0.002456 0.01086 0.6874  0.623
## log10(Read_depth)  1 0.011628 0.05142 6.5086  0.004 **
## Year               1 0.005367 0.02373 3.0040  0.045 *
## Residual          72 0.128628 0.56881
## Total             83 0.226136 1.00000
## ---
## Signif. codes:  0 '***' 0.001 '**' 0.01 '*' 0.05 '.' 0.1 ' ' 1
```

```
#Pairwise comparisons for beta diversity for species and site
pairwise.adonis2(carapace_bray ~ Species, data = sampledf, p.adjust.m="BH")
```

```
## $parent_call
## [1] "carapace_bray ~ Species , strata = Null , permutations 999"
##
## $KISU_vs_STOD
##          Df SumOfSqs      R2      F Pr(>F)
## Species   1   1.4662 0.13111 5.5829  0.001 ***
## Residual 37   9.7168 0.86889
## Total    38  11.1829 1.00000
## ---
## Signif. codes:  0 '***' 0.001 '**' 0.01 '*' 0.05 '.' 0.1 ' ' 1
##
## $KISU_vs_TRSC
##          Df SumOfSqs      R2      F Pr(>F)
## Species   1   2.8191 0.19561 8.2681  0.001 ***
## Residual 34  11.5927 0.80439
## Total    35  14.4119 1.00000
## ---
## Signif. codes:  0 '***' 0.001 '**' 0.01 '*' 0.05 '.' 0.1 ' ' 1
##
```

```
## $KISU_vs_PSCO
##          Df SumOfSqs      R2      F Pr(>F)
## Species   1   2.1826 0.22956 7.1511  0.001 ***
## Residual 24   7.3250 0.77044
## Total    25   9.5076 1.00000
## ---
## Signif. codes:  0 '***' 0.001 '**' 0.01 '*' 0.05 '.' 0.1 ' ' 1
##
## $KISU_vs_STCA
##          Df SumOfSqs      R2      F Pr(>F)
## Species   1   2.5454 0.32532 9.6436  0.001 ***
## Residual 20   5.2790 0.67468
## Total    21   7.8244 1.00000
## ---
## Signif. codes:  0 '***' 0.001 '**' 0.01 '*' 0.05 '.' 0.1 ' ' 1
##
## $KISU_vs_CHSE
##          Df SumOfSqs      R2      F Pr(>F)
## Species   1   0.4304 0.10695 1.7963   0.14
## Residual 15   3.5937 0.89305
## Total    16   4.0240 1.00000
##
## $STOD_vs_TRSC
##          Df SumOfSqs      R2      F Pr(>F)
## Species   1   3.4769 0.19665 11.016  0.001 ***
## Residual 45  14.2037 0.80335
## Total    46  17.6807 1.00000
## ---
## Signif. codes:  0 '***' 0.001 '**' 0.01 '*' 0.05 '.' 0.1 ' ' 1
##
## $STOD_vs_PSCO
##          Df SumOfSqs      R2      F Pr(>F)
## Species   1    2.493 0.20058 8.7817  0.001 ***
## Residual 35    9.936 0.79942
## Total    36   12.429 1.00000
## ---
## Signif. codes:  0 '***' 0.001 '**' 0.01 '*' 0.05 '.' 0.1 ' ' 1
##
## $STOD_vs_STCA
##          Df SumOfSqs      R2      F Pr(>F)
## Species   1   2.0067 0.20277 7.8845  0.001 ***
## Residual 31   7.8900 0.79723
## Total    32   9.8967 1.00000
## ---
## Signif. codes:  0 '***' 0.001 '**' 0.01 '*' 0.05 '.' 0.1 ' ' 1
##
## $STOD_vs_CHSE
##          Df SumOfSqs      R2      F Pr(>F)
## Species   1   0.7563 0.10865 3.1693  0.012 *
## Residual 26   6.2047 0.89135
## Total    27   6.9610 1.00000
## ---
## Signif. codes:  0 '***' 0.001 '**' 0.01 '*' 0.05 '.' 0.1 ' ' 1
##
```

```
## $TRSC_vs_PSCO
##          Df SumOfSqs      R2      F Pr(>F)
## Species   1   0.6667 0.05342 1.8061   0.05 *
## Residual 32  11.8120 0.94658
## Total    33  12.4787 1.00000
## ---
## Signif. codes:  0 '***' 0.001 '**' 0.01 '*' 0.05 '.' 0.1 ' ' 1
##
## $TRSC_vs_STCA
##          Df SumOfSqs      R2      F Pr(>F)
## Species   1   1.3613 0.12234 3.9031  0.001 ***
## Residual 28   9.7659 0.87766
## Total    29  11.1273 1.00000
## ---
## Signif. codes:  0 '***' 0.001 '**' 0.01 '*' 0.05 '.' 0.1 ' ' 1
##
## $TRSC_vs_CHSE
##          Df SumOfSqs      R2      F Pr(>F)
## Species   1   1.5812 0.16366 4.5007  0.002 **
## Residual 23   8.0807 0.83634
## Total    24   9.6619 1.00000
## ---
## Signif. codes:  0 '***' 0.001 '**' 0.01 '*' 0.05 '.' 0.1 ' ' 1
##
## $PSCO_vs_STCA
##          Df SumOfSqs      R2      F Pr(>F)
## Species   1   1.9397 0.26079 6.3502  0.001 ***
## Residual 18   5.4982 0.73921
## Total    19   7.4380 1.00000
## ---
## Signif. codes:  0 '***' 0.001 '**' 0.01 '*' 0.05 '.' 0.1 ' ' 1
##
## $PSCO_vs_CHSE
##          Df SumOfSqs      R2      F Pr(>F)
## Species   1   1.3321 0.25891 4.5416  0.004 **
## Residual 13   3.8130 0.74109
## Total    14   5.1451 1.00000
## ---
## Signif. codes:  0 '***' 0.001 '**' 0.01 '*' 0.05 '.' 0.1 ' ' 1
##
## $STCA_vs_CHSE
##          Df SumOfSqs      R2      F Pr(>F)
## Species   1   1.6738 0.48647 8.5256  0.012 *
## Residual  9   1.7669 0.51353
## Total    10   3.4406 1.00000
## ---
## Signif. codes:  0 '***' 0.001 '**' 0.01 '*' 0.05 '.' 0.1 ' ' 1
##
## attr(,"class")
## [1] "pwadstrata" "list"
```

```
pairwise.adonis2(carapace_bray ~ Site, data = sampledf, p.adjust.m="BH")
```

```
## $parent_call
```

```
## [1] "carapace_bray ~ Site , strata = Null , permutations 999"
##
## $BP4_vs_S4
##          Df SumOfSqs      R2      F Pr(>F)
## Site      1   0.8835 0.03499 2.3927  0.021 *
## Residual 66  24.3706 0.96501
## Total    67  25.2541 1.00000
## ---
## Signif. codes:  0 '***' 0.001 '**' 0.01 '*' 0.05 '.' 0.1 ' ' 1
##
## $BP4_vs_S1
##          Df SumOfSqs      R2      F Pr(>F)
## Site      1   2.3625 0.09834 6.2165  0.001 ***
## Residual 57  21.6615 0.90166
## Total    58  24.0240 1.00000
## ---
## Signif. codes:  0 '***' 0.001 '**' 0.01 '*' 0.05 '.' 0.1 ' ' 1
##
## $S4_vs_S1
##          Df SumOfSqs      R2     F Pr(>F)
## Site      1   2.5411 0.16979 7.976  0.001 ***
## Residual 39  12.4250 0.83021
## Total    40  14.9661 1.00000
## ---
## Signif. codes:  0 '***' 0.001 '**' 0.01 '*' 0.05 '.' 0.1 ' ' 1
##
## attr(,"class")
## [1] "pwadstrata" "list"
```

```
pairwise.adonis2(carapace_jacc ~ Species, data = sampledf, p.adjust.m="BH")
```

```
## $parent_call
## [1] "carapace_jacc ~ Species , strata = Null , permutations 999"
##
## $KISU_vs_STOD
##          Df SumOfSqs      R2      F Pr(>F)
## Species   1   1.2318 0.09149 3.7259  0.001 ***
## Residual 37  12.2319 0.90851
## Total    38  13.4636 1.00000
## ---
## Signif. codes:  0 '***' 0.001 '**' 0.01 '*' 0.05 '.' 0.1 ' ' 1
##
## $KISU_vs_TRSC
##          Df SumOfSqs      R2      F Pr(>F)
## Species   1   2.1734 0.14058 5.5617  0.001 ***
## Residual 34  13.2865 0.85942
## Total    35  15.4599 1.00000
## ---
## Signif. codes:  0 '***' 0.001 '**' 0.01 '*' 0.05 '.' 0.1 ' ' 1
##
## $KISU_vs_PSCO
##          Df SumOfSqs      R2      F Pr(>F)
## Species   1   1.7381 0.16794 4.8442  0.001 ***
## Residual 24   8.6113 0.83206
```

```
## Total    25  10.3494 1.00000
## ---
## Signif. codes:  0 '***' 0.001 '**' 0.01 '*' 0.05 '.' 0.1 ' ' 1
##
## $KISU_vs_STCA
##          Df SumOfSqs      R2     F Pr(>F)
## Species   1   1.9584 0.22702 5.874  0.001 ***
## Residual 20   6.6679 0.77298
## Total    21   8.6263 1.00000
## ---
## Signif. codes:  0 '***' 0.001 '**' 0.01 '*' 0.05 '.' 0.1 ' ' 1
##
## $KISU_vs_CHSE
##          Df SumOfSqs      R2      F Pr(>F)
## Species   1   0.4964 0.09971 1.6612  0.145
## Residual 15   4.4823 0.90029
## Total    16   4.9787 1.00000
##
## $STOD_vs_TRSC
##          Df SumOfSqs      R2      F Pr(>F)
## Species   1   2.6661 0.13721 7.1566  0.001 ***
## Residual 45  16.7639 0.86279
## Total    46  19.4300 1.00000
## ---
## Signif. codes:  0 '***' 0.001 '**' 0.01 '*' 0.05 '.' 0.1 ' ' 1
##
## $STOD_vs_PSCO
##          Df SumOfSqs      R2      F Pr(>F)
## Species   1   2.0057 0.14231 5.8072  0.001 ***
## Residual 35  12.0887 0.85769
## Total    36  14.0945 1.00000
## ---
## Signif. codes:  0 '***' 0.001 '**' 0.01 '*' 0.05 '.' 0.1 ' ' 1
##
## $STOD_vs_STCA
##          Df SumOfSqs      R2     F Pr(>F)
## Species   1   1.6151 0.13733 4.935  0.001 ***
## Residual 31  10.1454 0.86267
## Total    32  11.7604 1.00000
## ---
## Signif. codes:  0 '***' 0.001 '**' 0.01 '*' 0.05 '.' 0.1 ' ' 1
##
## $STOD_vs_CHSE
##          Df SumOfSqs      R2      F Pr(>F)
## Species   1   0.7924 0.09054 2.5884   0.01 **
## Residual 26   7.9597 0.90946
## Total    27   8.7522 1.00000
## ---
## Signif. codes:  0 '***' 0.001 '**' 0.01 '*' 0.05 '.' 0.1 ' ' 1
##
## $TRSC_vs_PSCO
##          Df SumOfSqs      R2      F Pr(>F)
## Species   1   0.6191 0.04499 1.5074  0.083 .
## Residual 32  13.1433 0.95501
```

```
## Total    33  13.7625 1.00000
## ---
## Signif. codes:  0 '***' 0.001 '**' 0.01 '*' 0.05 '.' 0.1 ' ' 1
##
## $TRSC_vs_STCA
##          Df SumOfSqs      R2      F Pr(>F)
## Species   1   1.1447 0.09273 2.8619  0.001 ***
## Residual 28  11.2000 0.90727
## Total    29  12.3447 1.00000
## ---
## Signif. codes:  0 '***' 0.001 '**' 0.01 '*' 0.05 '.' 0.1 ' ' 1
##
## $TRSC_vs_CHSE
##          Df SumOfSqs      R2      F Pr(>F)
## Species   1   1.4469 0.13831 3.6917  0.003 **
## Residual 23   9.0143 0.86169
## Total    24  10.4612 1.00000
## ---
## Signif. codes:  0 '***' 0.001 '**' 0.01 '*' 0.05 '.' 0.1 ' ' 1
##
## $PSCO_vs_STCA
##          Df SumOfSqs      R2      F Pr(>F)
## Species   1   1.5571 0.19266 4.2955  0.001 ***
## Residual 18   6.5248 0.80734
## Total    19   8.0818 1.00000
## ---
## Signif. codes:  0 '***' 0.001 '**' 0.01 '*' 0.05 '.' 0.1 ' ' 1
##
## $PSCO_vs_CHSE
##          Df SumOfSqs      R2      F Pr(>F)
## Species   1   1.2350 0.22155 3.6999  0.003 **
## Residual 13   4.3391 0.77845
## Total    14   5.5741 1.00000
## ---
## Signif. codes:  0 '***' 0.001 '**' 0.01 '*' 0.05 '.' 0.1 ' ' 1
##
## $STCA_vs_CHSE
##          Df SumOfSqs      R2      F Pr(>F)
## Species   1   1.4768 0.38136 5.5479  0.007 **
## Residual  9   2.3958 0.61864
## Total    10   3.8726 1.00000
## ---
## Signif. codes:  0 '***' 0.001 '**' 0.01 '*' 0.05 '.' 0.1 ' ' 1
##
## attr(,"class")
## [1] "pwadstrata" "list"
```

```
pairwise.adonis2(carapace_jacc ~ Site, data = sampledf, p.adjust.m="BH")
```

```
## $parent_call
## [1] "carapace_jacc ~ Site , strata = Null , permutations 999"
##
## $BP4_vs_S4
##          Df SumOfSqs      R2      F Pr(>F)
```

```
## Site      1   0.7825 0.02828 1.9209  0.024 *
## Residual 66  26.8874 0.97172
## Total    67  27.6699 1.00000
## ---
## Signif. codes:  0 '***' 0.001 '**' 0.01 '*' 0.05 '.' 0.1 ' ' 1
##
## $BP4_vs_S1
##          Df SumOfSqs     R2      F Pr(>F)
## Site      1   1.8125 0.0705 4.3231  0.001 ***
## Residual 57  23.8978 0.9295
## Total    58  25.7103 1.0000
## ---
## Signif. codes:  0 '***' 0.001 '**' 0.01 '*' 0.05 '.' 0.1 ' ' 1
##
## $S4_vs_S1
##          Df SumOfSqs      R2      F Pr(>F)
## Site      1   1.9933 0.12009 5.3229  0.001 ***
## Residual 39  14.6044 0.87991
## Total    40  16.5976 1.00000
## ---
## Signif. codes:  0 '***' 0.001 '**' 0.01 '*' 0.05 '.' 0.1 ' ' 1
##
## attr(,"class")
## [1] "pwadstrata" "list"
```

```
pairwise.adonis2(carapace_uni ~ Species, data = sampledf, p.adjust.m="BH")
```

```
## $parent_call
## [1] "carapace_uni ~ Species , strata = Null , permutations 999"
##
## $KISU_vs_STOD
##          Df SumOfSqs      R2      F Pr(>F)
## Species   1   0.7776 0.05879 2.3112  0.003 **
## Residual 37  12.4478 0.94121
## Total    38  13.2254 1.00000
## ---
## Signif. codes:  0 '***' 0.001 '**' 0.01 '*' 0.05 '.' 0.1 ' ' 1
##
## $KISU_vs_TRSC
##          Df SumOfSqs      R2      F Pr(>F)
## Species   1   0.9205 0.07785 2.8704  0.001 ***
## Residual 34  10.9035 0.92215
## Total    35  11.8241 1.00000
## ---
## Signif. codes:  0 '***' 0.001 '**' 0.01 '*' 0.05 '.' 0.1 ' ' 1
##
## $KISU_vs_PSCO
##          Df SumOfSqs      R2      F Pr(>F)
## Species   1   0.6913 0.08441 2.2127  0.006 **
## Residual 24   7.4985 0.91559
## Total    25   8.1899 1.00000
## ---
## Signif. codes:  0 '***' 0.001 '**' 0.01 '*' 0.05 '.' 0.1 ' ' 1
##
```

```
## $KISU_vs_STCA
##          Df SumOfSqs      R2      F Pr(>F)
## Species   1   1.8563 0.25883 6.9844  0.001 ***
## Residual 20   5.3156 0.74117
## Total    21   7.1719 1.00000
## ---
## Signif. codes:  0 '***' 0.001 '**' 0.01 '*' 0.05 '.' 0.1 ' ' 1
##
## $KISU_vs_CHSE
##          Df SumOfSqs     R2      F Pr(>F)
## Species   1   0.7027 0.1292 2.2256  0.024 *
## Residual 15   4.7357 0.8708
## Total    16   5.4384 1.0000
## ---
## Signif. codes:  0 '***' 0.001 '**' 0.01 '*' 0.05 '.' 0.1 ' ' 1
##
## $STOD_vs_TRSC
##          Df SumOfSqs      R2      F Pr(>F)
## Species   1   1.2583 0.07941 3.8817  0.001 ***
## Residual 45  14.5878 0.92059
## Total    46  15.8462 1.00000
## ---
## Signif. codes:  0 '***' 0.001 '**' 0.01 '*' 0.05 '.' 0.1 ' ' 1
##
## $STOD_vs_PSCO
##          Df SumOfSqs    R2      F Pr(>F)
## Species   1   0.8677 0.072 2.7157  0.003 **
## Residual 35  11.1828 0.928
## Total    36  12.0505 1.000
## ---
## Signif. codes:  0 '***' 0.001 '**' 0.01 '*' 0.05 '.' 0.1 ' ' 1
##
## $STOD_vs_STCA
##          Df SumOfSqs      R2      F Pr(>F)
## Species   1   1.4230 0.13652 4.9014  0.001 ***
## Residual 31   8.9999 0.86348
## Total    32  10.4228 1.00000
## ---
## Signif. codes:  0 '***' 0.001 '**' 0.01 '*' 0.05 '.' 0.1 ' ' 1
##
## $STOD_vs_CHSE
##          Df SumOfSqs     R2      F Pr(>F)
## Species   1   0.6572 0.0724 2.0292   0.01 **
## Residual 26   8.4200 0.9276
## Total    27   9.0772 1.0000
## ---
## Signif. codes:  0 '***' 0.001 '**' 0.01 '*' 0.05 '.' 0.1 ' ' 1
##
## $TRSC_vs_PSCO
##          Df SumOfSqs     R2      F Pr(>F)
## Species   1   0.6295 0.0613 2.0898  0.004 **
## Residual 32   9.6385 0.9387
## Total    33  10.2680 1.0000
## ---
```

```
## Signif. codes:  0 '***' 0.001 '**' 0.01 '*' 0.05 '.' 0.1 ' ' 1
##
## $TRSC_vs_STCA
##          Df SumOfSqs      R2      F Pr(>F)
## Species   1   1.8894 0.20218 7.0957  0.001 ***
## Residual 28   7.4556 0.79782
## Total    29   9.3449 1.00000
## ---
## Signif. codes:  0 '***' 0.001 '**' 0.01 '*' 0.05 '.' 0.1 ' ' 1
##
## $TRSC_vs_CHSE
##          Df SumOfSqs      R2      F Pr(>F)
## Species   1   0.7889 0.10293 2.6389  0.002 **
## Residual 23   6.8757 0.89707
## Total    24   7.6646 1.00000
## ---
## Signif. codes:  0 '***' 0.001 '**' 0.01 '*' 0.05 '.' 0.1 ' ' 1
##
## $PSCO_vs_STCA
##          Df SumOfSqs      R2     F Pr(>F)
## Species   1   1.2811 0.24028 5.693  0.001 ***
## Residual 18   4.0506 0.75972
## Total    19   5.3317 1.00000
## ---
## Signif. codes:  0 '***' 0.001 '**' 0.01 '*' 0.05 '.' 0.1 ' ' 1
##
## $PSCO_vs_CHSE
##          Df SumOfSqs      R2      F Pr(>F)
## Species   1   0.4582 0.11661 1.7161  0.032 *
## Residual 13   3.4707 0.88339
## Total    14   3.9289 1.00000
## ---
## Signif. codes:  0 '***' 0.001 '**' 0.01 '*' 0.05 '.' 0.1 ' ' 1
##
## $STCA_vs_CHSE
##          Df SumOfSqs      R2      F Pr(>F)
## Species   1  0.63831 0.33141 4.4611  0.009 **
## Residual  9  1.28776 0.66859
## Total    10  1.92608 1.00000
## ---
## Signif. codes:  0 '***' 0.001 '**' 0.01 '*' 0.05 '.' 0.1 ' ' 1
##
## attr(,"class")
## [1] "pwadstrata" "list"
```

```
pairwise.adonis2(carapace_uni ~ Site, data = sampledf, p.adjust.m="BH")
```

```
## $parent_call
## [1] "carapace_uni ~ Site , strata = Null , permutations 999"
##
## $BP4_vs_S4
##          Df SumOfSqs      R2      F Pr(>F)
## Site      1   0.7683 0.03363 2.2966  0.002 **
## Residual 66  22.0798 0.96637
```

```
## Total    67  22.8482 1.00000
## ---
## Signif. codes:  0 '***' 0.001 '**' 0.01 '*' 0.05 '.' 0.1 ' ' 1
##
## $BP4_vs_S1
##          Df SumOfSqs      R2      F Pr(>F)
## Site      1   2.0326 0.09927 6.2821  0.001 ***
## Residual 57  18.4427 0.90073
## Total    58  20.4753 1.00000
## ---
## Signif. codes:  0 '***' 0.001 '**' 0.01 '*' 0.05 '.' 0.1 ' ' 1
##
## $S4_vs_S1
##          Df SumOfSqs      R2      F Pr(>F)
## Site      1   1.4122 0.11431 5.0336  0.001 ***
## Residual 39  10.9418 0.88569
## Total    40  12.3540 1.00000
## ---
## Signif. codes:  0 '***' 0.001 '**' 0.01 '*' 0.05 '.' 0.1 ' ' 1
##
## attr(,"class")
## [1] "pwadstrata" "list"
```

```
pairwise.adonis2(carapace_wuni ~ Species, data = sampledf, p.adjust.m="BH")
```

```
## $parent_call
## [1] "carapace_wuni ~ Species , strata = Null , permutations 999"
##
## $KISU_vs_STOD
##          Df  SumOfSqs       R2       F Pr(>F)
## Species   1 -0.001933 -0.03703 -1.3213   0.98
## Residual 37  0.054116  1.03703
## Total    38  0.052183  1.00000
##
## $KISU_vs_TRSC
##          Df SumOfSqs     R2      F Pr(>F)
## Species   1 0.014784 0.1034 3.9211  0.005 **
## Residual 34 0.128195 0.8966
## Total    35 0.142979 1.0000
## ---
## Signif. codes:  0 '***' 0.001 '**' 0.01 '*' 0.05 '.' 0.1 ' ' 1
##
## $KISU_vs_PSCO
##          Df SumOfSqs      R2      F Pr(>F)
## Species   1 0.000605 0.01621 0.3954  0.803
## Residual 24 0.036707 0.98379
## Total    25 0.037312 1.00000
##
## $KISU_vs_STCA
##          Df SumOfSqs      R2      F Pr(>F)
## Species   1 0.042536 0.39066 12.822  0.001 ***
## Residual 20 0.066347 0.60934
## Total    21 0.108883 1.00000
## ---
```

```
## Signif. codes:  0 '***' 0.001 '**' 0.01 '*' 0.05 '.' 0.1 ' ' 1
##
## $KISU_vs_CHSE
##          Df SumOfSqs      R2      F Pr(>F)
## Species   1 0.000708 0.01995 0.3053   0.69
## Residual 15 0.034798 0.98005
## Total    16 0.035506 1.00000
##
## $STOD_vs_TRSC
##          Df  SumOfSqs     R2       F Pr(>F)
## Species   1 -0.004543 -0.042 -1.8137  0.994
## Residual 45  0.112724  1.042
## Total    46  0.108181  1.000
##
## $STOD_vs_PSCO
##          Df  SumOfSqs      R2      F Pr(>F)
## Species   1 0.0017327 0.07544 2.8557  0.104
## Residual 35 0.0212360 0.92456
## Total    36 0.0229687 1.00000
##
## $STOD_vs_STCA
##          Df SumOfSqs      R2      F Pr(>F)
## Species   1 0.023046 0.31176 14.043  0.001 ***
## Residual 31 0.050876 0.68824
## Total    32 0.073922 1.00000
## ---
## Signif. codes:  0 '***' 0.001 '**' 0.01 '*' 0.05 '.' 0.1 ' ' 1
##
## $STOD_vs_CHSE
##          Df  SumOfSqs      R2      F Pr(>F)
## Species   1 0.0009285 0.04584 1.2491  0.113
## Residual 26 0.0193268 0.95416
## Total    27 0.0202554 1.00000
##
## $TRSC_vs_PSCO
##          Df SumOfSqs      R2      F Pr(>F)
## Species   1 0.003216 0.03264 1.0797  0.446
## Residual 32 0.095315 0.96736
## Total    33 0.098531 1.00000
##
## $TRSC_vs_STCA
##          Df SumOfSqs      R2     F Pr(>F)
## Species   1 0.027419 0.17994 6.144  0.003 **
## Residual 28 0.124955 0.82006
## Total    29 0.152374 1.00000
## ---
## Signif. codes:  0 '***' 0.001 '**' 0.01 '*' 0.05 '.' 0.1 ' ' 1
##
## $TRSC_vs_CHSE
##          Df SumOfSqs      R2      F Pr(>F)
## Species   1 0.003215 0.03328 0.7917  0.368
## Residual 23 0.093406 0.96672
## Total    24 0.096621 1.00000
##
```

```
## $PSCO_vs_STCA
##          Df SumOfSqs      R2      F Pr(>F)
## Species   1 0.026967 0.44623 14.504  0.001 ***
## Residual 18 0.033467 0.55377
## Total    19 0.060434 1.00000
## ---
## Signif. codes:  0 '***' 0.001 '**' 0.01 '*' 0.05 '.' 0.1 ' ' 1
##
## $PSCO_vs_CHSE
##          Df  SumOfSqs      R2      F Pr(>F)
## Species   1 0.0001992 0.09409 1.3503  0.222
## Residual 13 0.0019178 0.90591
## Total    14 0.0021170 1.00000
##
## $STCA_vs_CHSE
##          Df SumOfSqs      R2      F Pr(>F)
## Species   1 0.016386 0.34178 4.6732   0.04 *
## Residual  9 0.031558 0.65822
## Total    10 0.047944 1.00000
## ---
## Signif. codes:  0 '***' 0.001 '**' 0.01 '*' 0.05 '.' 0.1 ' ' 1
##
## attr(,"class")
## [1] "pwadstrata" "list"
```

```
pairwise.adonis2(carapace_wuni ~ Site, data = sampledf, p.adjust.m="BH")
```

```
## $parent_call
## [1] "carapace_wuni ~ Site , strata = Null , permutations 999"
##
## $BP4_vs_S4
##          Df  SumOfSqs       R2       F Pr(>F)
## Site      1 -0.001791 -0.01895 -1.2273  0.909
## Residual 66  0.096311  1.01895
## Total    67  0.094520  1.00000
##
## $BP4_vs_S1
##          Df SumOfSqs     R2      F Pr(>F)
## Site      1  0.06593 0.3016 24.615  0.001 ***
## Residual 57  0.15267 0.6984
## Total    58  0.21860 1.0000
## ---
## Signif. codes:  0 '***' 0.001 '**' 0.01 '*' 0.05 '.' 0.1 ' ' 1
##
## $S4_vs_S1
##          Df SumOfSqs      R2      F Pr(>F)
## Site      1 0.048088 0.41917 28.145  0.001 ***
## Residual 39 0.066634 0.58083
## Total    40 0.114722 1.00000
## ---
## Signif. codes:  0 '***' 0.001 '**' 0.01 '*' 0.05 '.' 0.1 ' ' 1
##
## attr(,"class")
## [1] "pwadstrata" "list"
```

ANOSIM: analysis of similarity testing

For anosim tests, values range from -1 to +1. Values closer to +1 indicate stronger similarities within groups compared to between groups. Values close to zero indicates no difference between groups. Anosim tests are done for each beta diversity metric, first for turtle species and then by collection site.

```r
anosim(carapace_bray, sampledf$Species, permutations = 999)
```

```
##
## Call:
## anosim(x = carapace_bray, grouping = sampledf$Species, permutations = 999)
## Dissimilarity: bray
##
## ANOSIM statistic R: 0.528
##       Significance: 0.001
##
## Permutation: free
## Number of permutations: 999
```

```r
anosim(carapace_jacc, sampledf$Species, permutations = 999)
```

```
##
## Call:
## anosim(x = carapace_jacc, grouping = sampledf$Species, permutations = 999)
## Dissimilarity: jaccard
##
## ANOSIM statistic R: 0.528
##       Significance: 0.001
##
## Permutation: free
## Number of permutations: 999
```

```r
anosim(carapace_uni, sampledf$Species, permutations = 999)
```

```
##
## Call:
## anosim(x = carapace_uni, grouping = sampledf$Species, permutations = 999)
## Dissimilarity:
##
## ANOSIM statistic R: 0.1883
##       Significance: 0.001
##
## Permutation: free
## Number of permutations: 999
```

```r
anosim(carapace_wuni, sampledf$Species, permutations = 999)
```

```
##
## Call:
## anosim(x = carapace_wuni, grouping = sampledf$Species, permutations = 999)
## Dissimilarity:
```

```
##
## ANOSIM statistic R: 0.1611
##       Significance: 0.002
##
## Permutation: free
## Number of permutations: 999
```

```r
anosim(carapace_bray, sampledf$Site, permutations = 999)
```

```
##
## Call:
## anosim(x = carapace_bray, grouping = sampledf$Site, permutations = 999)
## Dissimilarity: bray
##
## ANOSIM statistic R: 0.1341
##       Significance: 0.001
##
## Permutation: free
## Number of permutations: 999
```

```r
anosim(carapace_jacc, sampledf$Site, permutations = 999)
```

```
##
## Call:
## anosim(x = carapace_jacc, grouping = sampledf$Site, permutations = 999)
## Dissimilarity: jaccard
##
## ANOSIM statistic R: 0.1341
##       Significance: 0.001
##
## Permutation: free
## Number of permutations: 999
```

```r
anosim(carapace_uni, sampledf$Site, permutations = 999)
```

```
##
## Call:
## anosim(x = carapace_uni, grouping = sampledf$Site, permutations = 999)
## Dissimilarity:
##
## ANOSIM statistic R: 0.09335
##       Significance: 0.02
##
## Permutation: free
## Number of permutations: 999
```

```r
anosim(carapace_wuni, sampledf$Site, permutations = 999)
```

```
##
## Call:
## anosim(x = carapace_wuni, grouping = sampledf$Site, permutations = 999)
```

```
## Dissimilarity:
##
## ANOSIM statistic R: 0.1988
##       Significance: 0.001
##
## Permutation: free
## Number of permutations: 999
```

generate UpSetR plots

UpSetR plots are generated in place of Venn diagrams to enable effective comparison between all six turtle species. UpSetR plots for collection sites are also generated.

```r
library(UpSetR)
library(gridExtra)
library(methods)
library(grDevices)
library(mltools)
library(data.table)

setwd("/Users/mparks10/Desktop/atoka.2021_2022.combined/phyloseq")
data = read.csv('18S.OTU_table.0_1.txt', na.strings="Not Applicable")
newdata <- one_hot(as.data.table(data))#create the visualization
#with numbers over bars
upset(newdata, nsets=6, order.by="freq", mainbar.y.label = "# ASV in Intersection", sets.x.label = "# A
```

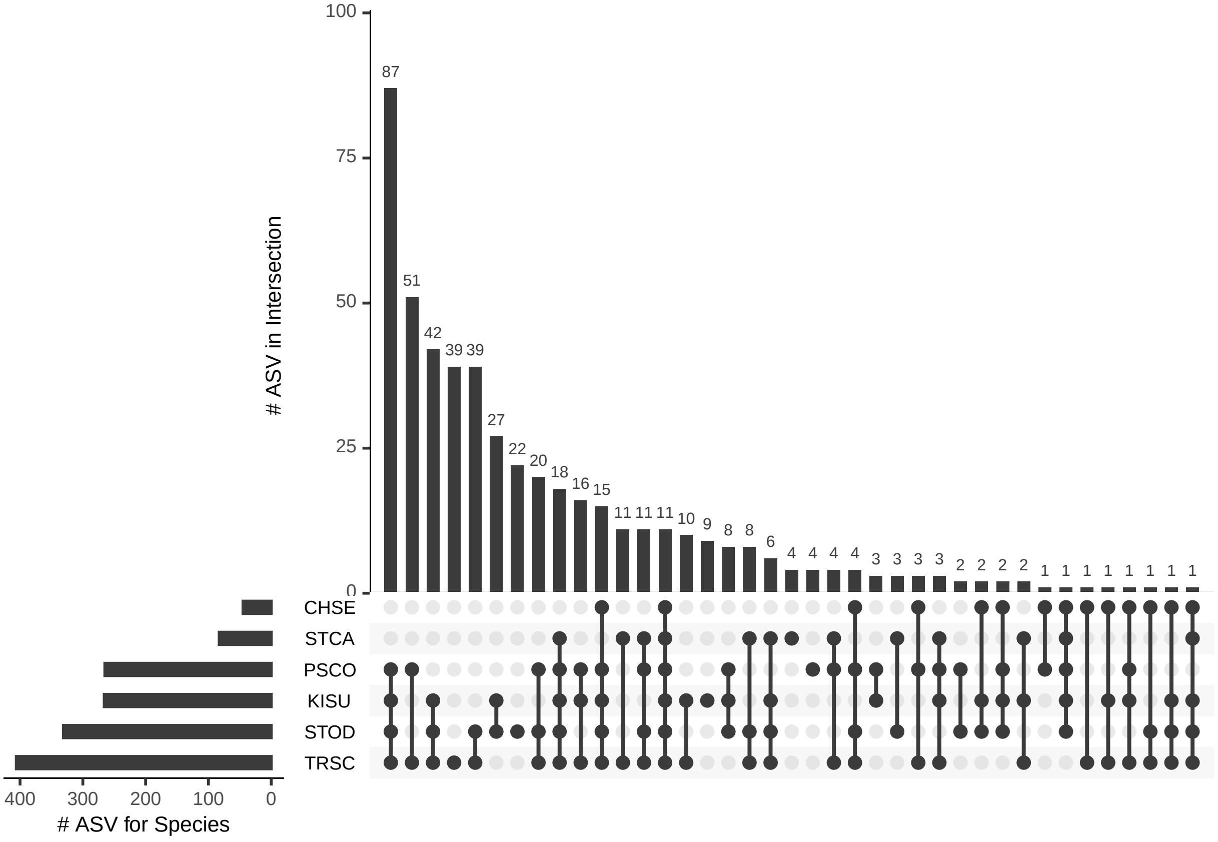

```
#without numbers over bars
#upset(newdata, nsets=6, order.by="freq", mainbar.y.label = "# ASV in Intersection", sets.x.label = "# 

data = read.csv('18S.OTU_site_table.0_1.txt', na.strings="Not Applicable")#install packages (can skip i
newdata <- one_hot(as.data.table(data))#create the visualization
#with numbers over bars
upset(newdata, nsets=3, order.by="freq", mainbar.y.label = "# ASV in Intersection", sets.x.label = "# AS
```

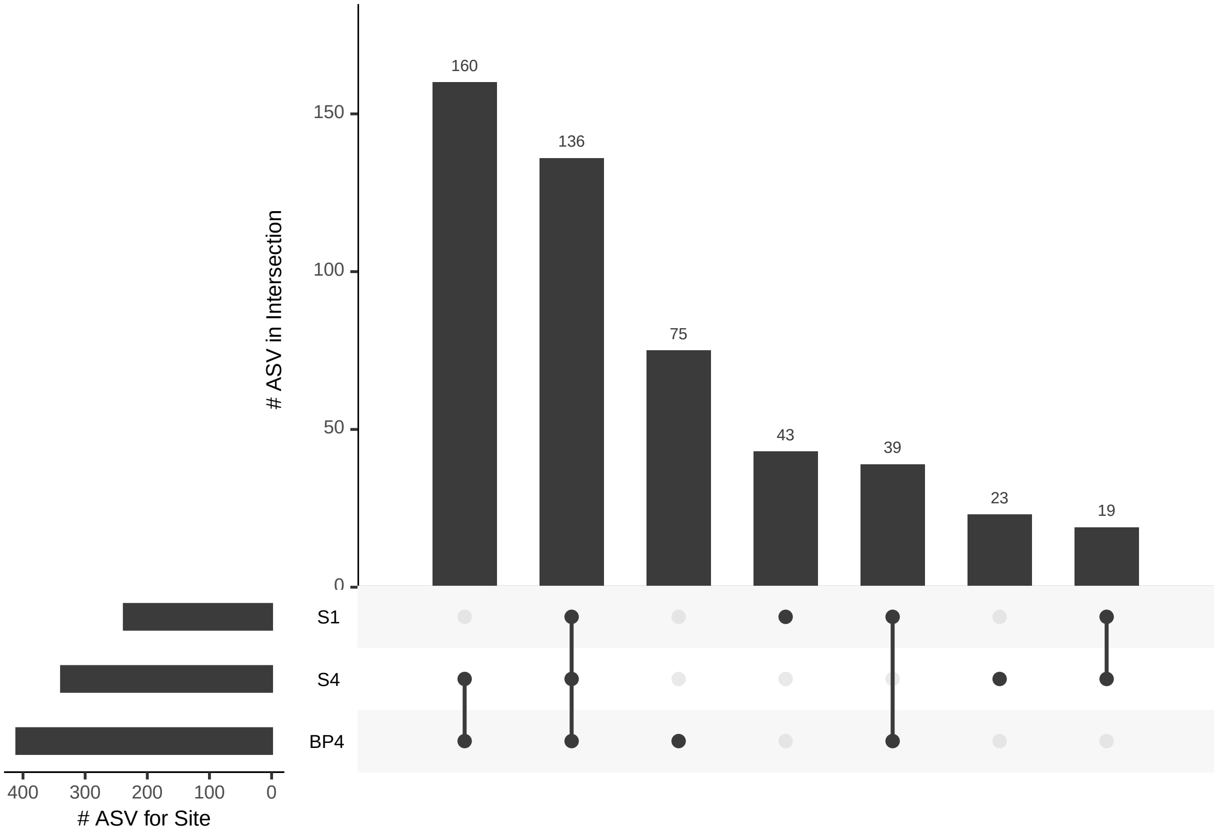


```
#without numbers over bars
#upset(newdata, nsets=3, order.by="freq", mainbar.y.label = "# ASV in Intersection", sets.x.label = "# 
```
